# Patient safety in inpatient mental health settings: a systematic review

**DOI:** 10.1136/bmjopen-2019-030230

**Published:** 2019-12-23

**Authors:** Bethan Thibaut, Lindsay Helen Dewa, Sonny Christian Ramtale, Danielle D'Lima, Sheila Adam, Hutan Ashrafian, Ara Darzi, Stephanie Archer

**Affiliations:** 1 NIHR Imperial Patient Safety Tranlsational Research Centre, Department of Surgery and Cancer, Imperial College London, London, UK; 2 Centre for Behaviour Change, Department of Clinical, Educational and Health Psychology, University College London, London, UK; 3 Department of Public Health and Primary Care, University of Cambridge, Cambridge, Cambridgeshire, UK

**Keywords:** patient safety, mental health, inpatient settings, systematic review

## Abstract

**Objectives:**

Patients in inpatient mental health settings face similar risks (eg, medication errors) to those in other areas of healthcare. In addition, some unsafe behaviours associated with serious mental health problems (eg, self-harm), and the measures taken to address these (eg, restraint), may result in further risks to patient safety. The objective of this review is to identify and synthesise the literature on patient safety within inpatient mental health settings using robust systematic methodology.

**Design:**

Systematic review and meta-synthesis. Embase, Cumulative Index to Nursing and Allied Health Literature, Health Management Information Consortium, MEDLINE, PsycINFO and Web of Science were systematically searched from 1999 to 2019. Search terms were related to ‘mental health’, ‘patient safety’, ‘inpatient setting’ and ‘research’. Study quality was assessed using the Hawker checklist. Data were extracted and grouped based on study focus and outcome. Safety incidents were meta-analysed where possible using a random-effects model.

**Results:**

Of the 57 637 article titles and abstracts, 364 met inclusion criteria. Included publications came from 31 countries and included data from over 150 000 participants. Study quality varied and statistical heterogeneity was high. Ten research categories were identified: interpersonal violence, coercive interventions, safety culture, harm to self, safety of the physical environment, medication safety, unauthorised leave, clinical decision making, falls and infection prevention and control.

**Conclusions:**

Patient safety in inpatient mental health settings is under-researched in comparison to other non-mental health inpatient settings. Findings demonstrate that inpatient mental health settings pose unique challenges for patient safety, which require investment in research, policy development, and translation into clinical practice.

**PROSPERO registration number:**

CRD42016034057.

**Strengths and limitations of this study**

- This is the first review to examine patient safety within inpatient mental health settings that uses robust systematic methodology.
- The use of a robust patient safety taxonomy provides a comprehensive list of all incident types and resulted in a wide coverage of publications in terms of setting, country and population.
- This review only included peer-reviewed studies with primary data.
- The last systematic literature search was conducted on 27 June 2019, meaning that literature published since this date will not have been included.

## Introduction

Patient safety has been defined as the ‘avoidance, prevention and amelioration of adverse outcomes or injuries stemming from the process of healthcare’.^1^ Those receiving care in inpatient mental health settings face similar risks (eg, medication errors) to patients in other areas of healthcare. In addition, some of the unsafe behaviours associated with serious mental health problems (eg, self-harm), and the measures taken to address these (eg, restraint), may result in further risks to patient safety.^2–6^ There may also be a tension between maximising patient safety and maintaining patient autonomy. Inpatient services will often include patients who are experiencing high levels of mental distress and are therefore at greatest risk.

While mental health research has focused on components of quality of care, published research lacks focus on the science of patient safety^7–9^; the stigma and discrimination associated with mental health problems may contribute to this relative neglect.^7^ Only two reviews have examined patient safety in a mental health context and described factors that influence patient safety.^7 10^ These reviews highlighted the complexity of patient safety in mental health, including the importance of wider organisational safety culture. While these reviews offer important insights into this complex topic, only a small number of specific patient safety incidents and concepts were examined. As such, the current breadth and depth of patient safety research in inpatient mental health settings is unknown.

The review presented here is exploratory in nature; building on previous reviews, we aimed to report an overview of the existing research base on patient safety in inpatient mental health settings. We also aimed to critically reflect on quality and methods used in included studies in the field.^11^ In addition to our original protocol,^11^ we aimed to collate, describe and construct the main research categories, allowing for an easily accessible reference index.

## Search strategy and selection criteria

A systematic search was developed in line with the Preferred Reporting Items for Systematic Reviews and Meta-Analyses (PRISMA) guidelines.^12^ The protocol for this systematic review has been published elsewhere.^11^

Six databases were searched: Embase, Cumulative Index to Nursing and Allied Health Literature (CINAHL), Health Management Information Consortium (HMIC), MEDLINE, PsycINFO and Web of Science. The search was originally conducted on 5 April 2016 and then updated on 27 June 2019 using a comprehensive list of search terms (n=343) related to ‘mental health’ (n=73), ‘patient safety’ (n=206), ‘inpatient setting’ (n=13) and ‘research’ (n=51); see online supplementary files 1 and 2 for full search criteria and terms. The search terms included in the ‘patient safety’ facet were based on the National Reporting and Learning System (NRLS) taxonomy for England and Wales^13^ to ensure all incident types were identified in the search. A Google Scholar search using the main search terms was also conducted; it was originally anticipated that the first 20 pages of Google scholar would need to be screened against criteria,^11^ but screening stopped at five pages as no new publications were retrieved. Similarly, we had anticipated hand-searching references of all included papers within the review. However, due to the large number of papers included in the review, only the reference lists of the two existing systematic reviews were searched for additional references.

10.1136/bmjopen-2019-030230.supp1 Supplementary data

10.1136/bmjopen-2019-030230.supp2 Supplementary data

Five reviewers (BT, CR, LD, DD and SAr) screened all titles against the inclusion and exclusion criteria, with 10% independently screened by a second reviewer (split equally between BT, CR, LD, DD and SAr). Full definitions and descriptions of these criteria can be found in online supplementary file 1 and the protocol published elsewhere.^11^ Inclusion and exclusion criteria were developed over several iterative rounds among the research team to ensure consistency between reviewers (online supplementary file 1). Any disagreements between reviewers were resolved through discussion and an overall consensus was obtained. Agreement between reviewers was calculated using Cohen’s kappa,^14^ which is a widely accepted measure of inter-rater reliability.^15 16^ Full-text papers were assessed for inclusion by two reviewers from the research team (BT and one other from CR, LD and SAr); a third reviewer (DD) was consulted if necessary.

Inclusion criteria:

- Population: mental health inpatients;
- Intervention/outcomes: patient safety outcomes;
- Setting: inpatient setting;
- Comparators: no restriction;
- General inclusion criteria: empirical peer-reviewed studies with a clear aim or research question, that used primary data and written up in the English language between 1 January 1999 and 27 June 2019 (in line with the publication of the Institute of Medicine’s report ‘To Err is Human: Building a Safer Health System’).^17^

Exclusion criteria:

- Population: centres on physical healthcare patients;
- Intervention/outcomes: patient safety was not the central aim, research question or outcome
- Setting: amalgamation of data from inpatient and outpatient settings (where inpatient sample cannot be separated out); primary care, outpatient mental health services, community or social care settings and risk assessment tool reliability/validity checks;
- Comparators: no restrictions;
- General exclusion criteria: secondary data, not in English language, protocols, editorials, commentaries/clinical case reviews/‘snapshot’ studies of a patient group, book chapters, conference abstracts, audits, dissertations, epidemiological studies and reviews.

## Quality assessment

Quality assessment was performed to give an overview of the methodological rigour of included studies and to support readers’ interpretation of the literature. Publications were not excluded based on poor quality because the review was purposively exploratory and all-encompassing. Quality was assessed by four reviewers (BT, CR, LD and SAr) using the tool derived by Hawker *et al*,^18^ to allow appropriate assessment of the wide variety of studies included in this review. The checklist by Hawker *et al* evaluates nine domains: 1) abstract/title; 2) introduction and aims; 3) method and data; 4) sampling; 5) data analysis; 6) ethics and bias; 7) results; 8) transferability and generalisability and 9) implications and usefulness. For each study, the nine domains were assessed using one of four quality categories: very poor (10 points), poor (20 points), fair (30 points) and good (40 points). The scores for each study were then summed and divided by nine to get an average score.

## Data extraction

Data were extracted by five reviewers (BT, CR, LD, DD and SAr) using a standardised form that included study design information, participant characteristics, intervention description and patient safety outcomes. Extractions were compared within the research team to ensure reliability. Only published data were extracted; study authors were contacted only for confirmation or information clarity. If the contact attempt was unsuccessful, the article was assessed in its current form.

## Data synthesis

Studies were grouped into research categories through consensus. First, four research team members (BT, CR, LD and SAr) individually re-read the included full-text publications and assigned each one based on the main topic area (eg, aggression). Second, each assigned topic area was checked by another team member to ensure reliability. Third, topic areas were grouped into broader research categories (eg, interpersonal violence) that best described the patient safety focus for easier navigation of the literature. Finally, these categories and the related subcategories (initially called topic areas) from the previous stage were finalised after group discussion and consensus was reached. This was to ensure mutual exclusivity and appropriate definition (table 1 and online supplementary file 3). Where data allowed, meta-analysis was performed applying a random-effects model, specifically calculating pooled prevalence considering both between-study and within-study variances that contributed to study weighting. Pooled values and 95% CIs were computed and represented on forest plots. Statistical heterogeneity was determined by the I^2^ statistic, where <30% is low, 30%–60% is moderate and >60% is high. Analyses were performed using Stata V.15 (StataCorp, College Station, Texas, USA).

10.1136/bmjopen-2019-030230.supp3 Supplementary data

**Table 1:** Overview of study characteristic identified within each category

Category	Subcategory	Category definition	Number of studies	Countries	Number of studies using staff participants	Number of studies using patient participants	Total number of participants	Settings (number of studies conducted in each setting)
Interpersonal violence	Aggression Violence Challenging behaviour Violence and aggression Critical incidents Conflict Sexual Assault Agitation	Behaviours or events that are considered hostile with the intent to cause harm, including violence, aggression and conflicts. This also encompasses sudden emergency incidents that require management.	115	UK-31 USA-20 Australia-9 Canada-7 The -6 Sweden-7 Taiwan-4 South Africa-2 Switzerland-2 India-3 Italy-2 Turkey-3 Europe-2 New Zealand-1 South Korea-1 Finland-3 Greece-1 Spain-1 Hong Kong-1 Israel-2 Nigeria-1 Norway-2 Denmark-1 Japan-1 Germany-1 Slovakia-1	52 22 mixed	39 22 mixed 1 family member of patients 1 N/A	20 066 (excl. missing data)	Psychiatric inpatient wards/facilities- 73 Forensic inpatient facilities-22 Long-term care/nursing homes-13 Specialised research unit-1 Mixed-6
Coercive interventions	Restraint Seclusion Attitudes to coercion Seclusion and restraint Containment Process of coercion Alternative interventions Shielding Conflict Personal factors	Techniques for managing patient behaviour that are applied without consent, for the safety of the patient and others. These include seclusion, restraint and containment.	99	UK-31 Finland-7 USA-8 The Netherlands-5 Australia-5 Canada-7 Norway-4 Germany-2 Sweden-3 Japan-2 Mixed-5 New Zealand-2 Europe-1 China-1 Switzerland-3 South Korea-1 India-2 Brazil-1 Denmark-1	36 29 mixed	34 29 mixed	59 732 (excl. missing data)	Psychiatric inpatient wards/facilities- 74 Forensic inpatient facilities-13 Long-term care/nursing homes-3 Mixed-8 Health board-1
Safety culture	Process Culture Policy Building therapeutic relationships Patient/family engagement	The organisational attitudes, beliefs and values concerning safety. This encompasses the policies and procedures within the healthcare organisation in relation to safety.	49	UK-12 Australia-10 USA-5 Sweden-4 Finland-4 Canada-2 Ireland-2 The Netherlands-1 Greece-1 Italy-1 Germany-1 Belgium-2 Taiwan-1 Europe-1 Iran-2	33 13 mixed	3 13 mixed	59 420 (excl. missing data)	Psychiatric inpatient wards/facilities- 36 Forensic inpatient facilities-8 Long-term care/nursing homes-1 Mixed-4
Harm to self	Self-harm Suicidal behaviour Self-neglect	The ways in which the healthcare system attempts to prevent, mitigate or manage deliberate behaviours displayed by patients that are intended to cause harm or death to themselves.	36	USA-11 UK-8 Ireland-3 Norway-4 The Netherlands-2 Sweden-2 Taiwan-2 Australia-1 Japan-2 Belgium-1	16 3 mixed	17 3 mixed	3631 (excl. missing data)	Psychiatric inpatient wards/facilities- 29 Forensic inpatient facilities-3 Long-term care/nursing homes-2 Learning disability homes-1 Mixed-1
Safety of the physical environment	Security Environmental design Transitions of care Patient distribution Staffing Ligatures	The factors related to the physical environment of the healthcare setting that could impact on safety. This includes ligature points, staffing, security (door locking) and patient distribution.	21	UK-6 The Netherlands- 3 USA-4 Australia-4 Germany-2 Mixed-1 Sweden-1	6 8 mixed	7 8 mixed	3140 (excl. missing data)	Psychiatric inpatient wards/facilities- 17 Forensic inpatient facilities-1 Long-term care/nursing homes-3
Medication safety	Adverse events Medication administration Medication management Medication dispensing Adherence Substance use	Mistakes made at any stage of the medication use process, from preparation, to administration and recording. This includes adverse drug events (or injuries that are the result of a drug-related intervention) and issues surrounding drug/alcohol use.	17	UK-7 Turkey-1 Spain-2 The Netherlands-1 Croatia-1 Germany-1 Denmark-1 Canada-2 Mixed-1	9 1 mixed	7 1 mixed	2396 (excl. missing data)	Psychiatric inpatient wards/facilities- 13 Forensic inpatient facilities-2 Long-term care/nursing homes-1 Mixed-1
Unauthorised leave	Absconding Wandering	The act of a patient leaving the healthcare setting without the knowledge or consent of staff/carers. This can be either with (absconding) or without intent (wandering) on the part of the patient.	11	UK-4 Australia-3 USA-1 Canada-1 Italy-1 Indonesia-1	3 1 mixed	7 1 mixed	978 (excl. missing data)	Psychiatric inpatient wards/facilities- 10 Long-term care/nursing homes-1
Clinical decision making	Incident management Risk assessment Diagnosis	Incorrect diagnoses, risk assessments and other decision making processes of healthcare staff that impact on the safety of a patient.	9	USA-3 UK-3 Canada-1 Greece-1 The Netherlands-1	6	3	529	Psychiatric inpatient wards/facilities- 8 Forensic inpatient facilities-1
Falls	Falls Injuries	Falling events that lead to the unintentional harm of an individual. This includes trips and injuries such as fractures.	6	USA-3 Sweden-2 Israel-1	3	3	180 (excl. missing data)	Psychiatric inpatient wards/facilities- 5 Long-term care/nursing homes-1
Infection prevention and control	Infection prevention and control	Preventing harm caused by infection to patients and health workers.	1	Taiwan-1	1	0	13	Psychiatric inpatient wards/facilities- 1

## Patient and public involvement

Patients and the public were not involved in this study.

## Results

**Figure 1:**
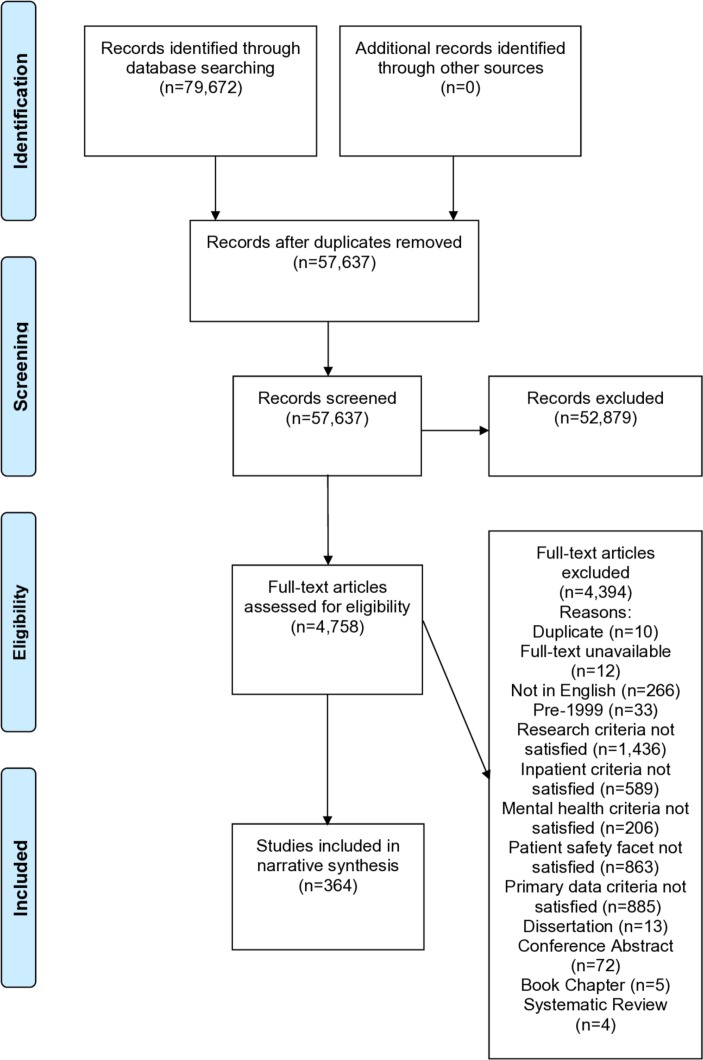
Flow chart of studies.

The search resulted in 79 672 records (figure 1) and reduced to 57 637 after de-duplication. Titles and abstracts were screened and excluded if they did not satisfy inclusion criteria (BT, CR, LD, DD and SAr). Ten per cent were then screened (n=5763) by a second independent reviewer (split equally between BT, CR, LD, DD and SAr), in line with guidance on improving decision making by including more than one person in this process^19^; good agreement was found between pairs of reviewers (κ=0.72). A total of 4758 publications were subjected to full-text review (BT, CR, LD and SAr). Two reviewers independently screened the full-text articles against inclusion criteria (BT, CR, LD and SAr). The third reviewer (DD) was consulted 59 times. Substantial agreement was reached (κ=0.64). From the full-text review, 4394 publications were excluded. Three hundred and sixty-four publications met the inclusion criteria and data were extracted (online supplementary file 4).

10.1136/bmjopen-2019-030230.supp4 Supplementary data

### Study characteristics

Table 1 provides an overview of the study characteristics. The publications spanned 5 continents and 31 countries. The three countries contributing the greatest number of studies were the UK (n=102), the USA (n=55) and Australia (n=32). The included studies collected data from over 150 000 participants. Studies included staff (n=165; 45%), patients (n=120; 33%) and a mixture of staff, patients and/or carers (n=77; 21%). Only one study focused solely on patient family members (<1%). Most studies were quantitative in nature (n=192; 53%), just over a third were qualitative (n=133; 37%) and a small proportion used mixed methodology (n=39; 11%). Studies were conducted in a variety of settings comprising: psychiatric inpatient wards/facilities (n=266;73%), forensic inpatient facilities (n=50; 14%), long-term care/nursing homes (n=25; 7%), mixed inpatient settings (n=20; 5%), a learning disability unit (n=1; <1%), a health board (n=1; <1%) and a specialised research unit (n=1; <1%). More information about the study designs used is included in online supplementary file 4.

### Quality assessment

Most research was assessed as ‘fair’ quality (n=251; 69%), 86 (24%) papers were assessed as ‘good’ quality and 26 (7%) were assessed as ‘poor’ quality. None was assessed as ‘very poor’ quality. Studies rated as ‘poor’ mainly did not discuss ethical considerations, potential biases or give sample or setting characteristics. For example, they did not consider recruitment strategies, sample demographics or provide detailed information on the research setting. All ‘good’ studies provided setting and sampling information to allow for replicability. In addition, ‘good’ studies provided detail on data analysis justification, more thorough literature reviews to place the study in context and had clear research aims/objectives. Online supplementary file 5 includes a table showing the breakdown of the quality domain scores for each paper.

10.1136/bmjopen-2019-030230.supp5 Supplementary data

### Synthesis

Ten research categories were identified: interpersonal violence, coercive interventions, safety culture, harm to self, safety of the physical environment, medication safety, unauthorised leave, clinical decision making, falls and infection prevention and control. Within these categories 46 subcategories were identified (table 1).

### Interpersonal violence

Interpersonal violence was the largest category (n=116; 32%). Studies were primarily concerned with the prevalence, management and prevention of violent and aggressive behaviours (n=75). The pooled prevalence for physical violence was 43.2% (95% CI 0.37 to 0.49) with high heterogeneity (I^2^ 100.0%) in 20 studies^20–39^ (online supplementary file 6). The pooled prevalence for verbal aggression was 57.4% (95% CI 0.34 to 0.81) with a high heterogeneity (I^2^ 100.0%) in 10 studies^22–24 26 29 33–36 40^ (online supplementary file 6).

10.1136/bmjopen-2019-030230.supp6 Supplementary data

One study examined the characteristics of aggressive incidents by ward type,^41^ and two studies identified correlates of violence.^42 43^ One study explored how patients described their aggressive behaviours.^44^ Twenty-four studies evaluated intervention effectiveness (eg, staff training and medication use) to reduce violent and aggressive behaviours, with most finding significant improvements,^45–65^ two reporting negative outcomes^66 67^ and one reporting mixed findings.^68^ The general management of violent and aggressive behaviours was explored in 15 studies.^22 25 29 30 69–79^ Two studies explored the ways in which treatment can affect violence incidence.^31 80^

Twenty-seven studies explored violent and aggressive incident experiences in staff,^81–96^ patients,^97–99^ mixed groups^100–106^ and patient family members.^27^ Five studies explored the risk factors associated with verbal and physical aggression.^35 37 107–109^ Three studies explored mental health nurses’ perspectives on the response to violent situations in high secure environments: one on the psychological impact of physical assault on staff,^110^ one on making violence risk assessments in imminent violent situations^111^ and one on the decline of incident reports.^112^ One study explored the link between aggressive behaviour and levels of burnout in staff^113^ and one study looked at the role of social support for staff following a violent incident.^32^

Ten studies^114–123^ examined challenging behaviour and techniques, such as de-escalation and communication strategies, which could be used to manage this; seven studies found techniques that were effective.^114–120^ A further four studies investigated conflict behaviour management techniques employed by staff^124–126^ and patients^127^; techniques used in the two intervention studies were effective in reducing conflict.^126 127^ Staff and patient attitudes towards critical incidents were the focus of four qualitative studies^128–131^; a further three studies focused on maintaining the psychological safety of patients who had experienced physical or sexual assault during an inpatient stay^132^ and outside of healthcare.^133 134^ Finally, one study explored an acupressure intervention to reduce agitation, which was found to be effective.^135^

### Coercive interventions

Coercive interventions were the focus of 98 papers (27%). Most studies (n=42) reported on restraint and seclusion techniques. The pooled prevalence for coercive interventions was 47.8% (95% CI 0.38 to 0.57) with high heterogeneity (I^2^ 100.0%) in 12 studies^136–147^ (online supplementary file 6).

Studies explored staff,^148–157^ patient^147 158–165^ and mixed groups’^166–173^ views and experiences of seclusion and restraint. Nine studies focused on the processes surrounding seclusion and restraint.^136 137 174–180^ A further 16 studies evaluated interventions to reduce seclusion and restraint, with 13 finding significant decreases in rates of use,^146 181–192^ one reporting an increase^193^ and one reporting increased levels of knowledge about the topic area.^194^ Four studies examined prevalence, trends and preventative factors^138 195–197^; one found that 45% of patients were subjected to restraint,^138^ and another found that restraint and seclusion declined over time.^197^ One study explored the context in which seclusion and restraint had taken place.^198^ Two studies found preventative factors of mechanical restraint to be staff education and increased patient involvement.^195 196^ The training of staff in techniques for seclusion and restraint were explored in two studies^199 200^ and one study examined adverse events resulting from restraint and seclusion.^201^ Other studies explored staff and patient views of containment measures,^202–205^ Maori views of initiatives to reduce/prevent seclusion,^206^ the process of shielding (segregation under staff supervision),^207^ conflict management^208^ and alternative interventions.^209^

Thirty-two studies focused on coercion; one study examined prevalence of coercive measures^141^ and one study explored how the experience of staff might contribute to the use of restrictive practices.^210^ The attitudes of staff,^142 144 211–222^ patients^145 223–226^ and mixed groups^143 168 227–230^ towards coercion were explored in 25 studies, and 5 studies examined the process of coercive interventions^139 140 231 232^ and rules of engagement in caring for aggressive patients.^233^

### Safety culture

Safety culture included studies on processes, culture and policy across 49 papers (13%). Eighteen studies concerned safety-related organisational processes. Eleven of these investigated processes of treatment or care that healthcare staff undertake; processes included limit-setting and clothing restrictions,^234–240^ risk assessment^241–243^ and nursing handover.^244^ Two investigated errors and reporting^245 246^ and a further two studies explored staff and patient perceptions of safety when involved in treatment processes.^247 248^ Two studies focused on change implementation.^249 250^ One study focused on the role of training.^251^ Safety culture was featured in 18 publications relating to the management of serious incidents,^252–254^ stress and burnout,^255–257^ staff^258^ and patient perspectives of safety^259–263^ and communication^264^; there were also three papers that explored safety culture more generally.^265–267^ A further two evaluated the TeamSTEPPS (Team Strategies and Tools to Enhance Performance and Patient Safety) programme^268 269^ and both found significant clinical benefits in reducing seclusion and improving team functioning. One paper looked at the barriers and facilitators to implementing a Safewards intervention.^270^ With regard to policy, eight studies concerned safety policies related to: observation,^271 272^ risk assessment,^273 274^ treatment,^275^ safeguarding,^276^ security^277^ and ergonomic improvement.^278^ Two papers focused on the role of patient and family engagement in safety,^279 280^ and two papers focused on how to build better therapeutic relationships to improve patient safety.^281 282^

### Harm to self

Three subcategories centred on harmful behaviours: self-harm, suicidal behaviour and self-neglect (n=36; 10%). Half of the studies (n=18) focused on self-harm. One paper explored the prevalence of self-harm.^283^ Two studies explored risk factors for self-harm which included use of psychotropic medication.^284 285^ Eight papers explored staff attitudes and experiences of managing self-harm,^286–293^ and three explored patient experiences.^294–296^ Three intervention studies focused on training,^297^ therapy^298^ and observation^299^; all reported a reduction in self-harm behaviours and a further intervention focusing on training for staff resulted in positive attitude towards self-harm patients, greater closeness and improved self-efficacy.^300^ Of the 17 papers that centred on suicidal behaviours, five studies investigated the observance of risk factors^301–305^ and three intervention studies found significant reductions in suicide-related behaviours and cognitions.^306–308^ An additional eight papers explored staff,^309–312^ patient^313 314^ and both staff and patient^315 316^ views and attitudes towards suicidal behaviour. One study looked at the acceptability of an intervention to reduce suicide.^317^ Finally, one study explored types of self-neglect behaviours in patients with dementia, including functional difficulties, serious hygiene problems and safety risks.^318^

### Safety of the physical environment

The safety of the physical environment category included 21 papers (6%). Seven studies investigated security measures (eg, door locking).^319–325^ Five studies investigated the effects of the physical environmental design on the safety of treatment settings.^326–330^ Three studies focused on safety during transitions of care,^331–333^ with most based in dementia care settings. Three studies examined how the location of patients within the hospital setting can impact on safety, focusing on topics such as: privacy, female-only wards and the use of segregated or combined wards/units.^334–336^ The remaining three studies concerned staffing levels^337 338^ and ligature points.^339^

### Medication safety

The medication safety category included 17 publications (5%). Five studies focused on adverse events, and examined: antipsychotics side effects,^340^ how best to manage the effect of psychotropics on long QT segments,^341^ antidepressants^342^ and medication error reporting.^343 344^ Three studies investigated errors occurring in broader medication management processes^345–347^ and a further five studies focused on medication administration specifically.^348–352^ The only intervention study aiming to reduce these errors found that a new medication dispensing system did not have any significant impact on patient safety.^353^ Two studies explored staff perceptions of illicit substance use.^354 355^ One further study described the development of a medication adherence intervention for patients who are prescribed mood-stabilising medication for bipolar disorder.^356^

### Unauthorised leave

Unauthorised leave included 11 publications (3%). Three explored the patient experience of absconding, specifically relating to patient perspectives of treatment and involuntary commitment.^357–359^ One study explored staff perspectives of absconding management techniques,^360^ and two studies evaluated interventions to reduce absconding rates; both were found to be effective.^361 362^ Two studies focused on wandering behaviour in women with dementia, linking wandering to physical environment factors, such as light, sound, crowding^363^ and falls.^364^ The pooled prevalence of wandering behaviour was 50.2% (95% CI 0.49 to 0.52) with high heterogeneity (I^2^ 78.0%) in two studies^363 364^ (online supplementary file 6). The final three studies examined the consequences^365 366^ and security measures surrounding absconding.^367^

### Clinical decision making

Clinical decision making accounted for 2% of the included publications (n=9). These publications covered the development of clinical judgements and decisions relating to incident management, risk assessment and diagnosis. Two studies explored the cultural differences considered by clinicians in the diagnosis of African-American patients.^368 369^ Clinical decisions on whether to engage in seclusion and/or restraint were explored in five studies^370–374^ and two studies explored the variation in assessment and prediction of violence between staff and settings.^375 376^

### Falls

Publications on falls formed the second smallest category within the review (n=6; 1%). Studies in this category focused on fall prevalence, falls experienced by older psychiatric inpatients with dementia and prevention/harm reduction techniques. A recurring risk factor for falling was found to be medication use.^377–379^ Two fall prevention intervention studies did not identify significant benefits,^380 381^ and one study explored barriers and facilitators to such interventions.^382^

### Infection prevention and control

One paper (<1%) focused on staff experiences of infection prevention and control in psychiatric clinical settings.^383^

## Discussion

### Main findings

This is the first review to examine patient safety within inpatient mental health settings that uses robust systematic methodology. As a result, we have identified ten research categories: interpersonal violence, coercive interventions, safety culture, harm to self, safety of the physical environment, medication safety, unauthorised leave, clinical decision making, falls and infection prevention and control. In addition, we have been able to include a meta-analysis of incidence and prevalence of aggression (verbal and physical), coercive intervention and wandering behaviour as well as providing an easily accessible reference index of literature in the inpatient mental health and patient safety domain. Previous reviews on this topic had focused on collating the literature on a restricted number (n=8) of predefined patient safety incidents (eg, violence and aggression),^7^ or the concept of patient safety in inpatient mental health setting more broadly (eg, organisation management).^10^ As such, the findings presented here offer a contemporary view of the breadth and depth of patient safety research in inpatient mental health settings.

We were concerned to see that only 364 papers were identified as a result of our comprehensive search. Although this can be seen as a large number of publications for a systematic review, it is a relatively small number to cover the care of a wide range of patients in a variety of inpatient mental health settings over a 20-year period (around 18 papers per year across all countries). While important work not meeting our inclusion criteria (eg, quality improvement initiatives and studies using secondary analysis of data) may have focused on patient safety in mental health, the lack of prospective peer-reviewed publications adds to the ongoing discussion surrounding the disparity in research focusing on patient safety in physical and mental healthcare.^384^ In addition, there was a paucity of high-quality research in the area; just over two-thirds of the studies were considered to be ‘fair’, and only nine studies included in the meta-analysis were deemed ‘good’. ‘Poor’ studies most frequently did not have clear research aims and objectives, study details were missing (eg, sample(s) and setting(s) used) and they failed to discuss issues related to ethical and researcher bias. Some qualitative studies explored both staff and patients’ perspectives, an important aspect of research, particularly when safety in this context is a relatively new area of knowledge. However, there was limited intervention research, particularly randomised controlled trials (RCTs). In the RCTs that were identified, sample sizes were mostly small.

The findings from the review also challenged our expectations in terms of breadth and depth of research. For example, we expected to find many publications on the prevention of suicide within inpatient settings due to the severity of harm. However, only one study that met inclusion criteria discussed suicide in relation to ligature points.^339^ A scoping review also found only this one study, suggesting a consistency of approach.^385^ This indicates that while the prevention of suicide is a well-established aspect of patient safety, it is now reviewed routinely, using pre-existing and secondary data, rather than through empirical research.

We also found little research focusing on the concepts required for system level reform,^386^ which was disappointing seeing as some improvements have been made in physical healthcare.^387^ For example, in line with research in the physical health domain,^388 389^ we were hoping to find several studies exploring how patient and family engagement in care can promote patient safety.^390^ However, only two studies identified in our review had patient/family engagement as their primary focus.^279 280^ Similarly, we were expecting to identify literature investigating the lack of integration between physical and mental healthcare and the impact it has on patient safety.^391^ However, the need to prevent and manage co-existing physical ill health was not identified in the review. This is surprising as patients with serious mental illness are twice as likely to die prematurely and much more likely to develop long-term conditions or become disabled, as those without serious mental illness.^392^ This patient group is also vulnerable to asphyxiation during restraint and rapid tranquilisation.^393^

Research on medication safety in inpatient mental health settings was also limited in this review. This was unexpected considering two-thirds of patients with mental health problems are prescribed medication and are therefore potentially at risk of experiencing a medication safety incident. Research pertaining to falls was also limited, contrasting with patient safety research within the physical health domain that includes a focus on slips, trips and falls.^394^

### Strengths and limitations

We used a robust patient safety taxonomy to provide a comprehensive list of all incident types. This resulted in a wide coverage of publications in terms of setting, country and population. We systematically searched, screened, extracted and appraised data. As a result, our systematic review draws together all relevant literature concerning patient safety within inpatient mental health settings, simultaneously operating as an index resource for clinicians and researchers.

There were several limitations. We used the definition of patient safety given by Vincent^1^ to guide this review. While this is more nuanced than the original Institute of Medicine definition of safety ‘freedom from accidental injury’^395^ and is widely accepted within the patient safety movement, it may be that a more suitable definition reflects the specific challenges within the inpatient mental health setting.^396^ This review only included peer-reviewed studies with primary data. Therefore, literature using secondary data such as pre-existing datasets and data from internal audits was excluded as it did not fulfil the criteria of being a prospective research study with clear research aims.^397^ For example, data examined by the National Confidential Inquiry into Suicide and Homicide by People with Mental Illness is collected retrospectively from various sites across the country and would have been excluded from this review.^398^ Moreover, non-peer-reviewed quality improvement reports have also been excluded. The decision was made to only include peer-previewed studies with primary data due to (i) the large number of potential publications in this area, (ii) the need to define the scope and focus of the review and (iii) the need for specificity as well as sensitivity. The investigation of patient safety in mental health inpatient settings using secondary data or in non-peer-reviewed formats is an avenue for additional systematic reviews.

The last systematic literature search was conducted on 27 June 2019, meaning that literature published since this date will not have been included. In order to further build on the review published here, a *living* systematic review (an ongoing updated summary of high-quality research)^399^ would continue to identify relevant literature in this area. In terms of the meta-analysis, there was expected statistical and methodological variability in studies, particularly for physical and verbal aggression. It is possible that this was due to the inclusion of different definitions of aggression, time periods and type of inpatient setting. In relation to the agreement between reviewers (including the use of recommended piloting of inclusion and exclusion criteria within the screening stage),^400^ inter-rater reliability calculations only achieved substantial agreement (κ=0.61–0.80) at both the title and full-text screening stages. Although higher kappas have been reported in other systematic reviews, a substantial agreement is classified as more than acceptable.^401^

While the research spanned five continents, the UK, the USA and Australia contributed over 50% of the included studies, leading to a potential cultural bias in the body of research identified within the review. We recommend that, where possible, future systematic reviews incorporate manuscripts in languages other than English to establish greater insight into the global literature on patient safety in inpatient mental health settings, with a view to limiting any cultural bias. Similarly, while the removal of publications denoting non-inpatient setting restricted the conclusions to the inpatient setting, issues pertaining to this environment are likely to be different to that of community, primary or social care settings. Additionally, studies were excluded before 1999 to coincide with the release of the Institute of Medicine’s report ‘To Err is Human: Building a Safer Health System’^395^; this may have narrowed the review scope as the historical context was minimised.

### Clinical implications and future research

This review informs academics, clinicians and service providers about the evidence base in the patient safety field within inpatient mental health settings. The findings allow researchers and clinicians to be directed to literature relevant to a given patient safety topic area, a useful starting point when developing practice guidelines.^402^ Similarly, the findings may influence clinical practice, with those implementing interventions or designing service changes being able to easily access the current scientific understanding.

Future research should be informed by patient safety science more broadly and focus on filling the knowledge gaps highlighted in this review, that is, studies that explore (i) systems level improvement, (ii) patient and carer engagement in safety, (iii) suicide prevention across different countries, (iv) the nature of medication safety in inpatient mental health settings and (v) the prevalence and impact of staff to patient violence. These findings support our previous expert consensus study where academic and service user experts agreed that patient-driven research studies were needed.^403^ The limited rigorous research surrounding patient safety within inpatient mental health settings necessitates future studies to: (i) include large inpatient samples relevant to the research design, (ii) perform appropriate intervention testing and (iii) examine safety from different perspectives. It should also focus on high-quality reporting of research, paying particular attention to the area of ethics, sampling and setting characteristics.

## Conclusion

This is the first systematic review to comprehensively examine research on patient safety within inpatient mental health settings. It has drawn together the existing literature and shed light on the gaps in knowledge. Inpatient mental health settings may demonstrate unique patient safety challenges and more research is needed to achieve parity with physical health. Addressing this through a strong body of evidence, informed by patient safety science more broadly, will mean that mental healthcare policy makers are in a better position to address safety issues, and implement robust and evidence-based interventions to improve care.

## Supplementary Material

Reviewer comments

Author's manuscript

## References

[1] Vincent C Patient safety. Edinburgh: Churchill Livingstone, 2006.

[2] Brickell TA, McLean C Emerging issues and challenges for improving patient safety in mental health: a qualitative analysis of expert perspectives. J Patient Saf 2011;7:39–44. 10.1097/PTS.0b013e31820cd78e 21921866

[3] Powell J, Geddes J, Deeks J, et al Suicide in psychiatric hospital in-patients. risk factors and their predictive power. Br J Psychiatry 2000;176:266–72. 10.1192/bjp.176.3.266 10755075

[4] Roy A, Draper R Suicide among psychiatric hospital in-patients. Psychol Med 1995;25:199–202. 10.1017/S0033291700028233 7792356

[5] Gray NS, Hill C, McGleish A, et al Prediction of violence and self-harm in mentally disordered offenders: a prospective study of the efficacy of HCR-20, PCL-R, and psychiatric symptomatology. J Consult Clin Psychol 2003;71:443–51. 10.1037/0022-006X.71.3.443 12795569

[6] Goh SE, Salmons PH, Whittington RM Hospital suicides: are there preventable factors? profile of the psychiatric hospital suicide. Br J Psychiatry 1989;154:247–9. 10.1192/bjp.154.2.247 2775953

[7] Brickell TA, Nicholls TL, Procyshyn RM Patient safety in mental health. Canadian Patient Safety Institute, 2000.

[8] Vincent GM Psychopathy and violence risk assessment in youth. Child Adolesc Psychiatr Clin N Am 2006;15:407–28. 10.1016/j.chc.2005.12.001 16527663

[9] Taylor TL, Killaspy H, Wright C, et al A systematic review of the International published literature relating to quality of institutional care for people with longer term mental health problems. BMC Psychiatry 2009;9:55 10.1186/1471-244X-9-55 19735562 PMC2753585

[10] Kanerva A, Lammintakanen J, Kivinen T Patient safety in psychiatric inpatient care: a literature review. J Psychiatr Ment Health Nurs 2013;20:541–8. 10.1111/j.1365-2850.2012.01949.x 22776063

[11] D’Lima D, Archer S, Thibaut BI, et al A systematic review of patient safety in mental health: a protocol based on the inpatient setting. Syst Rev 2016;5:203 10.1186/s13643-016-0365-7 27894331 PMC5126859

[12] Moher D, Shamseer L, Clarke M, et al Preferred reporting items for systematic review and meta-analysis protocols (PRISMA-P) 2015 statement. Syst Rev 2015;4:1 10.1186/2046-4053-4-1 25554246 PMC4320440

[13] NHS Improvement Learning from patient safety incidents, 2014 Available: http://www.nrls.npsa.nhs.uk/patient-safety-data/

[14] Cohen J A coefficient of agreement for nominal scales. Educ Psychol Meas 1960;20:37–46. 10.1177/001316446002000104

[15] McHugh ML Interrater reliability: the kappa statistic. Biochemia Medica 2012;22:276–82. 10.11613/BM.2012.031 23092060 PMC3900052

[16] Belur J, Tompson L, Thornton A, et al Interrater reliability in systematic review methodology: exploring variation in coder decision-making. Soc Med Res 2018.

[17] Donaldson MS, Corrigan JM, Kohn LT To err is human: building a safer health system. 6 National Academies Press, 2000. 25077248

[18] Hawker S, Payne S, Kerr C, et al Appraising the evidence: reviewing disparate data systematically. Qual Health Res 2002;12:1284–99. 10.1177/1049732302238251 12448672

[19] Edwards P, Clarke M, DiGuiseppi C, et al Identification of randomized controlled trials in systematic reviews: accuracy and reliability of screening records. Stat Med 2002;21:1635–40. 10.1002/sim.1190 12111924

[20] Amoo G, Fatoye FO Aggressive behaviour and mental illness: a study of in-patients at Aro neuropsychiatric Hospital, Abeokuta. Niger J Clin Pract 2010;13:351–5. 20857802

[21] Boström A-M, Squires JE, Mitchell A, et al Workplace aggression experienced by frontline staff in dementia care. J Clin Nurs 2012;21:1453–65. 10.1111/j.1365-2702.2011.03924.x 22151034

[22] Chen W-C, Hwu H-G, Wang J-D Hospital staff responses to workplace violence in a psychiatric hospital in Taiwan. Int J Occup Environ Health 2009;15:173–9. 10.1179/oeh.2009.15.2.173 19496484

[23] Daffern M, Mayer M, Martin T Staff gender ratio and aggression in a forensic psychiatric hospital. Int J Ment Health Nurs 2006;15:93–9. 10.1111/j.1447-0349.2006.00408.x 16643344

[24] Ilkiw-Lavalle O, Grenyer BFS Differences between patient and staff perceptions of aggression in mental health units. Psychiat Ser 2003;54:389–93. 10.1176/appi.ps.54.3.389 12610249

[25] Jacob P, Seshadri S, Girimaji SC, et al Clinical characteristics of aggression in children and adolescents admitted to a tertiary care centre. Asian J Psychiatr 2013;6:556–9. 10.1016/j.ajp.2013.08.070 24309872

[26] Lehmann LS, McCormick RA, Kizer KW A survey of assaultive behavior in veterans health administration facilities. Psychiatr Serv 1999;50:384–9. 10.1176/ps.50.3.384 10096644

[27] Raveendranathan D, Chandra PS, Chaturvedi SK Violence among psychiatric inpatients: a victim's perspective. East Asian Arch Psychiatry 2012;22:141–5. 23271582

[28] Ryan EP, Hart VS, Messick DL, et al A prospective study of assault against staff by youths in a state psychiatric hospital. Psychiatr Serv 2004;55:665–70. 10.1176/appi.ps.55.6.665 15175464

[29] Schwartz TL, Park TL Assaults by patients on psychiatric residents: a survey and training recommendations. Psychiatr Serv 1999;50:381–3. 10.1176/ps.50.3.381 10096643

[30] Sukhodolsky DG, Cardona L, Martin A Characterizing aggressive and noncompliant behaviors in a children’s psychiatric inpatient setting. Child Psychiatry Hum Dev 2005;36:177–93. 10.1007/s10578-005-3494-0 16228146

[31] Wystanski M Assaultive behaviour in psychiatrically hospitalized elderly: a response to psychosocial stimulation and changes in pharmacotherapy. Int J Geriatr Psychiatry 2000;15:582–5. 10.1002/1099-1166(200007)15:7<582::AID-GPS152>3.0.CO;2-5 10918337

[32] Kelly EL, Fenwick KM, Brekke JS, et al Sources of social support after patient assault as related to staff well-being. J Interpers Violence 2017;8:088626051773877–79. 10.1177/0886260517738779 PMC6445776 29294965

[33] Niu S-F, Kuo S-F, Tsai H-T, et al Prevalence of workplace violent episodes experienced by nurses in acute psychiatric settings. PLoS One 2019;14:e0211183 10.1371/journal.pone.0211183 30677077 PMC6345477

[34] Danivas V, Lepping P, Punitharani S, et al Observational study of aggressive behaviour and coercion on an Indian acute ward. Asian J Psychiatr 2016;22:150–6. 10.1016/j.ajp.2016.06.004 27520919

[35] Podubinski T, Lee S, Hollander Y, et al Patient characteristics associated with aggression in mental health units. Psychiatry Res 2017;250:141–5. 10.1016/j.psychres.2017.01.078 28161609

[36] van den Bogaard KJHM, Nijman HLI, Palmstierna T, et al Characteristics of aggressive behavior in people with mild to borderline intellectual disability and co-occurring psychopathology. J Ment Health Res Intellect Disabil 2018;11:124–42. 10.1080/19315864.2017.1408726

[37] Pekurinen VM, Välimäki M, Virtanen M, et al Organizational justice and collaboration among nurses as correlates of violent assaults by patients in psychiatric care. Psychiatr Serv 2017;68:490–6. 10.1176/appi.ps.201600171 28142388

[38] Kelly EL, Fenwick K, Brekke JS, et al Well-Being and safety among inpatient psychiatric staff: the impact of conflict, assault, and stress reactivity. Adm Policy Ment Health 2016;43:703–16. 10.1007/s10488-015-0683-4 26377816 PMC4794422

[39] Bilici R, Sercan M, Izci F Levels of the staff's exposure to violence at locked psychiatric clinics: a comparison by occupational groups. Issues Ment Health Nurs 2016;37:501–6. 10.3109/01612840.2016.1162883 27104294

[40] Daffern M, Ogloff J, Howells K Aggression in an Australian forensic psychiatric hospital. J British Forensic Pract 2003;5:18–28. 10.1108/14636646200300024

[41] Sato M, Noda T, Sugiyama N, et al Characteristics of aggression among psychiatric inpatients by ward type in Japan: Using the Staff Observation Aggression Scale - Revised (SOAS-R). Int J Ment Health Nurs 2017;26:602–11. 10.1111/inm.12228 27445160

[42] Ben-Zeev D, Scherer EA, Brian RM, et al Use of multimodal technology to identify digital correlates of violence among inpatients with serious mental illness: a pilot study. Psychiatr Serv 2017;68:1088–92. 10.1176/appi.ps.201700077 28669285 PMC5891222

[43] Bowers L, Allan T, Simpson A, et al Identifying key factors associated with aggression on acute inpatient psychiatric wards. Issues Ment Health Nurs 2009;30:260–71. 10.1080/01612840802710829 19363731

[44] Selenius H, Leppänen Östman S, Strand S Self-Harm as a risk factor for inpatient aggression among women admitted to forensic psychiatric care. Nord J Psychiatry 2016;70:554–60. 10.1080/08039488.2016.1183707 27224513

[45] van de Sande R, Nijman HLI, Noorthoorn EO, et al Aggression and seclusion on acute psychiatric wards: effect of short-term risk assessment. Br J Psychiatry 2011;199:473–8. 10.1192/bjp.bp.111.095141 22016437

[46] Park JS, Lee K Modification of severe violent and aggressive behavior among psychiatric inpatients through the use of a short-term Token economy. J Korean Acad Nurs 2012;42:1062–9. 10.4040/jkan.2012.42.7.1062 23377602

[47] Needham I, Abderhalden C, Meer R, et al The effectiveness of two interventions in the management of patient violence in acute mental inpatient settings: report on a pilot study. J Psychiatr Ment Health Nurs 2004;11:595–601. 10.1111/j.1365-2850.2004.00767.x 15450028

[48] Murphy CJ, Siv AM A one year study of mode deactivation therapy: adolescent residential patients with conduct and personality disorders. Int J Behav Cogn Ther 2007;3:327–41. 10.1037/h0100809

[49] McLaughlin S, Bonner G, Mboche C, et al A pilot study to test an intervention for dealing with verbal aggression. Br J Nurs 2010;19:489–94. 10.12968/bjon.2010.19.8.47638 20505614

[50] Killick S, Allen D Training staff in an adolescent inpatient psychiatric unit in positive approaches to managing aggressive and harmful behaviour: does it improve confidence and knowledge? Child Care Prac 2005;11:323–39. 10.1080/13575270500151870

[51] Giles GM, Wager J, Fong L, et al Twenty-month effectiveness of a non-aversive, long-term, low-cost programme for persons with persisting neurobehavioural disability. Brain Injury 2005;19:753–64. 10.1080/02699050500110108 16175836

[52] Daffern M, Howells K, Hamilton L, et al The impact of structured risk assessments followed by management recommendations on aggression in patients with personality disorder. J Forens Psychiatry Psychol 2009;20:661–79. 10.1080/14789940903173990

[53] C-Y Yip V, Gudjonsson GH, Perkins D, et al A non-randomised controlled trial of the R&R2MHP cognitive skills program in high risk male offenders with severe mental illness. BMC Psychiatry 2013;13:267 10.1186/1471-244X-13-267 24498962 PMC3853927

[54] Lanza M, Ridenour M, Hendricks S, et al The violence prevention community meeting: a multi-site study. Arch Psychiatr Nurs 2016;30:382–6. 10.1016/j.apnu.2016.01.003 27256945 PMC4894320

[55] Lanza ML, Rierdan J, Forester L, et al Reducing violence against nurses: the violence prevention community meeting. Issues Ment Health Nurs 2009;30:745–50. 10.3109/01612840903177472 19916808

[56] Calabro K, MacKey TA, Williams S Evaluation of training designed to prevent and manage patient violence. Issues Ment Health Nurs 2002;23:3–15. 10.1080/01612840252825446 11887608

[57] Björkdahl A, Hansebo G, Palmstierna T The influence of staff training on the violence prevention and management climate in psychiatric inpatient units. J Psychiatr Ment Health Nurs 2013;20:396–404. 10.1111/j.1365-2850.2012.01930.x 22632809

[58] Arguvanli S.Ç et al Effect of aggression management training program on knowledge and attitudes of nurses working at psychiatric clinics/Agresyon yönetimi egitim programinin psikiyatri kliniklerinde çalisan hemsirelerin bilgi ve tutumlarina etkisi. Anadolu Psikiyatri Dergisi 2015;16.

[59] Caspi N, Modai I, Barak P, et al Pindolol augmentation in aggressive schizophrenic patients: a double-blind crossover randomized study. Int Clin Psychopharmacol 2001;16:111–5. 10.1097/00004850-200103000-00006 11236069

[60] Davies BE, Lowe K, Morgan S, et al An evaluation of the effectiveness of positive behavioural support within a medium secure mental health forensic service. J Forens Psychiatry Psychol 2019;30:38–52. 10.1080/14789949.2018.1459785

[61] Isaak V, Vashdi D, Bar-Noy D, et al Enhancing the safety climate and reducing violence against staff in closed hospital wards. Workplace Health Saf 2017;65:409–16. 10.1177/2165079916672478 27941087

[62] Price O, Baker J, Bee P, et al Patient perspectives on barriers and enablers to the use and effectiveness of de-escalation techniques for the management of violence and aggression in mental health settings. J Adv Nurs 2018;74:614–25. 10.1111/jan.13488 29082552

[63] Hvidhjelm J, Sestoft D, Skovgaard LT, et al Aggression in psychiatric wards: effect of the use of a structured risk assessment. Issues Ment Health Nurs 2016;37:960–7. 10.1080/01612840.2016.1241842 27901619

[64] Olsson H, Schön U-K Reducing violence in forensic care - how does it resemble the domains of a recovery-oriented care? J Ment Health 2016;25:506–11. 10.3109/09638237.2016.1139075 26854519

[65] Mistler LA, Ben-Zeev D, Carpenter-Song E, et al Mobile mindfulness intervention on an acute psychiatric unit: feasibility and acceptability study. JMIR Mental Health 2017;4 10.2196/mental.7717 PMC5583505 28827214

[66] Needham I, Abderhalden C, Halfens RJG, et al The effect of a training course in aggression management on mental health nurses’ perceptions of aggression: a cluster randomised controlled trial. Int J Nurs Stud 2005;42:649–55. 10.1016/j.ijnurstu.2004.10.003 15982464

[67] Sjöström N, Eder DN, Malm U, et al Violence and its prediction at a psychiatric hospital. Eur Psychiatry 2001;16:459–65. 10.1016/S0924-9338(01)00607-1 11777736

[68] Sival RC, Albronda T, Haffmans PMJ, et al Is aggressive behaviour influenced by the use of a behaviour rating scale in patients in a psychogeriatric nursing home? Int J Geriatr Psychiatry 2000;15:108–11. 10.1002/(SICI)1099-1166(200002)15:2<108::AID-GPS80>3.0.CO;2-H 10679841

[69] Skovdahl K, Kihlgren AL, Kihlgren M Dementia and aggressiveness: video recorded morning care from different care units. J Clin Nurs 2003;12:888–98. 10.1046/j.1365-2702.2003.00809.x 14632982

[70] Stone T, McMillan M, Hazelton M, et al Wounding words: swearing and verbal aggression in an inpatient setting. Perspect Psychiatr Care 2011;47:194–203. 10.1111/j.1744-6163.2010.00295.x 21950366

[71] Sutton D, Wilson M, Van Kessel K, et al Optimizing arousal to manage aggression: a pilot study of sensory modulation. Int J Ment Health Nurs 2013;22:500–11. 10.1111/inm.12010 23374543

[72] Lipscomb JA, London M, Chen YM, et al Safety climate and workplace violence prevention in state-run residential addiction treatment centers. Work 2012;42:47–56. 10.3233/WOR-2012-1330 22635149

[73] Wright S, Sayer J, Parr A-M, et al Breakaway and physical restraint techniques in acute psychiatric nursing: results from a national survey of training and practice. J Forens Psychiatry Psychol 2005;16:380–98. 10.1080/14789940412331270735

[74] Berg J, Kaltiala-Heino R, Välimäki M Management of aggressive behaviour among adolescents in forensic units: a four-country perspective. J Psychiatr Ment Health Nurs 2011;18:776–85. 10.1111/j.1365-2850.2011.01726.x 21985680

[75] Biancosino B, Delmonte S, Grassi L, et al Violent behavior in acute psychiatric inpatient facilities: a national survey in Italy. J Nerv Ment Dis 2009;197:772–82. 10.1097/NMD.0b013e3181bb0d6b 19829207

[76] Caspi E Aggressive behaviors between residents with dementia in an assisted living residence. Dementia 2015;14:528–46. 10.1177/1471301213502588 24339115

[77] Chaplin R, McGeorge M, Hinchcliffe G, et al Aggression on psychiatric inpatient units for older adults and adults of working age. Int J Geriatr Psychiatry 2008;23:874–6. 10.1002/gps.1975 18229875

[78] Delaney J, Cleary M, Jordan R, et al An exploratory investigation into the nursing management of aggression in acute psychiatric settings. J Psychiatr Ment Health Nurs 2001;8:77–84. 10.1111/j.1365-2850.2001.00350.x 11879497

[79] Meaden A, Hacker D, Spencer K Acute aggression risk: an early warning signs methodology. The Journal of Forensic Practice 2013;15:21–31. 10.1108/14636641311299059

[80] Umut G, Altun Zeren Öztürk, Danışmant BS, et al Relationship between treatment adherence, insight and violence among schizophrenia inpatients in a training Hospital sample. Düşünen Adam 2012;25:212–20.

[81] Bahareethan M, Shah A Aggressive behaviour, staff attitude and staff perception of patients on two continuing care psychogeriatric wards. Aging Ment Health 2000;4:66–71. 10.1080/13607860055991

[82] Berg J, Kaltiala-Heino R, Löyttyniemi V, et al Staff's perception of adolescent aggressive behaviour in four European forensic units: a qualitative interview study. Nord J Psychiatry 2013;67:124–31. 10.3109/08039488.2012.697190 22774936

[83] Stevenson KN, Jack SM, O’Mara L, et al Registered nurses’ experiences of patient violence on acute care psychiatric inpatient units: an interpretive descriptive study. BMC Nurs 2015;14:35 10.1186/s12912-015-0079-5 25999795 PMC4440495

[84] Spokes K, Bond K, Lowe T, et al HOVIS - The Hertfordshire/Oxfordshire Violent Incident Study. J Psychiatr Ment Health Nurs 2002;9:199–209. 10.1046/j.1365-2850.2002.00467.x 11966990

[85] Zuzelo PR, Curran SS, Zeserman MA Registered nurses’ and behavior health associates’ responses to violent inpatient interactions on behavioral health units. J Am Psychiatr Nurses Assoc 2012;18:112–26. 10.1177/1078390312438553 22412084

[86] Camuccio CA, CHAMBERS M, VÄLIMÄKI M, et al Managing distressed and disturbed patients: the thoughts and feelings experienced by Italian nurses. J Psychiatr Ment Health Nurs 2012;19:807–15. 10.1111/j.1365-2850.2011.01857.x 22296342

[87] McCann TV, Baird J, Muir-Cochrane E Factors influencing clinicians' attitudes about aggression in Australian acute old age psychiatry inpatient units: a cross sectional survey design. Issues Ment Health Nurs 2014;35:542–50. 10.3109/01612840.2014.883559 24963855

[88] Tema TR, Poggenpoel M, Myburgh CPH Experiences of psychiatric nurses exposed to hostility from patients in a forensic ward. J Nurs Manag 2011;19:915–24. 10.1111/j.1365-2834.2011.01304.x 21988439

[89] Nijman H, Bowers L, Oud N, et al Psychiatric nurses' experiences with inpatient aggression. Aggress Behav 2005;31:217–27. 10.1002/ab.20038

[90] Chen Wen‐Ching, Wang Jung‐Der, Lew‐Ting Chih‐Yin, et al Workplace violence on workers caring for Long‐term institutionalized schizophrenic patients in Taiwan. J Occup Health 2007;49:311–6. 10.1539/joh.49.311 17690525

[91] Cutcliffe JR Qualified nurses' lived experience of violence perpetrated by individuals suffering from enduring mental health problems: a hermeneutic study. Int J Nurs Stud 1999;36:105–16. 10.1016/S0020-7489(99)00013-9 10376220

[92] Evans RE, Petter S Identifying mitigating and challenging beliefs in dealing with threatening patients: an analysis of experiences of clinicians working in a psychiatric intensive care unit. Journal of Psychiatric Intensive Care 2012;8:113–9. 10.1017/S1742646411000318

[93] Lantta T, Daffern M, Kontio R, et al Implementing the dynamic appraisal of situational aggression in mental health units. Clinical Nurse Specialist 2015;29:230–43. 10.1097/NUR.0000000000000140 26053606

[94] Tomágová M, Bóriková I, Lepiešová M, et al NURSES’ EXPERIENCE AND ATTITUDES TOWARDS INPATIENT AGGRESSION ON PSYCHIATRIC WARDS. CEJNM 2016;7:462–9. 10.15452/CEJNM.2016.07.0016

[95] Hylén U, Engström I, Engström K, et al Providing good care in the shadow of violence – an interview study with nursing staff and ward managers in psychiatric inpatient care in Sweden. Issues Ment Health Nurs 2019;40:148–57. 10.1080/01612840.2018.1496207 30376382

[96] Lantta T, Anttila M, Kontio R, et al Violent events, ward climate and ideas for violence prevention among nurses in psychiatric wards: a focus group study. Int J Ment Health Syst 2016;10 10.1186/s13033-016-0059-5 PMC4820948 27051463

[97] Meehan T, McINTOSH W, Bergen H Aggressive behaviour in the high-secure forensic setting: the perceptions of patients. J Psychiatr Ment Health Nurs 2006;13:19–25. 10.1111/j.1365-2850.2006.00906.x 16441389

[98] Olsson H, Audulv Åsa, Strand S, et al Reducing or increasing violence in forensic care: a qualitative study of inpatient experiences. Arch Psychiatr Nurs 2015;29:393–400. 10.1016/j.apnu.2015.06.009 26577553

[99] Van Wijk E, Traut A, Julie H Environmental and nursing-staff factors contributing to aggressive and violent behaviour of patients in mental health facilities. Curationis 2014;37:01–8. 10.4102/curationis.v37i1.1122 25686162

[100] Daffern M Assessing the functions of aggression in psychiatric inpatients. Behav Anal Today 2007;8:43–51. 10.1037/h0100107

[101] Duxbury J, Whittington R Causes and management of patient aggression and violence: staff and patient perspectives. J Adv Nurs 2005;50:469–78. 10.1111/j.1365-2648.2005.03426.x 15882363

[102] Janicki N Prosecuting inpatient violence: perceptions of staff, patients and others in a women's enhanced medium secure service. The British Journal of Forensic Practice 2009;11:27–38. 10.1108/14636646200900026

[103] Lawn S, Pols R Nicotine withdrawal: pathway to aggression and assault in the locked psychiatric ward? Australasian Psychiatry 2003;11:199–203. 10.1046/j.1039-8562.2003.00548.x

[104] Nolan KA, Shope CB, Citrome L, et al Staff and patient views of the reasons for aggressive incidents: a prospective, incident-based study. Psychiatr Q 2009;80:167–72. 10.1007/s11126-009-9104-8 19412665

[105] Dickens G, Piccirillo M, Alderman N Causes and management of aggression and violence in a forensic mental health service: perspectives of nurses and patients. Int J Ment Health Nurs 2013;22:532–44. 10.1111/j.1447-0349.2012.00888.x 23167989

[106] Wright KM, Duxbury JA, Baker A, et al A qualitative study into the attitudes of patients and staff towards violence and aggression in a high security Hospital. J Psychiatr Ment Health Nurs 2014;21:184–8. 10.1111/jpm.12108 23980566

[107] Lamanna D, Ninkovic D, Vijayaratnam V, et al Aggression in psychiatric hospitalizations: a qualitative study of patient and provider perspectives. J Ment Health 2016;25:536–42. 10.1080/09638237.2016.1207222 27809615

[108] Paschali M, Kamp D, Reichmann C, et al A systematic evaluation of impulsive-aggressive behavior in psychogeriatric inpatients using the staff observation aggression scale-revision (SOAS-R). Int Psychogeriatr 2018;30:61–8. 10.1017/S1041610217001600 28851471

[109] Almvik R, Rasmussen K, Woods P Challenging behaviour in the elderly—monitoring violent incidents. Int J Geriatr Psychiatry 2006;21:368–74. 10.1002/gps.1474 16534771

[110] Reininghaus U, Craig T, Gournay K, et al The High Secure Psychiatric Hospitals’ Nursing Staff Stress Survey 3: Identifying stress resistance resources in the stress process of physical assault. Pers Individ Dif 2007;42:397–408. 10.1016/j.paid.2006.07.013

[111] Trenoweth S Perceiving risk in dangerous situations: risks of violence among mental health inpatients. J Adv Nurs 2003;42:278–87. 10.1046/j.1365-2648.2003.02617.x 12680972

[112] DE Niet GJ, Hutschemaekers GJM, Lendemeijer BHHG Is the reducing effect of the staff observation aggression scale owing to a learning effect? an explorative study. J Psychiatr Ment Health Nurs 2005;12:687–94. 10.1111/j.1365-2850.2005.00895.x 16336593

[113] de Looff P, Nijman H, Didden R, et al Burnout symptoms in forensic psychiatric nurses and their associations with personality, emotional intelligence and client aggression: a cross-sectional study. J Psychiatr Ment Health Nurs 2018;25:506–16. 10.1111/jpm.12496 30199590

[114] Bharwani G, Parikh PJ, Lawhorne LW, et al Individualized behavior management program for Alzheimer’s/dementia residents using behavior-based ergonomic therapies. Am J Alzheimers Dis Other Demen 2012;27:188–95. 10.1177/1533317512443869 22517891 PMC10845423

[115] Carlson GA, Potegal M, Margulies D, et al Liquid risperidone in the treatment of rages in psychiatrically hospitalized children with possible bipolar disorder. Bipolar Disord 2010;12:205–12. 10.1111/j.1399-5618.2010.00793.x 20402713 PMC2990969

[116] Chan S, Fung MY, Tong CW, et al The clinical effectiveness of a multisensory therapy on clients with developmental disability. Res Dev Disabil 2005;26:131–42. 10.1016/j.ridd.2004.02.002 15590244

[117] DeYoung S, Just G, Harrison R Decreasing aggressive, agitated, or disruptive behavior: participation in a behavior management unit. J Gerontol Nurs 2002;28:22–31. 10.3928/0098-9134-20020601-08 12071270

[118] Higueras A et al Effects of a humor-centered activity on disruptive behavior in patients in a general Hospital psychiatric ward. International Journal of Clinical and Health Psychology 2006;6:53–64.

[119] Speziale J, Black E, Coatsworth-Puspoky R, et al Moving forward: Evaluating a curriculum for managing responsive behaviors in a geriatric psychiatry inpatient population. Gerontologist 2009;49): :570–6. 10.1093/geront/gnp069 10.1093/geront/gnp069 19520841

[120] Zwijsen SA, Smalbrugge M, Eefsting JA, et al Coming to grips with challenging behavior: a cluster randomized controlled trial on the effects of a multidisciplinary care program for challenging behavior in dementia. J Am Med Dir Assoc 2014;15:531.e1–531.e10. : p. e1-531 10.1016/j.jamda.2014.04.007 24878214

[121] Bennett R, Ramakrishna V, Maganty D Management of disturbed behaviour in a psychiatric intensive care unit: views of staff on options for intervention. Journal of Psychiatric Intensive Care 2011;7:85–9. 10.1017/S1742646410000257

[122] Foley KL, Sudha S, Sloane PD, et al Staff perceptions of successful management of severe behavioral problems in dementia special care units. Dementia 2003;2:105–24. 10.1177/1471301203002001998

[123] Hallett N, Dickens GL De-escalation: a survey of clinical staff in a secure mental health inpatient service. Int J Ment Health Nurs 2015;24:324–33. 10.1111/inm.12136 25975221

[124] Bowers L Association between staff factors and levels of conflict and containment on acute psychiatric wards in England. Psychiatric Services 2009;60:231–9. 10.1176/ps.2009.60.2.231 19176418

[125] Lowe T, Wellman N, Taylor R Limit-setting and decision-making in the management of aggression. J Adv Nurs 2003;41:154–61. 10.1046/j.1365-2648.2003.02517.x 12519274

[126] Ireland JL, Sebalo I, McNeill K, et al Impacting on factors promoting intra-group aggression in secure psychiatric settings. Heliyon 2019;5 10.1016/j.heliyon.2019.e01400 PMC6439227 30976684

[127] Quirk A, Lelliott P, Seale C Risk management by patients on psychiatric wards in London: an ethnographic study. Health Risk Soc 2005;7:85–91. 10.1080/13698570500034683

[128] Ireland CA, Halpin L, Sullivan C Critical incidents in a forensic psychiatric population: an exploratory study of motivational factors. J Forens Psychiatry Psychol 2014;25:714–32. 10.1080/14789949.2014.955809

[129] Jeffs L, Rose D, Macrae C, et al What near misses tell us about risk and safety in mental health care. J Psychiatr Ment Health Nurs 2012;19:430–7. 10.1111/j.1365-2850.2011.01812.x 22070194

[130] Koukia E, Mangoulia P, Stathopoulos T, et al Greek mental health nurses’ practices and attitudes in the management of acute cases. Issues Ment Health Nurs 2013;34:192–7. 10.3109/01612840.2012.733908 23477440

[131] Terkelsen TB, Larsen IB Fear, danger and aggression in a Norwegian locked psychiatric ward: dialogue and ethics of care as contributions to combating difficult situations. Nursing Ethics 2016;23:308–17. 25552587 10.1177/0969733014564104

[132] Cole M, Baldwin D, Thomas P Sexual assault on wards: staff actions and reactions. Int J Psychiatry Clin Pract 2003;7:239–42. 10.1080/13651500310002355 24930410

[133] Phillips L Reflections on the education and training of mental health staff who work with women who have been sexually abused in childhood. J Psychiatr Ment Health Nurs 2011;18:696–705. 10.1111/j.1365-2850.2010.01680.x 21896112

[134] Gallop R, Engels S, DiNunzio R, et al Abused women's concerns about safety and the therapeutic environment during psychiatric hospitalization. Can J Nurs Res 1999;31:53–70. 10696160

[135] Yang M-H, Wu S-C, Lin J-G, et al The efficacy of acupressure for decreasing agitated behaviour in dementia: a pilot study. J Clin Nurs 2007;16:308–15. 10.1111/j.1365-2702.2006.01428.x 17239066

[136] Cormac I, Russell I, Ferriter M Review of seclusion policies in high secure hospitals and medium secure units in England, Scotland and Wales. J Psychiatr Ment Health Nurs 2005;12:380–2. 10.1111/j.1365-2850.2005.00833.x 15876247

[137] Georgieva I, Mulder CL, Noorthoorn E Reducing seclusion through involuntary medication: a randomized clinical trial. Psychiatry Res 2013;205:48–53. 10.1016/j.psychres.2012.08.002 22951334

[138] Kirkevold Øyvind, Engedal K Prevalence of patients subjected to constraint in Norwegian nursing homes. Scand J Caring Sci 2004;18:281–6. 10.1111/j.1471-6712.2004.00278.x 15355522

[139] Raboch J, Kalisová L, Nawka A, et al Use of coercive measures during involuntary hospitalization: findings from ten European countries. Psychiatr Serv 2010;61:1012–7. 10.1176/ps.2010.61.10.1012 20889640

[140] Steinert T, Martin V, Baur M, et al Diagnosis-Related frequency of compulsory measures in 10 German psychiatric hospitals and correlates with Hospital characteristics. Soc Psychiatry Psychiatr Epidemiol 2007;42:140–5. 10.1007/s00127-006-0137-0 17180296

[141] Gowda GS, Lepping P, Noorthoorn EO, et al Restraint prevalence and perceived coercion among psychiatric inpatients from South India: a prospective study. Asian J Psychiatr 2018;36:10–16. 10.1016/j.ajp.2018.05.024 29857265

[142] Gowda GS, Lepping P, Ray S, et al Clinician attitude and perspective on the use of coercive measures in clinical practice from tertiary care mental health establishment - A cross-sectional study. Indian J Psychiatry 2019;61:151–5. 10.4103/psychiatry.IndianJPsychiatry_336_18 PMC6425791 30992609

[143] Hotzy F, Jaeger M, Buehler E, et al Attitudinal variance among patients, next of kin and health care professionals towards the use of containment measures in three psychiatric hospitals in Switzerland. BMC Psychiatry 2019;19:128 10.1186/s12888-019-2092-9 31035954 PMC6489208

[144] Vedana KGG, da Silva DM, Ventura CAA, et al Physical and mechanical restraint in psychiatric units: perceptions and experiences of nursing staff. Arch Psychiatr Nurs 2018;32:367–72. 10.1016/j.apnu.2017.11.027 29784216

[145] Krieger E, Moritz S, Weil R, et al Patients’ attitudes towards and acceptance of coercion in psychiatry. Psychiatry Res 2018;260:478–85. 10.1016/j.psychres.2017.12.029 29287276

[146] Reynolds EK, Grados MA, Praglowski N, et al Use of modified positive behavioral interventions and supports in a psychiatric inpatient unit for high-risk youths. Psychiatric Services 2016;67:570–3. 10.1176/appi.ps.201500039 26876659

[147] Brady NS, Spittal MJ, Brophy LM, et al Patients’ Experiences of Restrictive Interventions in Australia: Findings From the 2010 Australian Survey of Psychosis. Psychiatric Services 2017;68:966–9. 10.1176/appi.ps.201600300 28457209

[148] Bigwood S, Crowe M ‘It’s part of the job, but it spoils the job': A phenomenological study of physical restraint. Int J Ment Health Nurs 2008;17:215–22. 10.1111/j.1447-0349.2008.00526.x 18460083

[149] Lee S, GRAY R, GOURNAY K, et al Views of nursing staff on the use of physical restraint. J Psychiatr Ment Health Nurs 2003;10:425–30. 10.1046/j.1365-2850.2003.00625.x 12887634

[150] Perkins E, Prosser H, Riley D, et al Physical restraint in a therapeutic setting; a necessary evil? Int J Law Psychiatry 2012;35:43–9. 10.1016/j.ijlp.2011.11.008 22178072

[151] Sequeira H, Halstead S The psychological effects on nursing staff of administering physical restraint in a secure psychiatric hospital: ‘When I go home, it's then that I think about it’. The British Journal of Forensic Practice 2004;6:3–15. 10.1108/14636646200400002

[152] Exworthy T, Mohan D, Hindley N, et al Seclusion: punitive or protective? The Journal of Forensic Psychiatry 2001;12:423–33. 10.1080/09585180121877

[153] Kuosmanen L, Makkonen P, Lehtila H, et al Seclusion experienced by mental health professionals. J Psychiatr Ment Health Nurs 2015;22:333–6. 10.1111/jpm.12224 26014830

[154] Muir‐Cochrane EC, Baird J, McCann T Nurses' experiences of restraint and seclusion use in short-stay acute old age psychiatry inpatient units: a qualitative study. J Psychiatr Ment Health Nurs 2015;22:109–15. 10.1111/jpm.12189 25524501

[155] Duxbury J, Thomson G, Scholes A, et al Staff experiences and understandings of the restrain yourself initiative to minimize the use of physical restraint on mental health wards. Int J Ment Health Nurs 2019;28:845–56. 10.1111/inm.12577 30887624

[156] Nielsen LD, Gildberg FA, Bech P, et al Forensic mental health clinician's experiences with and assessment of alliance regarding the patient's readiness to be released from mechanical restraint. Int J Ment Health Nurs 2018;27:116–25. 10.1111/inm.12300 27982496

[157] Kontio R, Välimäki M, Putkonen H, et al Patient restrictions: are there ethical alternatives to seclusion and restraint? Nurs Ethics 2010;17:65–76. 10.1177/0969733009350140 20089626

[158] Chien W-T, Chan CWH, Lam L-W, et al Psychiatric inpatients’ perceptions of positive and negative aspects of physical restraint. Patient Educ Couns 2005;59:80–6. 10.1016/j.pec.2004.10.003 16198221

[159] Knowles SF, Hearne J, Smith I Physical restraint and the therapeutic relationship. J Forens Psychiatry Psychol 2015;26:461–75. 10.1080/14789949.2015.1034752

[160] Ezeobele IE, Malecha AT, Mock A, et al Patients’ lived seclusion experience in acute psychiatric hospital in the United States: a qualitative study. J Psychiatr Ment Health Nurs 2014;21:303–12. 10.1111/jpm.12097 23834325

[161] Faschingbauer KM, Peden-McAlpine C, Tempel W Use of Seclusion: finding the voice of the patient to influence practice. J Psychosoc Nurs Ment Health Serv 2013;51:32–8. 10.3928/02793695-20130503-01 23668381

[162] Holmes D, Kennedy SL, Perron A The mentally ill and social exclusion: a critical examination of the use of seclusion from the patient's perspective. Issues Ment Health Nurs 2004;25:559–78. 10.1080/01612840490472101 15371143

[163] Bergk J, Einsiedler B, Flammer E, et al A randomized controlled comparison of seclusion and mechanical restraint in inpatient settings. Psychiatr Serv 2011;62:1310–7. 10.1176/ps.62.11.pss6211_1310 22211210

[164] Kontio R, Joffe G, Putkonen H, et al Seclusion and restraint in psychiatry: patients' experiences and practical suggestions on how to improve practices and use alternatives. Perspect Psychiatr Care 2012;48:16–24. 10.1111/j.1744-6163.2010.00301.x 22188043

[165] Larue C, Dumais A, Boyer R, et al The experience of seclusion and restraint in psychiatric settings: perspectives of patients. Issues Ment Health Nurs 2013;34:317–24. 10.3109/01612840.2012.753558 23663018

[166] Bonner G, Lowe T, Rawcliffe D, et al Trauma for all: a pilot study of the subjective experience of physical restraint for mental health inpatients and staff in the UK. J Psychiatr Ment Health Nurs 2002;9:465–73. 10.1046/j.1365-2850.2002.00504.x 12164909

[167] Fish R, Hatton C Gendered experiences of physical restraint on locked wards for women. Disabil Soc 2017;32:790–809. 10.1080/09687599.2017.1329711

[168] Wilson C, Rouse L, Rae S, et al Mental health inpatients’ and staff members’ suggestions for reducing physical restraint: A qualitative study. J Psychiatr Ment Health Nurs 2018;25:188–200. 10.1111/jpm.12453 29323442

[169] Fish R ‘Behind This Wall’ – Experiences of Seclusion on Locked Wards for Women. Scandinavian Journal of Disability Research 2018;20:139–51. 10.16993/sjdr.59

[170] Gowda GS, Kumar CN, Ray S, et al Caregivers’ attitude and perspective on coercion and restraint practices on psychiatric inpatients from South India. J Neurosci Rural Pract 2019;10:261–6. 10.4103/jnrp.jnrp_302_18 31001015 PMC6454949

[171] Larue C, Goulet M-H, Prevost M-J, et al Identification and analysis of factors contributing to the reduction in Seclusion and restraint for a population with intellectual disability. Journal of Applied Research in Intellectual Disabilities 2018;31:e212–22. 10.1111/jar.12309 27910254

[172] Wilson C, Rouse L, Rae S, et al Is restraint a ‘necessary evil’ in mental health care? Mental health inpatients’ and staff members’ experience of physical restraint. Int J Ment Health Nurs 2017;26:500–12. 10.1111/inm.12382 28960742

[173] Holmes D, Murray SJ, Knack N Experiencing Seclusion in a forensic psychiatric setting: a phenomenological study. J Forensic Nurs 2015;11:200–13. 10.1097/JFN.0000000000000088 26457901

[174] Bonner G, Wellman N Postincident review of aggression and violence in mental health settings. J Psychosoc Nurs Ment Health Serv 2010;48:35–40. 10.3928/02793695-20100504-05 20608583

[175] Bowers L, Van Der Merwe M, Paterson B, et al Manual restraint and shows of force: the City-128 study. Int J Ment Health Nurs 2012;21:30–40. 10.1111/j.1447-0349.2011.00756.x 21733054

[176] Tompsett CJ, Domoff S, Boxer P Prediction of restraints among youth in a psychiatric Hospital: application of translational action research. J Clin Psychol 2011;67:368–82. 10.1002/jclp.20772 21254060 PMC3217493

[177] Boumans CE, Egger JIM, Souren PM, et al Nurses' decision on seclusion: patient characteristics, contextual factors and reflexivity in teams. J Psychiatr Ment Health Nurs 2012;19:264–70. 10.1111/j.1365-2850.2011.01777.x 22074324

[178] Mason T, Whitehead E Some specific problems of secluding female patients. Med Sci Law 2001;41:315–24. 10.1177/002580240104100408 11693227

[179] Ryan R, Happell B Learning from experience: using action research to discover consumer needs in post-seclusion Debriefing. Int J Ment Health Nurs 2009;18:100–7. 10.1111/j.1447-0349.2008.00579.x 19290973

[180] Whitecross F, Seeary A, Lee S Measuring the impacts of seclusion on psychiatry inpatients and the effectiveness of a pilot single-session post-seclusion counselling intervention. Int J Ment Health Nurs 2013;22:512–21. 10.1111/inm.12023 23682907

[181] Bleijlevens MHC, Gulpers MJM, Capezuti E, et al Process evaluation of a multicomponent intervention program (EXBELT) to reduce belt restraints in nursing homes. J Am Med Dir Assoc 2013;14:599–604. 10.1016/j.jamda.2013.03.002 23608527

[182] Pellfolk TJ-E, Gustafson Y, Bucht G, et al Effects of a restraint minimization program on staff knowledge, attitudes, and practice: a cluster randomized trial. J Am Geriatr Soc 2010;58:62–9. 10.1111/j.1532-5415.2009.02629.x 20122041

[183] Schreiner GM, Crafton CG, Sevin JA Decreasing the use of mechanical restraints and locked seclusion. Adm Policy Ment Health 2004;31:449–63. 10.1023/B:APIH.0000036413.87440.83 15478875

[184] Ching H, Daffern M, Martin T, et al Reducing the use of seclusion in a forensic psychiatric Hospital: assessing the impact on aggression, therapeutic climate and staff confidence. J Forens Psychiatry Psychol 2010;21:737–60. 10.1080/14789941003681361

[185] Long CG, West R, Afford M, et al Reducing the use of seclusion in a secure service for women. Journal of Psychiatric Intensive Care 2015;11:84–94. 10.1017/S174264641400017X

[186] Smith S, Jones J Use of a sensory room on an intensive care unit. J Psychosoc Nurs Ment Health Serv 2014;52:22–30. 10.3928/02793695-20131126-06 24305908

[187] Espinosa L, Harris B, Frank J, et al Milieu improvement in psychiatry using evidence-based practices: the long and winding road of culture change. Arch Psychiatr Nurs 2015;29:202–7. 10.1016/j.apnu.2014.08.004 26165973

[188] Kontio R, LAHTI M, PITKÄNEN A, et al Impact of eLearning course on nurses' professional competence in seclusion and restraint practices: a randomized controlled study (ISRCTN32869544). J Psychiatr Ment Health Nurs 2011;18:813–21. 10.1111/j.1365-2850.2011.01729.x 21985684

[189] Goulet M-H, Larue C, Lemieux AJ A pilot study of “post-seclusion and/or restraint review” intervention with patients and staff in a mental health setting. Perspect Psychiatr Care 2018;54:212–20. 10.1111/ppc.12225 28635150

[190] Blair EW, Woolley S, Szarek BL, et al Reduction of Seclusion and restraint in an inpatient psychiatric setting: a pilot study. Psychiatr Q 2017;88:1–7. 10.1007/s11126-016-9428-0 26897657

[191] Newman J, Paun O, Fogg L Effects of a staff training intervention on Seclusion rates on an adult inpatient psychiatric unit. J Psychosoc Nurs Ment Health Serv 2018;56:23–30. 10.3928/02793695-20180212-02 29447413

[192] Elzubeir K, Dye S Can amount and duration of seclusion be reduced in psychiatric intensive care units by agreeing smart goals with patients? Journal of Psychiatric Intensive Care 2017;13:109–16. 10.20299/jpi.2017.010

[193] Huizing AR, Hamers JPH, Gulpers MJM, et al Short-Term effects of an educational intervention on physical restraint use: a cluster randomized trial. BMC Geriatr 2006;6:17 10.1186/1471-2318-6-17 17067376 PMC1635553

[194] Abdel-Hussein NH, Mohamed SH Effectiveaness of an Educational Program on Nurses’ Knowledge toward Restraint and Seclusion for inpatients at Psychiatric Teaching Hospitals. Indian J Public Health Res Dev 2018;9:1175–80. 10.5958/0976-5506.2018.02009.0

[195] Bak J, Zoffmann V, Sestoft DM, et al Mechanical restraint in psychiatry: preventive factors in theory and practice. A Danish-Norwegian association study. Perspect Psychiatr Care 2014;50:155–66. 10.1111/ppc.12036 25040212

[196] Bak J, Zoffmann V, Sestoft DM, et al Comparing the effect of non-medical mechanical restraint preventive factors between psychiatric units in Denmark and Norway. Nord J Psychiatry 2015;69:1715–25. 10.3109/08039488.2014.996600 25614990

[197] Keski-Valkama A, Sailas E, Eronen M, et al A 15-year national follow-up: legislation is not enough to reduce the use of seclusion and restraint. Soc Psychiatry Psychiatr Epidemiol 2007;42:747–52. 10.1007/s00127-007-0219-7 17598058

[198] Goulet M-H, Larue C, Dumais A Evaluation of seclusion and restraint reduction programs in mental health: a systematic review. Aggress Violent Behav 2017;34:139–46. 10.1016/j.avb.2017.01.019

[199] Lee SW, Sayer J, Parr A-M, et al Soo, physical restraint training for nurses in English and Welsh psychiatric intensive care and regional secure units. J Ment Health 2001;10:151–62.

[200] Kontio R, Välimäki M, Putkonen H, et al Nurses' and physicians' educational needs in seclusion and restraint practices. Perspect Psychiatr Care 2009;45:198–207. 10.1111/j.1744-6163.2009.00222.x 19566692

[201] Hatta K, Shibata N, Ota T, et al Association between physical restraint and drug-induced liver injury. Neuropsychobiology 2007;56:180–4. 10.1159/000119736 18332646

[202] Bowers L, Alexander J, Simpson A, et al Student psychiatric nurses’ approval of containment measures: Relationship to perception of aggression and attitudes to personality disorder. Int J Nurs Stud 2007;44:349–56. 10.1016/j.ijnurstu.2005.03.002 17336606

[203] Braham LG, Heasley JF, Akiens S An evaluation of night confinement in a high secure Hospital. Mental Healt Rev J 2013;18:21–31. 10.1108/13619321311306947

[204] Chu S, McNeill K, Wright KM, et al The impact of a night confinement policy on patients in a UK high secure inpatient mental health service. The Jnl of Forensic Practice 2015;17:21–30. 10.1108/JFP-11-2014-0045

[205] Hottinen A, VÄLIMÄKI M, SAILAS E, et al Attitudes towards different containment measures: a questionnaire survey in Finnish adolescent psychiatry. J Psychiatr Ment Health Nurs 2012;19:521–7. 10.1111/j.1365-2850.2011.01820.x 22093236

[206] Wharewera-Mika JP, Cooper EP, Wiki NRN, et al Strategies to reduce the use of seclusion with tāngata whai I te ora (Māori mental health service users). Int J Ment Health Nurs 2016;25:258–65. 10.1111/inm.12219 27219838

[207] Haugom EW, Granerud A Shielding in mental health hospitals: description and assessment by staff. SAGE Open 2016;6.

[208] Papadopoulos C, Bowers L, Quirk A, et al Events preceding changes in conflict and containment rates on acute psychiatric wards. Psychiatr Serv 2012;63:40–7. 10.1176/appi.ps.201000480 22227758

[209] Ejneborn Looi G-M, Engström Åsa, Sävenstedt S A self-destructive care: self-reports of people who experienced coercive measures and their suggestions for alternatives. Issues Ment Health Nurs 2015;36:96–103. 10.3109/01612840.2014.951134 25625709

[210] Barr L, Wynaden D, Heslop K Promoting positive and safe care in forensic mental health inpatient settings: evaluating critical factors that assist nurses to reduce the use of restrictive practices. Int J Ment Health Nurs 2019;28:888 10.1111/inm.12588 30916443

[211] Bak J, Aggernæs H Coercion within Danish psychiatry compared with 10 other European countries. Nord J Psychiatry 2012;66:297–302. 10.3109/08039488.2011.632645 22087631

[212] Jaeger M, Ketteler D, Rabenschlag F, et al Informal coercion in acute inpatient setting—Knowledge and attitudes held by mental health professionals. Psychiatry Res 2014;220:1007–11. 10.1016/j.psychres.2014.08.014 25249438

[213] Seo MK, Kim SH, Rhee M Coercion in psychiatric care: can paternalism justify coercion? Int J Soc Psychiatry 2013;59:217–23. 10.1177/0020764011431543 22222850

[214] Lovell A, Smith D, Johnson P A qualitative investigation into nurses' perceptions of factors influencing staff injuries sustained during physical interventions employed in response to service user violence within one secure learning disability service. J Clin Nurs 2015;24:1926–35. 10.1111/jocn.12830 25926294

[215] Tateno M, Sugiura K, Uehara K, et al Attitude of young psychiatrists toward coercive measures in psychiatry: a case vignette study in Japan. Int J Ment Health Syst 2009;3:20 10.1186/1752-4458-3-20 19772614 PMC2754431

[216] Elmer T, Rabenschlag F, Schori D, et al Informal coercion as a neglected form of communication in psychiatric settings in Germany and Switzerland. Psychiatry Res 2018;262:400–6. 10.1016/j.psychres.2017.09.014 28958458

[217] Gustafsson N, Salzmann-Erikson M Effect of complex working conditions on nurses who exert coercive measures in forensic psychiatric care. J Psychosoc Nurs Ment Health Serv 2016;54:37–43. 10.3928/02793695-20160817-06 27576227

[218] Jalil R, Huber JW, Sixsmith J, et al Mental health nurses' emotions, exposure to patient aggression, attitudes to and use of coercive measures: cross sectional questionnaire survey. Int J Nurs Stud 2017;75:130–8. 10.1016/j.ijnurstu.2017.07.018 28797822

[219] Martello M, Doronina O, Perillo A, et al Nurses’ Perceptions of Engaging With Patients to Reduce Restrictive Practices in an Inpatient Psychiatric Unit. Health Care Manag 2018;37:342–53. 10.1097/HCM.0000000000000235 30216195

[220] McKeown M, Thomson G, Scholes A, et al "Catching your tail and firefighting": The impact of staffing levels on restraint minimization efforts. J Psychiatr Ment Health Nurs 2019;26:131–41. 10.1111/jpm.12532 31111648

[221] Molewijk B, Kok A, Husum T, et al Staff’s normative attitudes towards coercion: the role of moral doubt and professional context—a cross-sectional survey study. BMC Med Ethics 2017;18 10.1186/s12910-017-0190-0 PMC5445484 28545519

[222] Raveesh BN, Pathare S, Noorthoorn EO, et al Staff and caregiver attitude to coercion in India. Indian J Psychiatry 2016;58:221–9. 10.4103/0019-5545.196847 PMC5282619 28216773

[223] Georgieva I, Mulder CL, Wierdsma A Patients' preference and experiences of forced medication and seclusion. Psychiatr Q 2012;83:1–13. 10.1007/s11126-011-9178-y 21516449 PMC3289788

[224] Haw C, Stubbs J, Bickle A, et al Coercive treatments in forensic psychiatry: a study of patients' experiences and preferences. J Forens Psychiatry Psychol 2011;22:564–85. 10.1080/14789949.2011.602097

[225] Sequeira H, Halstead S “Is it meant to hurt, is it?” Management of violence in women with developmental disabilities. Violence against women 2001;7:462–76.

[226] Sustere E, Tarpey E Least restrictive practice: its role in patient independence and recovery. J Forens Psychiatry Psychol 2019;30:614–29. 10.1080/14789949.2019.1566489

[227] Larsen IB, Terkelsen TB Coercion in a locked psychiatric ward: perspectives of patients and staff. Nurs Ethics 2014;21:426–36. 10.1177/0969733013503601 24106262

[228] Whittington R, Bowers L, Nolan P, et al Approval ratings of inpatient coercive interventions in a national sample of mental health service users and staff in England. Psychiatr Serv 2009;60:792–8. 10.1176/ps.2009.60.6.792 19487349

[229] Reisch T, Beeri S, Klein G, et al Comparing attitudes to containment measures of patients, health care professionals and next of kin. Front Psychiatry 2018;9 10.3389/fpsyt.2018.00529 PMC6212593 30416459

[230] Rippon D, Reid K, Kay G Views on restrictive practices on young people in psychiatric wards. Nursing Times 2018;114:24–8.

[231] Ryan CJ, Bowers L Coercive manoeuvres in a psychiatric intensive care unit. J Psychiatr Ment Health Nurs 2005;12:695–702. 10.1111/j.1365-2850.2005.00899.x 16336594

[232] Johnston MS, Kilty JM “It’s for their own good”: Techniques of neutralization and security guard violence against psychiatric patients. Punishment & Society 2016;18:177–97. 10.1177/1462474516635884

[233] Mackay I, Paterson B, Cassells C Constant or special observations of inpatients presenting a risk of aggression or violence: nurses' perceptions of the rules of engagement. J Psychiatr Ment Health Nurs 2005;12:464–71. 10.1111/j.1365-2850.2005.00867.x 16011502

[234] Delaney KR, Johnson ME Keeping the unit safe: Mapping psychiatric nursing skills. J Am Psychiatr Nurses Assoc 2006;12:198–207. 10.1177/1078390306294462

[235] Happell B, Koehn S Seclusion as a necessary intervention: the relationship between burnout, job satisfaction and therapeutic optimism and Justification for the use of seclusion. J Adv Nurs 2011;67:1222–31. 10.1111/j.1365-2648.2010.05570.x 21261695

[236] Johnson ME, Delaney KR Keeping the unit safe: a grounded theory study. J Am Psychiatr Nurses Assoc 2006;12:13–21. 10.1177/1078390306286440

[237] Jonker EJ, Goossens PJJ, Steenhuis IHM, et al Patient aggression in clinical psychiatry: perceptions of mental health nurses. J Psychiatr Ment Health Nurs 2008;15:492–9. 10.1111/j.1365-2850.2008.01261.x 18638210

[238] Langan C, McDonald C Daytime night attire as a therapeutic intervention in an acute adult psychiatric in-patient unit. Psychiatric Bulletin 2008;32:221–4. 10.1192/pb.bp.107.017491

[239] Maguire T, Daffern M, Martin T Exploring nurses' and patients' perspectives of limit setting in a forensic mental health setting. Int J Ment Health Nurs 2014;23:153–60. 10.1111/inm.12034 23822138

[240] Salzmann-Erikson M, Lützén K, Ivarsson A-B, et al The core characteristics and nursing care activities in psychiatric intensive care units in Sweden. Int J Ment Health Nurs 2008;17:98–107. 10.1111/j.1447-0349.2008.00517.x 18307598

[241] Millar R, Sands N 'He did what? Well, that wasn't handed over!' Communicating risk in mental health. J Psychiatr Ment Health Nurs 2013;20:345–54. 10.1111/j.1365-2850.2012.01948.x 22827401

[242] Sjöstrand M, Sandman L, Karlsson P, et al Ethical deliberations about involuntary treatment: interviews with Swedish psychiatrists. BMC Med Ethics 2015;16:37 10.1186/s12910-015-0029-5 26016885 PMC4446957

[243] Woods P Risk assessment and management approaches on mental health units. J Psychiatr Ment Health Nurs 2013;20:807–13. 10.1111/jpm.12022 23205535

[244] Cowan D, Brunero S, Luo X, et al Developing a guideline for structured content and process in mental health nursing handover. Int J Ment Health Nurs 2018;27:429–39. 10.1111/inm.12337 28401728

[245] Cullen SW, Nath SB, Marcus SC Toward understanding errors in inpatient psychiatry: a qualitative inquiry. Psychiatr Q 2010;81:197–205. 10.1007/s11126-010-9129-z 20204514

[246] Gifford ML, Anderson JE Barriers and motivating factors in reporting incidents of assault in mental health care. J Am Psychiatr Nurses Assoc 2010;16:288–98. 10.1177/1078390310384862 21659279

[247] Martin T, Daffern M Clinician perceptions of personal safety and confidence to manage inpatient aggression in a forensic psychiatric setting. J Psychiatr Ment Health Nurs 2006;13:90–9. 10.1111/j.1365-2850.2006.00920.x 16441399

[248] Mezey G, Hassell Y, Bartlett A Safety of women in mixed-sex and single-sex medium secure units: staff and patient perceptions. Br J Psychiatry 2005;187:579–82. 10.1192/bjp.187.6.579 16319412

[249] Brennan G, Flood C, Bowers L Constraints and blocks to change and improvement on acute psychiatric wards - lessons from the City Nurses project. J Psychiatr Ment Health Nurs 2006;13:475–82. 10.1111/j.1365-2850.2006.00956.x 16965464

[250] Kuosmanen A, Tiihonen J, Repo-Tiihonen E, et al Changes in patient safety culture: a patient safety intervention for Finnish forensic psychiatric hospital staff. J Nurs Manag 2019;27:848–57. 10.1111/jonm.12760 30784144

[251] Lavelle M, Attoe C, Tritschler C, et al Managing medical emergencies in mental health settings using an interprofessional in-situ simulation training programme: a mixed methods evaluation study. Nurse Educ Today 2017;59:103–9. 10.1016/j.nedt.2017.09.009 28968516

[252] Bowers L, Simpson A, Eyres S, et al Serious untoward incidents and their aftermath in acute inpatient psychiatry: the Tompkins acute ward study. Int J Ment Health Nurs 2006;15:226–34. 10.1111/j.1447-0349.2006.00428.x 17064318

[253] Gabrielsson S, Looi G-ME, Zingmark K, et al Knowledge of the patient as decision-making power: staff members' perceptions of interprofessional collaboration in challenging situations in psychiatric inpatient care. Scand J Caring Sci 2014;28:784–92. 10.1111/scs.12111 24400837

[254] Ward L Ready, aim fire! mental health nurses under siege in acute inpatient facilities. Issues Ment Health Nurs 2013;34:281–7. 10.3109/01612840.2012.742603 23566191

[255] Ryan P, Anczewska M, Laijarvi H, et al Demographic and situational variations in levels of burnout in European mental health services: a comparative study. Diversity Health Soc Care 2007;4:101–12.

[256] Nathan R, Brown A, Redhead K, et al Staff responses to the therapeutic environment: a prospective study comparing burnout among nurses working on male and female wards in a medium secure unit. J Forens Psychiatry Psychol 2007;18:342–52. 10.1080/14789940701441136

[257] Vlayen A et al A nationwide Hospital survey on patient safety culture in Belgian hospitals: setting priorities at the Launch of a 5-year patient safety plan. BMJ Publishing Group Ltd 2012. 10.1136/bmjqs-2011-051607 22927488

[258] Ajalli A et al Explanation of patient safety provided by nurses in inpatient psychiatric wards in Iran: a qualitative study. Iranian Journal of Psychiatry and Behavioral Sciences 2018;12.

[259] Wood D, Pistrang N A safe place? service users' experiences of an acute mental health ward. J Community Appl Soc Psychol 2004;14:16–28. 10.1002/casp.755

[260] Jones J, NOLAN P, BOWERS L, et al Psychiatric wards: places of safety? J Psychiatr Ment Health Nurs 2010;17:124–30. 10.1111/j.1365-2850.2009.01482.x 20465757

[261] Ireland CA, Ireland JL, Jones NS, et al Predicting security incidents in high secure male psychiatric care. Int J Law Psychiatry 2019;64:40–52. 10.1016/j.ijlp.2019.01.004 31122639

[262] Pelto-Piri V, Wallsten T, Hylén U, et al Feeling safe or unsafe in psychiatric inpatient care, a hospital-based qualitative interview study with inpatients in Sweden. Int J Ment Health Syst 2019;13:23 10.1186/s13033-019-0282-y 30996733 PMC6452515

[263] Haines A, Brown A, McCabe R, et al Factors impacting perceived safety among staff working on mental health wards. BJPsych Open 2017;3:204–11. 10.1192/bjpo.bp.117.005280 28904814 PMC5584653

[264] Kanerva A, Kivinen T, Lammintakanen J Communication elements supporting patient safety in psychiatric inpatient care. J Psychiatr Ment Health Nurs 2015;22:298–305. 10.1111/jpm.12187 25689543

[265] Kuosmanen A, Tiihonen J, Repo-Tiihonen E, et al Patient safety culture in two Finnish state-run forensic psychiatric hospitals. J Forensic Nurs 2013;9:207–16. 10.1097/JFN.0b013e318281068c 24256983

[266] Wu J-C, Tung T-H, Chen PY, et al Determinants of workplace violence against clinical physicians in hospitals. J Occup Health 2015;57:540–7. 10.1539/joh.15-0111-OA 26423827 PMC6706174

[267] Kanerva A, Lammintakanen J, Kivinen T Nursing staff's perceptions of patient safety in psychiatric inpatient care. Perspect Psychiatr Care 2016;52:25–31. 10.1111/ppc.12098 25623953

[268] Stead K, Kumar S, Schultz TJ, et al Teams communicating through STEPPS. Medical Journal of Australia 2009;190:S128–32. 10.5694/j.1326-5377.2009.tb02619.x 19485861

[269] Mahoney JS, Ellis TE, Garland G, et al Supporting a psychiatric hospital culture of safety. J Am Psychiatr Nurses Assoc 2012;18:299–306. 10.1177/1078390312460577 22967939

[270] Higgins N, Meehan T, Dart N, et al Implementation of the Safewards model in public mental health facilities: a qualitative evaluation of staff perceptions. Int J Nurs Stud 2018;88:114–20. 10.1016/j.ijnurstu.2018.08.008 30236863

[271] Bowers L, Gournay K, Duffy D Suicide and self-harm in inpatient psychiatric units: a national survey of observation policies. J Adv Nurs 2000;32:437–44. 10.1046/j.1365-2648.2000.01510.x 10964193

[272] O'Brien L, Cole R Mental health nursing practice in acute psychiatric close-observation areas. Int J Ment Health Nurs 2004;13:89–99. 10.1111/j.1440-0979.2004.00324.x 15318903

[273] Stein WM The use of discharge risk assessment tools in general psychiatric services in the UK. J Psychiatr Ment Health Nurs 2002;9:713–24. 10.1046/j.1365-2850.2002.00495.x 12472825

[274] Stübner S, GROß G, Nedopil N Inpatient risk management with mentally ill offenders: results of a survey on clinical decision-making about easing restrictions. Criminal Behaviour and Mental Health 2006;16:111–23. 10.1002/cbm.619 16755523

[275] O'Neill C, Heffernan P, Goggins R, et al Long-Stay forensic psychiatric inpatients in the Republic of ireland: aggregated needs assessment. Ir J Psychol Med 2003;20:119–25. 10.1017/S0790966700007916 30308720

[276] Rees P, Manthorpe J Managers' and staff experiences of adult protection allegations in mental health and learning disability residential services: a qualitative study. Br J Soc Work 2010;40:513–29. 10.1093/bjsw/bcn146

[277] Koukia E, Giannouli E, Gonis N, et al Security rules and banned items in psychiatric acute admission wards in Athens, Greece. Int J Ment Health Nurs 2010;19:428–36. 10.1111/j.1447-0349.2010.00695.x 21054729

[278] Silvana S, Laura F, Ursula DF, et al Ergonomics in the psychiatric ward towards workers or patients? Work 2012;41 Suppl 1:1832–5. 10.3233/WOR-2012-0393-1832 22316981

[279] True G, Frasso R, Cullen SW, et al Adverse events in Veterans Affairs inpatient psychiatric units: staff perspectives on contributing and protective factors. Gen Hosp Psychiatry 2017;48:65–71. 10.1016/j.genhosppsych.2017.07.001 28843113 PMC5605148

[280] Vandewalle J, Malfait S, Eeckloo K, et al Patient safety on psychiatric wards: a cross-sectional, multilevel study of factors influencing nurses' willingness to share power and responsibility with patients. Int J Ment Health Nurs 2018;27:877–90. 10.1111/inm.12376 28795468

[281] Gerace A, Muir-Cochrane E Perceptions of nurses working with psychiatric consumers regarding the elimination of seclusion and restraint in psychiatric inpatient settings and emergency departments: an Australian survey. Int J Ment Health Nurs 2019;28:209–25. 10.1111/inm.12522 30019798 PMC7818138

[282] Vahidi M, Ebrahimi H, Areshtanab HN, et al Therapeutic relationships and safety of care in Iranian psychiatric inpatient units. Issues Ment Health Nurs 2018;39:967–76. 10.1080/01612840.2018.1485795 30204047

[283] Holth F, Walby F, Røstbakken T, et al Extreme challenges: psychiatric inpatients with severe self-harming behavior in Norway: a national screening investigation. Nord J Psychiatry 2018;72:605–12. 10.1080/08039488.2018.1511751 30348040

[284] de Jonghe-Rouleau AP, Pot AM, de Jonghe JFM Self-Injurious behaviour in nursing home residents with dementia. Int J Geriatr Psychiatry 2005;20:651–7. 10.1002/gps.1337 16021657

[285] Sansone RA, McLean JS, Wiederman MW The relationship between medically self-sabotaging behaviors and borderline personality disorder among psychiatric inpatients. Prim Care Companion J Clin Psychiatry 2008;10:448–52. 10.4088/PCC.v10n0604 19287553 PMC2644476

[286] Gough K, Hawkins A Staff attitudes to Self‐harm and its management in a forensic psychiatric service. The British Journal of Forensic Practice 2000;2:22–8. 10.1108/14636646200000030

[287] O'Donovan A Pragmatism rules: the intervention and prevention strategies used by psychiatric nurses working with non-suicidal self-harming individuals. J Psychiatr Ment Health Nurs 2007;14:64–71. 10.1111/j.1365-2850.2007.01044.x 17244007

[288] Tofthagen R, Talseth A-G, Fagerström L Mental Health Nurses’ Experiences of Caring for Patients Suffering from Self-Harm. Nurs Res Pract 2014;2014:1–10. 10.1155/2014/905741 PMC4248333 25512876

[289] Lundegaard Mattson Åse, Binder P-E, Mattson L A qualitative exploration of how health care workers in an inpatient setting in Norway experience working with patients who self-injure. Nordic Psychology 2012;64:272–90.

[290] Thomas JB, Haslam CO How people who self-harm negotiate the inpatient environment: the mental healthcare workers perspective. Journal of Psychiatric & Mental Health Nursing (John Wiley & Sons, Inc 2017;24:480–90. 10.1111/jpm.12384 28294466

[291] Shaw DG, Sandy PT Mental health nurses' attitudes toward self-harm: Curricular implications. Health Sa Gesondheid 2016;21.

[292] Sandy PT The use of observation on patients who self-harm: Lessons from a learning disability service. Health Sa Gesondheid 2016;21.

[293] James K, Samuels I, Moran P, et al Harm reduction as a strategy for supporting people who self-harm on mental health wards: the views and experiences of practitioners. J Affect Disord 2017;214:67–73. 10.1016/j.jad.2017.03.002 28284098

[294] Brown J, Beail N Self-Harm among people with intellectual disabilities living in secure service provision: a qualitative exploration. Journal of Applied Research in Intellectual Disabilities 2009;22:503–13. 10.1111/j.1468-3148.2009.00504.x

[295] Lindgren B-M, Aminoff C, Hällgren Graneheim U Features of everyday life in psychiatric inpatient care for self-harming: an observational study of six women. Issues Ment Health Nurs 2015;36:82–8. 10.3109/01612840.2014.941077 25625707

[296] Weber MT Triggers for self-abuse: a qualitative study. Arch Psychiatr Nurs 2002;16:118–24. 10.1053/apnu.2002.32948 12037797

[297] Gibson J, Booth R, Davenport J, et al Dialectical behaviour therapy-informed skills training for deliberate self-harm: a controlled trial with 3-month follow-up data. Behav Res Ther 2014;60:8–14. 10.1016/j.brat.2014.06.007 25036538

[298] Booth R, Keogh K, Doyle J, et al Living through distress: a skills training group for reducing deliberate self-harm. Behav Cogn Psychother 2014;42:156–65. 10.1017/S1352465812001002 23218099

[299] Bowers L, Whittington R, Nolan P, et al Relationship between service ecology, special observation and self-harm during acute in-patient care: City-128 study. Br J Psychiatry 2008;193:395–401. 10.1192/bjp.bp.107.037721 18978321

[300] Kool N, van Meijel B, Koekkoek B, et al Improving communication and practical skills in working with inpatients who self-harm: a pre-test/post-test study of the effects of a training programme. BMC Psychiatry 2014;14:64 10.1186/1471-244X-14-64 24592861 PMC3975943

[301] Drew BL No-suicide contracts to prevent suicidal behavior in inpatient psychiatric settings. Journal of the American Psychiatric Nurses Association 1999;5:23–8.

[302] Sjöström N, Hetta J, Waern M Sense of coherence and suicidality in suicide attempters: a prospective study. J Psychiatr Ment Health Nurs 2012;19:62–9. 10.1111/j.1365-2850.2011.01755.x 22074158

[303] Swogger MT, Van Orden KA, Conner KR The relationship of outwardly directed aggression to suicidal ideation and suicide attempts across two high-risk samples. Psychol Violence 2014;4:184–95. 10.1037/a0033212 26180654 PMC4500128

[304] Hill RM, Hatkevich CE, Kazimi I, et al The Columbia-Suicide severity rating scale: associations between interrupted, aborted, and actual suicide attempts among adolescent inpatients. Psychiatry Res 2017;255:338–40. 10.1016/j.psychres.2017.06.014 28601718

[305] Inoue K, Kawanishi C, Otsuka K, et al A large-scale survey of inpatient suicides: comparison between medical and psychiatric settings. Psychiatry Res 2017;250:155–8. 10.1016/j.psychres.2017.01.076 28161611

[306] Ellis TE, Green KL, Allen JG, et al Collaborative assessment and management of suicidality in an inpatient setting: results of a pilot study. Psychotherapy 2012;49:72–80. 10.1037/a0026746 22369081 PMC3752846

[307] Ellis TE, Rufino KA, Allen JG, et al Impact of a Suicide-Specific intervention within inpatient psychiatric care: the collaborative assessment and management of suicidality. Suicide Life Threat Behav 2015;45:556–66. 10.1111/sltb.12151 25581595

[308] Awenat YF, Peters S, Gooding PA, et al A qualitative analysis of suicidal psychiatric inpatients views and expectations of psychological therapy to counter suicidal thoughts, acts and deaths. BMC Psychiatry 2018;18:334 10.1186/s12888-018-1921-6 30326878 PMC6192165

[309] Cleary M, Jordan R, Horsfall J, et al Suicidal patients and special observation. J Psychiatr Ment Health Nurs 1999;6:461–7. 10.1046/j.1365-2850.1999.00246.x 10818869

[310] Takahashi C, Chida F, Nakamura H, et al The impact of inpatient suicide on psychiatric nurses and their need for support. BMC Psychiatry 2011;11:38 10.1186/1471-244X-11-38 21385448 PMC3063822

[311] Vråle GB, Steen E The dynamics between structure and flexibility in constant observation of psychiatric inpatients with suicidal ideation. J Psychiatr Ment Health Nurs 2005;12:513–8. 10.1111/j.1365-2850.2005.00854.x 16164500

[312] Vandewalle J, Beeckman D, Van Hecke A, et al ‘Promoting and preserving safety and a life‐oriented perspective’: A qualitative study of nurses’ interactions with patients experiencing suicidal ideation. Int J Ment Health Nurs 2019;28:1122–34. 10.1111/inm.12623 31184415

[313] Davis SE, Williams IS, Hays LW Psychiatric inpatients' perceptions of written no-suicide agreements: an exploratory study. Suicide Life Threat Behav 2002;32:51–66. 10.1521/suli.32.1.51.22180 11931011

[314] Esposito-Smythers C, McClung TJ, Fairlie AM Adolescent perceptions of a suicide prevention group on an inpatient unit. Archives of Suicide Research 2006;10:265–75. 10.1080/13811110600582554 16717043

[315] Sun F-K, Long A, Boore J, et al Nursing people who are suicidal on psychiatric wards in Taiwan: action/interaction strategies. J Psychiatr Ment Health Nurs 2005;12:275–82. 10.1111/j.1365-2850.2005.00831.x 15876233

[316] Sun F-K, Long A, Boore J, et al Patients and nurses' perceptions of ward environmental factors and support systems in the care of suicidal patients. J Clin Nurs 2006;15:83–92. 10.1111/j.1365-2702.2005.01232.x 16390527

[317] Pfeiffer PN et al Development and pilot study of a suicide prevention intervention delivered by peer support specialists. Psychological Services 2018. 10.1037/ser0000257 PMC6494743 30382743

[318] Caspi E Does self-neglect occur among older adults with dementia when unsupervised in assisted living? an exploratory, observational study. J Elder Abuse Negl 2014;26:123–49. 10.1080/08946566.2013.830532 24499280

[319] Bowers L, HAGLUND K, MUIR-COCHRANE E, et al Locked doors: a survey of patients, staff and visitors. J Psychiatr Ment Health Nurs 2010;17:873–80. 10.1111/j.1365-2850.2010.01614.x 21078002

[320] Haglund K, von Essen L Locked entrance doors at psychiatric wards – advantages and disadvantages according to voluntarily admitted patients. Nord J Psychiatry 2005;59:511–5. 10.1080/08039480500360781 16316906

[321] Muir-Cochrane E, van der Merwe M, Nijman H, et al Investigation into the acceptability of door locking to staff, patients, and visitors on acute psychiatric wards. Int J Ment Health Nurs 2012;21:41–9. 10.1111/j.1447-0349.2011.00758.x 21740492

[322] Cowman S, Bowers L Safety and security in acute admission psychiatric wards in Ireland and London: a comparative study. J Clin Nurs 2009;18:1346–53. 10.1111/j.1365-2702.2008.02601.x 19077013

[323] Simpson A, Bowers L, Haglund K, et al The relationship between substance use and exit security on psychiatric wards. J Adv Nurs 2011;67:519–30. 10.1111/j.1365-2648.2010.05499.x 21073504

[324] Kalagi J, Otte I, Vollmann J, et al Requirements for the implementation of open door policies in acute psychiatry from a mental health professionals' and patients' view: a qualitative interview study. BMC Psychiatry 2018;18:304 10.1186/s12888-018-1866-9 30231893 PMC6147044

[325] Fletcher J, Hamilton B, Kinner S, et al Working towards least restrictive environments in acute mental health wards in the context of locked door policy and practice. Int J Ment Health Nurs 2019;28:538–50. 10.1111/inm.12559 30516024

[326] van der Schaaf PS, Dusseldorp E, Keuning FM, et al Impact of the physical environment of psychiatric wards on the use of seclusion. British Journal of Psychiatry 2013;202:142–9. 10.1192/bjp.bp.112.118422 23307922

[327] Verbeek H, Zwakhalen SMG, van Rossum E, et al Effects of small-scale, home-like facilities in dementia care on residents’ behavior, and use of physical restraints and psychotropic drugs: a quasi-experimental study. International Psychogeriatrics 2014;26:657–68. 10.1017/S1041610213002512 24411467

[328] Curtis S, Gesler W, Wood V, et al Compassionate containment? balancing technical safety and therapy in the design of psychiatric wards. Soc Sci Med 2013;97:201–9. 10.1016/j.socscimed.2013.06.015 23916450

[329] Dreyfus S, Phillipson L, Fleming R Staff and family attitudes to fences as a means of detaining people with dementia in residential aged care settings: the tension between physical and emotional safety. Australian Journal of Social Issues 2018;53:107–22. 10.1002/ajs4.34

[330] Bayramzadeh S An assessment of levels of safety in psychiatric units. HERD 2017;10:66–80. 10.1177/1937586716656002 27413058

[331] Bellantonio S, Kenny AM, Fortinsky RH, et al Efficacy of a geriatrics team intervention for residents in Dementia-Specific assisted living facilities: effect on unanticipated transitions. J Am Geriatr Soc 2008;56:523–8. 10.1111/j.1532-5415.2007.01591.x 18179497

[332] Chandler G From traditional inpatient to trauma-informed treatment: transferring control from staff to patient. J Am Psychiatr Nurses Assoc 2008;14:363–71. 10.1177/1078390308326625 21665779

[333] Wilkes L, Fleming A, Wilkes BL, et al Environmental approach to reducing agitation in older persons with dementia in a nursing home. Australas J Ageing 2005;24:141–5. 10.1111/j.1741-6612.2005.00105.x

[334] Gebhardt RP, Steinert T Should severely disturbed psychiatric patients be distributed or concentrated in specialized wards? an empirical study on the effects of hospital organization on ward atmosphere, aggressive behavior, and sexual molestation. Eur Psychiatry 1999;14:291–7. 10.1016/S0924-9338(99)00168-6 10572360

[335] Stolker JJ, Nijman HLI, Zwanikken P-H Are patients' views on seclusion associated with lack of privacy in the ward? Arch Psychiatr Nurs 2006;20:282–7. 10.1016/j.apnu.2006.05.004 17145456

[336] Kulkarni J, Gavrilidis E, Lee S, et al Establishing female-only areas in psychiatry wards to improve safety and quality of care for women. Australasian Psychiatry 2014;22:551–6. 10.1177/1039856214556322 25358653

[337] Bowers L, Crowder M Nursing staff numbers and their relationship to conflict and containment rates on psychiatric wards—A cross sectional time series poisson regression study. Int J Nurs Stud 2012;49:15–20. 10.1016/j.ijnurstu.2011.07.005 21813126

[338] Triplett P, Dearholt S, Cooper M, et al The milieu manager: a nursing staffing strategy to reduce observer use in the acute psychiatric inpatient setting. J Am Psychiatr Nurses Assoc 2017;23:422–30. 10.1177/1078390317723709 28754070

[339] Hunt IM, Windfuhr K, Shaw J, et al Ligature points and ligature types used by psychiatric inpatients who die by hanging: a national study. Crisis 2012;33:87–94. 10.1027/0227-5910/a000117 22343063

[340] Ruzić K, Francisković T, Suković Z, et al Aggresiveness in institutionalised schizophrenic patients and the selection of antipsychotics. Coll Antropol 2011;35:265–9. 21648345

[341] Rodríguez-Leal CM, López-Lunar E, Carrascosa-Bernáldez JM, et al Electrocardiographic surveillance in a psychiatric institution: avoiding iatrogenic cardiovascular death. Int J Psychiatry Clin Pract 2017;21:64–6. 10.1080/13651501.2016.1234623 27686282

[342] Seemüller F, Riedel M, Obermeier M, et al The controversial link between antidepressants and suicidality risks in adults: data from a naturalistic study on a large sample of in-patients with a major depressive episode. Int. J. Neuropsychopharm. 2009;12:181–9. 10.1017/S1461145708009139 18662490

[343] Haw C, Stubbs J, Dickens GL Barriers to the reporting of medication administration errors and near misses: an interview study of nurses at a psychiatric hospital. J Psychiatr Ment Health Nurs 2014;21:n/a–805. 10.1111/jpm.12143 24646372

[344] Soerensen AL et al The medication process in a psychiatric Hospital: are errors a potential threat to patient safety? Risk management and healthcare policy 2013;6:23. 24049464 10.2147/RMHP.S47723 PMC3775703

[345] Keers RN, Plácido M, Bennett K, et al What causes medication administration errors in a mental health hospital? A qualitative study with nursing staff. PLoS One 2018;13:e0206233 10.1371/journal.pone.0206233 30365509 PMC6203370

[346] Bademli K, Buldukoglu K Oral medication management in inpatient psychiatric care in turkey. J Psychiatr Ment Health Nurs 2009;16:355–62. 10.1111/j.1365-2850.2009.01384.x 19383014

[347] Prins MC, Drenth-van Maanen AC, Kok RM, et al Use of a structured medication history to establish medication use at admission to an old age psychiatric clinic: a prospective observational study. CNS Drugs 2013;27:963–9. 10.1007/s40263-013-0103-9 23959814

[348] Xie N, Kalia K, Strudwick G, et al Understanding mental health nurses' perceptions of barcode medication administration: a qualitative descriptive study. Issues Ment Health Nurs 2019;40:326–34. 10.1080/01612840.2018.1528321 30917055

[349] Strudwick G, Clark C, McBride B, et al Thank you for asking: exploring patient perceptions of barcode medication administration identification practices in inpatient mental health settings. Int J Med Inform 2017;105:31–7. 10.1016/j.ijmedinf.2017.05.019 28750909

[350] Cottney A, Innes J Medication-administration errors in an urban mental health Hospital: a direct observation study. Int J Ment Health Nurs 2015;24:65–74. 10.1111/inm.12096 25394525

[351] Dickens G, Stubbs J, Haw C, Raw C Delegation of medication administration: an exploratory study. Nursing Standard 2008;22:35–40. 10.7748/ns2008.02.22.22.35.c6356 18333555

[352] Haw C, Stubbs J, Dickens G An observational study of medication administration errors in old-age psychiatric inpatients. International Journal for Quality in Health Care 2007;19:210–6. 10.1093/intqhc/mzm019 17562662

[353] Cottney A Improving the safety and efficiency of nurse medication rounds through the introduction of an automated dispensing cabinet. BMJ Open Quality 2014;3. 10.1136/bmjquality.u204237.w1843 PMC4645698 26734256

[354] Dolan M, Kirwan H Survey of staff perceptions of illicit drug use among patients in a medium secure unit. Psychiatric Bulletin 2001;25:14–17. 10.1192/pb.25.1.14

[355] Hughes E, Bressington D, Sharratt K, et al Novel psychoactive substance use by mental health service consumers: an online survey of inpatient health professionals’ views and experiences. Adv Dual Diagn 2018;11:30–9. 10.1108/ADD-07-2017-0008

[356] Gonzalez-Pinto A, De Azua SR S.2.04 adherence to treatment in bipolar disorders. European Neuropsychopharmacology 2011;21:S108–9. 10.1016/S0924-977X(11)70116-X

[357] Meehan T, Morrison P, McDougall S Absconding behaviour: an exploratory investigation in an acute inpatient unit. Aust N Z J Psychiatry 1999;33:533–7. 10.1080/j.1440-1614.1999.00603.x 10483848

[358] Muir-Cochrane E, Oster C, Grotto J, et al The inpatient psychiatric unit as both a safe and unsafe place: implications for absconding. Int J Ment Health Nurs 2013;22:304–12. 10.1111/j.1447-0349.2012.00873.x 23009358

[359] Nurjannah I, FitzGerald M, Foster K Patients' experiences of absconding from a psychiatric setting in Indonesia. Int J Ment Health Nurs 2009;18:326–35. 10.1111/j.1447-0349.2009.00611.x 19740142

[360] Grotto J, Gerace A, O'Kane D, et al Risk assessment and absconding: perceptions, understandings and responses of mental health nurses. J Clin Nurs 2015;24:855–65. 10.1111/jocn.12671 25209549

[361] Bowers L, Alexander J, Gaskell C A trial of an anti-absconding intervention in acute psychiatric wards. J Psychiatr Ment Health Nurs 2003;10:410–6. 10.1046/j.1365-2850.2003.00619.x 12887632

[362] Simpson AIF, Penney SR, Fernane S, et al The impact of structured decision making on absconding by forensic psychiatric patients: results from an A-B design study. BMC Psychiatry 2015;15:103 10.1186/s12888-015-0474-1 25935745 PMC4424885

[363] Algase DL, Beattie ERA, Antonakos C, et al Wandering and the physical environment. Am J Alzheimers Dis Other Demen 2010;25:340–6. 10.1177/1533317510365342 20378834 PMC10845602

[364] Colombo M, Vitali S, Cairati M, et al Wanderers: features, findings, issues. Arch Gerontol Geriatr 2001;33:99–106. 10.1016/S0167-4943(01)00127-3 11431052

[365] Hunt IM, Clements C, Saini P, et al Suicide after absconding from inpatient care in England: an exploration of mental health professionals’ experiences. Journal of Mental Health 2016;25:245–53. 10.3109/09638237.2015.1124394 27150467

[366] Hunt IM, Windfuhr K, Swinson N, et al Suicide amongst psychiatric in-patients who abscond from the ward: a national clinical survey. BMC Psychiatry 2010;10:14 10.1186/1471-244X-10-14 20128891 PMC2845552

[367] Nijman H et al Door locking and exit security measures on acute psychiatric admission wards. Journal of Psychiatric and Mental Health Nursing 2011;18): :614–21. 21848596 10.1111/j.1365-2850.2011.01716.x

[368] Whaley AL A two-stage method for the study of cultural bias in the diagnosis of schizophrenia in African Americans. Journal of Black Psychology 2004;30:167–86. 10.1177/0095798403262062

[369] Whaley AL Cultural mistrust and the clinical diagnosis of paranoid schizophrenia in African American patients. J Psychopathol Behav Assess 2001;23:93–100. 10.1023/A:1010911608102

[370] Green R, Shelly C, Gibb J, et al Implementing seclusion in forensic mental health care: a qualitative study of staff decision making. Arch Psychiatr Nurs 2018;32:764–8. 10.1016/j.apnu.2018.04.008 30201206

[371] Koukia E, Madianos MG, Katostaras T “On the spot” interventions by mental health nurses in inpatient psychiatric wards in Greece. Issues Ment Health Nurs 2009;30:327–36. 10.1080/01612840902754586 19437252

[372] Lindsey PL Psychiatric nurses’ decision to restrain: the association between empowerment and individual factors. Journal of psychosocial nursing and mental health services 2009;47:41–9. 10.3928/02793695-20090730-02 19772250

[373] Mann-Poll PS, Smit A, de Vries WJ, et al Factors contributing to mental health professionals' decision to use seclusion. Psychiatric Services 2011;62:498–503. 10.1176/ps.62.5.pss6205_0498 21532075

[374] Marangos-Frost S, Wells D Psychiatric nurses' thoughts and feelings about restraint use: a decision dilemma. J Adv Nurs 2000;31:362–9. 10.1046/j.1365-2648.2000.01290.x 10672094

[375] Brown B, Rakow T Understanding clinicians' use of cues when assessing the future risk of violence: a clinical judgement analysis in the psychiatric setting. Clin Psychol Psychother 2016;23:125–41. 10.1002/cpp.1941 25652696

[376] Fuller J, Cowan J Risk assessment in a multi-disciplinary forensic setting: clinical judgement revisited. The Journal of Forensic Psychiatry 1999;10:276–89. 10.1080/09585189908403681

[377] Abraham S Managing patient falls in psychiatric inpatient units. Health Care Manag 2016;35:21–7. 10.1097/HCM.0000000000000094 27892909

[378] Fonad E, Emami A, Wahlin T-BR, et al Falls in somatic and dementia wards at community care units. Scand J Caring Sci 2009;23:2–10. 10.1111/j.1471-6712.2007.00574.x 19055593

[379] Tängman S, Eriksson S, Gustafson Y, et al Precipitating factors for falls among patients with dementia on a psychogeriatric ward. International Psychogeriatrics 2010;22:641–9. 10.1017/S1041610209991724 20122302

[380] Garfinkel D, Radomislsky Z, Jamal S, et al High efficacy for hip protectors in the prevention of hip fractures among elderly people with dementia. J Am Med Dir Assoc 2008;9:313–8. 10.1016/j.jamda.2007.10.011 18519111

[381] Holmes D et al An evaluation of a monitoring system intervention: falls, injuries, and affect in nursing homes. Clinical nursing research 2007;16:317–35. 17991911 10.1177/1054773807307870

[382] Powell-Cope G, Quigley P, Besterman-Dahan K, et al A qualitative understanding of patient falls in inpatient mental health units. J Am Psychiatr Nurses Assoc 2014;20:328–39. 10.1177/1078390314553269 25288601

[383] Li PH et al Infection preventionists' challenges in psychiatric clinical settings. American Journal of Infection Control 2019;47:123–7. 30314748 10.1016/j.ajic.2018.08.010

[384] D'Lima D, Crawford MJ, Darzi A, et al Patient safety and quality of care in mental health: a world of its own? BJPsych Bulletin 2017;41:241–3. 10.1192/pb.bp.116.055327 29018546 PMC5623880

[385] Sakinofsky I Preventing suicide among inpatients. The Canadian Journal of Psychiatry 2014;59:131–40. 10.1177/070674371405900304 24881161 PMC4079240

[386] Leape L, Berwick D, Clancy C, et al Transforming healthcare: a safety imperative. Qual Saf Health Care 2009;18:424–8. 10.1136/qshc.2009.036954 19955451

[387] Gandhi TK, Kaplan GS, Leape L, et al Transforming concepts in patient safety: a progress report. BMJ Qual Saf 2018;27:1019–26. 10.1136/bmjqs-2017-007756 PMC6288701 30018115

[388] Schwappach DLB Review: engaging patients as vigilant partners in safety: a systematic review. Med Care Res Rev 2010;67:119–48. 10.1177/1077558709342254 19671916

[389] Davis RE, Sevdalis N, Vincent CA Patient involvement in patient safety: how willing are patients to participate? BMJ Qual Saf 2011;20:108–14. 10.1136/bmjqs.2010.041871 21228083

[390] Institute, N.P.S.F.s.L.L. Safety is personal: partnering with patients and families for the safest care. Moston: MA.: National Patient Safety Foundation, 2014.

[391] Providers N Funding for mental health at a local level: Unpicking the variation. NHS Providers 2016.

[392] Prince M, Patel V, Saxena S, et al No health without mental health. The Lancet 2007;370:859–77. 10.1016/S0140-6736(07)61238-0 17804063

[393] Unsworth J, McKeever M, Kelleher M Recognition of physical deterioration in patients with mental health problems: the role of simulation in knowledge and skill development. J Psychiatr Ment Health Nurs 2012;19:536–45. 10.1111/j.1365-2850.2011.01828.x 22074049

[394] Cooper JB, Gaba DM, Liang B, et al The National patient safety Foundation agenda for research and development in patient safety. MedGenMed 2000;2:E38. 11104484

[395] Institute of Medicine Committee on Quality of Health Care in,, A in To Err is Human: Building a Safer Health System, L.T. Kohn, J.M. Corrigan, and M.S. Donaldson, Editors. 2000, National Academies Press (US) Copyright 2000 by the National Academy of Sciences. All rights reserved. Washington (DC. 25077248

[396] Emanuel L, Berwick D, Conway J, et al Advances in Patient Safety: New Directions and Alternative Approaches (Vol. 1: Assessment) : What exactly is patient safety? Bethesda MD, USA: U.S. National Library of Medicine, 2008.

[397] Hox JJ, Boeije HR Data collection, primary versus secondary 2005.

[398] Appleby L, Shaw J, Amos T National confidential inquiry into suicide and homicide by people with mental illness. Br J Psychiatry 1997;170:101–2. 10.1192/bjp.170.2.101 9093494

[399] Elliott JH, Turner T, Clavisi O, et al Living systematic reviews: an emerging opportunity to narrow the evidence-practice gap. PLoS Med 2014;11:e1001603 10.1371/journal.pmed.1001603 24558353 PMC3928029

[400] Long L Routine piloting in systematic reviews—a modified approach? Syst Rev 2014;3:77 10.1186/2046-4053-3-77 25035096 PMC4108964

[401] Landis JR, Koch GG The measurement of observer agreement for categorical data. Biometrics 1977;33:159–74. 10.2307/2529310 843571

[402] Gopalakrishnan S, Ganeshkumar P Systematic reviews and meta-analysis: understanding the best evidence in primary healthcare. J Family Med Prim Care 2013;2 10.4103/2249-4863.109934 PMC3894019 24479036

[403] Dewa LH, Murray K, Thibaut B, et al Identifying research priorities for patient safety in mental health: an international expert Delphi study. BMJ Open 2018;8:e021361 10.1136/bmjopen-2017-021361 PMC5855203 29502096

