# Supplementary material for: Patient safety in inpatient mental health settings: a systematic review
Source: BMJ Open. 2019 Dec 23;9(12):e030230. doi: 10.1136/bmjopen-2019-030230 (PMC7008434; doi:10.1136/bmjopen-2019-030230)

Online supplement 1

Development of inclusion and exclusion criteria for screening

Facets

- Research
- Patient Safety
- Mental Health
- Inpatient Setting

Each one of these becomes an ***inclusion criteria*** by having an agreed definition across the research team.

Good agreement was found between the two researcher's judgements κ = 0.72 (95% CI, 0.69 to 0.74), $p < .0005$. Disagreements were discussed until consensus was reached. Subsequently, two independent reviewers screened the full text articles of the abstracts that indicated that the paper met, or could meet, the inclusion criteria. Agreement rates were calculated using the kappa statistic. Substantial agreement was reached (k=0.64 (95% CI, 0.55 to 0.73), $p < .0005$).

- Mental Health (defined as a field comprising various professions, such as psychiatry and social work, that deals with the promotion of mental and psychological well-being and the prevention, diagnosis, or treatment of mental disorders as listed in the Diagnostic and Statistical Manual 5th edition (American Psychiatric Association, 2013))
- Patient Safety (defined as "The avoidance, prevention and amelioration of adverse outcomes or injuries stemming from the process of healthcare" (Vincent, 2006))
- Research (defined as diligent and systematic inquiry or investigation into a subject in order to discover or revise facts, theories, applications etc.)
- Inpatient Setting (defined as hospital settings which provide continuous care for a period of over 24 hours)

Inclusion Criteria:

- Interventions/outcomes- Articles that report patient safety outcome measures.

Exclusion Criteria:

- Population- Articles that use the words 'forensic', 'secure', 'closed' or 'locked' can be taken to refer to an inpatient environment.

- Population- Articles that amalgamate data from both inpatient and outpatient settings such that data for an inpatient only sample is not available.

- Interventions/outcomes- Articles that solely examine the reliability or validity of risk assessment tools, with no relation to the management of the risk that the tool is measuring.

- Interventions/outcomes- Articles that do not measure patient safety outcomes (this aspect needs to be overtly expressed in the article, as an aim or research question).

- Interventions/outcomes- Clinical effectiveness versus patient safety. Clinical effectiveness concerns providing the best possible treatment for patients (e.g. with effective drug treatment) as opposed to being a patient safety issue. To be included, the treatment being studied has to be linked to a patient safety outcome/measurement (like a behaviour or adverse event).

- Interventions/outcomes- Articles reporting staff perceptions/feelings concerning safety related issues are only to be included when directly related to, or discussed in terms of, a patient safety outcome/measurement (like a behaviour or adverse event).

- Settings- Articles that are not based on purely mental health care settings (i.e. exclude general hospital wards and nursing homes that do not specify mental health specialised wards). Mental health needs to be separately examined and linked to patient safety.

- Settings- Articles based in primary care, community or social care settings.

- Settings- Articles that report on patient safety events/outcomes that occur outside of the inpatient setting.

Other general exclusion criteria:

- Articles that are reviews of any kind (including literature and systematic reviews). These will be excluded, but the reference lists will be reviewed and cross-referenced to ensure we have included all relevant articles that were used in data synthesis. Will see if grads can assist with this part of the process.

- Articles that are not empirical research; i.e. articles that do not have clearly defined hypotheses, research questions or aims that generate new primary data (data being collected for the purposes of the research). Secondary data is to be excluded, even if a new analysis has been done on it. Look for results and method sections here to ensure there were clear aims and that the data produced relates to those aims. For quantitative studies, there should be hypotheses and for qualitative studies, there should be clear aims or a research question.

- Opinion/editorials/commentaries (save any information we come across for developing our priorities in line with the expert consensus study)

- Articles not in English

- Conference abstracts

- Audits (this is not new data but routinely collected data).

- Case studies based on individual patients and their clinical presentation should be excluded. Inclusion of case study in the research facet is to pick up case studies based on health organisations or countries etc. (surrounding mental health and patient safety) not individual people.

- Reports and book abstracts

- Dissertations (not peer-reviewed).
- ‘Snapshot’ studies that provide clinical pictures of a patient group/setting (no new data).
- Epidemiological studies. Studies describing mental health conditions or the difference between similar conditions within an inpatient setting does not satisfy the patient safety facet (there is no management of patient safety).
- Ambiguities that prevent researchers from confirming any criteria (not enough resource available to contact researchers).

The process for reviewing full-text articles

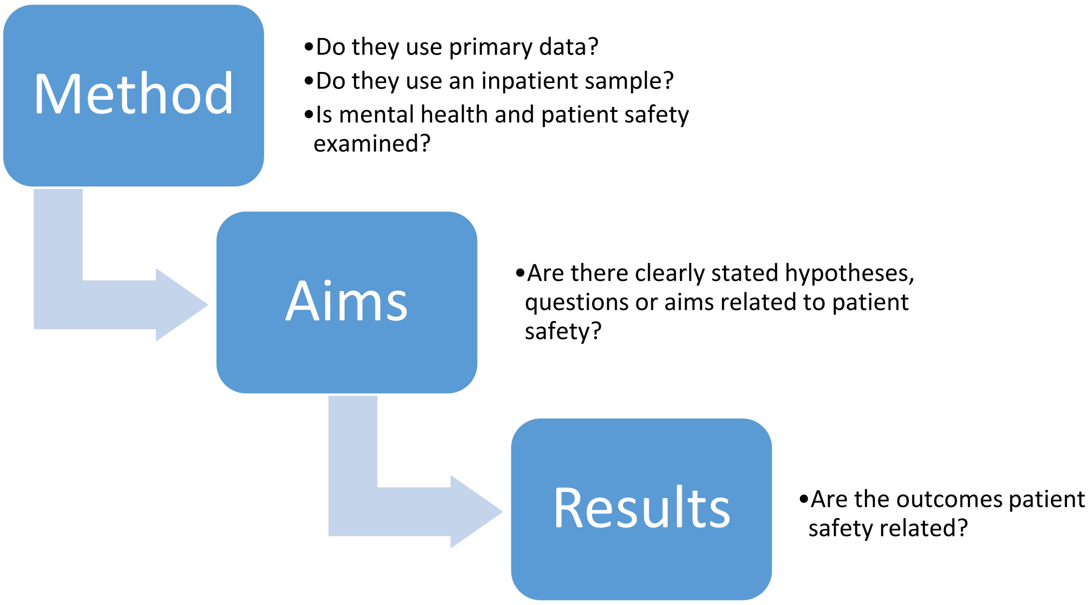

Supplement: Supplementary data [file bmjopen-2019-030230supp001.pdf]
