# Supplementary material for: Patient safety in inpatient mental health settings: a systematic review
Source: BMJ Open. 2019 Dec 23;9(12):e030230. doi: 10.1136/bmjopen-2019-030230 (PMC7008434; doi:10.1136/bmjopen-2019-030230)
Supplement: Supplementary data [file bmjopen-2019-030230supp002.pdf]

Online supplement 2

Full search strategy

**Search term**
Mental health.ts.
Mental well-being.ts.
Mental well being.ts.
Psychological well being.ts.
Psychological well-being.ts.
Mental disorder*.ts
Mental illness*.ts.
Mental disease*.ts.
Psychiatr*.ts.
Anxiety disorder*.ts.
Delirium.ts.
Dementia.ts.
Dissociative disorder*.ts.
Factitious disorder*.ts.
Impulse control disorder*.ts.
Mood disorder*.ts.
Affective disorder*.ts.
Psychotic disorder*.ts.
Depressive disorder*.ts.
Neurotic disorder*.ts.
Personality disorder*.ts.
Conduct disorder*.ts.
Schizophreni*.ts.
Somatoform disorder*.ts.
Substance related disorder*.ts.
Clinical Psychology.ts.

Impulsive behavio?r.ts.
Adjustment disorder*.ts.
Eating disorder*.ts.
Sleep disorder*.ts.
Neuros?s.ts.
Psychos?s.ts.
Delusion*.ts.
Paranoia.ts.
Hallucination*.ts.
Addiction*.ts.
Dependence.ts.
Misuse.ts.
New psychoactive substance*.ts.
Legal high*.ts.
Depression.ts.
Panic disorder*.ts.
Phobia*.ts.
Health anxiet*.ts.
Bipolar disorder*.ts.
Alcohol abuse.ts.
Alcoholism.ts.
Obsessive compulsive disorder*.ts.
Obsessive thought*.ts.
Intrusive thought*.ts.
Post traumatic stress disorder*.ts.
Post-traumatic stress disorder*.ts.
Cognitive Behavio?ral Therap*.ts.
Psychotherap*.ts.
Person centred therap*.ts.

Person-centred therap*.ts.
Counselling.ts.
Antidepressant medication*.ts.
Antipsychotic medication*.ts.
Antianxiety medication*.ts.
Psychotropic medication*.ts.
Mindfulness based cognitive therap*.ts.
Mindfulness-based cognitive therap*.ts.
Mindfulness based relapse prevention.ts.
Mindfulness-based relapse prevention.ts.
Mindfulness based stress reduction.ts.
Mindfulness-based stress reduction.ts.
Electroconvulsive therap*.ts.
Verbal de-escalation.ts.
Therapeutic.ts.
Functional Analys?s.ts.
Dialectical Behavio?r Therap*.ts.
Dysexecutive syndrome.ts.
ALL MENTAL HEALTH TERMS COMBINED WITH OR (MENTAL HEALTH FACET.ts.)
Patient safety.ts.
Adverse event*.ts.
Adverse drug event*.ts.
Sentinel event*.ts.
Incident*.ts.
Error*.ts.
Near miss*.ts.
Close call*.ts.
Never event*.ts.
Critical outcome*.ts.

Adverse outcome*.ts.
Unanticipated outcome*.ts.
Suicide*.ts.
Self-harm.ts.
Self harm.ts.
Behavio?r control.ts.
Restraint.ts.
Seclusion.ts.
Safety management.ts.
Failure to diagnose.ts.
Failure of diagnos?s.ts.
Under diagnosis.ts.
Over diagnosis.ts.
Misdiagnosis.ts.
Dual diagnos?s.ts.
Delay in diagnos?s.ts.
Wrong diagnos?s.ts.
Incorrect diagnos?s.ts.
Safety culture.ts.
Safety climate.ts.
Fall*.ts.
Slip*.ts.
Trip*.ts.
Accident prevention.ts.
Patient accident*.ts.
Patient in road traffic accident*.ts.
Collision with an object.ts.
Contact with an object.ts.
Contact with sharp*.ts.

Collision with sharp*.ts.
Exposure to hazardous substance*.ts.
Inappropriate patient handling.ts.
Inappropriate patient positioning.ts.
Elope.ts.
Wander.ts.
Runaway.ts.
Abscond*.ts.
Escorted leave.ts.
Unescorted leave.ts.
Aggressi*.ts.
Violence.ts.
Assault*.ts.
Abus*.ts.
Disruptive behavio?r.ts.
Racial attack*.ts.
Sexual attack*.ts.
Sexually inappropriate.ts.
Physical attack*.ts.
Verbal attack*.ts.
Missing patient*.ts.
Failure in access.ts.
Unexpected readmission*.ts.
Reattendance*.ts.
Unplanned admission*.ts.
Transfer to specialist care unit*.ts.
Delay in discharge.ts.
Failure to discharge.ts.
Inappropriate discharge.ts.

Planning failure.ts.
Self discharge.ts.
Self-discharge.ts.
Discharge against medical advice.ts.
Failure in referral process*.ts.
Failure to return from authorised leave.ts.
Transfer delay*.ts.
Transfer failure*.ts.
Inappropriate transfer*.ts.
Unsafe transfer*.ts.
Unsafe clinical environment*.ts.
Inappropriate clinical environment*.ts.
Inappropriate admission of a minor to an adult setting.ts.
Inappropriate transfer of a minor to an adult setting.ts.
Poor clinical assessment*.ts.
Lack of clinical assessment*.ts.
Lack of risk assessment*.ts.
Wrong scan*.ts.
Wrong x-ray*.ts.
Wrong specimen*.ts.
Inadequate scan*.ts.
Inadequate x-ray*.ts.
Inadequate specimen*.ts.
Incomplete scan*.ts.
Incomplete x-ray*.ts.
Incomplete specimen*.ts.
Mislabelled scan*.ts.
Mislabelled x-ray*.ts.
Mislabelled specimen*.ts.

Unlabelled scan*.ts.
Unlabelled x-ray*.ts.
Unlabelled specimen*.ts.
Missing scan*.ts.
Missing x-ray*.ts.
Missing specimen*.ts.
Failure to interpret test result*.ts.
Delay to interpret test result*.ts.
Failure to act on test result*.ts.
Delay to act on test result*.ts.
Failure to receive test result*.ts.
Delay to receive test result*.ts.
Incorrect test result*.ts.
Incorrect report*.ts.
Missing test result*.ts.
Missing report*.ts.
Failure to undertake test*.ts.
Delay to undertake test*.ts.
Patient confidentiality.ts.
Communication failure*.ts.
Failed communication*.ts.
Failure in communication*.ts.
Failure to receive informed consent.ts.
Inadequate handover.ts.
Documentation delay*.ts.
Mislabelled documentation.ts.
Missing documentation.ts.
Inadequate documentation.ts.
Wrong documentation.ts.

Illegible documentation.ts.
Mislabelled healthcare record*.ts.
Inadequate healthcare record*.ts.
Missing healthcare record*.ts.
Wrong healthcare record*.ts.
Illegible healthcare record*.ts.
Mislabelled referral letter*.ts.
Inadequate referral letter*.ts.
Missing referral letter*.ts.
Wrong referral letter*.ts.
Illegible referral letter*.ts.
Misfiled documentation.ts.
No access to documentation.ts.
Patient incorrectly identified.ts.
Delay in obtaining clinical assistance.ts.
Difficulty in obtaining clinical assistance.ts.
Delay in recogni?ing complication* of treatment.ts.
Failure in recogni?ing complication* of treatment.ts.
Delay in monitoring.ts.
Failure to monitor.ts.
Failure to follow up.ts.
Infection Control.ts.
Failure of sterili?ation of equipment.ts.
Contamination of equipment.ts.
Health care acquired infection*.ts.
Healthcare acquired infection*.ts.
Health care associated infection*.ts.
Healthcare associated infection*.ts.
Wound infection*.ts.

Surgical site infection*.ts.
Unsafe environment*.ts.
Inappropriate environment*.ts.
Unsafe equipment.ts.
Inappropriate equipment.ts.
Availability of equipment.ts.
Availability of bed*.ts.
Availability of IT.ts.
Staff shortage*.ts.
Unavailability of staff.ts.
Lack of skilled staff.ts.
Unskilled staff.ts.
Lack of suitably trained staff.ts.
Failure of device*.ts.
Failure of equipment.ts.
Unavailability of device*.ts.
Extended stay.ts.
Extended episode* of care.ts.
Failure to discontinue treatment*.ts.
Infusion injur*.ts.
Missing needle*.ts.
Missing swab*.ts.
Missing instrument*.ts.
Retained needle*.ts.
Retained swab*.ts.
Retained instrument*.ts.
Theatre list details incorrect.ts.
Inappropriate treatment*.ts.
Wrong treatment*.ts.

Unplanned return to theatre.ts.
Maternal death*.ts.
Anaesthetic complication*.ts.
Intensive Therapy Unit Admission*.ts.
Intensive Treatment Unit Admission*.ts.
Intensive Care Unit Admission*.ts.
Venous thromboembolism*.ts.
Pulmonary embolism*.ts.
Readmission of mother.ts.
Stillbirth*.ts.
Neonatal death*.ts.
Birth trauma*.ts.
Term baby admitted to neonatal unit.ts.
Undiagnosed f?etal abnormalit*.ts.
Pressure ulcer*.ts.
Padded room*.ts.
Ligature point*.ts.
Self-neglect.ts.
Self neglect.ts.
Splint*.ts.
Head bang*.ts.
Head-bang*.ts.
ALL PATIENT SAFETY TERMS COMBINED WITH OR (PATIENT SAFETY FACET.ts.)
Research.ts.
Academic work.ts.
Academic understanding.ts.
Theor*.ts.
Randomised controlled trial*.ts.
Controlled clinical trial*.ts.

Random allocation.ts.
Double blind method.ts.
Single blind method.ts.
Single blind stud*.ts.
Double blind stud*.ts.
Triple blind stud*.ts.
Multicentre stud*.ts.
Random sample*.ts.
Evidence base*.ts.
Evidence scan*.ts.
Systematic review*.ts.
Scoping review*.ts.
Narrative review*.ts.
Literature review*.ts.
Meta narrative*.ts.
Meta synthesi*.ts.
Meta-analys*.ts.
Clinical trial*.ts.
Placebo*.ts.
Comparative stud*.ts.
Evaluation stud*.ts.
Evaluative stud*.ts.
Descriptive stud*.ts.
Community trial*.ts.
Follow up stud*.ts.
Prospective stud*.ts.
Longitudinal stud*.ts.
Qualitative.ts.
Quantitative.ts.

Focus group*.ts.
Semi-structured interview*.ts.
Quality improvement project*.ts.
Data collection.ts.
Data analysis.ts.
Survey*.ts.
Observation*.ts.
Ethnograph*.ts.
Intervention*.ts.
Investigation*.ts.
Experiment*.ts.
Case stud*.ts.
Delphi.ts.
Nominal group technique*.ts.
Nominal group stud*.ts.
Consensus stud*.ts.
ALL RESEARCH TERMS COMBINED WITH OR (RESEARCH FACET.ts.)
Hospital*.ts.
Acute care.ts.
Secondary care.ts.
Tertiary care.ts.
Unit*.ts.
Ward*.ts.
Low secure.ts.
Medium secure.ts.
High secure.ts.
Secure facilit*.ts.
Forensic*.ts.
Inpatient*.ts.

Triage.ts.
ALL INPATIENT SETTING TERMS COMBINED WITH OR (INPATIENT SETTING FACET.ts.)
FINAL SEARCH RESULT: ALL FACETS COMBINED WITH AND (MENTAL HEALTH AND PATIENT SAFETY AND RESEARCH AND INPATIENT SETTING.ts.)

Final search string:

((Patient safety or Adverse event* or Adverse drug event* or Sentinel event* or Incident* or Error* or Near miss* or Close call* or Never event* or Critical outcome* or Adverse outcome* or Unanticipated outcome* or Suicide* or Self-harm or Self harm or Behavio?r control or Restraint or Seclusion or Safety management or Failure to diagnose or Failure of diagnos?s or Under diagnosis or Over diagnosis or Misdiagnosis or Dual diagnos?s or Delay in diagnos?s or Wrong diagnos?s or Incorrect diagnos?s or Safety culture or Safety climate or Fall* or Slip* or Trip* or Falling or Slipping or Tripping or Accident prevention or Patient accident* or Patient in road traffic accident* or Collision with an object or Contact with an object or Contact with sharp* or Collision with sharp* or Exposure to hazardous substance* or Inappropriate patient handling or Inappropriate patient positioning or Elope or Wander or Runaway or Abscond* or Escorted leave or Unescorted leave or Aggressi* or Violence or Assault* or Abus* or Disruptive behavio?r or Racial attack* or Sexual attack* or Sexually inappropriate or Physical attack* or Verbal attack* or Missing patient* or Failure in access or Unexpected readmission* or Reattendance* or Unplanned admission* or Transfer to specialist care unit* or Delay in discharge or Failure to discharge or Inappropriate discharge or Planning failure or Self discharge or Self-discharge or Discharge against medical advice or Failure in referral process* or Failure to return from authorised leave or Transfer delay* or Transfer failure* or Inappropriate transfer* or Unsafe transfer* or Unsafe clinical environment* or Inappropriate clinical environment* or Inappropriate admission of a minor to an adult setting or Inappropriate transfer of a minor to an adult setting or Poor clinical assessment* or Lack of clinical assessment* or Lack of risk assessment* or Wrong scan* or Wrong x-ray* or Wrong specimen* or Inadequate scan* or Inadequate x-ray* or Inadequate specimen* or Incomplete scan* or Incomplete x-ray* or Incomplete specimen* or Mislabelled scan* or Mislabelled x-ray* or Mislabelled specimen* or Unlabelled scan* or Unlabelled x-ray* or Unlabelled specimen* or Missing scan* or Missing x-ray* or Missing specimen* or Failure to interpret test result* or Delay to interpret test result* or Failure to act on test result* or Delay to act on test result* or Failure to receive test result* or Delay to receive test result* or Incorrect test result* or Incorrect report* or Missing test result* or Missing report* or Failure to undertake test* or Delay to undertake test* or Patient confidentiality or

Communication failure* or Failed communication* or Failure in communication* or Failure to receive informed consent or Inadequate handover of care or Inadequate handover or Documentation delay* or Mislabelled documentation or Missing documentation or Inadequate documentation or Wrong documentation or Illegible documentation or Mislabelled healthcare record* or Inadequate healthcare record* or Missing healthcare record* or Wrong healthcare record* or Illegible healthcare record* or Mislabelled referral letter* or Inadequate referral letter* or Missing referral letter* or Wrong referral letter* or Illegible referral letter* or Misfiled documentation or No access to documentation or Patient incorrectly identified or Delay in obtaining clinical assistance or Difficulty in obtaining clinical assistance or Delay in recogni?ing complication* of treatment or Failure in recogni?ing complication* of treatment or Delay in monitoring or Failure to monitor or Failure to follow up or Infection Control or Failure of sterili?ation of equipment or Contamination of equipment or Healthcare associated infection or Healthcare acquired infection or Health care associated infection or Health care acquired infection or Health care acquired infection* or Healthcare acquired infection* or Health care associated infection* or Healthcare associated infection* or Wound infection* or Surgical site infection* or Unsafe environment* or Inappropriate environment* or Unsafe equipment or Inappropriate equipment or Availability of equipment or Availability of bed* or Availability of IT or Staff shortage* or Unavailability of staff or Lack of skilled staff or Unskilled staff or Lack of suitably trained staff or Failure of device* or Failure of equipment or Unavailability of device* or Extended stay or Extended episode of care or Extended episode* of care or Failure to discontinue treatment* or Infusion injur* or Missing needle* or Missing swab* or Missing instrument* or Retained needle* or Retained swab* or Retained instrument* or Theatre list details incorrect or Inappropriate treatment* or Wrong treatment* or Unplanned return to theatre or Maternal death* or Anaesthetic complication* or Intensive Therapy Unit Admission* or Intensive Treatment Unit Admission* or Intensive Care Unit Admission* or Venous thromboembolism or Venous thromboembolism* or Pulmonary embolism* or Readmission of mother or Stillbirth* or Neonatal death* or Birth trauma* or Term baby admitted to neonatal unit or Undiagnosed f?etal abnormalit* or Pressure ulcer* or Padded room* or Ligature point* or Self-neglect or Self neglect or Splint* or Head bang* or Head-bang* or Patient safety) and (Research or Academic work or Academic understanding or Theor* or Randomised controlled trial* or Controlled clinical trial* or Random allocation or Double blind method or Single blind method or Single blind stud* or Double blind stud* or Triple blind stud* or Multicentre stud* or Random sample* or Evidence base* or Evidence scan* or Systematic review* or Scoping review* or Narrative review* or Literature review* or Meta narrative* or Meta synthesi* or Meta-analys* or Clinical trial* or Placebo* or Research design or Comparative stud* or Evaluation stud* or Evaluative stud* or Descriptive stud* or Community trial* or Follow up stud* or Prospective stud* or Longitudinal stud* or Qualitative or Quantitative or Focus

group* or Semi-structured interview* or Quality improvement project* or Data collection or Data analysis or Survey* or Observation* or Ethnograph* or Intervention* or Investigation* or Experiment* or Case stud* or Delphi or Nominal group technique* or Nominal group stud* or Consensus stud* or (Research or Descriptive research methods or Psychological research or qualitative research or mixed methods research or Experimental research or Applied research or research methodology or Medical research or Research implementation or Research design or Scientific research or Research findings or Sociological research or Research reports or Social research or Empirical research methods or Research methods or Social service research or Social welfare research or quantitative research or Research projects or Health services research)) and (Mental health or Mental well-being or Mental well being or Psychological well being or Psychological well-being or Mental disorder* or Mental illness* or Mental disease* or Anxiety disorder* or Delirium or Dementia or Dissociative disorder* or Factitious disorder* or Impulse control disorder* or Mood disorder* or Affective disorder* or Psychotic disorder* or Depressive disorder* or Neurotic disorder* or Personality disorder* or Conduct disorder* or Schizophreni* or Somatoform disorder* or Substance related disorder* or Clinical Psychology or Impulsive behavio?r or Adjustment disorder* or Eating disorder* or Sleep disorder* or Neuros?s or Psychos?s or Delusion* or Paranoia or Hallucination* or Addiction* or Dependence or Misuse or New psychoactive substance* or Legal high* or Depression or Panic disorder* or Phobia* or Health anxiet* or Bipolar disorder* or Alcohol abuse or Alcoholism or Obsessive compulsive disorder* or Obsessive thought* or Intrusive thought* or Post traumatic stress disorder* or Post-traumatic stress disorder* or Cognitive Behavio?ral Therap* or Psychotherap* or Person centred therap* or Person-centred therap* or Counselling or Antidepressant medication* or Antipsychotic medication* or Antianxiety medication* or Psychotropic medication* or Mindfulness based cognitive therap* or Mindfulness-based cognitive therap* or Mindfulness based relapse prevention or Mindfulness-based relapse prevention or Mindfulness based stress reduction or Mindfulness-based stress reduction or Electroconvulsive therap* or Verbal de-escalation or Therapeutic or Functional Analys?s or Dialectical Behavio?r Therap* or Dysexecutive syndrome or Mental health or mental disorders or Psychiatr*) and (Hospital* or Acute care or Secondary care or Tertiary care or Low secure or Medium secure or High secure or Secure facilit* or Forensic* or Inpatient* or Triage or (Acute hospitals or Mental health hospitals or hospitals) or In patients or Unit* or Ward*)).ts.
