# Supplementary material for: Patient safety in inpatient mental health settings: a systematic review
Source: BMJ Open. 2019 Dec 23;9(12):e030230. doi: 10.1136/bmjopen-2019-030230 (PMC7008434; doi:10.1136/bmjopen-2019-030230)
Supplement: Supplementary data [file bmjopen-2019-030230supp003.pdf]

Online supplement 3

Research categories, subcategories by subsequent articles and reference

**Category**	**Subcategory**	**Articles** *(Reference number)*
Interpersonal Violence	Aggression	49 *([20-21, 23-25, 30, 35-38, 40-41, 44-52, 58-59, 66, 68-71, 74-79, 81-82, 86-89, 94, 97, 100-101, 104, 107-108, 112-113])*
	Violence	33 *([22, 26-29, 31-33, 43, 53-57, 64, 72-73, 80, 83-85, 90-93, 95-96, 98, 102-103,109-111])*
	Challenging Behaviour	10 *([114-123])*
	Violence and Aggression	12 *([34, 39, 42, 60-63 ,65, 67, 99, 105-106])*
	Critical Incidents	4 *([128-131])*
	Conflict	4 *([124-127])*
	Sexual Assault	2 *([133-134])*
	Agitation	1 *([135])*
	Abuse	1 *([132])*
	**Total**	**116** ***(32%)***
Coercive Interventions	Restraint	23 *([138, 148-151, 155-156, 158-159, 166-167, 172,174-176, 181-183, 193, 195-196, 199, 201])*
	Seclusion	20 *([136-137, 147, 152-153, 160-162, 169, 173, 177-180, 184-186, 191-192, 206])*
	Attitudes to Coercion	25 *([142-145, 167, 211-30])*
	Seclusion & Restraint	16 *([146, 154, 157, 163-165, 170-171, 187-190, 194, 197-198, 200])*
	Containment	4 *([202-205])*
	Process of Coercion	6 *([139-141, 231-233])*
	Alternative Interventions	1*([209])*
	Shielding	1 *([207])*
	Conflict	1 *([208])*
	Personal Factors	1 *([210])*
	**Total**	**98** ***(27%)***
Safety Culture	Process	18 *([234-251])*
	Culture	19 *([252-270])*
	Policy	8 *([271-278])*
	Building Therapeutic	2 *([281-282])*

	Relationships	
	Patient/Family Engagement	2 ([279-280])
	**Total**	**49 *(13%)***
Harm to Self	Self-harm	18 ([283-300])
	Suicidal Behaviour	17 ([301-317])
	Self-neglect	1 ([318])
	**Total**	**36 *(10%)***
Safety of the Physical Environment	Security	7 ([319-325])
	Environment Design	5 ([326-330])
	Transitions of Care	3 ([331-333])
	Patient Distribution	3 ([334-336])
	Staffing	2 ([337-338])
	Ligatures	1 ([339])
	**Total**	**21 *(6%)***
Medication Safety	Adverse Events	5 ([340-344])
	Medication Administration	5 ([348-352])
	Medication Management	3 ([345-347])
	Medication Dispensing	1 ([353])
	Adherence	1 ([356])
	Substance Use	2 ([354, 355])
	**Total**	**17 *(5%)***
Unauthorised Leave	Absconding	9 ([357-362, 365-367])
	Wandering	2 ([363-364])
	**Total**	**11 *(3%)***
Clinical Decision Making	Incident Management	5 ([370-374])
	Risk Assessment	2 ([375-376])
	Diagnosis	2 ([368-369])
	**Total**	**9 *(2%)***
Falls	Falls	4 ([377-379, 382])
	Injuries	2 ([380-381])
	**Total**	**6 *(1%)***
Infection Prevention & Control	Infection Prevention & Control	1 ([383])

	**Total**	**1 *(<1%)***
