# Supplementary material for: Patient safety in inpatient mental health settings: a systematic review
Source: BMJ Open. 2019 Dec 23;9(12):e030230. doi: 10.1136/bmjopen-2019-030230 (PMC7008434; doi:10.1136/bmjopen-2019-030230)
Supplement: Supplementary data [file bmjopen-2019-030230supp004.pdf]

Online supplement 4

Data extraction tables by research category

Interpersonal Violence

Author, year, country	Study design	Setting	Participants	Aims/ objectives related to patient safety	Outcomes related to patient safety	Study quality
Almvik, Rasmussen & Woods (2006) Norway	Observation/ Questionnaire/ Quantitative	Geriatric psychiatric wards and special care units for patients with dementia (SCU) within nursing homes (n=2)	Residents (n=82); 43.9% male; mean age 81.96 (range 56-104).	To explore violent incidents in an elderly population	39% of patients were reported to be violent. The mean score on the SOAS-R severity scale was 9.89 (median 10, SD 5.00, 95% CI 9.21–10.56). The situations where the client was denied something were the most provocative ones (37.9% of occurrences). Verbal aggression was the most frequent type of attack (58%). The staff were the most frequent targets of the violent incidents (78.8%). The incident had no consequences for the victim in 47.6% of cases. Talking to patients was the most common measure taken to stop aggression (63.7%). Most incidents occurred in the morning (42.9%).	Fair
Amoo & Fatoye (2010) Nigeria	Questionnaire/ Quantitative	Inpatient psychiatric wards of a neuropsychiatric hospital	Psychiatric inpatients (n=305); 69.8% male. Age range of the aggressive patients 13-66.	To investigate aggressive behaviour and related variables in psychiatric inpatients	43 patients manifested aggressive behaviour (13.8% of sample). Most aggressive behaviour occurred during evening and night periods (69.8% of incidents) and was evenly distributed throughout the week. Physical aggression was the most common type of behaviour (37.2%) and female nurses were the most common targets. Hallucinations were the most common probable cause (34.9%).	Fair

Arguvanli, et al. (2015) Turkey	Pre-post questionnaire/ Quantitative	Psychiatric inpatient clinics within hospitals (n=2)	Nurses (n=27). 78% female. Mean age 33.2±5.4 (range 22-47)	To evaluate the Aggression Management Training Program (AMTP) in psychiatric inpatient clinics	96.3% of participants reported that they did not receive aggression management training. Participant knowledge and functional reactions to aggression increased significantly from pre-post intervention.	Fair
Bahareethan & Shah (2000) UK	Questionnaire/ Quantitative	Continuing care psychogeriatric wards within a London hospital (n=2)	Patients (n=39); 26% male. Median age 83 (range 68-99).	To investigate aggressive behaviour and staff perception and attitudes in psychogeriatric wards	There was a moderate correlation between aggressive behaviour (RAGE scale) and staff perception of the patient (MAS scale) (r=+0.67, p<0.00001). The highest correlation was between the RAGE score and the MAS subscale of 'alienation of the patient (r=+0.85, p<0.00001).	Fair
Bennett, Ramakrishna & Maganty (2011) UK	Questionnaire/ Quantitative	A psychiatric intensive care unit (PICU) within a medium-secure unit	Nursing and multidisciplinary staff of the PICU (n=43)	To investigate staff views of different management techniques for disturbed behaviour in PICUs	Individual counselling was the preferred choice of management (n=40, mode rank given=1; mean rank given=1.25; median rank given=1; SD=0.77)	Fair
Ben-Zeev, et al. (2017) USA	Questionnaires / Quantitative	Secure hospital	Inpatients (n=27). 86% male. Mean age 33+11.13	To examine multimodal technologies to identify correlates of violence among inpatients with serious mental illness.	Self-reported delusions were associated with violent ideation (odds ratio [OR]=3.08), damaging property (OR=8.24), and physical aggression (OR=12.39). Alcohol and cigarette cravings were associated with violent ideation (OR=5.20 andOR=6.08, respectively), damaging property (OR=3.71 and OR=4.26, respectively), threatening others (OR=3.62 and OR=3.04, respectively), and physical aggression (OR=6.26, and OR=8.02, respectively). Drug cravings were associated with violent ideation (OR=2.76) and damaging property	Fair

					(OR=5.09). Decreased variability in physical activity and noisyward conditionswere associatedwith violent ideation (OR=.71 and OR=2.82, respectively).	
Berg, et al. (2013) Europe	Interviews/ Qualitative	Forensic psychiatric units (n=4) in Belgium (n=1) Finland (n=1), Netherlands (n=1) and the UK (n=1)	Staff in the units (n=58). Mean age 36 (range 20-75)	To investigate staff perceptions of aggressive behaviour in forensic psychiatric patients	Participants described aggressive behaviour as consisting of verbal, non-verbal and physical dimensions. Staff being authoritative with patients was described as provoking aggressive behaviour and more commonly leading to physical management techniques. Limit-setting situations were also identified as provoking aggressive behaviour towards staff.	Good
Berg, Kaltiala-Heino & Välimäki (2011) Europe	Interviews/ Qualitative	Forensic psychiatric units (n=4) in Belgium (n=1) Finland (n=1), Netherlands (n=1) and The UK (n=1)	Staff in the units (n=58). Mean age 36 (range 20-75)	To explore practices for managing adolescent aggressive behaviour in forensic psychiatric units	Participants favoured the use of verbal and less restrictive aggression management interventions. The use of coercive management interventions was described as a last option. Participants described using knowledge of the patient, the level of aggression, the situation development and available resources to decide on appropriate action. Countries differed on the practical responses to aggression but shared the same basic components.	Good

Bharwani, et al. (2012) USA	Observation and questionnaire/ Mixed methods	An Alzheimer unit within a continuing care retirement community	Residents of the unit (n=18). 54% female. Average age 82	To explore the benefits of a Behaviour-Based Ergonomic Therapy (BBET) program designed as an intervention for challenging behaviour	Resident falls decreased by 32.5% during the first 6 months of BBET implementation; from an average of 6.67 falls per month (n=40 in the 6 months before implementation) to 4.5 falls per month (n=27 in the first 6 months of implementation). Staff and family members commented on a reduction in agitation, including emergency calls about resident behaviour.	Fair
Biancosino, et al. (2009) Italy	Questionnaire and chart review/ Quantitative	Public (n=113) and private (n=32) acute psychiatric inpatient facilities	Psychiatric inpatients in the facilities (n=1324). 51% male. 28.2% were 35-44.	To examine the nature and predictors of aggressive behaviour in psychiatric inpatients	6.3% of patients had been physically restrained. Violent patients were more likely to be restrained than non-violent patients (up to 51.4% of this group (χ2= 203.07; p<0.001). No association with violent behaviour was found when treatment setting variables were examined (restrictiveness, standardization of the process of care, treatment, and staff number/qualification).	Fair
Bilici et al. (2016) Turkey	Quantitative	Locked psychiatric clinic	Staff (n=137). Chief nurse (n=9), resident (n=34), chief resident (n=10), assistant clinic chief (n=3) and clinic chief (3).	To examine the rates of exposure to violence including quantity, types of violence and compare to occupation types.	The majority of staff had been exposed to a violent incident (92.7%) and viewed security measures to be insufficient (87.6%). Positive correlation with the number of years working in the locked unit and number of exposures to verbal aggression (p<0.05).	Fair

Björkdahl, Hansebo & Palmstierna (2013) Sweden	Non-randomised pre-post/ Quantitative	Psychiatric inpatient units (n=41). Emergency and admission wards (n = 2), general wards (n = 30), psychiatric intensive care units (n = 2), drug and alcohol dependence wards (n = 2) and forensic wards (n = 5).	Staff in the units (n=854 before training; n=260 after). 40% male before training; 42% male after. Inpatients (n=297 before training; n=156 after). 55% male before training; 51% male after. Age range <25->40.	To explore the influence of a violence prevention and management staff-training programme on the climate related to violence management in psychiatric inpatient wards.	Staff perceived the violence management climate as significantly more positive on the trained wards compared with the wards that had not yet been trained, Mann–Whitney P=0.045. Specifically, the areas viewed more positively were: ward rules (odds [OR] = 1.97, P=0.001), the emotional regulation of staff members in challenging situations (odds [OR] = 2.24, P=0.007), the staff's interest in possible causes for patient aggression (odds [OR] = 1.64, P=0.031) and the staff's readiness to intervene at an early stage of patient aggression (odds [OR] = 2.10, P=0.001). On the trained wards, patients viewed perceptions of the staff's interest in finding possible causes for patient aggression significantly more positively (odds [OR]= 1.98, P=0.022)	Fair
Boström, et al. (2012) Canada	Questionnaire/ Mixed methods	Resident care units representing two models of residential care, Residential Alzheimer's Care Centres RACCs (n = 2) and Secured Dementia Units SDUs (n = 2).	Staff in the units (n=91). 98% female.	To explore aggressive incidents experienced by frontline staff in two types of residential care units	The most frequent type of aggression was physical assault (50% of staff reported experiencing this). 79% of incidences were officially reported by staff. Aggressive incidents were associated with working in SDUs rather than RACCs in a regression model (F =4.667, df = 6, p < 0.001). Other factors associated with aggressive incidents were: staff educational level, experience,	Fair

					exhaustion, staffing levels and staff informal interactions.	
Bowers (2009) UK	Cross-sectional/ Quantitative	Acute psychiatric wards (n=136) in 67 hospitals within 26 National Health Service (NHS) Trusts	All staff and patients within the units	To explore the effect of the positive appreciation of patients by staff, the staff's management of their own emotional reactions to patient behaviour, and the provision of an effective structure on conflict in acute psychiatric wards.	Only 18% of the variance in containment rates was explained by conflict. The regression model found that effective structure and order on the ward (the order and organization subscale and the program clarity subscale of the WAS) was the factor most strongly predictive of lower conflict and containment rates (Coefficient=-0.048, p=0.048).	Good
Bowers, et al. (2009) UK	Questionnaire/Quantitativ e	Acute psychiatric wards (n=136) in 67 hospitals within 26 National Health Service (NHS) Trusts	All staff and patients within the units	To explore the relationship of patient violence to other patient characteristics, features of the service and physical environment and staff factors.	Higher levels of aggression were associated with high patient turnover, alcohol use by patients, ward doors being locked, and higher staffing numbers (especially qualified nurses).	Fair
Calabro, Mackey & Williams (2002) USA	Pre-post evaluation/Quantitative	An acute care psychiatric hospital	Staff (n=118). 67% female. Modal age group 40-49	To evaluate the effectiveness of a training program to prevent and manage patient violence within acute psychiatric wards	There were improvements in post-test measures for: knowledge (t[109]=7.29, p < 0.001); attitude (t[109]=-5.68, p < 0.001); self-efficacy (t[114]=-2.82, p < 0.01); and intention to use the training techniques	Fair

					(t[114]=-1.99, p < 0.05).	
Camuccio, et al. (2012) Italy	Focus groups/ Qualitative	Acute psychiatric inpatient units (n=7)	Nurses (n=33). 70% female. Mean age 40.	To explore the experiences of acute psychiatric inpatient unit nurses when managing aggressive and distressed patients	The predominant theme was of fear, and this consisted of: fear of harm to self or patient; team safety; the known/unknown patient; non-psychiatric patients; and patient physique. Co-ordination with other services was expressed as key to good quality care.	Fair
Carlson, et al. (2010) USA	Observation, chart review and semi-structured interviews/ Quantitative	A psychiatric inpatient unit	Patients (n=130; n=151 admissions). 80% male. Mean age 9.64 ± 2.09	To examine the safety and efficacy of liquid risperidone to reduce rages in children admitted to a psychiatric inpatient unit.	75% of the sample had multiple rages in hospital. No adverse events related to the medication were observed. In the 16 children who had more than 3 rage outbursts during hospitalisation, there was a significant drop in duration of the outbursts from the non-medicated state to the highest dose state (t(15)=3.43, p<0.004).	Fair

Caspi (2015) USA	Observation, chart review and semi-structured interviews/ Qualitative	Special dementia care units within an assisted living residence (n=2)	Residents (n=12). 92% female. Mean age 81.	To explore aggression between residents (ABBR) with dementia within assisted living residences	For the majority of ABBR incidents there were observable causes and triggers (77% in one unit; 57% in the other). The most common cause was problematic seating arrangement (n=17). There were 12 effective staff management strategies: being alert; being proactive; being informed about aggression history; redirecting residents; offering to take a walk; separating; positioning, repositioning, or changing seating arrangement; refocusing; distracting the person; staying calm; never arguing with a resident; seeking help from other staff members.	Good
Caspi, et al. (2001) Israel	Double-blind crossover trial/ Quantitative	A maximum security unit within a mental health centre	Schizophrenic inpatients (n=30). 100% male. Mean age 37.2 ±9.6 (range 20-65)	To examine the efficacy of augmenting antipsychotic treatment with pindolol to reduce aggression in schizophrenic inpatients.	Pindolol treatment significantly reduced the number of aggressive incidents towards objects and other persons (0.59 versus 1.46, F=6.09, P<0.02; 1.96 versus 3.23, F=4.17, P<0.05, respectively); and reduced the severity of the incidents (0.89 versus 3.58, F=19.42, P<0.0001; 2.89 versus 6.85, F=10.11, P<0.004, respectively).	Fair

Chan, et al. (2005) Hong Kong	Pre-post questionnaire/ Quantitative	A developmental disability unit within a mental health hospital	Patients (n=89). Experimental group: 60% female. Control group: 59% female. Modal age group 31-40	To evaluate the impact of multisensory therapy on the challenging behaviour of patients with developmental disabilities	Both groups of patients experienced reductions in levels of challenging behaviour over time. Between the mid- and post-1 assessments, the control group had greater reductions in challenging behaviour (F=4.26, p=0.04). Between the post-2 and post-3 assessments, the experimental group had greater reductions in challenging behaviour (F=4.22, p=0.04).	Fair
Chaplin, et al. (2008) UK	Questionnaire/ Mixed methods	Older adult (OA) wards (n=75) and adults of working age (WAA) wards (n=88) within 66 English and Welsh NHS trusts and independent sector providers	Staff and patients (n=3,332)	To explore physical aggression/assault on psychiatric wards	Nurses on WAA wards were more likely to report dealing with aggressive incidents (85%, x^2=11 p=0.0006); using rapid tranquilisation (68%, x^2=73 p<0.0001); receiving adequate training in aggression management/prevention (79%, x^2=6.5 p=0.011); and having a personal alarm (86%, x^2=35 p=0.0001) than nurses on OA wards. The frequency of restraint was the same between the two types of wards. Provision of ward based activity and therapy were significantly better for patients on OA than WAA wards.	Poor

Chen, Hwu & Wang (2009) Taiwan	Questionnaire/ Quantitative	A psychiatric hospital	Staff (n=222). 53% female. Modal age group 30-44.	To explore staff responses to workplace violence in a psychiatric hospital	Staff experiencing verbal abuse were most likely to take no action (27%). Staff experiencing sexual harassment were more likely to report the incident to senior staff than staff experiencing other types of violence (76%). The most common form of management following an incident of physical violence or racial harassment was medication injection (29% and 30%); and for incidents of verbal abuse, bullying/mobbing and sexual harassment it was issuing a verbal warning (30%, 29% and 29%). The most common reason for not reporting violent incidents was that it was considered unimportant.	Fair
Chen, et al. (2007) Taiwan	In-depth interviews/ Qualitative	A psychiatric hospital	Staff victims of assault (n=13). Age range 23-60.	To explore the effects of assault on staff caring for schizophrenic patients in a psychiatric hospital	All staff reported that they did not receive enough post-incident support. The most common time for violence to occur was during ward inspections when staff were alone. The most severe psychological harm to staff was post-traumatic stress disorder. Staff gave suggestions for preventing further attacks: pre-placement training, good practice (asking for consent, not being alone, taking precautions and respecting patients' rights), and addressing organisational culture.	Fair

Cole, Baldwin & Thomas (2003) UK	Questionnaire/ Quantitative	Acute inpatient wards (n=3), a rehabilitation ward (n=1), and an intensive care ward (n=1) within a general adult psychiatric unit	Staff (n=109)	To explore staff practices and emotions concerning patient sexual assault in a psychiatric unit	The actions that most participants felt should be taken following a sexual assault were recording in notes (98.1% agreed) and informing teams involved (98.1% agreed). The emotional response to patient sexual assault that most participants reported feeling 'a lot/extremely' was being calm (38.3%). the majority of participants worried about being blamed in the instance of a sexual assault.	Fair
Cutcliffe (1999) UK	Semi-structured interviews/ Qualitative	An inpatient unit within a psychiatric hospital	Qualified nurses (n=6)	To explore the experiences of nurses who experience violence perpetrated by psychiatric patients.	3 key themes emerged: personal construct of violence, feeling equipped, and feeling supported. A relationship was found between exposure to violence and nurse's ability to manage these therapeutically. This is influenced by formal systems in place within the unit.	Fair
Daffern (2007) UK	Questionnaire/Qualitative	A high secure Dangerous and Severe Personality Disorder (DSPD) service within a secure hospital	Nursing staff and patients (n=18)	To explore nursing staff ability to assess the function of psychiatric patients' aggressive behaviour	The nursing staff's assessment of the functions of aggression differed from the patients' most significantly for the following: to enhance status or social approval, to obtain tangibles, and to observe suffering.	Fair
Daffern, et al. (2009) UK	Pre-post questionnaires/Quantitati ve	A high secure Dangerous and Severe Personality Disorder (DSPD) service within a secure hospital	Patients (n=51). Mean age 34 (range 18-54)	To evaluate whether structured risk assessment followed by results and management recommendations can reduce the frequency of aggression in DSPD patients	There was no significant difference in the frequency of reported aggression across the phases of the intervention. Staff reported that they did not feel the structured risk assessment was superior to clinical judgement and did not use it regularly in the management of aggression.	Fair

Daffern, Mayer & Martin (2006) Australia	Longitudinal/ Quantitative	Acute (n=2) and continuing care (n=1) wards within a secure forensic psychiatric hospital	Staff and patients	To explore the relationship between staff gender ratio and incidents of aggression in acute psychiatric wards	Verbal aggression was the most common form of aggression (n = 196, 62% of incidents). Victims were mostly staff (70%). Staff gender did not significantly affect the frequency or severity of aggressive incidents, or the likelihood of seclusion being used.	Fair
Daffern, Ogloff & Howells (2003) Australia	Longitudinal/ Quantitative	Acute (n=3) and continuing care (n=2) wards within a secure forensic psychiatric hospital	Staff and patients	To explore aggression and seclusion in secure psychiatric settings and to examine the reporting of aggression using aggression-specific recording instruments	Verbal aggression was the most common form of aggression (n = 205, 62% of incidents). Victims were mostly staff (n=229). 21% of aggressive incidents resulted in seclusion. There was no significant relationship between seclusion and severity of incidents, victim type (staff or patient), time of day or number of incidents in the preceding week.	Fair
Danivas, et al. (2016) India	Observational study/Quantitative	Acute psychiatric inpatient wards (n=2)	Inpatients (n=63)	To examine prevalence of aggression and coercion in a acute psychiatric ward.	There were 229 violent incidents reported; 55.9% identified as severe on SOAS-RI.Parents provoked 35% of the violent incidents and were the targets of the agression in 56% of the cases. Some incidents were managed using coercive measures (38%).	Good

Davies, et al. (2019) UK	Quantitative	Medium secure psychaitric hospital	Patients; Intervention group (n=22) and control group (n=17)	To evaluate the effectiveness of PBS in reducing frequency, management difficulty and severity of challenging behaviour	PBS significantly reduced challenging behaviour frequency and management frequency in the intervention group compared to the control group. Reductions were still evident six months after.	Fair
de Looff, et al. (2018) The Netherlands	Cross-sectional/Quantitative	Forensic psychiatric hospitals (n=4)	Forensic nursing staff (n=114). 59% female. Mean age 35 (SD=9.7)	To explore the relationship between type and severity of aggressive behaviour experienced by nursing staff and burnout symptoms	A positive association was found between physical aggression as experienced by staff and burnout symptoms (p=0.003)	Good
De Niet, Hutschemaekers & Lendemeijer (2005) Netherlands	Quasi-experimental time-series/ Mixed methods	A closed ward of a psychiatric institution	Nursing staff (n=23)	To explore reasons for the decline in the amount of reported aggression incidents when using the Staff Observation Aggression Scale (SOAS)	The number of reported aggression incidents decreased over the course of the intervention (t=2.598, df=55, P=0.012). Nursing staff did not perceive a reduction in the amount of aggression incidents, and stated that the SOAS did not add anything to the aggression management process, hence the reduction in reported incidents using this form.	Fair

Delaney, et al. (2001) Australia	Questionnaire, focus groups and chart review/ Mixed methods	Acute inpatient facilities (n=4)	Survey: nursing staff (n=59). 56% male. Modal age group 41-50. Focus groups: nursing staff	To explore the management of aggressive patients in psychiatric inpatient settings	88% of participants had been assaulted, with the majority (53%) stating that they had not attended aggression management training in the past year. Participants felt that history/background information of the patient, ongoing assessment, individualised care, peer support, policies/manual accessibility and stress management were key to effective aggression management.	Fair
De Young, Just & Harrison (2002) USA	Questionnaire/ Quantitative	A behaviour management unit within a long-term care facility	Patients (n=32). 53% male. Mean age 73 (range 38-95).	To evaluate a program designed to reduce the frequency of aggressive, agitated or disruptive (AAD) behaviours in a behaviour management unit	The most common AAD behaviours were resisting care and becoming upset/losing temper. There was a significant reduction in AAD incidents over the 6 month intervention period (t (df 18) =4.47, p=0.01). Staff reported using timing strategies to manage AAD behaviours most frequently.	Fair

Dickens, Piccirillo & Alderman (2013) UK	Questionnaire/ Quantitative	Low and medium secure wards of adult mental health care pathways of men's and women's services of a healthcare trust	Staff (n=72). Patients (n=98). 57% male. Mean age 34 (range 18-65)	To explore staff and patient attitudes concerning the management of violence and aggression in a secure, forensic mental health service	Patients and staff agreed on most internal causes of aggression, but differed on 2 points: patients agreed that it is difficult to prevent aggression and that being left alone can calm aggressive patients, whereas staff disagreed. Staff were more likely than patients to agree that external factors were causative of aggression. Patients and staff agreed on most situational/interactional causes of aggression, but patients felt that aggression could happen when staff do not listen, whereas staff disagreed with this. Patients and staff agreed on most management techniques, but patients felt that seclusion/restraint was sometimes overused, whereas staff did not, and patients felt that medication should be used more, whereas staff did not.	Fair
Duxbury & Whittington (2005) UK	Questionnaire/ Mixed methods	Inpatient mental health wards (n=3)	Nurses (n=80; 76% female) and patients (n=82; 51% female)	To explore the views of staff and patients concerning the causes and management of aggression in inpatient mental health wards	Both patients and staff agreed that external factors (restrictive environments) contributed to aggression. Staff were more likely to agree that a patient's illness contributed to aggression. Patients were more likely to agree that interactions/situations contributed to aggression. Staff were more likely to endorse management techniques such as medication and seclusion, although both	Fair

					groups agreed that restraint was an inevitable part of keeping the wards safe.	
Evans & Petter (2012) UK	Questionnaires/ Mixed methods	A PICU	Staff (n=15). 60% male. Age range 30-50.	To explore staff perceptions of violence and how safe they feel on a PICU	Overall staff reported feeling safe and supported at work. Themes arising from the interviews highlighted personal and systemic factors that relate to feeling safe and supported. The most commonly mentioned personal factors were: aggression as illness related, and nurses remaining emotionally unaffected. The most commonly mentioned systemic factors were: fear of letting the team down, lack of senior management support (fear of blame), reliance on external staff as increasing risk, and first admissions being higher risk.	Fair
Foley, et al. (2003) USA	Structured interview/ Mixed methods	Special care dementia units (n=53) within nursing facilities (n=49)	Staff (n=32)	To identify what differentiates successful from unsuccessful behaviour management of dementia patients in SCUs	Factors were found to be associated with successful management of behaviour problems: behaviour prevalence/severity, management techniques, use of psychotropic medication/restraint, comorbidity, and family interaction.	Fair

Gallop, et al. (1999) Canada	Semi-structured interviews/Qualitative	Acute care psychiatric units within hospitals (n=3)	Patients (n=20). 100% female.	To explore safety concerns amongst abused women in inpatient psychiatric environments	The most prevalent concerns raised were: mixed-gender units (preferring separate areas), night-time routines (restricting medications and staff contact at night), and primary nursing (as important for feeling safe). Participants expressed a desire to be involved in decision making.	Fair
Giles, et al. (2005). USA	Descriptive/Quantitative	Locked skilled nursing facility	Psychiatric inpatients (n=40); 27% female; mean age 46 (range 21-71)	To examine the effectiveness of a programme to reduce aggression in inpatients	The programme resulted in a small reduction in aggressive incidents.	Fair
Hallett & Dickens (2015) UK	Cross-sectional/Mixed methods	Secure inpatient mental health unit	Clinical staff (n=72); 50% female	To explore perspectives of clinical staff about de-escalation including interventions used in de-escalation, what staff believe constitutes de-escalation and which interventions are effective	Half of the staff suggested medication was used as an intervention for de-escalation. 15% incorrectly stated that interventions such as seclusion, restraint and emergency intramuscular medication could be used as a de-escalation technique. Different aggression types were identified as needing different interventions and a personalised approach.	Fair
Higueras, et al. (2006) Spain	Experimental/ Quantitative	Inpatient psychiatric ward of a general hospital	Psychiatric inpatients; baseline (n=83); mean age 41; 40% female	To examine the effects of a humour-based activity on disruptive behaviours	Disruptive behaviour significantly reduced after the intervention compared to baseline. Specifically, attempted escape, self-harm and fighting significantly reduced.	Fair
Hvidhjelm, et al. (2016) Denmark	Quasi-experimental design with a pre- and post-test/ Quantitative	Psychiatric wards (n=15) assigned to intervention (n=7) and control (n=8) group	Patients in control wards (n=595). 51.1% male. Mean age 45.1 + 16.7.<br><br>Patients in intervention (n=519) wards. 53% male. Mean age 46.4 +	To evaluate the effect of routine use of the Brøset Violence Checklist (BVC) throughout the entire admission on the risk of patient aggression as assessed at the ward	The analysis was conducted at the ward level because each ward was allocated to the intervention and control groups. At baseline, the risk of aggression varied between wards, from one	Good

			18.8.	level	aggressive incident per patient per 1,000 shifts to 147 aggressive incidents per patient per 1,000 shifts. The regression discontinuity analysis found a 45% reduction in the risk of aggression (Odds Ratio (OR) = 0.55, 95% confidence interval: 0.21–1.43). The study did not find a significant reduction in the risk of aggression after implementing a systematic short-term risk assessment with the BVC.	
Hylén, et al. (2019) Sweden	Focus groups and interviews/ Qualititative	Psychiatric inpatient care wards (n=3): general inpatient care, forensic care and addictive care.	Nurses and assistant nurses (n=17) and their managers (n=3)	To describe the nursing staff and ward managers' experiences of safety and violence in (1) the everyday meeting with the patient and (2) meetings that are violent or present a risk of violence	The qualitative content analyses resulted in four themes: the relationship with the patient is the basis of care; the organizational culture affects the care given; knowledge and competence are important for safe care; and the importance of balancing influence and coercion in care. The staff had a varied ability to meet patients in a respectful way.	Fair
Ilkiw-Lavalle & Grenyer (2003) Australia	Mixed-methods design/ Mixed-methods	Psychiatric inpatient unit (n=4)	Staff (n=29); 48% female; mean age 33 and 29 inpatients (n=29); 35% female; mean age 31	To examine views of staff and patients involved in incidents of aggression to understand emotions experienced, perceptions of causes and recommendations for reducing aggression	Staff and patient views differed on the cause of the aggression: staff felt it was the patient's mental illness and patient's felt illness, interpersonal and environmental factors were equally to blame for the aggression. Overall participants were satisfied with the management of	Fair

					aggressive incidents.	
Ireland, Halpin & Sullivan (2014) UK	Semi-structured interviews/ Qualitative	Forensic psychiatric inpatient hospital	Male inpatients (n=16); mean age 31	To examine motivations for forensic patients' engagement in critical incidents	The main themes included engaging in critical incidents to seek deliberate isolation, to gain control, to get needs met, a need to communicate and because they were influenced by peers.	Fair
Ireland, et al (2019) UK	Exploratory study/quantitative	High secure psychiatric hospital	Study 1 - patients (n=44); study 2 - patients (n=53) and staff (n=167); study 3 - (n=414)	To investigate the impact of making changes to factors relevant to engaging and experiencing intra-group aggression (bullying) in a secure hospital	Indirect changes to the environment lead to more positive effects, in relation to bullying, than direct changes.	Fair
Isaak, et al. (2016) Israel	Questionnaires pre-post evaluation/Quantitative	A maximum security unit at a mental health centre	All staff (pre-intervention n=112; post-intervention n=85). 53% female	To evaluate the effectiveness of an intervention to improve safety climate in a secure psychiatric setting	Six months after the intervention, significant improvements were found in perceptions of management's commitment to safety (p=0.02) and in communication about safety issues (p=0.056)	Fair
Jacob, et al. (2013) India	Observational/ Quantitative	Child and adolescent inpatient service	Children inpatients (n=31); 16% female; Mean age 12.9	To identify characteristics of aggressive incidents and how they are managed	Almost a quarter of all inpatients (n=131) demonstrated aggression (23.7%). Aggression was most likely to occur at night and against family members	Fair

Janicki (2009) UK	Semi-structured interviews and case note review/ Mixed methods	Women's medium secure hospital	Participants (n=15); patients (n=6), nurses (n=3); and other professionals (n=6)	To explore staff and patient perspectives on the involvement of the criminal justice system as a response to an assault on the medium secure ward	Overall, participants felt the involvement of the CJS was essential to ensure high morale and that incidents were dealt with so that this deterred other patients from committing assault	Fair
Jeffs, et al. (2012) Canada	Explorative/ Qualitative	Inpatient mental health care agencies (n=3)	Staff: Site A (n=24), site B (n=19), Site C (n=24); patients: site A (n=9), site B (n=9), site C (n=10)	To gain insight into how service providers and service users experience and define near misses.	Overall near misses were identified as safety threats and issues associated with patients' mental illness and also situations that avoid harm to others and prevent an incident	Good
Kelly, et al. (2017) USA	Cross-sectional/Quantitative	Psychiatric hospital	Staff (n=323)	To examine staff wellbeing and safety procedures in relation to aggression	The majority of staff had experienced verbal aggression (98%) and over two thirds of staff had experienced physical assault in the hospital (69.5%). Consequences of the assaults included staff not feeling staff (44.6%) and wanting to be more protected at work (90.1%). Staff wellbeing and safety concerns were adversely affected by conflicts eith other staff members. Staff-patient relationships , stress reactivity and violence are linked.	Fair
Kelly, et al. (2017) USA	Cross-sectional/Quantitative	Psychiatric hospital	Staff, female (n=348).	To examine social support in psychiatric inpatient staff following an assault	Over-two thirds experienced physical assault. 54% of staff reported feeling very unsafe or unsafe at work. 28% of staff who had experienced an assault did not receive post-assault support.	Good

Killick & Allen (2005) UK	Experimental/ Quantitative	Psychiatric inpatient unit	Experimental staff group (n=27); 66.7% female; 40.9 mean months' work experience [89.2]	To assess the effects of three training courses on managing aggression, staff knowledge and satisfaction before, during and after training and at one year follow-up	Staff knowledge and confidence in managing aggression increased following training but was not maintained at 1 year follow-up	Poor
Koukia, et al. (2013) Greece	Cohort design/Qualitative	Psychiatric hospital (n=3)	Nurses (n=26); 62% female; mean age 36 years [7.7] (range 23-54)	To identify nurses' interventions, views, and attitudes concerning critical incidents.	Nurses reported six interventions included counselling, performing security practices, monitoring thought disturbances, contacting the psychiatrist on-call, contacting the chief nurse on-call and administrating medication.	Fair
Lamanna, et al. (2016). Canada.	Interviews/ Qualititative	Psychiatric department of a general hospital.	"Inpatients (n=14). 64.3% female. Mean age 49.1+18.9 (range 18-77)	Lamanna, et al. (2016). Canada.	Interviews/ Qualititative	Psychiatric department of a general hospital.
Lantta, et al. (2015) Finland	Intervention design, literature review, case-note review and focus groups/ Mixed methods	Mental health inpatient units (n=3)	Nurses (n=22)	To explain the intervention of implementing a structured violence risk assessment procedure in mental health inpatient units using the Ottawa Model of Research Use (OMRU) as a guiding framework. To also consider nurses' perspectives of its clinical utility and implementation process	Some staff felt the model was useful but it was less preferred than nurse's own clinical judgement in some instances	Poor

Lantta, et al. (2016). Finland.	Focus groups/ Qualititative	Inpatient psychiatric wards (n=3): acute admissions ward, acute forensics ward and treatment and rehabilitation ward	Nurses (n=22). 73% female.	To explore nurses' experiences of violent events in psychiatric wards, give insight into ward climates and examine suggestions for violence prevention.	Nurses' experiences of violent events included a variety of warning signs and high-risk situations which helped them to predict forthcoming violence. Patient-instigated violent events were described as complicated situations involving both nurses and patients. When the wards were overloaded with work or emotions, or if nurses had become cynical from dealing with such events, well-being of nurses was impaired and nursing care was complicated. Suggestions for violence prevention were identified, and included, for example, more skilled interaction between nurses and patients and an increase in contact between nurses and patients on the ward.	Fair
Lanza, et al. (2016) USA	Experimental/ Quantitative	Locked psychiatric units (n=7)	Violence Prevention Community Meeting group (n=4) and control group (n=3).	To assess the violence prevention community meeting (VPCM) as an effective intervention to reduce workplace violence in acute care psychiatric units	Per week aggression rates reduced slightly in the VPCM hospitals (0.6% (95% CI: -5.6%, 6.5%; non-significant)), but reduced significantly more in the control hospitals (5.1% (95% CI: 0.4%, 9.6%; significant)).	Poor
Lanza, et al. (2009) USA	Pre-post experimental design/Quantitative	Acute psychiatric inpatient unit at a Veterans Affairs hospital	Male patients. Mean age 42.6. Staff (n=21)	To test the efficacy of the VPCM for reducing patient violence	There was a decrease of physical violence of 89% from pre-treatment to treatment and a decrease of 57% from pre- to post-treatment.	Fair

Lawn & Pols (2003) Australia	Mixed-methods	Locked psychiatric ward	Inpatients (n=24). Staff (n=26)	To explore experiences regarding smoking and violence in locked inpatient settings	There was conflict between staff and patients over staff controlling the supply of cigarettes. This was made worse by the physical structure of the smoking area.	Poor
Lehmann, McCormick & Kizer (1999) USA	Descriptive/ Quantitative	Veterans medical centres and free-standing clinics (including inpatient psychiatric units) (n=166)	N/A	To examine the scope and impact of violence in hospital settings	During October 1990 to 1991 there were 6,592 incidents of physical assault in inpatient psychiatric units. The incident rate per 100,000 patient days was 177.9, equating to the highest proportion across medical units. A quarter of staff recommended more training (24.1%)	Poor
Lipscomb, et al. (2012) USA	Questionnaire/ Mixed methods	Residential addiction treatment centres (ATCs) (n=13)	Staff (n=409); 59% female	To examine the relationship between violence prevention safety climate measures and self-reported violence toward staff.	Predictors of violence included management commitment to violence prevention as “never/hardly ever” (OR=4.30), client actively resisting program (OR=2.34) and working with clients with unknown history of violence (OR=1.91)	Fair
Lowe, Wellman & Taylor (2003) UK	Vignette and questionnaire/ Quantitative	Acute psychiatric inpatient unit	Nurses (n=70)	To examine nurses response to scenarios of conflict incidents	Autonomy and limit-setting were acknowledged as the most notable responses to incidents of conflict. Across nurses these were most likely to cause disagreement.	Fair
McCann, Baird & Muir-Cochrane (2014) Australia	Questionnaire design/ Quantitative	Locked old age psychiatric inpatient units (n=3)	Staff (n=85). Registered nurses (61.1%, n = 52), enrolled nurses (27.1%, n = 23) and medical and allied health staff (11.8%, n = 10); 66% female; Mean age 43 (range 24-62 years)	To examine the attitudes of clinical staff toward the causes and management of aggression	Causes of aggression were multifactorial. Staff felt patient factors did not contribute to aggression. They felt other patients and staff contributed to aggression and specific cultural groups were more likely to be aggressive. Participants differed in their view of prevention strategies for aggression. Medication	Fair

					was deemed the more viable option but staff were undecided on the appropriateness of seclusion.	
McLaughlin, et al. (2010) UK	Pre-post intervention study/ Quantitative	Acute psychiatric inpatient ward	Nurses (n=18); median age 33 (range 23-52)	To examine the potential effectiveness of an intervention for managing verbal aggression	Post intervention focus group analysis revealed there was an increase in staff diffusing the situation to manage verbal aggression	Poor
Meaden, Hacker & Spencer (2013) UK	Interview/ Quantitative	High dependency inpatient units	Nurses (n=25); 48% female	To assess the ability of an adapted early warning signs type of dynamic risk assessment to more accurately predict aggressive incidents	Receiver Operating Characteristic (ROC) analyses revealed that the assessment had moderate predictive validity regarding aggression (AUC=0.5) but effect size was small.	Fair
Meehan, McIntosh & Bergen (2006) Australia	Focus groups/ Qualitative	High Secure Forensic Unit (HSFU)	Patients (n=27); 23% female	To explore the patient perspective on aggressive behaviour	There were five main themes that explored the cause of aggression: the environment, empty days, staff interactions, medication issues and patient centred factors. Potential management to reduce aggression was identified as improved training, separation of acutely disturbed patients, early intervention, improving staff attitudes and implementation of effective justice procedures.	Fair

Murphy, & Siv (2007) USA	Pre-post questionnaire/ Quantitative	Child and adolescent residential treatment unit	Patients (n=20). Average age in treatment as usual (TAU) group 14.8 and in Mode Deactivation group 15.7	To replicate previous findings supporting the effectiveness of Mode Deactivation Therapy (MDT).	The results showed MDT to be more effective then TAU in reducing both physical aggression and therapeutic restraints. MDT showed a reduction of 66.8% in physical aggression compared to TAU (27.9%). MDT showed a reduction of 70.7% in therapeutic holds compared to TAU (24.7%).	Fair
Needham, et al. (2005) Switzerland	Randomised controlled trial/ Quantitative	Acute psychiatric inpatient wards (n=6)	Nurses in control group (n=28); 50% female; mean age 39.1. Nurses in treatment group (n = 30); 60% female.	To investigate the effects of a training course in the management of aggressive behaviour in psychiatric acute inpatient settings.	No statistically significant differences of the means were found between the intervention and control groups on the positive perception of aggression (p=0.912), the negative perception of aggression (p=0.315), or the tolerance scale (p= 0.614).	Fair
Needham, et al. (2004) Switzerland	Feasibility study/ Quantitative	Acute mental health care inpatient settings (n=2)	Nurses. Patients (n=576); 41% females, mean age 38, age range 15–88	To implement a risk-prediction procedure and a standardized aggression management intervention.	There was a significant reduction in the percentage of days with attacks against persons (trend-test P = 0.04) and percentage of days with usage of coercive measures against any patient (trend-test P =0.01).	Good
Nijman, et al. (2005) UK	Cross-sectional/ Quantitative	Acute wards (n=12), psychiatric intensive care units (n=2), long-term rehabilitation wards (n=2), wards for the elderly mentally ill (n=3), and forensic wards at a medium security level (n=5)	Nurses (n=154); 58% female; 70% were under 40, and 36% were younger than 30	To explore psychiatric nurses' experiences with aggression	33 of 148 respondents (22%) said that they had not been able to go to work due to workplace violence at least once during the year. They had stayed at home for a total of 172 days, with an average of 5.2 sick days per nurse (range 1–23 days). Severe physical violence was the strongest predictor of sick leave (r=0.50). Frequent sexual harassment and intimidation also increased the likelihood	Fair

					of sick leave (r=0.38).	
Niu, et al. (2019) Taiwan	Cross-sectional/Quantitative	Acute psychiatric hospital	Nurses (n=429)	To examine prevalence of violence in acute psychiatric settings in staff.	The majority of staff had experienced workplace violence (88.3%); just over half (55.7%) had experienced physical violence.	Good
Nolan, et al. (2009) USA	Questionnaire/ Quantitative	Specialised research unit	Patients	To describe the reasons for aggressive behaviour reported by patients and staff and the relationships between those reasons and the subsequent interventions delivered in response to aggression.	Patients reported more often than staff that aggression was caused by external factors. Staff cited internal factors more often than patients. Responses to aggression were not related to the cause of the behaviour.	Fair
Olsson, et al. (2015) Sweden	Semi-structured interviews/ Qualitative	A maximum security forensic psychiatric hospital	Patients (n=12); 83% male; mean age 37.	To examine the experiences of forensic inpatients that have decreased their risk of becoming violent in forensic care.	3 themes emerged: 1) Staff's attitudes and actions. Patients' perception of staff´s ability to manage conflicts. 2) Patients' own insight and actions: Being insightful and managing the situation Dealing with aggression Attending to signs of warnings. 3) Interactions in the health care environment: Experiences of the physical environment- participants found overcrowded wards to be stressful and anxiety-provoking.	Fair

Olsson & Schon (2016) Sweden	Interviews/Qualitative	A maximum security forensic psychiatric hospital	Staff (n=13). 54% male. Mean age 30	To explore forensic psychiatric staffs' methods of violence prevention and compare these to recovery-oriented care	Three themes arose: internal knowledge; peer security; and control-oriented strategies	Fair
Park & Lee (2012) South Korea	Non-random experiment/ Quantitative	A psychiatric hospital	Inpatients in experimental group (n=22) and control group (n=22). 100% male (age range 30-60)	To measure the effect of behaviour modification using Short-Term Token Economy (STTE) on aggressive behaviour.	In the experimental group, aggressive behaviour decreased by 20.8% compared with the comparison group after STTE. The number of verbal attacks decreased by 27.6%. Property damage or physical attacks decreased by 14.3%.	Fair
Paschali, Lange-Asschhenfeldt and Kamp (2017) Germany	Cross-sectional/Quantitative	Secure psychiatric hospital	Inpatients (n=66).	To identify risk factors that lead to aggression using the SOAS-R scale	Over half of patients who had been involved in an aggressive incident had dementia (57%). 80% had a precipitating event. Majority of incidents were directed towards nurses.	Fair

Pekurinen, et al (2017) Finland	Cross-sectional/Quantitative	Psychiatric unit (n=84)	Staff (n=1033)	To examine the association between workplace factors and the likelihood of aggression	Workplace strain was significantly associated with aggression (OR 1.65, p = 0.02). Poor and average staff satisfaction (OR 1.83, p = 0.04, OR 1.93, p = 0.02, respectively) were also significantly related to increased levels of aggression.	Good
Phillips (2011) UK	Focus groups/ Qualitative	Inpatient wards	Mental health staff	To discuss issues raised during workshops aimed at encouraging and enabling staff to work in a gender-sensitive way to develop strategies for decreasing sexual assault incidence for female patients who may have histories of being abuse victims.	Staff taking part in the workshops expressed the importance of maintaining boundaries, developing adequate policies, and one-to-one observation. Issues regarding the truth of allegations and roles of the female patients in sexual assault incidents was mentioned as an issue. It was agreed that adequate staffing levels are essential for effective patient care.	Fair
Podubinski, et al. (2017) Australia	Cohort/Quantitative	Acute psychiatric units (n=2)	Inpatients (n=200). 66% female.	To examine different factors on aggression during psychiatriic hospitalisation	Just over a third of patients were deemed to be aggressive (35%). Interpersonal factors including hostile-domniance interpersonal style (OR 1.29 95% 1.17-1.43), psychopathy (OR 1.35 95% 1.16-1.58), aggressive script rehearsal (OR 1.21 95% 1.06-1.38), attitudes towards violence (OR 1.15 1.04-1.27), trait anger (OR 1.07 1.02-1.12) and disorganised (OR 1.09 95% 1.03-1.16) and excited symptoms	Good

					(PANSS) (OR 1.18 95% 1.04-1.34) all significantly predicted aggression.	
Price, et al. (2018) UK	Interviews/ Qualititative	In-patient wards (n=5) including male psychiatric intensive care units (n=3), female acute ward (n=1) and male acute ward (n=1)	Clinical staff (n=20). 50% female. Age range 18-60.	To obtain staff descriptions of de-escalation techniques currently used in mental health settings and explore factors perceived to influence their implementation and effectiveness.	Participants described 14 techniques used in response to escalated aggression applied on a continuum between support and control. Techniques along the support-control continuum could be classified in three groups: ‘support’ (e.g. problem-solving, distraction, reassurance) ‘non-physical control’ (e.g. reprimands, deterrents, instruction) and ‘physical control’ (e.g. physical restraint and seclusion). Charting the reasoning staff provided for technique selection against the described behavioural outcome enabled a preliminary understanding of staff, patient and environmental influences on de-escalation success or failure. Importantly, the more coercive ‘non-physical control’ techniques are currently conceptualised by staff as a feature of de-escalation techniques, yet, there was evidence of a link between these and increased aggression/use of restrictive practices.	Fair

Quirk, Lelliott & Seale (2005) UK	Longitudinal/ Qualitative	Inpatient wards (n=3) in general hospitals (n=2) and a psychiatric hospital (n=1)	Observations made by a research sociologist.	To explore how patients manage risks arising from their interaction with other patients on the ward, such as assault and sexual harassment.	Patients seemed to manage interaction risks by: avoiding risky situations or people; de-escalating situations; seeking safety interventions by staff or increased surveillance; and seeking protection from other patients.	Fair
Raveendranathan, Chandra, & Chaturvedi (2012) India	Semi-structured interviews/ Quantitative	Adult psychiatric wards	Family members of patients (n=75); 51% male; mean age 29	To assess inpatient violence from victims' perspectives, in settings where family members accompanied patients during inpatient stay and played a significant role in caregiving.	Family members were the targets of violence in 70% of the 100 incidents studied. 81% of these episodes were provoked. Provocation factors included the patient being prevented from leaving the ward and strict rules enforced by the staff. Family members suggested several preventative measures: more staff, sedation, and improved communication.	Fair
Reininghaus, et al. (2007) UK	Questionnaire/ Quantitative	A high secure psychiatric hospital	Nurses (n=636); 68% male. Mean age 39	To investigate different stress resistance resources (SRR) in the stress processes of staff at a psychiatric hospital when physical assault occurs.	Physical assault was found to be significantly related to psychological distress (P = 0.004, B = 0.181, 95% CI 0.060–0.303, R2 = 0.014). Self-esteem, self-confidence and coping were found to be effective SRRs for mediating levels of psychological distress following a physical assault.	Fair

Ryan, et al. (2004) US	Prospective/Quantitative	An inpatient psychiatric hospital	Patients (n=111); 56% male; mean age 13.9 (SD= 2.8)	This study examined the frequency and nature of violence directed at staff in a state inpatient psychiatric hospital.	The highest reported reason (68%) for assaults of staff by patients was related to verbal exchanges with staff: 'request, direction, or command potentially viewed by the patient as limit setting or as coercive'	Fair
Sato, et al. (2017) Japan	Cross-sectional / Quantitative	Emergency psychiatric (EP) wards (n=8), acute psychiatric (AP) wards (n=6), and standard (S) wards (n=16) across psychiatric hospitals (n=20)	Patients:<br>EP wards: (n=111). 64.9% male. Mean age 42.4+21.3<br>AP wards: (n=131). 58.8% male. Mean age 50.3+17.6<br>S wards: (n=201). 60.7% male. Mean age 51.4+13.7<br>Psychiatric nurses.	To examine the characteristics of aggressive incidents by ward type	Findings suggest that ward environment was an important factor influencing aggressive behaviour. In acute and emergency psychiatric wards, staff members were the most common target of aggression. In acute psychiatric wards, staff requiring patients to take medication was the most common provocation, and verbal aggression was the most commonly used means. In emergency psychiatric wards, victims felt threatened. In contrast, in standard wards, both the target and provocation of aggression were most commonly other patients, hands were used, victims reported experiencing physical pain, and seclusion was applied to stop their behaviour.	Good
Schwartz & Park (1999) US	Cross-sectional/ Quantitative	A psychiatric facility	Residents (n=517) 52% male; age range 25-61.	To investigate the prevalence of assaults on psychiatric residents and the violence management training they receive.	73% of residents reported being threatened, and 36% reported being physically assaulted. A third received no violence management training, and a third felt that their training was inadequate.	Fair

Selenius & Strand (2017) Sweden	Interviews/Qualitative	A high secure forensic hospital	Forensic psychiatric inpatients (n=13). 100% female	To explore how female psychiatric patients describe their aggression and self-harm behaviours	Three themes arose: from triggers to negative thoughts and emotions; strategies for handling negative thoughts and emotions; and satisfaction	Good
Sival, et al. (2000) Netherlands	Prospective cohort/ Quantitative	Wards of a psychogeriatric nursing home (n=2)	Residents (n=64); 72% female; mean age 80.2 (SD=7.8)	To investigate the effects of introducing a behaviour rating scale on the prevalence and management of aggressive behaviours in psychogeriatric patients.	The frequency of aggressive behaviour reported increased significantly (p<.001), while prescribing of psychotropic drugs decreased significantly (p<.05).	Fair
Sjöström, et al. (2001) Sweden	Quasi-experimental/ Quantitative	Inpatient psychiatric care	Nursing staff members (n=185) before training (n=144) after training.	To investigate whether aggressive behaviour and injury-related sick leave would be reduced by staff training and to explore predictors of violent behaviour.	No statistically significant reduction was found in the number of aggressive patients or in the number of staff members on sick leave. Directed verbal aggressiveness (OR = 1.92, P = 0.04) and violence towards things (OR = 1.82, P = 0.02). were found to be predictors of violence.	Fair
Skovdahl, Kihlgren & Kihlgren (2003) Sweden	Cross-sectional/ Quantitative	Units housing residents with dementia (n=2)	Unit 1: Caregivers (n=3); 100% female; mean age 38.5, age range 36-42; female resident (n=1); aged 92. Unit 2: Caregivers (n=6); 67% female; mean age 29.5, age range 22-43; female resident (n=1); aged 85.	To investigate, using video recordings, interactions between those with dementia and aggressive behaviour, and caregivers reporting experiencing problems dealing with this behaviour.	Interactions followed either a positive or negative spiral pattern. Caregivers who had reported problems dealing with behavioural symptoms focused on finishing the task (goals). Caregivers who were satisfied with their management of aggressiveness, focused on the processes of how goals could be achieved.	Fair

Speziale, et al. (2009) Canada	Pre-post intervention/ Quantitative	An inpatient facility for older adults with serious mental illness.	Staff who received the geriatric programme curriculum (n=99)	To examine the impact of training in Gentle Persuasive Approaches (GPA) on staff knowledge and competency regarding challenging patient behaviours (verbal and physical) and also on patient risk events and occupational health incidents.	Surveys found that GPA training significantly improved staff's response to challenging behaviours, and their learning of management strategies. The predicted use of body containment techniques decreased after training. Physical aggression rates declined by 50% 3 months after training (χ^2 (2, N = 564) = 27.51, p = .0001).	Fair
Spokes, et al. (2002) UK	Cross-sectional/ Qualitative	Psychiatric inpatient units (n=13); comprising adult acute admissions wards (n=10), psychiatric intensive care units (n=2) and a low-secure unit on five hospital sites	Qualified mental health nurses (n=68) and unqualified care assistants (n=40)	To investigate the views of mental health nurses about staff behaviours and other factors which may impact upon inpatient violence.	Three themes were identified by participants as being involved in the occurrence of violent incidents: clinical skills (experience, knowledge of techniques, job grade, etc.), personal characteristics (self-confidence, calmness, control, etc.) and interpersonal skills (rapport with patients, explaining things, etc.) Respondents also emphasized training needs, both in terms of new knowledge and of means of coping with actual physical violence.	Fair
Stevenson, et al. (2015) Canada	Cross-sectional/ Qualitative	Acute inpatient psychiatry settings	Registered nurses (n=12); 67% female; median age 37.5, range 27-57.	To explore nurses exposure to and experiences of patient violence, as well as the strategies they describe as influencing current practices of patient violence	For many, patient violence was considered "part of the job." Nurses often struggled with role conflict between one's duty to care and one's duty to self when providing care following a critical incident involving violence. Issues of power, control and stigma also influenced nurse participant perceptions and their responses to patient violence. The majority had difficulty identifying	Good

					any strategies that they perceived would have been successful to prevent the violence in their workplaces.	
Stone, et al. (2011) Australia	Cross-sectional/Mixed methods	A mental health inpatient setting	Nurses (n=107)	To determine the nature of interventions nurses used in response to aggression and whether interventions used varied with perceived causes of aggression. To identify the relationship between swearing and verbal aggression, and the impact of both on nurses.	The most frequently reported intervention for incidents of verbal aggression were talking to the patient (70% of incidents). The majority reported that exposure to swearing was highly distressing. Females appeared to be very distressed irrespective of the situation, whereas males reported being significantly more distressed by relatives of patients swearing at them.	Fair
Sukhodolsky, Cardona & Martin (2005) USA	Cross-sectional/ Quantitative	An inpatient psychiatric unit	Child inpatients (n=86); 67% male; mean age 10.8 (SD=2.4);	To evaluate the contribution of aggressive and non-compliant behaviours to restraint and seclusion use, length of stay, and psychotropic medication use in a psychiatric inpatient population.	Severity of aggressive behaviour was significantly associated with the use of seclusion and restraint, but not with the length of hospitalization or the number of psychiatric medications at discharge. By contrast, the levels of noncompliant behaviour were associated with the length of hospitalization and the number of psychiatric medications, but not with the use of seclusion and restraint.	Good
Sutton, et al. (2013) New Zealand	Prospective/Qualitative	Inpatient mental health units (n=4)	Clinical staff: (n= 40) 90% female. Service users (n = 20) 90% female	To investigate whether sensory-based approaches can develop the knowledge and practice of managing aggression in mental health settings.	Three main themes were found that described elements of sensory modulation that were perceived as helpful in the management of aggression: (i) facilitating a calm state; (ii) enhancing interpersonal connection; and (iii) supporting self-management.	Fair

Tema, Poggenpoel & Myburgh (2011) South Africa	Cross-sectional/ Qualitative	A forensic ward	Psychiatric nurses (n=9); 78% female; age range 26-58	To explore psychiatric nurses' experiences of patients' aggressive behaviour in a forensic ward and to develop recommendations for managers to equip nurses with the skills and knowledge necessary to effectively manage patient aggression.	Four themes emerged: challenging therapeutic relationships with patients; experiences of fear resulting from threats of aggression; experiences of disempowerment related to a lack of recognition; and experiences of emotional and physical distress related to interactions with patients. Recommendations for management were to provide nurses access to: information, support, resources, opportunity and growth.	Fair
Terkelsen & Larsen (2016) Norway	Observational/Interviews/ Qualitative	A locked psychiatric ward	Patients (n=12; 75% male; age range 17-53) and staff (n=22; 64% male; age range 20s-60s)	To explore what happens in a dangerous situation on a locked psychiatric ward and how staff and patients interpret them	Three themes arose: atmosphere and material surroundings; stereotypes; and triggers	Good
Tomagova, et al (2016) Slovakia	Cross-sectional/Quantitative	Psychiatric inpatient wards	Nurses (n=223)	To examine differences between staff educational training and patient aggression.	The majority of staff had experienced patient aggression (98.6%). Verbal aggression had the highest incidence (3.27 ± 1.04; p<0.077) followed by physical aggression without a weapon (2.61 ± 2.22). Staff had a negative attitude towards patient aggression (offensive, destructive and intrusive) dermined the formation of aggression management. Nurses expressed strongest aggreement in using restrictive tools and medication to manage patient aggression.	Good

Trenoweth, (2003) UK.	Cross-sectional/ Qualitative	A secure mental health environment	Mental health nurses (n=10)	To explore how mental health nurses make risk assessments in situations where violence is perceived to be likely.	It was found that nurses use information regarding their knowledge of the patient, observations of behaviour, assessing the situation as a whole, and then team working to intervene, when assessing risk in potentially violent situations.	Fair
Umut, et al. (2012) Turkey	Cross-sectional/ Quantitative	A research and training hospital	Inpatients (n=104); 31% female; age range 20-65	To investigate the relationship between violence and insight, clinical symptoms and treatment adherence in schizophrenia patients.	Treatment adherent patients scored significantly higher on the insight measure ($Z=2.793$; $p<0.01$). Non-treatment adherent patients scored significantly higher on the measure of aggression ($Z=2.992$; $p<0.01$).	Fair
van den Bogaard, et al. (2018) Netherlands	Quantitative	Specialised treatment centre with closed wards for mild to borderline intellectual functioning (n=3)	Staff (n=40)	To examine incidents of aggression, changes over time and and the circumstances of which the incidents happen.	There were 264 incidents of which 210 were deemed aggressive; mean incidents 8.9 per year. The most common incident type was verbal aggression (57.1%) followed by physical aggression (31.9%) and aggression against property (9.5%).	Fair
Van de Sande, et al. (2011) Netherlands	Cluster randomised controlled trial/ Quantitative	Acute psychiatric wards (n=2)	Experimental ward: baseline period (n=80); Mean age 38 (SD=13), 66% male. Intervention period (n=207); Mean age 38, 65% male. Control ward: baseline period (n=90); mean age 40 (SD=11) 60% male. Intervention period (n=251); Mean age 39, 55% male.	To investigate the effect of risk assessment on the prevalence of aggression and time in seclusion for acute psychiatric ward inpatients.	The experimental wards demonstrated a reduction in aggressive incidents (relative risk reduction, $P<0.001$), patients engaging in aggression (relative risk reduction, $P<0.05$), and time spent in seclusion ($P<0.05$) compared to control wards. No reductions were found in the number of seclusions or the number of patients exposed to seclusion.	Good

Van Wijk, Traut & Julie (2014) South Africa	Phenomenological, Cross-sectional/ Qualitative	Mental health facilities (n=2)	Inpatients (n=40); age range 21-55	To explore patients' perceptions of environmental and staff contributory factors for their aggression and violence; and to propose prevention and management strategies for this behaviour.	Two categories of contributory factors were found: environmental factors (such as living conditions and ward atmosphere), and the attitude and behaviour of staff.	Fair
Wright, et al. (2005) UK	Cross-sectional/ Quantitative	Acute inpatient settings	Nurses (n=771); 63% female; mean age 36.5 (SD=9.2)	To report and discuss the findings of a survey of nurse training and practice in the prevention and management of violence in acute psychiatric units	Most respondents reported a good balance of theory and practical skills training, although some aspects of theoretical training were not explored in enough depth. Reported confidence in the ability to use skills safely or effectively was fairly low. Training was generally considered to be safe and well run.	Good
Wright, et al. (2014) UK	Cross-sectional / Qualitative	High secure facilities (n=3)	Patients (n=8); 100% male. Staff (n=10); 70% male	To explore and compare staff and patient attitudes to the management of violence and aggression in a high security hospital	Seven themes emerged for both staff and patients: 'Environmental stimuli' where boredom was identified as a reason for violence and aggression occurring, 'Medication' in terms of aggression management, medication could be seen as both positive and negative, and 'Relationships' where therapeutic relationships were valued by both staff and patient and aggression was directly related to staff attitudes.	Poor

Wystanski (2000) Canada	Observational, Longitudinal/ Quantitative	A psychogeriatric inpatient ward in a psychiatric hospital.	Inpatients (n=29); 66% female; mean age 73.9 (SD=7.6);	To investigate the relationship between psychosocial stimulation and medication changes, and assaultive behaviour in a psychogeriatric ward.	Those with organic syndromes were more likely to become, and stay, aggressive. Psychosocial stimulation and changes in non-psychotropic medications influenced the duration of the aggressive behaviour.	Poor
Yang, et al. (2007) Taiwan	Pre-post experimental/ Quantitative	A nursing home specializing in care for patients with dementia.	Individuals living with dementia (n=20); 65% male; mean age 74.2 (SD=6.7), age range 65-86	To evaluate the effectiveness of acupressure in reducing agitation in those with dementia.	Significant differences on all outcome measures were found between control and experimental phases, indicating a positive treatment effect (Cohen–Mans-field Agitation Inventory, daily agitation records about physical attack, verbal and non-verbal attack and non-physical attack).	Fair
Yip, et al. (2013) UK	Quasi-experimental/ Quantitative	A high secure hospital	High risk patients (n=59); 100% male; age range 18-65	To evaluate a programme's effectiveness in reducing violent attitudes, anger, coping processes, social problem solving, disruptive behaviour and social functioning in forensic psychiatric patients.	Medium to large treatment effects were found for the treatment group in relation to: self-reported measures of violent attitudes, social problem-solving and coping. Staff also rated behaviour on the wards as being markedly improved post-treatment.	Good
Zuzelo, Curran & Zeserman (2012) USA	Cross-sectional/ Qualitative	An inpatient psychiatric unit	Professional nurses and behavioural health associations (N = 19); 47% male; age range >50 (5), 40-50 (3), 30-40 (6), <30 (5).	To explore nursing staff's responses to violent incidents perpetrated by patients against caregivers.	The themes that arose regarding management of violence were: sharing information about violence, therapeutic and non-therapeutic intervention, recognizing team influences, experiencing emotions following violence, and understanding the work environment.	Good

Zwijsen, et al. (2014) Netherlands	Cluster randomized controlled trial/Quantitative	Nursing care homes with special care units for people with dementia (n=17)	Residents with dementia (n=659); 70% female; mean age 84 (SD=7.3)	To evaluate the use of a care program in decreasing challenging behaviour and the prescription of psychoactive drugs without increasing the use of restraints.	Participants in the intervention condition compared with the control condition differed significantly in the presentation of challenging behaviours. Significant effects were found on the use of antipsychotics and antidepressants. No effect on use of restraints was observed.	Good

Coercive Interventions

Author, year, country	Study design	Setting	Participants	Aims/ objectives related to patient safety	Outcomes related to patient safety	Study quality
Abdel-Hussein & Mohamed (2018) Iraq	Questionnaires pre-post evaluation/Quantitative	A psychiatric teaching hospital	Nurses (n=25). 56% female. Modal age group 30-39	To evaluate the effectiveness of an educational programme aimed at increasing nurses' knowledge of restraint and seclusion in psychiatric wards	There was a significant increase in nurses' knowledge of restraint and seclusion across the course of the educational programme (p=<0.01)	Poor

Bak & Aggernæs (2012) Europe	Questionnaire/ Quantitative	Inpatient facilities in Denmark, Sweden, Norway, Finland, Iceland, Belgium, Netherlands, United Kingdom, Ireland, France and Italy.	Governmental health authorities (European Platform of Supervisory Organisations, EPSO) and the Psychiatric Section of the European Union of Medical Specialists (Union Européenne des médicins Spécialistes, UEMS).	To compare the use of coercive measures on psychiatric inpatients of different European countries	All countries allowed the use of forced medication in some form in 2009. The UK was the only country where mechanical restraint was not allowed and Denmark was the only country where seclusion was not allowed. Coercion was perceived differently across countries. Forced medication/long period was considered worst in Norway; forced medication/short period in Belgium; mechanical restraint in Finland, Iceland and France; seclusion in the UK; holding/physical restraint in Netherlands and mechanical restraint/ambulatory in Sweden, Denmark and Iceland. In the countries using both seclusion and mechanical restraint, mechanical restraint was regarded as the most intrusive.	Poor
Bak, et al. (2014) Denmark & Norway	Questionnaire/ Quantitative	Inpatient psychiatric hospital units in Denmark (n=87) and Norway (n=96)	Clinical nurse managers (n=90).	To investigate manual restraint-preventative practices and their association with the frequency of manual restraint episodes in psychiatric hospital units in Denmark and Norway	Three factors were found to be associated with lower rates of mechanical restraint: mandatory review (exp[B] = .36, p < .01), patient involvement (exp[B] = .42, p < .01), and no crowding (exp[B] = .54, p < .01).	Good
Bak, et al. (2015) Denmark & Norway	Questionnaire/ Quantitative	Inpatient psychiatric hospital units in Denmark (n=87) and Norway (n=96)	Clinical nurse managers (n=90).	To examine how manual restraint- preventive factors may be associated with the differing number of manual restraint episodes in Denmark and Norway.	Staff education [exp(B)= 0.34, P=0.00], was associated with a lower frequency of manual restraint episodes in Denmark. In Norway, three factors were associated with a higher frequency of manual	Good

					restraint episodes: cognitive milieu therapy [exp(B)=7.46, P=0.00], patient-centred care [exp(B)=5.01, P=0.00] and alarm systems [exp(B)=3.72, P=0.00]. Six factors were associated with the difference in manual restraint episodes between the two countries: identification of the patients' crisis triggers [exp(B)=- 10%], patient–staff ratio [exp(B)=-11%], staff education [exp(B)=-51%], acceptable work environment [exp(B)=-15%], substitute staff [exp(B)=-17%] and separation of acutely disturbed patients [exp(B)=13%].	
Barr, et al. (2019) Australia	Interviews/Qualitative	State Forensic Mental Health Service	31 registered and enrolled nurses	To: (i) document the experiences of nurses working in the forensic mental health setting, (ii) articulate their perceived unique skill set to manage challenging patient behaviours, and (iii) determine how their experiences and skill set can inform practice changes to reduce the use of restrictive practices.	Four categories: Working in a challenging but interesting environment, Specialty expertize, Exposure to aggression and resilience as a protective factor, Effective teamwork and leadership	Good
Bergk, et al. (2011) Germany	Randomised controlled trial/ Quantitative	A psychiatric hospital	Psychiatric inpatients in the hospital (n=102). Those randomly assigned to seclusion intervention (n=12). 75% male. Mean age 40.8 (SD=10.1) (range 23-61). Those randomly assigned to mechanical restraint intervention (n=14). 71% male. Mean age 38.6 (SD=12.0)	To explore opinions regarding the restrictiveness of seclusion and mechanical restraint on psychiatric patients	There was no significant difference in perceived coercion (CES scores) between the seclusion and mechanical restraint groups. No significant differences were found between the seclusion and mechanical restraint groups in the number of adverse events. The mechanical restraint	Fair

			(range 20-63). Those not randomly assigned to seclusion intervention (n=48). 38% male. Mean age 40.2 (SD=12.1) (range 19-66). Those not randomly assigned to mechanical restraint intervention (n=28). 46% male. Mean age 39.7 (SD=13.4) (range 18-64).		group experienced lower levels of fear then the seclusion group (mechanical restraint median score =1.00, range= 1–5; seclusion median score=2.25, range=1–5, p=.049).	
Bigwood & Crowe (2008) New Zealand	Descriptive phenomenological/ Qualitative	An acute adult psychiatric inpatient service	Registered comprehensive nurses and registered psychiatric nurses working in the service (n=7). 57% male.	To examine how nurses experience physical restraint of patients within an inpatient psychiatric service	The predominant theme was one of 'it's part of the job' and expected. However, participants describe being conflicted and fearful of physical restraint and prefer to use other management techniques.	Fair
Blair, et al. (2017) USA	Pre-post intervention/ Quantitative	Psychiatric inpatient service	Patient admissions pre-intervention (n=3884). 49.7% female.<br><br>Patient admissions post-intervention (n=8029). 48.5% female.	To describe an intervention designed to decrease seclusion and restraint and present the results of a pilot study that evaluated the effectiveness of this program.	Statistically significant associations were found between the intervention and a decrease in both the number of seclusions (p < 0.01) and the duration of seclusion per admission (p < 0.001). These preliminary results support the conclusion that this intervention was effective in reducing use of seclusion.	Fair
Bleijlevens, et al. (2013) Netherlands	Quasi-experimental and questionnaire/ Mixed methods	Psychogeriatric nursing home wards (n=15) in Dutch nursing homes (n=6)	Nursing home staff (n=143); Nurse specialists (n=2); delegates representing nursing home associations; relatives of nursing home residents (n=38 residents).	To assess an intervention to reduce the use of belt restraints in psychogeriatric nursing homes.	In more than 50% of the cases in which a belt was removed, no alternative interventions were used. The most frequently used alternative interventions were infrared barrier alarm systems (21%) and adjustable low-height beds (12%). 96% of participants felt that the intervention met their learning needs. 76% of the 38 resident's relatives did not agree with the use of belt restraints but thought it	Fair

					was necessary before the intervention, and 78% were satisfied with the policy change. 79% felt involved in the decision making process regarding belt restraint removal. Three barriers to implementation of the intervention were found: availability of preferred alternative interventions, removing all physical restraint at the same time (increasing risk), and time constraints.	
Bonner, et al. (2002) UK	Semi-structured interviews/ Qualitative	Psychiatric inpatient unit(s)	Staff in the units (n=12); patients in the units (n=6)	To explore the experiences of restraint from patient and staff perspectives in psychiatric inpatient units	Ward atmosphere and failed communication were seen as antecedents to restraint incidents. The incident itself was characterised by fear and embarrassment, with staff regarding restraint as a last resort. Debriefing and the need for understanding the incident were reported as helpful after a restraint incident. Both patients and staff spoke of the fear of re-traumatization and the difference in care from temporary and permanent members of staff.	Fair
Bonner & Wellman (2010) UK	Questionnaire/ Quantitative	Acute psychiatric inpatient units and PICUs within an NHS Mental Health Trust	Staff in the units (n=30), 57% female; patients in the units (n=30), 57% female	To explore the usefulness of post incident reviews after incidents of restraint in psychiatric inpatient units	97% of staff and 94% of patients reported that they found the post incident review process useful. The element that most participants agreed was useful was the opportunity for discussion after an incident (100% of staff; 93% of patients). 61% of staff and 20% of patients believed the incident could have been	Fair

					predicted. 67% of staff and 50% of patients believed the incident had been well managed.	
Boumans, et al. (2012) Netherlands	Questionnaire/Quantitative	Long stay wards (n=2); a forensic psychiatric ward; and a crisis intervention ward within a psychiatric hospital.	Staff in the units (n=60). 57% male.	To explore the importance of several factors in nurse decision making on seclusion and to explore the effect of reflexivity on the decision to seclude patients.	Approachability was found to be the patient variable with the greatest impact on likelihood of seclusion: if the patient was ‘hardly approachable’ the mean tendency to seclude was 0.52 higher than when they were deemed ‘approachable’ (95% CI 0.44, 0.60). The more reflexive a team was, the less likely they were to seclude (Pearson correlation coefficient -0.97, P = 0.017).	Fair
Bowers, et al. (2012) UK	Randomised controlled trial/Quantitative	Acute psychiatric wards (n=136) in 67 hospitals within 26 National Health Service (NHS) Trusts	All staff and patients within the units	To explore the relationship of manual restraint and show of force to conflict behaviours, containment methods, environment, routines and staff variables in acute psychiatric wards	Manual restraint was used less (0.20 incidents per day) than show of force (0.28 incidents per day). Both were associated with aggressive behaviours and the enforcement of treatment and detention. Staff provision was associated with the use of these coercive interventions. Clearer ward structure and routine was associated with decreased use of manual restraint and show of force.	Fair
Bowers, et al. (2007) UK, Netherlands, Finland, and Australia	Questionnaire/ Quantitative	Psychiatric inpatient settings	Psychiatric professionals (n=844). UK (n=114; modal age under 30; 61% female). Netherlands (n=146; modal age under 30; 65% female). Finland (n=304; modal age 30-39; 44% female). Australia (n=280; modal age 40-49; 67% female).	To explore the attitudes towards containment measures of psychiatric professionals in four countries	Staff in Finland expressed the highest level of approval of containment, and staff in the UK expressed the least. Preferences for different containment measures were influenced by whether they considered it: safe for the patients; preventative of injury to others; and rapidly	Fair

					calming.	
Brady, et al. (2017) Australia	Cross-sectional/Quantitative	Psychiatric hospital	Inpatients (n=625)	To examine patient experiences of restrictive interventions in inpatient settings	Most patients had experienced restrictive interventions (68%) and a third had experienced seclusion (35%). Reported benefits of restrictive interventions were lower in those who had experienced the most interventions.	Good
Braham, Heasley & Akiens (2013) UK	Questionnaire and semi-structured interviews/ Mixed methods	One admitting ward from four clinical services within a high secure hospital (male mental health, personality disorder, learning disability and the women's service)	Patients (n=31; 72% male; mean age 37; range 22-56) and staff (n=84) within the wards.	To evaluate the impact of night confinement in a high secure hospital	Night confinement was not shown to have any adverse effects. Before the pilot, the majority of staff (70%) felt that it could have adverse effects on patients, and the majority of patients (74%) felt that the effects would be minimal. After the pilot, 46% of staff felt that the impact had been minimal, and 58% of patients felt that the impact had been positive.	Fair
Chien, Chan & Kam (2005) China	Semi-structured interviews and clinical records/ Qualitative	Acute admission wards (n=2) within a psychiatric hospital	Psychiatric inpatients (n=30). Mean age 31	To explore the perspectives of psychiatric inpatients concerning the use of restraint.	The majority of participants reported positive feelings towards staff who had shown concern and had offered help during restraint. Negative effects of restraint were related to patient needs not being met. In particular: concern, empathy, active listening, and information about restraint.	Fair

Ching, et al. (2010) Australia	Questionnaires/Quantitative	Units within a secure forensic psychiatric hospital (n=5): male acute units (n=2); female acute unit (n=1); mixed-sex sub-acute unit (n=1); rehabilitation unit (n=1)	Phase 1: Staff (n=60; 50% female; modal age group 18-30) and patients (n=13; 31% female; modal age group 18-30). Phase 2: Staff (n=61; 48% female; modal age group 31-40) and patients (n=7; 29% female; modal age group 18-30).	To evaluate a group of interventions for reducing the use of seclusion in a forensic psychiatric hospital.	There was no change in levels of staff confidence to manage aggression from pre-post intervention. There was a significant reduction in reported absconding risk from pre-post intervention (F (1, 92) =4.2, p=0.04). Post-intervention, staff were more likely to report that seclusion was used therapeutically (t(112)=-2.41, p=0.02).	Good
Chu, et al. (2015) UK	Pre-post questionnaire/ Quantitative	Wards (n=6) within a high-secure forensic mental hospital	Patients (n=30). Mean age 39 (range 23-60). Ward staff: Night confinement respondents (n=144); EssenCES respondents (n=119)	To explore the views of staff and patients concerning a new night-time confinement policy within a high-secure forensic hospital	No significant differences from pre-post night-time confinement were found for patient sleep quality, behaviour, ward atmosphere, or therapy engagement. Patient attitudes towards night-time confinement generally became more positive from pre-post implementation. Staff attitudes remained largely negative, however, proportions decreased post-implementation.	Fair
Cormac, Russell & Ferriter (2005) UK	Questionnaire/ Quantitative	High secure hospitals and medium secure units (n=39)	Senior managers (n=39)	To explore the use of seclusion and seclusion policies and procedures in medium and high secure units	69% of the units reported using seclusion in the past year. All policies followed Code of Practice guidelines. Seclusion environment requirements varied. Additional requirements found in some policy documents were thought to potentially enhance practice: privacy and dignity, observation, fittings and fixtures, cleanliness, communication, physical health, ending of seclusion and contact with significant others.	Poor

Duxbury, et al. (2019) UK	Interviews/Qualitative	Acute mental health wards (n=14)	Staff (n=36)	To explore the views of staff regarding the value and impact of an intervention aimed at reducing levels of restraint (REsTRAIN Yourself)	Eight themes arose: leadership and impetus towards organizational change; data to inform practice; meaningful activities and events; changing hearts and minds; use of restraint reduction tools; patient voice and agency; debriefing techniques; and embedding and sustaining development	Fair
Elmer, et al. (2018) Germany and Switzerland	Questionnaires/Quantitative	Inatient departments of psychiatric clinics (n=5)	Mental health professionals (n=424). 62% female. Modal age group 26-35	To investigate mental health professionals' recognition, attitude, and application of informal coercion in psychiatric institutions	Stronger forms of coercion were more likely to be underestimated by respondents. Acceptance of coercive measures was predicted by use of the measure by the respondent, and the attitude that coercion is a form of treatment and not an offence	Fair
Elzubeir & Dye. (2017) UK	Pre-post intervention/ Quantitative	Psychiatric intensive care unit	Pre-intervention: patients (n=70). 51% male. Mean age 38.9. Post-intervention: patients (n=23). 74% male. Mean age 32.6	To investigate the effect on rate and duration of seclusion episodes when Specific, Measurable, Achievable, Reproducible, Time bound (SMART) targets are agreed with patients and health professionals for ending a period of seclusion.	Following the intervention, there was a reduction in the total number of seclusion episodes in all settings as well as a reduction in total seclusion time spent in both the Low Stimulus Area and the Locked Seclusion Room. We also observed a decline in the number of seclusion episodes in the less restrictive, longer-term seclusion setting (ECS) although overall there was a four-fold increase in the time spent in the longer-term seclusion setting.	Good

Espinosa, et al. (2015) USA	Pre-post evaluation/Mixed methods	Inpatient psychiatric units (n=15) within a large multi-site medical centre	Staff and patients	To evaluate interventions to improve milieu on psychiatric inpatient units	Restraint and seclusion rates reduced dramatically, but this meant that staff confidence in their skills regarding these interventions decreased. Violent incidents also reduced in frequency.	Poor
Exworthy, et al. (2001) UK	Questionnaire/ Quantitative	Maximum security hospitals (n=3) and medium security units	Psychiatrists and doctors (n=117)	To explore the views of doctors and psychiatrists concerning the practice of seclusion in secure units	56.4% of respondents agreed that seclusion is a form of treatment. 82% agreed that it was not a form of punishment. 60% felt that it helped avoid the use of medication and 86% support its use when a patient is threatening physical violence to others. 70% were against its use for self-harm behaviours. 45% felt that seclusion should be defined in law.	Poor
Ezeobele, et al. (2014) USA	Semi-structured interviews/ Qualitative	A psychiatric acute care hospital	Patients (n=20). 60% male. Mean age 28 (range 19-53)	To explore the psychiatric patients' lived seclusion experience.	60% of participants felt that seclusion was a penalizing and negative experience. Themes regarding the experience of seclusion were: feeling alone, staff exerting power/control, feeling resentful of staff, and having time for meditation.	Good
Faschingbauer, Peden-McAlpine & Tempel (2013) USA	Semi-structured interviews/ Qualitative	Psychiatric inpatient facilities	Psychiatric inpatients (n=12). 50% female. Mean age 33 (range 18-50)	To explore the psychiatric patients' lived seclusion experience.	Themes described by patients regarding the experience of seclusion were: hope for respect and communication, emotional response to seclusion (heightened anger/anxiety), and insight into behaviour and positive coping skills (need debriefing with staff).	Fair

Fish & Hatton (2017) UK	Interviews/Qualitative	Female wards (n=3) within a locked forensic learning disability unit	Staff (n=10) and residents (n=16; 100% female)	To explore women's experience of physical restraint in a secure learning disability setting	Three themes arose: gendered experiences of restraint, reasons for using restraint and alternatives to restraint	Fair
Fish (2018) UK	Interviews/Qualitative	Female wards (n=3) within a locked forensic learning disability unit	Staff (n=10) and residents (n=16; 100% female)	To explore the experiences of seclusion of women in a secure learning disability setting	Four themes arose: the seclusion room environment, reasons for using seclusion, termination of seclusion and alternatives to seclusion	Fair
Georgieva, Mulder & Wierdsma (2012) Netherlands	Questionnaire/ Quantitative	Acute psychiatric hospital	Psychiatric inpatients (n=161); 54% female.	To examine patient's experience and preferences regarding coercive interventions and associated factors	Previous experience of seclusion predicted preference for seclusion in emergency situations. However, most inpatients preferred medication in an emergency situation.	Fair
Georgieva, Mulder & Noorthoorn (2013) Netherlands	Experimental/ Quantitative	Acute psychiatric hospital	Psychiatric inpatients (n=520). Involuntary medication, (n=236); 48% male; mean age 40 and seclusion (n=284); 53% male; mean age 40	To examine the number and duration of seclusion and coercive measures relating to inpatients who are allocated to involuntary medication intervention compared with seclusion (TAU)	Relative risk (RR) of seclusion was lower for patients who received involuntary medication than TAU. Seclusion episode duration and the number of coercive incidents were not significantly different between the two groups.	Fair
Goulet and Larue (2018) Canada	Participatory case study/quantitative	Acute psychiatric hospital	Patients (n=3) and staff (n=14)	To explore the context in which seclusion and restraint practice take place.	Three overarching themes were identified: patient characteristics (e.g. etiology of violence), staff characteristics (e.g. feelings of safety) and environmental characteristics (e.g. overcrowding).	Good

Goulet, Larue and Lemieux (2018) Canada	Participatory methodology/Qualitative	Acute adult psychiatric care unit	Staff (n=12) and patients (n=3)	To develop and evaluate post-seclusion and/or restraint review (PSRR) intervention	Seclusion use was significantly reduced 6 months after the implementation (21.3% to 10.4%; p.046) but not for restraint (p=0.334).	Good
Gowda, et al (2018) India	Cohort/Quantitative	Psychiatric hospital	Inpatients (n=200)	To examine prevalence of coercive measures used in a psychiiatric hospital	Across the patients, 66.5% experienced more or one restraint measures, 58% experienced chemical restraint, 32% involuntary medication, 20% experienced physical restraint and 18% experienced seclusion.	Fair
Gowda, et al (2019) India	Cross-sectional/Quantitative	Psychiatric hospital	Inpatients and caregivers (n=200)	To examine caregivers perspectives on coercion practices.	Chemical restraint was deemed the most acceptable (82.5%) followed by physical restraint (71%) and seclusion (25.5%).	Fair
Gowda, et al. (2019) India	Questionnaire/Quantitativ e	Inpatient psychiatry	189 psychiatrists	To assess clinicians' attitude and perspective on the use of coercive measures in psychiatric practice.	Coercion proved to be a common measure applied in nearly 70% of the patients studied. The 189 psychiatrists participating in the study almost all perceived coercion as care, protection and safety, and as protection from dangerous situations. About 66% of psychiatrists perceived physical and chemical restraint (sedation) as necessary and acceptable in acute emergency care. One-third of the	Fair

					psychiatrists felt their patients lost autonomy, dignity, and the possibility of interpersonal contact. The same amount agreed that some patients could have been treated with less restriction and fewer coercive measures.	
Gustafsson & Salzmann-Erikson (2016) Sweden	Interviews/ Qualititative	Forensic psychiatric care	Nurses (n=8). 63% female. Mean age 44.5 (range 32-55).	To describe nurses' experiences and thoughts concerning the exertion of coercive measures in forensic psychiatric care.	Results described participants' thoughts and experiences of coercive measures from four main categories: (a) acting against the patients' will, (b) reasoning about ethical justifications, (c) feelings of compassion, and (d) the need for debriefing.	Good
Hatta, et al. (2007) Japan	Cohort/Quantitative	PICU	Psychiatric inpatients; restrained patients (n=106) and non-restrained patients (n=528)	To investigate the effects of physical restraint on the development of drug induced liver injury	Prevalence of drug induced liver injury was significantly higher for those who were restrained (8.5%) than the non-restrained group (1.9%; odds ratio 4.81). Rates of those receiving antipsychotics were higher in the restrained group.	Fair
Haugom & Granerud (2016) Norway	Descriptive/ Qualitative	Psychiatric wards (n=57)	Staff (n=149)	To investigate how psychiatric patients and staff describe and assess the practice of shielding	Shielding is an ambiguous practice. Shielding as a form of control was seen as more important than as a form of treatment.	Fair

Haw, et al. (2011) UK	Semi-structured interviews and case note review/ Mixed methods	Low and medium secure wards within a psychiatric hospital	Patients (n=57); 48% male; median age 29 (range 19-52)	To explore patients' experiences of, and preferences for, physical restraint, forced medication and seclusion	Coercive treatments were generally perceived as negative experiences; however, 16% of participants reported that the last episode of seclusion or restraint had been positive. Most patients preferred medication to seclusion. Patients felt that advance statements and views concerning restrictive practice should be a part of their care plans.	Fair
Holmes, Kennedy & Perron (2004) Canada	Phenomenological semi-structured interview study/ Qualitative	Specialised psychiatric unit	Psychiatric inpatients (n=6)	To explore the experiences of seclusion of patients with severe and persistent psychiatric disorder	Themes emerged: emotional experience of seclusion (feelings of rejection, isolation, etc.); patients' perceptions of seclusion (as punitive and a way of exerting social control), and coping mechanisms. The lack of staff contact during seclusion seemed to have more of a negative impact on patients than the seclusion itself.	Fair
Holmes, Murray & Knack (2015) Canada	Phenomenological semi-structured interview study/ Qualitative	Forensic psychiatric inpatient hospital	Participants (n=26): Forensic psychiatric inpatients (n=13) and forensic psychiatric nurses (n=13)	To study the lived experience of the secluded room	Three main themes from inpatients emerged: experience of seclusion, assessing quality of care and space of confinement. Themes from staff interviews were: resorting to seclusion, observing and assessing patients and experiencing seclusion. The therapeutic relationship is important to both staff and patients.	Fair
Hottinen, et al. (2012) Finland	Descriptive/ Quantitative	Adolescent closed ward of a general hospital	Clinical staff (n=128); 74% female; 75% nurses	To investigate staff attitudes towards containment measures used in adolescent psychiatric wards	Preferred containment methods were medication, transfer to specialist locked wards and mechanical restraint. The net bed was the least preferred containment method.	Fair

Hotzy, et al (2019) Switzerland	Cohort/Quantitative	Acute psychiatric inpatient hospitals (n=3)	Patients (n=418), staff (n=364) and patient relatives (n=180)	To compare and contrast perspectives of using coercive measures in psychiatric sites in Switzerland	Mean global attitude towards containment measures was 2.51 (SD=0.66). PRN medication was deemed the most acceptable across groups (M 1.62 ± 0.85). Stark differences were identified across the three sites in relation to attitude and use of containment measures in psychiatric hospitals.	Good
Huizing, et al. (2006) Netherlands	Experimental/ Quantitative	Psychogeriatric nursing home wards (n=5)	Residents with dementia (n=167); baseline (n=145); experimental (n=83); mean age 82; 78% female and post-intervention (n=144); experimental (n=86); mean age 82; 73% female	To investigate the effects of an educational intervention on the use of physical restraints in psychogeriatric nursing home wards	Despite education on the use of restraint, the use of restraint did not significantly decrease in the experimental group. Residents in the control group experienced significantly more restraint than the educational group.	Fair
Jaeger, et al. (2014) Switzerland	Cross-sectional/ Quantitative	Acute psychiatric hospital	Mental health professionals (n=39); 59% female	To evaluate how staff recognise different levels of coercion and treatment pressures	Results showed low levels of coercion were adequately recognised, whereas high levels of coercion were underestimated.	Fair
Jalil, et al. (2017) UK	Cross-sectional, correlational and observational design/ Quantitative	Secure mental health hospitals (n=3)	Mental health nurses (n=68). 70.6% female. Mean age 41.6 + 9.0	To identify relationships between mental health nurses' exposure to patient aggression, their emotions, their attitudes towards coercive containment measures, and their involvement in incidents involving seclusion and restraint	Nurses who reported greater exposure to a related set of aggressive behaviours, mostly verbal in nature, which seemed personally derogatory, targeted, or humiliating, also reported higher levels of anger-related provocation. Exposure to mild and severe physical aggression was unrelated to nurses' emotions. Nurses' reported anger was significantly positively correlated with their endorsement of restraint	Good

					as a management technique, but not with their actual involvement in restraint episodes. Significant differences in scores related to anger and fatigue, and to fatigue and guilt, between those involved/not involved in physical restraint and in physical restraint plus seclusion respectively were detected.	
Johnston & Kilty (2016) Canada	Interviews/Qualitative	Psychiatric units (n=2)	Security guards (n=8). 100% male	To explore how security guards use neutralization to shift responsibility during violent encounters on to medical staff in psychiatric units	Themes drawn from the interviews included: mitigation of responsibility through discourses of blame; refusing injury: ''It's for their own good''; constructing patients as dangerous and deserving of punishment; condemning the nurses; and appealing to militant codes of conduct	Fair
Keski-Valkama, et al. (2007) Finland	Descriptive/ Quantitative	Psychiatric hospitals	Patients restrained in 1990 (n=94), 1991 (n=107), 1994 (n=71), 1998 (n=59), 2004 (n=59). Patients secluded in 1990 (n=75), 1991 (n=43), 1994 (n=86), 1998 (n=40), 2004 (n=36)	To examine the use of restraint and seclusion over time	The number of seclusion and restraint incidents decreased over the five time periods; however the duration of seclusion significantly increased over time (v2 (4) = 36.111, $p < 0.001$).	Fair
Kirkevold & Engedal (2004) Norway	Descriptive/ Quantitative	Regular units (RU) for patients with dementia (n=142). Special care units (SCU) for inpatients with dementia (n=80)	Staff interview details not recorded. Interviews based on inpatients (n=1501): RU (n=1057); 66% female; mean age 84.4 [6.9]; SCU (n=444); 75% female; mean age 83.4 [6.1]	To identify the prevalence of patient restraint in nursing homes	Significantly more restraint occurred in SCUs than in RUs (45.0% and 36.7% respectively). The most common restraint interventions were mechanical restraint and use of force/pressure in daily activities.	Fair

Knowles, Hearne & Smith (2015) UK	Semi-structured interview/ Qualitative	Medium secure unit	Inpatients (n=8); mean age 39; 13% female	To explore the impact of employing physical restraint on maintaining therapeutic relationships between staff and patients	Five themes were found: Restraint reinforces the inequality of power in the staff-patient relationship; abusive, degrading and traumatic experiences; whether restraint justification influences whether it is an accepted intervention; negative attitudes and motives of some staff; and coping with having no power during and after restraint.	Fair
Kontio, et al. (2012) Finland	Interviews/ Qualitative	Psychiatric inpatient hospital	Inpatients (n=30); 37% female; mean age 41 (range 20-64 years)	To explore patient perspectives on the use of, and future directions of restraint and seclusion	In general patients had negative experiences during seclusion/restraint and did not have enough information before it happened regarding why the intervention occurred. Future improvement ideas included recent information about their care plan and treatment, and reasons why they were restrained. Alternatives to restraint and seclusion included empathetic patient–staff interaction, meaningful activities, therapeutic community, and biological treatments.	Fair
Kontio, et al. (2011) Finland	Experimental/ Quantitative	Psychiatric inpatient wards in general hospital (n=12)	Nurses (n=158); intervention group (n=95); 55% female; mean age 43 [9.0] (range 25-60) and control group (n=63); 52% female; mean age 45 [10] (range 24-64)	To examine the effect of an online learning course on nurse competence in restraint and seclusion interventions	Knowledge of physical restraint improved at three month follow-up whereas knowledge of seclusion did not change. Self-efficacy increased in the intervention group.	Fair
Kontio, et al. (2009) Finland	Focus group/ Qualitative	Psychiatric hospital (n=2)	Staff (n=27); 52% female; mean age 44 years (range 26-59)	To examine staff perspectives on current educational needs in relation to implementing seclusion and restraint, and their future needs	Difference found on guidance regarding seclusion and restraint: some staff wanted structured guidelines and some wanted to rely on knowledge/experience. All seemed to want more education regarding the ethical, clinical and legal	Fair

					aspects of restraint and seclusion interventions. This was in addition to a desire for staff support to ensure the success of the intervention.	
Kontio, et al. (2010) Finland	Focus group/ Qualitative	Psychiatric hospital (n=2)	Staff (n=27); 52% female; mean age 44 years (range 26-59)	To explore what happens when an aggressive incident occurs in a psychiatric ward and what alternatives to restraint and seclusion are possible	Staff felt that the patient perspective was not considered enough. Staff also proposed numerous alternatives to seclusion and restraint including being present and having a conversation with the patient. Cooperation and communication via multi-professional agreements that involve the patient perspective were deemed useful.	Fair
Krieger, et al. (2018) Germany	Naturalistic/quantitative	Psychiatric intensive units (n=3)	Involuntary patients (n=213) and voluntary patients (n=51)	To examine patient's attitudes towards coercion	Perspectives varied aross the four different types of coersion (mechanical restraint, forced medication, seclusion, involuntary hospitalisation). The majority of patients felt helpless and desparate particularly during mechanical restraint (80.6% and 75% respectively) and seclusion (90.0% and 73.7% and respectively). Patients reported that they would prefer a soft room and least prefer mechanical restraint when "coercive interventions against my will are neccessary" (M=2.46 ± 1.26, M=3.19 ± 1.05)..	Good

Kuosmanen, et al. (2015) Finland	Experimental/ Qualitative	Acute psychiatric hospital	Staff (n=2)	To explore the experience of being secluded and to understand and evaluate the impact of seclusion from a staff perspective.	Overall, staff perceived their seclusion experience as negative and inhumane. The staff felt anxiety and frustration at being locked up. Future practical suggestions for change included updating seclusion practice guidance and re-designing seclusion facilities. Ideas included introducing a clock and normal height bed and chair.	Poor
Larsen & Terkelsen (2014) Norway	Observational/ Qualitative	Locked psychiatric ward	Inpatients (n=12). Staff (n=22)	To explore coercion experience	There were four main themes: corrections and house rules, coercion as a necessary intervention, material surroundings as being of great importance, and being treated as a human being. Staff and patients differed in their experience. Patients felt inferior, and staff sometimes felt guilty when implementing the interventions.	Fair
Larue, et al. (2013) Canada	Structured interview/ Mixed methods	Psychiatric hospital	Inpatients (n=50); 38% female	To identify and describe perceptions of the seclusion and restraint protocol in a psychiatric hospital	Just over half of inpatients indicated they were not offered an alternative to restraint (n=28). Eighteen patients felt seclusion fulfilled their need for calm, sleep and safety. Almost all said staff did not follow up with them after their seclusion/restraint experience and deemed this as an essential need in the future.	Fair

Larue, et al. (2016) Canada	Focus groups/Qualitative	A psychiatric hospital intellectual disability programme	Staff (n=24) and family members (n=7)	To explore how staff and family members of psychiatric inpatients feel about reductions in coercive measures and the interventions used by staff that contributed to this reduction	Four themes arose: leadership; organisational culture; characteristics of the care providers; and characteristics of the patients	Fair
Lee, et al. (2003) UK	Descriptive/ Mixed methods	Secure inpatient mental health unit and PICU	Nurses (n=269)	To examine staff views of their last experience of employing physical restraint	The majority of nurses reported positive results after the coercive incident. Issue raised regarding the after effects of the incident were concern and ambivalence. Negative aspects of interventions included injury, management and clinical issues. Future improvements reported included less crowed environment, improved staff training in de-escalation and adopting positive care philosophy around relationships with patients	Poor
Lee, et al. (2001) UK	Cross-sectional/ Quantitative	Regional secure inpatient mental health units and PICUs (n=63)	Nurses (n=338); 53% female; mean age 36.2 [8.9] (range 19-63)	To investigate training in physical restraint in order to compare course content and length, and injuries in training	Course content varied despite a core curriculum in place. Length of course also differed (range 0.5-21 days) but two thirds attended a 5 day course. Sometimes refresher courses did not happen as expected. About a third of respondents reported being hurt in training.	Poor
Long, et al. (2015) UK	Pre-post intervention design/ Quantitative	Medium secure inpatient unit	Patients (n=38). Experimental group (n=19), and matched control group (n=19); mean age 31.1 (range 19-49); 100% female.	To assess the effectiveness of interventions designed to minimise the use of seclusion in response to risk behaviours.	There was a significant decrease in the number of seclusion incidents and risky behaviour post-intervention change, as well as more positive staff ratings of patient behaviour, improved treatment engagement	Fair

					and reduced patient time spent in medium security. Staff perceived training and use of de-escalation techniques as the most effective whereas patients felt individual engagement and initiatives to reduce bullying, harassment and discrimination was the most effective.	
Looi, Engstrom & Savenstedt (2015) Sweden	Self-report/Qualitative	Psychiatric inpatient unit	Inpatients (n=19)	To explore the perceptions of people who self-harm regarding alternatives to coercive measures and how this relates to lived experiences of psychiatric care	Content analysis revealed three main themes: understanding rather than neglect, mutual relation rather than distrust, and professionalism rather than counterproductive care.	Fair
Lovell, Smith & Johnson (2015) UK	Semi-structured interviews/ Qualitative	Secure learning disability service	Nurses (n=20)	To explore the views of learning disability nurses regarding physical intervention incidents, and contributory factors of injuries sustained	The overarching theme, knowledge and understanding, placed three other themes in context: physical intervention techniques employed, staff interpretation of the safety incident and the impact on staff. All staff felt it was important to know the patient and use an individualised approach when an incident occurred.	Fair
Mackay, Paterson & Cassells (2005) UK	Unstructured interviews/ Qualitative	Sector Acute Psychiatric Admission wards (n=3) and PICU (n=1)	Nurses (n=6)	To explore the perceptions of observing for patients with perceived risk of violence/aggression and important factors in day to day practice	Procedure, skills and role were identified as three higher level categories. An additional six categories made up "role": intervening; maintaining patient safety and that of others; prevention de-escalation; and managing aggressive and violent incidents; assessment; communication; and therapy. Staff skills were built within this and	Fair

					deemed interconnected.	
Martello, et al. (2018) Canada	Interviews/Qualitative	An inpatient psychiatric unit	Nurses (n=6). 84% female	To explore the views of nurses towards engaging with patients to reduce the use of restrictive practices in an inpatient psychiatric unit	Four themes arose: unit engagement practices, managing the escalation by engaging with patient, engaging during the use of restrictive practices, and factors influencing engaging with patient to reduce restrictive practices	Good
Mason & Whitehead (2001) UK	Structured interview/ Mixed methods	Forensic psychiatric inpatient hospital	Female inpatients	To explore the problems associated with secluding female inpatients	There were 34 seclusion incidents with the longest duration identified as 23 days, 8 hours and 45 minutes. The most common reason for seclusion was actual violence to staff (928 hours), threat of violence (238 hours) and threat of property damage (129 hours). Staff rationale for stripping patients while in seclusion was for patients' safety. PRN medication was usually given before a seclusion.	Fair
McKeown, et al. (2019) UK	Interviews/ Qualititative	Acute mental health wards (n=14)	Staff (n = 130). 52% male.<br>Service users (n = 32). 47% male.	To explore views on staffing levels in a context of attempting to minimize physical restraint practices on mental health wards.	Five themes were identified regarding how staffing levels impact experiences and complicate efforts to minimize physical restraint. Themes—“insufficient staff to do the job”; “detriment to	

					staff and service users"; "a paperwork exercise: the burden of non-clinical tasks"; "false economies"; and, "you can't do these interventions."	
Mistler, et al. (2017) USA	Pre-post evaluation/Mixed methods	An acute care state hospital	Inpatients (n=13). 83% male; 92% white; mean age 33 [SD 10.7]	To examine the feasability, usability and acceptability of a mindfulness mobile app intended to reduce aggression in psychiatric inpatients	All participants reported the app to be easy to use and engaging. 83% of participants felt comfortable using the app and would recommend it to others. Participants reported that the app gave them a sense of control and alleviated boredom	Fair
Molewijk, et al. (2017) Norway	Cross-sectional/ Quantitative	Acute ward (n=2)	Mental health care professionals (n=379). 40% male.	To examine the following:<br>1). What are mental health care professionals' normative attitudes towards coercion and how often do they experience moral doubt?<br>2). How is health care professionals' experienced moral doubt related to their normative attitude towards coercion?<br>3). How are professional and contextual characteristics related to the staff's normative attitude towards coercion and to experiencing moral doubt?	On average employees neither agreed nor disagreed with the statements that described coercion as offending, respondents from Acute Care (Hospital 2) seemed to agree a bit more that coercion could be seen as offending. On average, respondents from all wards agreed that coercion could be seen as care and security.	Good

Muir-Cochrane, et al. (2015) Australia	Semi-structured interviews/ Qualitative	Old age psychiatric unit (n=3)	Mental health nurses (n=39)	To explore nurses experiences of restraint and seclusion and resistance to getting rid of these interventions	"Lack of accessible alternatives to restraint and seclusion" was the overarching theme. Interrelated themes covered environmental factors contributing to restraint and seclusion interventions; the consequences of poor staff-patient relationships; and the influence of ward environment on restraint and seclusion intervention implementation (an unfavourable physical environment impacts upon the aggression, restraint and seclusion tactics used).	Fair
Newman, Paun & Fogg. (2018) USA	Pre-post intervention and survey /Mixed methods	Adult inpatient psychiatric unit	Staff members (n=35) who completed the 3-month post-survey	To evaluate the effects of a 90-minute trauma-informed care-based staff training on patient seclusion rates in an adult psychiatric unit.<br><br>To evaluate staff knowledge and attitudes about seclusion trauma, commitment to seclusion alternatives, confidence in personal de-escalation skills, and use of new de-escalation.	Seclusion rates were reduced from a 6-month preintervention average of 2.95 seclusion hours per 1,000 patient hours to a 6-month post-intervention average of 0.29 seclusion hours per 1,000 patient hours, a 90.2% reduction.<br><br>Completed staff surveys showed significant staff knowledge gains, non-significant changes in staff attitudes about seclusion, non-significant changes in staff de-escalation skill confidence, and use of the new resource sheet by only 17% of staff .	Fair

Nielsen, et al. Denmark (2018)	Focus groups/ Qualititative	Forensic mental health setting including secure units (n=4) and rehabilitation unit (n=1)	Clinicians (n=17) composed of nurses (n=8), social and healthcare assistants (n=8) and nurse assistant (n=1). 76% female. Age range 31-60	To report on clinicians' experiences of the clinician-patient alliance during mechanical restraint (MR), including what and how parameters of alliance are assessed regarding the patient's readiness to be released from MR.	The results show that a pre-established personal clinician-patient alliance formed the basis for entering into, and weighing the quality of, the alliance during MR. In consideration of the patient's psychiatric condition, the clinicians observed and assessed two quality parameters for the alliance: 'the patient's insight into or understanding of present situation' (e.g. the reasons for MR and the behaviour required of the patient to discontinue restraint) and 'the patient's ability to have good and stable contact and cooperation with and across clinicians.	Fair
Papadopoulos, et al. (2012) UK	Cross-sectional/ Qualitative	Acute inpatient psychiatric wards (n=16)	Ward managers and consultant psychiatrist (n=120)	To investigate what influences the likelihood of patient conflict (verbal abuse, violence, and rule breaking) and containment (seclusion, manual restraint, and enforced medication)	Negative staff morale increased the likelihood of conflict and containment, whereas positive staff practice (environment improvement, increased staff activity, proactive ward manager) decreased the likelihood of such events occurring.	Good
Pellfolk, et al. (2010) Sweden	Cluster-randomized controlled trial/ Quantitative	Group dwelling units for people with dementia (n=40)	Control group at baseline: Residents (n=162); mean age 83.4 and staff (n=162); mean age 43.2. Intervention group at baseline: Residents (n=191); mean age 80.5 and staff (n=184); mean age 43.5	To evaluate a restraint minimization education program.	In the intervention group, staff had more knowledge about restraint, but attitudes toward restraint use were not significantly different from the control group. The likelihood of being restrained was lower in the intervention group (OR) =0.21, 95% (CI) =0.08–0.57, *P*=.002, n=281).	Good

Perkins, et al. (2012) UK	Semi-structured interviews and focus groups/ Qualitative	An acute adult mental health setting	Nursing staff (n=30)	To explore the attitudes of staff towards restraint.	Factors thought to influence the decision to restrain were: contextual demands; lack of alternatives; the escalatory effects of restraint; and perceptions of risk. Nurses described restraint as a "necessary evil" due to the unpredictability of mental illness and their work environment.	Fair
Raboch, et al. (2010) Europe and Israel	Questionnaire/ Quantitative	Psychiatric inpatient facilities	Patients (n=2,030); age range 18-65.	To compare the use of coercive measures in psychiatric inpatient facilities in different European countries.	1,462 coercive measures were used with 770 patients (38%). 21%-59% of patients received coercive measures, with the most common reason being patient aggression against others. In eight of the ten countries, the most common measure was forced medication, and in two of the countries it was mechanical restraint. Seclusion was rare (only 6 countries reported it).	Fair

Raveesh, et al (2016) India	Questionnaire/ Quantitative	Department of Psychiatry	Psychiatrists (n=210). 80% male. Caregivers (n=210). 54.4% male.	To assess attitudes of Indian psychiatrists and caregivers toward coercion.	Both groups agreed that coercion was related to scarce resources, security concerns, and harm reduction. Both groups agreed that coercion is necessary, but not as treatment. Older caregivers and male experienced psychiatrists considered coercion related to scarce resources to violate patient integrity. All participants considered coercion necessary for protection in dangerous situations. Professionals and caregivers significantly disagreed on most items.	Good
Reisch, et al (2018) Switzerland	Cross-sectional/Quantitative	Psychiatric hospital (n=3)	Patients (n=435), Staff (n=372) and patient relatives (n=230)	To compare and contrast perspectives of using coercive measures in a psychiatric hospital	34.7% of patients had experienced severe coercion; of these, the measures were rated less acceptable than those who hadn't had that experience (t=3.15, p=0.002). Staff felt coercive measures were more acceptable than patients or their family members. Patients also strongly rejected intramusclar injection medication.	Fair

Reynolds, et al. (2016) USA	Naturalistic/quantitative	Youth psychiatric inpatient setting	Young inpatients (n=1485)	To examine the effectiveness of the Positive Behavioural Interventions and Supports (M-PBIS) to reduce seclusion and restraint in a youth psychiatric inpatient setting	The number of seclusion and restraint incidents significantly reduced after using the intervention (pre=142 to post=102; p<0.001). The number of prescribed PRN medications also significantly reduced (pre=301 to post=223; p<0.001).	Fair
Rippon, Reid & Kay. (2018) UK	Focus groups and interviews/ Qualititative	CYP (children and young people) psychiatric inpatient services	Health professionals (n=11), non-clinical staff (n=6), service users and relatives (n=9)	To gain an understanding of the attitudes of frontline health professionals, non-qualified staff, patients and relatives regarding the use of restrictive practices to manage behaviours that challenge in CYP psychiatric inpatient services.	Although restrictive practices were sometimes seen as necessary to manage certain situations – for instance, when staff felt there was a risk of a patient committing suicide – they were also considered to be potentially harmful to staff and patient wellbeing.	Fair
Ryan & Bowers (2005)	Cross-sectional observational/ Qualitative	A PICU in an inner city hospital	Staff nurses and patients	To explore the implementation of coercive manoeuvres in a PICU.	Within the PICU, nurses practised several coercive strategies, which enabled them to manage low-level conflict situations. More serious conflict necessitated	Fair

					more severe containment methods. For example, ‘time out’ was used regularly (confinement in the patient’s own bedroom or in an open seclusion room), as well as seclusion.	
Ryan & Happell (2009) Australia	Participatory action research/Qualitative	Adult acute mental health inpatient units with high-dependency unit suites and seclusion facilities (n=2)	Mental health nurses (MHN) (n= 31) and consumer consultants (CC) (n=4)	To describe current practice and debriefing needs of staff, in order to gauge the need for a training program to facilitate post-seclusion debriefing.	Themes emerged from the data: 1) Debriefing, support, and flexibility- MHNs stated that debriefing consumers after seclusion is important. 2) Inherently unethical? Some CCs had concerns around being involved in a debriefing program, for example: “Seclusion is not an evidence-based intervention, it is a breach of human rights” 3) Support us, don’t preach to us- debriefing should be about providing psychological support. Consumer choice is also important.	Good
Schreiner, Crafton & Sevin (2004) US.	Pre-post intervention study/ Mixed methods	An adolescent inpatient unit	Inpatients (n=23); 56% male; age range 13-17.	To describe the effects of a staff re-education campaign in reducing the use of physical restraints treating adolescents with developmental delays and severe psychiatric disturbances	During the assessment phase, a monthly average of 18.67 seclusion events were recorded. During the intervention phase, monthly seclusion events declined to 12.14, a decrease of 35%.	Fair
Seo, Kim & Rhee (2012) South Korea	Descriptive/ Quantitative	Psychiatric hospital	Inpatients (n=248); 38% female; mean age 38.6 [11.4]	To examine whether coercive interventions can be justified by the assumption of incompetence, the assumption of dangerousness and the assumption of impairment	Legal status, perceived coercion and experienced coercive measures were justified as measures of coercion under the assumption of incompetence.	Poor

Sequeira & Halstead (2001) UK	Interviews/ Mixed methods	Secure wards in a psychiatric hospital (n=5)	Patients (n=116); 22% female; age range 13-54.	To investigate the use of coercive measures with patients who have developmental disabilities and to explore the experience of these measures.	Women had a significantly higher probability of being given rapid tranquilization following violent incident, seclusion was more likely to be used with men. Interviews with women demonstrated a commonly held understanding of interventions as punishment and expressions of intense anger and anxiety.	Fair
Sequeira & Halstead (2004) UK	Cross-sectional / Qualitative	Secure mental health service	Nursing staff (n=17); 53% male; age range 18-50. Restrained patients (n=14) and patients who observed the same event (n=5)	To examine the experiences of physical restraint processes reported by nursing staff	A range of emotional responses by nursing staff included distress, anxiety and crying. These were sometimes corroborated by patient descriptions. Staff used laughter to reduce stress following an incident, and many reported feeling little emotion during incidents, instead taking any distress home.	Fair
Smith & Jones (2014) UK	Descriptive and semi-structured interviews/ Mixed methods	A PICU within an inner city heath service	Staff members (n=10); 90% male. Patients (n=7); 100% male	To explore the effects of a sensory room on a PICU on seclusion rates and staff and patients' experiences	Interviews revealed a perception by staff that seclusion rates had reduced, despite no significant reduction occurring. Both staff and patients expressed that the sensory room was positive and therapeutic, improving staff-patient communication and patients' experience of the PICU.	Fair

Steinert, et al. (2007) Germany	Prospective/ Quantitative	Psychiatric hospitals (n=10)	Treated cases (n=36,690)	To investigate the prevalence of coercive measures in different psychiatric hospitals	9.5% of 36,690 cases were exposed to coercive measures. On average, these measures were applied 5.4 times per case and lasted 9.7 hours each. The use of seclusion and restraint guidelines was associated with a lower incidence of coercive measures.	Fair
Sustere & Tarpey (2019) UK	Interviews/Qualitative	A medium secure unit within an NHS hospital	Inpatients (n=12). 100% male	To explore patients' experiences of least restrictive practice and it's relationship to independence and recovery	Five themes arose: positive changes, perceived lack of transparency, social isolation, institutionalisation, and normality	Fair
Tateno, et al. (2009) Japan	Cross-sectional vignette study/ Quantitative	Psychiatric inpatient facilities	Young psychiatrists (n=183)	To explore Japanese psychiatrists' attitudes about emergency coercive interventions for patients with acute psychosis.	Most participants agreed that the vignette case should be admitted to hospital and secluded. Regarding the likelihood of prescribing restraint, results varied; psychiatrists at general hospitals were more likely to prescribe restraint than those working at university or psychiatric hospitals.	Fair
Tompsett, Domoff & Boxer (2011) USA	Prospective/ Quantitative	A secure psychiatric hospital	Adolescents (n=149); 59% male; mean age 14 (SD=3)	To investigate predictors of aggression in adolescent inpatients.	Unique predictors of restraint involvement were found: history of aggression against adults and history of previous psychiatric hospitalizations. No predictors were significant for the extent of restraint involvement.	Good

Vedana, et al (2018) Brazil	Semi-structured interviews/Qualitative	Psychiatric units (n=2)	Nurses (n=29)	To explore and understand nurses experiences and perceptions of using physical restraint.	Almost all staff had had training about how to perform physical restraint (93.1%) but over half had experienced restraint related accidents (58.2%). Three main themes were identified: agressiveness and restraint: unpleasant, challenging and harmful situations; the need and purpose of the physical restraint and strategies to reduce physical restraint-related damage.	Fair
Wharewera-Mika et al. (2016) New Zealand	Focus groups/Qualitative	District health boards (n=4)	Cultural advisors (n=5), Maori nurse leaders (n=5), Maori consumer advisors (n=2), Maori elders (n=4)	To explore Maori views of initiatives to reduce or prevent seclusion of mental health inpatients	Three themes arose: access to a Maori wordview; transforming practice; and Rangatiratanga (leadership, power, and control)	Fair
Whitecross, Seeary & Lee (2013) Australia	Interventional/ Quantitative	Inpatient wards	Patients in the TAU group (n=14); 71% male; mean age 35.8 (SD=9.9). Intervention group (n=17); 76% male; mean age 37.8 (SD=10.1)	To identify the impacts of seclusion on patients and evaluate the effectiveness of a post-seclusion counselling intervention in reducing time spent in seclusion and trauma experienced.	There was no difference in trauma experience between the TAU group and post-seclusion counselling intervention group.	Fair
Whittington, et al. (2009) UK	Cross-sectional/ Qualitative	Acute care mental health services	Service users (n=1,361); 67% female. Staff (n=1,226); 48% female	To ascertain the degree of approval amongst service users and staff for commonly used coercive measures in acute mental health care	Both service users and staff disapproved strongly of mechanical restraint and expressed a relative preference for compulsory intramuscular medication and seclusion. Male staff, older service users and staff who had implemented coercion reported greater approval of coercive measures.	Fair

Wilson, et al. (2017) UK	Interviews/Qualitative	Adult mental health inpatient	13 patients and 22 staff members	To feed key findings into a coproduced, evidence-based proactive care toolkit that eliminates reliance on restraint in mental health care	One main theme: Is restraint a necessary evil? Two subthemes: It never is very nice' and 'It's got to be done. . .it's a necessary evil'	Fair
Wilson, et al. (2018). UK	Interviews/ Qualititative	Adult mental health inpatient wards	Inpatients (n=13). 54% female. Age range 18-65. Staff members (n=22). 68% female. Age range from early 20s to late 50s.	To explore mental health patients' and staff members' suggestions for reducing physical restraint.	Findings centred on four overarching themes: improving communication and relationships between staff/patients; making staff-related changes; improving ward environments/spaces; and having more activities. However, concerns were raised around practicalities/feasibility of their implementation.	Good

Safety culture

Author, year, country	Study design	Setting	Participants	Aims/ objectives related to patient safety	Outcomes related to patient safety	Study quality
Ajalli, et al. (2018) Iran	Interviews/ Qualititative	Acute care wards (n=8)	Supervisors (n=2), head nurses (n=4) and registered nurses (n=13). 47% female. Median age 38.	To focus on nurses' opinions and experiences about patient safety in inpatient psychiatric wards	Nurses' experiences are reflected based on the main theme, including "intelligent care and protection from risk creators." Two themes and eight related categories were emerged including "vigilant care (warning of highrisk patients, curious attention, care of vulnerable patients, early intervention) and "close observing (confronting with dangerous concealment, access control, objective observation, continuous observation)".	Good
Bowers, Gournay & Duffy (2000) UK	Questionnaire/ Quantitative	A random stratified sample of NHS Trusts providing psychiatric inpatient services in England and Wales (n=27).	Directors of Nursing for these Trusts	To explore the observation policies and usage in psychiatric inpatient services	12% of the services had no written observation policy, and 38% had no clinical recording system. Only two policies studied used the same terminology for the different levels of intensity of constant observation (CO). There was variation across Trusts in who was qualified to undertake CO and whether agency staff were employed to do this. Gender of staff was a consideration for most Trusts when deciding who to allocate to CO. Most policies stated that patients should be given a rationale for CO. The most agreed upon reason for using CO was	Fair

					to reduce harm/suicide.	
Bowers, et al. (2006) UK	Questionnaire/ Qualitative	14 acute psychiatric wards and 3 psychiatric intensive care units across 3 hospital sites, within 1 NHS Trust	Staff from these wards (n=56). Ward Managers (n = 16), F Grade mental health nurses (n = 17), Occupational Therapists (n = 14) and Consultant Psychiatrists (n = 9).	To explore the impact of serious incidents on inpatient psychiatric wards	Staff reported feelings of shock, depression, demoralisation, loss, and grief after incidents, with periods of rumination, guilt and anxiety. Following incidents, levels of containment increased, along with the focus on risk assessment. Staff reported the following as making it difficult to emotionally process the incidents: the pace of ward life, a lack of external support, and management investigations. Patient responses to the incidents were rarely acknowledged.	Fair
Brennan, Flood & Bowers (2006) UK	Observation and action research/ Qualitative	Generic acute admission wards (n=2)	City Nurses within the wards (n=2). All staff and patients within the wards	To explore change implementation issues in psychiatric acute admission wards	Barriers to change were: limited staffing resources, the physical environment, insufficient beds, unclear hierarchical structure and multidisciplinary issues, over demanding ward managers, and anxiety about serious incidents.	Fair
Cowan, et al. (2018) Australia	Participant observations, focus groups and interviews/ Mixed methods	Acute mental health inpatient units (n=2)	Patients (n=233) Nurses (n=36)	To develop a guideline to support uniform structure and process in mental health nursing handover.	Data collected as part of that investigation contributed to the development of a handover guideline that incorporates the key components of structure, content, and leadership. The research indicated a link between these components, and further revealed the necessity to have other forums, such as supervision and clinical review, to ensure that handover serves its	Fair

					intended purpose in an efficient manner	
Cullen, Nath & Marcus (2010) USA	Semi-structured interviews/ Qualitative	Inpatient psychiatric units within urban teaching hospitals (n=2)	Staff (n=17). 53% female	To explore the typology of errors and precipitating factors in inpatient psychiatry	Preventive errors (such as falls, suicide) were mentioned the most (38% of errors mentioned). Provider factors were most commonly attributed to the precipitation of errors (74% of factors mentioned). Most of the broad typologies and precipitating factors echo those found in general medicine and surgery, whilst the specifics are unique to inpatient psychiatry.	Fair
Delaney & Johnson (2006) USA	Observation and semi-structured interviews/ Qualitative	Inpatient psychiatric units (n=2)	Staff (n=16; age range 22-50+) and patients (n=12; 50% female; age range 22-56)	To explore de-escalation processes and the skills of staff in inpatient psychiatric units to create a safe environment	Staff behaviours can create a safe milieu and positive culture. Important skills for maintaining safety were: awareness, attending, caring, and connecting.	Fair
Gabrielsson, et al. (2014) Sweden	Focus groups/Qualitative	The inpatient ward of a psychiatric clinic	Staff (n=26). 54% female.	To explore staff perceptions of inter-professional collaboration relating to challenging situations in psychiatric inpatient care.	Physicians were described as distant decision-makers, ward managers as suspicious supervisors, psychiatric nurses as mediating moderators, and nursing assistants as informed performers. Expectations of staff during challenging situations were: to talk to each other, control the situation, know the patient and set the stage. The common thread throughout all discussion was recognising knowledge of the patient as decision-making power in challenging situations.	Fair

Gerace, et al. (2018) Australia	Interviews/Qualitative	Acute psychiatric settings	13 nurses, 7 consumers	To investigate how empathy is developed and maintained when there is conflict between nurses and consumers, and the ways in which empathy can be used to achieve positive outcomes.	Antecedents of the empathy experience (MY role as a nurse - the orle of my nurse, What nurses and consumers brought to the situation, the sitution) Processes (Perspective taking as trying to understand); Intrapersonal outcomes (Feelings for the consumer); Interpersonal outcomes (being there, Empathic relationshop withstand conflict, Empathy inflences nurse-consumer satidfaction).	Fair
Gifford & Anderson (2010) Canada	Nominal group consensus technique/ Qualitative	Inpatient psychiatric mental health care facility	Healthcare nurses with inpatient nursing experience (n=16); 75% female; mean age 49	To identify staff attitudes to reporting assault in an inpatient psychiatric setting	Safety culture was the predominant factor in deciding whether to report an incident. The design of the reporting system, and the perceived effect on patients were also deemed important when making a reporting decision.	Fair
Happell & Koehn (2011) Australia	Descriptive/ Quantitative	Acute inpatient unit	Psychiatric nurses (n=123); 51% female; mean age 41 (range 20-62)	To investigate associations between burnout, job satisfaction and therapeutic optimism, and seclusion attitudes	A significant negative relationship was identified between optimism and seclusion attitudes. Staff attitudes influenced the likelihood of secluding patients. Staff with higher levels of therapeutic optimism and lower levels of emotional exhaustion are less likely to support the use of seclusion.	Fair

Haines, et al. (2017) UK	Cross-sectional/Quantitative	NHS psychiatric wards (n=60)	Staff (n=191). 50% female. Modal age group 25-34 (27%)	To identify the factors affecting perceived safety of staff on mental health inpatient wards	The factors that were found to increase perceptions of staff safety were: ward brightness, more patient beds, lower staff to patient ratios, less dayroom space and more urban views	Good
Higgins, et al. (2018) Australia	Interviews/Qualitative	Acute mental health wards (n=3)	Registered nurses (n=15). Age range 23-59	To explore the views of nursing staff regarding the factors that impact their ability to implement Safewards in acuteadult inpatient wards	Four themes arose: mixed views of Safewards; support from senior staff; understanding practice is just as important as individual skills training; and project teams need to recognise and acknowledge the local culture if it is to be changed	Fair
Ireland, et al. (2019) UK	Questionnaire/Quantitativ e	Male high secure forensic psychiatric facility	Staff (n=151; 61% male). Patients (n=62; 100% male). Age range 23-59	To examine the relationship between interpersonal factors and security incidents on a psychiatric ward	Perception of fair treatment was found to be a mediating link between dominant and hostile interpersonal styles, assertive control and victimisation incidents	Good
Johnson & Delaney (2006) USA	Descriptive/ Qualitative	Psychiatric inpatient unit	Staff (n=16); 56% female and patients (n=12); 50% female; mean age 33	To describe the context and conditions under which specific interventions were used to assist patients to regain control	The overarching theme was dimensions of “keeping the unit safe" that was split and interconnected between ideology, people, space, and time.	Fair
Jones, et al. (2010) UK	Semi-structured interviews/ Qualitative	Acute psychiatric hospital	Inpatients (n=60); 40% female; mean age 43 (19-81)	To explore the experiences of service users on acute inpatient psychiatric wards regarding safety and security	The majority of patients felt safe and supported by staff and other service users during their stay in hospital. However, safety incidents such as aggression, drug misuse or bullying negatively influenced this	Fair

					perspective, resulting in patients feeling unsafe.	
Jonker, et al. (2008) Netherlands	Cross-sectional/ Quantitative	Wards of a mental health institution (n=6)	Nurses (n=85); 68% female	To explore nurses' perceptions of the process of managing aggression and the determinants of the use of coercive interventions	Nurses felt in control of most aggression incidents and felt that they had social support from colleagues. Despite the high prevalence of aggressive incidents, staff did not feel that it was a large problem.	Fair
Kanerva, et al. (2015) Finland	Semi-structured interviews/ Qualitative	Psychiatric inpatient unit	Nurses (n=26); mean age 39 (range 23-60)	To describe the communication components that support patient safety	Communication that supports patient safety in psychiatric inpatient care was the main theme and overarched three sub-themes of fluent information transfer, open communication culture and being active in communication.	Fair
Kanerva, Lammintakanen & Kivinen (2016). Finland	Semi-structured/ Qualitative	Psychiatric hospital (n=2)	Nurses (n=26); 61.5% female; mean age 39 years (23-60)	To explore nursing staff's views of patient safety in inpatient care	Two themes were: experiences of safety (issues related to feelings about patient safety), and implementation of safe care (practical issues related to patient safety). Medication safety was deemed particularly important, whereas seclusion, restraint and suicide were barely mentioned. More emphasis was given to the skills staff felt they need to give safe care.	Fair
Koukia, et al. (2010) Greece	Questionnaire/Quantitativ e	Inpatient wards (n=14) in acute psychiatric hospitals (n=3)	Mental health nurses (n=164); 51% female; mean age 36.5 [3.5]	To investigate safety measures taken by mental health nurses and identify security policies in acute mental health wards	Standardised protocols did not exist across the hospitals. There was a lack of safety measures across the wards. Banned substances and search procedures varied greatly.	Fair

Kuosmanen, et al. (2013) Finland	Cross-sectional/Quantitative	Forensic hospital (n=2)	Staff (n=283); 51% female	To evaluate the current patient safety culture and identify potential improvements	Nearly two-thirds rated the patient safety level as excellent or very good. Teamwork within psychiatric units received the highest score (72% positive), and non-punitive response to errors received the lowest (26% positive).	
Kuosmanen, et al. (2019) Finland	Questionnaire/Quantitative	Forensic psychiatric hospitals (n=2)	Staff at the control hospital (n=84 baseline; n=77 follow-up). 54% female baseline; 62% female follow-up. Staff at the study hospital (n=199 baseline; n=207 follow-up). 50% female baseline; 52% female follow-up	To examine how the implementation of a patient safety incident reporting system impacts upon patient safety culture within a forensic psychiatric setting	Five patient safety culture dimensions showed a significant (p<0.05) increase in positive response rates (supervisor/manager expectations and actions regarding patient safety; handoffs and transitions; feedback and communication about errors; non-punitive response to error), whereas none were found for the control hospital.	Good
Langan & McDonald (2008) Ireland	Descriptive/ Quantitative	Acute psychiatric inpatient unit	Staff (n=27); mean age 35.7 [9.9]. Patients (n=35); 43% female; mean age 38.7 [10.0]	To identify the prevalence of placing patients in night attire, its clinical and demographic associations, and attitudes towards it	Patients were unhappy and uncomfortable in the night attire and suggested they should be able to wear what they want. Staff had a different view and felt only voluntary patients should be allowed to have their own clothes. Staff felt patients wearing night-attire helped with security and reducing absconding and self-harm.	Fair

Lavelle, et al. (2017) UK	Pre-post evaluation/Mixed methods	Psychiatric triage wards (n=2)	Staff (n=53). Mental health nurses (n=36); psychiatrists (n=6); healthcare assistants (n=9); activity co-ordinators (n=2)	To explore the impact of training on the knowledge, attitudes, and confidence levels of staff towards medical deterioration in psychiatric inpatients	Knowledge (p<0.001), confidence (p<0.001) and attitudes (p<0.02) towards managing medical deterioration significantly improved. Focus group themes were: improved confidence, understanding of effective communication, self-reflection, team working and sense of responsibility	Fair
Maguire, Daffern & Martin (2014) Australia	Semi-structured interviews/ Qualitative	Forensic hospital	Patients (n=12) Nurses (n=12); 58% female	To explore patient and staff perspectives of limit setting in a forensic hospital	Patients were unfamiliar with limit setting as a term and understood it as setting boundaries for behaviour. Staff didn't like the term as they perceived it to mean controlling the patients. Both suggested it was necessary to ensure safety. Overall, a therapeutic relationship and a consistent, empathetic, authoritative, and knowledgeable approach to limit setting was deemed important.	Fair
Mahoney, et al. (2012) USA	Pre-post questionnaire design/ Quantitative	Psychiatric Hospital	Staff: pre-intervention (n=108), post-intervention (n=143)	To explore how a programme aiming to improve team work and patient safety can be used effectively by describing the implementation process and identifying any team differences before and after implementation	The programme was implemented with success. The team attributes positively differed post-intervention (p<.01). This included team foundation, functioning, performance, skills and climate and atmosphere.	Poor

Martin & Daffern (2006) Australia	Questionnaire design/ Quantitative	Forensic psychiatric inpatient hospital	Staff (n=69); 46% female	To examine staff perceptions of personal safety and confidence	Clinicians reported feeling safe and confident in managing a violent incident. However, staff reported factors that affected their ability to feel confident including: knowledge, experience and skill; working as a team; and their use of intervention strategies.	Fair
Mezey, Hassell & Bartlett (2005) UK	Semi-structured interviews/ Qualitative	Single-sex and mixed-sex medium secure units (n=16)	Inpatients (n=31); 19% female. Staff (n=58); 53% female	To assess the extent to which women inpatients consider themselves to be safe and to whether these views are identified in staff	Patients in both types of unit experienced violence, both sexual and physical. However, women patients in the single-sex units experienced more intimidation, threats and physical abuse but less sexual and physical violence.	Fair
Millar & Sands (2013) Australia	Exploratory descriptive design/ Qualitative	Acute inpatient psychiatric unit	Inpatients (n=25) covering 500 handovers	To identify the frequency and type of risk information transferred at handover	Patient safety is compromised by the reduced or inadequate communication over risk. Verbal communication was the most common method for handover updates.	Good
Nathan, et al. (2007) UK	Prospective/ Quantitative	A medium secure forensic psychiatric hospital	Nurses on the women's ward that were followed-up (n=14); 71% female; mean age 38.1 (SD=10.00). Nurses on the men's ward that were followed-up (n=14); 93% male; mean age 37.7 (SD=9.8)	To explore gender differences in psychopathology in forensic mental health settings and how this may affect the risk of staff burnout	Nurses on the women's ward were found to score significantly higher in the emotional exhaustion component of burnout. Suggested inter-related factors that are key to the development of emotional exhaustion include: nature of the mental disorder may differ between genders, and men can display more aggressive behaviour whilst women display more relational aggression.	Fair

O'Brien & Cole (2004) Australia	Participatory action research/ Qualitative	Acute inpatient general hospital psychiatric facility	Patients, relatives, carers and nurses (n=42)	To explore the context and experiences of nurses, patients, and relatives in the close-observation area and to develop recommendations for clinical practice	Data revealed three main themes: design and environment (concern was raised about the lack of privacy and security), lack of activity and structured time, and nursing care (some patients indicated that they did not feel cared about).	Fair
O'Neill, et al. (2003) Ireland	Structured interviews/ Mixed methods	A psychiatric hospital	Inpatients. Length of Stay < 2 years (n=45); median age 29.3) Length of Stay > 2 years (n=43); Medium age 48.8, and key workers.	To describe the patient groups present in Irish forensic psychiatric beds and to ascertain unmet treatment and placement needs	Twenty (47%) of the long-stay group and eleven (24%) of those with shorter durations of stay were felt to be inappropriately placed. 30% of long-stay patients could be safely transferred to lower levels of security within six months and 63% within three years.	Fair
Pelto-Piri, et al. (2019) Sweden	Interviews/Qualitative	General psychiatric clinic (n=1); psychiatric addiction clinic (n=1); forensic psychiatric clinic (n=2)	Adult patients (n=17)	To explore patients' perceptions of feeling safe or unsafe in psychiatric inpatient care	Interview themes were: predictable and supportive services increase feelings of safety; communication and responsibility increase feelings of safety; powerlessness and unpleasant encounters reduce feelings of safety	
Qi, et al. (2014) China	Cross-sectional/ Quantitative	Psychiatric hospitals (n=2)	Nurses (n=705); 100% female; mean age 31.3 (SD=7.1)	To compare the level of work-related stress between female nurses working in psychiatric and general hospitals and explore associated individual and environmental factors	Psychiatric nurses were more likely to be exposed to workplace violence (23.5% vs. 80.5%, $p < .001$), and had greater levels of stress in the domains of working environment ($p < .001$) and patient care (accidents, patient-staff relationships, and the impact of patient suffering or death) ($p < .001$).	Good

Rees & Manthorpe (2010) UK	Semi-structured interviews/ Qualitative	Residential services (n=8)	Managers (n=13) and care workers (n=10).	To explore the impact of adult protection investigations on managers of residential services and staff	Service managers stated that the application of policy and practice can be both beneficial and detrimental to their services (including service disruption, stress for residents, staff and managers). Multi-agency collaboration, transparency, training, reflective practice, and effective supervision were given as influencing the implementation of adult protection policies.	Fair
Ryan (2007) Europe	Longitudinal/ Quantitative	Acute inpatient psychiatric centres	Staff (n=205)	To compare, across six European mental health services, the levels of occupational stress and burnout among mental health workers in acute psychiatric hospitals	There were no statistically significant differences between sites and teams regarding emotional exhaustion. Great Britain had the highest score for depersonalisation.	Fair
Salzmann-Erikson, et al. (2008) Sweden	Cross-sectional/ Qualitative	A PICU	Caregivers (n=18); 50% female; age range 23-56.	To describe which care activities are practiced by registered nurses and assistant nurses in the PICU.	Categories describing the fundamentals of the PICU were found: protests and refusal of treatment due to lack of capacity, escalating behaviours, and using coercive measures to manage violence.	Fair
Silvana, et al. (2012) Italy	Cross-sectional/ Qualitative	A psychiatric ward	Nurses (n=20)	To plan ergonomic improvement from preliminary results of a psychiatric ward case-study	Ergonomic issues were found: a locked and polluted environment (smoking indoors) and an unusual staff gender ratio (hospital management considers staff gender ratio a safety issue due to men's strength, allowing them to cope with aggressive behaviour). No mental health care programmes are available for nursing staff, not even after	Fair

					safety incidents.	
Sjöstrand, et al. (2015) Sweden	Cross-sectional/ Qualitative	General psychiatry, forensic psychiatry, and addiction psychiatry settings	Psychiatrists (n=8); 50% male; age range 30-68.	To explore the psychiatrists' ethical reasoning regarding involuntary psychiatric treatment.	Participants were focused on the consequences of involuntary treatment, balancing risk of damaging the therapeutic alliance against ensuring patients received the treatment they needed. Suicidal and psychotic patients were examples where involuntary care was seen as justified. However, it was also argued that risk of suicide might not be sufficient for justified involuntary care. Organisational factors were seen as sometimes resulting in compulsory treatment that could have been avoided.	Fair

Stead, et al. (2009) Australia	Pre-post intervention/ Quantitative	An inpatient mental health facility	Multidisciplinary staff	To evaluate the implementation of a staff educational program aimed at increasing team work and patient safety, addressing three outcomes: observed team behaviours; attitudes and opinions; and clinical performance and outcomes	Changes implemented included the restructuring of multidisciplinary meetings and the introduction of structured communication tools. There was also a significant improvement in aspects of patient safety culture (frequency of event reporting, and organisational learning) and knowledge, skills and attitudes scores increased by 6.8%. Seclusion rates also reduced.	Fair
Stein (2002) UK	Cross-sectional/ Qualitative	Mental health inpatient facilities	Clinical directors (n=25), medical directors, (n=22), managers (n=6), consultants (n=3), nurses (n=1), other (n=1)	To explore the development and effective use of tools to predict risk to patients and public following discharge into the community	60.3% of sites used a discharge checklist, so development of risk assessment tools is varied at Trust level. Developments seem to be stimulated by clinical governance in the absence of an overarching national strategy. It seems that information technology for risk data collection is not widely used, instead using paper and informal communication.	Fair
Stübner, Groß & Nedopil (2006) Germany	Cross-sectional/ Quantitative	Forensic institutions (n=8)	Heads of department or their locum tenens	To investigate risk factors for incidents during hospitalization and criteria allowing easing of regulations and confinement	Among the risk factors, 87% of the terms related to patient characteristics, while among the protective factors the proportion was 77.5%. Good relations with the therapeutic team accounted for 14.6% of the criteria for easing restrictions, while poor relations accounted for less than 4% of the risk factors.	Fair

Truea, et al. (2017) USA	Interviews/Qualitative	7 Veteran's Health Administration inpatient psychiatric units	Staff (n=20) working within 7 VHA hospitals including psychiatrists (n=7) and other management/staff (N=13)	To identify risk factors and protective factors, along with the mechanisms by which they relate to patient safety events in this setting in order to inform interventions geared toward improving quality of care for persons with serious mental illness	Two broad thematic domains related to patient safety: risks – threats to patient safety events at the system-, provider-, and patient-level; and protective factors associated with psychiatric inpatient safety—processes and infrastructure in the treatment environment that, when in place, thwart or mitigate these risks.	Good
Vahidi, et al. (2018) Iran	Interviews/Qualitative	Psychiatric referral center. The center is comprised of separate male and female inpatient units that have similar conditions and regulations.	Patients at the point of discharge (n=7); Staff (n=19)	To generate more understanding of the nature of therapeutic relationships in Iranian psychiatric inpatient settings and how these relationships may be enhanced to improve quality and safety of care.	Facilitators of a safe environment included “supportive relationship with patients” and “improving patient capacity for self-efficacy/self-control”. Inhibitors of a safe environment included “detachment from patients” and “domination over patients”.	Good
Vandewalle, et al. (2018) Belgium	Questionnnaires/Quantitative	173 psychiatric wards within 37 hospitals in Belgium	705 nurses	To investigate the demographic and contextual factors that influence the willingness of nurses on psychiatric wards to share power and responsibility with patients concerning patient safety.	The willingness of psychiatric nurses to accept a new role is positively associated with being male, older, employed on an open ward, and perceiving personal competence and support to facilitate patient participation. The ‘acceptance of a new role’ component does not include items about specific patient safety situations and conditions, such as aggression, self-harm, or suicidal ideation. The receptivity of nurses to patients’	Good

					factual and challenging questions is negatively associated with nurses' willingness to accept a new role.	
Vlayen, et al. (2012) Belgium	Cross-sectional / Quantitative	Acute (n=90), psychiatric (n=42) and long-term (n=11) care hospitals	Staff in acute (n=47,635), psychiatric (n=6341) and long-term care hospitals (n=1249)	To explore patient safety culture in Belgian hospitals and the underlying safety culture dimensions	Strengths of patient safety culture were: team work, supervisor/ manager expectations and actions promoting safety, and organisational learning. Handoffs and transitions, staffing, management support for patient safety, non-punitive response to error and teamwork across units could be improved. Positive dimension scores were higher for psychiatric and long-term care hospitals than for acute hospitals, suggesting that patient safety is more encouraged within these settings.	Good
Ward (2013) Australia	Cross-sectional / Qualitative	An inpatient mental health care facility	Nurses (n=13); 100% female	To investigate nursing practices, the nurse-patient relationship, violence, and aggression in acute inpatient mental health care	Some workplace stressors included poor staffing skill matrix, complex patient diagnoses, and limited workspace design. Effective communication was seen as essential to prevent violence. Coping mechanisms included debriefing with other colleagues.	Good

Wood & Pistrang (2004) UK	Cross-sectional / Qualitative	An acute inpatient psychiatric unit	Patients (n=9); nursing staff (n=7)	To explore the experiences of safety and threat from the perspective of psychiatric inpatients	Ten themes relating to factors that influenced feelings safety were grouped into three clusters—patient interactions, staff behaviour and attitudes, and non-consensual treatment. Patients expressed feeling vulnerable and helpless.	Fair
Woods (2013) Canada	Cross-sectional / Qualitative	Inpatient mental health units (n=7) and a forensic unit	Registered Psychiatric Nurses (n=33); Registered Nurses (n=2); Licensed Practical Nurse (n=1); Special Care Aides (n=7); Social Workers (n=2); Student Nurse (n=1); and other (n=2). Total (n=48)	To identify and describe current risk assessment and management approaches used in adult inpatient mental health and forensic units	Key issues were discussed as important: the pitfalls of relying on clinical judgement alone; considering risk as a wider concept; risk management being reactive; lack of education and training, and client involvement.	Fair

Harm to self

Author, year, country	Study design	Setting	Participants	Aims/ objectives related to patient safety	Outcomes related to patient safety	Study quality
Awenat, et al. (2018) England	Interviews/Qualitative	NHS mental health service in Northern England with five acute adult psychiatric wards	Patients in five acute adult psychiatric wards (n=20), aged 22-65	To investigate suicidal in-patients' views and expectations of a novel ward-based suicide-focussed psychological therapy intervention	Two themes: Theme 1 - 'A therapy that works', epitomised participants' perceptions of the influences and necessary components for effective ward-based suicide-focused psychological therapy. Theme 2 - 'Concerns about in-patient suicide-focused therapy' depicted participants' fears about engaging with therapy.	Good

Booth, et al. (2014) Ireland	Questionnaire/Quantitative	A psychiatric hospital	Patients (n=114). 81% female. Mean age 35	To evaluate the effectiveness of a brief intervention for decreasing self-harm and increasing distress tolerance.	There was a decrease in the frequency of self-harm incidents from pre-intervention (M=13.68, SD=21.81) to post-intervention (M=4.50, SD=11.01) (p=.02, N=48) and from pre-intervention to 3 months post-intervention (M=3.62, SD=11.33) (p=.01, N=48). There was an increase in distress tolerance from pre-intervention (M=6.32, SD=2.35) to post-intervention (M=10.36, SD=3.68) (p=.00, N=32) and from pre-intervention to 3 months post-intervention (M=9.72, SD=4.33) (p=.00, N=32). There was a decrease in inpatient days from pre-intervention (M=39.90, SD=33.25) to post-intervention (M=23.09, SD=40.56) (p=0.01, N=65), from pre-intervention to 3 months post-intervention (M=8.78, SD=25.40) (p=.00, N=65) and from post-intervention to 3 months post-intervention (p=.00, N=65).	Fair
Bowers, et al. (2008) UK	Questionnaire/ Quantitative	Acute psychiatric wards (n=136) in 67 hospitals within 26 National Health Service (NHS) Trusts	Staff and patients within the units	To explore the relationship between special observation and self-harm rates in acute psychiatric wards	Constant special observation was not associated with self-harm, but intermittent observation (OR=0.82 (0.78–0.87); p<0.001), levels of qualified nursing staff (OR=0.94 (0.90–0.98); p<0.01), and more intense programmes of patient activities (OR=0.53 (0.38–0.75); p<0.001) were associated with reduced self-harm.	Good

Brown & Beail (2009) UK	Semi-structured interviews/ Qualitative	A secure service for people with intellectual disabilities and challenging behaviour	Residents (n=9). 56% male.	To explore the experiences of self-harm, and interventions for this self-harm, among residents with intellectual disabilities within secure accommodation	Self-harm management interventions imposed by the service were mostly perceived as controlling, punitive and evoked strong negative reactions from residents.	Good
Caspi (2014) USA	Observation and semi-structured interviews/ Qualitative	Special dementia care units within an assisted living residence (n=2)	Residents (n=12). 92% female. Mean age 81 (range 75-86).	To explore self-neglect and other behaviours when residents with dementia are unsupervised within assisted living residences	There were 158 incidents of negative behaviour expression, emotional states and self-neglect. 56% of these were self-neglect incidents. 97% of these took place during times when residents were not engaged in structured activity. The incidents included a wide variety of safety risks, including falls, attempted absconding and risk of aggressive interactions.	Good
Cleary, et al. (1999) Australia	Interviews/ Qualitative	Acute inpatient psychiatric wards (n=4) within a psychiatric hospital	Clinical registered nurses (n=10)	To explore the role of registered nurse in the care of patients on special observation	9 themes were developed to highlight the topics discussed by nurses when considering their role in the special observation process: safety; therapeutic relationships; supporting patients and carers; consequences of special observation for nurses; continuity of care concerns; peer support; suicide indicators; responsibilities and rights; and nurses, doctors and the hospital hierarchy.	Fair

Davis, Williams & Hays (2002) USA	Questionnaire/ Quantitative	An acute-care psychiatric facility	Patients (n=135). Mean age 36. 70% female	To explore the views of suicidal psychiatric inpatients concerning no-suicide agreements	Patients had an overall positive view of no-suicide agreements, relating to the therapeutic features, coerciveness and detachment of the process. 92% of participants agreed that they had confidence in their ability to keep to their commitment.	Fair
de Jonghe-Rouleau, Pot & de Jonghe (2005) Netherlands	Questionnaire/ Quantitative	Psycho-geriatric wards of a large nursing home (n=3)	Residents (n=110). 77% female. Mean age 83 (range 67–105)	To explore self-injurious behaviour (SIB) in nursing home residents with dementia	SIB was reported in 22% of patients. Pinching, scratching and banging one's fist against objects were the most commonly reported behaviours (8% of patients). SIB was associated with: prescribed psychotropics (OR=5.62), immobility (OR=3.71) and restraint (OR=6.19).	Fair
Drew (1999) USA	Questionnaire/ Quantitative	Psychiatric hospitals (n=84)	Staff	To explore the use of no-suicide contracts in psychiatric hospitals	The most common suicide prevention intervention used by the hospitals was limiting access to objects used for self-harm (100%). 79% of the hospitals used no-suicide contracts. Contracts were mostly drawn up after suicidal ideation was expressed (83% of hospitals that used no-suicide contracts) by nurses. They were mostly verbal (74%). 53% of respondents estimated suicide behaviour frequency of 10+ incidents a year.	Fair
Ellis, et al. (2012) USA	Open-trial case-series/ Quantitative	A private psychiatric hospital	Patients (n=20). 80% female. Mean age 37 (range 21-55)	To evaluate a program designed to reduce the risk of suicide in inpatient psychiatric hospitals	From pre-post treatment, there were significant reductions (effect sizes= > .80) in depression, hopelessness, suicide cognitions, and suicidal ideation, as well as improvement on contributory factors of	Fair

					suicidality. The factors with the highest effect sizes were: depressive symptoms, psychological pain, and self-hate.	
Ellis, et al. (2015) USA	Controlled trial/ Quantitative	A private psychiatric hospital	Patients (n=52). 69% female. Mean age 33 (range 18-68)	To evaluate the effectiveness of a program designed to reduce the risk of suicide in inpatient psychiatric hospitals above and beyond that of intensive, psychotherapeutic, milieu-based treatment	The program group showed greater improvement on measures of suicidal ideation and suicidal cognition (effect sizes= > .80).	Fair
Esposito-Smythers, McClung & Fairlie (2006) USA	Descriptive/ Quantitative	An acute adolescent psychiatric inpatient unit	Patients (n=250). 60% female. Mean age 15 (range 12-18)	To explore participant perceptions of a suicide prevention group for psychiatrically hospitalised patients	94% of participants reported that they learnt something from the group that would prevent them attempting suicide in the future. The most helpful part of the group was reported to be the Reasons to Live List (32% of participants stated this was most helpful). The least helpful part was reported to be the Safety List (30%).	Fair
Gibson, et al. (2014) Ireland	Controlled trial/ Quantitative	Psychiatric hospital	Psychiatric inpatients (n=103). (range 18-60); Living through distress programme (LTD), (n=82); 79% female; mean age 38 and waiting list (TAU) (n=21); 57% female; mean age 32	To examine whether additional DBT skills training improved outcomes including self-harm compared to TAU	Self-harm significantly reduced in the LTD group compared with TAU (49% of the LTD group had a reduction in self-harm of 75% or greater) and this was maintained at 3 month follow-up.	Fair
Gough & Hawkins (2000) UK	Questionnaire/ Quantitative	Forensic psychiatric hospital	Clinical staff (n=77)	To explore staff attitudes regarding self-harm in psychiatric patients	Staff attitudes towards self-harm varied. Staff felt self-harm was used to communicate with others about how distressed they are (M=2.9; SD = 0.8). Some staff felt self-harm wasted staff time (M=1.9;	Poor

					SD= 1.0). Overall, staff felt more training on self-harm was needed.	
Hill, et al. (2017) USA	Questionnaires and interview/ Quantitative	Acute-care psychiatric hospital	Adolescent psychiatric inpatients (n=142). 59.9% female. Mean age 14.73 (range 12–17)	To better understand the role of interrupted and aborted attempts in suicide risk assessment.	Results suggest that interrupted and aborted suicide attempts are associated with the frequency of actual suicide attempts, controlling for suicidal ideation and depressive symptoms.	Fair
Holth, et al. (2018) Norway	Questionnaire/Mixed methods	Inpatient wards within mental health centres (n=32) and psychiatric hospitals (n=29)	Department managers (n=61)	To assess extensive psychiatric hospitalisation due to self-harm, associated severe medical sequelae and health service collaboration issues	There were 427 cases of extensive hospitalisation due to self-harm. In 109 cases, there were severe medical consequences, including five deaths. Collaboration issues were recorded in 122 cases (including diagnosis, treatment and resource disagreements).	Fair
Inoue, et al. (2017) Japan	Questionnaire/Quantitative	Psychiatric settings within hospitals (n=97)	Staff	To investigate inpatient suicides and risk factors in psychiatric settings	131 inpatient suicides were recorded in inpatient settings. Hanging was the most common suicide method (58% of suicides) and most happened on the ward (47%) between 8.00 and 16.00 (43%). The most comon risk factor was previous suicide attempt or self-harm (46%). The most common psychiatric diagnosis was schizophrenia (39%).	Fair

James, et al. (2017) UK	Survey and interview/ Mixed methods	Acute psychiatric wards (n=31)	Phase I: Inpatient mental health practitioners (n=387). 57% female. Age range 20-60 or over. Phase II: Inpatient mental health practitioners (n=18). 72% female. Age range 20-49.	To explore nursing practitioners' perspectives and experiences of harm reduction practices for self-harm on mental health wards.	Practitioners who had implemented the approach reported positive outcomes including a reduction in incidence and severity of self-harm and a perceived increase in empowerment of service users. Practitioners with no experience of using harm reduction were concerned that self-harm would increase in severity, and were unsure how to assess and manage risk in people under a harm reduction care plan. Some fundamentally disagreed with the principle of harm reduction for self-harm because it challenged their core beliefs about the morality of self-harm, or the ethical and potential legal ramifications of allowing individuals to harm themselves.	Good
Kool, et al. (2014) Netherlands	Quasi-experimental pre-post-test/ Quantitative	Mental health centres (n=8) and a forensic-psychiatric centre (n=1)	Staff (n=178); mean age 38	To measure the effects of a self-harm reduction programme on attitudes towards self-harm patients; self-efficacy in managing self-harm patients; and the distancing of self-harm patients and treatment staff	After the programme, staff were significantly more likely to have perceived confidence in assessing and referring self-harm patients; managing them effectively; have an emphatic approach; and be able to cope effectively with regulation in relation to self-harm guidance.	Fair

Lindgren, Aminoff & Graneheim (2015) Sweden	Participant observational and interview study/ Qualitative	Psychiatric hospital	Women inpatients (n=6); median age 23.5 (range 21-37 years)	To describe features of everyday life of patients who self-harm	The main factor associated with everyday life was being surrounded by the disorder. This encompassed residing in a confusing environment, being confined to routines and rules around safety that lack consistency. Loneliness was an issue. Staff spent limited time with the patients.	Fair
Lundegaard Mattson & Binder (2012) Norway	Cross-sectional/ Qualitative	A psychiatric hospital	Healthcare workers (n=8)	To explore the perceptions, emotions and actions of healthcare staff when working with patients who self-harm	Several themes emerged, detailing: the frustration at having to use coercive interventions; the process of change from coercion to alliance; experiences of useful management strategies; and the distinction between self-harm and suicide attempt. On a structural level, a cap was put on the number of patients diagnosed with Emotionally Unstable Personality Disorder, and staff felt that this made it easier to work with patients, reducing frequency of self-injury. Management also became more involved in supporting staff training to reduce use of coercion.	Good

O'Donovan (2007) Ireland	Semi-structured interviews/ Qualitative	Acute psychiatric inpatient units (n=2)	Psychiatric nurses (n=8); 75% female (age range 25-55)	To explore the practices of psychiatric nurses relating to people who self-harm, but are not suicidal	Participants emphasized preventing self-harm and providing a physically safe environment. The primary methods for ensuring a service user's safety were: removal of sharp objects; and requesting that service users stay in their night clothes. Participants reported spending 15 to 90 min a day with each service user in therapeutic interaction. This was then used to inform staff of the potential for self-harm, to assess mood, and to inform future care.	Fair
Pfeiffer, et al. (2019) USA	Questionnaire/Quantitative	Psychiatry units of two midwestern facilities.	70 adult psychiatric inpatients	To assess the acceptability, feasibility, and fidelity of a peer specialist intervention titled Peers for Valued Living (PREVAIL) to reduce suicide risk, incorporating components of motivational interviewing and psychotherapies targeting suicide risk into recovery-based peer support.	Those in the PREVAIL arm completed an average of 6.1 (SD 5.0) peer sessions over the course of 12 weeks. Fidelity was rated for 20 peer support sessions, and 85% of the peer specialist sessions demonstrated adequate fidelity to administering a conversation tool regarding hope, belongingness, or safety, and 72.5% of general support skills (e.g., validation) were performed with adequate fidelity. Participants' qualitative responses (n 23) were highly positive regarding peer specialists' ability to relate, listen, and advise and to provide support specifically during discussions about suicide.	Fair

Sansone, McLean & Wiederman (2008) USA	Cross-sectional/ Quantitative	A community hospital	Inpatients (N=120). 61% female; mean age 38.69, SD=11.74 (range 18-74)	To explore, in a sample of psychiatric inpatients, the relationship between self-sabotaging behaviours and borderline personality disorder	76 respondents (63.3%) reported engaging in medically self-sabotaging behaviour, with the average number of different behaviours of this type being 4.11 (SD = 3.93). The most commonly endorsed behaviours (endorsed by around a quarter of participants) were: damaging self on purpose and seeking medical treatment; not going for medical treatment; to purposefully hurt self; not taking a prescribed medication; and involvement in dangerous situations.	Fair
Sandy (2016) United Kingdom	Interviews/Qualitative	Learning disability service in the west of England. Service comprises of 7 locked clinical areas with six registered nurses working in each area	Registered nurses (n=35)	To explore nurses' knowledge and understanding of the use of observation on patients who self-harm in a learning disability service in the United Kingdom.	Three superordinate themes 1) observation: its meaning, 2) observation: does it prevent self-harm? 3) Observation: making it work.	Good

Shaw & Sandy (2016) UK	Mixed methods/Qualitative	A large forensic mental health unit, containing medium and low secure facilities.	Mental health nurses (n=61), aged 25-56	To report the attitudes of nurses toward user who self-harm in secure environments, and propose educational recommendations	One super-ordinate theme (attitudes to self-harm), two second order themes had several subthemes. Positive attitdues included: need for training, understanding of self-harm, unconditional acceptabce, partnership working optimism and provision of choice activities. Negative attitudes included rigid authorative apporach, refusal to undertake training, blanket approach and insensitive responses.	Good
Sjöström, Hetta & Waern (2012) Sweden	Prospective cross-sectional/ Quantitative	Psychiatric units	Inpatients who attended follow-up interview (n=98)	To investigate whether low Sense of Coherence (SOC) is a predictor of suicidality and of risk of a repeat suicide attempt	2 months after a suicide attempt, low baseline SOC was significantly associated with an eightfold increase in risk of high suicidality (*P* = <0.001). Low SOC score at baseline was also associated with repeat attempts within 3 years (*P* = 0.038), but was not an independent predictor when other mental health symptoms were taken into account.	Good
Sun, et al. (2005) Taiwan	Cross-sectional/ Qualitative	Psychiatric admission wards	Patients (n=15); 60% female; age range 16-47. Psychiatric nurses (n=15); 100% female; age range 21-49.	To develop a suicide care theory that could help nurses to improve suicide prevention and care	In developing the theory, four categories were highlighted relating to interaction strategies: holistic assessment of people who are suicidal; protection; basic care; and advanced care.	Fair

Sun, et al. (2006) Taiwan	Cross-sectional/ Qualitative	Acute psychiatric wards and a psychiatric stress ward	Patients (n=15); 60% female; age range 16-47. Psychiatric nurses (n=15); 100% female; age range 21-49.	To: (1) explore and examine psychiatric nurses' and patients' perceptions of the care offered to patients with suicidal ideations on psychiatric wards, and (2) develop a nursing theory to guide the care of patients at risk of suicide	Fifteen categories were generated, relating to contexts and intervening conditions of care for suicidal patients. These categories centred on the therapeutic relationship, staff attitudes, blame, autonomy, staff powerlessness due to lack of training, and time constraints.	Fair
Swogger, Van Orden & Conner (2014) USA	Cross-sectional/ Quantitative	Acute inpatient hospitals (n=3)	Patients (n=892); 58% male; mean age 29.9 (SD=6.2) age range 18-40	To study the relationship of outwardly-directed aggression to suicidal ideation and attempts in psychiatric inpatients	Outwardly-directed aggression was associated with suicide attempts, but not ideation. It was also associated with planned, but not unplanned, suicide attempts.	Fair
Takahashi, et al. (2011) Japan	Cross-sectional/ Quantitative	Psychiatric medical institutions (n=8)	Nurses (n=531); 63% female; mean age 41.9 (SD=12.3)	To investigate issues related to staff experiences of patient suicide in mental health nursing	55% of participants had experienced patient suicide. 13.7% of participants were at a high risk of post-traumatic stress disorder. However, only 15.8% stated that they had access to post-suicide mental health care programmes. There was also low attendance at in-hospital seminars on suicide prevention or mental health care for nurses (26.4% and 12.8% respectively).	Fair

Thomas & Haslam (2017) UK	Interviews/Qualitative	Mental health inpatient settings	Staff (n=10). 80% female. Age range 23-31	To explore the experience of mental health staff of self-harm prevention and distress management	Interview themes were: characteristics of the inpatient environment, experiences of distress and urges to self-harm in the inpatient environment, when the inpatient environment does not provide alternative means of managing distress, ways the inpatient environment does provide alternative means of managing distress, and factors that influnece patient responses to inpatient environment and interventions	Fair
Tofthagen, Talseth & Fagerström (2014) Norway	Cross-sectional/ Qualitative	Psychiatric clinics (n=4)	Mental health nurses (n=15); 87% female	To explore the experiences of mental health nurses in caring for inpatients who self-harm	Two main categories were identified: challenging and collaborative nurse-patient relationships, and promoting well-being through nursing interventions. Participants sought to understand the self-harm behaviour, its triggers and signs in a person-centred way, helping patients learn to be aware of their feelings/behaviours and learn coping strategies.	Fair

Vandewalle, et al. (2019) Belgium	Interviews/ Qualititative	Closed ward in psychiatric hospital	Nurses (n=5). Age range 25- >55.	To uncover and understand the actions and aims of nurses in psychiatric hospitals during their interactions with patients experiencing suicidal ideation.	The findings show that nurses' actions and aims in their interactions with patients experiencing suicidal ideation are captured in the core element 'promoting and preserving safety and a life-oriented perspective'. This core element represents the three interconnected elements 'managing the risk of suicide', 'guiding patients away from suicidal ideation', and 'searching for balance in the minefield'.	Good
Vråle & Steen (2005) Norway	Cross-sectional/ Qualitative	Acute psychiatric wards	Nurses (n=5); 60% female	To explore the dynamics of performing constant observation of suicidal patients	The process of constant observation seems to follow organizing phases, from assessment to transitioning out of the process. The process requires a balance between the need for control and keeping the patient from self-harm, structure and flexibility, as well as maintaining a therapeutic relationship.	Fair
Weber (2002) USA	Cross-sectional/ Qualitative	A locked psychiatric hospital	Patients (n=9); 100% female	To explore how self-abusing women in a state psychiatric hospital defined self-abuse	Four themes were identified: the need to be listened to and receive help; specific triggers for self-abuse (noise on the ward, especially screaming and profanity); the causes of self-abuse; and how to stop the self-abuse (someone talking to them during times of crisis, and staff providing	Fair

comfort or distraction).

Safety of the Physical Environment

Author, year, country	Study design	Setting	Participants	Aims/ objectives related to patient safety	Outcomes related to patient safety	Study quality
Bayramzadeh (2016) USA	Focus groups/Qualitative (retrospecitve audit not reported here)	Institute for mental health in the southeast region of the USA. 81-beds patients admitted for substance and alcohol abuse, adult psychiatry, child and adolescent patients, and geriatric patients	Staff (n=9)	To explore if incident reports and staff perceptions confirm the differences in safety levels among different areas in the facility and if specific types of safety incidents occur more frequently in specific areas of the facility?	Participatns indicated that locations designated as Safety Level 4 (patient bedrooms and bathrooms) were the most problematic, followed by locations designated as Safety Level 3 (lounges and activity rooms).	Poor
Bellantonio, et al. (2008) USA	Randomized controlled trial/ Quantitative	Dementia-specific assisted living facilities (n=2)	Residents of the facilities with dementia (control group, n=52; intervention group, n=48)	To investigate the effects of a multidisciplinary team intervention on unexpected transitions from assisted living in people with dementia	Falls were the most frequent reason for unanticipated transition (n=40). The intervention reduced the risk of unanticipated transitions by 13%, but this was not statistically significant (p=0.67). The intervention reduced the risk of death by 63%, but this was not statistically significant (p=0.08).	Good
Bowers & Crowder (2012) UK	Questionnaire/ Quantitative	Acute psychiatric wards (n=32)	All staff and patients within the units	To explore the relationship between staffing numbers and adverse incidents on the wards of psychiatric hospitals	Lower qualified nurse staffing levels were associated with higher conflict (Incident Rate Ratio (IRR) =1.03)) and containment (IRR=1.03) rates.	Good
Bowers, et al. (2010) UK	Questionnaire/ Quantitative	Acute psychiatric wards (n=136) in 67 hospitals within 26 National Health Service (NHS) Trusts	Staff (n=638; 62% female; modal age group 25-34), patients (n=393; 52% female; modal age group 35-44) and visitors (n=168; 50% female; modal age group 35-44) within the units	To explore the acceptability of door locking to staff, patients and visitors within acute psychiatric wards	Five factors were found to relate to the acceptability of door locking: adverse effects, staff benefits, patient safety benefits, patient comforts and cold milieu. Patients felt more negatively about door locking than staff.	Fair

Chandler (2008) USA	Interviews/ Qualitative	An inpatient psychiatric unit within a community hospital	Staff (n=10)	To explore the experience of staff when moving from traditional care to trauma-informed care within inpatient psychiatric units	The transition was described by staff in the context of creating a culture of safety. The overarching theme was of transferring control from staff to patients, and this included: changed perspectives, collaborative patient–staff relationships, the implementation of safety protocols, and the prescription of individualized evidence-based educational resources.	Fair
Cowman & Bowers (2008) UK & Ireland	Comparative questionnaire/ Quantitative	Acute psychiatric wards in England (n=87) and acute admission psychiatric wards in Ireland (n=37)	Staff	To explore safety and security measures in acute psychiatric wards in England and Ireland	Irish wards were found to have higher levels of security and safety restrictions. Irish wards are more likely to ban items, restrict access to areas/items, routinely search patients, have access to security guards at all times, and have staff carry personal alarms. Door locking procedures were similar, with 29% of English wards and 28% of Irish wards always keeping doors locked.	Fair

Curtis, et al. (2013) UK	Unstructured interviews/ Qualitative	An inpatient mental health care facility	Staff (Phase 1 n=23; Phase 2 n=8; Phase 3 n=26)	To explore the views of staff concerning technical safety and therapy in the design of an inpatient mental health facility	Participants mentioned how responsibility for technical safety was being invested in the physical infrastructure. Surveillance and observation were also important. Staff felt that relying on technical safety measures meant shirking responsibility for risks they should manage. They had concerns about how focusing on technical safety might conflict with a therapeutic approach.	Fair
Dreyfus, Phillipson & Fleming (2018) Australia	Focus groups/Qualitative	Aged care facilites for individuals with dementia	Staff (n=25) and family members (n=6)	To explore attitudes of staff and family members to fences in dementia care settings	Focus group themes were: fences as a necessary evil, tension between physical and emotional safety, pressure on staff to keep residents safe, fences as a threat to physical safety, the positive impact of fences on emotional safety and wellbeing. There were also ideas for mitigating the negative effects of fences on residents' wellbeing	Fair

Fletcher, et al. (2019) Australia	Facilitated forums/ Mixed methods	Acute mental health hospital wards	Patients (n=9). 56% male. Mean age 44 (range 29-62). Carers (n=9). 89% female. Mean age 61 (range 46-73). Staff (n=17). 65% female. Mean age 40 (range 23-56).	To describe the research process and findings from stakeholder facilitated forums that revised and approved a set of evidence informed recommendations, to improve the least restrictive practices of acute mental health wards within the context of a locked doors policy in Queensland, Australia.	All participants endorsed the recommendation that acute wards should take a recovery-oriented approach. This was discussed at length in every forum, with many of the recommendations in the Recovery Orientation category rated highly by participants. Reducing boredom and increasing availability of peer support workers were considered key to achieving this.	Fair
Gebhardt & Steinert (1999) Germany	Pre-post questionnaire/ Quantitative	Acute general psychiatry wards (n=4) within a psychiatric hospital	Patients (n=183). 55% female. Mean age (Time 1=38; Time 2=38; Time 3=36). Staff (n=162)	To explore the effects of an equal distribution of severely disturbed patients on social climate, aggressive behaviour, and sexual molestation in acute wards	A significant improvement of ward atmosphere (Rao's R=1.654, P=0.037) and a reduction of aggressive behaviour ($_{x}2$>16, df=2, P<0.001) was found after structural changes were implemented, whereas the impact on sexual molestation could not be determined as these events were rare at baseline.	Fair
Haglund & von Essen (2005) Sweden	Semi-structured interviews/ Qualitative	Psychiatric inpatient unit	Voluntarily admitted inpatients (n=20); median age 43 years (range 19-87)	To describe patient perspectives of advantages and disadvantages of being cared for on a unit that has locked doors	Advantages included protecting patients and staff from external factors, providing patients with security and efficient care, and providing staff with a sense of control. Disadvantages included: making patients feel confined, making patients dependent on staff, and making patients feel worse emotionally.	Poor

Hunt, et al. (2012) UK	Descriptive/ Quantitative	Psychiatric inpatient wards	Inpatient suicides who died by hanging between 1999-2007 (n=344); median age 39 (range 17-85); 63% male	To establish the principal ligatures and ligature points on the ward and whether there are trends over time	During 1999-2007, a quarter of inpatients who died by hanging, did so within a week of admission (25%); half had been on a high or medium observation level (49%). A third of the deaths were thought to be preventable (33%). The door, hooks, handles and windows were collectively the most common ligature points (59%). Belts, sheets or towels accounted for almost two-thirds of the ligatures (61%).	Fair
Kalagi, et al. (2018) Germany	Interviews/ Qualititative	Acute psychiatric wards	Psychiatrists (n=15). 60% female. Mean age 35.3 (range 28–54). Psychiatric nurses (n=15). 60% female. Mean age 35.2 (range 24–63). Patients (n=15). 80% male. Mean age 38.9 (range 20–60).	To assess the opinions and values of relevant stakeholders with regard to the requirements for implementing open wards in psychiatric hospitals.	The interviewees identified conceptual, personnel and spatial requirements necessary for an open door policy. Observation and door watch concepts are judged to be essential for open wards, and patients appreciate the therapeutic value they hold. However, nurses find the door watch problematic. All groups suggest seclusion or small locked divisions as a possible way of handling agitated patients. All stakeholders agree that such concepts can only succeed if sufficient, qualified staff is available. They also agree that freedom of movement is a key element in the management of acutely ill patients, which can be achieved with an open door policy. Finally, the interviewees suggested removing the door from direct view to prevent	Good

					absconding.	
Kulkarni, et al. (2014) Australia	Descriptive/Quantitative	Psychiatric ward (n=2)	Inpatients (n=65) and staff (n=20)	To evaluate the impact of a female-only area within a mixed-gender inpatient psychiatry service on female experiences of patient safety and care	There were significantly more patient safety incidents within the mixed-gender inpatient ward compared to the female only area. Specifically, female patients and staff perceived female-only wards as safer.	Fair
Muir-Cochrane, et al. (2012) UK	Semi-structured interviews/ Qualitative	Acute psychiatric unit	Participants (n=35); 43% female; mean age 43 years. Inpatients (n=15); visitors (n=6); nurses (n=14)	To explore perceptions of the acceptability of locking doors on inpatient wards	Several themes found that acknowledge the importance of locking doors on inpatient wards. Generally, all participants felt door locking helped reduce absconding. Staff felt guilt and fear of being blamed for the patient absconding, whereas patients expressed depression and low self-esteem when doors were locked.	Fair
Simpson, et al. (2011) UK	Cross-sectional/ Quantitative	Acute psychiatric wards (n=136)	Exit security staff and qualified nurses	To investigate the relationship between rates of drug/alcohol use on acute psychiatric wards and exit security measures	Daily rates of alcohol/drug use by wards are likely to be skewed, as few wards reported high levels. Door locking and security measures do not seem to be significantly related to alcohol use (r=-0.035, p=0.690) or other substance use (r = 0.108, p = 0.216).	Fair

Stolker, Nijman & Zwanikken (2006) Netherlands	Prospective/ Quantitative	A locked ward of a psychiatric hospital	Patients (n=54); 70% male; mean age 36, age range 18-58.	To explore whether residing in single- versus multiple-bed rooms in a psychiatric ward influenced psychiatric patients' views regarding seclusion	Findings suggest that seclusion is perceived as a less negative experience after having resided in a multiple-bed room before being secluded, t (30) = 3.4, P < .05, compared to patients residing in a single bed room.	Good
Triplett, et al. (2017) USA	Pre-post intervention and survey /Quantitative	Inpatient psychiatric units (n=2)	Nursing staff	To manage patient acuity with less reliance on observers, maintain a safe milieu, keep patient beds open to admissions, and reduce observer cost.	Both units saw decreases in acts of aggression (Unit A from a monthly average of 0.9 to 0.5 events per month; Unit B dropped from 0.2 events per month to 0.1), though neither was statistically significant. Unit A saw an increase in the overall number of patients requiring seclusion or restraints, but this actually represented a decrease when adjusted for the overall increase in patients seen, though neither was statistically significant. Unit B saw decreases in the number of patients put in seclusion or restraints during the pilot that were not statistically significant. Overall minutes in seclusion or restraints increased on Unit A, and this increase persisted when adjusted for increased patient throughput on the unit, though no measure was statistically significant.	Good

Van der Schaaf, et al. (2013) Netherlands	Longitudinal/ Quantitative	Forensic wards (n=25)	Patients (n=616); 78.7% male; mean age 37.6 (SD=10.5)	To explore the effect of design features on the seclusion risk, frequency, and duration within locked wards for intensive psychiatric care	Design features increasing the risk of being secluded were: ‘presence of an outdoor space’; the availability of ‘special safety measures’; and a large ‘number of patients in the building’. Design features decreasing the risk of being secluded were: more ‘total private space per patient’; a higher ‘level of comfort’; and greater ‘visibility on the ward’.	Good
Verbeek, et al. (2014) Netherlands Ì	Quasi-experimental/ Quantitative	Nursing homes with specialized psychogeriatric, somatic, or rehabilitation wards	Residents (n=124) in small-scale living facilities; 80% female; mean age 82.4 (SD=7.9). Control (n=135); 70% female; mean age 83.1 (SD=6.5)	To examine the effects of small-scale living facilities on residents’ behaviour, focusing on neuropsychiatric symptoms, social engagement, and the use of physical restraints and psychotropic drugs	The small-scale facilities employed significantly fewer physical restraints and psychotropic drugs compared with traditional wards. Levels of social engagement and physically non-aggressive behaviour were higher in small-scale facilities than traditional wards.	Good
Wilkes, et al. (2005) Australia	Quasi-experimental/Quantitative	A Special Care Unit (SCU) at a nursing home	Persons with dementia (n=16); 81% female	To explore the effects on challenging (agitated) behaviours, when relocating patients with dementia to a special unit	Results revealed no significant differences in resident aggressive behaviour scores ($P = 0.220$) over time. Verbal agitation reduced throughout the 6 months of the study after moving into a SCU ($P < 0.01$).	Fair

Medication safety

Author, year, country	Study design	Setting	Participants	Aims/ objectives related to patient safety	Outcomes related to patient safety	Study quality
Bademli & Buldukoglu (2009) Turkey	Questionnaire/ Quantitative	Inpatient psychiatric wards in hospitals (n=34)	Psychiatric nurses (n=471); 100% female. Mean age 32.4+/-7.3	To describe oral medication management by nurses in psychiatric wards	73.9% of nurses collected medication history data, with 90.23% asking whether the patient had taken the medication previously. The orders were checked by 80.5% of nurses every day. If a nurse was not sure of something, 93.4% asked the physician. 59.7% of the nurses checked all the patients' mouths after each pill was given. The most common patient reaction during medication administration was refusal to take the medication (93.2%). 86.8% of nurses informed the physician when this occurred. Nurses primarily observed the patient to evaluate the effect of a medication (84.3%). Age, education and years of experience created a significant difference in approaches to patients who did not take their medications.	Fair
Cottney (2014) UK	Pre-post observation/ Quantitative	An acute adult inpatient mental health ward	Staff	To evaluate the benefits of automated dispensing cabinets (ADCs) for increased medication safety	There was a reduction in the rate of medication administration errors from 8.9% to 7.2%, but this was not statistically significant (p=0.065, 95% CI 0% to 3.5%.	Poor

Cottney & Innes (2015) UK	Observation/ Quantitative	Inpatient mental health wards (n=43) within a mental health trust	Staff and patients	To explore medication-administration errors within a mental health trust	In 4177 medication administration opportunities, 139 medication errors were detected (3.3%). The error most frequently made was incorrect dose omission (37%). 11% of the errors had a serious clinical severity level. Four factors predicted medication error: administering nurse having to attend to other duties during the medication round (relative risk (RR) =1.48, 95% (CI): 1.14–1.93, p=0.003); number of 'pro re nata' (prn) doses given (RR=1.15, 95% CI: 1.03–1.28, p=0.012); number of patients on the ward (RR=1.06, 95% CI: 1.01–1.13, p=0.03); and number of regular doses due (RR=1.02, 95% CI: 1.02–1.03, p=≤ 0.0001).	Good
Dickens, Stubbs & Haw (2008) UK	Observational/Quantitative	Inpatient wards for older people (n=2) within a psychiatric hospital	Registered nurses (n=9)	To explore the delegation of medication administration within older people mental health wards	78% of delegated medication doses were to another registered nurse, but 22% were delegated to care workers. Care workers were more likely to administer medication to aggressive/confused patients. Errors occurred in 20% of doses, with the most common being the opening/crushing of medication without authorisation (41% of errors). 99% of errors were in the preparation/recording and so not attributable to the administrator of the medication.	Fair

Dolan & Kirwan (2001) UK	Questionnaire/ Quantitative	A medium secure unit for mentally disordered offenders (MSU)	Staff (n=62)	To explore staff perceptions of illicit drug use and its impact on MSUs	60% of staff reported awareness of drug misuse on the unit. The majority of staff (82%) mentioned the adverse effects on psychopathology (worsening symptoms, aggression). 45% also expressed concern regarding negative impacts upon staff-patient relationships. Most staff commented that the unit had policies of bag, room or visitor searching. 31% of staff felt that the police should be called if patients were in possession of drugs, 40% felt that sanctions should be imposed.	Fair
Haw, et al. (2007) UK	Cross-sectional/ Mixed methods	Elderly long-stay wards in a psychiatric hospital (n=2)	Nurses (n=9)	To use observations to determine the frequency and nature of medication administration errors	In 1423 doses, 369 medication errors were made (25.9%). The most frequent error type was crushing tablets without the authorization of the prescriber (28.7%). The median rate of medication error was one in every 6.4 doses.	Poor
Haw, et al. (2014) UK	Vignettes and semi-structured interviews/ Qualitative	Psychiatric hospital	Nurses (n=50)	To explore reasons for not reporting medication errors	48% of nurses stated that they would report a medication error made by someone else, and 40% would report a near-miss involving themselves. Thematic analysis revealed reasons for this was four-fold: knowledge, fear, burden of work, and excusing the error.	Fair
Hughes, et al. (2018) Europe, Australasia, the Middle East, South East Asia, North America and Africa	Online survey/ Quantitative	Inpatient mental health setting	Mental health professionals (n=98) working in Europe (n=77) 79%, Australasia (n=9) 9%, the Middle East (n=6) 6%, South East Asia (n=4) 4%, North America (n=1) 1% and	To undertake a scoping survey to explore inpatient mental health workers' perceptions of novel psychoactive substances (NPS) use by consumers.	Over 90% of participants reported observing at least one adverse event relating to NPS use in the previous month. The majority of participants reported that patients had used NPS during their inpatient admission. Three quarters	Fair

			Africa (n=1) 1%.		were not clear if their workplace had a policy about NPS. Most wanted access to specific NPS information and training. Participants reported that they lacked the necessary knowledge and skills to manage NPS use in the patients they worked with.	
Gonzalez-Pinto, et al. (2010) Spain	Prospective Observational/ Quantitative	An inpatient setting	Non-adherent inpatients (n=161) and adherent inpatients (n=460)	To describe the frequency of adherence and non-adherence with anti-manic and mood stabilizing medication among bipolar disorder patients, and to identify factors associated with treatment adherence	Higher levels of insight was associated with higher levels of medication adherence. Cannabis abuse/dependence during the acute phase of the patient's condition, work impairment, and higher levels of hallucination/delusion at baseline were associated with lower levels of medication adherence.	Fair
Keers, et al. (2018) England	Interviews/Qualitative	A mental health National Health Service (NHS) hospital.	20 Student and Registered Mental Health Nurses	To investigate in-depth the underlying causes of MAEs in a mental health National Health Service (NHS) hospital.	The majority of errors involved medications used to treat central nervous system (CNS) disorders (n = 21), and most commonly involved either wrong drug (n = 7), wrong patient (n = 5) or wrong dose errors (n = 4). The reported MAEs involved nurses of varying levels of experience post-qualification, with over half occurring when participants had 1 year or less (n = 14, 54%) experience (including 2 student nurse errors) and 7 (27%) when nurses had more than 5 years' experience. Each error and near miss (active failure) was preceded by combinations of underlying error and violation	Good

					provoking conditions.	
Prins, et al. (2013) Netherlands	Observation and structured interviews/Quantitative	An inpatient old age psychiatric clinic of a large psychiatric teaching hospital	Patients (n=50); mean age 68.9; 52% female	To compare the number of medication use discrepancies at admission when using the structured history of medication use (SHIM) procedure and usual procedure for taking medication history	Compared with usual care, the SHIM procedure identified a discrepancy in medication use in 78% of patients, and provided a more comprehensive and accurate approach to obtain medication history.	Good
Rodreguez-Leal, et al. (2016) Spain	Longitudinal/Quantitative	All in-patients addmitted to a psychiatric hospital	Psychiatric inpatients (n=225), 54% male, mean age 54.64. 76.7% had schizophrenia and schizoaffective disorders. 9.2% had major affective disorder. 5.5% of patients had a diagnosis form axis II as main diagnosis. The rest (8.6%) had other mental illness.	To detect QT interval prolongation among inpatients in a mental health setting	Over the 12-month study period, 225 patients were evaluated and 9 cases (4%) of long QT segment were detected. Findings led to treatment modification and patient close monitoring. No sudden cardiac deaths occurred during the study period.	Fair
Ružić, et al. (2011) Croatia	Cross-sectional/ Quantitative	Forensic psychiatric institutions (n=2)	Psychiatric patients (n=98); mean age 47	To examine the effect of antipsychotics selection (typical or atypical) on patient aggressiveness, side effects, and hospitalisation length	There were no significant differences between participants receiving typical and atypical antipsychotics.	Fair
Seemüller, et al. (2009) Germany	Naturalistic prospective/ Quantitative	Psychiatric university hospitals (n=7) and psychiatric district hospitals (n=5)	Inpatients (n=1014); 63% female; mean age 45.03 (SD=11.89)	To explore any suicidality promoting effects of antidepressants that cause an increased rate of suicidality-related events in high risk populations under routine treatment conditions	The rate of suicides (13.44/1000 patient-years) was low in the routine treatment condition compared to the rate observed in randomized controlled antidepressant trials. Predictors of extended emergence of suicide ideation were: Age (younger individuals were at higher risk), treatment	Good

					resistance, number of hospitalizations, and presence of akathisia and comorbid personality disorder.	
Sørensen, et al. (2013) Denmark	Descriptive, cross-sectional/ Quantitative	Psychiatric wards at a university hospital (n=3)	Patients (n=67); 64% female; mean age 46, age range 20-79	To investigate errors in the medication process in an inpatient psychiatric setting	There were errors in 9/324 (3%) opportunities for error of dispensed drugs in the observational study, and in 9/67 (13%) of dispensed drugs in the unannounced control visit, of which the majority was associated with one nurse assistant. Fewest errors were detected at the prescribing stage.	Fair
Strudwick, et al. (2017) Canada	Interviews/ Qualititative	Inpatient care settings: forensic, youth, geriatric, acute, and rehabilitation services (n=5)	Inpatients (n=52)	To elicit patient perceptions of barcode medication administration identification practices in inpatient mental health settings	Six themes emerged as a result of the inductive data analysis. These included: management of information, privacy and security, stigma, relationships, safety and comfort, and negative associations with the technology. Patients also indicated that they would like a choice in the type of identification method used during barcode medication administration. As well, suggestions were made for how barcode medication administration practices could be modified to become more patient-centered.	Good

Xie, at al. (2019) Canada	Interviews/Qualitative	Mental health and addiction academic teaching hospital	Mental health nurses (n=10).	To explore mental health nurses' perceptions of practices to improve barcode medication administration take-up in mental health inpatient settings	Interview themes were: safety, clinical workflow, education, accountability and strategies	Good

Unauthorised Leave

Author, year, country	Study design	Setting	Participants	Aims/ objectives related to patient safety	Outcomes related to patient safety	Study quality
Algase, et al. (2010) USA	Observational, questionnaire/ Quantitative	Long-term care units (LTCs) with dementia-specific units (N=28). These included nursing homes (n=22) and assisted living facilities (n=6)	Residents with dementia (n=122); 77% female; mean age 83.7 (range 68-102)	To investigate the influence of the physical environment on wandering behaviour	80% of wandering occurred in the residents' room, the community day room, the hall, or the eating halls. Location, light and sound impacted upon wandering behaviour. Odds ratio was highest for proximity to people (odds [OR] = 1.87).	Good
Bowers, Alexander & Gaskell (2003) UK	Controlled trial/ Quantitative	Acute psychiatric wards (n=5) in a hospital	All staff and patients within the units	To evaluate an intervention to reduce absconding by patients from acute psychiatric wards	Absconding fell by 25% during the intervention. Severe violent incidents increased significantly during the intervention ($\chi2=7.74$, $P=0.005$). However, wards that saw a decrease in absconding, there was no change in the frequency of violent incidents, and there were significant decreases in door locking.	Good
Colombo, et al. (2001) Italy	Questionnaire/ Quantitative	An SCU	Inpatients (n=132). 100% female. Mean age 78.6±9.4	To develop a profile of female wanderers with dementia within an SCU	Wandering occurred in 51% of participants. Wanderers were found to fall 3 times as much as non-wanderers over 3 months (1.6 and 0.6 falls respectively).	Poor
Grotto, et al. (2015) Australia	Interpretative inquiry/ Qualitative	Inpatient mental health units (n=3)	Mental health nurses (n=11); 55% female; age range (35-60)	To explore nurses' perceptions of assessment and management practices inpatient absconding	Clinical judgement influenced absconding assessment including historic markers of absconding. Nurses deemed risk assessment methods to be inadequate. There was no standardised management for dealing with absconding patients.	Fair
Hunt, et al. (2010) UK	Descriptive/ Quantitative	Psychiatric inpatient wards	Inpatient suicides away from ward (n=469); 66% male	To describe inpatient suicides by individuals who had absconded and to compare against those on agreed leave	Absconders were significantly more likely to die by jumping than those on agreed leave (49% vs 30%, $p< 0.001$ respectively). Absconders were also more likely to die within the first week of admission (19% vs 8%, $p< 0.001$) and to be non-compliant with medication (25% vs 13%, $p< 0.001$). Factors that could have	Fair

					reduced the likelihood of suicide were suggested: more patient supervision, better treatment compliance, and increased staff numbers, communication and training.	
Hunt, et al. (2016) UK	Questionnaire and interview/ Mixed methods	Inpatient ward	Mental health staff (n=21). 57% male. Median age 46 (range 37–58).	To identify the characteristics of inpatients who died by suicide after absconding and to explore these and further key issues related to suicide risk from the perspective of clinical staff.	Four themes were identified as areas of concern for clinicians: problems with ward design, staffing problems, difficulties in assessing risk, and patient specific factors.	Good
Meehan, Morrison & McDougall (1999) Australia	Semi-structured interviews/ Quantitative	Acute psychiatric unit in a general hospital campus	Inpatients (n=14); 36% female; mean age 37 (range 19-58 years)	To explore absconding behaviour from the patient perspective	Six issues were identified regarding absconding behaviour: boredom, lack of interesting activities, disturbed ward environment, the need for hospitalisation, issues at home, and perceived rewards from absconding. Situational and environmental factors were likely to increase the absconding risk.	Poor
Muir-Cochrane, et al. (2013) Australia	Semi-structured interviews/ Qualitative	An acute psychiatric unit	Mental health patients (n=12). 33% male	To explore the experiences of people who had been held involuntarily in an inpatient psychiatric unit and who had absconded or attempted to abscond	The inpatient unit is perceived as a safe or unsafe place, depending on physical, individual, social, and symbolic aspects of the unit. Absconding occurred when the unit was perceived as unsafe. Factors that would decrease the likelihood of absconding were identified: a therapeutic relationship with staff, familiarity with the unit, a comfortable environment, and positive interactions with peers.	Fair

Nijman, et al. (2011) UK	Questionnaire/ Quantitative	Acute psychiatric wards (n=133)	A qualified nurse	To investigate the prevalence of door locking and the use of other exit security measures on psychiatric wards, as well as investigating relationships between locking exit doors and absconding	There was no statistically significant relationship between exit security measures and absconding rates. However, there was a reduction of approximately 30% in absconding rates when the ward door was permanently locked compared to when doors were open.	Fair
Nurjannah, FitzGerald, & Foster (2009) Indonesia	Semi-structured interviews/ Qualitative	A psychiatric hospital	Patients (n=16). 94% male; mean age 33, range 13-65	To provide a rich description of the experience of patients related to incidents of absconding in a psychiatric setting	Three themes were highlighted: the call to home, hopes and realities, and us and them. The first theme concerned patients' need to connect with others and to feel safe. The second theme concerned patients' wishes to feel happy, which were often not compatible with realities of life at home or in the ward. The final theme concerned the competing interests and different opinions of patients regarding others, such as hospital staff and family.	Fair
Simpson, et al. (2015) Canada	Intervention design/ Quantitative	Forensic inpatient units (n=8) within a psychiatric hospital	Inpatients (n=188)	To investigate the impact of a new policy designed to reduce incidents of absconding in a forensic setting	The absconding rate decreased from 17.8% pre-implementation, to 13.8% during implementation, and further to 12.0% post-implementation. The most common reason for absconding was boredom/frustration.	Good

Clinical Decision Making

Author, year, country	Study design	Setting	Participants	Aims/ objectives related to patient safety	Outcomes related to patient safety	Study quality
Brown & Rakow (2015) UK	Questionnaire/ Quantitative	Psychiatric hospitals	Clinicians with an active role in violence risk assessment (n=9)	To explore clinicians' use of static and dynamic cues when assessing risk in psychiatric inpatients	Clinicians mostly used history of recent violence cues in assessments of in-hospital risk. Clinicians mostly used in-hospital frequency and severity of violence cues when assessing the risk posed by the patient if they remained in the current hospital.	Fair
Fuller & Cowan (1999) UK	Natural experiment/ Quantitative	Inpatient units (n=2) within a forensic hospital	Patients (n=75). 87% male. Modal age group 20-29	To assess the accuracy of staff predictions of patient clinical risk in forensic settings	The most common risk event was acting out against fellow patients (25% of risk events). Multidisciplinary clinical judgement predicted a variety of risk events at a higher than chance level. The risk event with the highest AUC statistic was prediction of serious incidents involving staff (AUC=0.856, $p \leq 0.0001$).	Fair
Green, et al. (2018) UK	Focus group/ Qualitative	Medium secure ward	Registered nurses (n=12). 75% female.	To specifically explore the decision-making process behind qualified nurses' decisions to implement the use of seclusion in forensic mental health care.	Participants described the need to reduce the use of seclusion and the problematic nature of its utility as an ongoing intervention in contemporary mental healthcare. It was clear that there were complexities and competing variables involved in the decision-making process. The data analysis resulted in the identification of four themes: 1) seclusion as a last resort, 2) presenting behaviours, 3) organisational influences and 4) professional judgement.	Good
Koukia, Madianos & Katostaras (2009) Greece	Cohort design/ Qualitative	Psychiatric hospital	Nurses (n=103); 57% female; mean age 36.3 [7.7]	To explore the on-the-spot decision making process of nurses when faced with a critical incident	When violent incidents occur, physical restraints were frequently used. However, reassurance and support were common interventions when a critical incident occurred. Nurses wanted	Fair

					more skills training and higher autonomy.	
Lindsey (2009) USA	Correlational descriptive design/ Quantitative	Psychiatric hospital	Nurses (n=30); 87% female	To explore nurses' decisions to restrain psychiatric inpatients, and associations with work empowerment and individual patient/staff characteristics	When needed nurses chose high frequency medication and did not display a standardised decision making process in their restraint use.	Fair
Mann-Poll, et al. (2011) Netherlands	Vignette and questionnaire/ Quantitative	Inpatient wards (n=4)	Staff (n=82)	To identify factors that contribute to decision making in relation to seclusion	The factors contributing to decisions to seclude patients came mostly from rater characteristics (e.g. type of care received from staff, frequency of participation in seclusion, experience of using seclusion as an intervention) and vignette variables (e.g. how approachable patient seemed, dangerousness and availability of rooms).	Fair
Marangos-Frost & Wells (2000) Canada	Ethnographic/ Qualitative	Psychiatric inpatient unit	Staff (n=6)	To understand nurses' decisions to restrain patients and feelings about those decisions	There were four main themes: framing the situation as a potential for immediate harm; unsuccessful exploration of alternative interventions; the conflicted nurse; and the conditions of restraint	Fair
Whaley (2001) USA	Cross-sectional/ Quantitative	A psychiatric hospital	Patients (n=118), age range 18-59	To evaluate agreement between clinical diagnoses and research diagnoses of schizophrenia for African American psychiatric patients. To explore the relationship of cultural mistrust with any disagreement	Levels of agreement for diagnoses was poor in 5 out of 6 comparisons. There were, however, significantly more cases using clinical diagnoses than other methods. Level of cultural mistrust did not seem to predict the excess in clinical diagnoses of paranoid schizophrenia.	Fair

Whaley (2004) USA	Cross-sectional/ Quantitative	A psychiatric hospital	Patients (n=94)	To explore cultural bias and diagnoses of schizophrenia for African American psychiatric patients	Highest rates of agreement were found between clinical and best estimate diagnoses of schizophrenia (kappa=.77, p< .01). Patients' cultural mistrust was positively associated with the number of clinical diagnoses of paranoid schizophrenia, b=.364, se=.123, p < .01, and best estimate diagnoses of paranoid schizophrenia, b= .314, se= .128, p< .05. This implies a bias towards diagnosing cultural attitudes as pathology in African American psychiatric patients.	Fair

Falls

Author, year, country	Study design	Setting	Participants	Aims/ objectives related to patient safety	Outcomes related to patient safety	Study quality
Abraham (2016) USA	Cross-sectional/Quantitative	Inpatient psychiatric units, USA	Psychiatric inpatient program supervisors, managers, directors, or administrators (n=80). 45.5% were 55-64, 36.4% were 45-54. 3.0% were 25-34. One participant was older than 65.	To explore psychiatric unit directors' perceptions of the factors that contribute to patient falls in psychiatric inpatient units in the State of Michigan.	Team work was the most common extrinsic factor related to decreasing patient falls (M, 4.55 [0.61]). Supervision and 15-min checks were also deemed relevant (M 4.00 [0.88] and M 3.56 [0.84] respectively). Participants agreed mostly that staff training and education rather than employing certified psychiatric technicians were needed to prevent falls in the unit (M, 4.32 [0.59 and 4.05 [1.07] respectively). One on one supervision had the lowest level of agreement as factor to reduce falls in the unit (M 2.92 [1.19]).	Fair

Fonad, et al. (2009) Sweden	Longitudinal/Quantitativ e	Dementia nursing home units	Staff nurses	To explore associations between falls and: fall risks, fractures, the use of physical restraints and the use of medications in dementia nursing home units	737 fall incidents ocurred over the 4 year period. Falls were associated with fractures, assessed risk of falling, the use of certain medication (neuroleptics and sleeping medication), and physical restraints (wheelchairs, belts and bed rails).	Fair
Garfinkel, et al. (2007) Israel	Longitudinal case-control/Quantitative	Dementia-specialised departments (n=4) within a medical centre	Patients (n=206).	To evaluate the efficacy of hip protectors in reducing hip fractures in dementia patients.	There was no significant difference in the fall rate between patients wearing/not wearing hip protectors. There was a significant decrase in the rate of hip fracture per fall for those patients wearing hip protectors (P= 0.007, relative risk (RR)=5.63).	Fair
Holmes, et al. (2007). USA	Quasi-experimental study	Special care unit (SCU) for inpatients with dementia	Residents with dementia (n=78): intervention group (n=38) and comparison unit (n=40); no other details mentioned	To assess the extent to which modern technology can augument or substitute for direct staff intervention in late-evening and nighttime situations. Specifically that modern technology would significantly contribute to the reduction of falls, accidents and injuries	The modern technology intervention did not significantly reduce the number of falls or accidents on the unit compared to comparison unit. Furthermore there was no extra staff-perceived burden with the presence of the intervention.	
Powell-Cope, et al. (2014). United States.	Descriptive Study using focus groups	One inpatient unit at a Veterans' hospital	Registered nurses (n=22), physical therapist (n=1) and physicians (n=2)	Determine the barriers and facilitators for implementing fall prevention and protection programs in inpatient psychiatry.	Results were grouped into 3 fall-related categories: Fall Risk Assessment (e.g. Participants noted a ceiling effect with existing fall risk instruments and lack of sensitivity to psychiatry populations) Clinical Fall Risk Precautions (e.g. use of bed modifications to improve mobility), Programmatic Fall Prevention (e.g. The necessity of using an interdisciplinary focus for falls prevention programs was	Good

					echoed by all study participants)	
Tängman, et al. (2010). Sweden.	Prospective study	A 24 bed psychogeriatric ward	Patients (n=91); 50.5% female; mean age 80.3, age range 60-94 (SD=7.4)	The aim of this study was to identify precipitating factors for falls among people with dementia.	The most likely factor or combination of factors could be ascertained in 247 falls (83%). Acute disease or symptoms of disease and/or acute drug side-effects were, alone or in combination with other factors, judged to precipitate more than three out of four falls.	Fair

Infection prevention and control

Author, year, country	Study design	Setting	Participants	Aims/ objectives related to patient safety	Outcomes related to patient safety	Study quality
Li, et al. (2019) Taiwan	Interviews/ Qualitative	Psychiatric hospitals (n=6) and general hospitals with psychiatric facilities (n=2)	Psychiatric infection preventionists (n=13). 100% female. Mean age 41.9 (range 31- 48)	To understand how infection preventionists (IPs) perceived their challenges and how these challenges negatively affect their infection prevention work in psychiatric clinical settings.	Data analysis revealed that the participants' experiences of working as IPs in the psychiatric wards and associated challenges were captured by 6 themes: (1) lack of preservice training in psychiatric infection control, (2) insufficient staffing in practice, (3) working within environmental limits, (4) patient noncompliance, (5) undervaluation of the importance of infection control by professionals, and (6) involvement of hospital administrators.	Fair
