# Supplementary material for: Patient safety in inpatient mental health settings: a systematic review
Source: BMJ Open. 2019 Dec 23;9(12):e030230. doi: 10.1136/bmjopen-2019-030230 (PMC7008434; doi:10.1136/bmjopen-2019-030230)
Supplement: Supplementary data [file bmjopen-2019-030230supp005.pdf]

Author, year	**Abstract and title:** *Did they provide a clear description of the study?*	**Introduction and aims:** *Was there a good background and clear statement of the aims of the research?*	**Method and data:** *Is the method appropriate and clearly explained?*	**Sampling:** *Was the sampling strategy appropriate to address the aims?*	**Data analysis:** *Was the description of the data analysis sufficiently rigorous?*	**Ethics and bias:** *Have ethical issues been addressed, and necessary ethical approval gained? Has the relationship between researchers and participants been considered?*	**Results:** *Is there a clear statement of the findings?*	**Transferability or generalizability:** *Are the findings of this study transferable to a wider population?*	**Implications and usefulness:** *How important are these findings to policy and practice?*	**Overall Quality Score**	**Overall Quality Rating**
Abdel-Hussein & Mohamed (2018)	20	20	20	20	10	10	20	20	10	16.67	**Poor**
Abraham (2016)	20	20	20	30	20	0	30	20	20	20.00	**Fair**
Ajalli et al. (2018)	30	20	30	20	40	30	40	30	30	30.00	**Good**
Algase et al. (2010)	30	30	30	30	40	20	40	30	30	31.11	**Good**
Almvik, Rasmussen & Woods (2006)	30	20	20	20	20	20	40	20	20	23.33	**Fair**
Amoo & Fatoye (2010)	30	30	20	20	30	20	40	20	40	27.78	**Fair**
Arguvanli, et al. (2015)	30	20	30	20	30	20	30	20	30	25.56	**Fair**
Awenat et al. (2018)	40	40	30	30	30	30	30	40	40	34.44	**Good**
Bademli & Buldukoglu (2009)	30	20	30	20	30	20	40	20	40	27.78	**Fair**
Bahareethan & Shah (2000)	30	20	30	20	30	20	30	20	40	26.67	**Fair**
Bak & Aggernæs	30	20	20	10	10	0	30	20	20	17.78	**Poor**

(2012)											
Bak, et al. (2013)	30	20	30	30	40	20	40	30	40	31.11	**Good**
Bak, et al. (2015)	30	20	30	30	40	20	40	30	30	30.00	**Good**
Barr et al. (2019)	30	40	30	30	30	20	30	30	30	30.00	**Good**
Bayramzadeh (2016)	30	30	10	10	10	20	20	20	20	18.89	**Poor**
Bellantonio, et al. (2008)	30	20	40	30	30	20	40	30	40	31.11	**Good**
Bennett, Ramakrishna & Maganty (2011)	20	20	20	20	40	0	40	20	40	24.44	**Fair**
Ben-Zeev et al. (2017)	30	20	30	30	30	10	30	30	30	26.67	**Fair**
Berg, et al. (2013)	40	20	40	20	30	20	40	20	40	30.00	**Good**
Berg, Kaltiala-Heino & Välimäki (2011)	30	40	40	20	40	20	40	20	30	31.11	**Good**
Bergk, et al. (2011)	40	20	30	20	30	20	40	20	30	27.78	**Fair**
Bharwani, et al. (2012)	30	20	30	20	20	0	30	20	30	22.22	**Fair**
Biancosino, et al. (2009)	30	30	30	20	30	0	40	20	40	26.67	**Fair**
Bigwood & Crowe (2008)	30	20	40	20	40	20	30	20	30	27.78	**Fair**
Bilici et al. (2016)	20	30	30	20	30	20	30	20	40	26.67	**Fair**
Björkdahl, Hansebo & Palmstierna (2013)	30	20	30	20	30	20	40	20	30	26.67	**Fair**
Blair et al (2017)	30	20	20	30	30	20	30	30	20	25.56	**Fair**
Bleijlevens,	40	30	30	20	30	20	30	20	30	27.78	**Fair**

et al. (2013)											
Bonner & Wellman (2010)	30	20	40	20	20	20	40	20	40	27.78	**Fair**
Bonner, et al. (2002)	20	20	40	20	30	20	30	20	40	26.67	**Fair**
Booth, et al. (2014)	30	20	30	20	20	20	40	20	40	26.67	**Fair**
Boström, et al. (2011)	30	40	30	20	20	20	40	20	40	28.89	**Fair**
Boumans, et al. (2012)	20	40	30	20	30	0	40	20	40	26.67	**Fair**
Bowers & Crowder (2012)	40	20	30	30	40	20	30	30	40	31.11	**Good**
Bowers (2009)	30	30	30	30	40	20	30	30	40	31.11	**Good**
Bowers, Alexander & Gaskell (2003)	40	20	30	30	30	20	40	30	30	30.00	**Good**
Bowers, et al. (2006)	30	20	30	20	20	20	40	20	30	25.56	**Fair**
Bowers, et al. (2007)	40	20	20	20	30	20	30	20	40	26.67	**Fair**
Bowers, et al. (2008)	30	20	30	30	40	20	40	30	40	31.11	**Good**
Bowers, et al. (2009)	30	20	30	30	30	20	30	30	40	28.89	**Fair**
Bowers, et al. (2010)	30	20	30	30	30	20	30	30	30	27.78	**Fair**
Bowers, et al. (2012)	30	20	30	30	30	20	30	30	30	27.78	**Fair**
Bowers, Gournay & Duffy (2000)	40	20	30	20	20	0	30	20	30	23.33	**Fair**
Brady et al. (2017)	40	30	30	30	30	20	30	30	40	31.11	**Good**
Braham, Heasley & Akiens (2013)	30	20	30	20	20	30	30	20	30	25.56	**Fair**

Brennan, Flood & Bowers (2006)	20	20	30	30	20	30	40	30	30	27.78	**Fair**
Brown & Beail (2009)	40	20	30	20	40	30	40	20	30	30.00	**Good**
Brown & Rakow (2016)	40	20	40	20	30	20	30	20	40	28.89	**Fair**
Calabro, Mackey & Williams (2002)	30	20	30	20	30	20	40	20	40	27.78	**Fair**
Camuccio, et al. (2012)	30	30	30	20	20	20	40	20	40	27.78	**Fair**
Carlson, et al. (2010)	30	20	30	20	10	20	30	10	20	21.11	**Fair**
Caspi (2014)	40	20	30	20	20	40	40	20	40	30.00	**Good**
Caspi (2015)	20	30	30	20	30	40	40	20	40	30.00	**Good**
Caspi, et al. (2001)	30	20	30	20	20	20	40	20	30	25.56	**Fair**
Chan, et al. (2005)	30	20	30	30	10	20	40	30	20	25.56	**Fair**
Chandler (2008)	20	20	30	20	30	20	40	20	40	26.67	**Fair**
Chaplin, et al. (2008)	0	20	20	20	0	0	40	20	20	15.56	**Poor**
Chen, et al. (2007)	30	20	40	20	20	20	30	20	30	25.56	**Fair**
Chen, Hwu & Wang (2009)	30	20	30	20	30	20	30	20	30	25.56	**Fair**
Chien, Chan & Kam (2005)	30	20	40	20	30	20	40	20	30	27.78	**Fair**
Ching, et al. (2010)	20	40	30	20	40	20	40	20	40	30.00	**Good**
Chu, et al. (2015)	30	20	30	20	0	10	40	20	30	22.22	**Fair**
Cleary, et al. (1999)	20	20	30	20	20	0	40	20	30	22.22	**Fair**
Cole,	30	20	20	20	30	0	30	20	30	22.22	**Fair**

Baldwin & Thomas (2003)											
Colombo, et al. (2001)	20	20	20	20	10	0	30	20	20	17.78	**Poor**
Cormac, Russell & Ferriter (2005)	0	20	20	20	10	0	30	20	30	16.67	**Poor**
Cottney & Innes (2015)	30	20	40	20	30	30	40	20	40	30.00	**Good**
Cottney (2014)	30	20	30	10	0	0	30	20	30	18.89	**Poor**
Cowan et al. (2018)	30	20	30	20	40	20	40	20	30	27.78	**Fair**
Cowman & Bowers (2008)	30	20	20	20	40	0	30	20	40	24.44	**Fair**
Cullen, Nath & Marcus (2010)	30	20	20	20	40	20	30	20	40	26.67	**Fair**
Curtis, et al. (2013)	20	20	20	20	30	20	30	20	30	23.33	**Fair**
Cutcliffe (1999)	20	20	30	20	30	20	30	20	40	25.56	**Fair**
Daffern (2007)	20	20	20	20	0	10	30	20	40	20.00	**Fair**
Daffern, et al. (2009)	20	20	30	20	30	20	40	20	40	26.67	**Fair**
Daffern, Mayer & Martin (2006)	20	40	40	10	30	10	30	20	40	26.67	**Fair**
Daffern, Ogloff & Howells (2003)	20	20	40	10	10	20	30	20	40	23.33	**Fair**
Danivas et al. (2016)	40	30	40	30	40	20	40	20	40	33.33	**Good**
Davies et al. (2019)	30	30	30	20	30	20	30	20	40	27.78	**Fair**
Davis,	20	20	30	20	20	20	30	20	40	24.44	**Fair**

Williams & Hays (2002)											
de Jonghe-Rouleau, Pot & de Jonghe (2005)	20	20	30	30	20	10	40	30	30	25.56	**Fair**
de Looff et al. (2018)	40	40	30	30	40	20	30	30	40	33.33	**Good**
De Niet, Hutschemaekers & Lendemeijer (2005)	30	30	30	20	20	0	40	20	30	24.44	**Fair**
De Young, Just & Harrison (2002)	30	20	30	20	20	20	30	20	40	25.56	**Fair**
Delaney & Johnson (2006)	30	20	30	20	30	20	40	20	0	23.33	**Fair**
Delaney, et al. (2001)	20	20	20	20	20	0	30	20	40	21.11	**Fair**
Dickens, Piccirillo & Alderman (2013)	20	20	30	20	40	20	30	20	30	25.56	**Fair**
Dickens, Stubbs & Haw (2008)	30	20	40	20	10	30	30	20	40	26.67	**Fair**
Dolan & Kirwan (2001)	40	20	20	20	0	0	40	20	30	21.11	**Fair**
Drew (1999)	30	20	30	20	0	10	40	20	40	23.33	**Fair**
Dreyfus et al. (2018)	30	30	30	20	20	10	40	20	10	23.33	**Fair**
Duxbury & Whittington (2005)	30	20	30	20	20	20	30	20	40	25.56	**Fair**
Duxbury et al. (2019)	30	30	20	10	20	10	40	20	30	23.33	**Fair**
Ellis, et al. (2012)	20	20	30	20	30	20	40	20	30	25.56	**Fair**

Ellis, et al. (2015)	20	30	30	20	30	20	40	20	40	27.78	**Fair**
Elmer et al. (2018)	30	20	20	20	30	20	30	20	40	25.56	**Fair**
Elzubeir et al (2017)	40	30	40	20	40	30	40	20	40	33.33	**Good**
Espinosa, et al. (2015)	30	20	20	10	0	0	20	20	30	16.67	**Poor**
Esposito-Smythers, McClung & Fairlie (2006)	20	20	30	20	10	20	40	20	40	24.44	**Fair**
Evans & Petter (2012)	30	30	30	20	30	20	30	20	40	27.78	**Fair**
Exworthy, et al. (2001)	20	20	30	20	0	0	40	20	20	18.89	**Poor**
Ezeobele, et al. (2014)	30	30	40	20	30	20	40	20	40	30.00	**Good**
Faschingbauer, Peden-McAlpine & Tempel (2013)	30	20	40	30	30	20	40	20	30	28.89	**Fair**
Fish & Hatton (2017)	20	30	20	10	20	30	40	20	20	23.33	**Fair**
Fish (2018)	30	20	20	10	20	30	30	20	30	23.33	**Fair**
Fletcher et al. (2019)	30	20	40	20	30	20	30	20	30	26.67	**Fair**
Foley, et al. (2003)	20	30	40	20	30	20	40	20	40	28.89	**Fair**
Fonad, et al. (2009)	30	30	30	20	30	10	40	20	40	27.78	**Fair**
Fuller & Cowan (1999)	20	20	40	20	30	0	30	20	40	24.44	**Fair**
Gabrielsson, et al. (2014)	30	20	30	20	30	20	40	20	40	27.78	**Fair**
Gallop, et al. (1999)	20	30	40	20	40	20	40	20	30	28.89	**Fair**

Garfinkel, et al. (2007)	30	10	40	20	20	20	30	20	30	24.44	**Fair**
Gebhardt & Steinert (1999)	30	20	30	20	30	0	40	20	30	24.44	**Fair**
Georgieva, Mulder & Noorthoorn (2013)	30	20	40	30	40	30	40	30	40	33.33	**Fair**
Georgieva, Mulder & Wierdsma (2012)	20	20	30	30	40	20	40	30	40	30.00	**Fair**
Gerace et al. (2018)	30	20	20	20	30	30	20	20	20	23.33	**Fair**
Gibson et al. (2014)	30	20	40	30	40	20	40	20	30	30.00	**Fair**
Gifford & Anderson (2010)	30	30	30	20	30	20	40	30	30	28.89	**Fair**
Giles et al. (2005)	30	40	40	30	10	0	30	20	20	24.44	**Fair**
Gonzalez-Pinto et al. (2010)	30	40	30	20	40	30	40	20	30	31.11	**Good**
Gough & Hawkins (2000)	30	20	30	30	20	10	30	20	30	24.44	**Poor**
Goulet et al (2017)	30	30	30	40	40	20	30	40	40	33.33	**Good**
Goulet et al (2018)	30	20	40	30	40	20	40	30	40	32.22	**Good**
Gowda et al (2018)	30	30	30	20	20	20	20	20	40	25.56	**Fair**
Gowda et al (2019)	30	20	20	20	20	20	30	20	40	24.44	**Fair**
Gowda et al. (2019)	30	20	20	10	20	20	20	20	20	20.00	**Fair**
Green et al. (2018)	30	30	40	20	30	30	40	20	30	30.00	**Good**
Grotto et al. (2014)	30	20	30	20	40	20	30	20	40	27.78	**Fair**

Gustafsso et al (2016)	30	30	30	20	40	30	40	20	30	30.00	**Good**
Haglund & von Essen (2005)	30	20	30	20	30	20	30	20	20	24.44	**Poor**
Haines et al. (2017)	40	30	30	20	40	20	40	20	40	31.11	**Good**
Hallett & Dickens (2015)	40	30	30	40	30	20	40	30	40	33.33	**Fair**
Happell & Koehn (2011)	30	30	40	20	40	20	40	20	40	31.11	**Fair**
Hatta et al. (2007)	30	20	30	20	30	10	40	20	30	25.56	**Fair**
Haugom & Granerud (2016)	30	20	30	10	40	30	40	20	40	28.89	**Fair**
Haw et al. (2007)	40	20	30	20	20	20	20	20	30	24.44	**Poor**
Haw et al. (2011)	30	20	40	20	40	20	30	20	40	28.89	**Fair**
Haw et al. (2014)	40	20	40	20	40	20	40	20	30	30.00	**Fair**
Higgins et al. (2018)	40	30	30	20	30	20	40	20	30	28.89	**Fair**
Higueras et al. (2006)	30	20	30	20	30	20	20	20	40	25.56	**Fair**
Hill et al. (2017)	30	20	30	30	30	20	30	30	30	27.78	**Fair**
Holmes et al. (2007)	30	20	30	30	40	10	30	20	40	27.78	**Fair**
Holmes, Kennedy & Perrorn (2004)	40	20	40	20	40	40	40	20	40	33.33	**Fair**
Holmes, Murray & Knack (2015)	30	20	30	20	40	30	40	20	40	30.00	**Fair**
Holth et al. (2018)	40	20	30	20	30	20	40	20	30	27.78	**Fair**
Hottinen et	30	40	40	20	40	20	20	20	40	30.00	**Fair**

al. (2012)											
Hotzy et al (2019)	30	30	30	20	30	20	30	20	40	27.78	**Good**
Hughes et al. (2018)	30	20	30	30	30	20	30	30	40	28.89	**Fair**
Huizing et al. (2006)	40	30	40	30	40	30	30	30	40	34.44	**Fair**
Hunt et al (2016)	40	30	20	30	40	20	40	30	20	30.00	**Good**
Hunt et al. (2010)	40	20	40	30	40	20	40	40	30	33.33	**Fair**
Hunt et al. (2012)	30	20	40	30	40	10	40	30	30	30.00	**Fair**
Hvidhjelm et al (2016)	30	20	40	30	40	30	40	30	30	32.22	**Good**
Hylén et al. (2019)	20	20	30	10	40	30	40	20	30	26.67	**Fair**
Ilkiw-Lavalle & Grenyer (2003)	30	20	30	20	30	30	30	20	40	27.78	**Fair**
Inoue et al. (2017)	30	20	30	20	30	20	40	20	20	25.56	**Fair**
Ireland et al (2019)	20	20	30	20	10	20	30	20	30	22.22	**Fair**
Ireland et al. (2019)	30	40	30	20	40	20	30	20	40	30.00	**Good**
Ireland, Halpin & Sullivan (2014)	30	30	40	20	30	20	30	20	30	27.78	**Fair**
Isaak et al. (2016)	30	40	30	20	30	10	30	20	40	27.78	**Fair**
Jacob et al. (2013)	30	20	40	20	20	20	30	20	40	26.67	**Fair**
Jaeger et al. (2014)	30	30	40	10	40	20	30	20	30	27.78	**Fair**
Jalil et al. (2017)	40	40	30	30	40	20	40	30	40	34.44	**Good**
James et al. (2017)	40	20	30	40	40	20	30	40	30	32.22	**Good**
Janicki (2009)	30	20	30	30	40	30	40	40	40	33.33	**Fair**

Jeffs et al. (2012)	30	30	40	30	40	40	40	40	40	36.67	**Good**
Johnson & Delaney (2006)	40	20	30	20	40	10	40	20	40	28.89	**Fair**
Johnston & Kilty (2016)	20	30	20	10	10	20	40	20	10	20.00	**Fair**
Jones et al. (2010)	30	20	40	20	40	20	40	20	40	30.00	**Fair**
Jonker et al. (2008)	30	30	30	20	40	20	40	20	40	30.00	**Fair**
Kalagi et al. (2018)	40	30	30	30	40	30	40	30	30	33.33	**Good**
Kanerva et al. (2015)	30	20	40	20	40	20	40	20	40	30.00	**Fair**
Kanerva, Lammintakanen & Kivinen (2016)	30	20	40	20	40	20	40	20	30	28.89	**Fair**
Keers et al. (2018)	40	40	40	30	40	30	30	30	30	34.44	**Good**
Kelly et al. (2016)	20	20	40	20	30	20	30	20	40	26.67	**Fair**
Kelly et al. (2017)	30	30	40	30	30	20	40	30	40	32.22	**Good**
Keski-Valkama et al. (2007)	30	20	40	30	40	20	40	20	30	30.00	**Fair**
Killick & Allen (2005)	30	20	40	20	30	10	20	20	20	23.33	**Poor**
Kirkevold & Engedal (2004)	30	20	40	30	10	20	30	40	30	27.78	**Fair**
Knowles, Hearne & Smith (2015)	30	20	30	30	40	20	40	30	40	31.11	**Fair**
Kontio et al. (2010)	30	30	30	20	40	20	30	20	40	28.89	**Fair**
Kontio et al. (2009)	30	20	30	30	40	20	40	30	40	31.11	**Fair**
Kontio et al.	40	20	40	20	40	20	40	20	40	31.11	**Fair**

(2011)											
Kontio et al. (2012)	30	20	40	30	40	20	40	40	40	33.33	**Fair**
Kool et al. (2014)	40	20	30	20	40	20	40	20	40	30.00	**Fair**
Koukia, Madianos & Katostaras (2009)	30	20	40	20	40	20	20	20	40	27.78	**Fair**
Koukia et al. (2010)	30	20	40	20	40	20	30	20	40	28.89	**Fair**
Koukia et al. (2013)	30	20	40	30	40	10	30	20	40	28.89	**Fair**
Krieger et al. (2018)	30	30	40	30	40	10	40	30	40	32.22	**Good**
Kulkarni et al. (2014)	30	20	30	20	40	20	20	20	40	26.67	**Fair**
Kuosmanen et al. (2013)	30	30	30	20	40	20	30	30	30	28.89	**Fair**
Kuosmanen et al. (2015)	30	20	30	10	10	10	20	20	30	20.00	**Poor**
Kuosmanen et al. (2019)	40	40	30	30	40	20	40	30	40	34.44	**Good**
Lamanna et al. (2016)	40	20	30	20	30	10	40	20	30	26.67	**Fair**
Langan & McDonald (2008)	30	20	30	30	20	20	40	20	30	26.67	**Fair**
Lantta et al. (2015)	20	20	20	10	20	20	20	20	40	21.11	**Poor**
Lantta et al. (2016)	40	20	40	20	30	20	40	20	30	28.89	**Fair**
Lanza et al. (2009)	20	20	40	20	40	20	30	20	40	27.78	**Fair**
Lanza et al. (2016)	20	20	20	10	20	10	20	20	20	17.78	**Poor**
Larsen & Terkelsen (2014)	30	40	40	20	40	40	40	20	30	33.33	**Fair**
Larue et al. (2013)	30	20	40	30	40	30	40	30	30	32.22	**Fair**
Larue et al.	30	40	30	20	10	20	30	20	40	26.67	**Fair**

(2016)											
Lavelle et al. (2017)	40	30	30	20	20	20	40	20	40	28.89	**Fair**
Lawn & Pols (2003)	30	20	20	20	20	10	20	20	30	21.11	**Poor**
Lee et al. (2001)	30	20	20	20	10	10	20	20	30	20.00	**Poor**
Lee et al. (2003)	20	10	20	10	20	10	20	10	30	16.67	**Poor**
Lehmann, McCormick & Kizer (1999)	40	20	20	20	10	10	20	20	30	21.11	**Poor**
Li et al. (2019)	30	30	30	20	30	20	40	20	30	27.78	**Fair**
Lindgren, Aminoff & Graneheim (2015)	30	20	40	20	40	20	40	20	30	28.89	**Fair**
Lindsey (2009)	30	20	40	30	40	20	40	30	40	32.22	**Fair**
Lipscomb et al. (2012)	30	20	20	20	40	10	30	20	40	25.56	**Fair**
Long et al. (2014)	30	20	20	20	40	20	30	20	40	26.67	**Fair**
Looi, Engstrom & Savenstedt (2015)	30	20	30	10	40	20	40	10	30	25.56	**Fair**
Lovell, Smith & Johnson. (2015)	40	20	40	20	40	40	40	20	30	32.22	**Fair**
Lowe, Wellman & Taylor. (2003)	30	20	30		40	20	30	30	40	30.00	**Fair**
Lundegaard Mattson & Binder (2012)	40	30	30	20	40	40	40	20	30	32.22	**Good**
Mackay, Paterson &	30	30	40	30	30	10	40	40	40	32.22	**Fair**

Cassells (2005)											
Maguire, Daffern & Martin (2014)	30	20	40	20	40	20	40	20	40	30.00	**Fair**
Mahoney et al. (2012)	30	30	20	10	20	20	20	20	20	21.11	**Poor**
Mann-Poll et al. (2011)	30	40	30	20	40	10	30	20	40	28.89	**Fair**
Marangos-Frost & Wells (2000)	30	30	40	20	40	20	40	20	40	31.11	**Fair**
Martello et al. (2018)	30	30	30	20	40	20	40	20	40	30.00	**Good**
Martin & Daffern (2006)	30	40	40	20	20	20	40	20	40	30.00	**Fair**
Mason & Whitehead (2001)	30	40	30	20	20	10	30	20	40	26.67	**Fair**
McCann, Baird & Muir-Cochrane (2014)	30	20	40	20	30	20	30	20	40	27.78	**Fair**
McKeown et al. (2019)	40	30	30	20	40	30	40	20	30	31.11	**Good**
McLaughlin et al. (2010)	20	20	20	20	20	10	30	20	40	#REF!	**Poor**
Meaden, Hacker & Spencer (2013)	30	20	40	20	40	20	40	20	40	30.00	**Fair**
Meehan, McIntosh & Bergen. (2006)	30	20	40	20	40	20	40	20	30	28.89	**Fair**
Meehan, Morrison & McDougall. (1999)	30	20	30	20	20	10	30	20	40	24.44	**Poor**

Mezey, Hassell & Bartlett (2005)	30	20	40	30	40	20	40	40	40	33.33	**Fair**
Millar & Sands (2013)	30	40	40	30	40	20	40	40	40	35.56	**Good**
Mistler et al. (2017)	30	30	30	20	30	20	40	20	40	28.89	**Fair**
Molewijk et al (2017)	40	40	40	20	40	30	30	20	30	32.22	**Good**
Muir-Cochrane et al. (2012)	30	20	40	20	40	20	40	20	40	30.00	**Fair**
Muir-Cochrane et al. (2013)	30	20	30	20	40	20	40	20	20	26.67	**Fair**
Muir-Cochrane et al. (2015)	40	20	40	20	40	20	40	20	40	31.11	**Fair**
Murphy & Siv (2007)	20	20	20	30	20	20	30	30	30	24.44	**Fair**
Nathan et al. (2007)	30	40	30	20	30	30	30	20	30	28.89	**Fair**
Needham et al. (2004)	30	20	30	30	30	40	40	30	30	31.11	**Good**
Needham et al. (2005)	30	20	30	20	30	20	40	20	20	25.56	**Fair**
Newman et al. (2018)	30	30	30	10	30	30	30	20	40	27.78	**Fair**
Nielsen et al. (2018)	30	20	40	20	30	20	40	20	30	27.78	**Fair**
Nijman et al. (2011)	30	30	20	30	40	20	40	30	20	28.89	**Fair**
Nijman et al. (2005)	30	30	30	20	40	20	40	20	30	28.89	**Fair**
Niu et al. (2019)	40	30	40	30	40	30	40	30	30	34.44	**Good**
Nolan et al. (2009)	30	20	20	20	30	30	40	30	30	27.78	**Fair**
Nurjannah, FitzGerald & Foster	20	20	30	20	40	30	40	20	40	28.89	**Fair**

(2009)											
O'Donovan (2007)	20	20	30	20	30	30	30	20	30	25.56	**Fair**
O'Brien & Cole (2004)	20	20	30	20	40	30	40	20	30	27.78	**Fair**
Olsson & Schon (2016)	30	20	30	20	30	20	40	20	30	26.67	**Fair**
Olsson et al. (2015)	30	30	40	30	40	30	40	30	20	28.89	**Fair**
O'Neill et al. (2003)	30	30	30	20	30	20	30	20	20	25.56	**Fair**
Papadopoulos et al. (2012)	30	30	30	30	30	20	40	30	40	31.11	**Good**
Park & Lee (2012)	20	30	30	30	30	20	40	20	40	28.89	**Fair**
Paschali et al. (2017)	30	20		20	40	20	20	20	30	25.00	**Fair**
Pekurinen et al (2017)	40	30	40	20	40	20	40	20	40	32.22	**Good**
Pellfolk et al. (2010)	40	20	30	30	30	20	40	30	30	30.00	**Good**
Pelto-Piri et al. (2019)	40	30	40	20	40	20	40	20	30	31.11	**Good**
Perkins et al. (2012)	20	20	20	20	40	20	30	20	20	23.33	**Fair**
Pfeiffer et al. (2019)	30	40	30	30	20	20	30	30	30	28.89	**Fair**
Phillips (2011)	20	30	20	20	30	20	30	20	30	24.44	**Fair**
Podubinski et al. (2017)	40	40	40	40	40	40	40	40	30	38.89	**Good**
Powell-Cope et al. (2014)	40	30	30	20	30	30	40	20	40	31.11	**Good**
Price et al. (2018)	40	20	30	20	40	20	40	20	30	28.89	**Fair**
Prins et al. (2013)	40	20	40	20	30	40	40	20	20	30.00	**Good**
Quirk, et al. (2005)	40	30	30	10	20	20	30	20	30	25.56	**Fair**
Raboch et al.	30	20	30	30	30	30	30	30	30	28.89	**Fair**

(2010)											
Raveendranathan, Chandra & Chaturvedi (2012)	30	20	30	30	10	20	30	30	30	25.56	**Fair**
Raveesh et al (2019)	40	30	30	40	30	20	40	40	30	33.33	**Good**
Rees & Manthorpe (2010)	30	20	30	20	20	30	30	20	30	25.56	**Fair**
Reininghaus et al. (2007)	20	10	30	20	30	20	30	20	30	23.33	**Fair**
Reisch et al (2018)	30	20	30	20	30	20	20	20	30	24.44	**Fair**
Reynolds et al. (2016)	30	30	30	20	10	10	30	20	30	23.33	**Fair**
Rippon et al (2018)	30	30	20	10	40	10	40	20	20	24.44	**Fair**
Rodreguez-Leal et al. (2016)	30	20	30	40	20	10	30	30	30	26.67	**Fair**
Ružić et al. (2011)	20	20	30	20	30	20	40	20	20	24.44	**Fair**
Ryan & Bowers (2005)	30	30	40	20	30	20	30	20	30	27.78	**Fair**
Ryan & Happell (2009)	30	20	30	30	30	30	40	30	30	30.00	**Good**
Ryan et al. (2004)	40	20	30	30	30	20	40	30	20	28.89	**Fair**
Ryan et al. (2007)	30	30	30	30	40	20	30	30	20	28.89	**Fair**
Salzmann-Erikson et al. (2008)	30	20	30	20	40	20	40	20	20	26.67	**Fair**
Sandy (2016)	40	30	40	40	20	30	30	20	30	31.11	**Good**
Sansone, McLean & Wiederman	30	10	30	40	20	20	30	30	20	25.56	**Fair**

(2008)											
Sato et al. (2017)	30	30	30	30	40	20	40	30	30	31.11	**Good**
Schreiner, Crafton & Sevin (2004)	20	10	20	20	30	30	30	20	30	23.33	**Fair**
Schwartz & Park (1999)	40	30	20	20	20	20	40	20	20	25.56	**Fair**
Seemüller et al. (2009)	40	40	30	30	40	40	40	30	20	34.44	**Good**
Selenius & Strand (2017)	30	30	40	30	40	20	40	30	30	32.22	**Good**
Seo, Kim & Rhee (2012)	30	20	20	20	20	10	20	10	20	18.89	**Poor**
Sequeira & Halstead (2001)	20	10	20	20	20	30	30	20	30	22.22	**Fair**
Sequeira & Halstead (2004)	30	20	20	20	40	30	40	20	30	27.78	**Fair**
Shaw & Sandy (2016)	30	40	40	40	20	20	30	30	30	31.11	**Good**
Silvana et al. (2012)	20	20	30	10	20	20	30	10	20	20.00	**Fair**
Simpson et al. (2011)	30	20	30	10	40	30	30	10	30	25.56	**Fair**
Simpson et al. (2015)	40	30	30	20	40	30	40	20	40	32.22	**Good**
Sival et al. (2000)	40	20	30	20	40	30	40	20	20	28.89	**Fair**
Sjöstrand et al. (2015)	40	20	30	20	40	20	40	20	20	27.78	**Fair**
Sjöström et al. (2001)	10	10	30	20	30	30	40	20	30	24.44	**Fair**
Sjöström, Hetta & Waern (2012)	30	30	30	30	30	20	40	30	30	30.00	**Good**
Skovdahl, Kihlgren &	20	10	40	20	40	40	30	20	30	27.78	**Fair**

Kihlgren (2003)											
Smith & Jones (2014)	20	20	20	20	30	30	40	20	30	25.56	**Fair**
Sørensen et al. (2013)	30	20	30	30	40	30	40	30	20	30.00	**Fair**
Speziale et al. (2009)	30	20	30	20	20	30	40	20	30	26.67	**Fair**
Spokes et al. (2002)	20	20	30	30	20	20	30	30	30	25.56	**Fair**
Stead et al. (2009)	30	20	30	20	20	20	30	20	30	24.44	**Fair**
Stein (2002)	20	30	30	20	30	30	30	20	30	26.67	**Fair**
Steinert et al. (2007)	30	20	30	10	30	40	30	10	30	25.56	**Fair**
Stevenson, Jack, O'Mara & LeGris (2015)	40	30	40	30	30	40	40	30	30	34.44	**Good**
Stolker, Nijman & Zwanikken (2006)	30	30	30	30	30	30	40	30	30	31.11	**Good**
Stone et al. (2011)	30	30	30	20	40	20	40	20	30	28.89	**Fair**
Strudwick et al. (2017)	30	30	30	20	40	30	40	20	40	31.11	**Good**
Stübner et al. (2006)	40	20	30	20	20	20	30	20	30	25.56	**Fair**
Sukhodolsky, Cardona & Martin (2005)	30	30	30	30	40	20	40	30	30	31.11	**Good**
Sun et al. (2005)	30	20	30	20	40	20	30	20	30	26.67	**Fair**
Sun et al. (2006)	30	30	30	20	40	30	40	20	20	28.89	**Fair**
Sustere & Tarpey (2019)	20	40	30	10	30	20	40	20	20	25.56	**Fair**
Sutton et al. (2013)	30	20	20	30	40	20	30	30	30	27.78	**Fair**

Swogger, Van Orden & Conner (2014)	20	20	30	30	30	0	30	30	30	24.44	**Fair**
Takahashi et al. (2011)	30	20	30	20	30	30	40	20	20	26.67	**Fair**
Tängman et al. (2010)	30	20	30	20	30	20	40	20	20	25.56	**Fair**
Tateno et al. (2009)	40	20	30	20	20	20	40	20	20	25.56	**Fair**
Tema, Poggenpoel & Myburgh (2011)	30	30	20	20	30	30	40	20	30	27.78	**Fair**
Terkelsen & Larsen (2016)	30	40	30	20	30	40	40	20	30	31.11	**Good**
Thomas & Haslam (2017)	30	30	30	10	30	30	40	10	40	27.78	**Fair**
Tofthagen, Talseth & Fagerström (2014)	30	30	30	20	30	20	40	20	20	26.67	**Fair**
Tomagova et al (2016)	30	40	40	20	30	20	30	20	40	30.00	**Good**
Tompsett, Domoff & Boxer (2011)	40	30	30	30	30	20	30	30	30	30.00	**Good**
Trenoweth (2003)	30	20	20	20	30	30	40	20	30	26.67	**Fair**
Triplett et al. (2017)	40	30	30	10	40	20	40	20	40	30.00	**Good**
Truea et al. (2017)	30	30	30	40	30	20	30	30	30	30.00	**Good**
Umut et al. (2012)	30	20	20	20	30	30	40	20	20	25.56	**Fair**
Vahidi et al. (2018)	30	30	40	30	30	30	30	30	30	31.11	**Good**
Van de Sande et al. (2011)	40	20	30	20	40	40	40	20	30	31.11	**Good**

van den Bogaard et al. (2018)	30	30	40	20	10	20	30	20	30	25.56	**Fair**
Van der Schaaf et al. (2013)	30	30	40	30	40	10	40	30	30	31.11	**Good**
Van Wijk, Traut & Julie (2014)	30	20	30	30	30	30	30	30	30	28.89	**Fair**
Vandewalle et al. (2018)	30	40	40	30	40	40	30	30	30	34.44	**Good**
Vandewalle et al. (2019)	40	20	30	20	40	30	40	20	30	30.00	**Good**
Vedana et al (2018)	30	20	20	20	20	20	20	20	30	22.22	**Fair**
Verbeek et al. (2014)	40	20	40	30	30	30	30	30	30	31.11	**Good**
Vlayen et al. (2012)	40	30	30	20	30	30	30	20	40	30.00	**Good**
Vråle & Steen (2005)	20	20	20	20	30	30	40	20	30	25.56	**Fair**
Ward (2013)	20	30	20	20	40	30	30	20	30	26.67	**Fair**
Weber (2002)	30	20	20	30	40	10	30	30	20	25.56	**Fair**
Whaley (2001)	20	20	20	20	20	10	30	20	30	21.11	**Fair**
Whaley (2004)	20	20	20	30	20	10	20	30	30	22.22	**Fair**
Wharewera-Mika et al. (2016)	30	20	20	10	20	20	40	20	30	23.33	**Fair**
Whitecross, Seeary & Lee (2013)	40	20	30	20	30	20	40	20	30	27.78	**Fair**
Whittington et al. (2009)	40	20	20	20	20	20	20	20	20	22.22	**Fair**
Wilkes et al. (2005)	30	20	20	30	20	20	30	30	30	25.56	**Fair**
Wilson et al (2018)	40	30	30	20	40	30	40	20	30	31.11	**Good**
Wilson et al. (2017)	30	30	30	20	30	20	20	30	30	26.67	**Fair**

Wood & Pistrang (2004)	30	20	20	20	30	0	40	20	30	23.33	**Fair**
Woods (2013)	20	20	30	20	30	30	40	20	30	26.67	**Fair**
Wright et al. (2005)	40	30	30	30	0	30	40	30	30	28.89	**Fair**
Wright et al. (2014)	10	20	10	20	10	20	30	20	30	18.89	**Poor**
Wu et al. (2015)	30	30	30	20	40	30	40	20	40	31.11	**Good**
Wystanski (2000)	30	10	20	30	20	0	30	20	10	18.89	**Poor**
Xie et al. (2019)	30	30	40	20	40	20	40	20	30	30.00	**Good**
Yang et al. (2007)	30	20	40	20	30	20	30	20	30	26.67	**Fair**
Yip et al. (2013)	40	30	30	30	40	30	40	30	30	33.33	**Good**
Zuzelo, Curran & Zeserman (2012)	30	20	30	20	40	30	40	20	40	30.00	**Good**
Zwijsen et al. (2014)	40	30	40	30	40	40	40	30	40	36.67	**Good**
