# Supplementary material for: Patient safety in inpatient mental health settings: a systematic review
Source: BMJ Open. 2019 Dec 23;9(12):e030230. doi: 10.1136/bmjopen-2019-030230 (PMC7008434; doi:10.1136/bmjopen-2019-030230)

Online supplement 6 – Forest plots

Prevalence of physical aggression

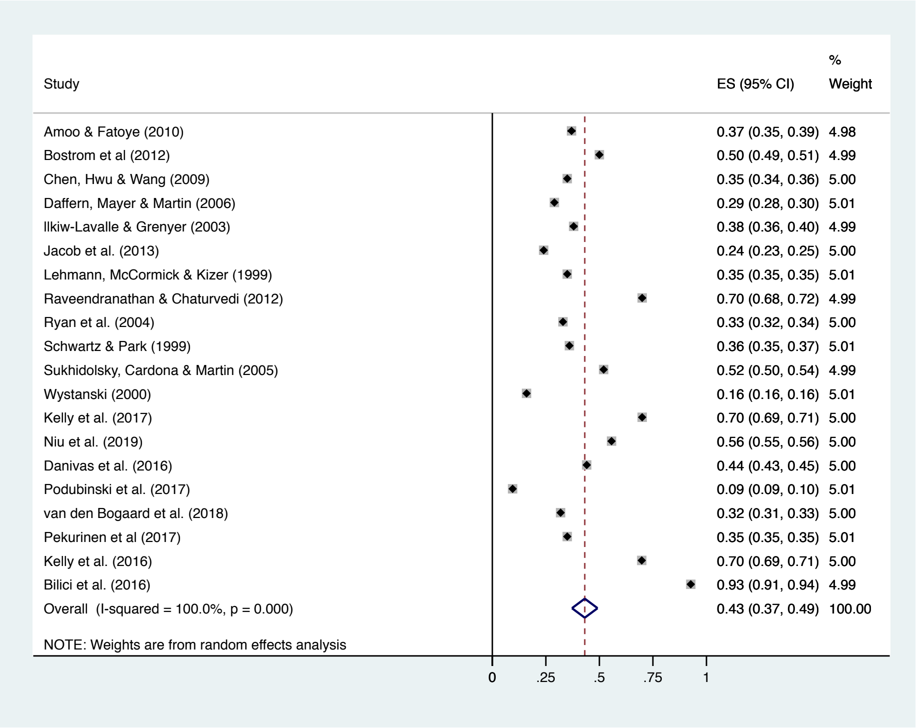

Prevalence of verbal aggression

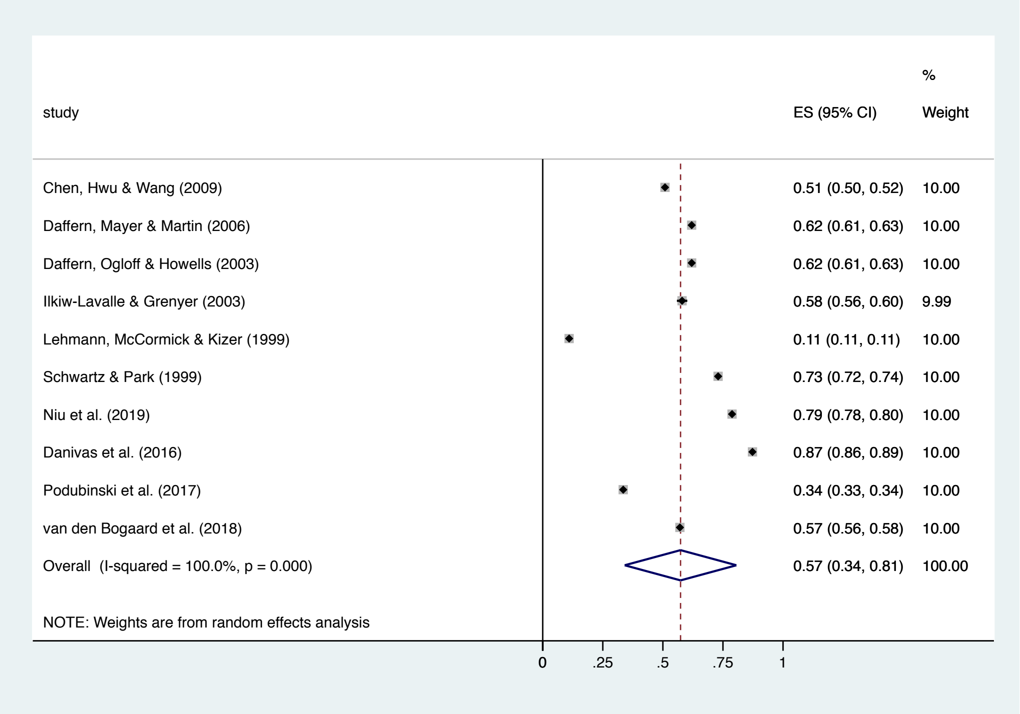

Use of coercive interventions

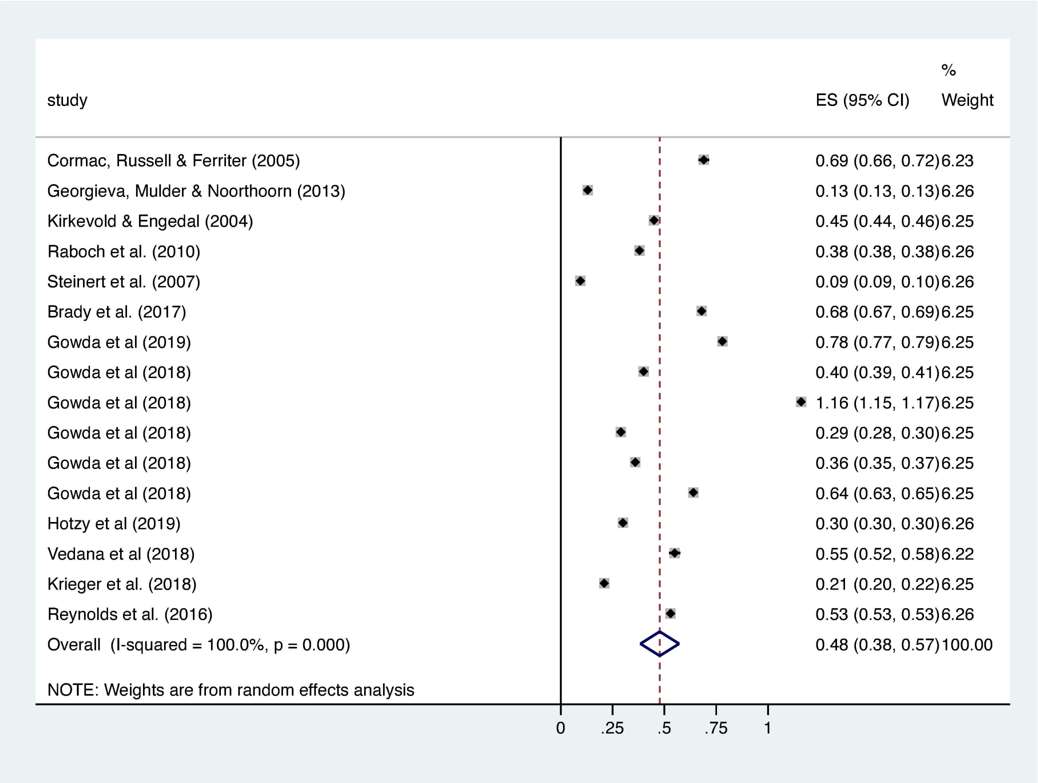

Prevalence of wandering behaviour

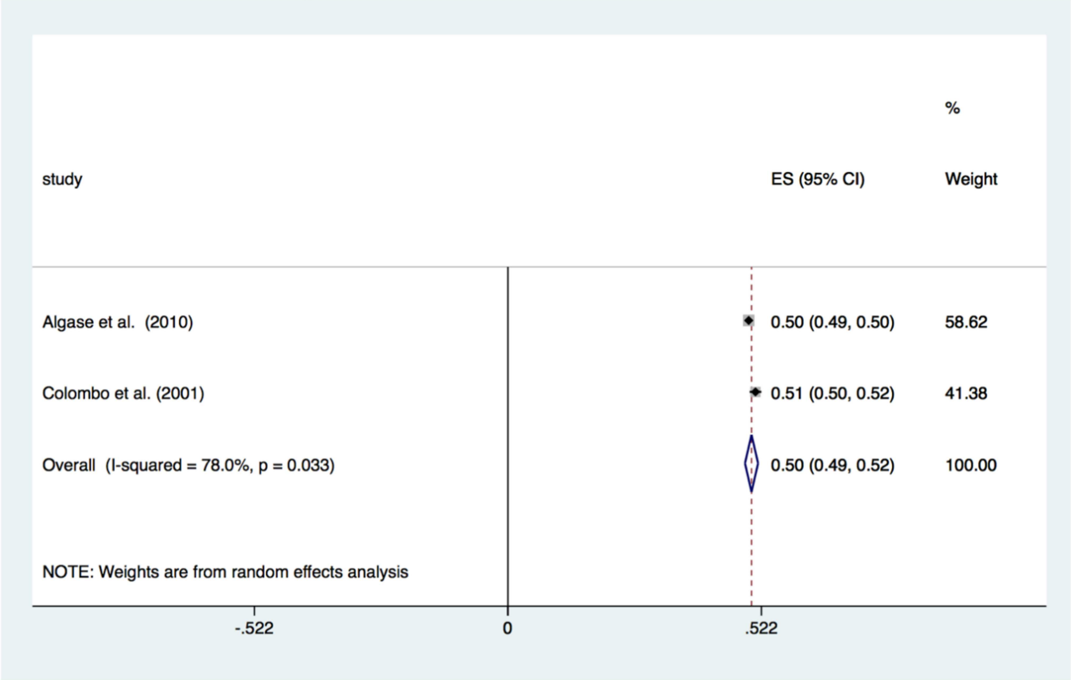

Supplement: Supplementary data [file bmjopen-2019-030230supp006.pdf]
