# Supplementary material for: Patient safety in inpatient mental health settings: a systematic review
Source: BMJ Open. 2019 Dec 23;9(12):e030230. doi: 10.1136/bmjopen-2019-030230 (PMC7008434; doi:10.1136/bmjopen-2019-030230)
Supplement: Author's manuscript [file bmjopen-2019-030230.draft_revisions.pdf]

BMJ Open

BMJ Open is committed to open peer review. As part of this commitment we make the peer review history of every article we publish publicly available.

When an article is published we post the peer reviewers' comments and the authors' responses online. We also post the versions of the paper that were used during peer review. These are the versions that the peer review comments apply to.

The versions of the paper that follow are the versions that were submitted during the peer review process. They are not the versions of record or the final published versions. They should not be cited or distributed as the published version of this manuscript.

BMJ Open is an open access journal and the full, final, typeset and author-corrected version of record of the manuscript is available on our site with no access controls, subscription charges or pay-per-view fees (http://bmjopen.bmj.com).

If you have any questions on BMJ Open's open peer review process please email info.bmjopen@bmj.com

BMJ Open

Patient safety in inpatient mental health settings: a systematic review

Journal:	*BMJ Open*
Manuscript ID	bmjopen-2019-030230
Article Type:	Original research
Date Submitted by the Author:	05-Mar-2019
Complete List of Authors:	Thibaut, Bethan; Imperial College London<br>Dewa, Lindsay; Imperial College London<br>Ramtale, Sonny; Imperial College London<br>D'Lima, Danielle; University College London<br>Adam, Sheila; Imperial College London<br>Ashrafian, Hutan; Imperial College London<br>Darzi, Ara; Imperial College London<br>Archer, Stephanie; Imperial College London; University of Cambridge
Keywords:	Patient safety, MENTAL HEALTH, Inpatient settings, Systematic Review

SCHOLARONE™
Manuscripts

Patient safety in inpatient mental health settings: a systematic review

Authors: Bethan Thibaut[1,2*], Lindsay H. Dewa[1,2*], Sonny Christian Ramtale[1], Danielle D'Lima[3], Sheila Adam[1], Hutan Ashrafian[4], Ara Darzi[1,4] and Stephanie Archer[1]

[1]NIHR Imperial Patient Safety Translational Research Centre, Imperial College London, London, UK

[2] School of Public Health, Imperial College London, London, UK

[3] Centre for Behaviour Change, Department of Clinical, Educational and Health Psychology, London, UK

[4] Department of Surgery and Cancer, St Mary's Hospital, Imperial College London, London, UK

*Bethan Thibaut and Dr Lindsay Dewa should be considered joint first author

Corresponding author: Dr Stephanie Archer, NIHR Patient Safety Translational Research Centre, Imperial College London, London, UK; stephanie.archer@imperial.ac.uk

For peer review only

For peer review only - http://bmjopen.bmj.com/site/about/guidelines.xhtml

Abstract

Objectives: Patients in inpatient mental health settings face similar risks as those in other areas of health care (e.g. medication errors). In addition, some unsafe behaviours associated with serious mental health problems (e.g. self-harm), and the measures taken to address these (e.g. restraint), may result in further risks to patient safety. The objective of this review is to collate and describe research on patient safety in inpatient mental health settings.

Design: Systematic review and meta-synthesis. Embase, Cumulative Index to Nursing and Allied Health Literature (CINAHL), Health Management Information Consortium (HMIC), MEDLINE, PsycINFO and Web of Science were systematically searched from 1999 to 2016. Search terms were related to “mental health”, “patient safety”, “inpatient setting” and “research”. Study quality was assessed using the Hawker checklist. Data was extracted and grouped based on study focus and outcome. Safety incidents were meta-analysed where possible using a random effects model.

Results: Of the 37,140 article titles and abstracts, 270 met inclusion criteria. Included publications came from 30 countries and included data from over 130,000 participants. Study quality varied and statistical heterogeneity was high. Nine research categories were identified: interpersonal violence, coercive interventions, safety culture, harm to self, safety of the physical environment, medication safety, unauthorised leave, clinical decision making and accidents.

Conclusions: Research on patient safety in inpatient mental health settings is under researched in comparison to other non-mental health inpatient settings. Findings demonstrate that inpatient mental health settings pose unique challenges for patient safety which require investment in research, policy development, and translation into clinical practice.

PROSPERO registration: CRD42016034057

Key words

Patient safety, Mental health, Inpatient settings, Review

For peer review only

Article Summary

This is the first review to examine patient safety within inpatient mental health settings that uses robust systematic methodology.

The use of a robust patient safety taxonomy provides a comprehensive list of all incident types and resulted in a wide coverage of publications in terms of setting, country and population.

This review only included peer reviewed studies with primary data.

The last systematic literature search was conducted on 5th April 2016, meaning that literature published since this date will not have been included.

For peer review only

Introduction

Patient safety is the "avoidance, prevention and amelioration of adverse outcomes or injuries stemming from the process of healthcare" [1]. Those receiving care in inpatient mental health settings face similar risks as patients in other areas of health care (e.g. medication errors). In addition, some of the unsafe behaviours associated with serious mental health problems (e.g. self-harm), and the measures taken to address these (e.g. restraint), may result in further risks to patient safety[2–6]. There may also be a tension between maximising patient safety and maintaining patient autonomy. Inpatient services will often include those patients who are most unwell and therefore at greatest risk.

Whilst mental health research has focused on components of quality of care, published research lacks focus on the science of patient safety [7,8,9]; the stigma and discrimination associated with mental health problems may contribute to this relative neglect [7]. Only two reviews have examined patient safety in a mental health context and described factors that influence patient safety [7,10]. These reviews highlighted the complexity of patient safety in mental health, including the importance of wider organisational safety culture. Whilst these reviews offer important insights into this complex topic, only a small number of specific patient safety incidents and concepts were examined. As such, the current breadth and depth of patient safety research in inpatient mental health settings is unknown.

The review presented here is exploratory in nature; building on previous reviews, we aimed to report an overview of the existing research base on patient safety in inpatient mental health settings. We also aimed to critically reflect on quality and methods used in included studies in the field [11]. In addition to our original protocol [11], we aimed to collate, describe and construct the main research categories, allowing for an easily accessible reference index.

Search strategy and selection criteria

A systematic search was developed and deployed in relation to the Preferred Reporting Items for Systematic Reviews and Meta-Analyses (PRISMA) guidelines [12]. The protocol for this systematic review was registered with PROSPERO (2016: CRD42016034057) and has been published elsewhere [11].

Six databases were searched: Embase, Cumulative Index to Nursing and Allied Health Literature (CINAHL), Health Management Information Consortium (HMIC), MEDLINE, PsycINFO and Web of Science. The search was conducted on the 5th April 2016. Search terms were related to "mental health", "patient safety", "inpatient setting" and "research" (see online supplement 1 for full search terms). The search terms included in the 'patient safety'

For peer review only - http://bmjopen.bmj.com/site/about/guidelines.xhtml

facet were based on the National Reporting and Learning System (NRLS) taxonomy for England and Wales [13] to ensure all incident types were identified in the search. A Google Scholar search using the main search terms was also conducted; it was originally anticipated that the first 20 pages of Google scholar would need to be screened against criteria [11] but screening stopped at five pages as no new publications were retrieved. Similarly, we had anticipated to hand-search references of all included papers within the review. However, due to the large amount of literature included in the review, only the reference lists of the two existing systematic reviews were searched for additional references.

One reviewer (BT) screened all titles against the inclusion and exclusion criteria, with 10% independently screened by a second reviewer (split equally between CR, DD or LD). Full definitions and descriptions of these criteria can be found in online supplement 1 and the protocol published elsewhere[11]. Inclusion and exclusion criteria were developed over several iterative rounds amongst the research team to ensure consistency between reviewers (online supplement 2). Any disagreements between reviewers were resolved through discussion and an overall consensus was obtained; a third reviewer (SAr) was consulted if necessary. Agreement rates were calculated using Cohen's kappa. Full text papers were assessed for inclusion by two reviewers from the research team (BT and one other from CR, DD and LD).

Inclusion criteria:

- Population: Mental health inpatients
- Intervention/outcomes: Patient safety outcomes
- Setting: Inpatient setting
- Comparators: No restriction
- General inclusion criteria: Empirical peer reviewed studies with a clear aim or research question, that used primary data and written up in the English language between January 1st 1999 and April 5th 2016 (in line with the publication of the Institute of Medicine's report "To Err is Human: Building a Safer Health System")[14]

Exclusion criteria:

- Population: Centres on physical healthcare services
- Intervention/outcomes: Patient safety was not the central aim, research question or outcome; amalgamation of data from inpatient and outpatient settings (where inpatient sample cannot be separated out); primary care, outpatient mental health services, community or social care settings, and risk assessment tool reliability/validity checks

For peer review only - http://bmjopen.bmj.com/site/about/guidelines.xhtml

- Comparators: No restrictions
- General exclusion criteria: Secondary data, not in English language, protocols, editorials, commentaries/clinical case reviews/'snapshot' studies of a patient group, book chapters, conference abstracts, audits, dissertations, epidemiological studies and reviews.

Quality Assessment

Quality was assessed by three reviewers (BT, CR and LD) using the tool derived by Hawker et al.[15], to allow appropriate assessment of the wide variety of studies included in this review. Hawker's checklist evaluates nine domains: 1) abstract/title; 2) introduction and aims; 3) method and data; 4) sampling; 5) data analysis; 6) ethics and bias; 7) results; 8) transferability and generalizability; and 9) implications and usefulness. For each study the nine domains were assessed using one of four quality categories: very poor (10 points), poor (20 points), fair (30 points) and good (40 points). The scores for each study were then summed and divided by nine to get an average score. Publications were not excluded based on poor quality because the review was purposively exploratory and all-encompassing.

Data extraction

Data was extracted by three reviewers (BT, CR and LD) using a standardised form that included study design information, participant characteristics, intervention description and patient safety outcomes. Extractions were compared within the research team to ensure reliability. Only published data was extracted; authors were contacted only for confirmation or information clarity. If the contact attempt was unsuccessful, the article was assessed in its current form.

Data synthesis

Studies were grouped into research categories through group consensus. Firstly, three research team members (BT, CR and LD) individually re-read the included full-text publications and assigned each one based on the main topic area (e.g. aggression). Secondly, each assigned topic area was subsequently checked by another team member to ensure reliability. Thirdly, topic areas were grouped into broader research categories (e.g. interpersonal violence) that best described the patient safety focus for easier navigation of the vast body of literature. Finally, these categories and the related subcategories (initially called topic areas) from the previous stage were finalised after group discussion and consensus was reached. This was to ensure mutual exclusivity and appropriate definition (Table 1 and online supplement 3). Where data allowed, meta-analysis was performed applying a random-effects model, specifically calculating pooled prevalence considering both

For peer review only - http://bmjopen.bmj.com/site/about/guidelines.xhtml

between-study and within-study variances that contributed to study weighting. Pooled values and 95%CIs were computed and represented on forrest plots. Statistical heterogeneity was determined by the I2 statistic; where <30% is low, 30-60% is moderate and >60% is high. Analyses were performed using Stata version 15 (StataCorp LP, College Station, TX) (online supplement 5).

Patient and Public Involvement

Patients and the public were not involved in this study.

Results

The search resulted in 59,172 records (Figure 1) and reduced to 37,140 after de-duplication. Title and abstracts were screened and excluded if they did not satisfy inclusion criteria (BT). Ten percent were then screened (n=3714) by a second independent reviewer (split equally between CR, DD and LD), in line with guidance on improving decision making by including more than one person in this process[16]; good agreement was found between pairs of reviewers (κ=0.72). A total of 4,249 publications were subjected to full-text review (BT). Two independent reviewers further screened the full text articles against inclusion criteria (CR, DD or LD). Substantial agreement was reached (κ=0.64). From the full text review, 3,979 publications were excluded. Two-hundred and seventy publications met the inclusion criteria and data was extracted (online supplement 4).

<Insert Figure 1 here>

Study characteristics

Table 1 provides a study characteristics overview. The publications spanned five continents and 30 countries. The three countries contributing the greatest number of studies were the UK (n=78), the USA (n=44) and Australia (n=25). The included studies collected data from over 130,000 participants. Studies included staff (n=117; 43%), patients (n=100; 37%) and a mixture of staff and patients (n=52; 19%). Only one study focused on patient family members (<1%). Most studies were quantitative in nature (n=151; 56%), a third were qualitative (n=91; 34%) and a small proportion used mixed methodology (n=28; 10%). Studies were conducted in a variety of settings comprising: psychiatric inpatient wards/facilities (n=190), forensic inpatient facilities (n=44), long-term care/nursing homes (n=25), mixed inpatient settings (n=10) and specialised research unit (n=1). More information is included about the study designs used in the included papers in online supplement 2.

For peer review only - http://bmjopen.bmj.com/site/about/guidelines.xhtml

Quality assessment

Most research was assessed as being of ‘Fair’ quality (n=203; 75%); 42 (16%) papers were assessed as ‘Good’ quality, and 24 (9%) were assessed as ‘Poor’ quality. None were assessed as being of ‘Very Poor’ quality. Studies rated as 'Poor’ mainly did not discuss ethical considerations, potential biases or give sample or setting characteristics. For example, they did not consider recruitment strategies, sample demographics or structure of the research settings. All ‘Good’ studies provided setting and sampling information to allow for replicability. In addition, ‘Good’ studies provided detail on data analysis justification, more thorough literature reviews to place the study in context and had clear research aims/objectives.

Synthesis

Nine research categories were identified: interpersonal violence, coercive interventions, safety culture, harm to self, safety of the physical environment, medication safety, unauthorised leave, clinical decision making, and accidents. Within these categories 43 subcategories were identified (see Table 1).

<Insert Table 1 here>

Interpersonal violence

Interpersonal violence was the largest category (n=91; 34%), including nine subcategories. Studies were primarily concerned with the prevalence, management and prevention of violent and aggressive behaviours (n=71). The pooled prevalence for physical violence was 37.9% (95%CI 0.31-0.45) with high heterogeneity (I^2 100.0%) in 12 studies (online supplement 5) [17–28]. The pooled prevalence for verbal aggression was 52.8% (95%CI 0.23-0.83) with a high heterogeneity (I^2 100.0%) in 6 studies [21–23,25,28,29] (online supplement 5).

Eighteen studies evaluated intervention effectiveness (e.g. staff training and medication use) to reduce violent and aggressive behaviours, with most finding significant improvements [30–44], two reporting negative outcomes [45,46], and one reporting mixed findings [47]. The general management of violent and aggressive behaviours was explored in 15 studies [19,21,24,28,48–58]. Two studies explored the ways in which treatment can affect violence incidence [20,59]. Twenty-five studies explored violent and aggressive incident experiences in staff [60–72], patients [73–75], mixed groups [23,76–82] and patient family members [26]. Three studies explored mental health nurses’ perspectives on the response to violent situations in high secure

For peer review only - http://bmjopen.bmj.com/site/about/guidelines.xhtml

environments: one on the psychological impact of physical assault on staff [83], one on making violence risk assessments in imminent violent situations [84] and one on the decline of incident reports [85]. Ten studies [86–95] examined challenging behaviour and techniques, such as de-escalation and communication strategies, that could be used to manage this; seven studies found techniques that were effective [86–92]. A further three studies investigated conflict behaviour management techniques employed by staff [96,97] and patients [98]; all techniques used in the two intervention studies were effective in reducing conflict [96,98]. Staff and patient attitudes towards critical incidents were the focus of three qualitative studies [99–101]; a further two studies focused on maintaining the patient psychological safety who had undergone sexual assault, during an inpatient stay [102] and outside of healthcare [103]. One study explored an acupressure intervention to reduce agitation, which was found to be effective [104]. Finally, one study investigated previously abused female patients' views, regarding their safety and the inpatient physical environment [105].

Coercive interventions

Coercive interventions were the focus of 64 papers (24%). Most studies (n=42) reported on restraint and seclusion techniques. The pooled prevalence for coercive interventions was 34.9% (95%CI 0.21-0.49) with high heterogeneity (I^2 100.0%) in 5 studies [106–110] (online supplement 5).

Studies explored staff [111–117], patient [118–125], and mixed groups' [126,127] views and experiences of seclusion and restraint using predominantly qualitative methodology (n=17). Nine studies focused on seclusion and restraint processes management [106,107,128–134]. A further nine studies evaluated interventions to reduce seclusion and restraint, with eight finding significant decreases in rates of use [135–142], and one reporting an increase [143]. Four studies examined prevalence, trends and preventative factors [108,144–146]; one found that 45% of patients were subjected to restraint [108], and another found that restraint and seclusion declined over time [146]. Two studies found preventative factors of mechanical restraint to be staff education and increased patient involvement [144,145]. The training of staff in techniques for seclusion and restraint were explored in two studies [147,148] and one study examined adverse events resulting from restraint and seclusion[149]. Other studies explored staff and patients views towards containment measures [150–153], the process of shielding[1] [154], conflict management [155] and alternative interventions [156,157]. Fourteen studies focused on coercion; the attitudes of staff [158–162], patients [163–165], and mixed groups [166,167] towards coercion were

[1] Segregation under staff supervision

For peer review only - http://bmjopen.bmj.com/site/about/guidelines.xhtml

explored in ten studies, and four studies examined the process of coercive interventions [109,110,168] and rules of engagement in caring for aggressive patients [169].

Safety culture

Safety culture included studies on process, culture and policy across 37 papers (14%). Fifteen studies concerned safety-related organisational processes. Ten of these investigated processes of treatment or care that healthcare staff undertake; processes included limit-setting and clothing restrictions [170–176] and risk assessment [177–179]. Two investigated errors and reporting [180,181] and a further two studies explored staff and patient perceptions of safety when involved in treatment processes [182,183]. One of these studies focused on change implementation [184]. Safety culture was featured in 14 publications relating to the management of serious incidents [185–187], stress and burnout [188–190], patient perspectives of safety [191,192] and communication [193]; there were also three papers that explored safety culture more generally [194–196]. A further two evaluated the TeamSTEPPS (Team Strategies and Tools to Enhance Performance and Patient Safety) programme [197,198] and both found significant clinical benefits in reducing seclusion and improving team functioning. With regards to policy, eight studies concerned safety policies related to: observation [199,200], risk assessment [201,202], treatment [203], safeguarding [204], security [205] and ergonomic improvement [206].

Harm to Self

Three subcategories centred on harmful behaviours: self-harm, suicidal behaviour and self-neglect (n=26; 10%). Half of the studies (n=13) focused on self-harm. Two studies explored risk factors for self-harm [207,208] which included use of psychotropic medication. Four papers explored staff attitudes and experiences of managing this behaviour [209–212], and three explored patient experiences [213–215]. Three intervention studies focusing on training [216], therapy [217] and observation [218] all reported a reduction in self-harm behaviours and a further intervention focusing on training for staff resulted in positive attitude towards self-harm patients, greater closeness and improved self-efficacy [219]. Of the 12 papers that centred on suicidal behaviours, three studies investigated the observance of risk factors [220–222] and two intervention studies found significant reductions in suicide-related behaviours and cognitions [223,224]. An additional six papers explored staff [225–227], patients [228,229], and both staff and patient [230,231] views and attitudes towards suicidal behaviour. Finally, one study explored types of self-neglect behaviours in dementia patients [232] including functional difficulties, serious hygiene problems and safety risks.

Safety of the physical environment

For peer review only - http://bmjopen.bmj.com/site/about/guidelines.xhtml

Safety of the physical environment category included 16 papers (6%). Five studies investigated security measures (e.g. door locking) [233–237]. Three studies investigated the effects of the physical environmental design on the safety of treatment settings [238–240]. Three studies focused on transitions of care safety [241–243], with most based in dementia care settings. Three studies examined how the location of patients within the hospital setting can impact on safety, focusing on topics such as: privacy, female only wards and the use of segregated or combined ward/units [244–246]. The remaining two studies concerned staffing levels [247] and ligature points [248].

Medication safety

The medication safety category included 12 publications (4%). Four studies focused on adverse events, and examined antipsychotics side effects [249], antidepressants [250], and medication error reporting [251,252]. Two studies investigated errors occurring in broader medication management processes [253,254] and a further three studies focused on medication administration specifically [255–257]. The only intervention study aiming to reduce these errors [258] found that a new medication dispensing system did not have any significant impact on patient safety. One study exploring staff perceptions of illicit substance use reported concern over potential adverse effects on patient psychopathology and the negative impact on the staff-patient relationship [259]. One further study described the development of a medication adherence intervention for patients who are prescribed mood-stabilizing medication for bipolar disorder [260].

Unauthorised leave

Unauthorised leave included 10 publications; three explored the patient experience of absconding, specifically relating patient perspectives of treatment and involuntary commitment [261–263]. One study explored staff perspectives of absconding management techniques [264], and two studies evaluated interventions to reduce absconding rates; both were found to be effective [265,266]. Two studies focused on wandering behaviour in women with dementia, linking wandering to physical environment factors, such as light, sound, crowding [267] and falls [268]. The pooled prevalence in wandering behaviour was 50.2% (95%CI 0.49-0.52) with high heterogeneity (I^2 78.0%) (online supplement 5). The final two studies examined the consequences [269] and security measures surrounding absconding [270].

Clinical decision making

Clinical decision making accounted for 3% of the included publications (n=8). These publications covered the development of clinical judgements and decisions relating to incident management, risk assessment and diagnosis. Two studies explored the cultural

For peer review only - http://bmjopen.bmj.com/site/about/guidelines.xhtml

differences considered by clinicians in the diagnosis of African American patients [271,272]. Clinical decisions on whether to engage in restraint were explored in four studies [273–276] and two studies explored the variation in assessment and prediction of violence between staff and settings [277,278].

Accidents

Publications on accidents formed the smallest category within the review (n=6; 2%). Studies in this category focused on falls prevalence of falls experienced by older psychiatric inpatients with dementia and prevention/harm reduction techniques. A recurring risk factor for falling was found to be medication use [279–281]. Two fall prevention intervention studies did not provide significant benefits [282,283], and one study explored barriers and facilitators to such interventions [284].

Discussion

Main findings

This is the first review to examine patient safety within inpatient mental health settings that uses robust systematic methodology. As a result, we have identified nine research categories: interpersonal violence, coercive interventions, safety culture, harm to self, safety of the physical environment, medication safety, unauthorised leave, clinical decision making, and accidents. The areas of research highlighted in this review extend those found in previous reviews [7,10]. In addition, we have been able to include a meta-analysis of incidence and prevalence of aggression (verbal and physical), coercive intervention and wandering behaviour as well as providing an easily accessible reference index of literature in the inpatient mental health and patient safety domain.

We were concerned to see that only 270 papers were identified as a result of our comprehensive search. Although this can be seen as a large number of publications for a systematic review, it is a relatively small number to cover the care of a wide range of patients in a variety of inpatient mental health providers over a 17-year period (around 16 papers per year across all countries). Whilst important work not meeting our inclusion criteria (e.g. quality improvement initiatives) may have focused on patient safety in mental health, the lack of peer reviewed publications adds to the ongoing discussion surrounding the disparity in research focusing on patient safety in physical and mental healthcare [11]. In addition, there was a paucity of high-quality research in the area; three-quarters of the studies were considered to be ‘fair’, and two studies included in the meta-analysis were deemed ‘good’. ‘Poor’ studies most frequently did not have clear research aims and objectives, study details

For peer review only - http://bmjopen.bmj.com/site/about/guidelines.xhtml

were missing (e.g. sample(s) and setting(s) used) and they failed to discuss ethical and researcher bias issues. Some qualitative studies explored both staff and patients' perspectives, an important aspect of research, particularly when safety in this context is a relatively new area of knowledge. However, there was limited intervention research, particularly randomised controlled trials (RCTs); in the RCTs that were identified, sample sizes were mostly small.

The findings from the review also challenged our expectations in terms of breadth and depth of research. For example, we expected to find many publications on the prevention of suicide within inpatient settings due to the severity of harm. However, only one study that met inclusion criteria which discussed suicide in relation to ligature points [248]. A scoping review also found only this one study, suggesting a consistency of approach [285]. This indicates that whilst the prevention of suicide is a well-established aspect of patient safety, it is now reviewed routinely, using pre-existing and secondary data, rather than through empirical research.

Similarly, we were expecting to identify literature investigating the lack of integration between physical and mental healthcare[286], and the impact it has on patient safety. However, the need to prevent and manage co-existing physical ill health was not identified in the review. This is surprising as patients with serious mental illness are twice as likely to die prematurely and much more likely to develop long term conditions or become disabled, as those without serious mental illness [287]. This patient group are also vulnerable to asphyxiation during restraint and rapid tranquilization [288]. In addition, research on medication safety in inpatient mental health settings was also limited in this review. This unexpected considering two-thirds of patients with mental health problems are prescribed medication and are therefore potentially at risk of experiencing a medication safety incident. Research pertaining to accidents was also limited, contrasting with patient safety research within the physical health domain that includes a focus on slips, trips and falls [289].

Strengths and limitations

We used a robust patient safety taxonomy to provide a comprehensive list of all incident types. This resulted in a wide coverage of publications in terms of setting, country and population. We systematically searched, screened, extracted and appraised data. As a result, our systematic review draws together all relevant literature concerning patient safety within inpatient mental health settings, simultaneously operating as an index resource for clinicians and researchers.

For peer review only - http://bmjopen.bmj.com/site/about/guidelines.xhtml

There were several limitations. The last systematic literature search was conducted on 5th April 2016, meaning that literature published since this date will not have been included. In order to further build on the review published here, a *living* systematic review [290] (an ongoing updated summary of high-quality research), would continue to identify relevant literature in this area. In terms of the meta-analysis, there was expected statistical and methodological variability in studies, particularly for physical and verbal aggression. It is possible that this was due to the inclusion of different definitions of aggression, time periods and type of inpatient setting. In relations to agreement between reviewers (including the use of recommended piloting of inclusion and exclusion criteria within screening stage) [291], inter-rater reliability calculations only achieved substantial agreement (κ=0.61-0.80) at both the title and full-text screening stages. Whilst higher kappas have been reported in other systematic reviews, a substantial agreement is classified as more than acceptable [292].

Whilst the research spanned five continents, the UK, USA and Australia contributed over 50% of the included studies, leading to a potential cultural bias in the body of research identified within the review. We recommend that, where possible, future systematic reviews incorporate manuscripts in languages other than English to establish greater insight into the global literature on patient safety in inpatient mental health settings, with a view to limiting any cultural bias. Similarly, whilst the removal of publications denoting non-inpatient setting restricted the conclusions to the inpatient setting, issues pertaining to this environment are likely to be different to that of community, primary or social care settings. Additionally, studies were excluded before 1999 to coincide with the release of the Institute of Medicine's report "To Err is Human: Building a Safer Health System" [14]; this narrowed the review scope as the historical context was minimised.

This review only included peer reviewed studies with primary data. Therefore, literature utilising secondary data such as pre-existing datasets data from internal audits was excluded as it did not fulfil the criteria of being a prospective research study with clear research aims [293]. For example, data examined by the National Confidential Inquiry into Suicide and Homicide by People with Mental Illness (NCISH) is collected retrospectively from various sites across the country [294] and would have been excluded from this review. Moreover, non-peer reviewed quality improvement reports would also have been excluded as there was a need to define the scope and focus, due to the large number of potential publications in this area and the need for specificity as well as sensitivity. The investigation of research using secondary data or in non-peer reviewed formats in this field is an avenue for additional systematic reviews.

For peer review only - http://bmjopen.bmj.com/site/about/guidelines.xhtml

Clinical implications and future research

This review informs academics, clinicians and service providers about the evidence base in the patient safety field within inpatient mental health settings. The findings allow researchers and clinicians to be directed to literature relevant to a given patient safety topic area, a useful starting point when developing practice guidelines [295]. Similarly, the findings may influence clinical practice, with those implementing interventions or designing service changes being able to easily access the current scientific understanding.

Future research should be informed by patient safety science more broadly and focus on filling the knowledge gaps highlighted in this review i.e. studies that explore (i) patient and carer perspectives, (ii) safety culture, (iii) suicide prevention across different countries and (iv) the nature of medication safety in inpatient mental health settings. These findings support our recent expert consensus study where academic and service user experts agreed that patient driven research studies were needed [296]. The limited rigorous research surrounding patient safety within inpatient mental health settings necessitates future studies to: (i) include large inpatient samples relevant to the research design, (ii) perform appropriate intervention testing and (iii) examine safety from different perspectives. It should also focus on high quality reporting of research, paying particular attention to the area of ethics, sampling and setting characteristics.

Conclusion

This is the first systematic review to comprehensively examine research on patient safety within inpatient mental health settings. It has drawn together the existing literature and shed light on the gaps in knowledge. Although inpatient mental health settings may demonstrate unique patient safety challenges, more research is needed to achieve parity with physical health. Addressing this through a strong body of evidence, informed by patient science more broadly, will mean that mental healthcare policy makers are in a better position to address safety issues, and implement robust and evidence-based interventions to improve care.

Contributors

BT contributed to the design, data searches, data extraction, synthesis and writing of the report. LD, CD and DDL contributed to the design, data extraction, synthesis and writing of the report. HA contributed to the design, data extraction, meta synthesis and writing of the report. SAd, SAr and AD contributed to the design and synthesis, as well as writing and critically reviewing the report.

Declaration of interests

For peer review only - http://bmjopen.bmj.com/site/about/guidelines.xhtml

We declare no competing interests.

Acknowledgements
This article represents independent research supported by the National Institute for Health Research (NIHR) Imperial Patient Safety Translational Research Centre. The views expressed are those of the author(s) and not necessarily those of the NHS, the NIHR or the Department of Health. All authors had full access to all data in the study and had final responsibility for the decision to submit for publication.

For peer review only

For peer review only - http://bmjopen.bmj.com/site/about/guidelines.xhtml

References

1 Vincent, C. (2006). *Patient safety*. Edinburgh: Churchill Livingstone.

2 Brickell T, McLean C. Emerging issues and challenges for improving patient safety in mental health: a qualitative analysis of expert perspectives. *J Patient Saf* 2011; **7**: 39–44.

3 Goh SE, Salmons PH, Whittington RM. Hospital suicides: Are there preventable factors? Profile of the psychiatric hospital suicide. *Br J Psychiatry* 1989; **154**: 247–9.

4 Gray NS, Hill C, McGleish A, Timmons D, MacCulloch MJ, Snowden RJ. Prediction of violence and self-harm in mentally disordered offenders: a prospective study of the efficacy of HCR-20, PCL-R, and psychiatric symptomatology. *J Consult Clin Psychol* 2003; **71**: 443–51.

5 Powell J, Geddes J, Deeks J, Goldacre M, Hawton K. Suicide in psychiatric hospital in-patients: Risk factors and their predictive power. *Br J Psychiatry* 2000; **176**: 266–72.

6 Roy A, Draper R. Suicide among psychiatric hospital in-patients. 1995; *Psychological Medicine*; **25**: 199-202.

7 Brickell T, Nicholls T, Procyshyn R, McLean C, Dempster R, Lavoie J, *et al. Patient safety in mental health*. Edmonton, Alberta: Canadian Patient Safety Institute and Ontario Hospital Association. 2009.

8 Vincent GM. Psychopathy and violence risk assessment in youth. *Child Adolesc Psychiatr Clin N Am* 2006; **15**: 407–428.

9 Taylor TL, Killaspy H, Wright C, Turton P, White S, Kallert TW, *et al.* A systematic review of the international published literature relating to quality of institutional care for people with longer term mental health problems. *BMC Psychiatry* 2009; **9**: 55-85.

10 Kanerva A, Lammintakanen J, Kivinen T. Patient safety in psychiatric inpatient care: a literature review. *J Psychiatr Ment Health Nurs* 2013; **20**: 541–8.

11 D'Lima D, Archer S, Thibaut BI, Ramtale SC, Dewa LH, Darzi A. A systematic review of patient safety in mental health: a protocol based on the inpatient setting. *Syst Rev* 2016; **5**: 203-14.

12 Moher D, Shamseer L, Clarke M, Ghersi D, Liberati A, Petticrew M, *et al.* Preferred reporting items for systematic review and meta-analysis protocols (PRISMA-P) 2015 statement. *Syst Rev* 2015; **4**: 1-10.

13 National Patient Safety Agency. http://www.nrls.npsa.nhs.uk/patient-safety-data/. Accessed on 1st February 2016.

14 Donaldson, M. S., Corrigan, J. M., & Kohn, L. T. (Eds.) (2000). *To err is human: building a safer health system* (Vol. 6). National Academies Press.

For peer review only - http://bmjopen.bmj.com/site/about/guidelines.xhtml

15 Hawker S, Payne S, Kerr C, Hardey M, Powell J. Appraising the evidence: reviewing disparate data systematically. *Qual Health Res* 2002; **12**: 1284–99.

16 Edwards P, Clarke M, DiGuiseppi C, Pratap S, Roberts I, Wentz R. Identification of randomized controlled trials in systematic reviews: Accuracy and reliability of screening records. *Stat Med* 2002; **21**: 1635–40.

17 Amoo G, Fatoye FO. Aggressive behaviour and mental illness: A study of inpatients at Aro Neuropsychiatry Hospital, Abeokuta. *Niger J Clin Pract* 2010; **13**: 351–5.

18 Bostrom AM, Squires JE, Mitchell A, Sales AE, Estabrooks CA. Workplace aggression experienced by frontline staff in dementia care. *J Clin Nurs* 2012; **21**: 1453–65.

19 Sukhodolsky DG, Cardona L, Martin A. Characterizing aggressive and noncompliant behaviors in a children's psychiatric inpatient setting. *Child Psychiatry Hum Dev* 2005; **36**: 177–93.

20 Wystanski M. Assaultive behaviour in psychiatrically hospitalized elderly: A response to psychosocial stimulation and changes in pharmacotherapy. *Int J Geriatr Psychiatry* 2000; **15**: 582–5.

21 Chen WC, Hwu HG, Wang JD. Hospital Staff Responses to Workplace Violence in a Psychiatric Hospital in Taiwan. *Int J Occup Environ Health* 2009; **15**: 173–9.

22 Daffern M, Mayer M, Martin T. Staff gender ratio and aggression in a forensic psychiatric hospital. *Int J Ment Health Nurs* 2006; **15**: 93–9.

23 Ilkiw-Lavalle O, Grenyer BF. Differences between patient and staff perceptions of aggression in mental health units. *Psychiatr Serv* 2003; **54**: 389–93.

24 Jacob P, Seshadri S, Girimaji SC, Srinath S, Sagar J V. Clinical characteristics of aggression in children and adolescents admitted to a tertiary care centre. *Asian J Psychiatr* 2013; **6**: 556–9.

25 Lehmann LS, McCormick RA, Kizer KW. A survey of assaultive behavior in veterans health administration facilities. *Psychiatr Serv* 1999; **50**: 384–9.

26 Raveendranathan D, Chandra P, Chaturvedi S. Violence among psychiatric inpatients: A victim's perspective. *East Asian Arch Psychiatry* 2012; **22**: 141–5.

27 Ryan EP, Hart VS, Messick DL, Aaron J, Burnette M. A prospective study of assault against staff by youths in a state psychiatric hospital. *Psychiatr Serv* 2004; **55**: 665–70.

28 Schwartz TL, Park TL. Assaults by patients on psychiatric residents: A survey and training recommendations. *Psychiatr Serv* 1999; **50**: 381–3.

29 Daffern M, Ogloff J, Howells K. Aggression in an Australian forensic psychiatric hospital. *Br J Forensic Pract* 2003; **5**: 18–28.

30 Caspi N, Modai I, Barak P, Waisbourd A, Zbarsky H, Hirschmann S, *et al.* Pindolol

For peer review only - http://bmjopen.bmj.com/site/about/guidelines.xhtml

augmentation in aggressive schizophrenic patients: a double-blind crossover randomized study. *Int Clin Psychopharmacol* 2001; **16**: 111–5.

31 Coban Arguvanli S, Karatas N, Baser M, Zararsiz G. Effect of aggression management training program on knowledge and attitudes of nurses working at psychiatric clinics. *Anadolu Psikiyatr Dergisi-Anatolian J Psychiatry* 2015; **16**: 323–8.

32 Bjorkdahl A, Hansebo G, Palmstierna T. The influence of staff training on the violence prevention and management climate in psychiatric inpatient units. *J Psychiatr Ment Health Nurs* 2013; **20**: 396–404.

33 Calabro K, Mackey TA, Williams S. Evaluation of training designed to prevent and manage patient violence. *Issues Ment Health Nurs* 2002; **23**: 3–15.

34 Lanza M, Ridenour M, Hendricks S, Rierdan J, Zeiss R, Schmidt S, *et al.* The violence prevention community meeting: A multi-site study. *Arch Psychiatr Nurs Jan* 2016; **30**: 382-86.

35 Lanza ML, Rierdan J, Forester L, Zeiss RA. Reducing violence against nurses: the violence prevention community meeting. *Issues Ment Health Nurs* 2009; **30**: 745–50.

36 V CYY, Gudjonsson GH, Perkins D, Doidge A, Hopkin G, Young S. A non-randomised controlled trial of the R&R2MHP cognitive skills program in high risk male offenders with severe mental illness. *BMC Psychiatry* 2013; **13**: 267-78.

37 Daffern M, Howells K, Hamilton L, Mannion A, Howard R, Lilly M. The impact of structured risk assessments followed by management recommendations on aggression in patients with personality disorder. *J Forens Psychiatry Psychol* 2009; **20**: 661–79.

38 Giles GM, Wager J, Fong L, Waraich BS. Twenty-month effectiveness of a non-aversive, long-term, low-cost programme for persons with persisting neurobehavioural disability. *Brain Inj* 2005; **19**: 753–64.

39 Killick S, Allen D. Training staff in an adolescent inpatient psychiatric unit in positive approaches to managing aggressive and harmful behaviour: Does it improve confidence and knowledge? *Child Care Pract* 2005; **11**: 323–39.

40 McLaughlin S, Bonner G, Mboche C, Fairlie T. A pilot study to test an intervention for dealing with verbal aggression. *Br J Nurs* 2010; **19**: 489–94.

41 Murphy CJ, Siv AM. A one year study of Mode Deactivation Therapy: Adolescent residential patients with conduct and personality disorders. *Int J Behav Consult Ther* 2007; **3**: 327–41.

42 Needham I, Abderhalden C, Meer R, Dassen T, Haug H, Halfens R, *et al.* The effectiveness of two interventions in the management of patient violence in acute mental inpatient settings: Report on a pilot study. *J Psychiatr Ment Health Nurs* 2004; **11**: 595–601.

For peer review only - http://bmjopen.bmj.com/site/about/guidelines.xhtml

43 Park JS, Lee K. Modification of Severe Violent and Aggressive Behavior among Psychiatric Inpatients through the Use of a Short-Term Token Economy. *J Korean Acad Nurs* 2012; **42**: 1062–9.

44 van de Sande R, Nijman H, Noorthoorn E, Wierdsma A, Hellendoorn E, van der Staak C, *et al.* Aggression and seclusion on acute psychiatric wards: Effect of short-term risk assessment. *Br J Psychiatry* 2011; **199**: 473–8.

45 Needham I, Abderhalden C, Halfens R, Dassen T, Haug H, Fischer J. The effect of a training course in aggression management on mental health nurses' perceptions of aggression: A cluster randomised controlled trial. *Int J Nurs Stud* 2005; **42**: 649–55.

46 Sjostrom N, Eder D, Malm U, Beskow J. Violence and its prediction at a psychiatric hospital. *Eur Psychiatry* 2001; **16**: 459–65.

47 Sival RC, Albronda T, Haffmans PMJ, Saltet ML, Schellekens C. Is aggressive behaviour influenced by the use of a behaviour rating scale in patients in a psychogeriatric nursing home? *Int J Geriatr Psychiatry* 2000; **15**: 108–11.

48 Skovdahl K, Kihlgren AL, Kihlgren M. Dementia and aggressiveness: video recorded morning care from different care units. *J Clin Nurs* 2003; **12**: 888–98.

49 Stone T, McMillan M, Hazelton M, Clayton EH. Wounding Words: Swearing and Verbal Aggression in an Inpatient Setting. *Perspect Psychiatr Care* 2011; **47**: 194–203.

50 Sutton D, Wilson M, Van Kessel K, Vanderpyl J. Optimizing arousal to manage aggression: A pilot study of sensory modulation. *Int J Ment Health Nurs* 2013; **22**: 500–11.

51 Lipscomb JA, London M, Chen YM, Flannery K, Watt M, Geiger-Brown J, *et al.* Safety climate and workplace violence prevention in state-run residential addiction treatment centers. *Work J Prev Assess Rehabil* 2012; **42**: 47–56.

52 Wright S, Sayer J, Parr AM, Gray R, Southern D, Gournay K. Breakaway and physical restraint techniques in acute psychiatric nursing: Results from a national survey of training and practice. *J Forens Psychiatry Psychol* 2005; **16**: 380–98.

53 Berg J, Kaltiala-Heino R, Valimaki M. Management of aggressive behaviour among adolescents in forensic units: a four-country perspective. *J Psychiatr Ment Health Nurs* 2011; **18**: 776–85.

54 Biancosino B, Delmonte S, Grassi L, Santone G, Preti A, Miglio R, *et al.* Violent behavior in acute psychiatric inpatient facilities: A national survey in italy. *J Nerv Ment Dis* 2009; **197**: 772–82.

55 Caspi E. Aggressive behaviors between residents with dementia in an assisted living residence. *Dementia-International J Soc Res Pract* 2015; **14**: 528–46.

56 Chaplin R, McGeorge M, Hinchcliffe G, Shinkwin L. Aggression on psychiatric

For peer review only - http://bmjopen.bmj.com/site/about/guidelines.xhtml

inpatient units for older adults and adults of working age. *Int J Geriatr Psychiatry* 2008; **23**: 874–6.

57 Delaney J, Cleary M, Jordan R, Horsfall J. An exploratory investigation into the nursing management of aggression in acute psychiatric settings. *J Psychiatr Ment Heal Nurs* 2001; **8**: 77–84.

58 Meaden A, Hacker D, Spencer K. Acute aggression risk: An early warning signs methodology. *J Forensic Pract* 2013; **15**: 21–31.

59 Umut G, Altun ZO, Danismant BS, Kucukparlak I, Karamustafalioglu N. Relationship between treatment adherence, insight and violence among schizophrenia inpatients in a training hospital sample. *Dusunen Adam* 2012; **25**: 212–20.

60 Bahareethan M, Shah A. Aggressive behaviour, staff attitude and staff perception of patients on two continuing care psychogeriatric wards. *Aging Ment Health* 2000; **4**: 66–71.

61 Berg J, Kaltiala-Heino R, Loyttyniemi V, Valimaki M. Staff's perception of adolescent aggressive behaviour in four European forensic units: A qualitative interview study. *Nord J Psychiatry* 2013; **67**: 124–31.

62 Spokes K, Bond K, Lowe T, Jones J, Illingworth P, Brimblecombe N, *et al.* HOVIS -- The Hertfordshire/Oxfordshire Violent Incident Study. *J Psychiatr Ment Heal Nurs* 2002; **9**: 199–209.

63 Stevenson KN, Jack SM, O'Mara L, LeGris J. Registered nurses' experiences of patient violence on acute care psychiatric inpatient units: an interpretive descriptive study. *BMC Nurs* 2015; **14**: 35.

64 Zuzelo PR, Curran SS, Zeserman MA. Registered Nurses' and Behavior Health Associates' Responses to Violent Inpatient Interactions on Behavioral Health Units. *J Am Psychiatr Nurses Assoc* 2012; **18**: 112–26.

65 Camuccio C, Chambers M, Valimaki M, Farro D, Zanotti R. Managing distressed and disturbed patients: The thoughts and feelings experienced by Italian nurses. *J Psychiatr Ment Health Nurs* 2012; **19**: 807–15.

66 McCann T V, Baird J, Muir-Cochrane E. Factors Influencing Clinicians' Attitudes about Aggression in Australian Acute Old Age Psychiatry Inpatient Units: A Cross Sectional Survey Design. *Issues Ment Health Nurs* 2014; **35**: 542–50.

67 Tema T, Poggenpoel M, Myburgh C. Experiences of psychiatric nurses exposed to hostility from patients in a forensic ward. *J Nurs Manag* 2011; **19**: 915–24.

68 Nijman H, Bowers L, Oud N, Jansen G. Psychiatric nurses' experiences with inpatient aggression. *Aggress Behav* 2005; **31**: 217–27.

69 Chen WC, Wang JD, Lew-Ting CY, Chiu HJ, Lin YP. Workplace violence on workers caring for long-term institutionalized schizophrenic patients in Taiwan. *J Occup Health*

For peer review only - http://bmjopen.bmj.com/site/about/guidelines.xhtml

2007; **49**: 311–6.

70 Cutcliffe JR. Qualified nurses' lived experience of violence perpetrated by individuals suffering from enduring mental health problems: A hermeneutic study. *Int J Nurs Stud* 1999; **36**: 105–16.

71 Evans RE, Petter S. Identifying mitigating and challenging beliefs in dealing with threatening patients: An analysis of experiences of clinicians working in a psychiatric intensive care unit. *J Psychiatr Intensive Care* 2012; **8**: 113–9.

72 Lantta T, Daffern M, Kontio R, Valimaki M. Implementing the Dynamic Appraisal of Situational Aggression in Mental Health Units. *Clin Nurse Spec* 2015; **29**: 230–43.

73 Meehan T, McIntosh W, Bergen H. Aggressive behaviour in the high-secure forensic setting: the perceptions of patients. *J Psychiatr Ment Health Nurs* 2006; **13**: 19–25.

74 Olsson H, Audulv A, Strand S, Kristiansen L. Reducing or Increasing Violence in Forensic Care: A Qualitative Study of Inpatient Experiences. *Arch Psychiatr Nurs* 2015; **29**: 393–400.

75 van Wijk E, Traut A, Julie H. Environmental and nursing-staff factors contributing to aggressive and violent behaviour of patients in mental health facilities. *Curationis* 2014; **37**: 1122.

76 Daffern M. Assessing the functions of aggression in psychiatric inpatients. *Behav Anal Today* 2007; **8**: 43–51.

77 Duxbury J, Whittington R. Causes and management of patient aggression and violence: staff and patient perspectives. *J Adv Nurs* 2005; **50**: 469–78.

78 Janicki N. Prosecuting inpatient violence: Perceptions of staff, patients and others in a women's enhanced medium secure service. *Br J Forensic Pract* 2009; **11**: 27–38.

79 Lawn S, Pols R. Nicotine withdrawal: Pathway to aggression and assault in the locked psychiatric ward? *Australas Psychiatry* 2003; **11**: 199–203.

80 Nolan KA, Shope CB, Citrome L, Volavka J. Staff and Patient Views of the Reasons for Aggressive Incidents: A Prospective, Incident-Based Study. *Psychiatr Q* 2009; **80**: 167–72.

81 Dickens G, Piccirillo M, Alderman N. Causes and management of aggression and violence in a forensic mental health service: Perspectives of nurses and patients. *Int J Ment Health Nurs* 2013; **22**: 532–44.

82 Wright K, Duxbury J, Baker A, Crumpton A. A qualitative study into the attitudes of patients and staff towards violence and aggression in a high security hospital. *J Psychiatr Ment Health Nurs* 2014; **21**: 184–8.

83 Reininghaus U, Craig T, Gournay K, Hopkinson P, Carson J. The High Secure Psychiatric Hospitals' Nursing Staff Stress Survey 3: Identifying stress resistance resources in the stress process of physical assault. *Pers Individ Dif* 2007; **42**: 397–

For peer review only - http://bmjopen.bmj.com/site/about/guidelines.xhtml

408.

84 Trenoweth S. Perceiving risk in dangerous situations: risks of violence among mental health inpatients. *J Adv Nurs* 2003; **42**: 278–87.

85 De Niet G, Hutschemaekers G, Lendemeijer B. Is the reducing effect of the Staff Observation Aggression Scale owing to a learning effect? An explorative study. *J Psychiatr Ment Health Nurs* 2005; **12**: 687–94.

86 Bharwani G, Parikh PJ, Lawhorne LW, VanVlymen E, Bharwani M. Individualized Behavior Management Program for Alzheimer's/Dementia Residents Using Behavior-Based Ergonomic Therapies. *Am J Alzheimers Dis Other Dementias* 2012; **27**: 188–95.

87 Carlson GA, Potegal M, Margulies D, Basile J, Gutkovich Z. Liquid risperidone in the treatment of rages in psychiatrically hospitalized children with possible bipolar disorder. *Bipolar Disord* 2010; **12**: 205–12.

88 Chan S, Fung MY, Tong CW, Thompson D. The clinical effectiveness of a multisensory therapy on clients with developmental disability. *Res Dev Disabil* 2005; **26**: 131–42.

89 DeYoung S, Just G, Harrison R. Decreasing aggressive, agitated, or disruptive behavior: perticipation in a behavior management unit. *J Gerontol Nurs* 2002; **28**: 22–31.

90 Higueras A, Carretero-Dios H, Munoz JP, Idini E, Ortiz A, Rincon F, *et al.* Effects of a humor-centered activity on disruptive behavior in patients in a general hospital psychiatric ward. *Int J Clin Heal Psychol* 2006; **6**: 53–64.

91 Speziale J, Black E, Coatsworth-Puspoky R, Ross T, O'Regan T. Moving forward: evaluating a curriculum for managing responsive behaviors in a geriatric psychiatry inpatient population. *Gerontologist* 2009; **49**: 570–6.

92 Zwijsen SA, Smalbrugge M, Eefsting JA, Twisk JWR, Gerritsen DL, Pot AM, *et al.* Coming to Grips With Challenging Behavior: A Cluster Randomized Controlled Trial on the Effects of a Multidisciplinary Care Program for Challenging Behavior in Dementia. *J Am Med Dir Assoc* 2014; **15**. doi:10.1016/j.jamda.2014.04.007.

93 Bennett R, Ramakrishna V, Maganty D. Management of disturbed behaviour in a psychiatric intensive care unit: Views of staff on options for intervention. *J Psychiatr Intensive Care* 2011; **7**: 85–9.

94 Foley KL, Sudha S, Sloane PD, Gold DT. Staff perceptions of successful management of severe behavioral problems in dementia special care units. *Dement Int J Soc Res Pract* 2003; **2**: 105–24.

95 Hallett N, Dickens GL. De-escalation: A survey of clinical staff in a secure mental health inpatient service. *Int J Ment Health Nurs* 2015; **24**: 324–33.

For peer review only

96 Bowers L. Association Between Staff Factors and Levels of Conflict and Containment on Acute Psychiatric Wards in England. *Psychiatr Serv* 2009; **60**: 231–9.

97 Lowe T, Wellman N, Taylor R. Limit-setting and decision-making in the management of aggression. *J Adv Nurs* 2003; **41**: 154–61.

98 Quirk A, Lelliott P, Seale C. Risk management by patients on psychiatric wards in London: An ethnographic study. *Heal Risk Soc* 2005; **7**: 85–91.

99 Ireland CA, Halpin L, Sullivan C. Critical incidents in a forensic psychiatric population: an exploratory study of motivational factors. *J Forens Psychiatry Psychol* 2014; **25**: 714–32.

100 Jeffs L, Rose D, Macrae C, Maione M, Macmillan KM. What near misses tell us about risk and safety in mental health care. *J Psychiatr Ment Health Nurs* 2012; **19**: 430–7.

101 Koukia E, Mangoulia P, Stathopoulos T, Madianos M. Greek Mental Health Nurses' Practices and Attitudes in the Management of Acute Cases. *Issues Ment Health Nurs* 2013; **34**: 192–7.

102 Cole M, Baldwin D, Thomas P. Sexual assault on wards: Staff actions and reactions. *Int J Psychiatry Clin Pract* 2003; **7**: 239–42.

103 Phillips L. Reflections on the education and training of mental health staff who work with women who have been sexually abused in childhood. *J Psychiatr Ment Health Nurs* 2011; **18**: 696–705.

104 Yang MH, Wu SC, Lin JG, Lin LC. The efficacy of acupressure for decreasing agitated behaviour in dementia: a pilot study. *J Clin Nurs* 2007; **16**: 308–15.

105 Gallop R, Engels S, DiNunzio R, Napravnik S. Abused women's concerns about safety and the therapeutic environment during psychiatric hospitalization. *Can J Nurs Res* 1999; **31**: 53–70.

106 Cormac I, Russell I, Ferriter M. Review of seclusion policies in high secure hospitals and medium secure units in England, Scotland and Wales. *J Psychiatr Ment Health Nurs* 2005; **12**: 380–2.

107 Georgieva I, Mulder CL, Noorthoorn E. Reducing seclusion through involuntary medication: A randomized clinical trial. *Psychiatry Res* 2013; **205**: 48–53.

108 Kirkevold O, Engedal K. Prevalence of patients subjected to constraint in Norwegian nursing homes. *Scand J Caring Sci* 2004; **18**: 281–6.

109 Raboch J, Kalisova L, Nawka A, Kitzlerova E, Onchev G, Karastergiou A, *et al.* Use of Coercive Measures During Involuntary Hospitalization: Findings From Ten European Countries. *Psychiatr Serv* 2010; **61**: 1012–7.

110 Steinert T, Martin V, Baur M, Bohnet U, Goebel R, Hermelink G, *et al.* Diagnosis-related frequency of compulsory measures in 10 German psychiatric hospitals and correlates with hospital characteristics. *Soc Psychiatry Psychiatr Epidemiol* 2007; **42**:

For peer review only - http://bmjopen.bmj.com/site/about/guidelines.xhtml

140–5.

111 Bigwood S, Crowe M. ‘It’s part of the job, but it spoils the job’: a phenomenological study of physical restraint. *Int J Ment Health Nurs* 2008; **17**: 215–22.

112 Lee S, Gray R, Gournay K, Wright S, Parr A, Sayer J. Views of nursing staff on the use of physical restraint. *J Psychiatr Ment Health Nurs* 2003; **10**: 425–30.

113 Perkins E, Prosser H, Riley D, Whittington R. Physical restraint in a therapeutic setting; a necessary evil? *Int J Law Psychiatry* 2012; **35**: 43–9.

114 Sequeira H, Halstead S. The psychological effects on nursing staff of administering physical restraint in a secure psychiatric hospital: ’When I go home, it’s then that I think about it. *Br J Forensic Pract* 2004; **6**: 3–15.

115 Exworthy T, Mohan D, Hindley N, Basson J. Seclusion: punitive or protective? *J Forensic Psychiatry* 2001; **12**: 423–33.

116 Kuosmanen L, Makkonen P, Lehtila H, Salminen H. Seclusion experienced by mental health professionals. *J Psychiatr Ment Health Nurs* 2015; **22**: 333–6.

117 Muir-Cochrane E, Baird J, McCann T. Nurses’ experiences of restraint and seclusion use in short-stay acute old age psychiatry inpatient units: A qualitative study. *J Psychiatr Ment Health Nurs* 2015; **22**: 109–15.

118 Chien WT, Chan CW, Lam LW, Kam CW. Psychiatric inpatients’ perceptions of positive and negative aspects of physical restraint. *Patient Educ Couns* 2005; **59**: 80–6.

119 Knowles SF, Hearne J, Smith I. Physical restraint and the therapeutic relationship. *J Forens Psychiatry Psychol* 2015; **26**: 461–75.

120 Ezeobele I, Malecha A, Mock A, Mackey-Godine A, Hughes M. Patients’ lived seclusion experience in acute psychiatric hospital in the United States: A qualitative study. *J Psychiatr Ment Health Nurs* 2014; **21**: 303–12.

121 Faschingbauer KM, Peden-McAlpine C, Tempel W. Use of Seclusion: Finding the Voice of the Patient to Influence Practice. *J Psychosoc Nurs Ment Health Serv* 2013; **51**: 32–8.

122 Holmes D, Kennedy SL, Perron A. The mentally ill and social exclusion: a critical examination of the use of seclusion from the patient’s perspective. *Issues Ment Health Nurs* 2004; **25**: 559–78.

123 Bergk J, Einsiedler B, Flammer E, Steinert T. A Randomized Controlled Comparison of Seclusion and Mechanical Restraint in Inpatient Settings. *Psychiatr Serv* 2011; **62**: 1310–7.

124 Kontio R, Joffe G, Putkonen H, Kuosmanen L, Hane K, Holi M, *et al.* Seclusion and Restraint in Psychiatry: Patients’ Experiences and Practical Suggestions on How to Improve Practices and Use Alternatives. *Perspect Psychiatr Care* 2012; **48**: 16–24.

For peer review only - http://bmjopen.bmj.com/site/about/guidelines.xhtml

125 Larue C, Dumais A, Boyer R, Goulet MH, Bonin JP, Baba N. The Experience of Seclusion and Restraint in Psychiatric Settings: Perspectives of Patients. *Issues Ment Health Nurs* 2013; **34**: 317–24.

126 Bonner G, Lowe T, Rawcliffe D, Wellman N. Trauma for all: a pilot study of the subjective experience of physical restraint for mental health inpatients and staff in the UK. *J Psychiatr Ment Heal Nurs* 2002; **9**: 465–73.

127 Holmes D, Murray SJ, Knack N. Experiencing Seclusion in a Forensic Psychiatric Setting: A Phenomenological Study. *J Forensic Nurs* 2015; **11**: 200–13.

128 Bonner G, Wellman N. POSTINCIDENT REVIEW of Aggression and Violence in Mental Health Settings. *J Psychosoc Nurs Ment Health Serv* 2010; **48**: 35–40.

129 Bowers L, Van Der Merwe M, Paterson B, Stewart D. Manual restraint and shows of force: The City-128 study. *Int J Ment Health Nurs* 2012; **21**: 30–40.

130 Tompsett CJ, Domoff S, Boxer P. Prediction of Restraints Among Youth in a Psychiatric Hospital: Application of Translational Action Research. *J Clin Psychol* 2011; **67**: 368–82.

131 Boumans C, Egger J, Souren P, Mann-Poll P, Hutschemaekers G. Nurses' decision on seclusion: Patient characteristics, contextual factors and reflexivity in teams. *J Psychiatr Ment Health Nurs* 2012; **19**: 264–70.

132 Mason T, Whitehead E. Some specific problems of secluding female patients. *Med Sci Law* 2001; **41**: 315–24.

133 Ryan R, Happell B. Learning from experience: Using action research to discover consumer needs in post-seclusion debriefing. *Int J Ment Health Nurs* 2009; **18**: 100–7.

134 Whitecross F, Seeary A, Lee S. Measuring the impacts of seclusion on psychiatry inpatients and the effectiveness of a pilot single-session post-seclusion counselling intervention. *Int J Ment Health Nurs* 2013; **22**: 512–21.

135 Bleijlevens MHC, Gulpers MJM, Capezuti E, van Rossum E, Hamers JPH. Process Evaluation of a Multicomponent Intervention Program (EXBELT) to Reduce Belt Restraints in Nursing Homes. *J Am Med Dir Assoc* 2013; **14**: 599–604.

136 Pellfolk TJ, Gustafson Y, Bucht G, Karlsson S. Effects of a restraint minimization program on staff knowledge, attitudes, and practice: a cluster randomized trial. *J Am Geriatr Soc* 2010; **58**: 62–9.

137 Schreiner GM, Crafton CG, Sevin JA. Decreasing the use of mechanical restraints and locked seclusion. *Adm Policy Ment Health* 2004; **31**: 449–63.

138 Ching H, Daffern M, Martin T, Thomas S. Reducing the use of seclusion in a forensic psychiatric hospital: assessing the impact on aggression, therapeutic climate and staff confidence. *J Forens Psychiatry Psychol* 2010; **21**: 737–60.

139 Long CG, West R, Afford M, Collins L, Dolley O. Reducing the use of seclusion in a

secure service for women. *J Psychiatr Intensive Care* 2015; **11**: 84–94.

140 Smith S, Jones J. Use of a Sensory Room on an Intensive Care Unit. *J Psychosoc Nurs Ment Health Serv* 2014; **52**: 22–30.

141 Espinosa L, Harris B, Frank J, Armstrong-Muth J, Brous E, Moran J, *et al.* Milieu Improvement in Psychiatry Using Evidence-Based Practices: The Long and Winding Road of Culture Change. *Arch Psychiatr Nurs* 2015; **29**: 202–7.

142 Kontio R, Lahti M, Pitkanen A, Joffe G, Putkonen H, Hatonen H, *et al.* Impact of eLearning course on nurses' professional competence in seclusion and restraint practices: a randomized controlled study (ISRCTN32869544). *J Psychiatr Ment Health Nurs* 2011; **18**: 813–21.

143 Huizing AR, Hamers JP, Gulpers MJ, Berger MP. Short-term effects of an educational intervention on physical restraint use: a cluster randomized trial. *BMC Geriatr* 2006; **6**: 17.

144 Bak J, Zoffmann V, Sestoft DM, Almvik R, Brandt-Christensen M. Mechanical Restraint in Psychiatry: Preventive Factors in Theory and Practice. A Danish-Norwegian Association Study. *Perspect Psychiatr Care* 2014; **50**: 155–66.

145 Bak J, Zoffmann V, Sestoft DM, Almvik R, Siersma VD, Brandt-Christensen M. Comparing the effect of non-medical mechanical restraint preventive factors between psychiatric units in Denmark and Norway. *Nord J Psychiatry* 2015; **69**: 1715–25.

146 Keski-Valkama A, Sailas E, Eronen M, Koivisto AM, Lonnqvist J, Kaltiala-Heino R. A 15-year national follow-up: legislation is not enough to reduce the use of seclusion and restraint. *Soc Psychiatry Psychiatr Epidemiol* 2007; **42**: 747–52.

147 Lee S, Wright S, Sayer J, Parr AM, Gray R, Gournay K. Physical restraint training for nurses in English and Welsh psychiatric intensive care and regional secure units. *J Ment Heal* 2001; **10**: 151–62.

148 Kontio R, Valimaki M, Putkonen H, Cocoman A, Turpeinen S, Kuosmanen L, *et al.* Nurses' and Physicians' Educational Needs in Seclusion and Restraint Practices. *Perspect Psychiatr Care* 2009; **45**: 198–207.

149 Hatta K, Shibata N, Ota T, Usui C, Ito M, Nakamura H, *et al.* Association between physical restraint and drug-induced liver injury. *Neuropsychobiology* 2007; **56**: 180–4.

150 Bowers L, Alexander J, Simpson A, Ryan C, Carr-Walker P. Student psychiatric nurses' approval of containment measures: Relationship to perception of aggression and attitudes to personality disorder. *Int J Nurs Stud* 2007; **44**: 349–56.

151 Braham LG, Heasley JF, Akiens S. An evaluation of night confinement in a high secure hospital. *Ment Heal Rev J* 2013; **18**: 21–31.

152 Chu S, McNeill K, Wright KM, Hague A, Wilkins T. The impact of a night confinement policy on patients in a UK high secure inpatient mental health service. *J Forensic*

For peer review only - http://bmjopen.bmj.com/site/about/guidelines.xhtml

Pract 2015; **17**: 21–30.

153 Hottinen A, Valimaki M, Sailas E, Putkonen H, Joffe G, Noda T, *et al.* Attitudes towards different containment measures: a questionnaire survey in Finnish adolescent psychiatry. *J Psychiatr Ment Health Nurs* 2012; **19**: 521–7.

154 Haugom EW, Granerud A. Shielding in Mental Health Hospitals: Description and Assessment by Staff. *Sage Open* 2016; **6**: 1-10. doi:10.1177/2158244016629526.

155 Papadopoulos C, Bowers L, Quirk A, Khanom H. Events Preceding Changes in Conflict and Containment Rates on Acute Psychiatric Wards. *Psychiatr Serv* 2012; **63**: 40–7.

156 Looi GME, Engstrom A, Savenstedt S. A Self-Destructive Care: Self-Reports of People Who Experienced Coercive Measures and their Suggestions for Alternatives. *Issues Ment Health Nurs* 2015; **36**: 96–103.

157 Kontio R, Valimaki M, Putkonen H, Kuosmanen L, Scott A, Joffe G. Patient restrictions: Are there ethical alternatives to seclusion and restraint? *Nurs Ethics* 2010; **17**: 65–76.

158 Bak J, Aggernaes H. Coercion within Danish psychiatry compared with 10 other European countries. *Nord J Psychiatry* 2012; **66**: 297–302.

159 Jaeger M, Ketteler D, Rabenschlag F, Theodoridou A. Informal coercion in acute inpatient setting-Knowledge and attitudes held by mental health professionals. *Psychiatry Res* 2014; **220**: 1007–11.

160 Kim S, Rhee M, Seo MK. Coercion related variables and effects of coercion on therapeutic relationship. *Int J Neuropsychopharmacol* 2010; **13**: 224.

161 Lovell A, Smith D, Johnson P. A qualitative investigation into nurses' perceptions of factors influencing staff injuries sustained during physical interventions employed in response to service user violence within one secure learning disability service. *J Clin Nurs* 2015; **24**: 1926–35.

162 Tateno M, Sugiura K, Uehara K, Fujisawa D, Zhao Y, Hashimoto N, *et al.* Attitude of young psychiatrists toward coercive measures in psychiatry: a case vignette study in Japan. *Int J Ment Health Syst* 2009; **3**: 20.

163 Georgieva I, Mulder C, Wierdsma A. Patients' preference and experiences of forced medication and seclusion. *Psychiatr Q* 2012; **83**: 1–13.

164 Haw C, Stubbs J, Bickle A, Stewart I. Coercive treatments in forensic psychiatry: a study of patients' experiences and preferences. *J Forens Psychiatry Psychol* 2011; **22**: 564–85.

165 Sequeira H, Halstead S. 'Is it meant to hurt, is it?': Management of violence in women with developmental disabilities. *Violence Against Women* 2001; **7**: 462–76.

166 Larsen IB, Terkelsen TB. Coercion in a locked psychiatric ward: Perspectives of

For peer review only - http://bmjopen.bmj.com/site/about/guidelines.xhtml

patients and staff. *Nurs Ethics* 2014; **21**: 426–36.

167 Whittington R, Bowers L, Nolan P, Simpson A, Neil L. Approval Ratings of Inpatient Coercive Interventions in a National Sample of Mental Health Service Users and Staff in England. *Psychiatr Serv* 2009; **60**: 792–8.

168 Ryan CJ, Bowers L. Coercive manoeuvres in a psychiatric intensive care unit. *J Psychiatr Ment Heal Nurs* 2005; **12**: 695–702.

169 Mackay I, Paterson B, Cassells C. Constant or special observations of inpatients presenting a risk of aggression or violence: nurses' perceptions of the rules of engagement. *J Psychiatr Ment Heal Nurs* 2005; **12**: 464–71.

170 Delaney KR, Johnson ME. Keeping the Unit Safe: Mapping Psychiatric Nursing Skills. *J Am Psychiatr Nurses Assoc* 2006; **12**: 198–207.

171 Happell B, Koehn S. Seclusion as a necessary intervention: the relationship between burnout, job satisfaction and therapeutic optimism and justification for the use of seclusion. *J Adv Nurs* 2011; **67**: 1222–31.

172 Johnson ME, Delaney KR. Keeping the Unit Safe: A Grounded Theory Study. *J Am Psychiatr Nurses Assoc* 2006; **12**: 13–21.

173 Jonker E, Goossens P, Steenhuis I, Oud N. Patient aggression in clinical psychiatry: Perceptions of mental health nurses. *J Psychiatr Ment Health Nurs* 2008; **15**: 492–9.

174 Langan C, McDonald C. Daytime night attire as a therapeutic intervention in an acute adult psychiatric in-patient unit. *Psychiatr Bull* 2008; **32**: 221–4.

175 Maguire T, Daffern M, Martin T. Exploring nurses' and patients' perspectives of limit setting in a forensic mental health setting. *Int J Ment Health Nurs* 2014; **23**: 153–60.

176 Salzmann-Erikson M, Lutzen K, Ivarsson AB, Eriksson H. The core characteristics and nursing care activities in psychiatric intensive care units in Sweden. *Int J Ment Health Nurs* 2008; **17**: 98–107.

177 Millar R, Sands N. 'He did what? Well that wasn't handed over!' Communicating risk in mental health. *J Psychiatr Ment Health Nurs* 2013; **20**: 345–54.

178 Sjostrand M, Sandman L, Karlsson P, Helgesson G, Eriksson S, Juth N. Ethical deliberations about involuntary treatment: interviews with Swedish psychiatrists. *BMC Med Ethics* 2015; **16**: 37-49.

179 Woods P. Risk assessment and management approaches on mental health units. *J Psychiatr Ment Health Nurs* 2013; **20**: 807–13.

180 Cullen SW, Nath SB, Marcus SC. Toward Understanding Errors in Inpatient Psychiatry: A Qualitative Inquiry. *Psychiatr Q* 2010; **81**: 197–205.

181 Gifford ML, Anderson JE. Barriers and motivating factors in reporting incidents of assault in mental health care. *J Am Psychiatr Nurses Assoc* 2010; **16**: 288–98.

182 Martin T, Daffern M. Clinician perceptions of personal safety and confidence to

For peer review only - http://bmjopen.bmj.com/site/about/guidelines.xhtml

manage inpatient aggression in a forensic psychiatric setting. *J Psychiatr Ment Health Nurs* 2006; **13**: 90–9.

183 Mezey G, Hassell Y, Bartlett A. Safety of women in mixed-sex and single-sex medium secure units: staff and patient perceptions. *Br J Psychiatry* 2005; **187**: 579–82.

184 Brennan G, Flood C, Bowers L. Constraints and blocks to change and improvement on acute psychiatric wards--lessons from the City Nurses project. *J Psychiatr Ment Heal Nurs* 2006; **13**: 475–82.

185 Bowers L, Simpson A, Eyres S, Nijman H, Hall C, Grange A, *et al.* Serious untoward incidents and their aftermath in acute inpatient psychiatry: The Tompkins Acute Ward Study. *Int J Ment Health Nurs* 2006; **15**: 226–34.

186 Gabrielsson S, Looi GME, Zingmark K, Savenstedt S. Knowledge of the patient as decision-making power: staff members' perceptions of interprofessional collaboration in challenging situations in psychiatric inpatient care. *Scand J Caring Sci* 2014; **28**: 784–92.

187 Ward L. Ready, Aim Fire! Mental Health Nurses Under Siege in Acute Inpatient Facilities. *Issues Ment Health Nurs* 2013; **34**: 281–7.

188 Ryan P, Anczewska M, Laijarvi H, Czabala C, Hill R, & Kurek A. Demographic and situational variations in levels of burnout in European mental health services: a comparative study. *Diversity in Health & Social Care* 2007; **4**: 101-112.

189 Nathan R, Brown A, Redhead K, Holt G, Hill J. Staff responses to the therapeutic environment: A prospective study comparing burnout among nurses working on male and female wards in a medium secure unit. *J Forens Psychiatry Psychol* 2007; **18**: 342–52.

190 Vlayen A, Hellings J, Claes N, Peleman H, Schrooten W. A nationwide hospital survey on patient safety culture in Belgian hospitals: setting priorities at the launch of a 5-year patient safety plan. *BMJ Qual Saf* 2012; **21**: 760–7.

191 Wood D, Pistrang N. A safe place? Service users' experiences of an acute mental health ward. *J Community Appl Soc Psychol* 2004; **14**: 16–28.

192 Jones J, Nolan P, Bowers L, Simpson A, Whittington R, Hackney D, *et al.* Psychiatric wards: places of safety? *J Psychiatr Ment Health Nurs* 2010; **17**: 124–30.

193 Kanerva A, Kivinen T, Lammintakanen J. Communication elements supporting patient safety in psychiatric inpatient care. *J Psychiatr Ment Health Nurs* 2015; **22**: 298–305.

194 Kuosmanen A, Tiihonen J, Repo-Tiihonen E, Eronen M, Turunen H. Patient Safety Culture in Two Finnish State-Run Forensic Psychiatric Hospitals. *J Forensic Nurs* 2013; **9**: 207–16.

195 Wu JC, Tung TH, Chen PY, Chen YL, Lin YW, Chen FL. Determinants of workplace violence against clinical physicians in hospitals. *J Occup Health* 2015; **57**: 540–7.

For peer review only - http://bmjopen.bmj.com/site/about/guidelines.xhtml

196 Kanerva A, Lammintakanen J, Kivinen T. Nursing Staff's Perceptions of Patient Safety in Psychiatric Inpatient Care. *Perspect Psychiatr Care* 2016; **52**: 25–31.

197 Stead K, Kumar S, Schultz TJ, Tiver S, Pirone CJ, Adams RJ, *et al.* Teams communicating through STEPPS. *Med J Aust* 2009; **190**: 128–32.

198 Mahoney JS, Ellis TE, Garland G, Palyo N, Greene PK. Supporting a Psychiatric Hospital Culture of Safety. *J Am Psychiatr Nurses Assoc* 2012; **18**: 299–306.

199 Bowers L, Gournay K, Duffy D. Suicide and self-harm in inpatient psychiatric units: a national survey of observation policies. *J Adv Nurs* 2000; **32**: 437–44.

200 O'Brien L, Cole R. Mental health nursing practice in acute psychiatric close-observation areas. *Int J Ment Health Nurs* 2004; **13**: 89–99.

201 Stein W. The use of discharge risk assessment tools in general psychiatric services in the UK. *J Psychiatr Ment Health Nurs* 2002; **9**: 713–24.

202 Stubner S, Gros G, Nedopil N. Inpatient risk management with mentally ill offenders: Results of a survey on clinical decision-making about easing restrictions. *Crim Behav Ment Heal* 2006; **16**: 111–23.

203 O'Neill C, Heffernan P, Goggins R, Corcoran C, Linehan S, Duffy D, *et al.* Long-stay forensic psychiatric inpatients in the Republic of Ireland: aggregated needs assessment. *Ir J Psychol Med* 2003; **20**: 119–125.

204 Rees P, Manthorpe J. Managers' and Staff Experiences of Adult Protection Allegations in Mental Health and Learning Disability Residential Services: A Qualitative Study. *Br J Soc Work* 2010; **40**: 513–29.

205 Koukia E, Giannouli E, Gonis N, Douzenis A. Security rules and banned items in psychiatric acute admission wards in Athens, Greece. *Int J Ment Health Nurs* 2010; **19**: 428–36.

206 Silvana S, Laura F, Di Fabio U, Figa Talamanca I, Paolo B. Ergonomics in the psychiatric ward towards workers or patients? *Work* 2012; **41 Suppl 1**: 1832–5.

207 de Jonghe-Rouleau AP, Pot AM, de Jonghe JF. Self-injurious behaviour in nursing home residents with dementia. *Int J Geriatr Psychiatry* 2005; **20**: 651–7.

208 Sansone RA, McLean JS, Wiederman MW. The relationship between medically self-sabotaging behaviors and borderline personality disorder among psychiatric inpatients. *Prim Care Companion J Clin Psychiatry* 2008; **10**: 448–52.

209 Gough K, Hawkins A. Staff attitudes to self-harm and its management in a forensic psychiatric service. *Br J Forensic Pract* 2000; **2**: 22–8.

210 O'Donovan A. Pragmatism rules: the intervention and prevention strategies used by psychiatric nurses working with non-suicidal self-harming individuals. *J Psychiatr Ment Health Nurs* 2007; **14**: 64–71.

211 Tofthagen R, Talseth AG, Fagerstrom L. Mental health nurses' experiences of caring

For peer review only - http://bmjopen.bmj.com/site/about/guidelines.xhtml

for patients suffering from self-harm. *Nurs Res Pract* 2014; **2014**: 379-388.

212 Mattson AL, Binder P-E. A qualitative exploration of how health care workers in an inpatient setting in Norway experience working with patients who self-injure. *Nord Psychol* 2012; **64**: 272–90.

213 Brown J, Beail N. Self-Harm Among People with Intellectual Disabilities Living in Secure Service Provision: A Qualitative Exploration. *J Appl Res Intellect Disabil* 2009; **22**: 503–13.

214 Lindgren BM, Aminoff C, Graneheim UH. Features of Everyday Life in Psychiatric Inpatient Care for Self-harming: An Observational Study of Six Women. *Issues Ment Health Nurs* 2015; **36**: 82–8.

215 Weber MT. Triggers for self-abuse: A qualitative study. *Arch Psychiatr Nurs* 2002; **16**: 118–24.

216 Gibson J, Booth R, Davenport J, Keogh K, Owens T. Dialectical behaviour therapy-informed skills training for deliberate self-harm: A controlled trial with 3-month follow-up data. *Behav Res Ther* 2014; **60**: 8–14.

217 Booth R, Keogh K, Doyle J, Owens T. Living Through Distress: A Skills Training Group for Reducing Deliberate Self-Harm. *Behav Cogn Psychother* 2014; **42**: 156–65.

218 Bowers L, Whittington R, Nolan P, Parkin D, Curtis S, Bhui K, *et al.* Relationship between service ecology, special observation and self-harm during acute in-patient care: City-128 study. *Br J Psychiatry* 2008; **193**: 395–401.

219 Kool N, van Meijel B, Koekkoek B, van der Bijl J, Kerkhof A. Improving communication and practical skills in working with inpatients who self-harm: a pre-test/post-test study of the effects of a training programme. *BMC Psychiatry* 2014; **14**: 64-73.

220 Drew BL. No-suicide contracts to prevent suicidal behavior in inpatient psychiatric settings. *J Am Psychiatr Nurses Assoc* 1999; **5**: 23–8.

221 Sjostrom N, Hetta J, Waern M. Sense of coherence and suicidality in suicide attempters: a prospective study. *J Psychiatr Ment Heal Nurs* 2012; **19**: 62–9.

222 Swogger MT, Van Orden KA, Conner KR. The Relationship of Outwardly Directed Aggression to Suicidal Ideation and Suicide Attempts Across Two High-Risk Samples. *Psychol Violence* 2014; **4**: 184–95.

223 Ellis TE, Green KL, Allen JG, Jobes DA, Nadorff MR. Collaborative Assessment and Management of Suicidality in an Inpatient Setting: Results of a Pilot Study. *Psychotherapy* 2012; **49**: 72–80.

224 Ellis TE, Rufino KA, Allen JG, Fowler JC, Jobes DA. Impact of a Suicide-Specific Intervention within Inpatient Psychiatric Care: The Collaborative Assessment and Management of Suicidality. *Suicide Life-Threatening Behav* 2015; **45**: 556–66.

225 Cleary M, Jordan R, Horsfall J, Mazoudier P, Delaney J. Suicidal patients and special

For peer review only - http://bmjopen.bmj.com/site/about/guidelines.xhtml

observation. *J Psychiatr Ment Heal Nurs* 1999; **6**: 461–7.

226 Takahashi C, Chida F, Nakamura H, Akasaka H, Yagi J, Koeda A, *et al.* The impact of inpatient suicide on psychiatric nurses and their need for support. *BMC Psychiatry* 2011; **11**: 38-46.

227 Vrale G, Steen E. The dynamics between structure and flexibility in constant observation of psychiatric inpatients with suicidal ideation. *J Psychiatr Ment Health Nurs* 2005; **12**: 513–8.

228 Davis SE, Williams IS, Hays LW. Psychiatric inpatients' perceptions of written no-suicide agreements: An exploratory study. *Suicide Life-Threatening Behav* 2002; **32**: 51–66.

229 Esposito-Smythers C, McClung TJ, Fairlie AM. Adolescent perceptions of a suicide prevention group on an inpatient unit. *Arch Suicide Res* 2006; **10**: 265–75.

230 Sun F, Long A, Boore J, Tsao L. Nursing people who are suicidal on psychiatric wards in Taiwan: Action/interaction strategies. *J Psychiatr Ment Health Nurs* 2005; **12**: 275–82.

231 Sun FK, Long A, Boore J, Tsao LI. Patients and nurses' perceptions of ward environmental factors and support systems in the care of suicidal patients. *J Clin Nurs* 2006; **15**: 83–92.

232 Caspi E. Does self-neglect occur among older adults with dementia when unsupervised in assisted living? An exploratory, observational study. *J Elder Abuse Negl* 2014; **26**: 123–49.

233 Bowers L, Haglund K, Muir-Cochrane E, Nijman H, Simpson A, Van Der Merwe M. Locked doors: a survey of patients, staff and visitors. *J Psychiatr Ment Heal Nurs* 2010; **17**: 873–80.

234 Haglund K, Von Essen L. Locked entrance doors at psychiatric wards - Advantages and disadvantages according to voluntarily admitted patients. *Nord J Psychiatry* 2005; **59**: 511–5.

235 Muir-Cochrane E, van der Merwe M, Nijman H, Haglund K, Simpson A, Bowers L. Investigation into the acceptability of door locking to staff, patients, and visitors on acute psychiatric wards. *Int J Ment Health Nurs* 2012; **21**: 41–9.

236 Cowman S, Bowers L. Safety and security in acute admission psychiatric wards in Ireland and London: a comparative study. *J Clin Nurs* 2009; **18**: 1346–53.

237 Simpson A, Bowers L, Haglund K, Muir-Cochrane E, Nijman H, Van Der Merwe M. The relationship between substance use and exit security on psychiatric wards. *J Adv Nurs* 2011; **67**: 519–30.

238 van der Schaaf P, Dusseldorp E, Keuning F, Janssen W, Noorthoorn E. Impact of the physical environment of psychiatric wards on the use of seclusion. *Br J Psychiatry*

For peer review only - http://bmjopen.bmj.com/site/about/guidelines.xhtml

2013; **202**: 142–9.
239 Verbeek H, Zwakhalen SM, van Rossum E, Ambergen T, Kempen GI, Hamers JP. Effects of small-scale, home-like facilities in dementia care on residents' behavior, and use of physical restraints and psychotropic drugs: a quasi-experimental study. *Int Psychogeriatrics* 2014; **26**: 657–68.
240 Curtis S, Gesler W, Wood V, Spencer I, Mason J, Close H, *et al.* Compassionate containment? Balancing technical safety and therapy in the design of psychiatric wards. *Soc Sci Med* 2013; **97**: 201–9.
241 Bellantonio S, Kenny AM, Fortinsky RH, Kleppinger A, Robison J, Gruman C, *et al.* Efficacy of a geriatrics team intervention for residents in dementia-specific assisted living facilities: Effect on unanticipated transitions. *J Am Geriatr Soc* 2008; **56**: 523–8.
242 Chandler G. From traditional inpatient to trauma-informed treatment: transferring control from staff to patient. *J Am Psychiatr Nurses Assoc* 2008; **14**: 363–71.
243 Wilkes L, Fleming A, Wilkes BL, Cioffi JM, Le Miere J. Environmental approach to reducing agitation in older persons with dementia in a nursing home. *Australas J Ageing* 2005; **24**: 141–5.
244 Gebhardt R, Steinert T. Should severely disturbed psychiatric patients be distributed or concentrated in specialized wards? An empirical study on the effects of hospital organization on ward atmosphere, aggressive behavior, and sexual molestation. *Eur Psychiatry* 1999; **14**: 291–7.
245 Stolker JJ, Nijman HL, Zwanikken PH. Are patients' views on seclusion associated with lack of privacy in the ward? *Arch Psychiatr Nurs* 2006; **20**: 282–7.
246 Kulkarni J, Gavrilidis E, Lee S, Van Rheenen TE, Grigg J, Hayes E, *et al.* Establishing female-only areas in psychiatry wards to improve safety and quality of care for women. *Australas Psychiatry* 2014; **22**: 551–6.
247 Bowers L, Crowder M. Nursing staff numbers and their relationship to conflict and containment rates on psychiatric wards-A cross sectional time series Poisson regression study. *Int J Nurs Stud* 2012; **49**: 15–20.
248 Hunt IM, Windfuhr K, Shaw J, Appleby L, Kapur N. Ligature points and ligature types used by psychiatric inpatients who die by hanging anational study. *Crisis* 2012; **33**: 87–94.
249 Ruzic K, Franciskovic T, Sukovic Z, Hero ED, Roncevic-Grzeta I, Graovac M, *et al.* Aggresiveness in institutionalised schizophrenic patients and the selection of antipsychotics. *Coll Antropol* 2011; **35**: 265–9.
250 Seemuller F, Riedel M, Obermeier M, Bauer M, Adli M, Mundt C, *et al.* The controversial link between antidepressants and suicidality risks in adults: Data from a naturalistic study on a large sample of in-patients with a major depressive episode. *Int*

For peer review only - http://bmjopen.bmj.com/site/about/guidelines.xhtml

J Neuropsychopharmacol 2009; **12**: 181–9.

251 Haw C, Stubbs J, Dickens G. Barriers to the reporting of medication administration errors and near misses: An interview study of nurses at a psychiatric hospital. *J Psychiatr Ment Health Nurs* 2014; **21**: 797–805.

252 Soerensen AL, Lisby M, Nielsen LP, Poulsen BK, Mainz J. The medication process in a psychiatric hospital: are errors a potential threat to patient safety? *Risk Manag Healthc Policy* 2013; **6**: 23–31.

253 Bademli K, Buldukoglu K. Oral medication management in inpatient psychiatric care in Turkey. *J Psychiatr Ment Health Nurs* 2009; **16**: 355–62.

254 Prins M, Drenth-van Maanen A, Kok R, Jansen P. Use of a Structured Medication History to Establish Medication Use at Admission to an Old Age Psychiatric Clinic: A Prospective Observational Study. *CNS Drugs* 2013; **27**: 963–9.

255 Cottney A, Innes J. Medication-administration errors in an urban mental health hospital: A direct observation study. *Int J Ment Health Nurs* 2015; **24**: 65–74.

256 Dickens G, Stubbs J, Haw C. Delegation of medication administration: an exploratory study. *Nurs Stand* 2008; **22**: 35–40.

257 Haw C, Stubbs J, Dickens G. An observational study of medication administration errors in old-age psychiatric inpatients. *Int J Qual Heal Care* 2007; **19**: 210–6.

258 Cottney A. Improving the safety and efficiency of nurse medication rounds through the introduction of an automated dispensing cabinet. *BMJ Qual Improv Reports* 2014; **3:** u204237.w1843.

259 Dolan M, Kirwan H. Survey of staff perceptions of illicit drug use among patients in a medium secure unit. *Psychiatr Bull* 2001; **25**: 14–7.

260 Gonzalez-Pinto A, Ruiz De Azua S. Adherence to treatment in bipolar disorders. *Eur Neuropsychopharmacol* 2011; **21**: S108–9.

261 Meehan T, Morrison P, McDougall S. Absconding behaviour: an exploratory investigation in an acute inpatient unit. *Aust N Z J Psychiatry* 1999; **33**: 533–7.

262 Muir-Cochrane E, Oster C, Grotto J, Gerace A, Jones J. The inpatient psychiatric unit as both a safe and unsafe place: Implications for absconding. *Int J Ment Health Nurs* 2013; **22**: 304–12.

263 Nurjannah I, FitzGerald M, Foster K. Patients' experiences of absconding from a psychiatric setting in Indonesia. *Int J Ment Health Nurs* 2009; **18**: 326–35.

264 Grotto J, Gerace A, O'Kane D, Simpson A, Oster C, Muir-Cochrane E. Risk assessment and absconding: perceptions, understandings and responses of mental health nurses. *J Clin Nurs* 2015; **24**: 855–65.

265 Bowers L, Alexander J, Gaskell C. A trial of an anti-absconding intervention in acute psychiatric wards. *J Psychiatr Ment Heal Nurs* 2003; **10**: 410–6.

For peer review only - http://bmjopen.bmj.com/site/about/guidelines.xhtml

266 Simpson AI, Penney SR, Fernane S, Wilkie T. The impact of structured decision making on absconding by forensic psychiatric patients: results from an A-B design study. *BMC Psychiatry* 2015; **15**: 103-116.

267 Algase DL, Beattie ER, Antonakos C, Beel-Bates CA, Yao L. Wandering and the physical environment. *Am J Alzheimer's Dis Other Dementias* 2010; **25**: 340–6.

268 Colombo M, Vitali S, Cairati M, Perelli-Cippo R, Bessi O, Gioia P, *et al.* Wanderers: Features, findings, issues. *Arch Gerontol Geriatr* 2001; **7**: 99–106.

269 Hunt IM, Windfuhr K, Swinson N, Shaw J, Appleby L, Kapur N. Suicide amongst psychiatric in-patients who abscond from the ward: A national clinical survey. *BMC Psychiatry* 2010; **10**: 14-20.

270 Nijman H, Bowers L, Haglund K, Muir-Cochrane E, Simpson A, Van der Merwe M. Door locking and exit security measures on acute psychiatric admission wards. *J Psychiatr Ment Health Nurs* 2011; **18**: 614–21.

271 Whaley AL. Cultural mistrust and the clinical diagnosis of paranoid schizophrenia in African American patients. *J Psychopathol Behav Assess* 2001; **23**: 93–100.

272 Whaley AL. A Two-Stage Method for the Study of Cultural Bias in the Diagnosis of Schizophrenia in African Americans. *J Black Psychol* 2004; **30**: 167–86.

273 Koukia E, Madianos MG, Katostaras T. 'On the spot' interventions by mental health nurses in inpatient psychiatric wards in Greece. *Issues Ment Health Nurs* 2009; **30**: 327–36.

274 Lindsey PL. Psychiatric nurses' decision to restrain. *J Psychosoc Nurs Ment Heal Serv* 2009; **47**: 41–9.

275 Mann-Poll PS, Smit A, de Vries WJ, Boumans CE, Hutschemaekers GJ. Factors contributing to mental health professionals' decision to use seclusion. *Psychiatr Serv* 2011; **62**: 498–503.

276 Marangos-Frost S, Wells D. Psychiatric nurses' thoughts and feelings about restraint use: a decision dilemma. *J Adv Nurs* 2000; **31**: 362–9.

277 Brown B, Rakow T. Understanding clinicians' use of cues when assessing the future risk of violence: A clinical judgement analysis in the psychiatric setting. *Clin Psychol Psychother* 2015; **23**: 125-141.

278 Fuller J, Cowan J. Risk assessment in a multi-disciplinary forensic setting: Clinical judgement revisited. *J Forensic Psychiatry* 1999; **10**: 276–89.

279 Abraham S. Managing Patient Falls in Psychiatric Inpatient Units Part 1. *Health Care Manag* 2016; **35**: 21–7.

280 Fonad E, Emami A, Wahlin TB, Winblad B, Sandmark H. Falls in somatic and dementia wards at Community Care Units. *Scand J Caring Sci* 2009; **23**: 2–10.

281 Tangman S, Eriksson S, Gustafson Y, Lundin-Olsson L. Precipitating factors for falls

For peer review only - http://bmjopen.bmj.com/site/about/guidelines.xhtml

among patients with dementia on a psychogeriatric ward. *Int Psychogeriatrics* 2010; **22**: 641–9.

282 Garfinkel D, Radomislsky Z, Jamal S, Ben-Israel J. High efficacy for hip protectors in the prevention of hip fractures among elderly people with dementia. *J Am Med Dir Assoc* 2008; **9**: 313–8.

283 Holmes D, Teresi JA, Ramirez M, Ellis J, Eimicke J, Jian K, *et al.* An evaluation of a monitoring system intervention: falls, injuries, and affect in nursing homes. *Clin Nurs Res* 2007; **16**: 317–35.

284 Powell-Cope G, Quigley P, Besterman-Dahan K, Smith M, Stewart J, Melillo C, *et al.* A Qualitative Understanding of Patient Falls in Inpatient Mental Health Units. *J Am Psychiatr Nurses Assoc* 2014; **20**: 328–39.

285 Sakinofsky I. Preventing Suicide Among Inpatients. *Can J Psychiatry-Revue Can Psychiatr* 2014; **59**: 131–40.

286 NHS Providers. Funding for mental health at a local level: Unpicking the variation. 2016. London.

287 Prince M, Patel V, Saxena S, Maj M, Maselko J, Phillips MR, *et al.* No health without mental health. *Lancet* 2007; **370**: 859–77.

288 Unsworth J, Mckeever M, Kelleher M. Recognition of physical deterioration in patients with mental health problems: The role of simulation in knowledge and skill development. *J Psychiatr Ment Health Nurs* 2012; **19**: 536–45.

289 Cooper JB, Gaba DM, Woods DD, Blum LN. Patient Safety. *Medscape* 2000; **2**: 1–12.

290 Elliott JH, Turner T, Clavisi O, Thomas J, Higgins JPT, Mavergames C, *et al.* Living Systematic Reviews: An Emerging Opportunity to Narrow the Evidence-Practice Gap. *PLoS Med* 2014; **11**: 1–6.

291 Long L. Routine piloting in systematic reviews-A modified approach? *Syst Rev* 2014; **3**: 1–5.

292 Landis JR, Koch GG. The Measurement of Observer Agreement for Categorical Data. *Biometrics* 1977; **33**: 159–74.

293 Hox JJ, Boeije HR. Data Collection, Primary vs. Secondary. Encycl. Soc. Meas. 2005; 593–9.

294 Appleby L, Director Nav Kapur Frcp, Shaw J, Hunt IM, Fellow Sandra Flynn R, Fellow Saied Ibrahim R, *et al.* The National Confidential Inquiry into Suicide and Homicide by People with Mental Illness. 2016; 35–84.

295 Gopalakrishnan S GP. Systematic Reviews and Meta-analysis: Understanding the Best Evidence in Primary Healthcare. *J Fam. Med Prim Care* 2013; **2**: 9–14.

296 Dewa, Lindsay H., Murray, Kevin., Ramtale, S.C., Thibaut, B.I., Adam, Sheila,. Darzi, A., Archer S. Identifying research priorities for patient safety in mental health: an

For peer review only - http://bmjopen.bmj.com/site/about/guidelines.xhtml

international expert Delphi study. *BMJ Open* 2018; **8**: e021361.

For peer review only

For peer review only - http://bmjopen.bmj.com/site/about/guidelines.xhtml

Table 1: Overview of study characteristic identified within each category

Category	Sub category	Category definition	Number of studies	Countries	Number of studies using staff participants	Number of studies using patient participants	Total number of participants	Settings (number of studies conducted in each setting)
Interpersonal Violence	Aggression Violence<br>Challenging behaviour<br>Violence and aggression<br>Critical incidents<br>Conflict<br>Sexual Assault<br>Agitation	Behaviours or events that are considered hostile with the intent to cause harm, including violence, aggression and conflicts. This also encompasses sudden emergency incidents that require management.	91	UK-28<br>USA-17<br>Australia-8<br>Canada-6<br>Netherlands-4<br>Sweden-4<br>Taiwan-3<br>South Africa-2<br>Switzerland-2<br>India-2<br>Italy-2<br>Turkey-2<br>Europe-2	39<br>18 mixed	32<br>18 mixed<br>1 family members of patients<br>1 N/A	14,472 (excl. missing data)	Psychiatric inpatient wards/facilities-55<br>Forensic inpatient facilities- 19<br>Long-term care/Nursing homes- 13<br>Specialised research unit - 1<br>Mixed- 3

For peer review only

	Abuse			New Zealand-1<br>South Korea-1<br>Finland-1<br>Greece-1<br>Spain-1<br>Hong Kong-1<br>Israel-1<br>Nigeria-1<br>Norway-1				
Coercive Interventions	Restraint<br><br>Seclusion<br><br>Attitudes to coercion<br><br>Seclusion and restraint	Techniques for managing patient behaviour that are applied without consent, for the safety of the patient and others. These include	64	UK-21<br>Finland-7<br>USA-5<br>Netherlands-5<br>Australia-4<br>Canada-3<br>Norway-3<br>Germany-2<br>Sweden-2	23<br>15 mixed	26<br>15 mixed	48,916 (excl. missing data)	Psychiatric inpatient wards/facilities-45<br>Forensic inpatient facilities- 13<br>Long-term care/Nursing homes- 3

For peer review only

	Containment<br><br>Process of coercion<br><br>Alternative interventions<br><br>Shielding<br><br>Conflict	seclusion, restraint and containment.		Japan-2<br>Denmark & Norway-2<br>New Zealand-1<br>Europe-1<br>Europe & Australia-1<br>Europe & Israel-1<br>China-1<br>Switzerland-1<br>South Korea-1				Mixed- 3
Safety Culture	Process<br><br>Culture<br>Policy	The organisational attitudes, beliefs and values concerning safety. This encompasses the policies and	37	UK-9<br>Australia-7<br>USA-4<br>Sweden-3<br>Finland-3<br>Canada-2<br>Ireland-2	26<br>9 mixed	2<br>9 mixed	57,788 (excl. missing data)	Psychiatric inpatient wards/facilities-27<br>Forensic inpatient facilities- 6

For peer review only

		procedures within the healthcare organisation in relation to safety.		Netherlands-1<br>Greece-1<br>Italy-1<br>Germany-1<br>Belgium-1<br>Taiwan-1<br>Europe-1				Long-term care/Nursing homes- 1<br>Mixed- 3
Harm to self	Self-harm<br><br>Suicidal behaviour<br><br>Self-neglect	The ways in which the healthcare system attempts to prevent, mitigate or manage deliberate behaviours displayed by patients that are intended to cause harm or death to themselves.	26	USA-9<br>UK-3<br>Ireland-3<br>Norway-3<br>Netherlands-2<br>Sweden-2<br>Taiwan-2<br>Australia-1<br>Japan-1	9<br>3 mixed	14<br>3 mixed	2822 (excl. missing data)	Psychiatric inpatient wards/facilities-21<br>Forensic inpatient facilities- 2<br>Long-term care/Nursing homes- 2<br>Mixed- 1

For peer review only

Safety of the physical environment	Security<br><br>Environmental design<br><br>Transitions of care<br><br>Patient distribution<br><br>Staffing<br><br>Ligatures	The factors related to the physical environment of the healthcare setting that could impact upon safety. This includes ligature points, staffing, security (door locking) and patient distribution.	16	UK-6<br>Netherlands-3<br>USA-2<br>Australia-2<br>Germany-1<br>UK & Ireland-1<br>Sweden-1	4<br>5 mixed	7<br>5 mixed	3140 (excl. missing data)	Psychiatric inpatient wards/facilities-12<br>Forensic inpatient facilities- 1<br>Long-term care/Nursing homes- 3
Medication Safety	Adverse events<br><br>Medication administration<br><br>Medication management	Mistakes made at any stage of the medication u se process, from preparation, to administration and recording. This includes adverse drug	12	UK-6<br>Turkey-1<br>Spain-1<br>Netherlands-1<br>Croatia-1<br>Germany-1<br>Denmark-1	6<br>1 mixed	5<br>1 mixed	1991 (excl. missing data)	Psychiatric inpatient wards/facilities-9<br>Forensic inpatient facilities- 2

For peer review only

	Medication dispensing<br>Adherence<br>Substance use	events (ADEs, or injuries that are the result of a drug-related intervention) and issues surrounding drug/alcohol use.						Long-term care/Nursing homes- 1
Unauthorised Leave	Absconding<br>Wandering	The act of a patient leaving the healthcare setting without the knowledge or consent of staff/carers. This can be either with (absconding) or without intent (wandering) on the part of the patient.	10	UK-3<br>Australia-3<br>USA-1<br>Canada-1<br>Italy-1<br>Indonesia-1	2<br>1 mixed	7<br>1 mixed	957 (excl. missing data)	Psychiatric inpatient wards/facilities-9<br>Long-term care/Nursing homes- 1

Clinical decision making	Incident management<br><br>Risk assessment<br><br>Diagnosis	Incorrect diagnoses, risk assessments and other decision making processes of healthcare staff that impact upon the safety of a patient.	8	USA-3<br>UK-2<br>Canada-1<br>Greece-1<br>Netherlands-1	5	3	517	Psychiatric inpatient wards/facilities-7<br>Forensic inpatient facilities- 1
Accidents	Falls<br><br>Injuries	Events that lead to the unintentional harm of an individual. This includes trips, falls and injuries such as fractures.	6	USA-3<br>Sweden-2<br>Israel-1	3	3	180 (excl. missing data)	Psychiatric inpatient wards/facilities-5<br>Long-term care/Nursing homes- 1

For peer review only

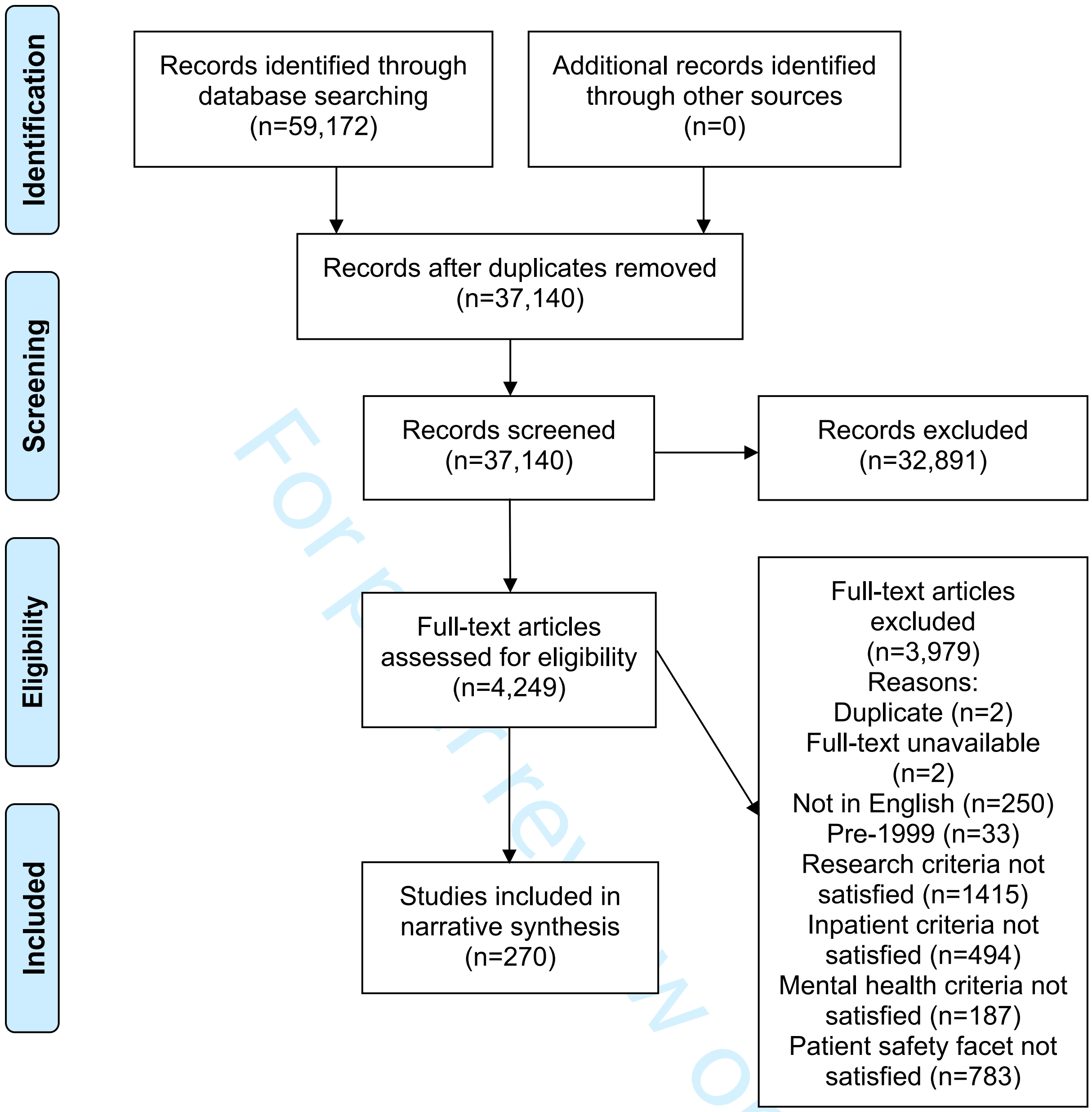

Figure 1: Flowchart of studies

For peer review only

Online supplement 1

Development of inclusion and exclusion criteria for screening

Facets

- Research
- Patient Safety
- Mental Health
- Inpatient Setting

Each one of these becomes an ***inclusion criteria*** by having an agreed definition across the research team.

Good agreement was found between the two researcher's judgements κ = 0.72 (95% CI, 0.69 to 0.74), $p < .0005$. Disagreements were discussed until consensus was reached. Subsequently, two independent reviewers screened the full text articles of the abstracts that indicated that the paper met, or could meet, the inclusion criteria. Agreement rates were calculated using the kappa statistic. Substantial agreement was reached (k=0.64 (95% CI, 0.55 to 0.73), $p < .0005$).

- Mental Health (defined as a field comprising various professions, such as psychiatry and social work, that deals with the promotion of mental and psychological well-being and the prevention, diagnosis, or treatment of mental disorders as listed in the Diagnostic and Statistical Manual 5th edition (American Psychiatric Association, 2013))
- Patient Safety (defined as "The avoidance, prevention and amelioration of adverse outcomes or injuries stemming from the process of healthcare" (Vincent, 2006))
- Research (defined as diligent and systematic inquiry or investigation into a subject in order to discover or revise facts, theories, applications etc.)
- Inpatient Setting (defined as hospital settings which provide continuous care for a period of over 24 hours)

Inclusion Criteria:

- Interventions/outcomes- Articles that report patient safety outcome measures.

Exclusion Criteria:

- Population- Articles that use the words 'forensic', 'secure', 'closed' or 'locked' can be taken to refer to an inpatient environment.

- Population- Articles that amalgamate data from both inpatient and outpatient settings such that data for an inpatient only sample is not available.

- Interventions/outcomes- Articles that solely examine the reliability or validity of risk assessment tools, with no relation to the management of the risk that the tool is measuring.

- Interventions/outcomes- Articles that do not measure patient safety outcomes (this aspect needs to be overtly expressed in the article, as an aim or research question).

For peer review only

- Interventions/outcomes- Clinical effectiveness versus patient safety. Clinical effectiveness concerns providing the best possible treatment for patients (e.g. with effective drug treatment) as opposed to being a patient safety issue. To be included, the treatment being studied has to be linked to a patient safety outcome/measurement (like a behaviour or adverse event).

- Interventions/outcomes- Articles reporting staff perceptions/feelings concerning safety related issues are only to be included when directly related to, or discussed in terms of, a patient safety outcome/measurement (like a behaviour or adverse event).

- Settings- Articles that are not based on purely mental health care settings (i.e. exclude general hospital wards and nursing homes that do not specify mental health specialised wards). Mental health needs to be separately examined and linked to patient safety.

- Settings- Articles based in primary care, community or social care settings.

- Settings- Articles that report on patient safety events/outcomes that occur outside of the inpatient setting.

Other general exclusion criteria:

- Articles that are reviews of any kind (including literature and systematic reviews). These will be excluded, but the reference lists will be reviewed and cross-referenced to ensure we have included all relevant articles that were used in data synthesis. Will see if grads can assist with this part of the process.

- Articles that are not empirical research; i.e. articles that do not have clearly defined hypotheses, research questions or aims that generate new primary data (data being collected for the purposes of the research). Secondary data is to be excluded, even if a new analysis has been done on it. Look for results and method sections here to ensure there were clear aims and that the data produced relates to those aims. For quantitative studies, there should be hypotheses and for qualitative studies, there should be clear aims or a research question.

- Opinion/editorials/commentaries (save any information we come across for developing our priorities in line with the expert consensus study)

- Articles not in English

- Conference abstracts

- Audits (this is not new data but routinely collected data).

- Case studies based on individual patients and their clinical presentation should be excluded. Inclusion of case study in the research facet is to pick up case studies based on health organisations or countries etc. (surrounding mental health and patient safety) not individual people.

- Reports and book abstracts

For peer review only

- Dissertations (not peer-reviewed).
- ‘Snapshot’ studies that provide clinical pictures of a patient group/setting (no new data).
- Epidemiological studies. Studies describing mental health conditions or the difference between similar conditions within an inpatient setting does not satisfy the patient safety facet (there is no management of patient safety).
- Ambiguities that prevent researchers from confirming any criteria (not enough resource available to contact researchers).

The process for reviewing full-text articles

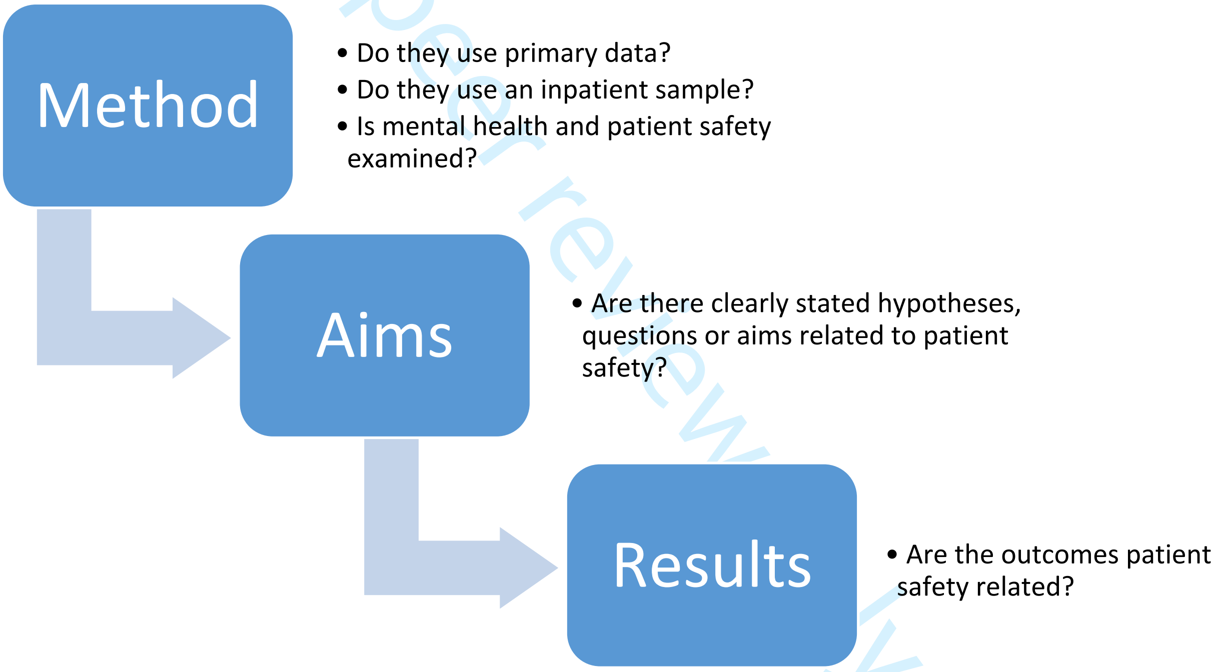

For peer review only

Online supplement 2

Full search strategy

Search hits by term

Search term	Number of hits
Mental health.ts.	365,884
Mental well-being.ts.	3,940
Mental well being.ts.	3,940
Psychological well being.ts.	22,247
Psychological well-being.ts.	22,247
Mental disorder*.ts	510,518
Mental illness*.ts.	84,610
Mental disease*.ts.	6,777
Psychiatr*.ts.	1,549,682
Anxiety disorder*.ts.	102,950
Delirium.ts.	42,017
Dementia.ts.	330,579
Dissociative disorder*.ts.	5,155
Factitious disorder*.ts.	2,456
Impulse control disorder*.ts.	5,576
Mood disorder*.ts.	62,076
Affective disorder*.ts.	59,066
Psychotic disorder*.ts.	59,618
Depressive disorder*.ts.	158,609
Neurotic disorder*.ts.	17,850
Personality disorder*.ts.	84,685
Conduct disorder*.ts.	18,858
Schizophreni*.ts.	422,487

Somatoform disorder*.ts.	13,969
Substance related disorder*.ts.	96,366
Clinical Psychology.ts.	7,526
Impulsive behavio?r.ts.	0
Adjustment disorder*.ts.	7,471
Eating disorder*.ts.	70,352
Sleep disorder*.ts.	60,587
Neuros?s.ts.	19,958
Psychos?s.ts.	132,109
Delusion*.ts.	33,018
Paranoia.ts.	7,196
Hallucination*.ts.	40,149
Addiction*.ts.	297,958
Dependence.ts.	1,629,560
Misuse.ts.	55,103
New psychoactive substance*.ts.	618
Legal high*.ts.	1,181
Depression.ts.	1,078,478
Panic disorder*.ts.	40,678
Phobia*.ts.	34,282
Health anxiet*.ts.	1,811
Bipolar disorder*.ts.	109,155
Alcohol abuse.ts.	42,632
Alcoholism.ts.	149,509
Obsessive compulsive disorder*.ts.	51,255
Obsessive thought*.ts.	394
Intrusive thought*.ts.	2,952
Post traumatic stress disorder*.ts.	24,746
Post-traumatic stress disorder*.ts.	24,746

Cognitive Behavio?ral Therap*.ts.	0
Psychotherap*.ts.	170,929
Person centred therap*.ts.	59
Person-centred therap*.ts.	59
Counselling.ts.	263,707
Antidepressant medication*.ts.	10,993
Antipsychotic medication*.ts.	16,867
Antianxiety medication*.ts.	224
Psychotropic medication*.ts.	13,262
Mindfulness based cognitive therap*.ts.	1,064
Mindfulness-based cognitive therap*.ts.	1,064
Mindfulness based relapse prevention.ts.	77
Mindfulness-based relapse prevention.ts.	77
Mindfulness based stress reduction.ts.	1,504
Mindfulness-based stress reduction.ts.	1,504
Electroconvulsive therap*.ts.	24,984
Verbal de-escalation.ts.	33
Therapeutic.ts.	5,373,875
Functional Analys?s.ts.	103,764
Dialectical Behavio?r Therap*.ts.	0
Dysexecutive syndrome.ts.	1,183
ALL MENTAL HEALTH TERMS COMBINED WITH OR (MENTAL HEALTH FACET.ts.)	2,286,421
Patient safety.ts.	16,339
Adverse event*.ts.	90,069
Adverse drug event*.ts.	4,125
Sentinel event*.ts.	410
Incident*.ts.	181,024
Error*.ts.	749,209
Near miss*.ts.	1,899

Close call*.ts.	184
Never event*.ts.	157
Critical outcome*.ts.	188
Adverse outcome*.ts.	16,399
Unanticipated outcome*.ts.	118
Suicide*.ts.	53,306
Self-harm.ts.	4,264
Self harm.ts.	4,264
Behavio?r control.ts.	0
Restraint.ts.	31,162
Seclusion.ts.	1,236
Safety management.ts.	4,219
Failure to diagnose.ts.	382
Failure of diagnos?s.ts.	28
Under diagnosis.ts.	528
Over diagnosis.ts.	459
Misdiagnosis.ts.	7,360
Dual diagnos?s.ts.	2,232
Delay in diagnos?s.ts.	2,610
Wrong diagnos?s.ts.	302
Incorrect diagnos?s.ts.	825
Safety culture.ts.	1,752
Safety climate.ts.	1,070
Fall*.ts.	272,075
Slip*.ts.	94,412
Trip*.ts.	361,592
Accident prevention.ts.	1,225
Patient accident*.ts.	30
Patient in road traffic accident*.ts.	0

Collision with an object.ts.	8
Contact with an object.ts.	93
Contact with sharp*.ts.	15
Collision with sharp*.ts.	2
Exposure to hazardous substance*.ts.	279
Inappropriate patient handling.ts.	0
Inappropriate patient positioning.ts.	5
Elope.ts.	1,318
Wander.ts.	22,871
Runaway.ts.	16,779
Abscond*.ts.	1,444
Escorted leave.ts.	5
Unescorted leave.ts.	0
Aggressi*.ts.	577,758
Violence.ts.	208,902
Assault*.ts.	41,956
Abus*.ts.	475,050
Disruptive behavio?r.ts.	0
Racial attack*.ts.	19
Sexual attack*.ts.	96
Sexually inappropriate.ts.	169
Physical attack*.ts.	957
Verbal attack*.ts.	104
Missing patient*.ts.	259
Failure in access.ts.	4
Unexpected readmission*.ts.	61
Reattendance*.ts.	329
Unplanned admission*.ts.	892
Transfer to specialist care unit*.ts.	0

Delay in discharge.ts.	185
Failure to discharge.ts.	40
Inappropriate discharge.ts.	151
Planning failure.ts.	69
Self discharge.ts.	1,760
Self-discharge.ts.	1,760
Discharge against medical advice.ts.	333
Failure in referral process*.ts.	0
Failure to return from authorised leave.ts.	0
Transfer delay*.ts.	1,104
Transfer failure*.ts.	270
Inappropriate transfer*.ts.	93
Unsafe transfer*.ts.	3
Unsafe clinical environment*.ts.	4
Inappropriate clinical environment*.ts.	0
Inappropriate admission of a minor to an adult setting.ts.	0
Inappropriate transfer of a minor to an adult setting.ts.	0
Poor clinical assessment*.ts.	11
Lack of clinical assessment*.ts.	4
Lack of risk assessment*.ts.	11
Wrong scan*.ts.	1
Wrong x-ray*.ts.	1
Wrong specimen*.ts.	13
Inadequate scan*.ts.	47
Inadequate x-ray*.ts.	28
Inadequate specimen*.ts.	480
Incomplete scan*.ts.	53
Incomplete x-ray*.ts.	35
Incomplete specimen*.ts.	214

Mislabelled scan*.ts.	0
Mislabelled x-ray*.ts.	0
Mislabelled specimen*.ts.	27
Unlabelled scan*.ts.	7
Unlabelled x-ray*.ts.	8
Unlabelled specimen*.ts.	21
Missing scan*.ts.	32
Missing x-ray*.ts.	18
Missing specimen*.ts.	17
Failure to interpret test result*.ts.	3
Delay to interpret test result*.ts.	0
Failure to act on test result*.ts.	0
Delay to act on test result*.ts.	0
Failure to receive test result*.ts.	0
Delay to receive test result*.ts.	0
Incorrect test result*.ts.	38
Incorrect report*.ts.	135
Missing test result*.ts.	33
Missing report*.ts.	102
Failure to undertake test*.ts.	0
Delay to undertake test*.ts.	0
Patient confidentiality.ts.	1,656
Communication failure*.ts.	1,415
Failed communication*.ts.	89
Failure in communication*.ts.	55
Failure to receive informed consent.ts.	0
Inadequate handover.ts.	10
Documentation delay*.ts.	3
Mislabelled documentation.ts.	0

Missing documentation.ts.	61
Inadequate documentation.ts.	452
Wrong documentation.ts.	10
Illegible documentation.ts.	0
Mislabelled healthcare record*.ts.	0
Inadequate healthcare record*.ts.	0
Missing healthcare record*.ts.	0
Wrong healthcare record*.ts.	0
Illegible healthcare record*.ts.	0
Mislabelled referral letter*.ts.	0
Inadequate referral letter*.ts.	4
Missing referral letter*.ts.	1
Wrong referral letter*.ts.	0
Illegible referral letter*.ts.	0
Misfiled documentation.ts.	0
No access to documentation.ts.	0
Patient incorrectly identified.ts.	3
Delay in obtaining clinical assistance.ts.	0
Difficulty in obtaining clinical assistance.ts.	0
Delay in recogni?ing complication* of treatment.ts.	0
Failure in recogni?ing complication* of treatment.ts.	0
Delay in monitoring.ts.	7
Failure to monitor.ts.	213
Failure to follow up.ts.	204
Infection Control.ts.	64,988
Failure of sterili?ation of equipment.ts.	0
Contamination of equipment.ts.	83
Health care acquired infection*.ts.	197
Healthcare acquired infection*.ts.	282

Health care associated infection*.ts.	2,853
Healthcare associated infection*.ts.	4,684
Wound infection*.ts.	87,621
Surgical site infection*.ts.	17,940
Unsafe environment*.ts.	232
Inappropriate environment*.ts.	152
Unsafe equipment.ts.	38
Inappropriate equipment.ts.	55
Availability of equipment.ts.	620
Availability of bed*.ts.	188
Availability of IT.ts.	61
Staff shortage*.ts.	1,191
Unavailability of staff.ts.	13
Lack of skilled staff.ts.	31
Unskilled staff.ts.	50
Lack of suitably trained staff.ts.	1
Failure of device*.ts.	70
Failure of equipment.ts.	99
Unavailability of device*.ts.	3
Extended stay.ts.	341
Extended episode* of care.ts.	0
Failure to discontinue treatment*.ts.	4
Infusion injur*.ts.	8
Missing needle*.ts.	10
Missing swab*.ts.	4
Missing instrument*.ts.	17
Retained needle*.ts.	72
Retained swab*.ts.	34
Retained instrument*.ts.	51

Theatre list details incorrect.ts.	0
Inappropriate treatment*.ts.	3,815
Wrong treatment*.ts.	309
Unplanned return to theatre.ts.	40
Maternal death*.ts.	13,665
Anaesthetic complication*.ts.	544
Intensive Therapy Unit Admission*.ts.	21
Intensive Treatment Unit Admission*.ts.	1
Intensive Care Unit Admission*.ts.	7,192
Venous thromboembolism*.ts.	57,981
Pulmonary embolism*.ts.	99,763
Readmission of mother.ts.	0
Stillbirth*.ts.	31,245
Neonatal death*.ts.	16,007
Birth trauma*.ts.	2,988
Term baby admitted to neonatal unit.ts.	0
Undiagnosed f?etal abnormalit*.ts.	0
Pressure ulcer*.ts.	21,706
Padded room*.ts.	3
Ligature point*.ts.	20
Self-neglect.ts.	1,014
Self neglect.ts.	1,014
Splint*.ts.	35,353
Head bang*.ts.	400
Head-bang*.ts.	400
ALL PATIENT SAFETY TERMS COMBINED WITH OR (PATIENT SAFETY FACET.ts.)	2,302,638
Research.ts.	2,000,108
Academic work.ts.	934
Academic understanding.ts.	67

Theor*.ts.	2,452,690
Randomised controlled trial*.ts.	25,697
Controlled clinical trial*.ts.	20,913
Random allocation.ts.	1,283
Double blind method.ts.	260
Single blind method.ts.	37
Single blind stud*.ts.	910
Double blind stud*.ts.	11,890
Triple blind stud*.ts.	25
Multicentre stud*.ts.	5,746
Random sample*.ts.	26,972
Evidence base*.ts.	75,385
Evidence scan*.ts.	4
Systematic review*.ts.	79,601
Scoping review*.ts.	788
Narrative review*.ts.	2,987
Literature review*.ts.	57,967
Meta narrative*.ts.	262
Meta synthesi*.ts.	579
Meta-analys*.ts.	98,287
Clinical trial*.ts.	282,495
Placebo*.ts.	192,907
Comparative stud*.ts.	125,351
Evaluation stud*.ts.	6,414
Evaluative stud*.ts.	435
Descriptive stud*.ts.	12,395
Community trial*.ts.	605
Follow up stud*.ts.	37,174
Prospective stud*.ts.	113,856

Longitudinal stud*.ts.	61,357
Qualitative.ts.	263,183
Quantitative.ts.	691,755
Focus group*.ts.	32,648
Semi-structured interview*.ts.	19,484
Quality improvement project*.ts.	1,369
Data collection.ts.	65,477
Data analysis.ts.	89,562
Survey*.ts.	2,485,526
Observation*.ts.	3,260,138
Ethnograph*.ts.	70,967
Intervention*.ts.	2,204,162
Investigation*.ts.	3,070,213
Experiment*.ts.	11,775,074
Case stud*.ts.	826,192
Delphi.ts.	26,859
Nominal group technique*.ts.	1,802
Nominal group stud*.ts.	30
Consensus stud*.ts.	722
ALL RESEARCH TERMS COMBINED WITH OR (RESEARCH FACET.ts.)	11,281,165
Hospital*.ts.	647,964
Acute care.ts.	12,255
Secondary care.ts.	3,743
Tertiary care.ts.	27,052
Unit*.ts.	1,470,582
Ward*.ts.	40,230
Low secure.ts.	53
Medium secure.ts.	284
High secure.ts.	175

Secure facilit*.ts.	82
Forensic*.ts.	42,822
Inpatient*.ts.	67,899
Triage.ts.	11,510
ALL INPATIENT SETTING TERMS COMBINED WITH OR (INPATIENT SETTING FACET.ts.)	2,125,669
FINAL SEARCH RESULT: ALL FACETS COMBINED WITH AND (MENTAL HEALTH AND PATIENT SAFETY AND RESEARCH AND INPATIENT SETTING.ts.)	23,613

Final search string:

((Patient safety or Adverse event* or Adverse drug event* or Sentinel event* or Incident* or Error* or Near miss* or Close call* or Never event* or Critical outcome* or Adverse outcome* or Unanticipated outcome* or Suicide* or Self-harm or Self harm or Behavio?r control or Restraint or Seclusion or Safety management or Failure to diagnose or Failure of diagnos?s or Under diagnosis or Over diagnosis or Misdiagnosis or Dual diagnos?s or Delay in diagnos?s or Wrong diagnos?s or Incorrect diagnos?s or Safety culture or Safety climate or Fall* or Slip* or Trip* or Falling or Slipping or Tripping or Accident prevention or Patient accident* or Patient in road traffic accident* or Collision with an object or Contact with an object or Contact with sharp* or Collision with sharp* or Exposure to hazardous substance* or Inappropriate patient handling or Inappropriate patient positioning or Elope or Wander or Runaway or Abscond* or Escorted leave or Unescorted leave or Aggressi* or Violence or Assault* or Abus* or Disruptive behavio?r or Racial attack* or Sexual attack* or Sexually inappropriate or Physical attack* or Verbal attack* or Missing patient* or Failure in access or Unexpected readmission* or Reattendance* or Unplanned admission* or Transfer to specialist care unit* or Delay in discharge or Failure to discharge or Inappropriate discharge or Planning failure or Self discharge or Self-discharge or Discharge against medical advice or Failure in referral process* or Failure to return from authorised leave or Transfer delay* or Transfer failure* or Inappropriate transfer* or Unsafe transfer* or Unsafe clinical environment* or Inappropriate clinical environment* or Inappropriate admission of a minor to an adult setting or Inappropriate transfer of a minor to an adult setting or Poor clinical assessment* or Lack of clinical assessment* or Lack of risk assessment* or Wrong scan* or Wrong x-ray* or Wrong specimen* or Inadequate scan* or Inadequate x-ray* or Inadequate specimen* or Incomplete scan* or Incomplete x-ray* or Incomplete specimen* or Mislabelled scan* or Mislabelled x-ray* or Mislabelled specimen* or Unlabelled scan* or Unlabelled x-ray* or Unlabelled specimen* or Missing scan* or Missing x-ray* or Missing specimen* or

For peer review only

Failure to interpret test result* or Delay to interpret test result* or Failure to act on test result* or Delay to act on test result* or Failure to receive test result* or Delay to receive test result* or Incorrect test result* or Incorrect report* or Missing test result* or Missing report* or Failure to undertake test* or Delay to undertake test* or Patient confidentiality or Communication failure* or Failed communication* or Failure in communication* or Failure to receive informed consent or Inadequate handover of care or Inadequate handover or Documentation delay* or Mislabelled documentation or Missing documentation or Inadequate documentation or Wrong documentation or Illegible documentation or Mislabelled healthcare record* or Inadequate healthcare record* or Missing healthcare record* or Wrong healthcare record* or Illegible healthcare record* or Mislabelled referral letter* or Inadequate referral letter* or Missing referral letter* or Wrong referral letter* or Illegible referral letter* or Misfiled documentation or No access to documentation or Patient incorrectly identified or Delay in obtaining clinical assistance or Difficulty in obtaining clinical assistance or Delay in recogni?ing complication* of treatment or Failure in recogni?ing complication* of treatment or Delay in monitoring or Failure to monitor or Failure to follow up or Infection Control or Failure of sterili?ation of equipment or Contamination of equipment or Healthcare associated infection or Healthcare acquired infection or Health care associated infection or Health care acquired infection or Health care acquired infection* or Healthcare acquired infection* or Health care associated infection* or Healthcare associated infection* or Wound infection* or Surgical site infection* or Unsafe environment* or Inappropriate environment* or Unsafe equipment or Inappropriate equipment or Availability of equipment or Availability of bed* or Availability of IT or Staff shortage* or Unavailability of staff or Lack of skilled staff or Unskilled staff or Lack of suitably trained staff or Failure of device* or Failure of equipment or Unavailability of device* or Extended stay or Extended episode of care or Extended episode* of care or Failure to discontinue treatment* or Infusion injur* or Missing needle* or Missing swab* or Missing instrument* or Retained needle* or Retained swab* or Retained instrument* or Theatre list details incorrect or Inappropriate treatment* or Wrong treatment* or Unplanned return to theatre or Maternal death* or Anaesthetic complication* or Intensive Therapy Unit Admission* or Intensive Treatment Unit Admission* or Intensive Care Unit Admission* or Venous thromboembolism or Venous thromboembolism* or Pulmonary embolism* or Readmission of mother or Stillbirth* or Neonatal death* or Birth trauma* or Term baby admitted to neonatal unit or Undiagnosed f?etal abnormalit* or Pressure ulcer* or Padded room* or Ligature point* or Self-neglect or Self neglect or Splint* or Head bang* or Head-bang* or Patient safety) and (Research or Academic work or Academic understanding or Theor* or Randomised controlled trial* or Controlled clinical trial* or Random allocation or Double blind method or Single blind method or Single blind stud* or Double blind stud* or Triple blind stud* or Multicentre stud* or Random sample* or Evidence base* or Evidence scan* or Systematic review* or Scoping

For peer review only

review* or Narrative review* or Literature review* or Meta narrative* or Meta synthesi* or Meta-analys* or Clinical trial* or Placebo* or Research design or Comparative stud* or Evaluation stud* or Evaluative stud* or Descriptive stud* or Community trial* or Follow up stud* or Prospective stud* or Longitudinal stud* or Qualitative or Quantitative or Focus group* or Semi-structured interview* or Quality improvement project* or Data collection or Data analysis or Survey* or Observation* or Ethnograph* or Intervention* or Investigation* or Experiment* or Case stud* or Delphi or Nominal group technique* or Nominal group stud* or Consensus stud* or (Research or Descriptive research methods or Psychological research or qualitative research or mixed methods research or Experimental research or Applied research or research methodology or Medical research or Research implementation or Research design or Scientific research or Research findings or Sociological research or Research reports or Social research or Empirical research methods or Research methods or Social service research or Social welfare research or quantitative research or Research projects or Health services research)) and (Mental health or Mental well-being or Mental well being or Psychological well being or Psychological well-being or Mental disorder* or Mental illness* or Mental disease* or Anxiety disorder* or Delirium or Dementia or Dissociative disorder* or Factitious disorder* or Impulse control disorder* or Mood disorder* or Affective disorder* or Psychotic disorder* or Depressive disorder* or Neurotic disorder* or Personality disorder* or Conduct disorder* or Schizophreni* or Somatoform disorder* or Substance related disorder* or Clinical Psychology or Impulsive behavio?r or Adjustment disorder* or Eating disorder* or Sleep disorder* or Neuros?s or Psychos?s or Delusion* or Paranoia or Hallucination* or Addiction* or Dependence or Misuse or New psychoactive substance* or Legal high* or Depression or Panic disorder* or Phobia* or Health anxiet* or Bipolar disorder* or Alcohol abuse or Alcoholism or Obsessive compulsive disorder* or Obsessive thought* or Intrusive thought* or Post traumatic stress disorder* or Post-traumatic stress disorder* or Cognitive Behavio?ral Therap* or Psychotherap* or Person centred therap* or Person-centred therap* or Counselling or Antidepressant medication* or Antipsychotic medication* or Antianxiety medication* or Psychotropic medication* or Mindfulness based cognitive therap* or Mindfulness-based cognitive therap* or Mindfulness based relapse prevention or Mindfulness-based relapse prevention or Mindfulness based stress reduction or Mindfulness-based stress reduction or Electroconvulsive therap* or Verbal de-escalation or Therapeutic or Functional Analys?s or Dialectical Behavio?r Therap* or Dysexecutive syndrome or Mental health or mental disorders or Psychiatr*) and (Hospital* or Acute care or Secondary care or Tertiary care or Low secure or Medium secure or High secure or Secure facilit* or Forensic* or Inpatient* or Triage or (Acute hospitals or Mental health hospitals or hospitals) or In patients or Unit* or Ward*)).ts.

For peer review only

Online supplement 3

Research categories, subcategories by subsequent articles and reference

**Category**	**Subcategory**	**Articles** *(Reference number)*
Interpersonal Violence	Aggression	39 (*17-19, 22-24, 29-31, 37-45, 47-56, 61-62, 66-69, 74, 77-78, 81 , 86*)
	Violence	28 (20-21, 25-28, 32-36, 57-60, 63-65, 70-73, 75, 79-80, *84-85, 97*)
	Challenging Behaviour	10 (*87-96*)
	Violence and Aggression	4 (*46, 76, 82-83*)
	Critical Incidents	3 (*100-102*)
	Conflict	3 (*97-99*)
	Sexual Assault	2 (*103-104*)
	Agitation	1 (*105*)
	Abuse	1 (*106*)
	**Total**	**91 *(34%)***
Coercive Interventions	Restraint	19 (*109, 112-115, 119-120, 127, 129-131, 136-138, 144-146, 148, 150*)
	Seclusion	15 (*107-108, 116-117, 121-123, 128, 132-135, 139-141*)
	Attitudes to Coercion	10 (*159-168*)
	Seclusion & Restraint	8 (*118, 124-126, 142-143, 147, 149*)
	Containment	4 (*151-154*)
	Process of Coercion	4 (*110-111, 169-170*)
	Alternative Interventions	2 (*157-158*)
	Shielding	1 (*155*)
	Conflict	1 (*156*)
	**Total**	**64 *(24%)***
Safety Culture	Process	15 (*171-185*)
	Culture	14 (*186-199*)
	Policy	8 (*200-207*)
	**Total**	**37 *(14%)***
Harm to Self	Self-harm	13 (*208-220*)
	Suicidal Behaviour	12 (*220-232*)

For peer review only

	Self-neglect	1 ([233])
	**Total**	**26 *(10%)***
Safety of the Physical Environment	Security	5 ([234-238])
	Environment Design	3 ([239-241])
	Transitions of Care	3 ([242-244])
	Patient Distribution	3 ([245-247])
	Staffing	1 ([248])
	Ligatures	1 ([249])
	**Total**	**16 *(6%)***
Medication Safety	Adverse Events	4 ([250-253])
	Medication Administration	3 ([256-258])
	Medication Management	2 ([244-245])
	Medication Dispensing	1 ([259])
	Adherence	1 ([261])
	Substance Use	1 ([260])
	**Total**	**12 *(4%)***
Unauthorised Leave	Absconding	8 ([262-267, 270-271])
	Wandering	2 ([268-269])
	**Total**	**10 *(4%)***
Clinical Decision Making	Incident Management	4 ([274-277])
	Risk Assessment	2 ([278-279])
	Diagnosis	2 ([272-273])
	**Total**	**8 *(3%)***
Accidents	Falls	4 ([280-282, 285])
	Injuries	2 ([283-284])
	**Total**	**6 *(2%)***

For peer review only

Online supplement 4

Data extraction tables by research category

Interpersonal Violence

Author, year, country	Study design	Setting	Participants	Aims/ objectives related to patient safety	Outcomes related to patient safety	Study quality
Almvik, Rasmussen & Woods (2006) Norway	Observation/ Questionnaire/ Quantitative	Geriatric psychiatric wards and special care units for patients with dementia (SCU) within nursing homes (n=2)	Residents (n=82); 43.9% male; mean age 81.96 (range 56-104).	To explore violent incidents in an elderly population	39% of patients were reported to be violent. The mean score on the SOAS-R severity scale was 9.89 (median 10, SD 5.00, 95% CI 9.21–10.56). The situations where the client was denied something were the most provocative ones (37.9% of occurrences). Verbal aggression was the most frequent type of attack (58%). The staff were the most frequent targets of the violent incidents (78.8%). The incident had no consequences for the victim in 47.6% of cases. Talking to patients was the most common measure taken to stop aggression (63.7%). Most incidents occurred in the morning (42.9%).	Fair
Amoo & Fatoye (2010) Nigeria	Questionnaire/ Quantitative	Inpatient psychiatric wards of a neuropsychiatric hospital	Psychiatric inpatients (n=305); 69.8% male. Age range of the aggressive patients 13-66.	To investigate aggressive behaviour and related variables in psychiatric inpatients	43 patients manifested aggressive behaviour (13.8% of sample). Most aggressive behaviour occurred during evening and night periods (69.8% of incidents) and was evenly distributed throughout the week. Physical aggression was the most common type of behaviour (37.2%) and female nurses were the most common targets. Hallucinations	Fair

For peer review only

					were the most common probable cause (34.9%).	
Bahareethan & Shah (2000) UK	Questionnaire/ Quantitative	Continuing care psychogeriatric wards within a London hospital (n=2)	Patients (n=39); 26% male. Median age 83 (range 68-99).	To investigate aggressive behaviour and staff perception and attitudes in psychogeriatric wards	There was a moderate correlation between aggressive behaviour (RAGE scale) and staff perception of the patient (MAS scale) (r=+0.67, p<0.00001). The highest correlation was between the RAGE score and the MAS subscale of 'alienation of the patient (r=+0.85, p<0.00001).	Fair
Bennett, Ramakrishna & Maganty (2011) UK	Questionnaire/ Quantitative	A psychiatric intensive care unit (PICU) within a medium-secure unit	Nursing and multidisciplinary staff of the PICU (n=43)	To investigate staff views of different management techniques for disturbed behaviour in PICUs	Individual counselling was the preferred choice of management (n=40, mode rank given=1; mean rank given=1.25; median rank given=1; SD=0.77)	Fair
Berg, et al. (2013) Europe	Interviews/ Qualitative	Forensic psychiatric units (n=4) in Belgium (n=1) Finland (n=1), Netherlands (n=1) and the UK (n=1)	Staff in the units (n=58). Mean age 36 (range 20-75)	To investigate staff perceptions of aggressive behaviour in forensic psychiatric patients	Participants described aggressive behaviour as consisting of verbal, non-verbal and physical dimensions. Staff being authoritative with patients was described as provoking aggressive behaviour and more commonly leading to physical management techniques. Limit-setting situations were also identified as provoking aggressive behaviour towards staff.	Good

For peer review only

Berg, Kaltiala-Heino & Välimäki (2011) Europe	Interviews/ Qualitative	Forensic psychiatric units (n=4) in Belgium (n=1) Finland (n=1), Netherlands (n=1) and The UK (n=1)	Staff in the units (n=58). Mean age 36 (range 20-75)	To explore practices for managing adolescent aggressive behaviour in forensic psychiatric units	Participants favoured the use of verbal and less restrictive aggression management interventions. The use of coercive management interventions was described as a last option. Participants described using knowledge of the patient, the level of aggression, the situation development and available resources to decide on appropriate action. Countries differed on the practical responses to aggression but shared the same basic components.	Good
Bharwani, et al. (2012) USA	Observation and questionnaire/ Mixed methods	An Alzheimer unit within a continuing care retirement community	Residents of the unit (n=18). 54% female. Average age 82	To explore the benefits of a Behaviour-Based Ergonomic Therapy (BBET) program designed as an intervention for challenging behaviour	Resident falls decreased by 32.5% during the first 6 months of BBET implementation; from an average of 6.67 falls per month (n=40 in the 6 months before implementation) to 4.5 falls per month (n=27 in the first 6 months of implementation). Staff and family members commented on a reduction in agitation, including emergency calls about resident behaviour.	Fair
Biancosino, et al. (2009) Italy	Questionnaire and chart review/ Quantitative	Public (n=113) and private (n=32) acute psychiatric inpatient facilities	Psychiatric inpatients in the facilities (n=1324). 51% male. 28.2% were 35-44.	To examine the nature and predictors of aggressive behaviour in psychiatric inpatients	6.3% of patients had been physically restrained. Violent patients were more likely to be restrained than non-violent patients (up to 51.4% of this group (χ2= 203.07; p<0.001). No association with violent behaviour was found when treatment setting variables were examined (restrictiveness,	Fair

For peer review only

					standardization of the process of care, treatment, and staff number/qualification).	
Björkdahl, Hansebo & Palmstierna (2013) Sweden	Non-randomised pre-post/ Quantitative	Psychiatric inpatient units (n=41). Emergency and admission wards (n = 2), general wards (n = 30), psychiatric intensive care units (n = 2), drug and alcohol dependence wards (n = 2) and forensic wards (n = 5).	Staff in the units (n=854 before training; n=260 after). 40% male before training; 42% male after. Inpatients (n=297 before training; n=156 after). 55% male before training; 51% male after. Age range <25->40.	To explore the influence of a violence prevention and management staff-training programme on the climate related to violence management in psychiatric inpatient wards.	Staff perceived the violence management climate as significantly more positive on the trained wards compared with the wards that had not yet been trained, Mann–Whitney P=0.045. Specifically, the areas viewed more positively were: ward rules (odds [OR] = 1.97, P=0.001), the emotional regulation of staff members in challenging situations (odds [OR] = 2.24, P=0.007), the staff's interest in possible causes for patient aggression (odds [OR] = 1.64, P=0.031) and the staff's readiness to intervene at an early stage of patient aggression (odds [OR] = 2.10, P=0.001). On the trained wards, patients viewed perceptions of the staff's interest in finding possible causes for patient aggression significantly more positively (odds [OR]= 1.98, P=0.022)	Fair

For peer review only

Boström, et al. (2012) Canada	Questionnaire/ Mixed methods	Resident care units representing two models of residential care, Residential Alzheimer's Care Centres RACCs (n = 2) and Secured Dementia Units SDUs (n = 2).	Staff in the units (n=91). 98% female.	To explore aggressive incidents experienced by frontline staff in two types of residential care units	The most frequent type of aggression was physical assault (50% of staff reported experiencing this). 79% of incidences were officially reported by staff. Aggressive incidents were associated with working in SDUs rather than RACCs in a regression model (F =4.667, df = 6, p < 0.001). Other factors associated with aggressive incidents were: staff educational level, experience, exhaustion, staffing levels and staff informal interactions.	Fair
Bowers (2009) UK	Cross-sectional/ Quantitative	Acute psychiatric wards (n=136) in 67 hospitals within 26 National Health Service (NHS) Trusts	All staff and patients within the units	To explore the effect of the positive appreciation of patients by staff, the staff's management of their own emotional reactions to patient behaviour, and the provision of an effective structure on conflict in acute psychiatric wards.	Only 18% of the variance in containment rates was explained by conflict. The regression model found that effective structure and order on the ward (the order and organization subscale and the program clarity subscale of the WAS) was the factor most strongly predictive of lower conflict and containment rates (Coefficient=-0.048, p=0.048).	Good
Bowers, et al. (2009) UK	Questionnaire/Quantitative	Acute psychiatric wards (n=136) in 67 hospitals within 26 National Health Service (NHS) Trusts	All staff and patients within the units	To explore the relationship of patient violence to other patient characteristics, features of the service and physical environment and staff factors.	Higher levels of aggression were associated with high patient turnover, alcohol use by patients, ward doors being locked, and higher staffing numbers (especially qualified nurses).	Fair

For peer review only

Calabro, Mackey & Williams (2002) USA	Pre-post evaluation/Quantitative	An acute care psychiatric hospital	Staff (n=118). 67% female. Modal age group 40-49	To evaluate the effectiveness of a training program to prevent and manage patient violence within acute psychiatric wards	There were improvements in post-test measures for: knowledge (t[109]=7.29, p < 0.001); attitude (t[109]=-5.68, p < 0.001); self-efficacy (t[114]=-2.82, p < 0.01); and intention to use the training techniques (t[114]=-1.99, p < 0.05).	Fair
Camuccio, et al. (2012) Italy	Focus groups/ Qualitative	Acute psychiatric inpatient units (n=7)	Nurses (n=33). 70% female. Mean age 40.	To explore the experiences of acute psychiatric inpatient unit nurses when managing aggressive and distressed patients	The predominant theme was of fear, and this consisted of: fear of harm to self or patient; team safety; the known/unknown patient; non-psychiatric patients; and patient physique. Co-ordination with other services was expressed as key to good quality care.	Fair
Carlson, et al. (2010) USA	Observation, chart review and semi-structured interviews/ Quantitative	A psychiatric inpatient unit	Patients (n=130; n=151 admissions). 80% male. Mean age 9.64 ± 2.09	To examine the safety and efficacy of liquid risperidone to reduce rages in children admitted to a psychiatric inpatient unit.	75% of the sample had multiple rages in hospital. No adverse events related to the medication were observed. In the 16 children who had more than 3 rage outbursts during hospitalisation, there was a significant drop in duration of the outbursts from the non-medicated state to the highest dose state (t(15)=3.43, p<0.004).	Fair

For peer review only

Caspi (2015) USA	Observation, chart review and semi-structured interviews/ Qualitative	Special dementia care units within an assisted living residence (n=2)	Residents (n=12). 92% female. Mean age 81.	To explore aggression between residents (ABBR) with dementia within assisted living residences	For the majority of ABBR incidents there were observable causes and triggers (77% in one unit; 57% in the other). The most common cause was problematic seating arrangement (n=17). There were 12 effective staff management strategies: being alert; being proactive; being informed about aggression history; redirecting residents; offering to take a walk; separating; positioning, repositioning, or changing seating arrangement; refocusing; distracting the person; staying calm; never arguing with a resident; seeking help from other staff members.	Good
Caspi, et al. (2001) Israel	Double-blind crossover trial/ Quantitative	A maximum security unit within a mental health centre	Schizophrenic inpatients (n=30). 100% male. Mean age 37.2 ±9.6 (range 20-65)	To examine the efficacy of augmenting antipsychotic treatment with pindolol to reduce aggression in schizophrenic inpatients.	Pindolol treatment significantly reduced the number of aggressive incidents towards objects and other persons (0.59 versus 1.46, F=6.09, P<0.02; 1.96 versus 3.23, F=4.17, P<0.05, respectively); and reduced the severity of the incidents (0.89 versus 3.58, F=19.42, P<0.0001; 2.89 versus 6.85, F=10.11, P<0.004, respectively).	Fair

For peer review only

Chan, et al. (2005) Hong Kong	Pre-post questionnaire/ Quantitative	A developmental disability unit within a mental health hospital	Patients (n=89). Experimental group: 60% female. Control group: 59% female. Modal age group 31-40	To evaluate the impact of multisensory therapy on the challenging behaviour of patients with developmental disabilities	Both groups of patients experienced reductions in levels of challenging behaviour over time. Between the mid- and post-1 assessments, the control group had greater reductions in challenging behaviour (F=4.26, p=0.04). Between the post-2 and post-3 assessments, the experimental group had greater reductions in challenging behaviour (F=4.22, p=0.04).	Fair
Chaplin, et al. (2008) UK	Questionnaire/ Mixed methods	Older adult (OA) wards (n=75) and adults of working age (WAA) wards (n=88) within 66 English and Welsh NHS trusts and independent sector providers	Staff and patients (n=3,332)	To explore physical aggression/assault on psychiatric wards	Nurses on WAA wards were more likely to report dealing with aggressive incidents (85%, x^2=11 p=0.0006); using rapid tranquilisation (68%, x^2=73 p<0.0001); receiving adequate training in aggression management/prevention (79%, x^2=6.5 p=0.011); and having a personal alarm (86%, x^2=35 p=0.0001) than nurses on OA wards. The frequency of restraint was the same between the two types of wards. Provision of ward based activity and therapy were significantly better for patients on OA than WAA wards.	Poor

For peer review only

Chen, Hwu & Wang (2009) Taiwan	Questionnaire/ Quantitative	A psychiatric hospital	Staff (n=222). 53% female. Modal age group 30-44.	To explore staff responses to workplace violence in a psychiatric hospital	Staff experiencing verbal abuse were most likely to take no action (27%). Staff experiencing sexual harassment were more likely to report the incident to senior staff than staff experiencing other types of violence (76%). The most common form of management following an incident of physical violence or racial harassment was medication injection (29% and 30%); and for incidents of verbal abuse, bullying/mobbing and sexual harassment it was issuing a verbal warning (30%, 29% and 29%). The most common reason for not reporting violent incidents was that it was considered unimportant.	Fair
Chen, et al. (2007) Taiwan	In-depth interviews/ Qualitative	A psychiatric hospital	Staff victims of assault (n=13). Age range 23-60.	To explore the effects of assault on staff caring for schizophrenic patients in a psychiatric hospital	All staff reported that they did not receive enough post-incident support. The most common time for violence to occur was during ward inspections when staff were alone. The most severe psychological harm to staff was post-traumatic stress disorder. Staff gave suggestions for preventing further attacks: pre-placement training, good practice (asking for consent, not being alone, taking precautions and respecting patients' rights), and addressing organisational culture.	Fair

For peer review only

Arguvanli, et al. (2015) Turkey	Pre-post questionnaire/ Quantitative	Psychiatric inpatient clinics within hospitals (n=2)	Nurses (n=27). 78% female. Mean age 33.2±5.4 (range 22-47)	To evaluate the Aggression Management Training Program (AMTP) in psychiatric inpatient clinics	96.3% of participants reported that they did not receive aggression management training. Participant knowledge and functional reactions to aggression increased significantly from pre-post intervention.	Fair
Cole, Baldwin & Thomas (2003) UK	Questionnaire/ Quantitative	Acute inpatient wards (n=3), a rehabilitation ward (n=1), and an intensive care ward (n=1) within a general adult psychiatric unit	Staff (n=109)	To explore staff practices and emotions concerning patient sexual assault in a psychiatric unit	The actions that most participants felt should be taken following a sexual assault were recording in notes (98.1% agreed) and informing teams involved (98.1% agreed). The emotional response to patient sexual assault that most participants reported feeling 'a lot/extremely' was being calm (38.3%). the majority of participants worried about being blamed in the instance of a sexual assault.	Fair
Cutcliffe (1999) UK	Semi-structured interviews/ Qualitative	An inpatient unit within a psychiatric hospital	Qualified nurses (n=6)	To explore the experiences of nurses who experience violence perpetrated by psychiatric patients.	3 key themes emerged: personal construct of violence, feeling equipped, and feeling supported. A relationship was found between exposure to violence and nurse's ability to manage these therapeutically. This is influenced by formal systems in place within the unit.	Fair
Daffern (2007) UK	Questionnaire/Qualitative	A high secure Dangerous and Severe Personality Disorder (DSPD) service within a secure hospital	Nursing staff and patients (n=18)	To explore nursing staff ability to assess the function of psychiatric patients' aggressive behaviour	The nursing staff's assessment of the functions of aggression differed from the patients' most significantly for the following: to enhance status or social approval, to obtain tangibles, and to observe suffering.	Fair

For peer review only

Daffern, et al. (2009) UK	Pre-post questionnaires/Quantitative	A high secure Dangerous and Severe Personality Disorder (DSPD) service within a secure hospital	Patients (n=51). Mean age 34 (range 18-54)	To evaluate whether structured risk assessment followed by results and management recommendations can reduce the frequency of aggression in DSPD patients	There was no significant difference in the frequency of reported aggression across the phases of the intervention. Staff reported that they did not feel the structured risk assessment was superior to clinical judgement and did not use it regularly in the management of aggression.	Fair
Daffern, Mayer & Martin (2006) Australia	Longitudinal/ Quantitative	Acute (n=2) and continuing care (n=1) wards within a secure forensic psychiatric hospital	Staff and patients	To explore the relationship between staff gender ratio and incidents of aggression in acute psychiatric wards	Verbal aggression was the most common form of aggression (n = 196, 62% of incidents). Victims were mostly staff (70%). Staff gender did not significantly affect the frequency or severity of aggressive incidents, or the likelihood of seclusion being used.	Fair
Daffern, Ogloff & Howells (2003) Australia	Longitudinal/ Quantitative	Acute (n=3) and continuing care (n=2) wards within a secure forensic psychiatric hospital	Staff and patients	To explore aggression and seclusion in secure psychiatric settings and to examine the reporting of aggression using aggression-specific recording instruments	Verbal aggression was the most common form of aggression (n = 205, 62% of incidents). Victims were mostly staff (n=229). 21% of aggressive incidents resulted in seclusion. There was no significant relationship between seclusion and severity of incidents, victim type (staff or patient), time of day or number of incidents in the preceding week.	Fair

For peer review only

De Niet, Hutschemaekers & Lendemeijer (2005) Netherlands	Quasi-experimental time-series/ Mixed methods	A closed ward of a psychiatric institution	Nursing staff (n=23)	To explore reasons for the decline in the amount of reported aggression incidents when using the Staff Observation Aggression Scale (SOAS)	The number of reported aggression incidents decreased over the course of the intervention (t=2.598, df=55, P=0.012). Nursing staff did not perceive a reduction in the amount of aggression incidents, and stated that the SOAS did not add anything to the aggression management process, hence the reduction in reported incidents using this form.	Fair
Delaney, et al. (2001) Australia	Questionnaire, focus groups and chart review/ Mixed methods	Acute inpatient facilities (n=4)	Survey: nursing staff (n=59). 56% male. Modal age group 41-50. Focus groups: nursing staff	To explore the management of aggressive patients in psychiatric inpatient settings	88% of participants had been assaulted, with the majority (53%) stating that they had not attended aggression management training in the past year. Participants felt that history/background information of the patient, ongoing assessment, individualised care, peer support, policies/manual accessibility and stress management were key to effective aggression management.	Fair
De Young, Just & Harrison (2002) USA	Questionnaire/ Quantitative	A behaviour management unit within a long-term care facility	Patients (n=32). 53% male. Mean age 73 (range 38-95).	To evaluate a program designed to reduce the frequency of aggressive, agitated or disruptive (AAD) behaviours in a behaviour management unit	The most common AAD behaviours were resisting care and becoming upset/losing temper. There was a significant reduction in AAD incidents over the 6 month intervention period (t (df 18) =4.47, p=0.01). Staff reported using timing strategies to manage AAD behaviours most frequently.	Fair

For peer review only

Dickens, Piccirillo & Alderman (2013) UK	Questionnaire/ Quantitative	Low and medium secure wards of adult mental health care pathways of men's and women's services of a healthcare trust	Staff (n=72). Patients (n=98). 57% male. Mean age 34 (range 18-65)	To explore staff and patient attitudes concerning the management of violence and aggression in a secure, forensic mental health service	Patients and staff agreed on most internal causes of aggression, but differed on 2 points: patients agreed that it is difficult to prevent aggression and that being left alone can calm aggressive patients, whereas staff disagreed. Staff were more likely than patients to agree that external factors were causative of aggression. Patients and staff agreed on most situational/interactional causes of aggression, but patients felt that aggression could happen when staff do not listen, whereas staff disagreed with this. Patients and staff agreed on most management techniques, but patients felt that seclusion/restraint was sometimes overused, whereas staff did not, and patients felt that medication should be used more, whereas staff did not.	Fair
Duxbury & Whittington (2005) UK	Questionnaire/ Mixed methods	Inpatient mental health wards (n=3)	Nurses (n=80; 76% female) and patients (n=82; 51% female)	To explore the views of staff and patients concerning the causes and management of aggression in inpatient mental health wards	Both patients and staff agreed that external factors (restrictive environments) contributed to aggression. Staff were more likely to agree that a patient's illness contributed to aggression. Patients were more likely to agree that interactions/situations contributed to aggression. Staff were more likely to endorse management techniques	Fair

For peer review only

					such as medication and seclusion, although both groups agreed that restraint was an inevitable part of keeping the wards safe.	
Evans & Petter (2012) UK	Questionnaires/ Mixed methods	A PICU	Staff (n=15). 60% male. Age range 30-50.	To explore staff perceptions of violence and how safe they feel on a PICU	Overall staff reported feeling safe and supported at work. Themes arising from the interviews highlighted personal and systemic factors that relate to feeling safe and supported. The most commonly mentioned personal factors were: aggression as illness related, and nurses remaining emotionally unaffected. The most commonly mentioned systemic factors were: fear of letting the team down, lack of senior management support (fear of blame), reliance on external staff as increasing risk, and first admissions being higher risk.	Fair
Foley, et al. (2003) USA	Structured interview/ Mixed methods	Special care dementia units (n=53) within nursing facilities (n=49)	Staff (n=32)	To identify what differentiates successful from unsuccessful behaviour management of dementia patients in SCUs	Factors were found to be associated with successful management of behaviour problems: behaviour prevalence/severity, management techniques, use of psychotropic medication/restraint, comorbidity, and family interaction.	Fair

For peer review only

Gallop, et al. (1999) Canada	Semi-structured interviews/Qualitative	Acute care psychiatric units within hospitals (n=3)	Patients (n=20). 100% female.	To explore safety concerns amongst abused women in inpatient psychiatric environments	The most prevalent concerns raised were: mixed-gender units (preferring separate areas), night-time routines (restricting medications and staff contact at night), and primary nursing (as important for feeling safe). Participants expressed a desire to be involved in decision making.	Fair
Giles et al. (2005). USA	Descriptive/Quantitative	Locked skilled nursing facility	Psychiatric inpatients (n=40); 27% female; mean age 46 (range 21-71)	To examine the effectiveness of a programme to reduce aggression in inpatients	The programme resulted in a small reduction in aggressive incidents.	Fair
Hallett & Dickens (2015) UK	Cross-sectional/Mixed methods	Secure inpatient mental health unit	Clinical staff (n=72); 50% female	To explore perspectives of clinical staff about de-escalation including interventions used in de-escalation, what staff believe constitutes de-escalation and which interventions are effective	Half of the staff suggested medication was used as an intervention for de-escalation. 15% incorrectly stated that interventions such as seclusion, restraint and emergency intramuscular medication could be used as a de-escalation technique. Different aggression types were identified as needing different interventions and a personalised approach.	Fair
Higueras et al. (2006) Spain	Experimental/ Quantitative	Inpatient psychiatric ward of a general hospital	Psychiatric inpatients; baseline (n=83); mean age 41; 40% female	To examine the effects of a humour-based activity on disruptive behaviours	Disruptive behaviour significantly reduced after the intervention compared to baseline. Specifically, attempted escape, self-harm and fighting significantly reduced.	Fair

For peer review only

Ilkiw-Lavalle & Grenyer (2003) Australia	Mixed-methods design/ Mixed-methods	Psychiatric inpatient unit (n=4)	Staff (n=29); 48% female; mean age 33 and 29 inpatients (n=29); 35% female; mean age 31	To examine views of staff and patients involved in incidents of aggression to understand emotions experienced, perceptions of causes and recommendations for reducing aggression	Staff and patient views differed on the cause of the aggression: staff felt it was the patient's mental illness and patient's felt illness, interpersonal and environmental factors were equally to blame for the aggression. Overall participants were satisfied with the management of aggressive incidents.	Fair
Ireland, Halpin & Sullivan (2014) UK	Semi-structured interviews/ Qualitative	Forensic psychiatric inpatient hospital	Male inpatients (n=16); mean age 31	To examine motivations for forensic patients' engagement in critical incidents	The main themes included engaging in critical incidents to seek deliberate isolation, to gain control, to get needs met, a need to communicate and because they were influenced by peers.	Fair
Jacob et al. (2013) India	Observational/ Quantitative	Child and adolescent inpatient service	Children inpatients (n=31); 16% female; Mean age 12.9	To identify characteristics of aggressive incidents and how they are managed	Almost a quarter of all inpatients (n=131) demonstrated aggression (23.7%). Aggression was most likely to occur at night and against family members	Fair
Janicki (2009) UK	Semi-structured interviews and case note review/ Mixed methods	Women's medium secure hospital	Participants (n=15); patients (n=6), nurses (n=3); and other professionals (n=6)	To explore staff and patient perspectives on the involvement of the criminal justice system as a response to an assault on the medium secure ward	Overall, participants felt the involvement of the CJS was essential to ensure high morale and that incidents were dealt with so that this deterred other patients from committing assault	Fair
Jeffs et al. (2012) Canada	Explorative/ Qualitative	Inpatient mental health care agencies (n=3)	Staff: Site A (n=24), site B (n=19), Site C (n=24); patients: site A (n=9), site B (n=9), site C (n=10)	To gain insight into how service providers and service users experience and define near misses.	Overall near misses were identified as safety threats and issues associated with patients' mental illness and also situations that avoid harm to others and prevent an incident	Good

For peer review only

Killick & Allen (2005) UK	Experimental/ Quantitative	Psychiatric inpatient unit	Experimental staff group (n=27); 66.7% female; 40.9 mean months' work experience [89.2]	To assess the effects of three training courses on managing aggression, staff knowledge and satisfaction before, during and after training and at one year follow-up	Staff knowledge and confidence in managing aggression increased following training but was not maintained at 1 year follow-up	Poor
Koukia et al. (2013) Greece	Cohort design/Qualitative	Psychiatric hospital (n=3)	Nurses (n=26); 62% female; mean age 36 years [7.7] (range 23-54)	To identify nurses' interventions, views, and attitudes concerning critical incidents.	Nurses reported six interventions included counselling, performing security practices, monitoring thought disturbances, contacting the psychiatrist on-call, contacting the chief nurse on-call and administrating medication.	Fair
Lantta et al. (2015) Finland	Intervention design, literature review, case-note review and focus groups/ Mixed methods	Mental health inpatient units (n=3)	Nurses (n=22)	To explain the intervention of implementing a structured violence risk assessment procedure in mental health inpatient units using the Ottawa Model of Research Use (OMRU) as a guiding framework. To also consider nurses' perspectives of its clinical utility and implementation process	Some staff felt the model was useful but it was less preferred than nurse's own clinical judgement in some instances	Poor
Lanza et al. (2016) USA	Experimental/ Quantitative	Locked psychiatric units (n=7)	Violence Prevention Community Meeting group (n=4) and control group (n=3).	To assess the violence prevention community meeting (VPCM) as an effective intervention to reduce workplace violence in acute care psychiatric units	Per week aggression rates reduced slightly in the VPCM hospitals (0.6% (95% CI: -5.6%, 6.5%; non-significant)), but reduced significantly more in the control hospitals (5.1% (95% CI: 0.4%, 9.6%; significant)).	Poor
Lanza et al. (2009) USA	Pre-post experimental design/Quantitative	Acute psychiatric inpatient unit at a Veterans Affairs hospital	Male patients. Mean age 42.6. Staff (n=21)	To test the efficacy of the VPCM for reducing patient violence	There was a decrease of physical violence of 89% from pre-treatment to treatment and a decrease of 57% from pre- to post-treatment.	Fair

For peer review only

Lawn & Pols (2003) Australia	Mixed-methods	Locked psychiatric ward	Inpatients (n=24). Staff (n=26)	To explore experiences regarding smoking and violence in locked inpatient settings	There was conflict between staff and patients over staff controlling the supply of cigarettes. This was made worse by the physical structure of the smoking area.	Poor
Lehmann, McCormick & Kizer (1999) USA	Descriptive/ Quantitative	Veterans medical centres and free-standing clinics (including inpatient psychiatric units) (n=166)	N/A	To examine the scope and impact of violence in hospital settings	During October 1990 to 1991 there were 6,592 incidents of physical assault in inpatient psychiatric units. The incident rate per 100,000 patient days was 177.9, equating to the highest proportion across medical units. A quarter of staff recommended more training (24.1%)	Poor
Lipscomb et al. (2012) USA	Questionnaire/ Mixed methods	Residential addiction treatment centres (ATCs) (n=13)	Staff (n=409); 59% female	To examine the relationship between violence prevention safety climate measures and self-reported violence toward staff.	Predictors of violence included management commitment to violence prevention as “never/hardly ever” (OR=4.30), client actively resisting program (OR=2.34) and working with clients with unknown history of violence (OR=1.91)	Fair
Lowe, Wellman & Taylor (2003) UK	Vignette and questionnaire/ Quantitative	Acute psychiatric inpatient unit	Nurses (n=70)	To examine nurses response to scenarios of conflict incidents	Autonomy and limit-setting were acknowledged as the most notable responses to incidents of conflict. Across nurses these were most likely to cause disagreement.	Fair
McCann, Baird & Muir-Cochrane (2014) Australia	Questionnaire design/ Quantitative	Locked old age psychiatric inpatient units (n=3)	Staff (n=85). Registered nurses (61.1%, n = 52), enrolled nurses (27.1%, n = 23) and medical and allied health staff (11.8%, n = 10); 66% female; Mean age 43 (range 24-62 years)	To examine the attitudes of clinical staff toward the causes and management of aggression	Causes of aggression were multifactorial. Staff felt patient factors did not contribute to aggression. They felt other patients and staff contributed to aggression and specific cultural groups were more likely to be aggressive. Participants differed in their view of prevention strategies for	Fair

For peer review only

					aggression. Medication was deemed the more viable option but staff were undecided on the appropriateness of seclusion.	
McLaughlin et al. (2010) UK	Pre-post intervention study/ Quantitative	Acute psychiatric inpatient ward	Nurses (n=18); median age 33 (range 23-52)	To examine the potential effectiveness of an intervention for managing verbal aggression	Post intervention focus group analysis revealed there was an increase in staff diffusing the situation to manage verbal aggression	Poor
Meaden, Hacker & Spencer (2013) UK	Interview/ Quantitative	High dependency inpatient units	Nurses (n=25); 48% female	To assess the ability of an adapted early warning signs type of dynamic risk assessment to more accurately predict aggressive incidents	Receiver Operating Characteristic (ROC) analyses revealed that the assessment had moderate predictive validity regarding aggression (AUC=0.5) but effect size was small.	Fair
Meehan, McIntosh & Bergen (2006) Australia	Focus groups/ Qualitative	High Secure Forensic Unit (HSFU)	Patients (n=27); 23% female	To explore the patient perspective on aggressive behaviour	There were five main themes that explored the cause of aggression: the environment, empty days, staff interactions, medication issues and patient centred factors. Potential management to reduce aggression was identified as improved training, separation of acutely disturbed patients, early intervention, improving staff attitudes and implementation of effective justice procedures.	Fair

For peer review only

Murphy & Siv (2007) USA	Pre-post questionnaire/ Quantitative	Child and adolescent residential treatment unit	Patients (n=20). Average age in treatment as usual (TAU) group 14.8 and in Mode Deactivation group 15.7	To replicate previous findings supporting the effectiveness of Mode Deactivation Therapy (MDT).	The results showed MDT to be more effective then TAU in reducing both physical aggression and therapeutic restraints. MDT showed a reduction of 66.8% in physical aggression compared to TAU (27.9%). MDT showed a reduction of 70.7% in therapeutic holds compared to TAU (24.7%).	Fair
Needham, et al. (2005) Switzerland	Randomised controlled trial/ Quantitative	Acute psychiatric inpatient wards (n=6)	Nurses in control group (n=28); 50% female; mean age 39.1. Nurses in treatment group (n = 30); 60% female.	To investigate the effects of a training course in the management of aggressive behaviour in psychiatric acute inpatient settings.	No statistically significant differences of the means were found between the intervention and control groups on the positive perception of aggression (p=0.912), the negative perception of aggression (p=0.315), or the tolerance scale (p= 0.614).	Fair
Needham, et al. (2004) Switzerland	Feasibility study/ Quantitative	Acute mental health care inpatient settings (n=2)	Nurses. Patients (n=576); 41% females, mean age 38, age range 15–88	To implement a risk-prediction procedure and a standardized aggression management intervention.	There was a significant reduction in the percentage of days with attacks against persons (trend-test P = 0.04) and percentage of days with usage of coercive measures against any patient (trend-test P =0.01).	Good
Nolan, et al. (2009) USA	Questionnaire/ Quantitative	Specialised research unit	Patients	To describe the reasons for aggressive behaviour reported by patients and staff and the relationships between those reasons and the subsequent interventions delivered in response to aggression.	Patients reported more often than staff that aggression was caused by external factors. Staff cited internal factors more often than patients. Responses to aggression were not related to the cause of the behaviour.	Fair

For peer review only

Olsson, et al. (2015) Sweden	Semi-structured interviews/ Qualitative	A maximum security forensic psychiatric hospital	Patients (n=12); 83% male; mean age 37.	To examine the experiences of forensic inpatients that have decreased their risk of becoming violent in forensic care.	3 themes emerged: 1) Staff's attitudes and actions. Patients' perception of staff´s ability to manage conflicts. 2) Patients' own insight and actions: Being insightful and managing the situation Dealing with aggression Attending to signs of warnings. 3) Interactions in the health care environment: Experiences of the physical environment- participants found overcrowded wards to be stressful and anxiety-provoking.	Fair
Park & Lee (2012) South Korea	Non-random experiment/ Quantitative	A psychiatric hospital	Inpatients in experimental group (n=22) and control group (n=22). 100% male (age range 30-60)	To measure the effect of behaviour modification using Short-Term Token Economy (STTE) on aggressive behaviour.	In the experimental group, aggressive behaviour decreased by 20.8% compared with the comparison group after STTE. The number of verbal attacks decreased by 27.6%. Property damage or physical attacks decreased by 14.3%.	Fair
Phillips (2011) UK	Focus groups/ Qualitative	Inpatient wards	Mental health staff	To discuss issues raised during workshops aimed at encouraging and enabling staff to work in a gender-sensitive way to develop strategies for decreasing sexual assault incidence for female patients who may have histories of being abuse victims.	Staff taking part in the workshops expressed the importance of maintaining boundaries, developing adequate policies, and one-to-one observation. Issues regarding the truth of allegations and roles of the female patients in sexual assault incidents was mentioned as an issue. It was agreed that adequate staffing levels are essential for effective patient care.	Fair

For peer review only

Raveendranathan, Chandra, & Chaturvedi (2012) India	Semi-structured interviews/ Quantitative	Adult psychiatric wards	Family members of patients (n=75); 51% male; mean age 29	To assess inpatient violence from victims' perspectives, in settings where family members accompanied patients during inpatient stay and played a significant role in caregiving.	Family members were the targets of violence in 70% of the 100 incidents studied. 81% of these episodes were provoked. Provocation factors included the patient being prevented from leaving the ward and strict rules enforced by the staff. Family members suggested several preventative measures: more staff, sedation, and improved communication.	Fair
Reininghaus, et al. (2007) UK	Questionnaire/ Quantitative	A high secure psychiatric hospital	Nurses (n=636); 68% male. Mean age 39	To investigate different stress resistance resources (SRR) in the stress processes of staff at a psychiatric hospital when physical assault occurs.	Physical assault was found to be significantly related to psychological distress (P = 0.004, B = 0.181, 95% CI 0.060–0.303, R2 = 0.014). Self-esteem, self-confidence and coping were found to be effective SRRs for mediating levels of psychological distress following a physical assault.	Fair
Ryan, et al. (2004) US	Prospective/Quantitative	An inpatient psychiatric hospital	Patients (n=111); 56% male; mean age 13.9 (SD= 2.8)	This study examined the frequency and nature of violence directed at staff in a state inpatient psychiatric hospital.	The highest reported reason (68%) for assaults of staff by patients was related to verbal exchanges with staff: 'request, direction, or command potentially viewed by the patient as limit setting or as coercive'	Fair
Schwartz & Park (1999) US	Cross-sectional/ Quantitative	A psychiatric facility	Residents (n=517) 52% male; age range 25-61.	To investigate the prevalence of assaults on psychiatric residents and the violence management training they receive.	73% of residents reported being threatened, and 36% reported being physically assaulted. A third received no violence management training, and a third felt that their training was inadequate.	Fair

For peer review only

Sival, et al. (2000) Netherlands	Prospective cohort/ Quantitative	Wards of a psychogeriatric nursing home (n=2)	Residents (n=64); 72% female; mean age 80.2 (SD=7.8)	To investigate the effects of introducing a behaviour rating scale on the prevalence and management of aggressive behaviours in psychogeriatric patients.	The frequency of aggressive behaviour reported increased significantly (p<.001), while prescribing of psychotropic drugs decreased significantly (p<.05).	Fair
Sjöström, et al. (2001) Sweden	Quasi-experimental/ Quantitative	Inpatient psychiatric care	Nursing staff members (n=185) before training (n=144) after training.	To investigate whether aggressive behaviour and injury-related sick leave would be reduced by staff training and to explore predictors of violent behaviour.	No statistically significant reduction was found in the number of aggressive patients or in the number of staff members on sick leave. Directed verbal aggressiveness (OR = 1.92, P = 0.04) and violence towards things (OR = 1.82, P = 0.02). were found to be predictors of violence.	Fair
Skovdahl, Kihlgren & Kihlgren (2003) Sweden	Cross-sectional/ Quantitative	Units housing residents with dementia (n=2)	Unit 1: Caregivers (n=3); 100% female; mean age 38.5, age range 36-42; female resident (n=1); aged 92. Unit 2: Caregivers (n=6); 67% female; mean age 29.5, age range 22-43; female resident (n=1); aged 85.	To investigate, using video recordings, interactions between those with dementia and aggressive behaviour, and caregivers reporting experiencing problems dealing with this behaviour.	Interactions followed either a positive or negative spiral pattern. Caregivers who had reported problems dealing with behavioural symptoms focused on finishing the task (goals). Caregivers who were satisfied with their management of aggressiveness, focused on the processes of how goals could be achieved.	Fair

For peer review only

Speziale, et al. (2009) Canada	Pre-post intervention/ Quantitative	An inpatient facility for older adults with serious mental illness.	Staff who received the geriatric programme curriculum (n=99)	To examine the impact of training in Gentle Persuasive Approaches (GPA) on staff knowledge and competency regarding challenging patient behaviours (verbal and physical) and also on patient risk events and occupational health incidents.	Surveys found that GPA training significantly improved staff's response to challenging behaviours, and their learning of management strategies. The predicted use of body containment techniques decreased after training. Physical aggression rates declined by 50% 3 months after training (χ^2 (2, N = 564) = 27.51, p = .0001).	Fair
Spokes, et al. (2002) UK	Cross-sectional/ Qualitative	Psychiatric inpatient units (n=13); comprising adult acute admissions wards (n=10), psychiatric intensive care units (n=2) and a low-secure unit on five hospital sites	Qualified mental health nurses (n=68) and unqualified care assistants (n=40)	To investigate the views of mental health nurses about staff behaviours and other factors which may impact upon inpatient violence.	Three themes were identified by participants as being involved in the occurrence of violent incidents: clinical skills (experience, knowledge of techniques, job grade, etc.), personal characteristics (self-confidence, calmness, control, etc.) and interpersonal skills (rapport with patients, explaining things, etc.) Respondents also emphasized training needs, both in terms of new knowledge and of means of coping with actual physical violence.	Fair
Stevenson, et al. (2015) Canada	Cross-sectional/ Qualitative	Acute inpatient psychiatry settings	Registered nurses (n=12); 67% female; median age 37.5, range 27-57.	To explore nurses exposure to and experiences of patient violence, as well as the strategies they describe as influencing current practices of patient violence	For many, patient violence was considered "part of the job." Nurses often struggled with role conflict between one's duty to care and one's duty to self when providing care following a critical incident involving violence. Issues of power, control and stigma also influenced nurse participant perceptions and their responses to patient violence. The majority had difficulty	Good

For peer review only

					identifying any strategies that they perceived would have been successful to prevent the violence in their workplaces.	
Stone, et al. (2011) Australia	Cross-sectional/Mixed methods	A mental health inpatient setting	Nurses (n=107)	To determine the nature of interventions nurses used in response to aggression and whether interventions used varied with perceived causes of aggression. To identify the relationship between swearing and verbal aggression, and the impact of both on nurses.	The most frequently reported intervention for incidents of verbal aggression were talking to the patient (70% of incidents). The majority reported that exposure to swearing was highly distressing. Females appeared to be very distressed irrespective of the situation, whereas males reported being significantly more distressed by relatives of patients swearing at them.	Fair
Sukhodolsky, Cardona & Martin (2005) USA	Cross-sectional/ Quantitative	An inpatient psychiatric unit	Child inpatients (n=86); 67% male; mean age 10.8 (SD=2.4);	To evaluate the contribution of aggressive and non-compliant behaviours to restraint and seclusion use, length of stay, and psychotropic medication use in a psychiatric inpatient population.	Severity of aggressive behaviour was significantly associated with the use of seclusion and restraint, but not with the length of hospitalization or the number of psychiatric medications at discharge. By contrast, the levels of noncompliant behaviour were associated with the length of hospitalization and the number of psychiatric medications, but not with the use of seclusion and restraint.	Good
Sutton, et al. (2013) New Zealand	Prospective/Qualitative	Inpatient mental health units (n=4)	Clinical staff: (n= 40) 90% female. Service users (n = 20) 90% female	To investigate whether sensory-based approaches can develop the knowledge and practice of managing aggression in mental health settings.	Three main themes were found that described elements of sensory modulation that were perceived as helpful in the management of aggression: (i) facilitating a calm state; (ii) enhancing interpersonal connection; and (iii)	Fair

For peer review only

					supporting self-management.	
Tema, Poggenpoel & Myburgh (2011) South Africa	Cross-sectional/ Qualitative	A forensic ward	Psychiatric nurses (n=9); 78% female; age range 26-58	To explore psychiatric nurses' experiences of patients' aggressive behaviour in a forensic ward and to develop recommendations for managers to equip nurses with the skills and knowledge necessary to effectively manage patient aggression.	Four themes emerged: challenging therapeutic relationships with patients; experiences of fear resulting from threats of aggression; experiences of disempowerment related to a lack of recognition; and experiences of emotional and physical distress related to interactions with patients. Recommendations for management were to provide nurses access to: information, support, resources, opportunity and growth.	Fair
Trenoweth (2003) UK.	Cross-sectional/ Qualitative	A secure mental health environment	Mental health nurses (n=10)	To explore how mental health nurses make risk assessments in situations where violence is perceived to be likely.	It was found that nurses use information regarding their knowledge of the patient, observations of behaviour, assessing the situation as a whole, and then team working to intervene, when assessing risk in potentially violent situations.	Fair

For peer review only

Umut, et al. (2012) Turkey	Cross-sectional/ Quantitative	A research and training hospital	Inpatients (n=104); 31% female; age range 20-65	To investigate the relationship between violence and insight, clinical symptoms and treatment adherence in schizophrenia patients.	Treatment adherent patients scored significantly higher on the insight measure (Z=2.793; p<0.01). Non-treatment adherent patients scored significantly higher on the measure of aggression (Z=2.992; p<0.01).	Fair
Yip, et al. (2013) UK	Quasi-experimental/ Quantitative	A high secure hospital	High risk patients (n=59); 100% male; age range 18-65	To evaluate a programme's effectiveness in reducing violent attitudes, anger, coping processes, social problem solving, disruptive behaviour and social functioning in forensic psychiatric patients.	Medium to large treatment effects were found for the treatment group in relation to: self-reported measures of violent attitudes, social problem-solving and coping. Staff also rated behaviour on the wards as being markedly improved post-treatment.	Good
Van de Sande, et al. (2011) Netherlands	Cluster randomised controlled trial/ Quantitative	Acute psychiatric wards (n=2)	Experimental ward: baseline period (n=80); Mean age 38 (SD=13), 66% male. Intervention period (n=207); Mean age 38, 65% male. Control ward: baseline period (n=90); mean age 40 (SD=11) 60% male. Intervention period (n=251); Mean age 39, 55% male.	To investigate the effect of risk assessment on the prevalence of aggression and time in seclusion for acute psychiatric ward inpatients.	The experimental wards demonstrated a reduction in aggressive incidents (relative risk reduction, P<0.001), patients engaging in aggression (relative risk reduction, P<0.05), and time spent in seclusion (P<0.05) compared to control wards. No reductions were found in the number of seclusions or the number of patients exposed to seclusion.	Good

For peer review only

Van Wijk, Traut & Julie (2014) South Africa	Phenomenological, Cross-sectional/ Qualitative	Mental health facilities (n=2)	Inpatients (n=40); age range 21-55	To explore patients' perceptions of environmental and staff contributory factors for their aggression and violence; and to propose prevention and management strategies for this behaviour.	Two categories of contributory factors were found: environmental factors (such as living conditions and ward atmosphere), and the attitude and behaviour of staff.	Fair
Wright, et al. (2014) UK	Cross-sectional / Qualitative	High secure facilities (n=3)	Patients (n=8); 100% male. Staff (n=10); 70% male	To explore and compare staff and patient attitudes to the management of violence and aggression in a high security hospital	Seven themes emerged for both staff and patients: 'Environmental stimuli' where boredom was identified as a reason for violence and aggression occurring, 'Medication' in terms of aggression management, medication could be seen as both positive and negative, and 'Relationships' where therapeutic relationships were valued by both staff and patient and aggression was directly related to staff attitudes.	Poor
Wystanski (2000) Canada	Observational, Longitudinal/ Quantitative	A psychogeriatric inpatient ward in a psychiatric hospital.	Inpatients (n=29); 66% female; mean age 73.9 (SD=7.6);	To investigate the relationship between psychosocial stimulation and medication changes, and assaultive behaviour in a psychogeriatric ward.	Those with organic syndromes were more likely to become, and stay, aggressive. Psychosocial stimulation and changes in non-psychotropic medications influenced the duration of the aggressive behaviour.	Poor

For peer review only

Yang, et al. (2007) Taiwan	Pre-post experimental/ Quantitative	A nursing home specializing in care for patients with dementia.	Individuals living with dementia (n=20); 65% male; mean age 74.2 (SD=6.7), age range 65-86	To evaluate the effectiveness of acupressure in reducing agitation in those with dementia.	Significant differences on all outcome measures were found between control and experimental phases, indicating a positive treatment effect (Cohen–Mans-field Agitation Inventory, daily agitation records about physical attack, verbal and non-verbal attack and non-physical attack).	Fair
Zuzelo, Curran & Zeserman (2012) USA	Cross-sectional/ Qualitative	An inpatient psychiatric unit	Professional nurses and behavioural health associations (N = 19); 47% male; age range >50 (5), 40-50 (3), 30-40 (6), <30 (5).	To explore nursing staff's responses to violent incidents perpetrated by patients against caregivers.	The themes that arose regarding management of violence were: sharing information about violence, therapeutic and non-therapeutic intervention, recognizing team influences, experiencing emotions following violence, and understanding the work environment.	Good
Zwijsen, et al. (2014) Netherlands	Cluster randomized controlled trial/Quantitative	Nursing care homes with special care units for people with dementia (n=17)	Residents with dementia (n=659); 70% female; mean age 84 (SD=7.3)	To evaluate the use of a care program in decreasing challenging behaviour and the prescription of psychoactive drugs without increasing the use of restraints.	Participants in the intervention condition compared with the control condition differed significantly in the presentation of challenging behaviours. Significant effects were found on the use of antipsychotics and antidepressants. No effect on use of restraints was observed.	Good
Wright, et al. (2005) UK	Cross-sectional/ Quantitative	Acute inpatient settings	Nurses (n=771); 63% female; mean age 36.5 (SD=9.2)	To report and discuss the findings of a survey of nurse training and practice in the prevention and management of violence in acute psychiatric units	Most respondents reported a good balance of theory and practical skills training, although some aspects of theoretical training were not explored in enough depth. Reported confidence in the ability to use skills safely or effectively was fairly low. Training was generally considered to be safe and well run.	Good

For peer review only

Nijman, et al. (2005) UK	Cross-sectional/ Quantitative	Acute wards (n=12), psychiatric intensive care units (n=2), long-term rehabilitation wards (n=2), wards for the elderly mentally ill (n=3), and forensic wards at a medium security level (n=5)	Nurses (n=154); 58% female; 70% were under 40, and 36% were younger than 30	To explore psychiatric nurses' experiences with aggression	33 of 148 respondents (22%) said that they had not been able to go to work due to workplace violence at least once during the year. They had stayed at home for a total of 172 days, with an average of 5.2 sick days per nurse (range 1–23 days). Severe physical violence was the strongest predictor of sick leave (r=0.50). Frequent sexual harassment and intimidation also increased the likelihood of sick leave (r=0.38).	Fair
Quirk, Lelliott & Seale (2005) UK	Longitudinal/ Qualitative	Inpatient wards (n=3) in general hospitals (n=2) and a psychiatric hospital (n=1)	Observations made by a research sociologist.	To explore how patients manage risks arising from their interaction with other patients on the ward, such as assault and sexual harassment.	Patients seemed to manage interaction risks by: avoiding risky situations or people; de-escalating situations; seeking safety interventions by staff or increased surveillance; and seeking protection from other patients.	Fair

For peer review only

Coercive Interventions

Author, year, country	Study design	Setting	Participants	Aims/ objectives related to patient safety	Outcomes related to patient safety	Study quality
Bak & Aggernæs (2012) Europe	Questionnaire/ Quantitative	Inpatient facilities in Denmark, Sweden, Norway, Finland, Iceland, Belgium, Netherlands, United Kingdom, Ireland, France and Italy.	Governmental health authorities (European Platform of Supervisory Organisations, EPSO) and the Psychiatric Section of the European Union of Medical Specialists (Union Européenne des médicins Spécialistes, UEMS).	To compare the use of coercive measures on psychiatric inpatients of different European countries	All countries allowed the use of forced medication in some form in 2009. The UK was the only country where mechanical restraint was not allowed and Denmark was the only country where seclusion was not allowed. Coercion was perceived differently across countries. Forced medication/long period was considered worst in Norway; forced medication/short period in Belgium; mechanical restraint in Finland, Iceland and France; seclusion in the UK; holding/physical restraint in Netherlands and mechanical restraint/ambulatory in Sweden, Denmark and Iceland. In the countries using both seclusion and mechanical restraint, mechanical restraint was regarded as the most intrusive.	Poor
Bak, et al. (2014) Denmark & Norway	Questionnaire/ Quantitative	Inpatient psychiatric hospital units in Denmark (n=87) and Norway (n=96)	Clinical nurse managers (n=90).	To investigate manual restraint-preventative practices and their association with the frequency of manual restraint episodes in psychiatric hospital units in Denmark and Norway	Three factors were found to be associated with lower rates of mechanical restraint: mandatory review (exp[B] = .36, $p < .01$), patient involvement (exp[B] = .42, $p < .01$), and no crowding (exp[B] = .54, $p < .01$).	Good
Bak, et al. (2015) Denmark & Norway	Questionnaire/ Quantitative	Inpatient psychiatric hospital units in Denmark (n=87) and Norway (n=96)	Clinical nurse managers (n=90).	To examine how manual restraint- preventive factors may be associated with the	Staff education [exp(B)= 0.34, P=0.00], was associated with a lower frequency of manual	Good

				differing number of manual restraint episodes in Denmark and Norway.	restraint episodes in Denmark. In Norway, three factors were associated with a higher frequency of manual restraint episodes: cognitive milieu therapy [exp(B)=7.46, P=0.00], patient-centred care [exp(B)=5.01, P=0.00] and alarm systems [exp(B)=3.72, P=0.00]. Six factors were associated with the difference in manual restraint episodes between the two countries: identification of the patients' crisis triggers [exp(B)=- 10%], patient–staff ratio [exp(B)=-11%], staff education [exp(B)=-51%], acceptable work environment [exp(B)=-15%], substitute staff [exp(B)=-17%] and separation of acutely disturbed patients [exp(B)=13%].	
Bergk, et al. (2011) Germany	Randomised controlled trial/ Quantitative	A psychiatric hospital	Psychiatric inpatients in the hospital (n=102). Those randomly assigned to seclusion intervention (n=12). 75% male. Mean age 40.8 (SD=10.1) (range 23-61). Those randomly assigned to mechanical restraint intervention (n=14). 71% male. Mean age 38.6 (SD=12.0) (range 20-63). Those not randomly assigned to seclusion intervention (n=48). 38% male. Mean age 40.2 (SD=12.1) (range 19-66). Those not randomly assigned to mechanical restraint intervention (n=28). 46% male. Mean age 39.7	To explore opinions regarding the restrictiveness of seclusion and mechanical restraint on psychiatric patients	There was no significant difference in perceived coercion (CES scores) between the seclusion and mechanical restraint groups. No significant differences were found between the seclusion and mechanical restraint groups in the number of adverse events. The mechanical restraint group experienced lower levels of fear then the seclusion group (mechanical restraint median score =1.00, range= 1–5; seclusion median score=2.25, range=1–5, p=.049).	Fair

For peer review only

			(SD=13.4) (range 18-64).			
Bigwood & Crowe (2008) New Zealand	Descriptive phenomenological/ Qualitative	An acute adult psychiatric inpatient service	Registered comprehensive nurses and registered psychiatric nurses working in the service (n=7). 57% male.	To examine how nurses experience physical restraint of patients within an inpatient psychiatric service	The predominant theme was one of 'it's part of the job' and expected. However, participants describe being conflicted and fearful of physical restraint and prefer to use other management techniques.	Fair
Bleijlevens, et al. (2013) Netherlands	Quasi-experimental and questionnaire/ Mixed methods	Psychogeriatric nursing home wards (n=15) in Dutch nursing homes (n=6)	Nursing home staff (n=143); Nurse specialists (n=2); delegates representing nursing home associations; relatives of nursing home residents (n=38 residents).	To assess an intervention to reduce the use of belt restraints in psychogeriatric nursing homes.	In more than 50% of the cases in which a belt was removed, no alternative interventions were used. The most frequently used alternative interventions were infrared barrier alarm systems (21%) and adjustable low-height beds (12%). 96% of participants felt that the intervention met their learning needs. 76% of the 38 resident's relatives did not agree with the use of belt restraints but thought it was necessary before the intervention, and 78% were satisfied with the policy change. 79% felt involved in the decision making process regarding belt restraint removal. Three barriers to implementation of the intervention were found: availability of preferred alternative interventions, removing all physical restraint at the same time (increasing risk), and time constraints.	Fair

For peer review only

Bonner, et al. (2002) UK	Semi-structured interviews/ Qualitative	Psychiatric inpatient unit(s)	Staff in the units (n=12); patients in the units (n=6)	To explore the experiences of restraint from patient and staff perspectives in psychiatric inpatient units	Ward atmosphere and failed communication were seen as antecedents to restraint incidents. The incident itself was characterised by fear and embarrassment, with staff regarding restraint as a last resort. Debriefing and the need for understanding the incident were reported as helpful after a restraint incident. Both patients and staff spoke of the fear of re-traumatization and the difference in care from temporary and permanent members of staff.	Fair
Bonner & Wellman (2010) UK	Questionnaire/ Quantitative	Acute psychiatric inpatient units and PICUs within an NHS Mental Health Trust	Staff in the units (n=30), 57% female; patients in the units (n=30), 57% female	To explore the usefulness of post incident reviews after incidents of restraint in psychiatric inpatient units	97% of staff and 94% of patients reported that they found the post incident review process useful. The element that most participants agreed was useful was the opportunity for discussion after an incident (100% of staff; 93% of patients). 61% of staff and 20% of patients believed the incident could have been predicted. 67% of staff and 50% of patients believed the incident had been well managed.	Fair

Boumans, et al. (2012) Netherlands	Questionnaire/Quantitative	Long stay wards (n=2); a forensic psychiatric ward; and a crisis intervention ward within a psychiatric hospital.	Staff in the units (n=60). 57% male.	To explore the importance of several factors in nurse decision making on seclusion and to explore the effect of reflexivity on the decision to seclude patients.	Approachability was found to be the patient variable with the greatest impact on likelihood of seclusion: if the patient was 'hardly approachable' the mean tendency to seclude was 0.52 higher than when they were deemed 'approachable' (95% CI 0.44, 0.60). The more reflexive a team was, the less likely they were to seclude (Pearson correlation coefficient - 0.97, P = 0.017).	Fair
Bowers, et al. (2012) UK	Randomised controlled trial/Quantitative	Acute psychiatric wards (n=136) in 67 hospitals within 26 National Health Service (NHS) Trusts	All staff and patients within the units	To explore the relationship of manual restraint and show of force to conflict behaviours, containment methods, environment, routines and staff variables in acute psychiatric wards	Manual restraint was used less (0.20 incidents per day) than show of force (0.28 incidents per day). Both were associated with aggressive behaviours and the enforcement of treatment and detention. Staff provision was associated with the use of these coercive interventions. Clearer ward structure and routine was associated with decreased use of manual restraint and show of force.	Fair
Bowers, et al. (2007) UK, Netherlands, Finland, and Australia	Questionnaire/ Quantitative	Psychiatric inpatient settings	Psychiatric professionals (n=844). UK (n=114; modal age under 30; 61% female). Netherlands (n=146; modal age under 30; 65% female). Finland (n=304; modal age 30-39; 44% female). Australia (n=280; modal age 40-49; 67% female).	To explore the attitudes towards containment measures of psychiatric professionals in four countries	Staff in Finland expressed the highest level of approval of containment, and staff in the UK expressed the least. Preferences for different containment measures were influenced by whether they considered it: safe for the patients; preventative of injury to others; and rapidly calming.	Fair

For peer review only

Braham, Heasley & Akiens (2013) UK	Questionnaire and semi-structured interviews/ Mixed methods	One admitting ward from four clinical services within a high secure hospital (male mental health, personality disorder, learning disability and the women's service)	Patients (n=31; 72% male; mean age 37; range 22-56) and staff (n=84) within the wards.	To evaluate the impact of night confinement in a high secure hospital	Night confinement was not shown to have any adverse effects. Before the pilot, the majority of staff (70%) felt that it could have adverse effects on patients, and the majority of patients (74%) felt that the effects would be minimal. After the pilot, 46% of staff felt that the impact had been minimal, and 58% of patients felt that the impact had been positive.	Fair
Chien, Chan & Kam (2005) China	Semi-structured interviews and clinical records/ Qualitative	Acute admission wards (n=2) within a psychiatric hospital	Psychiatric inpatients (n=30). Mean age 31	To explore the perspectives of psychiatric inpatients concerning the use of restraint.	The majority of participants reported positive feelings towards staff who had shown concern and had offered help during restraint. Negative effects of restraint were related to patient needs not being met. In particular: concern, empathy, active listening, and information about restraint.	Fair
Ching, et al. (2010) Australia	Questionnaires/Quantitative	Units within a secure forensic psychiatric hospital (n=5): male acute units (n=2); female acute unit (n=1); mixed-sex sub-acute unit (n=1); rehabilitation unit (n=1)	Phase 1: Staff (n=60; 50% female; modal age group 18-30) and patients (n=13; 31% female; modal age group 18-30). Phase 2: Staff (n=61; 48% female; modal age group 31-40) and patients (n=7; 29% female; modal age group 18-30).	To evaluate a group of interventions for reducing the use of seclusion in a forensic psychiatric hospital.	There was no change in levels of staff confidence to manage aggression from pre-post intervention. There was a significant reduction in reported absconding risk from pre-post intervention ($F(1, 92) = 4.2$, $p=0.04$). Post-intervention, staff were more likely to report that seclusion was used therapeutically ($t(112)=-2.41$, $p=0.02$).	Good

For peer review only

Chu, et al. (2015) UK	Pre-post questionnaire/ Quantitative	Wards (n=6) within a high-secure forensic mental hospital	Patients (n=30). Mean age 39 (range 23-60). Ward staff: Night confinement respondents (n=144); EssenCES respondents (n=119)	To explore the views of staff and patients concerning a new night-time confinement policy within a high-secure forensic hospital	No significant differences from pre-post night-time confinement were found for patient sleep quality, behaviour, ward atmosphere, or therapy engagement. Patient attitudes towards night-time confinement generally became more positive from pre-post implementation. Staff attitudes remained largely negative, however, proportions decreased post-implementation.	Fair
Cormac, Russell & Ferriter (2005) UK	Questionnaire/ Quantitative	High secure hospitals and medium secure units (n=39)	Senior managers (n=39)	To explore the use of seclusion and seclusion policies and procedures in medium and high secure units	69% of the units reported using seclusion in the past year. All policies followed Code of Practice guidelines. Seclusion environment requirements varied. Additional requirements found in some policy documents were thought to potentially enhance practice: privacy and dignity, observation, fittings and fixtures, cleanliness, communication, physical health, ending of seclusion and contact with significant others.	Poor
Espinosa, et al. (2015) USA	Pre-post evaluation/Mixed methods	Inpatient psychiatric units (n=15) within a large multi-site medical centre	Staff and patients	To evaluate interventions to improve milieu on psychiatric inpatient units	Restraint and seclusion rates reduced dramatically, but this meant that staff confidence in their skills regarding these interventions decreased. Violent incidents also reduced in frequency.	Poor
Exworthy, et al. (2001) UK	Questionnaire/ Quantitative	Maximum security hospitals (n=3) and medium security units	Psychiatrists and doctors (n=117)	To explore the views of doctors and psychiatrists concerning the practice of seclusion in secure units	56.4% of respondents agreed that seclusion is a form of treatment. 82% agreed that it was not a form of punishment. 60% felt that it helped	Poor

For peer review only

					avoid the use of medication and 86% support its use when a patient is threatening physical violence to others. 70% were against its use for self-harm behaviours. 45% felt that seclusion should be defined in law.	
Ezeobele, et al. (2014) USA	Semi-structured interviews/ Qualitative	A psychiatric acute care hospital	Patients (n=20). 60% male. Mean age 28 (range 19-53)	To explore the psychiatric patients' lived seclusion experience.	60% of participants felt that seclusion was a penalizing and negative experience. Themes regarding the experience of seclusion were: feeling alone, staff exerting power/control, feeling resentful of staff, and having time for meditation.	Good
Faschingbauer, Peden-McAlpine & Tempel (2013) USA	Semi-structured interviews/ Qualitative	Psychiatric inpatient facilities	Psychiatric inpatients (n=12). 50% female. Mean age 33 (range 18-50)	To explore the psychiatric patients' lived seclusion experience.	Themes described by patients regarding the experience of seclusion were: hope for respect and communication, emotional response to seclusion (heightened anger/anxiety), and insight into behaviour and positive coping skills (need debriefing with staff).	Fair
Georgieva, Mulder & Wierdsma (2012) Netherlands	Questionnaire/ Quantitative	Acute psychiatric hospital	Psychiatric inpatients (n=161); 54% female.	To examine patient's experience and preferences regarding coercive interventions and associated factors	Previous experience of seclusion predicted preference for seclusion in emergency situations. However, most inpatients preferred medication in an emergency situation.	Fair
Georgieva, Mulder & Noorthoorn (2013) Netherlands	Experimental/ Quantitative	Acute psychiatric hospital	Psychiatric inpatients (n=520). Involuntary medication, (n=236); 48% male; mean age 40 and seclusion (n=284); 53% male; mean age 40	To examine the number and duration of seclusion and coercive measures relating to inpatients who are allocated to involuntary medication intervention compared with seclusion (TAU)	Relative risk (RR) of seclusion was lower for patients who received involuntary medication than TAU. Seclusion episode duration and the number of coercive incidents were not significantly different between the two groups.	Fair

For peer review only

Hatta et al. (2007) Japan	Cohort/Quantitative	PICU	Psychiatric inpatients; restrained patients (n=106) and non-restrained patients (n=528)	To investigate the effects of physical restraint on the development of drug induced liver injury	Prevalence of drug induced liver injury was significantly higher for those who were restrained (8.5%) than the non-restrained group (1.9%; odds ratio 4.81). Rates of those receiving antipsychotics were higher in the restrained group.	Fair
Haugom & Granerud (2016) Norway	Descriptive/ Qualitative	Psychiatric wards (n=57)	Staff (n=149)	To investigate how psychiatric patients and staff describe and assess the practice of shielding	Shielding is an ambiguous practice. Shielding as a form of control was seen as more important than as a form of treatment.	Fair
Haw et al. (2011) UK	Semi-structured interviews and case note review/ Mixed methods	Low and medium secure wards within a psychiatric hospital	Patients (n=57); 48% male; median age 29 (range 19-52)	To explore patients' experiences of, and preferences for, physical restraint, forced medication and seclusion	Coercive treatments were generally perceived as negative experiences; however, 16% of participants reported that the last episode of seclusion or restraint had been positive. Most patients preferred medication to seclusion. Patients felt that advance statements and views concerning restrictive practice should be a part of their care plans.	Fair
Holmes, Kennedy & Perron (2004) Canada	Phenomenological semi-structured interview study/ Qualitative	Specialised psychiatric unit	Psychiatric inpatients (n=6)	To explore the experiences of seclusion of patients with severe and persistent psychiatric disorder	Themes emerged: emotional experience of seclusion (feelings of rejection, isolation, etc.); patients' perceptions of seclusion (as punitive and a way of exerting social control), and coping mechanisms. The lack of staff contact during seclusion seemed to have more of a negative impact on patients than the seclusion itself.	Fair

For peer review only

Holmes, Murray & Knack (2015) Canada	Phenomenological semi-structured interview study/ Qualitative	Forensic psychiatric inpatient hospital	Participants (n=26): Forensic psychiatric inpatients (n=13) and forensic psychiatric nurses (n=13)	To study the lived experience of the secluded room	Three main themes from inpatients emerged: experience of seclusion, assessing quality of care and space of confinement. Themes from staff interviews were: resorting to seclusion, observing and assessing patients and experiencing seclusion. The therapeutic relationship is important to both staff and patients.	Fair
Hottinen et al. (2012) Finland	Descriptive/ Quantitative	Adolescent closed ward of a general hospital	Clinical staff (n=128); 74% female; 75% nurses	To investigate staff attitudes towards containment measures used in adolescent psychiatric wards	Preferred containment methods were medication, transfer to specialist locked wards and mechanical restraint. The net bed was the least preferred containment method.	Fair
Huizing et al. (2006) Netherlands	Experimental/ Quantitative	Psychogeriatric nursing home wards (n=5)	Residents with dementia (n=167); baseline (n=145); experimental (n=83); mean age 82; 78% female and post-intervention (n=144); experimental (n=86); mean age 82; 73% female	To investigate the effects of an educational intervention on the use of physical restraints in psychogeriatric nursing home wards	Despite education on the use of restraint, the use of restraint did not significantly decrease in the experimental group. Residents in the control group experienced significantly more restraint than the educational group.	Fair
Jaeger et al. (2014) Switzerland	Cross-sectional/ Quantitative	Acute psychiatric hospital	Mental health professionals (n=39); 59% female	To evaluate how staff recognise different levels of coercion and treatment pressures	Results showed low levels of coercion were adequately recognised, whereas high levels of coercion were underestimated.	Fair
Keski-Valkama et al. (2007) Finland	Descriptive/ Quantitative	Psychiatric hospitals	Patients restrained in 1990 (n=94), 1991 (n=107), 1994 (n=71), 1998 (n=59), 2004 (n=59). Patients secluded in 1990 (n=75), 1991 (n=43), 1994 (n=86), 1998 (n=40), 2004 (n=36)	To examine the use of restraint and seclusion over time	The number of seclusion and restraint incidents decreased over the five time periods; however the duration of seclusion significantly increased over time (v2 (4) = 36.111, $p < 0.001$).	Fair

For peer review only

Kirkevold & Engedal (2004) Norway	Descriptive/ Quantitative	Regular units (RU) for patients with dementia (n=142). Special care units (SCU) for inpatients with dementia (n=80)	Staff interview details not recorded. Interviews based on inpatients (n=1501): RU (n=1057); 66% female; mean age 84.4 [6.9]; SCU (n=444); 75% female; mean age 83.4 [6.1]	To identify the prevalence of patient restraint in nursing homes	Significantly more restraint occurred in SCUs than in RUs (45.0% and 36.7% respectively). The most common restraint interventions were mechanical restraint and use of force/pressure in daily activities.	Fair
Knowles, Hearne & Smith (2015) UK	Semi-structured interview/ Qualitative	Medium secure unit	Inpatients (n=8); mean age 39; 13% female	To explore the impact of employing physical restraint on maintaining therapeutic relationships between staff and patients	Five themes were found: Restraint reinforces the inequality of power in the staff-patient relationship; abusive, degrading and traumatic experiences; whether restraint justification influences whether it is an accepted intervention; negative attitudes and motives of some staff; and coping with having no power during and after restraint.	Fair
Kontio et al. (2012) Finland	Interviews/ Qualitative	Psychiatric inpatient hospital	Inpatients (n=30); 37% female; mean age 41 (range 20-64 years)	To explore patient perspectives on the use of, and future directions of restraint and seclusion	In general patients had negative experiences during seclusion/restraint and did not have enough information before it happened regarding why the intervention occurred. Future improvement ideas included recent information about their care plan and treatment, and reasons why they were restrained. Alternatives to restraint and seclusion included empathetic patient–staff interaction, meaningful activities, therapeutic community, and biological treatments.	Fair

For peer review only

Kontio et al. (2011) Finland	Experimental/ Quantitative	Psychiatric inpatient wards in general hospital (n=12)	Nurses (n=158); intervention group (n=95); 55% female; mean age 43 [9.0] (range 25-60) and control group (n=63); 52% female; mean age 45 [10] (range 24-64)	To examine the effect of an online learning course on nurse competence in restraint and seclusion interventions	Knowledge of physical restraint improved at three month follow-up whereas knowledge of seclusion did not change. Self-efficacy increased in the intervention group.	Fair
Kontio et al. (2009) Finland	Focus group/ Qualitative	Psychiatric hospital (n=2)	Staff (n=27); 52% female; mean age 44 years (range 26-59)	To examine staff perspectives on current educational needs in relation to implementing seclusion and restraint, and their future needs	Difference found on guidance regarding seclusion and restraint: some staff wanted structured guidelines and some wanted to rely on knowledge/experience. All seemed to want more education regarding the ethical, clinical and legal aspects of restraint and seclusion interventions. This was in addition to a desire for staff support to ensure the success of the intervention.	Fair
Kontio et al. (2010) Finland	Focus group/ Qualitative	Psychiatric hospital (n=2)	Staff (n=27); 52% female; mean age 44 years (range 26-59)	To explore what happens when an aggressive incident occurs in a psychiatric ward and what alternatives to restraint and seclusion are possible	Staff felt that the patient perspective was not considered enough. Staff also proposed numerous alternatives to seclusion and restraint including being present and having a conversation with the patient. Cooperation and communication via multi-professional agreements that involve the patient perspective were deemed useful.	Fair

For peer review only

Kuosmanen et al. (2015) Finland	Experimental/ Qualitative	Acute psychiatric hospital	Staff (n=2)	To explore the experience of being secluded and to understand and evaluate the impact of seclusion from a staff perspective.	Overall, staff perceived their seclusion experience as negative and inhumane. The staff felt anxiety and frustration at being locked up. Future practical suggestions for change included updating seclusion practice guidance and re-designing seclusion facilities. Ideas included introducing a clock and normal height bed and chair.	Poor
Larsen & Terkelsen (2014) Norway	Observational/ Qualitative	Locked psychiatric ward	Inpatients (n=12). Staff (n=22)	To explore coercion experience	There were four main themes: corrections and house rules, coercion as a necessary intervention, material surroundings as being of great importance, and being treated as a human being. Staff and patients differed in their experience. Patients felt inferior, and staff sometimes felt guilty when implementing the interventions.	Fair
Larue et al. (2013) Canada	Structured interview/ Mixed methods	Psychiatric hospital	Inpatients (n=50); 38% female	To identify and describe perceptions of the seclusion and restraint protocol in a psychiatric hospital	Just over half of inpatients indicated they were not offered an alternative to restraint (n=28). Eighteen patients felt seclusion fulfilled their need for calm, sleep and safety. Almost all said staff did not follow up with them after their seclusion/restraint experience and deemed this as an essential need in the future.	Fair

For peer review only

Seo, Kim & Rhee (2013) South Korea	Descriptive/ Quantitative	Psychiatric hospital	Inpatients (n=248); 38% female; mean age 38.6 [11.4]	To examine whether coercive interventions can be justified by the assumption of incompetence, the assumption of dangerousness and the assumption of impairment	Legal status, perceived coercion and experienced coercive measures were justified as measures of coercion under the assumption of incompetence.	Poor
Lee et al. (2003) UK	Descriptive/ Mixed methods	Secure inpatient mental health unit and PICU	Nurses (n=269)	To examine staff views of their last experience of employing physical restraint	The majority of nurses reported positive results after the coercive incident. Issue raised regarding the after effects of the incident were concern and ambivalence. Negative aspects of interventions included injury, management and clinical issues. Future improvements reported included less crowed environment, improved staff training in de-escalation and adopting positive care philosophy around relationships with patients	Poor
Lee et al. (2001) UK	Cross-sectional/ Quantitative	Regional secure inpatient mental health units and PICUs (n=63)	Nurses (n=338); 53% female; mean age 36.2 [8.9] (range 19-63)	To investigate training in physical restraint in order to compare course content and length, and injuries in training	Course content varied despite a core curriculum in place. Length of course also differed (range 0.5-21 days) but two thirds attended a 5 day course. Sometimes refresher courses did not happen as expected. About a third of respondents reported being hurt in training.	Poor
Long et al. (2015) UK	Pre-post intervention design/ Quantitative	Medium secure inpatient unit	Patients (n=38). Experimental group (n=19), and matched control group (n=19); mean age 31.1 (range 19-49); 100% female.	To assess the effectiveness of interventions designed to minimise the use of seclusion in response to risk behaviours.	There was a significant decrease in the number of seclusion incidents and risky behaviour post-intervention change, as well as more positive staff ratings of patient behaviour,	Fair

For peer review only

					improved treatment engagement and reduced patient time spent in medium security. Staff perceived training and use of de-escalation techniques as the most effective whereas patients felt individual engagement and initiatives to reduce bullying, harassment and discrimination was the most effective.	
Looi, Engstrom & Savenstedt (2015) Sweden	Self-report/Qualitative	Psychiatric inpatient unit	Inpatients (n=19)	To explore the perceptions of people who self-harm regarding alternatives to coercive measures and how this relates to lived experiences of psychiatric care	Content analysis revealed three main themes: understanding rather than neglect, mutual relation rather than distrust, and professionalism rather than counterproductive care.	Fair
Lovell, Smith & Johnson (2015) UK	Semi-structured interviews/ Qualitative	Secure learning disability service	Nurses (n=20)	To explore the views of learning disability nurses regarding physical intervention incidents, and contributory factors of injuries sustained	The overarching theme, knowledge and understanding, placed three other themes in context: physical intervention techniques employed, staff interpretation of the safety incident and the impact on staff. All staff felt it was important to know the patient and use an individualised approach when an incident occurred.	Fair
Mackay, Paterson & Cassells (2005) UK	Unstructured interviews/ Qualitative	Sector Acute Psychiatric Admission wards (n=3) and PICU (n=1)	Nurses (n=6)	To explore the perceptions of observing for patients with perceived risk of violence/aggression and important factors in day to day practice	Procedure, skills and role were identified as three higher level categories. An additional six categories made up "role": intervening; maintaining patient safety and that of others; prevention de-escalation; and managing aggressive and violent incidents; assessment;	Fair

For peer review only

					communication; and therapy. Staff skills were built within this and deemed interconnected.	
Mason & Whitehead (2001) UK	Structured interview/ Mixed methods	Forensic psychiatric inpatient hospital	Female inpatients	To explore the problems associated with secluding female inpatients	There were 34 seclusion incidents with the longest duration identified as 23 days, 8 hours and 45 minutes. The most common reason for seclusion was actual violence to staff (928 hours), threat of violence (238 hours) and threat of property damage (129 hours). Staff rationale for stripping patients while in seclusion was for patients' safety. PRN medication was usually given before a seclusion.	Fair
Muir-Cochrane et al. (2015) Australia	Semi-structured interviews/ Qualitative	Old age psychiatric unit (n=3)	Mental health nurses (n=39)	To explore nurses experiences of restraint and seclusion and resistance to getting rid of these interventions	"Lack of accessible alternatives to restraint and seclusion" was the overarching theme. Interrelated themes covered environmental factors contributing to restraint and seclusion interventions; the consequences of poor staff-patient relationships; and the influence of ward environment on restraint and seclusion intervention implementation (an unfavourable physical environment impacts upon the aggression,	Fair

For peer review only

					restraint and seclusion tactics used).	
Papadopoulos, et al. (2012) UK	Cross-sectional/ Qualitative	Acute inpatient psychiatric wards (n=16)	Ward managers and consultant psychiatrist (n=120)	To investigate what influences the likelihood of patient conflict (verbal abuse, violence, and rule breaking) and containment (seclusion, manual restraint, and enforced medication)	Negative staff morale increased the likelihood of conflict and containment, whereas positive staff practice (environment improvement, increased staff activity, proactive ward manager) decreased the likelihood of such events occurring.	Good
Pellfolk, et al. (2010) Sweden	Cluster-randomized controlled trial/ Quantitative	Group dwelling units for people with dementia (n=40)	Control group at baseline: Residents (n=162); mean age 83.4 and staff (n=162); mean age 43.2. Intervention group at baseline: Residents (n=191); mean age 80.5 and staff (n=184); mean age 43.5	To evaluate a restraint minimization education program.	In the intervention group, staff had more knowledge about restraint, but attitudes toward restraint use were not significantly different from the control group. The likelihood of being restrained was lower in the intervention group (OR) =0.21, 95% (CI) =0.08–0.57, *P*=.002, n=281).	Good

For peer review only

Perkins, et al. (2012) UK	Semi-structured interviews and focus groups/ Qualitative	An acute adult mental health setting	Nursing staff (n=30)	To explore the attitudes of staff towards restraint.	Factors thought to influence the decision to restrain were: contextual demands; lack of alternatives; the escalatory effects of restraint; and perceptions of risk. Nurses described restraint as a “necessary evil” due to the unpredictability of mental illness and their work environment.	Fair
Raboch, et al. (2010) Europe and Israel	Questionnaire/ Quantitative	Psychiatric inpatient facilities	Patients (n=2,030); age range 18-65.	To compare the use of coercive measures in psychiatric inpatient facilities in different European countries.	1,462 coercive measures were used with 770 patients (38%). 21%-59% of patients received coercive measures, with the most common reason being patient aggression against others. In eight of the ten countries, the most common measure was forced medication, and in two of the countries it was mechanical restraint. Seclusion was rare (only 6 countries reported it).	Fair
Ryan & Bowers (2005)	Cross-sectional observational/ Qualitative	A PICU in an inner city hospital	Staff nurses and patients	To explore the implementation of coercive manoeuvres in a PICU.	Within the PICU, nurses practised several coercive strategies, which enabled them to manage low-level conflict situations. More serious conflict necessitated more severe containment methods. For example, ‘time out’ was used regularly (confinement in the patient’s own	Fair

For peer review only

					bedroom or in an open seclusion room), as well as seclusion.	
Ryan & Happell (2009) Australia	Participatory action research/Qualitative	Adult acute mental health inpatient units with high-dependency unit suites and seclusion facilities (n=2)	Mental health nurses (MHN) (n= 31) and consumer consultants (CC) (n=4)	To describe current practice and debriefing needs of staff, in order to gauge the need for a training program to facilitate post-seclusion debriefing.	Themes emerged from the data: 1) Debriefing, support, and flexibility- MHNs stated that debriefing consumers after seclusion is important. 2) Inherently unethical? Some CCs had concerns around being involved in a debriefing program, for example: "Seclusion is not an evidence-based intervention, it is a breach of human rights" 3) Support us, don't preach to us- debriefing should be about providing psychological support. Consumer choice is also important.	Good
Schreiner, Crafton & Sevin (2004) US.	Pre-post intervention study/ Mixed methods	An adolescent inpatient unit	Inpatients (n=23); 56% male; age range 13-17.	To describe the effects of a staff re-education campaign in reducing the use of physical restraints treating adolescents with developmental delays and severe psychiatric disturbances	During the assessment phase, a monthly average of 18.67 seclusion events were recorded. During the intervention phase, monthly seclusion events declined to 12.14, a decrease of 35%.	Fair
Sequeira & Halstead (2001) UK	Interviews/ Mixed methods	Secure wards in a psychiatric hospital (n=5)	Patients (n=116); 22% female; age range 13-54.	To investigate the use of coercive measures with patients who have developmental disabilities and to explore the experience of these measures.	Women had a significantly higher probability of being given rapid tranquilization following violent incident, seclusion was more likely to be used with men. Interviews with women demonstrated a commonly held understanding of interventions as punishment and expressions of intense anger and anxiety.	Fair

For peer review only

Steinert, et al. (2007) Germany	Prospective/ Quantitative	Psychiatric hospitals (n=10)	Treated cases (n=36,690)	To investigate the prevalence of coercive measures in different psychiatric hospitals	9.5% of 36,690 cases were exposed to coercive measures. On average, these measures were applied 5.4 times per case and lasted 9.7 hours each. The use of seclusion and restraint guidelines was associated with a lower incidence of coercive measures.	Fair
Tateno, et al. (2009) Japan	Cross-sectional vignette study/ Quantitative	Psychiatric inpatient facilities	Young psychiatrists (n=183)	To explore Japanese psychiatrists' attitudes about emergency coercive interventions for patients with acute psychosis.	Most participants agreed that the vignette case should be admitted to hospital and secluded. Regarding the likelihood of prescribing restraint, results varied; psychiatrists at general hospitals were more likely to prescribe restraint than those working at university or psychiatric hospitals.	Fair
Tompsett, Domoff & Boxer (2011) USA	Prospective/ Quantitative	A secure psychiatric hospital	Adolescents (n=149); 59% male; mean age 14 (SD=3)	To investigate predictors of aggression in adolescent inpatients.	Unique predictors of restraint involvement were found: history of aggression against adults and history of previous psychiatric hospitalizations. No predictors were significant for the extent of restraint involvement.	Good
Whitecross, Seeary & Lee (2013) Australia	Interventional/ Quantitative	Inpatient wards	Patients in the TAU group (n=14); 71% male; mean age 35.8 (SD=9.9). Intervention group (n=17); 76% male; mean age 37.8 (SD=10.1)	To identify the impacts of seclusion on patients and evaluate the effectiveness of a post-seclusion counselling intervention in reducing time spent in seclusion and trauma experienced.	There was no difference in trauma experience between the TAU group and post-seclusion counselling intervention group.	Fair

For peer review only - http://bmjopen.bmj.com/site/about/guidelines.xhtml

Whittington, et al. (2009) UK	Cross-sectional/ Qualitative	Acute care mental health services	Service users (n=1,361); 67% female. Staff (n=1,226); 48% female	To ascertain the degree of approval amongst service users and staff for commonly used coercive measures in acute mental health care	Both service users and staff disapproved strongly of mechanical restraint and expressed a relative preference for compulsory intramuscular medication and seclusion. Male staff, older service users and staff who had implemented coercion reported greater approval of coercive measures.	Fair
Sequeira & Halstead (2004) UK	Cross-sectional / Qualitative	Secure mental health service	Nursing staff (n=17); 53% male; age range 18-50. Restrained patients (n=14) and patients who observed the same event (n=5)	To examine the experiences of physical restraint processes reported by nursing staff	A range of emotional responses by nursing staff included distress, anxiety and crying. These were sometimes corroborated by patient descriptions. Staff used laughter to reduce stress following an incident, and many reported feeling little emotion during incidents, instead taking any distress home.	Fair
Smith & Jones (2014) UK	Descriptive and semi-structured interviews/ Mixed methods	A PICU within an inner city heath service	Staff members (n=10); 90% male. Patients (n=7); 100% male	To explore the effects of a sensory room on a PICU on seclusion rates and staff and patients' experiences	Interviews revealed a perception by staff that seclusion rates had reduced, despite no significant reduction occurring. Both staff and patients expressed that the sensory room was positive and therapeutic, improving staff-patient communication and patients' experience of the PICU.	Fair

For peer review only

Safety culture

Author, year, country	Study design	Setting	Participants	Aims/ objectives related to patient safety	Outcomes related to patient safety	Study quality
Bowers, Gournay & Duffy (2000) UK	Questionnaire/ Quantitative	A random stratified sample of NHS Trusts providing psychiatric inpatient services in England and Wales (n=27).	Directors of Nursing for these Trusts	To explore the observation policies and usage in psychiatric inpatient services	12% of the services had no written observation policy, and 38% had no clinical recording system. Only two policies studied used the same terminology for the different levels of intensity of constant observation (CO). There was variation across Trusts in who was qualified to undertake CO and whether agency staff were employed to do this. Gender of staff was a consideration for most Trusts when deciding who to allocate to CO. Most policies stated that patients should be given a rationale for CO. The most agreed upon reason for using CO was to reduce harm/suicide.	Fair
Bowers, et al. (2006) UK	Questionnaire/ Qualitative	14 acute psychiatric wards and 3 psychiatric intensive care units across 3 hospital sites, within 1 NHS Trust	Staff from these wards (n=56). Ward Managers (n = 16), F Grade mental health nurses (n = 17), Occupational Therapists (n = 14) and Consultant Psychiatrists (n = 9).	To explore the impact of serious incidents on inpatient psychiatric wards	Staff reported feelings of shock, depression, demoralisation, loss, and grief after incidents, with periods of rumination, guilt and anxiety. Following incidents, levels of containment increased, along with the focus on risk assessment. Staff reported the following as making it difficult to emotionally process the incidents: the pace of ward life, a lack of external support, and management investigations. Patient responses to the	Fair

For peer review only

					incidents were rarely acknowledged.	
Brennan, Flood & Bowers (2006) UK	Observation and action research/ Qualitative	Generic acute admission wards (n=2)	City Nurses within the wards (n=2). All staff and patients within the wards	To explore change implementation issues in psychiatric acute admission wards	Barriers to change were: limited staffing resources, the physical environment, insufficient beds, unclear hierarchical structure and multidisciplinary issues, over demanding ward managers, and anxiety about serious incidents.	Fair
Cullen, Nath & Marcus (2010) USA	Semi-structured interviews/ Qualitative	Inpatient psychiatric units within urban teaching hospitals (n=2)	Staff (n=17). 53% female	To explore the typology of errors and precipitating factors in inpatient psychiatry	Preventive errors (such as falls, suicide) were mentioned the most (38% of errors mentioned). Provider factors were most commonly attributed to the precipitation of errors (74% of factors mentioned). Most of the broad typologies and precipitating factors echo those found in general medicine and surgery, whilst the specifics are unique to inpatient psychiatry.	Fair
Delaney & Johnson (2006) USA	Observation and semi-structured interviews/ Qualitative	Inpatient psychiatric units (n=2)	Staff (n=16; age range 22-50+) and patients (n=12; 50% female; age range 22-56)	To explore de-escalation processes and the skills of staff in inpatient psychiatric units to create a safe environment	Staff behaviours can create a safe milieu and positive culture. Important skills for maintaining safety were: awareness, attending, caring, and connecting.	Fair

For peer review only

Gabrielsson, et al. (2014) Sweden	Focus groups/Qualitative	The inpatient ward of a psychiatric clinic	Staff (n=26). 54% female.	To explore staff perceptions of inter-professional collaboration relating to challenging situations in psychiatric inpatient care.	Physicians were described as distant decision-makers, ward managers as suspicious supervisors, psychiatric nurses as mediating moderators, and nursing assistants as informed performers. Expectations of staff during challenging situations were: to talk to each other, control the situation, know the patient and set the stage. The common thread throughout all discussion was recognising knowledge of the patient as decision-making power in challenging situations.	Fair
Gifford & Anderson (2010) Canada	Nominal group consensus technique/ Qualitative	Inpatient psychiatric mental health care facility	Healthcare nurses with inpatient nursing experience (n=16); 75% female; mean age 49	To identify staff attitudes to reporting assault in an inpatient psychiatric setting	Safety culture was the predominant factor in deciding whether to report an incident. The design of the reporting system, and the perceived effect on patients were also deemed important when making a reporting decision.	Fair
Happell & Koehn (2011) Australia	Descriptive/ Quantitative	Acute inpatient unit	Psychiatric nurses (n=123); 51% female; mean age 41 (range 20-62)	To investigate associations between burnout, job satisfaction and therapeutic optimism, and seclusion attitudes	A significant negative relationship was identified between optimism and seclusion attitudes. Staff attitudes influenced the likelihood of secluding patients. Staff with higher levels of therapeutic optimism and lower levels of emotional exhaustion are less likely to support the use of seclusion.	Fair

For peer review only

Johnson & Delaney (2006) USA	Descriptive/ Qualitative	Psychiatric inpatient unit	Staff (n=16); 56% female and patients (n=12); 50% female; mean age 33	To describe the context and conditions under which specific interventions were used to assist patients to regain control	The overarching theme was dimensions of "keeping the unit safe" that was split and interconnected between ideology, people, space, and time.	Fair
Jones et al. (2010) UK	Semi-structured interviews/ Qualitative	Acute psychiatric hospital	Inpatients (n=60); 40% female; mean age 43 (19-81)	To explore the experiences of service users on acute inpatient psychiatric wards regarding safety and security	The majority of patients felt safe and supported by staff and other service users during their stay in hospital. However, safety incidents such as aggression, drug misuse or bullying negatively influenced this perspective, resulting in patients feeling unsafe.	Fair
Jonker et al. (2008) Netherlands	Cross-sectional/ Quantitative	Wards of a mental health institution (n=6)	Nurses (n=85); 68% female	To explore nurses' perceptions of the process of managing aggression and the determinants of the use of coercive interventions	Nurses felt in control of most aggression incidents and felt that they had social support from colleagues. Despite the high prevalence of aggressive incidents, staff did not feel that it was a large problem.	Fair
Kanerva et al. (2015) Finland	Semi-structured interviews/ Qualitative	Psychiatric inpatient unit	Nurses (n=26); mean age 39 (range 23-60)	To describe the communication components that support patient safety	Communication that supports patient safety in psychiatric inpatient care was the main theme and overarched three sub-themes of fluent information transfer, open communication culture and being active in communication.	Fair
Kanerva, Lammintakanen & Kivinen (2016). Finland	Semi-structured/ Qualitative	Psychiatric hospital (n=2)	Nurses (n=26); 61.5% female; mean age 39 years (23-60)	To explore nursing staff's views of patient safety in inpatient care	Two themes were: experiences of safety (issues related to feelings about patient safety), and implementation of safe care (practical issues related to patient safety). Medication safety was deemed particularly important, whereas	Fair

For peer review only

					seclusion, restraint and suicide were barely mentioned. More emphasis was given to the skills staff felt they need to give safe care.	
Koukia et al. (2010) Greece	Questionnaire/Quantitative	Inpatient wards (n=14) in acute psychiatric hospitals (n=3)	Mental health nurses (n=164); 51% female; mean age 36.5 [3.5]	To investigate safety measures taken by mental health nurses and identify security policies in acute mental health wards	Standardised protocols did not exist across the hospitals. There was a lack of safety measures across the wards. Banned substances and search procedures varied greatly.	Fair
Kuosmanen et al. (2013) Finland	Cross-sectional/Quantitative	Forensic hospital (n=2)	Staff (n=283); 51% female	To evaluate the current patient safety culture and identify potential improvements	Nearly two-thirds rated the patient safety level as excellent or very good. Teamwork within psychiatric units received the highest score (72% positive), and non-punitive response to errors received the lowest (26% positive).	
Langan & McDonald (2008) Ireland	Descriptive/ Quantitative	Acute psychiatric inpatient unit	Staff (n=27); mean age 35.7 [9.9]. Patients (n=35); 43% female; mean age 38.7 [10.0]	To identify the prevalence of placing patients in night attire, its clinical and demographic associations, and attitudes towards it	Patients were unhappy and uncomfortable in the night attire and suggested they should be able to wear what they want. Staff had a different view and felt only voluntary patients should be allowed to have their own clothes. Staff felt patients wearing night-attire helped with security and reducing absconding and self-harm.	Fair

For peer review only

Maguire, Daffern & Martin (2014) Australia	Semi-structured interviews/ Qualitative	Forensic hospital	Patients (n=12) Nurses (n=12); 58% female	To explore patient and staff perspectives of limit setting in a forensic hospital	Patients were unfamiliar with limit setting as a term and understood it as setting boundaries for behaviour. Staff didn't like the term as they perceived it to mean controlling the patients. Both suggested it was necessary to ensure safety. Overall, a therapeutic relationship and a consistent, empathetic, authoritative, and knowledgeable approach to limit setting was deemed important.	Fair
Mahoney et al. (2012) USA	Pre-post questionnaire design/ Quantitative	Psychiatric Hospital	Staff: pre-intervention (n=108), post-intervention (n=143)	To explore how a programme aiming to improve team work and patient safety can be used effectively by describing the implementation process and identifying any team differences before and after implementation	The programme was implemented with success. The team attributes positively differed post-intervention ($p<.01$). This included team foundation, functioning, performance, skills and climate and atmosphere.	Poor
Martin & Daffern (2006) Australia	Questionnaire design/ Quantitative	Forensic psychiatric inpatient hospital	Staff (n=69); 46% female	To examine staff perceptions of personal safety and confidence	Clinicians reported feeling safe and confident in managing a violent incident. However, staff reported factors that affected their ability to feel confident including: knowledge, experience and skill; working as a team; and their use of intervention strategies.	Fair
Mezey, Hassell & Bartlett (2005) UK	Semi-structured interviews/ Qualitative	Single-sex and mixed-sex medium secure units (n=16)	Inpatients (n=31); 19% female. Staff (n=58); 53% female	To assess the extent to which women inpatients consider themselves to be safe and to whether these views are identified in staff	Patients in both types of unit experienced violence, both sexual and physical. However, women patients in the single-sex units experienced more intimidation, threats and physical abuse but less sexual and physical violence.	Fair

For peer review only

Millar & Sands (2013) Australia	Exploratory descriptive design/ Qualitative	Acute inpatient psychiatric unit	Inpatients (n=25) covering 500 handovers	To identify the frequency and type of risk information transferred at handover	Patient safety is compromised by the reduced or inadequate communication over risk. Verbal communication was the most common method for handover updates.	Good
O'Brien & Cole (2004) Australia	Participatory action research/ Qualitative	Acute inpatient general hospital psychiatric facility	Patients, relatives, carers and nurses (n=42)	To explore the context and experiences of nurses, patients, and relatives in the close-observation area and to develop recommendations for clinical practice	Data revealed three main themes: design and environment (concern was raised about the lack of privacy and security), lack of activity and structured time, and nursing care (some patients indicated that they did not feel cared about).	Fair
O'Neill, et al. (2003) Ireland	Structured interviews/ Mixed methods	A psychiatric hospital	Inpatients. Length of Stay < 2 years (n=45); median age 29.3) Length of Stay > 2 years (n=43); Medium age 48.8, and key workers.	To describe the patient groups present in Irish forensic psychiatric beds and to ascertain unmet treatment and placement needs	Twenty (47%) of the long-stay group and eleven (24%) of those with shorter durations of stay were felt to be inappropriately placed. 30% of long-stay patients could be safely transferred to lower levels of security within six months and 63% within three years.	Fair
Rees & Manthorpe (2010) UK	Semi-structured interviews/ Qualitative	Residential services (n=8)	Managers (n=13) and care workers (n=10).	To explore the impact of adult protection investigations on managers of residential services and staff	Service managers stated that the application of policy and practice can be both beneficial and detrimental to their services (including service disruption, stress for residents, staff and managers). Multi-agency collaboration, transparency, training, reflective practice, and effective supervision were given as influencing the implementation of adult protection policies.	Fair

For peer review only

Ryan (2007) Europe	Longitudinal/ Quantitative	Acute inpatient psychiatric centres	Staff (n=205)	To compare, across six European mental health services, the levels of occupational stress and burnout among mental health workers in acute psychiatric hospitals	There were no statistically significant differences between sites and teams regarding emotional exhaustion. Great Britain had the highest score for depersonalisation.	Fair
Salzmann-Erikson, et al. (2008) Sweden	Cross-sectional/ Qualitative	A PICU	Caregivers (n=18); 50% female; age range 23-56.	To describe which care activities are practiced by registered nurses and assistant nurses in the PICU.	Categories describing the fundamentals of the PICU were found: protests and refusal of treatment due to lack of capacity, escalating behaviours, and using coercive measures to manage violence.	Fair
Silvana, et al. (2012) Italy	Cross-sectional/ Qualitative	A psychiatric ward	Nurses (n=20)	To plan ergonomic improvement from preliminary results of a psychiatric ward case-study	Ergonomic issues were found: a locked and polluted environment (smoking indoors) and an unusual staff gender ratio (hospital management considers staff gender ratio a safety issue due to men's strength, allowing them to cope with aggressive behaviour). No mental health care programmes are available for nursing staff, not even after safety incidents.	Fair
Sjöstrand, et al. (2015) Sweden	Cross-sectional/ Qualitative	General psychiatry, forensic psychiatry, and addiction psychiatry settings	Psychiatrists (n=8); 50% male; age range 30-68.	To explore the psychiatrists' ethical reasoning regarding involuntary psychiatric treatment.	Participants were focused on the consequences of involuntary treatment, balancing risk of damaging the therapeutic alliance against ensuring patients received the treatment they needed. Suicidal and psychotic patients were examples where involuntary care was seen as justified. However, it was also argued that risk of	Fair

For peer review only

					suicide might not be sufficient for justified involuntary care. Organisational factors were seen as sometimes resulting in compulsory treatment that could have been avoided.	
Stead, et al. (2009) Australia	Pre-post intervention/ Quantitative	An inpatient mental health facility	Multidisciplinary staff	To evaluate the implementation of a staff educational program aimed at increasing team work and patient safety, addressing three outcomes: observed team behaviours; attitudes and opinions; and clinical performance and outcomes	Changes implemented included the restructuring of multidisciplinary meetings and the introduction of structured communication tools. There was also a significant improvement in aspects of patient safety culture (frequency of event reporting, and organisational learning) and knowledge, skills and attitudes scores increased by 6.8%. Seclusion rates also reduced.	Fair
Stein (2002) UK	Cross-sectional/ Qualitative	Mental health inpatient facilities	Clinical directors (n=25), medical directors, (n=22), managers (n=6), consultants (n=3), nurses (n=1), other (n=1)	To explore the development and effective use of tools to predict risk to patients and public following discharge into the community	60.3% of sites used a discharge checklist, so development of risk assessment tools is varied at Trust level. Developments seem to be stimulated by clinical governance in the absence of an overarching national strategy. It seems that information technology for risk data collection is not widely used, instead using paper and informal communication.	Fair

For peer review only

Stübner, Groß & Nedopil (2006) Germany	Cross-sectional/ Quantitative	Forensic institutions (n=8)	Heads of department or their locum tenens	To investigate risk factors for incidents during hospitalization and criteria allowing easing of regulations and confinement	Among the risk factors, 87% of the terms related to patient characteristics, while among the protective factors the proportion was 77.5%. Good relations with the therapeutic team accounted for 14.6% of the criteria for easing restrictions, while poor relations accounted for less than 4% of the risk factors.	Fair
Wood & Pistrang (2004) UK	Cross-sectional / Qualitative	An acute inpatient psychiatric unit	Patients (n=9); nursing staff (n=7)	To explore the experiences of safety and threat from the perspective of psychiatric inpatients	Ten themes relating to factors that influenced feelings safety were grouped into three clusters—patient interactions, staff behaviour and attitudes, and non-consensual treatment. Patients expressed feeling vulnerable and helpless.	Fair
Woods (2013) Canada	Cross-sectional / Qualitative	Inpatient mental health units (n=7) and a forensic unit	Registered Psychiatric Nurses (n=33); Registered Nurses (n=2); Licensed Practical Nurse (n=1); Special Care Aides (n=7); Social Workers (n=2); Student Nurse (n=1); and other (n=2). Total (n=48)	To identify and describe current risk assessment and management approaches used in adult inpatient mental health and forensic units	Key issues were discussed as important: the pitfalls of relying on clinical judgement alone; considering risk as a wider concept; risk management being reactive; lack of education and training, and client involvement.	Fair
Ward (2013) Australia	Cross-sectional / Qualitative	An inpatient mental health care facility	Nurses (n=13); 100% female	To investigate nursing practices, the nurse-patient relationship, violence, and aggression in acute inpatient mental health care	Some workplace stressors included poor staffing skill matrix, complex patient diagnoses, and limited workspace design. Effective communication was seen as essential to prevent violence. Coping mechanisms included debriefing with other colleagues.	Good

For peer review only

Nathan, et al. (2007) UK	Prospective/ Quantitative	A medium secure forensic psychiatric hospital	Nurses on the women's ward that were followed-up (n=14); 71% female; mean age 38.1 (SD=10.00). Nurses on the men's ward that were followed-up (n=14); 93% male; mean age 37.7 (SD=9.8)	To explore gender differences in psychopathology in forensic mental health settings and how this may affect the risk of staff burnout	Nurses on the women's ward were found to score significantly higher in the emotional exhaustion component of burnout. Suggested inter-related factors that are key to the development of emotional exhaustion include: nature of the mental disorder may differ between genders, and men can display more aggressive behaviour whilst women display more relational aggression.	Fair
Qi, et al. (2014) China	Cross-sectional/ Quantitative	Psychiatric hospitals (n=2)	Nurses (n=705); 100% female; mean age 31.3 (SD=7.1)	To compare the level of work-related stress between female nurses working in psychiatric and general hospitals and explore associated individual and environmental factors	Psychiatric nurses were more likely to be exposed to workplace violence (23.5% vs. 80.5%, p < .001), and had greater levels of stress in the domains of working environment (p < .001) and patient care (accidents, patient-staff relationships, and the impact of patient suffering or death) (p < .001).	Good
Vlayen, et al. (2012) Belgium	Cross-sectional / Quantitative	Acute (n=90), psychiatric (n=42) and long-term (n=11) care hospitals	Staff in acute (n=47,635), psychiatric (n=6341) and long-term care hospitals (n=1249)	To explore patient safety culture in Belgian hospitals and the underlying safety culture dimensions	Strengths of patient safety culture were: team work, supervisor/ manager expectations and actions promoting safety, and organisational learning. Handoffs and transitions, staffing, management support for patient safety, non-punitive response to error and teamwork across units could be improved. Positive dimension scores were higher for psychiatric and long-term care hospitals than for acute hospitals, suggesting that patient	Good

For peer review only

safety is more encouraged within these settings.

For peer review only

Harm to self

Author, year, country	Study design	Setting	Participants	Aims/ objectives related to patient safety	Outcomes related to patient safety	Study quality
Booth, et al. (2014) Ireland	Questionnaire/Quantitative	A psychiatric hospital	Patients (n=114). 81% female. Mean age 35	To evaluate the effectiveness of a brief intervention for decreasing self-harm and increasing distress tolerance.	There was a decrease in the frequency of self-harm incidents from pre-intervention (M=13.68, SD=21.81) to post-intervention (M=4.50, SD=11.01) (p=.02, N=48) and from pre-intervention to 3 months post-intervention (M=3.62, SD=11.33) (p=.01, N=48). There was an increase in distress tolerance from pre-intervention (M=6.32, SD=2.35) to post-intervention (M=10.36, SD=3.68) (p=.00, N=32) and from pre-intervention to 3 months post-intervention (M=9.72, SD=4.33) (p=.00, N=32). There was a decrease in inpatient days from pre-intervention (M=39.90, SD=33.25) to post-intervention (M=23.09, SD=40.56) (p=0.01, N=65), from pre-intervention to 3 months post-intervention (M=8.78, SD=25.40) (p=.00, N=65) and from post-intervention to 3 months post-intervention (p=.00, N=65).	Fair
Bowers, et al. (2008) UK	Questionnaire/ Quantitative	Acute psychiatric wards (n=136) in 67 hospitals within 26 National Health Service (NHS) Trusts	Staff and patients within the units	To explore the relationship between special observation and self-harm rates in acute psychiatric wards	Constant special observation was not associated with self-harm, but intermittent observation (OR=0.82 (0.78–0.87); p<0.001), levels of qualified nursing staff (OR=0.94 (0.90–0.98); p<0.01), and more intense programmes of patient	Good

For peer review only

					activities (OR=0.53 (0.38–0.75); p<0.001) were associated with reduced self-harm.	
Brown & Beail (2009) UK	Semi-structured interviews/ Qualitative	A secure service for people with intellectual disabilities and challenging behaviour	Residents (n=9). 56% male.	To explore the experiences of self-harm, and interventions for this self-harm, among residents with intellectual disabilities within secure accommodation	Self-harm management interventions imposed by the service were mostly perceived as controlling, punitive and evoked strong negative reactions from residents.	Good
Caspi (2014) USA	Observation and semi-structured interviews/ Qualitative	Special dementia care units within an assisted living residence (n=2)	Residents (n=12). 92% female. Mean age 81 (range 75-86).	To explore self-neglect and other behaviours when residents with dementia are unsupervised within assisted living residences	There were 158 incidents of negative behaviour expression, emotional states and self-neglect. 56% of these were self-neglect incidents. 97% of these took place during times when residents were not engaged in structured activity. The incidents included a wide variety of safety risks, including falls, attempted absconding and risk of aggressive interactions.	Good
Cleary, et al. (1999) Australia	Interviews/ Qualitative	Acute inpatient psychiatric wards (n=4) within a psychiatric hospital	Clinical registered nurses (n=10)	To explore the role of registered nurse in the care of patients on special observation	9 themes were developed to highlight the topics discussed by nurses when considering their role in the special observation process: safety; therapeutic relationships; supporting patients and carers; consequences of special observation for nurses; continuity of care concerns; peer support; suicide indicators; responsibilities and rights; and nurses, doctors and the hospital hierarchy.	Fair

For peer review only

Davis, Williams & Hays (2002) USA	Questionnaire/ Quantitative	An acute-care psychiatric facility	Patients (n=135). Mean age 36. 70% female	To explore the views of suicidal psychiatric inpatients concerning no-suicide agreements	Patients had an overall positive view of no-suicide agreements, relating to the therapeutic features, coerciveness and detachment of the process. 92% of participants agreed that they had confidence in their ability to keep to their commitment.	Fair
de Jonghe-Rouleau, Pot & de Jonghe (2005) Netherlands	Questionnaire/ Quantitative	Psycho-geriatric wards of a large nursing home (n=3)	Residents (n=110). 77% female. Mean age 83 (range 67–105)	To explore self-injurious behaviour (SIB) in nursing home residents with dementia	SIB was reported in 22% of patients. Pinching, scratching and banging one's fist against objects were the most commonly reported behaviours (8% of patients). SIB was associated with: prescribed psychotropics (OR=5.62), immobility (OR=3.71) and restraint (OR=6.19).	Fair
Drew (1999) USA	Questionnaire/ Quantitative	Psychiatric hospitals (n=84)	Staff	To explore the use of no-suicide contracts in psychiatric hospitals	The most common suicide prevention intervention used by the hospitals was limiting access to objects used for self-harm (100%). 79% of the hospitals used no-suicide contracts. Contracts were mostly drawn up after suicidal ideation was expressed (83% of hospitals that used no-suicide contracts) by nurses. They were mostly verbal (74%). 53% of respondents estimated suicide behaviour frequency of 10+ incidents a year.	Fair

For peer review only

Ellis, et al. (2012) USA	Open-trial case-series/ Quantitative	A private psychiatric hospital	Patients (n=20). 80% female. Mean age 37 (range 21-55)	To evaluate a program designed to reduce the risk of suicide in inpatient psychiatric hospitals	From pre-post treatment, there were significant reductions (effect sizes= > .80) in depression, hopelessness, suicide cognitions, and suicidal ideation, as well as improvement on contributory factors of suicidality. The factors with the highest effect sizes were: depressive symptoms, psychological pain, and self-hate.	Fair
Ellis, et al. (2015) USA	Controlled trial/ Quantitative	A private psychiatric hospital	Patients (n=52). 69% female. Mean age 33 (range 18-68)	To evaluate the effectiveness of a program designed to reduce the risk of suicide in inpatient psychiatric hospitals above and beyond that of intensive, psychotherapeutic, milieu-based treatment	The program group showed greater improvement on measures of suicidal ideation and suicidal cognition (effect sizes= > .80).	Fair
Esposito-Smythers, McClung & Fairlie (2006) USA	Descriptive/ Quantitative	An acute adolescent psychiatric inpatient unit	Patients (n=250). 60% female. Mean age 15 (range 12-18)	To explore participant perceptions of a suicide prevention group for psychiatrically hospitalised patients	94% of participants reported that they learnt something from the group that would prevent them attempting suicide in the future. The most helpful part of the group was reported to be the Reasons to Live List (32% of participants stated this was most helpful). The least helpful part was reported to be the Safety List (30%).	Fair
Gibson et al. (2014) Ireland	Controlled trial/ Quantitative	Psychiatric hospital	Psychiatric inpatients (n=103). (range 18-60); Living through distress programme (LTD), (n=82); 79% female; mean age 38 and waiting list (TAU) (n=21); 57% female; mean age 32	To examine whether additional DBT skills training improved outcomes including self-harm compared to TAU	Self-harm significantly reduced in the LTD group compared with TAU (49% of the LTD group had a reduction in self-harm of 75% or greater) and this was maintained at 3 month follow-up.	Fair

For peer review only

Gough & Hawkins (2000) UK	Questionnaire/ Quantitative	Forensic psychiatric hospital	Clinical staff (n=77)	To explore staff attitudes regarding self-harm in psychiatric patients	Staff attitudes towards self-harm varied. Staff felt self-harm was used to communicate with others about how distressed they are (M=2.9; SD = 0.8). Some staff felt self-harm wasted staff time (M=1.9; SD= 1.0). Overall, staff felt more training on self-harm was needed.	Poor
Kool et al. (2014) Netherlands	Quasi-experimental pre-post-test/ Quantitative	Mental health centres (n=8) and a forensic-psychiatric centre (n=1)	Staff (n=178); mean age 38	To measure the effects of a self-harm reduction programme on attitudes towards self-harm patients; self-efficacy in managing self-harm patients; and the distancing of self-harm patients and treatment staff	After the programme, staff were significantly more likely to have perceived confidence in assessing and referring self-harm patients; managing them effectively; have an emphatic approach; and be able to cope effectively with regulation in relation to self-harm guidance.	Fair
Lindgren, Aminoff & Graneheim (2015) Sweden	Participant observational and interview study/ Qualitative	Psychiatric hospital	Women inpatients (n=6); median age 23.5 (range 21-37 years)	To describe features of everyday life of patients who self-harm	The main factor associated with everyday life was being surrounded by the disorder. This encompassed residing in a confusing environment, being confined to routines and rules around safety that lack consistency. Loneliness was an issue. Staff spent limited time with the patients.	Fair

For peer review only

O'Donovan (2007) Ireland	Semi-structured interviews/ Qualitative	Acute psychiatric inpatient units (n=2)	Psychiatric nurses (n=8); 75% female (age range 25-55)	To explore the practices of psychiatric nurses relating to people who self-harm, but are not suicidal	Participants emphasized preventing self-harm and providing a physically safe environment. The primary methods for ensuring a service user's safety were: removal of sharp objects; and requesting that service users stay in their night clothes. Participants reported spending 15 to 90 min a day with each service user in therapeutic interaction. This was then used to inform staff of the potential for self-harm, to assess mood, and to inform future care.	Fair
Sansone, McLean & Wiederman (2008) USA	Cross-sectional/ Quantitative	A community hospital	Inpatients (N=120). 61% female; mean age 38.69, SD=11.74 (range 18-74)	To explore, in a sample of psychiatric inpatients, the relationship between self-sabotaging behaviours and borderline personality disorder	76 respondents (63.3%) reported engaging in medically self-sabotaging behaviour, with the average number of different behaviours of this type being 4.11 (SD = 3.93). The most commonly endorsed behaviours (endorsed by around a quarter of participants) were: damaging self on purpose and seeking medical treatment; not going for medical treatment; to purposefully hurt self; not taking a prescribed medication; and involvement in dangerous situations.	Fair
Sjöström, Hetta & Waern (2012) Sweden	Prospective cross-sectional/ Quantitative	Psychiatric units	Inpatients who attended follow-up interview (n=98)	To investigate whether low Sense of Coherence (SOC) is a predictor of suicidality and of risk of a repeat suicide attempt	2 months after a suicide attempt, low baseline SOC was significantly associated with an eightfold increase in risk of high suicidality (P = <0.001). Low SOC score at baseline was also associated with repeat attempts within 3 years	Good

For peer review only

					(*P* = 0.038), but was not an independent predictor when other mental health symptoms were taken into account.	
Sun, et al. (2005) Taiwan	Cross-sectional/ Qualitative	Psychiatric admission wards	Patients (n=15); 60% female; age range 16-47. Psychiatric nurses (n=15); 100% female; age range 21-49.	To develop a suicide care theory that could help nurses to improve suicide prevention and care	In developing the theory, four categories were highlighted relating to interaction strategies: holistic assessment of people who are suicidal; protection; basic care; and advanced care.	Fair
Sun, et al. (2006) Taiwan	Cross-sectional/ Qualitative	Acute psychiatric wards and a psychiatric stress ward	Patients (n=15); 60% female; age range 16-47. Psychiatric nurses (n=15); 100% female; age range 21-49.	To: (1) explore and examine psychiatric nurses' and patients' perceptions of the care offered to patients with suicidal ideations on psychiatric wards, and (2) develop a nursing theory to guide the care of patients at risk of suicide	Fifteen categories were generated, relating to contexts and intervening conditions of care for suicidal patients. These categories centred on the therapeutic relationship, staff attitudes, blame, autonomy, staff powerlessness due to lack of training, and time constraints.	Fair
Swogger, Van Orden & Conner (2014) USA	Cross-sectional/ Quantitative	Acute inpatient hospitals (n=3)	Patients (n=892); 58% male; mean age 29.9 (SD=6.2) age range 18-40	To study the relationship of outwardly-directed aggression to suicidal ideation and attempts in psychiatric inpatients	Outwardly-directed aggression was associated with suicide attempts, but not ideation. It was also associated with planned, but not unplanned, suicide attempts.	Fair

For peer review only

Takahashi, et al. (2011) Japan	Cross-sectional/ Quantitative	Psychiatric medical institutions (n=8)	Nurses (n=531); 63% female; mean age 41.9 (SD=12.3)	To investigate issues related to staff experiences of patient suicide in mental health nursing	55% of participants had experienced patient suicide. 13.7% of participants were at a high risk of post-traumatic stress disorder. However, only 15.8% stated that they had access to post-suicide mental health care programmes. There was also low attendance at in-hospital seminars on suicide prevention or mental health care for nurses (26.4% and 12.8% respectively).	Fair
Tofthagen, Talseth & Fagerström (2014) Norway	Cross-sectional/ Qualitative	Psychiatric clinics (n=4)	Mental health nurses (n=15); 87% female	To explore the experiences of mental health nurses in caring for inpatients who self-harm	Two main categories were identified: challenging and collaborative nurse-patient relationships, and promoting well-being through nursing interventions. Participants sought to understand the self-harm behaviour, its triggers and signs in a person-centred way, helping patients learn to be aware of their feelings/behaviours and learn coping strategies.	Fair

For peer review only

Vråle & Steen (2005) Norway	Cross-sectional/ Qualitative	Acute psychiatric wards	Nurses (n=5); 60% female	To explore the dynamics of performing constant observation of suicidal patients	The process of constant observation seems to follow organizing phases, from assessment to transitioning out of the process. The process requires a balance between the need for control and keeping the patient from self-harm, structure and flexibility, as well as maintaining a therapeutic relationship.	Fair
Weber (2002) USA	Cross-sectional/ Qualitative	A locked psychiatric hospital	Patients (n=9); 100% female	To explore how self-abusing women in a state psychiatric hospital defined self-abuse	Four themes were identified: the need to be listened to and receive help; specific triggers for self-abuse (noise on the ward, especially screaming and profanity); the causes of self-abuse; and how to stop the self-abuse (someone talking to them during times of crisis, and staff providing comfort or distraction).	Fair

For peer review only

Lundegaard Mattson & Binder (2012) Norway	Cross-sectional/ Qualitative	A psychiatric hospital	Healthcare workers (n=8)	To explore the perceptions, emotions and actions of healthcare staff when working with patients who self-harm	Several themes emerged, detailing: the frustration at having to use coercive interventions; the process of change from coercion to alliance; experiences of useful management strategies; and the distinction between self-harm and suicide attempt. On a structural level, a cap was put on the number of patients diagnosed with Emotionally Unstable Personality Disorder, and staff felt that this made it easier to work with patients, reducing frequency of self-injury. Management also became more involved in supporting staff training to reduce use of coercion.	Good

For peer review only

Safety of the Physical Environment

Author, year, country	Study design	Setting	Participants	Aims/ objectives related to patient safety	Outcomes related to patient safety	Study quality
Bellantonio, et al. (2008) USA	Randomized controlled trial/ Quantitative	Dementia-specific assisted living facilities (n=2)	Residents of the facilities with dementia (control group, n=52; intervention group, n=48)	To investigate the effects of a multidisciplinary team intervention on unexpected transitions from assisted living in people with dementia	Falls were the most frequent reason for unanticipated transition (n=40). The intervention reduced the risk of unanticipated transitions by 13%, but this was not statistically significant (p=0.67). The intervention reduced the risk of death by 63%, but this was not statistically significant (p=0.08).	Good
Bowers & Crowder (2012) UK	Questionnaire/ Quantitative	Acute psychiatric wards (n=32)	All staff and patients within the units	To explore the relationship between staffing numbers and adverse incidents on the wards of psychiatric hospitals	Lower qualified nurse staffing levels were associated with higher conflict (Incident Rate Ratio (IRR) =1.03)) and containment (IRR=1.03) rates.	Good
Bowers, et al. (2010) UK	Questionnaire/ Quantitative	Acute psychiatric wards (n=136) in 67 hospitals within 26 National Health Service (NHS) Trusts	Staff (n=638; 62% female; modal age group 25-34), patients (n=393; 52% female; modal age group 35-44) and visitors (n=168; 50% female; modal age group 35-44) within the units	To explore the acceptability of door locking to staff, patients and visitors within acute psychiatric wards	Five factors were found to relate to the acceptability of door locking: adverse effects, staff benefits, patient safety benefits, patient comforts and cold milieu. Patients felt more negatively about door locking than staff.	Fair
Chandler (2008) USA	Interviews/ Qualitative	An inpatient psychiatric unit within a community hospital	Staff (n=10)	To explore the experience of staff when moving from traditional care to trauma-informed care within inpatient psychiatric units	The transition was described by staff in the context of creating a culture of safety. The overarching theme was of transferring control from staff to patients, and this included: changed perspectives, collaborative patient–staff relationships, the implementation of safety protocols, and the prescription of individualized evidence-based educational resources.	Fair

For peer review only

Cowman & Bowers (2008) UK & Ireland	Comparative questionnaire/ Quantitative	Acute psychiatric wards in England (n=87) and acute admission psychiatric wards in Ireland (n=37)	Staff	To explore safety and security measures in acute psychiatric wards in England and Ireland	Irish wards were found to have higher levels of security and safety restrictions. Irish wards are more likely to ban items, restrict access to areas/items, routinely search patients, have access to security guards at all times, and have staff carry personal alarms. Door locking procedures were similar, with 29% of English wards and 28% of Irish wards always keeping doors locked.	Fair
Curtis, et al. (2013) UK	Unstructured interviews/ Qualitative	An inpatient mental health care facility	Staff (Phase 1 n=23; Phase 2 n=8; Phase 3 n=26)	To explore the views of staff concerning technical safety and therapy in the design of an inpatient mental health facility	Participants mentioned how responsibility for technical safety was being invested in the physical infrastructure. Surveillance and observation were also important. Staff felt that relying on technical safety measures meant shirking responsibility for risks they should manage. They had concerns about how focusing on technical safety might conflict with a therapeutic approach.	Fair
Gebhardt & Steinert (1999) Germany	Pre-post questionnaire/ Quantitative	Acute general psychiatry wards (n=4) within a psychiatric hospital	Patients (n=183). 55% female. Mean age (Time 1=38; Time 2=38; Time 3=36). Staff (n=162)	To explore the effects of an equal distribution of severely disturbed patients on social climate, aggressive behaviour, and sexual molestation in acute wards	A significant improvement of ward atmosphere (Rao's R=1.654, P=0.037) and a reduction of aggressive behaviour (χ2>16, df=2, P<0.001) was found after structural changes were implemented, whereas the impact on sexual molestation could not be determined as these events were rare at baseline.	Fair

For peer review only

Haglund & von Essen (2005) Sweden	Semi-structured interviews/ Qualitative	Psychiatric inpatient unit	Voluntarily admitted inpatients (n=20); median age 43 years (range 19-87)	To describe patient perspectives of advantages and disadvantages of being cared for on a unit that has locked doors	Advantages included protecting patients and staff from external factors, providing patients with security and efficient care, and providing staff with a sense of control. Disadvantages included: making patients feel confined, making patients dependent on staff, and making patients feel worse emotionally.	Poor
Hunt et al. (2012) UK	Descriptive/ Quantitative	Psychiatric inpatient wards	Inpatient suicides who died by hanging between 1999-2007 (n=344); median age 39 (range 17-85); 63% male	To establish the principal ligatures and ligature points on the ward and whether there are trends over time	During 1999-2007, a quarter of inpatients who died by hanging, did so within a week of admission (25%); half had been on a high or medium observation level (49%). A third of the deaths were thought to be preventable (33%). The door, hooks, handles and windows were collectively the most common ligature points (59%). Belts, sheets or towels accounted for almost two-thirds of the ligatures (61%).	Fair
Kulkarni et al. (2014) Australia	Descriptive/Quantitative	Psychiatric ward (n=2)	Inpatients (n=65) and staff (n=20)	To evaluate the impact of a female-only area within a mixed-gender inpatient psychiatry service on female experiences of patient safety and care	There were significantly more patient safety incidents within the mixed-gender inpatient ward compared to the female only area. Specifically, female patients and staff perceived female-only wards as safer.	Fair

For peer review only

Muir-Cochrane et al. (2012) UK	Semi-structured interviews/ Qualitative	Acute psychiatric unit	Participants (n=35); 43% female; mean age 43 years. Inpatients (n=15); visitors (n=6); nurses (n=14)	To explore perceptions of the acceptability of locking doors on inpatient wards	Several themes found that acknowledge the importance of locking doors on inpatient wards. Generally, all participants felt door locking helped reduce absconding. Staff felt guilt and fear of being blamed for the patient absconding, whereas patients expressed depression and low self-esteem when doors were locked.	Fair
Simpson, et al. (2011) UK	Cross-sectional/ Quantitative	Acute psychiatric wards (n=136)	Exit security staff and qualified nurses	To investigate the relationship between rates of drug/alcohol use on acute psychiatric wards and exit security measures	Daily rates of alcohol/drug use by wards are likely to be skewed, as few wards reported high levels. Door locking and security measures do not seem to be significantly related to alcohol use (r=-0.035, p=0.690) or other substance use (r = 0.108, p = 0.216).	Fair
Stolker, Nijman & Zwanikken (2006) Netherlands	Prospective/ Quantitative	A locked ward of a psychiatric hospital	Patients (n=54); 70% male; mean age 36, age range 18-58.	To explore whether residing in single- versus multiple-bed rooms in a psychiatric ward influenced psychiatric patients' views regarding seclusion	Findings suggest that seclusion is perceived as a less negative experience after having resided in a multiple-bed room before being secluded, t (30) = 3.4, P < .05, compared to patients residing in a single bed room.	Good
Van der Schaaf, et al. (2013) Netherlands	Longitudinal/ Quantitative	Forensic wards (n=25)	Patients (n=616); 78.7% male; mean age 37.6 (SD=10.5)	To explore the effect of design features on the seclusion risk, frequency, and duration within locked wards for intensive psychiatric care	Design features increasing the risk of being secluded were: 'presence of an outdoor space'; the availability of 'special safety measures'; and a large 'number of patients in the building'. Design features decreasing the risk of being secluded were: more 'total private space per patient'; a higher 'level of comfort'; and greater 'visibility on the ward'.	Good

For peer review only

Verbeek, et al. (2014) Netherlands Ì	Quasi-experimental/ Quantitative	Nursing homes with specialized psychogeriatric, somatic, or rehabilitation wards	Residents (n=124) in small-scale living facilities; 80% female; mean age 82.4 (SD=7.9). Control (n=135); 70% female; mean age 83.1 (SD=6.5)	To examine the effects of small-scale living facilities on residents' behaviour, focusing on neuropsychiatric symptoms, social engagement, and the use of physical restraints and psychotropic drugs	The small-scale facilities employed significantly fewer physical restraints and psychotropic drugs compared with traditional wards. Levels of social engagement and physically non-aggressive behaviour were higher in small-scale facilities than traditional wards.	Good
Wilkes, et al. (2005) Australia	Quasi-experimental/Quantitative	A Special Care Unit (SCU) at a nursing home	Persons with dementia (n=16); 81% female	To explore the effects on challenging (agitated) behaviours, when relocating patients with dementia to a special unit	Results revealed no significant differences in resident aggressive behaviour scores ($P = 0.220$) over time. Verbal agitation reduced throughout the 6 months of the study after moving into a SCU ($P < 0.01$).	Fair

For peer review only

Medication safety

Author, year, country	Study design	Setting	Participants	Aims/ objectives related to patient safety	Outcomes related to patient safety	Study quality
Bademli & Buldukoglu (2009) Turkey	Questionnaire/ Quantitative	Inpatient psychiatric wards in hospitals (n=34)	Psychiatric nurses (n=471); 100% female. Mean age 32.4+/-7.3	To describe oral medication management by nurses in psychiatric wards	73.9% of nurses collected medication history data, with 90.23% asking whether the patient had taken the medication previously. The orders were checked by 80.5% of nurses every day. If a nurse was not sure of something, 93.4% asked the physician. 59.7% of the nurses checked all the patients' mouths after each pill was given. The most common patient reaction during medication administration was refusal to take the medication (93.2%). 86.8% of nurses informed the physician when this occurred. Nurses primarily observed the patient to evaluate the effect of a medication (84.3%). Age, education and years of experience created a significant difference in approaches to patients who did not take their medications.	Fair
Cottney (2014) UK	Pre-post observation/ Quantitative	An acute adult inpatient mental health ward	Staff	To evaluate the benefits of automated dispensing cabinets (ADCs) for increased medication safety	There was a reduction in the rate of medication administration errors from 8.9% to 7.2%, but this was not statistically significant (p=0.065, 95% CI 0% to 3.5%.	Poor

For peer review only

Cottney & Innes (2015) UK	Observation/ Quantitative	Inpatient mental health wards (n=43) within a mental health trust	Staff and patients	To explore medication-administration errors within a mental health trust	In 4177 medication administration opportunities, 139 medication errors were detected (3.3%). The error most frequently made was incorrect dose omission (37%). 11% of the errors had a serious clinical severity level. Four factors predicted medication error: administering nurse having to attend to other duties during the medication round (relative risk (RR) =1.48, 95% (CI): 1.14–1.93, p=0.003); number of 'pro re nata' (prn) doses given (RR=1.15, 95% CI: 1.03–1.28, p=0.012); number of patients on the ward (RR=1.06, 95% CI: 1.01–1.13, p=0.03); and number of regular doses due (RR=1.02, 95% CI: 1.02–1.03, p=≤ 0.0001).	Good
Dickens, Stubbs & Haw (2008) UK	Observational/Quantitative	Inpatient wards for older people (n=2) within a psychiatric hospital	Registered nurses (n=9)	To explore the delegation of medication administration within older people mental health wards	78% of delegated medication doses were to another registered nurse, but 22% were delegated to care workers. Care workers were more likely to administer medication to aggressive/confused patients. Errors occurred in 20% of doses, with the most common being the opening/crushing of medication without authorisation (41% of errors). 99% of errors were in the preparation/recording and so not attributable to the administrator of the medication.	Fair

For peer review only

Dolan & Kirwan (2001) UK	Questionnaire/ Quantitative	A medium secure unit for mentally disordered offenders (MSU)	Staff (n=62)	To explore staff perceptions of illicit drug use and its impact on MSUs	60% of staff reported awareness of drug misuse on the unit. The majority of staff (82%) mentioned the adverse effects on psychopathology (worsening symptoms, aggression). 45% also expressed concern regarding negative impacts upon staff-patient relationships. Most staff commented that the unit had policies of bag, room or visitor searching. 31% of staff felt that the police should be called if patients were in possession of drugs, 40% felt that sanctions should be imposed.	Fair
Haw et al. (2007) UK	Cross-sectional/ Mixed methods	Elderly long-stay wards in a psychiatric hospital (n=2)	Nurses (n=9)	To use observations to determine the frequency and nature of medication administration errors	In 1423 doses, 369 medication errors were made (25.9%). The most frequent error type was crushing tablets without the authorization of the prescriber (28.7%). The median rate of medication error was one in every 6.4 doses.	Poor
Haw et al. (2014) UK	Vignettes and semi-structured interviews/ Qualitative	Psychiatric hospital	Nurses (n=50)	To explore reasons for not reporting medication errors	48% of nurses stated that they would report a medication error made by someone else, and 40% would report a near-miss involving themselves. Thematic analysis revealed reasons for this was four-fold: knowledge, fear, burden of work, and excusing the error.	Fair
Prins, et al. (2013) Netherlands	Observation and structured interviews/Quantitative	An inpatient old age psychiatric clinic of a large psychiatric teaching hospital	Patients (n=50); mean age 68.9; 52% female	To compare the number of medication use discrepancies at admission when using the structured history of medication use (SHIM) procedure and usual procedure for taking medication history	Compared with usual care, the SHIM procedure identified a discrepancy in medication use in 78% of patients, and provided a more comprehensive and accurate approach to obtain medication history.	Good

For peer review only

Ružić, et al. (2011) Croatia	Cross-sectional/ Quantitative	Forensic psychiatric institutions (n=2)	Psychiatric patients (n=98); mean age 47	To examine the effect of antipsychotics selection (typical or atypical) on patient aggressiveness, side effects, and hospitalisation length	There were no significant differences between participants receiving typical and atypical antipsychotics.	Fair
Seemüller, et al. (2009) Germany	Naturalistic prospective/ Quantitative	Psychiatric university hospitals (n=7) and psychiatric district hospitals (n=5)	Inpatients (n=1014); 63% female; mean age 45.03 (SD=11.89)	To explore any suicidality promoting effects of antidepressants that cause an increased rate of suicidality-related events in high risk populations under routine treatment conditions	The rate of suicides (13.44/1000 patient-years) was low in the routine treatment condition compared to the rate observed in randomized controlled antidepressant trials. Predictors of extended emergence of suicide ideation were: Age (younger individuals were at higher risk), treatment resistance, number of hospitalizations, and presence of akathisia and comorbid personality disorder.	Good
Sørensen, et al. (2013) Denmark	Descriptive, cross-sectional/ Quantitative	Psychiatric wards at a university hospital (n=3)	Patients (n=67); 64% female; mean age 46, age range 20-79	To investigate errors in the medication process in an inpatient psychiatric setting	There were errors in 9/324 (3%) opportunities for error of dispensed drugs in the observational study, and in 9/67 (13%) of dispensed drugs in the unannounced control visit, of which the majority was associated with one nurse assistant. Fewest errors were detected at the prescribing stage.	Fair
Gonzalez-Pinto, et al. (2010) Spain	Prospective Observational/ Quantitative	An inpatient setting	Non-adherent inpatients (n=161) and adherent inpatients (n=460)	To describe the frequency of adherence and non-adherence with anti-manic and mood stabilizing medication among bipolar disorder patients, and to identify factors associated with treatment adherence	Higher levels of insight was associated with higher levels of medication adherence. Cannabis abuse/dependence during the acute phase of the patient's condition, work impairment, and higher levels of hallucination/delusion at baseline were associated with lower levels of medication adherence.	Fair

For peer review only

Unauthorised Leave

Author, year, country	Study design	Setting	Participants	Aims/ objectives related to patient safety	Outcomes related to patient safety	Study quality
Algase, et al. (2010) USA	Observational, questionnaire/ Quantitative	Long-term care units (LTCs) with dementia-specific units (N=28). These included nursing homes (n=22) and assisted living facilities (n=6)	Residents with dementia (n=122); 77% female; mean age 83.7 (range 68-102)	To investigate the influence of the physical environment on wandering behaviour	80% of wandering occurred in the residents' room, the community day room, the hall, or the eating halls. Location, light and sound impacted upon wandering behaviour. Odds ratio was highest for proximity to people (odds [OR] = 1.87).	Good
Bowers, Alexander & Gaskell (2003) UK	Controlled trial/ Quantitative	Acute psychiatric wards (n=5) in a hospital	All staff and patients within the units	To evaluate an intervention to reduce absconding by patients from acute psychiatric wards	Absconding fell by 25% during the intervention. Severe violent incidents increased significantly during the intervention ($\chi 2=7.74$, $P=0.005$). However, wards that saw a decrease in absconding, there was no change in the frequency of violent incidents, and there were significant decreases in door locking.	Good
Colombo, et al. (2001) Italy	Questionnaire/ Quantitative	An SCU	Inpatients (n=132). 100% female. Mean age 78.6±9.4	To develop a profile of female wanderers with dementia within an SCU	Wandering occurred in 51% of participants. Wanderers were found to fall 3 times as much as non-wanderers over 3 months (1.6 and 0.6 falls respectively).	Poor
Grotto et al. (2015) Australia	Interpretative inquiry/ Qualitative	Inpatient mental health units (n=3)	Mental health nurses (n=11); 55% female; age range (35-60)	To explore nurses' perceptions of assessment and management practices inpatient absconding	Clinical judgement influenced absconding assessment including historic markers of absconding. Nurses deemed risk assessment methods to be inadequate. There was no standardised management for dealing with absconding patients.	Fair
Hunt et al. (2010) UK	Descriptive/ Quantitative	Psychiatric inpatient wards	Inpatient suicides away from ward (n=469); 66% male	To describe inpatient suicides by individuals who had absconded and to compare against those on agreed leave	Absconders were significantly more likely to die by jumping than those on agreed leave (49% vs 30%, $p< 0.001$ respectively). Absconders were also more likely to die within the first week of admission (19% vs 8%, $p< 0.001$) and to be non-compliant with medication (25% vs 13%, $p< 0.001$).	Fair

For peer review only

					Factors that could have reduced the likelihood of suicide were suggested: more patient supervision, better treatment compliance, and increased staff numbers, communication and training.	
Meehan, Morrison & McDougall (1999) Australia	Semi-structured interviews/ Quantitative	Acute psychiatric unit in a general hospital campus	Inpatients (n=14); 36% female; mean age 37 (range 19-58 years)	To explore absconding behaviour from the patient perspective	Six issues were identified regarding absconding behaviour: boredom, lack of interesting activities, disturbed ward environment, the need for hospitalisation, issues at home, and perceived rewards from absconding. Situational and environmental factors were likely to increase the absconding risk.	Poor
Muir-Cochrane, et al. (2013) Australia	Semi-structured interviews/ Qualitative	An acute psychiatric unit	Mental health patients (n=12). 33% male	To explore the experiences of people who had been held involuntarily in an inpatient psychiatric unit and who had absconded or attempted to abscond	The inpatient unit is perceived as a safe or unsafe place, depending on physical, individual, social, and symbolic aspects of the unit. Absconding occurred when the unit was perceived as unsafe. Factors that would decrease the likelihood of absconding were identified: a therapeutic relationship with staff, familiarity with the unit, a comfortable environment, and positive interactions with peers.	Fair
Nijman, et al. (2011) UK	Questionnaire/ Quantitative	Acute psychiatric wards (n=133)	A qualified nurse	To investigate the prevalence of door locking and the use of other exit security measures on psychiatric wards, as well as investigating relationships between locking exit doors and absconding	There was no statistically significant relationship between exit security measures and absconding rates. However, there was a reduction of approximately 30% in absconding rates when the ward door was permanently locked compared to when doors were open.	Fair

For peer review only

Nurjannah, FitzGerald, & Foster (2009) Indonesia	Semi-structured interviews/ Qualitative	A psychiatric hospital	Patients (n=16). 94% male; mean age 33, range 13-65	To provide a rich description of the experience of patients related to incidents of absconding in a psychiatric setting	Three themes were highlighted: the call to home, hopes and realities, and us and them. The first theme concerned patients' need to connect with others and to feel safe. The second theme concerned patients' wishes to feel happy, which were often not compatible with realities of life at home or in the ward. The final theme concerned the competing interests and different opinions of patients regarding others, such as hospital staff and family.	Fair
Simpson, et al. (2015) Canada	Intervention design/ Quantitative	Forensic inpatient units (n=8) within a psychiatric hospital	Inpatients (n=188)	To investigate the impact of a new policy designed to reduce incidents of absconding in a forensic setting	The absconding rate decreased from 17.8% pre-implementation, to 13.8% during implementation, and further to 12.0% post-implementation. The most common reason for absconding was boredom/frustration.	Good

For peer review only

Clinical Decision Making

Author, year, country	Study design	Setting	Participants	Aims/ objectives related to patient safety	Outcomes related to patient safety	Study quality
Brown & Rakow (2015) UK	Questionnaire/ Quantitative	Psychiatric hospitals	Clinicians with an active role in violence risk assessment (n=9)	To explore clinicians' use of static and dynamic cues when assessing risk in psychiatric inpatients	Clinicians mostly used history of recent violence cues in assessments of in-hospital risk. Clinicians mostly used in-hospital frequency and severity of violence cues when assessing the risk posed by the patient if they remained in the current hospital.	Fair
Fuller & Cowan (1999) UK	Natural experiment/ Quantitative	Inpatient units (n=2) within a forensic hospital	Patients (n=75). 87% male. Modal age group 20-29	To assess the accuracy of staff predictions of patient clinical risk in forensic settings	The most common risk event was acting out against fellow patients (25% of risk events). Multidisciplinary clinical judgement predicted a variety of risk events at a higher than chance level. The risk event with the highest AUC statistic was prediction of serious incidents involving staff (AUC=0.856, p=≤ 0.0001).	Fair
Koukia , Madianos & Katostaras (2009) Greece	Cohort design/ Qualitative	Psychiatric hospital	Nurses (n=103); 57% female; mean age 36.3 [7.7]	To explore the on-the-spot decision making process of nurses when faced with a critical incident	When violent incidents occur, physical restraints were frequently used. However, reassurance and support were common interventions when a critical incident occurred. Nurses wanted more skills training and higher autonomy.	Fair
Lindsey (2009) USA	Correlational descriptive design/ Quantitative	Psychiatric hospital	Nurses (n=30); 87% female	To explore nurses' decisions to restrain psychiatric inpatients, and associations with work empowerment and individual patient/staff characteristics	When needed nurses chose high frequency medication and did not display a standardised decision making process in their restraint use.	Fair

For peer review only

Mann-Poll et al. (2011) Netherlands	Vignette and questionnaire/ Quantitative	Inpatient wards (n=4)	Staff (n=82)	To identify factors that contribute to decision making in relation to seclusion	The factors contributing to decisions to seclude patients came mostly from rater characteristics (e.g. type of care received from staff, frequency of participation in seclusion, experience of using seclusion as an intervention) and vignette variables (e.g. how approachable patient seemed, dangerousness and availability of rooms).	Fair
Marangos-Frost & Wells (2000) Canada	Ethnographic/ Qualitative	Psychiatric inpatient unit	Staff (n=6)	To understand nurses' decisions to restrain patients and feelings about those decisions	There were four main themes: framing the situation as a potential for immediate harm; unsuccessful exploration of alternative interventions; the conflicted nurse; and the conditions of restraint	Fair
Whaley (2001) USA	Cross-sectional/ Quantitative	A psychiatric hospital	Patients (n=118), age range 18-59	To evaluate agreement between clinical diagnoses and research diagnoses of schizophrenia for African American psychiatric patients. To explore the relationship of cultural mistrust with any disagreement	Levels of agreement for diagnoses was poor in 5 out of 6 comparisons. There were, however, significantly more cases using clinical diagnoses than other methods. Level of cultural mistrust did not seem to predict the excess in clinical diagnoses of paranoid schizophrenia.	Fair
Whaley (2004) USA	Cross-sectional/ Quantitative	A psychiatric hospital	Patients (n=94)	To explore cultural bias and diagnoses of schizophrenia for African American psychiatric patients	Highest rates of agreement were found between clinical and best estimate diagnoses of schizophrenia (kappa=.77, p< .01). Patients' cultural mistrust was positively associated with the number of clinical diagnoses of paranoid schizophrenia, b=.364, se=.123, p < .01, and best estimate diagnoses of paranoid schizophrenia, b=	Fair

For peer review only

.314, se= .128, $p < .05$. This implies a bias towards diagnosing cultural attitudes as pathology in African American psychiatric patients.

For peer review only

Online supplement 5 – Forrest plots

Prevalence of physical aggression

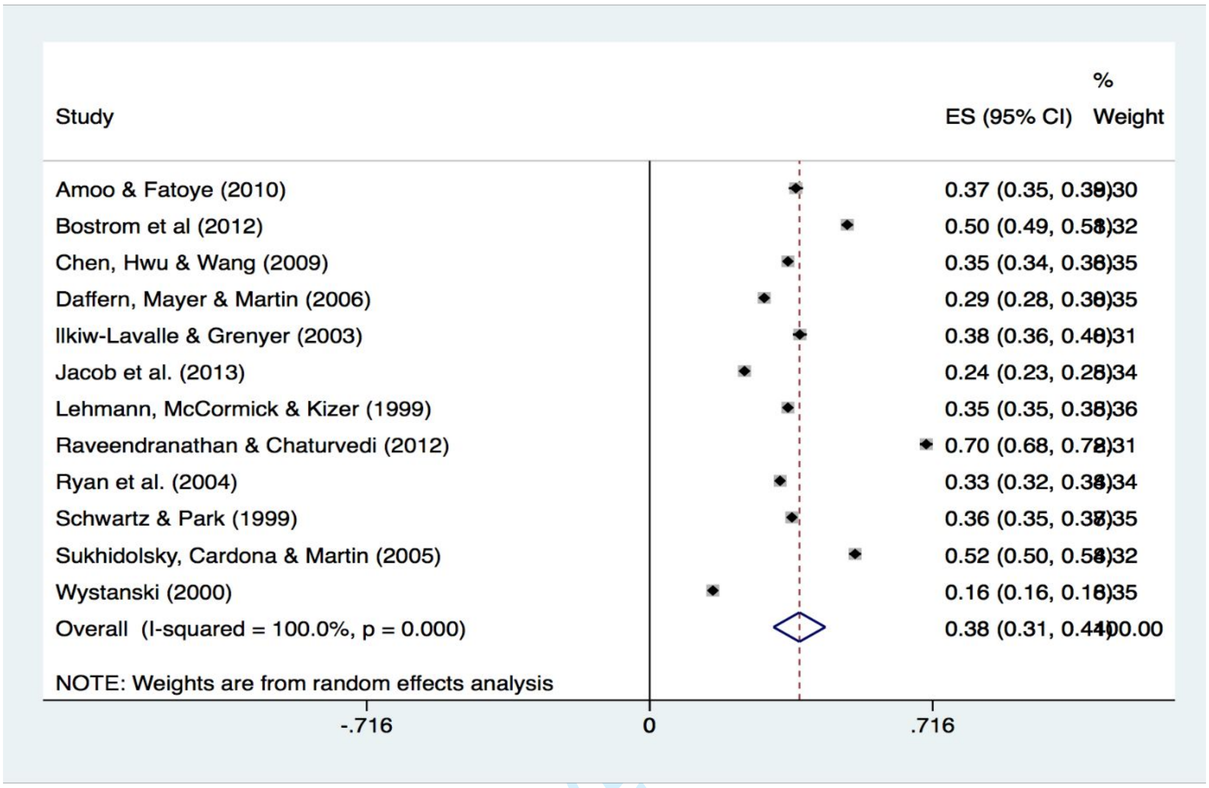

Prevalence of verbal aggression

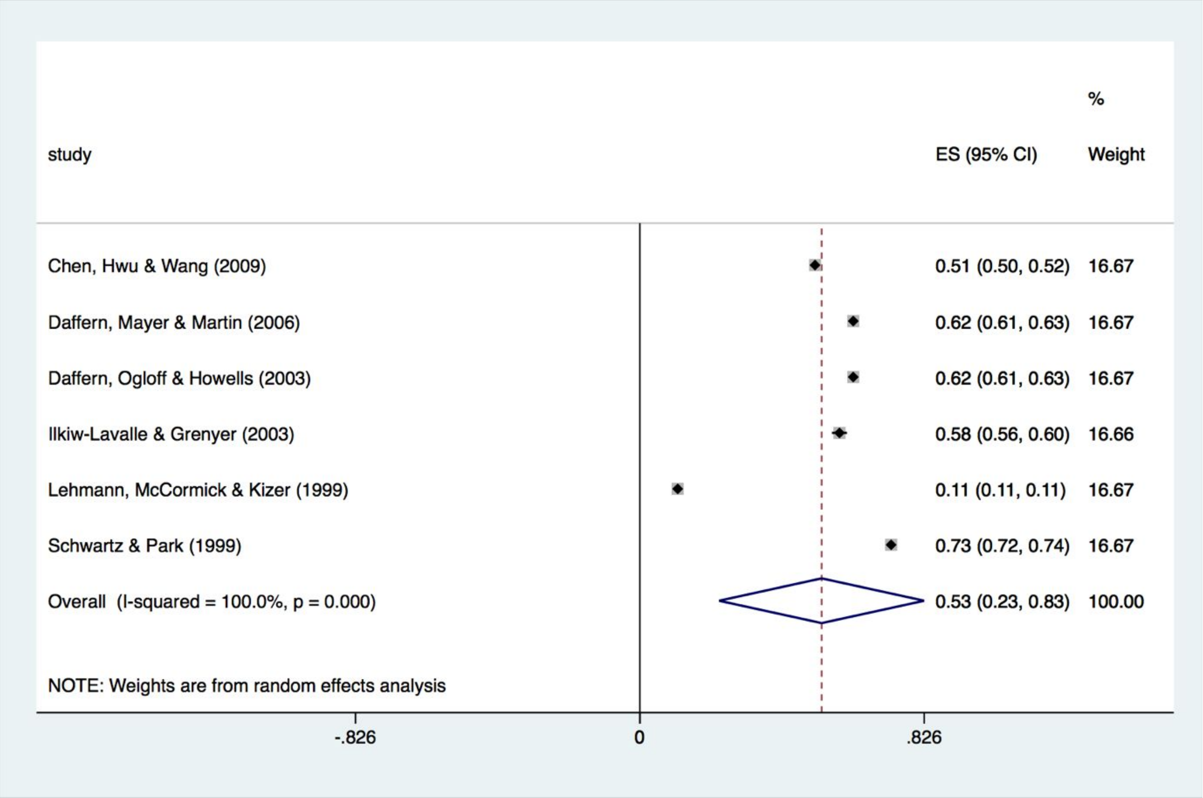

Use of coercive interventions

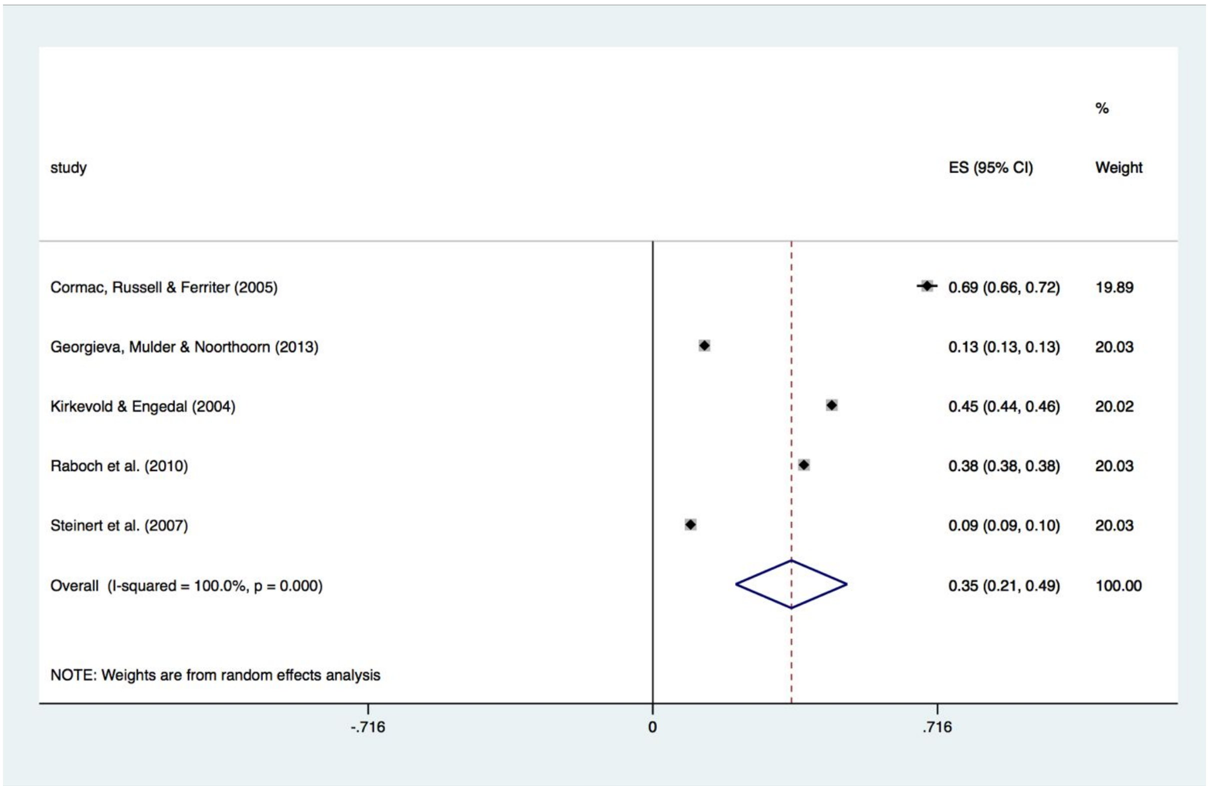

Prevalence of wandering behaviour

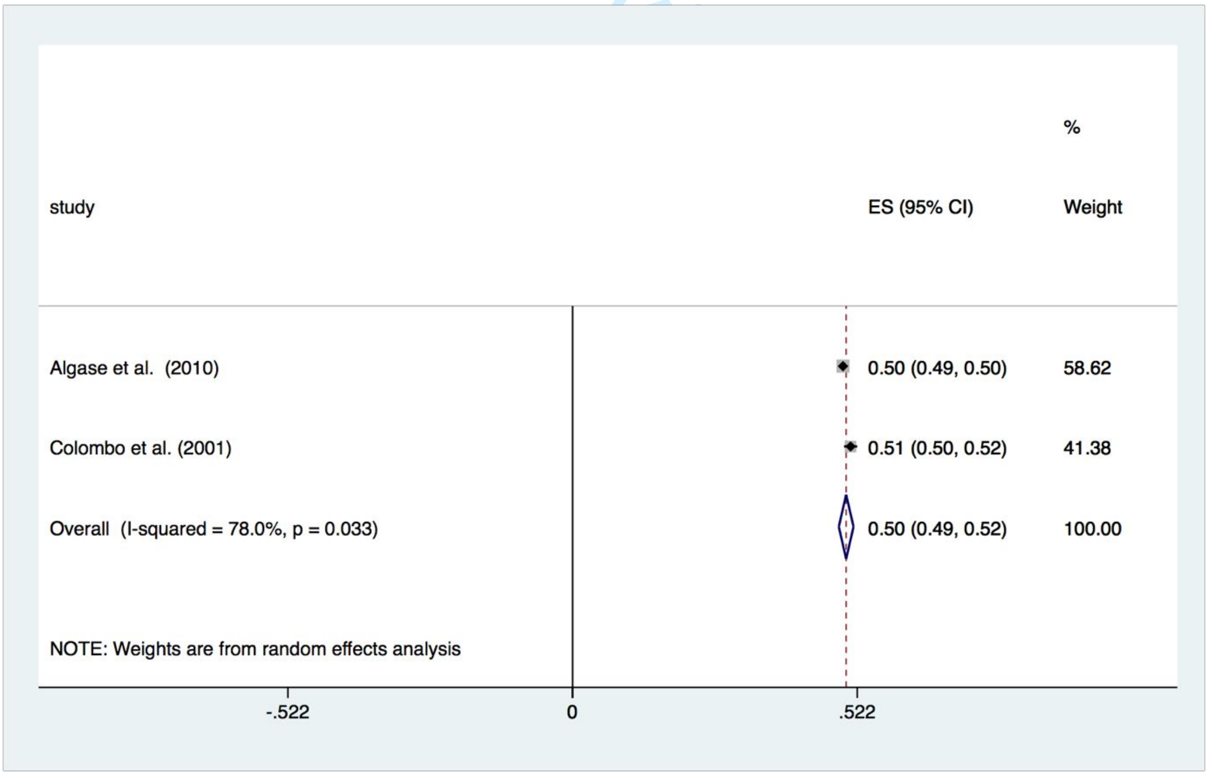

PRISMA 2009 Checklist

Section/topic	#	Checklist item	Reported on page #
**TITLE**			
Title	1	Identify the report as a systematic review, meta-analysis, or both.	1
**ABSTRACT**			
Structured summary	2	Provide a structured summary including, as applicable: background; objectives; data sources; study eligibility criteria, participants, and interventions; study appraisal and synthesis methods; results; limitations; conclusions and implications of key findings; systematic review registration number.	2
**INTRODUCTION**			
Rationale	3	Describe the rationale for the review in the context of what is already known.	3
Objectives	4	Provide an explicit statement of questions being addressed with reference to participants, interventions, comparisons, outcomes, and study design (PICOS).	3
**METHODS**			
Protocol and registration	5	Indicate if a review protocol exists, if and where it can be accessed (e.g., Web address), and, if available, provide registration information including registration number.	3
Eligibility criteria	6	Specify study characteristics (e.g., PICOS, length of follow-up) and report characteristics (e.g., years considered, language, publication status) used as criteria for eligibility, giving rationale.	4,5
Information sources	7	Describe all information sources (e.g., databases with dates of coverage, contact with study authors to identify additional studies) in the search and date last searched.	4
Search	8	Present full electronic search strategy for at least one database, including any limits used, such that it could be repeated.	Online Supplement 2
Study selection	9	State the process for selecting studies (i.e., screening, eligibility, included in systematic review, and, if applicable, included in the meta-analysis).	4
Data collection process	10	Describe method of data extraction from reports (e.g., piloted forms, independently, in duplicate) and any processes for obtaining and confirming data from investigators.	5
Data items	11	List and define all variables for which data were sought (e.g., PICOS, funding sources) and any assumptions and simplifications made.	4,5
Risk of bias in individual studies	12	Describe methods used for assessing risk of bias of individual studies (including specification of whether this was done at the study or outcome level), and how this information is to be used in any data synthesis.	n/a
Summary measures	13	State the principal summary measures (e.g., risk ratio, difference in means).	5, Online Supplement

For peer review only - http://bmjopen.bmj.com/site/about/guidelines.xhtml

PRISMA 2009 Checklist

			3
Synthesis of results	14	Describe the methods of handling data and combining results of studies, if done, including measures of consistency (e.g., I^2) for each meta-analysis.	5,6

Section/topic	#	Checklist item	Reported on page #
Risk of bias across studies	15	Specify any assessment of risk of bias that may affect the cumulative evidence (e.g., publication bias, selective reporting within studies).	n/a
Additional analyses	16	Describe methods of additional analyses (e.g., sensitivity or subgroup analyses, meta-regression), if done, indicating which were pre-specified.	n/a
**RESULTS**			
Study selection	17	Give numbers of studies screened, assessed for eligibility, and included in the review, with reasons for exclusions at each stage, ideally with a flow diagram.	6
Study characteristics	18	For each study, present characteristics for which data were extracted (e.g., study size, PICOS, follow-up period) and provide the citations.	6-10
Risk of bias within studies	19	Present data on risk of bias of each study and, if available, any outcome level assessment (see item 12).	n/a
Results of individual studies	20	For all outcomes considered (benefits or harms), present, for each study: (a) simple summary data for each intervention group (b) effect estimates and confidence intervals, ideally with a forest plot.	7-10
Synthesis of results	21	Present results of each meta-analysis done, including confidence intervals and measures of consistency.	6-10
Risk of bias across studies	22	Present results of any assessment of risk of bias across studies (see Item 15).	n/a
Additional analysis	23	Give results of additional analyses, if done (e.g., sensitivity or subgroup analyses, meta-regression [see Item 16]).	n/a
**DISCUSSION**			
Summary of evidence	24	Summarize the main findings including the strength of evidence for each main outcome; consider their relevance to key groups (e.g., healthcare providers, users, and policy makers).	10-12
Limitations	25	Discuss limitations at study and outcome level (e.g., risk of bias), and at review-level (e.g., incomplete retrieval of identified research, reporting bias).	12,13
Conclusions	26	Provide a general interpretation of the results in the context of other evidence, and implications for future research.	14
**FUNDING**			
Funding	27	Describe sources of funding for the systematic review and other support (e.g., supply of data); role of funders for the systematic review.	1,2

For peer review only - http://bmjopen.bmj.com/site/about/guidelines.xhtml

From: Moher D, Liberati A, Tetzlaff J, Altman DG, The PRISMA Group (2009). Preferred Reporting Items for Systematic Reviews and Meta-Analyses: The PRISMA Statement. PLoS Med 6(7): e1000097.

PRISMA 2009 Checklist

doi:10.1371/journal.pmed1000097 For more information, visit: www.prisma-statement.org.

For peer review only

For peer review only - http://bmjopen.bmj.com/site/about/guidelines.xhtml

BMJ Open

Patient safety in inpatient mental health settings: a systematic review

Journal:	*BMJ Open*
Manuscript ID	bmjopen-2019-030230.R1
Article Type:	Original research
Date Submitted by the Author:	11-Oct-2019
Complete List of Authors:	Thibaut, Bethan; Imperial College London<br>Dewa, Lindsay; Imperial College London<br>Ramtale, Sonny; Imperial College London<br>D'Lima, Danielle; University College London<br>Adam, Sheila; Imperial College London<br>Ashrafian, Hutan; Imperial College London<br>Darzi, Ara; Imperial College London<br>Archer, Stephanie; Imperial College London; University of Cambridge
**Primary Subject Heading**:	Mental health
Secondary Subject Heading:	Health policy
Keywords:	Patient safety, MENTAL HEALTH, Inpatient settings, Systematic Review

SCHOLARONE™
Manuscripts

Patient safety in inpatient mental health settings: a systematic review

Authors: Bethan Thibaut[1,2*], Lindsay H. Dewa[1,2*], Sonny Christian Ramtale[1], Danielle D'Lima[3], Sheila Adam[1], Hutan Ashrafian[4], Ara Darzi[1,4] and Stephanie Archer[1]

[1]NIHR Imperial Patient Safety Translational Research Centre, Imperial College London, London, UK

[2] School of Public Health, Imperial College London, London, UK

[3] Centre for Behaviour Change, Department of Clinical, Educational and Health Psychology, London, UK

[4] Department of Surgery and Cancer, St Mary's Hospital, Imperial College London, London, UK

*Bethan Thibaut and Dr Lindsay Dewa should be considered joint first author

Corresponding author: Dr Stephanie Archer, NIHR Patient Safety Translational Research Centre, Imperial College London, London, UK; stephanie.archer@imperial.ac.uk

For peer review only

Abstract

Objectives: Patients in inpatient mental health settings face similar risks to those in other areas of health care (e.g. medication errors). In addition, some unsafe behaviours associated with serious mental health problems (e.g. self-harm), and the measures taken to address these (e.g. restraint), may result in further risks to patient safety. The objective of this review is to identify and synthesise the literature on patient safety within inpatient mental health settings using robust systematic methodology.

Design: Systematic review and meta-synthesis. Embase, CINAHL, HMIC, MEDLINE, PsycINFO and Web of Science were systematically searched from 1999 to 2019. Search terms were related to “mental health”, “patient safety”, “inpatient setting” and “research”. Study quality was assessed using the Hawker checklist. Data was extracted and grouped based on study focus and outcome. Safety incidents were meta-analysed where possible using a random effects model.

Results: Of the 57,637 article titles and abstracts, 364 met inclusion criteria. Included publications came from 31 countries and included data from over 150,000 participants. Study quality varied and statistical heterogeneity was high. Ten research categories were identified: interpersonal violence, coercive interventions, safety culture, harm to self, safety of the physical environment, medication safety, unauthorised leave, clinical decision making, falls and infection prevention and control.

Conclusions: Patient safety in inpatient mental health settings is under researched in comparison to other non-mental health inpatient settings. Findings demonstrate that inpatient mental health settings pose unique challenges for patient safety which require investment in research, policy development, and translation into clinical practice.

PROSPERO registration: CRD42016034057

Key words

Patient safety, Mental health, Inpatient settings, Review

For peer review only

Strengths and limitations of this study

This is the first review to examine patient safety within inpatient mental health settings that uses robust systematic methodology.

The use of a robust patient safety taxonomy provides a comprehensive list of all incident types and resulted in a wide coverage of publications in terms of setting, country and population.

This review only included peer reviewed studies with primary data.

The last systematic literature search was conducted on 27th June 2019, meaning that literature published since this date will not have been included.

For peer review only

For peer review only - http://bmjopen.bmj.com/site/about/guidelines.xhtml

Introduction

Patient safety has been defined as the “avoidance, prevention and amelioration of adverse outcomes or injuries stemming from the process of healthcare” [1]. Those receiving care in inpatient mental health settings face similar risks to patients in other areas of health care (e.g. medication errors). In addition, some of the unsafe behaviours associated with serious mental health problems (e.g. self-harm), and the measures taken to address these (e.g. restraint), may result in further risks to patient safety [2-6]. There may also be a tension between maximising patient safety and maintaining patient autonomy. Inpatient services will often include patients who are experiencing high levels of mental distress and are therefore at greatest risk.

Whilst mental health research has focused on components of quality of care, published research lacks focus on the science of patient safety [7-9]; the stigma and discrimination associated with mental health problems may contribute to this relative neglect [7]. Only two reviews have examined patient safety in a mental health context and described factors that influence patient safety [7, 10]. These reviews highlighted the complexity of patient safety in mental health, including the importance of wider organisational safety culture. Whilst these reviews offer important insights into this complex topic, only a small number of specific patient safety incidents and concepts were examined. As such, the current breadth and depth of patient safety research in inpatient mental health settings is unknown.

The review presented here is exploratory in nature; building on previous reviews, we aimed to report an overview of the existing research base on patient safety in inpatient mental health settings. We also aimed to critically reflect on quality and methods used in included studies in the field [11]. In addition to our original protocol [11], we aimed to collate, describe and construct the main research categories, allowing for an easily accessible reference index.

Search strategy and selection criteria

A systematic search was developed and deployed in relation to the Preferred Reporting Items for Systematic Reviews and Meta-Analyses (PRISMA) guidelines [12]. The protocol for this systematic review was registered with PROSPERO (2016: CRD42016034057) and has been published elsewhere [11].

Six databases were searched: Embase, Cumulative Index to Nursing and Allied Health Literature (CINAHL), Health Management Information Consortium (HMIC), MEDLINE, PsycINFO and Web of Science. The search was originally conducted on the 5th April 2016

For peer review only - http://bmjopen.bmj.com/site/about/guidelines.xhtml

and then updated on the 27th June 2019 using a comprehensive list of search terms (n=343) related to "mental health" (n=73), "patient safety" (n=206), "inpatient setting" (n=13) and "research" (n=51); see online supplement 1 and 2 for full search criteria and terms. The search terms included in the 'patient safety' facet were based on the National Reporting and Learning System (NRLS) taxonomy for England and Wales [13] to ensure all incident types were identified in the search. A Google Scholar search using the main search terms was also conducted; it was originally anticipated that the first 20 pages of Google scholar would need to be screened against criteria [11] but screening stopped at five pages as no new publications were retrieved. Similarly, we had anticipated hand-searching references of all included papers within the review. However, due to the large amount of literature included in the review, only the reference lists of the two existing systematic reviews were searched for additional references.

Five reviewers (BT, CR, LD, DD and SAr) screened all titles against the inclusion and exclusion criteria, with 10% independently screened by a second reviewer (split equally between BT, CR, LD, DD and SAr). Full definitions and descriptions of these criteria can be found in online supplement 1 and the protocol published elsewhere[11]. Inclusion and exclusion criteria were developed over several iterative rounds amongst the research team to ensure consistency between reviewers (online supplement 2). Any disagreements between reviewers were resolved through discussion and an overall consensus was obtained. Agreement between reviewers was calculated using Cohen's kappa [14], which is a widely accepted measure of interrater reliability [15, 16]. Full text papers were assessed for inclusion by two reviewers from the research team (BT and one other from CR, LD and SAr); a third reviewer (DD) was consulted if necessary.

Inclusion criteria:

- Population: Mental health inpatients
- Intervention/outcomes: Patient safety outcomes
- Setting: Inpatient setting
- Comparators: No restriction
- General inclusion criteria: Empirical peer reviewed studies with a clear aim or research question, that used primary data and written up in the English language between January 1st 1999 and June 27th 2019 (in line with the publication of the Institute of Medicine's report "To Err is Human: Building a Safer Health System")[17]

Exclusion criteria:

- Population: Centres on physical healthcare patients

For peer review only - http://bmjopen.bmj.com/site/about/guidelines.xhtml

- Intervention/outcomes: Patient safety was not the central aim, research question or outcome; amalgamation of data from inpatient and outpatient settings (where inpatient sample cannot be separated out); primary care, outpatient mental health services, community or social care settings, and risk assessment tool reliability/validity checks
- Comparators: No restrictions
- General exclusion criteria: Secondary data, not in English language, protocols, editorials, commentaries/clinical case reviews/'snapshot' studies of a patient group, book chapters, conference abstracts, audits, dissertations, epidemiological studies and reviews.

Quality Assessment

Quality assessment was performed to give an overview of the methodological rigour of included studies and to support readers' interpretation of the literature. Publications were not excluded based on poor quality because the review was purposively exploratory and all-encompassing. Quality was assessed by four reviewers (BT, CR LD and SAr) using the tool derived by Hawker et al.[18], to allow appropriate assessment of the wide variety of studies included in this review. Hawker's checklist evaluates nine domains: 1) abstract/title; 2) introduction and aims; 3) method and data; 4) sampling; 5) data analysis; 6) ethics and bias; 7) results; 8) transferability and generalizability; and 9) implications and usefulness. For each study the nine domains were assessed using one of four quality categories: very poor (10 points), poor (20 points), fair (30 points) and good (40 points). The scores for each study were then summed and divided by nine to get an average score.

Data extraction

Data was extracted by five reviewers (BT, CR, LD, DD and SAr) using a standardised form that included study design information, participant characteristics, intervention description and patient safety outcomes. Extractions were compared within the research team to ensure reliability. Only published data was extracted; authors were contacted only for confirmation or information clarity. If the contact attempt was unsuccessful, the article was assessed in its current form.

Data synthesis

Studies were grouped into research categories through group consensus. Firstly, four research team members (BT, CR, LD and SAr) individually re-read the included full-text publications and assigned each one based on the main topic area (e.g. aggression). Secondly, each assigned topic area was checked by another team member to ensure

For peer review only - http://bmjopen.bmj.com/site/about/guidelines.xhtml

reliability. Thirdly, topic areas were grouped into broader research categories (e.g. interpersonal violence) that best described the patient safety focus for easier navigation of the vast body of literature. Finally, these categories and the related subcategories (initially called topic areas) from the previous stage were finalised after group discussion and consensus was reached. This was to ensure mutual exclusivity and appropriate definition (Table 1 and online supplement 3). Where data allowed, meta-analysis was performed applying a random-effects model, specifically calculating pooled prevalence considering both between-study and within-study variances that contributed to study weighting. Pooled values and 95%CIs were computed and represented on forest plots. Statistical heterogeneity was determined by the I2 statistic; where <30% is low, 30-60% is moderate and >60% is high. Analyses were performed using Stata version 15 (StataCorp LP, College Station, TX).

Patient and Public Involvement

Patients and the public were not involved in this study.

Results

The search resulted in 79,672 records (Figure 1) and reduced to 57,637 after de-duplication. Title and abstracts were screened and excluded if they did not satisfy inclusion criteria (BT, CR, LD, DD and SAr). Ten percent were then screened (n=5,763) by a second independent reviewer (split equally between BT, CR, LD, DD and SAr), in line with guidance on improving decision making by including more than one person in this process [19]; good agreement was found between pairs of reviewers (κ=0.72). A total of 4,758 publications were subjected to full-text review (BT, CR, LD and SAr). Two reviewers independently screened the full text articles against inclusion criteria (BT, CR, LD, and SAr). The third reviewer (DD) was consulted 59 times. Substantial agreement was reached (κ=0.64). From the full text review, 4,394 publications were excluded. Three hundred and sixty-four publications met the inclusion criteria and data was extracted (online supplement 4).

<Insert Figure 1 here>

Study characteristics

Table 1 provides a study characteristics overview. The publications spanned five continents and 31 countries. The three countries contributing the greatest number of studies were the UK (n=102), the USA (n=55) and Australia (n=32). The included studies collected data from over 150,000 participants. Studies included staff (n=165; 45%), patients (n=120; 33%) and a mixture of staff, patients and/or carers (n=77; 21%). Only one study focused solely on patient family members (<1%). Most studies were quantitative in nature (n=192; 53%), just

For peer review only - http://bmjopen.bmj.com/site/about/guidelines.xhtml

over a third were qualitative (n=133; 37%) and a small proportion used mixed methodology (n=39; 11%). Studies were conducted in a variety of settings comprising: psychiatric inpatient wards/facilities (n=266;73%), forensic inpatient facilities (n=50; 14%), long-term care/nursing homes (n=25; 7%), mixed inpatient settings (n=20; 5%), a learning disability unit (n=1; <1%), a health board (n=1; <1%) and a specialised research unit (n=1; <1%). More information is included about the study designs used in the included papers in online supplement 2.

Quality assessment

Most research was assessed as 'Fair' quality (n=251; 69%); 86 (24%) papers were assessed as 'Good' quality, and 26 (7%) were assessed as 'Poor' quality. None were assessed as 'Very Poor' quality. Studies rated as 'Poor' mainly did not discuss ethical considerations, potential biases or give sample or setting characteristics. For example, they did not consider recruitment strategies, sample demographics or structure of the research settings. All 'Good' studies provided setting and sampling information to allow for replicability. In addition, 'Good' studies provided detail on data analysis justification, more thorough literature reviews to place the study in context and had clear research aims/objectives. Online supplement 5 includes a table showing the breakdown of the quality domain scores for each paper.

Synthesis

Ten research categories were identified: interpersonal violence, coercive interventions, safety culture, harm to self, safety of the physical environment, medication safety, unauthorised leave, clinical decision making, falls and Infection prevention and control. Within these categories 46 subcategories were identified (see Table 1).

For peer review only

For peer review only - http://bmjopen.bmj.com/site/about/guidelines.xhtml

Table 1: Overview of study characteristic identified within each category

Category	Sub category	Category definition	Number of studies	Countries	Number of studies using staff participants	Number of studies using patient participants	Total number of participants	Settings (number of studies conducted in each setting)
Interpersonal Violence	Aggression<br>Violence<br>Challenging behaviour<br>Violence and aggression<br>Critical incidents<br>Conflict<br>Sexual Assault<br>Agitation	Behaviours or events that are considered hostile with the intent to cause harm, including violence, aggression and conflicts. This also encompasses sudden emergency incidents that require management.	115	UK-31<br>USA-20<br>Australia-9<br>Canada-7<br>Netherlands-6<br>Sweden-7<br>Taiwan-4<br>South Africa-2<br>Switzerland-2<br>India-3<br>Italy-2<br>Turkey-3<br>Europe-2<br>New	52<br>22 mixed	39<br>22 mixed<br>1 family members of patients<br>1 N/A	20,066 (excl. missing data)	Psychiatric inpatient wards/facilities-73<br>Forensic inpatient facilities- 22<br>Long-term care/Nursing homes- 13<br>Specialised research unit - 1<br>Mixed- 6

For peer review only - http://bmjopen.bmj.com/site/about/guidelines.xhtml

				Zealand-1<br>South Korea-1<br>Finland-3<br>Greece-1<br>Spain-1<br>Hong Kong-1<br>Israel-2<br>Nigeria-1<br>Norway-2<br>Denmark-1<br>Japan-1<br>Germany-1<br>Slovakia-1				
Coercive Interventions	Restraint<br><br>Seclusion<br><br>Attitudes to coercion	Techniques for managing patient behaviour that are applied without consent, for the safety of	99	UK-31<br>Finland-7<br>USA-8<br>Netherlands-5<br>Australia-5<br>Canada-7	36<br>29 mixed	34<br>29 mixed	59,732 (excl. missing data)	Psychiatric inpatient wards/facilities-74<br>Forensic inpatient facilities- 13

For peer review only - http://bmjopen.bmj.com/site/about/guidelines.xhtml

	Seclusion and restraint<br>Containment<br>Process of coercion<br>Alternative interventions<br>Shielding<br>Conflict<br>Personal factors	the patient and others. These include seclusion, restraint and containment.		Norway-4<br>Germany-2<br>Sweden-3<br>Japan-2<br>Mixed-5<br>New Zealand-2<br>Europe-1<br>China-1<br>Switzerland-3<br>South Korea-1<br>India-2<br>Brazil-1<br>Denmark-1				Long-term care/Nursing homes- 3<br>Mixed- 8<br>Health board-1
Safety Culture	Process<br>Culture<br>Policy<br>Building	The organisational attitudes, beliefs and values concerning safet y. This	49	UK-12<br>Australia-10<br>USA-5<br>Sweden-4<br>Finland-4<br>Canada-2	33<br>13 mixed	3<br>13 mixed	59,420 (excl. missing data)	Psychiatric inpatient wards/facilities-36<br>Forensic inpatient

For peer review only

	therapeutic relationships<br><br>Patient/family engagement	encompasses the policies and procedures within the healthcare organisation in relation to safety.		Ireland-2<br>Netherlands-1<br>Greece-1<br>Italy-1<br>Germany-1<br>Belgium-2<br>Taiwan-1<br>Europe-1<br>Iran-2				facilities- 8<br>Long-term care/Nursing homes- 1<br>Mixed- 4
Harm to self	Self-harm<br><br>Suicidal behaviour<br><br>Self-neglect	The ways in which the healthcare system attempts to prevent, mitigate or manage deliberate behaviours displayed by patients that are intended to	36	USA-11<br>UK-8<br>Ireland-3<br>Norway-4<br>Netherlands-2<br>Sweden-2<br>Taiwan-2<br>Australia-1<br>Japan-2<br>Belgium-1	16<br>3 mixed	17<br>3 mixed	3,631 (excl. missing data)	Psychiatric inpatient wards/facilities-29<br>Forensic inpatient facilities- 3<br>Long-term care/Nursing homes- 2<br>Learning disability

For peer review only - http://bmjopen.bmj.com/site/about/guidelines.xhtml

		cause harm or death to themselves.						homes-1<br>Mixed- 1
Safety of the physical environment	Security<br>Environmental design<br>Transitions of care<br>Patient distribution<br>Staffing<br>Ligatures	The factors related to the physical environment of the healthcare setting that could impact upon safety. This includes ligature points, staffing, security (door locking) and patient distribution.	21	UK-6<br>Netherlands-3<br>USA-4<br>Australia-4<br>Germany-2<br>Mixed-1<br>Sweden-1	6<br>8 mixed	7<br>8 mixed	3,140 (excl. missing data)	Psychiatric inpatient wards/facilities-17<br>Forensic inpatient facilities- 1<br>Long-term care/Nursing homes- 3
Medication Safety	Adverse events<br>Medication	Mistakes made at any stage of the medication u	17	UK-7<br>Turkey-1<br>Spain-2	9<br>1 mixed	7<br>1 mixed	2,396 (excl. missing data)	Psychiatric inpatient wards/facilities-

For peer review only

	administration<br><br>Medication management<br><br>Medication dispensing<br><br>Adherence<br><br>Substance use	se process, from preparation, to administration and recording. This includes adverse drug events (ADEs, or injuries that are the result of a drug-related intervention) and issues surrounding drug/alcohol use.		Netherlands-1<br>Croatia-1<br>Germany-1<br>Denmark-1<br>Canada-2<br>Mixed-1				13<br>Forensic inpatient facilities- 2<br>Long-term care/Nursing homes- 1<br>Mixed-1
Unauthorised Leave	Absconding<br><br>Wandering	The act of a patient leaving the healthcare setting without the knowledge or consent of staff/carers. This	11	UK-4<br>Australia-3<br>USA-1<br>Canada-1<br>Italy-1<br>Indonesia-1	3<br>1 mixed	7<br>1 mixed	978 (excl. missing data)	Psychiatric inpatient wards/facilities-10<br>Long-term care/Nursing homes- 1

For peer review only - http://bmjopen.bmj.com/site/about/guidelines.xhtml

		can be either with (absconding) or without intent (wandering) on the part of the patient.						
Clinical decision making	Incident management<br><br>Risk assessment<br><br>Diagnosis	Incorrect diagnoses, risk assessments and other decision making processes of healthcare staff that impact upon the safety of a patient.	9	USA-3<br>UK-3<br>Canada-1<br>Greece-1<br>Netherlands-1	6	3	529	Psychiatric inpatient wards/facilities-8<br>Forensic inpatient facilities- 1
Falls	Falls<br><br>Injuries	Falling events that lead to the unintentional harm of an individual. This	6	USA-3<br>Sweden-2<br>Israel-1	3	3	180 (excl. missing data)	Psychiatric inpatient wards/facilities-5<br>Long-term

For peer review only - http://bmjopen.bmj.com/site/about/guidelines.xhtml

		includes trips and injuries such as fractures.						care/Nursing homes- 1
Infection prevention and control	Infection prevention and control	Preventing harm caused by infection to patients and health workers	1	Taiwan-1	1	0	13	Psychiatric inpatient wards/facilities- 1

For peer review only - http://bmjopen.bmj.com/site/about/guidelines.xhtml

Interpersonal violence

Interpersonal violence was the largest category (n=116; 32%). Studies were primarily concerned with the prevalence, management and prevention of violent and aggressive behaviours (n=75). The pooled prevalence for physical violence was 43.2% (95%CI 0.37-0.49) with high heterogeneity (I^2 100.0%) in 20 studies [20-39] (online supplement 6). The pooled prevalence for verbal aggression was 57.4% (95%CI 0.34-0.81) with a high heterogeneity (I^2 100.0%) in 10 studies [22-24, 26, 29, 33-36, 40] (online supplement 6).

One study examined the characteristics of aggressive incidents by ward type [41], and two studies identified correlates of violence [42, 43]. One study explored how patients describe their aggressive behaviours [44]. Twenty-four studies evaluated intervention effectiveness (e.g. staff training and medication use) to reduce violent and aggressive behaviours, with most finding significant improvements [45-65], two reporting negative outcomes [66, 67], and one reporting mixed findings [68]. The general management of violent and aggressive behaviours was explored in 15 studies [22, 25, 29, 30, 69-79]. Two studies explored the ways in which treatment can affect violence incidence [31, 80].

Twenty-seven studies explored violent and aggressive incident experiences in staff [81-96], patients [97-99], mixed groups [100-106] and patient family members [27]. Five studies explored the risk factors associated with verbal and physical aggression [35, 37, 107-109]. Three studies explored mental health nurses' perspectives on the response to violent situations in high secure environments: one on the psychological impact of physical assault on staff [110], one on making violence risk assessments in imminent violent situations [111] and one on the decline of incident reports [112]. One study explored the link between aggressive behaviour and levels of burnout in staff [113] and one study looked at the role of social support for staff following a violent incident [32].

Ten studies [114-123] examined challenging behaviour and techniques, such as de-escalation and communication strategies, that could be used to manage this; seven studies found techniques that were effective [114-120]. A further four studies investigated conflict behaviour management techniques employed by staff [124-126] and patients [127]; techniques used in the two intervention studies were effective in reducing conflict [126, 127]. Staff and patient attitudes towards critical incidents were the focus of four qualitative studies [128-131]; a further three studies focused on maintaining the psychological safety of patients who had experienced physical or sexual assault during an inpatient stay [132] and outside of healthcare [133, 134]. Finally, one study explored an acupressure intervention to reduce agitation, which was found to be effective [135].

For peer review only - http://bmjopen.bmj.com/site/about/guidelines.xhtml

For peer review only

Coercive interventions

Coercive interventions were the focus of 98 papers (27%). Most studies (n=42) reported on restraint and seclusion techniques. The pooled prevalence for coercive interventions was 47.8% (95%CI 0.38-0.57) with high heterogeneity (I^2 100.0%) in 12 studies [136-147] (online supplement 6).

Studies explored staff [148-157], patient [147, 158-165], and mixed groups' [166-173] views and experiences of seclusion and restraint. Nine studies focused on the processes surrounding seclusion and restraint [136, 137, 174-180]. A further 16 studies evaluated interventions to reduce seclusion and restraint, with 13 finding significant decreases in rates of use [146, 181-192], one reporting an increase [193] and one reporting increased levels of knowledge about the topic area [194]. Four studies examined prevalence, trends and preventative factors [138, 195-197]; one found that 45% of patients were subjected to restraint [138], and another found that restraint and seclusion declined over time [197]. One study explored the context in which seclusion and restraint had taken place [198]. Two studies found preventative factors of mechanical restraint to be staff education and increased patient involvement [195, 196]. The training of staff in techniques for seclusion and restraint were explored in two studies [199, 200] and one study examined adverse events resulting from restraint and seclusion [201]. Other studies explored staff and patient views of containment measures [202-205], Maori views of initiatives to reduce/prevent seclusion [206], the process of shielding[1] [207], conflict management [208] and alternative interventions [209].

Thirty-two studies focused on coercion; one study examined prevalence of coercive measures [141] and one study explored how the experience of staff might contribute to the use of restrictive practices [210]. The attitudes of staff [142, 144, 211-222], patients [145, 223-226], and mixed groups [143, 168, 227-230] towards coercion were explored in 25 studies, and five studies examined the process of coercive interventions [139, 140, 231, 232] and rules of engagement in caring for aggressive patients [233].

Safety culture

Safety culture included studies on process, culture and policy across 49 papers (13%). Eighteen studies concerned safety-related organisational processes. Eleven of these

---

[1] Segregation under staff supervision

For peer review only - http://bmjopen.bmj.com/site/about/guidelines.xhtml

investigated processes of treatment or care that healthcare staff undertake; processes included limit-setting and clothing restrictions [234-240], risk assessment [241-243] and nursing handover [244]. Two investigated errors and reporting [245, 246] and a further two studies explored staff and patient perceptions of safety when involved in treatment processes [247, 248]. Two studies focused on change implementation [249, 250]. One study focused on the role of training [251]. Safety culture was featured in 18 publications relating to the management of serious incidents [252-254], stress and burnout [255-257], staff [258] and patient perspectives of safety [259-263] and communication [264]; there were also three papers that explored safety culture more generally [265-267]. A further two evaluated the TeamSTEPPS (Team Strategies and Tools to Enhance Performance and Patient Safety) programme [268, 269] and both found significant clinical benefits in reducing seclusion and improving team functioning. One paper looked at the barriers and facilitators to implementing a Safewards intervention [270] . With regards to policy, eight studies concerned safety policies related to: observation [271, 272], risk assessment [273, 274], treatment [275], safeguarding [276], security [277] and ergonomic improvement [278]. Two papers focused on the role of patient and family engagement in safety [279, 280], and two papers focused on how to build better therapeutic relationships to improve patient safety [281, 282].

Harm to Self

Three subcategories centred on harmful behaviours: self-harm, suicidal behaviour and self-neglect (n=36; 10%). Half of the studies (n=18) focused on self-harm. One paper explored the prevalence of self-harm [283]. Two studies explored risk factors for self-harm which included use of psychotropic medication [284, 285]. Eight papers explored staff attitudes and experiences of managing self-harm [286-293], and three explored patient experiences [294-296]. Three intervention studies focused on training [297], therapy [298] and observation [299]; all reported a reduction in self-harm behaviours and a further intervention focusing on training for staff resulted in positive attitude towards self-harm patients, greater closeness and improved self-efficacy [300]. Of the 17 papers that centred on suicidal behaviours, five studies investigated the observance of risk factors [301-305] and three intervention studies found significant reductions in suicide-related behaviours and cognitions [306-308]. An additional eight papers explored staff [309-312], patient [313, 314], and both staff and patient [315, 316] views and attitudes towards suicidal behaviour. One study looked at the acceptability of an intervention to reduce suicide [317]. Finally, one study explored types of self-neglect behaviours in dementia patients, including functional difficulties, serious hygiene problems and safety risks [318].

Safety of the physical environment

For peer review only - http://bmjopen.bmj.com/site/about/guidelines.xhtml

The safety of the physical environment category included 21 papers (6%). Seven studies investigated security measures (e.g. door locking) [319-325]. Five studies investigated the effects of the physical environmental design on the safety of treatment settings [326-330]. Three studies focused on transitions of care safety [331-333], with most based in dementia care settings. Three studies examined how the location of patients within the hospital setting can impact on safety, focusing on topics such as: privacy, female only wards and the use of segregated or combined wards/units [334-336]. The remaining three studies concerned staffing levels [337, 338] and ligature points [339].

Medication safety

The medication safety category included 17 publications (5%). Five studies focused on adverse events, and examined: antipsychotics side effects [340], how best to manage the effect of psychotropics on Long QT segments [341], antidepressants [342], and medication error reporting [343, 344]. Three studies investigated errors occurring in broader medication management processes [345-347] and a further five studies focused on medication administration specifically [348-352]. The only intervention study aiming to reduce these errors found that a new medication dispensing system did not have any significant impact on patient safety [353]. Two studies explored staff perceptions of illicit substance use [354, 355]. One further study described the development of a medication adherence intervention for patients who are prescribed mood-stabilizing medication for bipolar disorder [356].

Unauthorised leave

Unauthorised leave included 11 publications (3%). Three explored the patient experience of absconding, specifically relating to patient perspectives of treatment and involuntary commitment [357-359]. One study explored staff perspectives of absconding management techniques [360], and two studies evaluated interventions to reduce absconding rates; both were found to be effective [361, 362]. Two studies focused on wandering behaviour in women with dementia, linking wandering to physical environment factors, such as light, sound, crowding [363] and falls [364]. The pooled prevalence in wandering behaviour was 50.2% (95%CI 0.49-0.52) with high heterogeneity (I^2 78.0%) in two studies [363, 364] (online supplement 6). The final three studies examined the consequences [365, 366] and security measures surrounding absconding [367].

Clinical decision making

Clinical decision making accounted for 2% of the included publications (n=9). These publications covered the development of clinical judgements and decisions relating to incident management, risk assessment and diagnosis. Two studies explored the cultural

For peer review only - http://bmjopen.bmj.com/site/about/guidelines.xhtml

differences considered by clinicians in the diagnosis of African American patients [368, 369]. Clinical decisions on whether to engage in seclusion and/or restraint were explored in five studies [370-374] and two studies explored the variation in assessment and prediction of violence between staff and settings [375, 376].

Falls

Publications on falls formed the second smallest category within the review (n=6; 1%). Studies in this category focused on fall prevalence, falls experienced by older psychiatric inpatients with dementia and prevention/harm reduction techniques. A recurring risk factor for falling was found to be medication use [377-379]. Two fall prevention intervention studies did not identify significant benefits [380, 381], and one study explored barriers and facilitators to such interventions [382].

Infection prevention and control

One paper (<1%) focused on staff experiences of infection prevention and control in psychiatric clinical settings [383].

Discussion

Main findings

This is the first review to examine patient safety within inpatient mental health settings that uses robust systematic methodology. As a result, we have identified ten research categories: interpersonal violence, coercive interventions, safety culture, harm to self, safety of the physical environment, medication safety, unauthorised leave, clinical decision making, falls and infection prevention and control. In addition, we have been able to include a meta-analysis of incidence and prevalence of aggression (verbal and physical), coercive intervention and wandering behaviour, as well as providing an easily accessible reference index of literature in the inpatient mental health and patient safety domain. Previous reviews on this topic had focused on collating the literature on a restricted number (n=8) of pre-defined patient safety incidents (e.g. violence and aggression) [7], or the concept of patient safety in inpatient mental health setting more broadly (e.g. organisation management) [10]. As such, the findings presented here offer a contemporary view of the breadth and depth of patient safety research in inpatient mental health settings.

We were concerned to see that only 364 papers were identified as a result of our comprehensive search. Although this can be seen as a large number of publications for a systematic review, it is a relatively small number to cover the care of a wide range of patients

For peer review only - http://bmjopen.bmj.com/site/about/guidelines.xhtml

in a variety of inpatient mental health settings over a 20-year period (around 18 papers per year across all countries). Whilst important work not meeting our inclusion criteria (e.g. quality improvement initiatives and studies using secondary analysis of data) may have focused on patient safety in mental health, the lack of prospective peer reviewed publications adds to the ongoing discussion surrounding the disparity in research focusing on patient safety in physical and mental healthcare [384]. In addition, there was a paucity of high-quality research in the area; just over two thirds of the studies were considered to be 'fair', and only nine studies included in the meta-analysis were deemed 'good'. 'Poor' studies most frequently did not have clear research aims and objectives, study details were missing (e.g. sample(s) and setting(s) used) and they failed to discuss ethical and researcher bias issues. Some qualitative studies explored both staff and patients' perspectives, an important aspect of research, particularly when safety in this context is a relatively new area of knowledge. However, there was limited intervention research, particularly randomised controlled trials (RCTs). In the RCTs that were identified, sample sizes were mostly small.

The findings from the review also challenged our expectations in terms of breadth and depth of research. For example, we expected to find many publications on the prevention of suicide within inpatient settings due to the severity of harm. However, only one study that met inclusion criteria discussed suicide in relation to ligature points [339]. A scoping review also found only this one study, suggesting a consistency of approach [385]. This indicates that whilst the prevention of suicide is a well-established aspect of patient safety, it is now reviewed routinely, using pre-existing and secondary data, rather than through empirical research.

We also found little research focusing on the concepts required for system level reform [386], which was disappointing seeing as some improvements have been made in physical healthcare [387]. For example, in line with research in the physical health domain [388, 389], we were hoping to find several studies exploring how patient and family engagement in care can promote patient safety [390]. However, only two studies identified in our review had patient/family engagement as their primary focus [279, 280]. Similarly, we were expecting to identify literature investigating the lack of integration between physical and mental healthcare and the impact it has on patient safety [391]. However, the need to prevent and manage co-existing physical ill health was not identified in the review. This is surprising as patients with serious mental illness are twice as likely to die prematurely and much more likely to develop long term conditions or become disabled, as those without serious mental illness [392]. This patient group are also vulnerable to asphyxiation during restraint and rapid tranquilization [393].

For peer review only - http://bmjopen.bmj.com/site/about/guidelines.xhtml

Research on medication safety in inpatient mental health settings was also limited in this review. This was unexpected considering two-thirds of patients with mental health problems are prescribed medication and are therefore potentially at risk of experiencing a medication safety incident. Research pertaining to falls was also limited, contrasting with patient safety research within the physical health domain that includes a focus on slips, trips and falls [394].

Strengths and limitations

We used a robust patient safety taxonomy to provide a comprehensive list of all incident types. This resulted in a wide coverage of publications in terms of setting, country and population. We systematically searched, screened, extracted and appraised data. As a result, our systematic review draws together all relevant literature concerning patient safety within inpatient mental health settings, simultaneously operating as an index resource for clinicians and researchers.

There were several limitations. We used the definition of patient safety given by Vincent to guide this review. Whilst this is more nuanced than the original Institute of Medicine definition of safety “freedom from accidental injury” [395] and is widely accepted within the patient safety movement, it may be that a more suitable definition reflects the specific challenges within the inpatient mental health setting [396]. This review only included peer reviewed studies with primary data. Therefore, literature utilising secondary data such as pre-existing datasets and data from internal audits was excluded as it did not fulfil the criteria of being a prospective research study with clear research aims [397]. For example, data examined by the National Confidential Inquiry into Suicide and Homicide by People with Mental Illness (NCISH) is collected retrospectively from various sites across the country and would have been excluded from this review [398]. Moreover, non-peer reviewed quality improvement reports have also been excluded. This is due to (i) the large number of potential publications in this area, (ii) the need to define the scope and focus of the review, and (iii) the need for specificity as well as sensitivity. The investigation of patient safety in mental health inpatient settings using secondary data or in non-peer reviewed formats is an avenue for additional systematic reviews.

The last systematic literature search was conducted on 27th June 2019, meaning that literature published since this date will not have been included. In order to further build on the review published here, a *living* systematic review (an ongoing updated summary of high-quality research) [399], would continue to identify relevant literature in this area. In terms of

For peer review only - http://bmjopen.bmj.com/site/about/guidelines.xhtml

the meta-analysis, there was expected statistical and methodological variability in studies, particularly for physical and verbal aggression. It is possible that this was due to the inclusion of different definitions of aggression, time periods and type of inpatient setting. In relation to agreement between reviewers (including the use of recommended piloting of inclusion and exclusion criteria within the screening stage) [400], inter-rater reliability calculations only achieved substantial agreement (κ=0.61-0.80) at both the title and full-text screening stages. Whilst higher kappas have been reported in other systematic reviews, a substantial agreement is classified as more than acceptable [401].

Whilst the research spanned five continents, the UK, USA and Australia contributed over 50% of the included studies, leading to a potential cultural bias in the body of research identified within the review. We recommend that, where possible, future systematic reviews incorporate manuscripts in languages other than English to establish greater insight into the global literature on patient safety in inpatient mental health settings, with a view to limiting any cultural bias. Similarly, whilst the removal of publications denoting non-inpatient setting restricted the conclusions to the inpatient setting, issues pertaining to this environment are likely to be different to that of community, primary or social care settings. Additionally, studies were excluded before 1999 to coincide with the release of the Institute of Medicine's report "To Err is Human: Building a Safer Health System" [395]; this narrowed the review scope as the historical context was minimised.

Clinical implications and future research

This review informs academics, clinicians and service providers about the evidence base in the patient safety field within inpatient mental health settings. The findings allow researchers and clinicians to be directed to literature relevant to a given patient safety topic area, a useful starting point when developing practice guidelines [402]. Similarly, the findings may influence clinical practice, with those implementing interventions or designing service changes being able to easily access the current scientific understanding.

Future research should be informed by patient safety science more broadly and focus on filling the knowledge gaps highlighted in this review i.e. studies that explore (i) systems level improvement (ii) patient and carer engagement in safety, (iii) suicide prevention across different countries, (iv) the nature of medication safety in inpatient mental health settings and (v) the prevalence and impact of staff to patient violence. These findings support our previous expert consensus study where academic and service user experts agreed that patient driven research studies were needed [403]. The limited rigorous research surrounding patient safety within inpatient mental health settings necessitates future studies

For peer review only - http://bmjopen.bmj.com/site/about/guidelines.xhtml

to: (i) include large inpatient samples relevant to the research design, (ii) perform appropriate intervention testing and (iii) examine safety from different perspectives. It should also focus on high quality reporting of research, paying particular attention to the area of ethics, sampling and setting characteristics.

Conclusion

This is the first systematic review to comprehensively examine research on patient safety within inpatient mental health settings. It has drawn together the existing literature and shed light on the gaps in knowledge. Inpatient mental health settings may demonstrate unique patient safety challenges and more research is needed to achieve parity with physical health. Addressing this through a strong body of evidence, informed by patient safety science more broadly, will mean that mental healthcare policy makers are in a better position to address safety issues, and implement robust and evidence-based interventions to improve care.

Contributors

BT, LD, SCR, SAr and DD contributed to the design, data searches, data extraction, synthesis and writing of the report. HA contributed to the design, data extraction, meta-analysis and writing of the report. SAd and AD contributed to the design and synthesis, as well as writing and critically reviewing the report.

Declaration of interests

We declare no competing interests.

Funding

This work is supported by the National Institute for Health Research (NIHR) Imperial Patient Safety Translation Research Centre. Infrastructure support was provided by the NIHR Imperial Biomedical Research Centre.

Acknowledgements

We'd like to thank the librarians at St Mary's Library, Imperial College London for their support with the study.

Data Availability

No additional data available

Figure legends

For peer review only - http://bmjopen.bmj.com/site/about/guidelines.xhtml

Figure 1 - Flowchart of studies

For peer review only

References

1. Vincent, C., *Patient Safety* 2006, Edinburgh: Churchill Livingstone.
2. Brickell, T.A. and C. McLean, *Emerging issues and challenges for improving patient safety in mental health: a qualitative analysis of expert perspectives.* Journal of patient safety, 2011. **7**(1): p. 39-44.
3. Powell, J., et al., *Suicide in psychiatric hospital in-patients: risk factors and their predictive power.* The British Journal of Psychiatry, 2000. **176**(3): p. 266-272.
4. Roy, A. and R. Draper, *Suicide among psychiatric hospital in-patients.* Psychological Medicine, 1995. **25**(1): p. 199-202.
5. Gray, N.S., et al., *Prediction of violence and self-harm in mentally disordered offenders: A prospective study of the efficacy of HCR-20, PCL-R, and psychiatric symptomatology.* Journal of consulting and clinical psychology, 2003. **71**(3): p. 443.
6. Goh, S.E., P.H. Salmons, and R. Whittington, *Hospital Suicides: Are There Preventable Factors?: Profile of the Psychiatric Hospital Suicide.* The British Journal of Psychiatry, 1989. **154**(2): p. 247-249.
7. Brickell, T.A., T.L. Nicholls, and R.M. Procyshyn, *Patient safety in mental health.* 2000: Canadian Patient Safety Institute.
8. Vincent, G.M., *Psychopathy and violence risk assessment in youth.* Child and Adolescent Psychiatric Clinics, 2006. **15**(2): p. 407-428.
9. Taylor, T.L., et al., *A systematic review of the international published literature relating to quality of institutional care for people with longer term mental health problems.* BMC psychiatry, 2009. **9**(1): p. 55.
10. Kanerva, A., J. Lammintakanen, and T. Kivinen, *Patient safety in psychiatric inpatient care: a literature review.* Journal of psychiatric and mental health nursing, 2013. **20**(6): p. 541-548.
11. D'Lima, D., et al., *A systematic review of patient safety in mental health: a protocol based on the inpatient setting.* Systematic reviews, 2016. **5**(1): p. 203.
12. Moher, D., et al., *Preferred reporting items for systematic review and meta-analysis protocols (PRISMA-P) 2015 statement.* Systematic reviews, 2015. **4**(1): p. 1.
13. Agency., N.P.S. 1st Feb 2016]; Available from: http://www.nrls.npsa.nhs.uk/patient-safety-data/.
14. Cohen, J., *A coefficient of agreement for nominal scales.* Educational and psychological measurement, 1960. **20**(1): p. 37-46.
15. McHugh, M.L., *Interrater reliability: the kappa statistic.* Biochemia medica: Biochemia medica, 2012. **22**(3): p. 276-282.
16. Belur, J., et al., *Interrater reliability in systematic review methodology: exploring variation in coder decision-making.* Sociological methods & research, 2018: p. 0049124118799372.
17. Donaldson, M.S., J.M. Corrigan, and L.T. Kohn, *To err is human: building a safer health system*. Vol. 6. 2000: National Academies Press.
18. Hawker, S., et al., *Appraising the evidence: reviewing disparate data systematically.* Qualitative health research, 2002. **12**(9): p. 1284-1299.
19. Edwards, P., et al., *Identification of randomized controlled trials in systematic reviews: accuracy and reliability of screening records.* Statistics in medicine, 2002. **21**(11): p. 1635-1640.
20. Amoo, G. and F. Fatoye, *Aggressive behaviour and mental illness: a study of in-patients at Aro Neuropsychiatric Hospital, Abeokuta.* Nigerian journal of clinical practice, 2010. **13**(3).
21. Boström, A.M., et al., *Workplace aggression experienced by frontline staff in dementia care.* Journal of clinical nursing, 2012. **21**(9-10): p. 1453-1465.
22. Chen, W.-C., H.-G. Hwu, and J.-D. Wang, *Hospital staff responses to workplace violence in a psychiatric hospital in Taiwan.* International journal of occupational and environmental health, 2009. **15**(2): p. 173-179.

For peer review only - http://bmjopen.bmj.com/site/about/guidelines.xhtml

23. Daffern, M., M. Mayer, and T. Martin, *Staff gender ratio and aggression in a forensic psychiatric hospital.* International journal of mental health nursing, 2006. **15**(2): p. 93-99.
24. Ilkiw-Lavalle, O. and B.F. Grenyer, *Differences between patient and staff perceptions of aggression in mental health units.* Psychiatric Services, 2003. **54**(3): p. 389-393.
25. Jacob, P., et al., *Clinical characteristics of aggression in children and adolescents admitted to a tertiary care centre.* Asian journal of psychiatry, 2013. **6**(6): p. 556-559.
26. Lehmann, L.S., R.A. McCormick, and K.W. Kizer, *A survey of assaultive behavior in Veterans Health Administration facilities.* Psychiatric Services, 1999. **50**(3): p. 384-389.
27. Raveendranathan, D., P.S. Chandra, and S.K. Chaturvedi, *Violence among psychiatric inpatients: a victim's perspective.* East Asian archives of psychiatry, 2012. **22**(4).
28. Ryan, E.P., et al., *A prospective study of assault against staff by youths in a state psychiatric hospital.* Psychiatric services, 2004. **55**(6): p. 665-670.
29. Schwartz, T.L. and T.L. Park, *Assaults by patients on psychiatric residents: a survey and training recommendations.* Psychiatric Services, 1999. **50**(3): p. 381-383.
30. Sukhodolsky, D.G., L. Cardona, and A. Martin, *Characterizing aggressive and noncompliant behaviors in a children's psychiatric inpatient setting.* Child Psychiatry and Human Development, 2005. **36**(2): p. 177-193.
31. Wystanski, M., *Assaultive behaviour in psychiatrically hospitalized elderly: a response to psychosocial stimulation and changes in pharmacotherapy.* International journal of geriatric psychiatry, 2000. **15**(7): p. 582-585.
32. Kelly, E.L., et al., *Sources of Social Support After Patient Assault as Related to Staff Well-Being.* Journal of interpersonal violence, 2017: p. 886260517738779-886260517738779.
33. Niu, S.F., et al., *Prevalence of workplace violent episodes experienced by nurses in acute psychiatric settings.* Plos One, 2019. **14**(1).
34. Danivas, V., et al., *Observational study of aggressive behaviour and coercion on an Indian acute ward.* Asian Journal of Psychiatry, 2016. **22**: p. 150-156.
35. Podubinski, T., et al., *Patient characteristics associated with aggression in mental health units.* Psychiatry Research, 2017. **250**: p. 141-145.
36. van den Bogaard, K., et al., *Characteristics of Aggressive Behavior in People With Mild to Borderline Intellectual Disability and Co-Occurring Psychopathology.* Journal of Mental Health Research in Intellectual Disabilities, 2018. **11**(2): p. 124-142.
37. Pekurinen, V.M., et al., *Organizational justice and collaboration among nurses as correlates of violent assaults by patients in psychiatric care.* Psychiatric Services, 2017. **68**(5): p. 490-496.
38. Kelly, E.L., et al., *Well-Being and Safety Among Inpatient Psychiatric Staff: The Impact of Conflict, Assault, and Stress Reactivity.* Administration and policy in mental health, 2016. **43**(5): p. 703-716.
39. Bilici, R., M. Sercan, and F. Izci, *Levels of the Staff's Exposure to Violence at Locked Psychiatric Clinics: A Comparison by Occupational Groups.* Issues in Mental Health Nursing, 2016. **37**(7): p. 501-506.
40. Daffern, M., J. Ogloff, and K. Howells, *Aggression in an Australian forensic psychiatric hospital.* The British Journal of Forensic Practice, 2003. **5**(4): p. 18-28.
41. Sato, M., et al., *Characteristics of aggression among psychiatric inpatients by ward type in Japan: Using the Staff Observation Aggression Scale - Revised (SOAS-R).* International Journal of Mental Health Nursing, 2017. **26**(6): p. 602-611.
42. Ben-Zeev, D., et al., *Use of multimodal technology to identify digital correlates of violence among inpatients with serious mental illness: A pilot study.* Psychiatric Services, 2017. **68**(10): p. 1088-1092.
43. Bowers, L., et al., *Identifying key factors associated with aggression on acute inpatient psychiatric wards.* Issues Ment Health Nurs, 2009. **30**(4): p. 260-71.

For peer review only - http://bmjopen.bmj.com/site/about/guidelines.xhtml

44. Selenius, H., S. Leppanen Ostman, and S. Strand, *Self-harm as a risk factor for inpatient aggression among women admitted to forensic psychiatric care.* Nordic Journal of Psychiatry, 2016. **70**(7): p. 554-560.
45. Van de Sande, R., et al., *Aggression and seclusion on acute psychiatric wards: effect of short-term risk assessment.* The British Journal of Psychiatry, 2011. **199**(6): p. 473-478.
46. Park, J.S. and K. Lee, *Modification of severe violent and aggressive behavior among psychiatric inpatients through the use of a short-term token economy.* Journal of Korean Academy of Nursing, 2012. **42**(7): p. 1062-1069.
47. Needham, I., et al., *The effectiveness of two interventions in the management of patient violence in acute mental inpatient settings: report on a pilot study.* Journal of psychiatric and mental health nursing, 2004. **11**(5): p. 595-601.
48. Murphy, C.J. and A.M. Siv, *A one year study of Mode Deactivation Therapy: Adolescent residential patients with conduct and personality disorders.* International Journal of Behavioral Consultation and Therapy, 2007. **3**(3): p. 327.
49. McLaughlin, S., et al., *A pilot study to test an intervention for dealing with verbal aggression.* British journal of nursing, 2010. **19**(8): p. 489-494.
50. Killick, S. and D. Allen, *Training staff in an adolescent inpatient psychiatric unit in positive approaches to managing aggressive and harmful behaviour: does it improve confidence and knowledge?* Child Care in Practice, 2005. **11**(3): p. 323-339.
51. Giles, G., et al., *Twenty-month effectiveness of a non-aversive, long-term, low-cost programme for persons with persisting neurobehavioural disability.* Brain Injury, 2005. **19**(10): p. 753-764.
52. Daffern, M., et al., *The impact of structured risk assessments followed by management recommendations on aggression in patients with personality disorder.* The journal of forensic psychiatry & psychology, 2009. **20**(5): p. 661-679.
53. Yip, V.C., et al., *A non-randomised controlled trial of the R&R2MHP cognitive skills program in high risk male offenders with severe mental illness.* BMC psychiatry, 2013. **13**(1): p. 267.
54. Lanza, M., et al., *The Violence Prevention Community Meeting: A multi-site study.* Archives of Psychiatric Nursing, 2016. **30**(3): p. 382-386.
55. Lanza, M.L., et al., *Reducing violence against nurses: the violence prevention community meeting.* Issues in mental health nursing, 2009. **30**(12): p. 745-750.
56. Calabro, K., T.A. Mackey, and S. Williams, *Evaluation of training designed to prevent and manage patient violence.* Issues in Mental Health Nursing, 2002. **23**(1): p. 3-15.
57. Björkdahl, A., G. Hansebo, and T. Palmstierna, *The influence of staff training on the violence prevention and management climate in psychiatric inpatient units.* Journal of psychiatric and mental health nursing, 2013. **20**(5): p. 396-404.
58. Arguvanli, S.Ç., et al., *Effect of aggression management training program on knowledge and attitudes of nurses working at psychiatric clinics/Agresyon yönetimi egitim programinin psikiyatri kliniklerinde çalisan hemsirelerin bilgi ve tutumlarina etkisi.* Anadolu Psikiyatri Dergisi, 2015. **16**(5): p. 323.
59. Caspi, N., et al., *Pindolol augmentation in aggressive schizophrenic patients: a double-blind crossover randomized study.* International clinical psychopharmacology, 2001. **16**(2): p. 111-115.
60. Davies, B.E., et al., *An evaluation of the effectiveness of positive behavioural support within a medium secure mental health forensic service.* Journal of Forensic Psychiatry and Psychology, 2019. **30**(1): p. 38-52.
61. Isaak, V., et al., *Enhancing the safety climate and reducing violence against staff in closed hospital wards.* Workplace health & safety, 2017. **65**(9): p. 409-416.
62. Price, O., et al., *Patient perspectives on barriers and enablers to the use and effectiveness of de-escalation techniques for the management of violence and aggression in mental health settings.* Journal of advanced nursing, 2018. **74**(3): p. 614-625.

For peer review only - http://bmjopen.bmj.com/site/about/guidelines.xhtml

63. Hvidhjelm, J., et al., *Aggression in Psychiatric Wards: Effect of the Use of a Structured Risk Assessment.* Issues in mental health nursing, 2016. **37**(12): p. 960-967.
64. Olsson, H. and U.K. Schon, *Reducing violence in forensic care-how does it resemble the domains of a recovery-oriented care?* Journal of Mental Health, 2016. **25**(6): p. 506-511.
65. Mistler, L.A., et al., *Mobile Mindfulness Intervention on an Acute Psychiatric Unit: Feasibility and Acceptability Study.* Jmir Mental Health, 2017. **4**(3).
66. Needham, I., et al., *The effect of a training course in aggression management on mental health nurses' perceptions of aggression: a cluster randomised controlled trial.* International Journal of Nursing Studies, 2005. **42**(6): p. 649-655.
67. Sjöström, N., et al., *Violence and its prediction at a psychiatric hospital.* European Psychiatry, 2001. **16**(8): p. 459-465.
68. Sival, R.C., et al., *Is aggressive behaviour influenced by the use of a behaviour rating scale in patients in a psychogeriatric nursing home?* International journal of geriatric psychiatry, 2000. **15**(2): p. 108-111.
69. Skovdahl, K., A.L. Kihlgren, and M. Kihlgren, *Dementia and aggressiveness: video recorded morning care from different care units.* Journal of clinical nursing, 2003. **12**(6): p. 888-898.
70. Stone, T., et al., *Wounding words: swearing and verbal aggression in an inpatient setting.* Perspectives in psychiatric care, 2011. **47**(4): p. 194-203.
71. Sutton, D., et al., *Optimizing arousal to manage aggression: A pilot study of sensory modulation.* International Journal of Mental Health Nursing, 2013. **22**(6): p. 500-511.
72. Lipscomb, J.A., et al., *Safety climate and workplace violence prevention in state-run residential addiction treatment centers.* Work, 2012. **42**(1): p. 47-56.
73. Wright, S., et al., *Breakaway and physical restraint techniques in acute psychiatric nursing: results from a national survey of training and practice.* The Journal of Forensic Psychiatry & Psychology, 2005. **16**(2): p. 380-398.
74. Berg, J., R. KALTIALA-HEINO, and M. Välimäki, *Management of aggressive behaviour among adolescents in forensic units: a four-country perspective.* Journal of psychiatric and mental health nursing, 2011. **18**(9): p. 776-785.
75. Biancosino, B., et al., *Violent behavior in acute psychiatric inpatient facilities: a national survey in Italy.* The Journal of nervous and mental disease, 2009. **197**(10): p. 772-782.
76. Caspi, E., *Aggressive behaviors between residents with dementia in an assisted living residence.* Dementia, 2015. **14**(4): p. 528-546.
77. Chaplin, R., et al., *Aggression on psychiatric inpatient units for older adults and adults of working age.* International journal of geriatric psychiatry, 2008. **23**(8): p. 874-876.
78. Delaney, J., et al., *An exploratory investigation into the nursing management of aggression in acute psychiatric settings.* Journal of Psychiatric and Mental Health Nursing, 2001. **8**(1): p. 77-84.
79. Meaden, A., D. Hacker, and K. Spencer, *Acute aggression risk: an early warning signs methodology.* The Journal of Forensic Practice, 2013. **15**(1): p. 21-31.
80. Umut, G., et al., *Relationship between treatment adherence, insight and violence among schizophrenia inpatients in a training hospital sample.* Düsünen Adam: The Journal of Psychiatry and Neurological Sciences, 2012. **25**: p. 212-220.
81. Bahareethan, M. and A. Shah, *Aggressive behaviour, staff attitude and staff perception of patients on two continuing care psychogeriatric wards.* Aging & Mental Health, 2000. **4**(1): p. 66-71.
82. Berg, J., et al., *Staff's perception of adolescent aggressive behaviour in four European forensic units: A qualitative interview study.* Nordic journal of psychiatry, 2013. **67**(2): p. 124-131.

For peer review only - http://bmjopen.bmj.com/site/about/guidelines.xhtml

83. Stevenson, K.N., et al., *Registered nurses' experiences of patient violence on acute care psychiatric inpatient units: an interpretive descriptive study.* BMC nursing, 2015. **14**(1): p. 35.
84. Spokes, K., et al., *Hovis–the Hertfordshire/Oxfordshire violent incident study.* Journal of Psychiatric and Mental Health Nursing, 2002. **9**(2): p. 199-209.
85. Zuzelo, P.R., S.S. Curran, and M.A. Zeserman, *Registered nurses' and behavior health associates' responses to violent inpatient interactions on behavioral health units.* Journal of the American Psychiatric Nurses Association, 2012. **18**(2): p. 112-126.
86. Camuccio, C., et al., *Managing distressed and disturbed patients: the thoughts and feelings experienced by Italian nurses.* Journal of Psychiatric and Mental Health Nursing, 2012. **19**(9): p. 807-815.
87. McCann, T.V., J. Baird, and E. Muir-Cochrane, *Factors influencing clinicians' attitudes about aggression in Australian acute old age psychiatry inpatient units: a cross sectional survey design.* Issues in mental health nursing, 2014. **35**(7): p. 542-550.
88. Tema, T., M. Poggenpoel, and C. Myburgh, *Experiences of psychiatric nurses exposed to hostility from patients in a forensic ward.* Journal of Nursing Management, 2011. **19**(7): p. 915-924.
89. Nijman, H., et al., *Psychiatric nurses' experiences with inpatient aggression.* Aggressive behavior, 2005. **31**(3): p. 217-227.
90. Chen, W.-C., et al., *Workplace violence on workers caring for long-term institutionalized schizophrenic patients in Taiwan.* Journal of Occupational health, 2007. **49**(4): p. 311-316.
91. Cutcliffe, J.R., *Qualified nurses' lived experience of violence perpetrated by individuals suffering from enduring mental health problems: a hermeneutic study.* International Journal of Nursing Studies, 1999. **36**(2): p. 105-116.
92. Evans, R.E. and S. Petter, *Identifying mitigating and challenging beliefs in dealing with threatening patients: an analysis of experiences of clinicians working in a psychiatric intensive care unit.* Journal of Psychiatric Intensive Care, 2012. **8**(2): p. 113-119.
93. Lantta, T., et al., *Implementing the Dynamic Appraisal of Situational Aggression in mental health units.* Clinical nurse specialist, 2015. **29**(4): p. 230-243.
94. Tomagová, M., et al., *NURSES' EXPERIENCE AND ATTITUDES TOWARDS INPATIENT AGGRESSION ON PSYCHIATRIC WARDS.* Central European Journal of Nursing & Midwifery, 2016. **7**(3): p. 462-469.
95. Hylen, U., et al., *Providing Good Care in the Shadow of Violence - An Interview Study with Nursing Staff and Ward Managers in Psychiatric Inpatient Care in Sweden.* Issues in mental health nursing, 2019. **40**(2): p. 148-157.
96. Lantta, T., et al., *Violent events, ward climate and ideas for violence prevention among nurses in psychiatric wards: A focus group study.* International Journal of Mental Health Systems, 2016. **10**.
97. Meehan, T., W. McIntosh, and H. Bergen, *Aggressive behaviour in the high-secure forensic setting: the perceptions of patients.* Journal of Psychiatric and Mental Health Nursing, 2006. **13**(1): p. 19-25.
98. Olsson, H., et al., *Reducing or increasing violence in forensic care: a qualitative study of inpatient experiences.* Archives of psychiatric nursing, 2015. **29**(6): p. 393-400.
99. Van Wijk, E., A. Traut, and H. Julie, *Environmental and nursing-staff factors contributing to aggressive and violent behaviour of patients in mental health facilities.* curationis, 2014. **37**(1): p. 01-08.
100. Daffern, M., *Assessing the functions of aggression in psychiatric inpatients.* The Behavior Analyst Today, 2007. **8**(1): p. 43.

For peer review only - http://bmjopen.bmj.com/site/about/guidelines.xhtml

101. Duxbury, J. and R. Whittington, *Causes and management of patient aggression and violence: staff and patient perspectives.* Journal of advanced nursing, 2005. **50**(5): p. 469-478.
102. Janicki, N., *Prosecuting inpatient violence: perceptions of staff, patients and others in a women's enhanced medium secure service.* The British Journal of Forensic Practice, 2009. **11**(4): p. 27-38.
103. Lawn, S. and R. Pols, *Nicotine withdrawal: pathway to aggression and assault in the locked psychiatric ward?* Australasian Psychiatry, 2003. **11**(2): p. 199-203.
104. Nolan, K.A., et al., *Staff and patient views of the reasons for aggressive incidents: a prospective, incident-based study.* Psychiatric quarterly, 2009. **80**(3): p. 167-172.
105. Dickens, G., M. Piccirillo, and N. Alderman, *Causes and management of aggression and violence in a forensic mental health service: perspectives of nurses and patients.* International journal of mental health nursing, 2013. **22**(6): p. 532-544.
106. Wright, K., et al., *A qualitative study into the attitudes of patients and staff towards violence and aggression in a high security hospital.* Journal of psychiatric and mental health nursing, 2014. **21**(2): p. 184-188.
107. Lamanna, D., et al., *Aggression in psychiatric hospitalizations: a qualitative study of patient and provider perspectives.* Journal of Mental Health, 2016. **25**(6): p. 536-542.
108. Paschali, M., et al., *A systematic evaluation of impulsive-aggressive behavior in psychogeriatric inpatients using the staff observation aggression scale-revision (SOAS-R).* International psychogeriatrics, 2018. **30**(1): p. 61-68.
109. Almvik, R., K. Rasmussen, and P. Woods, *Challenging behaviour in the elderly—monitoring violent incidents.* International journal of geriatric psychiatry, 2006. **21**(4): p. 368-374.
110. Reininghaus, U., et al., *The high secure psychiatric hospitals' nursing staff stress survey 3: Identifying stress resistance resources in the stress process of physical assault.* Personality and individual differences, 2007. **42**(3): p. 397-408.
111. Trenoweth, S., *Perceiving risk in dangerous situations: risks of violence among mental health inpatients.* Journal of Advanced Nursing, 2003. **42**(3): p. 278-287.
112. De Niet, G., G. Hutschemaekers, and B. Lendemeijer, *Is the reducing effect of the Staff Observation Aggression Scale owing to a learning effect? An explorative study.* Journal of Psychiatric and Mental Health Nursing, 2005. **12**(6): p. 687-694.
113. de Looff, P., et al., *Burnout symptoms in forensic psychiatric nurses and their associations with personality, emotional intelligence and client aggression: A cross-sectional study.* Journal of psychiatric and mental health nursing, 2018. **25**(8): p. 506-516.
114. Bharwani, G., et al., *Individualized behavior management program for Alzheimer's/dementia residents using behavior-based ergonomic therapies.* American Journal of Alzheimer's Disease & Other Dementias®, 2012. **27**(3): p. 188-195.
115. Carlson, G.A., et al., *Liquid risperidone in the treatment of rages in psychiatrically hospitalized children with possible bipolar disorder.* Bipolar disorders, 2010. **12**(2): p. 205-212.
116. Chan, S., et al., *The clinical effectiveness of a multisensory therapy on clients with developmental disability.* Research in Developmental Disabilities, 2005. **26**(2): p. 131-142.
117. DeYoung, S., G. Just, and R. Harrison, *Decreasing aggressive, agitated, or disruptive behavior: Participation in a behavior management unit.* Journal of Gerontological Nursing, 2002. **28**(6): p. 22-31.
118. Higueras, A., et al., *Effects of a humor-centered activity on disruptive behavior in patients in a general hospital psychiatric ward.* International Journal of Clinical and Health Psychology, 2006. **6**(1): p. 53-64.
119. Speziale, J., et al., *Moving forward: Evaluating a curriculum for managing responsive behaviors in a geriatric psychiatry inpatient population.* The Gerontologist, 2009. **49**(4): p. 570-576.

For peer review only - http://bmjopen.bmj.com/site/about/guidelines.xhtml

120. Zwijsen, S.A., et al., *Coming to grips with challenging behavior: a cluster randomized controlled trial on the effects of a multidisciplinary care program for challenging behavior in dementia.* Journal of the American Medical Directors Association, 2014. **15**(7): p. 531. e1-531. e10.
121. Bennett, R., V. Ramakrishna, and D. Maganty, *Management of disturbed behaviour in a psychiatric intensive care unit: views of staff on options for intervention.* Journal of Psychiatric Intensive Care, 2011. **7**(2): p. 85-89.
122. Foley, K.L., et al., *Staff perceptions of successful management of severe behavioral problems in dementia special care units.* Dementia, 2003. **2**(1): p. 105-124.
123. Hallett, N. and G.L. Dickens, *De-escalation: A survey of clinical staff in a secure mental health inpatient service.* International Journal of Mental Health Nursing, 2015. **24**(4): p. 324-333.
124. Bowers, L., *Association between staff factors and levels of conflict and containment on acute psychiatric wards in England.* Psychiatric services, 2009. **60**(2): p. 231-239.
125. Lowe, T., N. Wellman, and R. Taylor, *Limit-setting and decision-making in the management of aggression.* Journal of Advanced Nursing, 2003. **41**(2): p. 154-161.
126. Ireland, J.L., et al., *Impacting on factors promoting intra-group aggression in secure psychiatric settings.* Heliyon, 2019. **5**(3).
127. Quirk, A., P. Lelliott, and C. Seale, *Risk management by patients on psychiatric wards in London: An ethnographic study.* Health, Risk & Society, 2005. **7**(1): p. 85-91.
128. Ireland, C.A., L. Halpin, and C. Sullivan, *Critical incidents in a forensic psychiatric population: an exploratory study of motivational factors.* The Journal of Forensic Psychiatry & Psychology, 2014. **25**(6): p. 714-732.
129. Jeffs, L., et al., *What near misses tell us about risk and safety in mental health care.* Journal of psychiatric and mental health nursing, 2012. **19**(5): p. 430-437.
130. Koukia, E., et al., *Greek mental health nurses' practices and attitudes in the management of acute cases.* Issues in mental health nursing, 2013. **34**(3): p. 192-197.
131. Terkelsen, T.B. and I.B. Larsen, *Fear, danger and aggression in a Norwegian locked psychiatric ward: Dialogue and ethics of care as contributions to combating difficult situations.* Nursing Ethics, 2016. **23**(3): p. 308-317.
132. Cole, M., D. Baldwin, and P. Thomas, *Sexual assault on wards: Staff actions and reactions.* International journal of psychiatry in clinical practice, 2003. **7**(4): p. 239-242.
133. Phillips, L., *Reflections on the education and training of mental health staff who work with women who have been sexually abused in childhood.* Journal of psychiatric and mental health nursing, 2011. **18**(8): p. 696-705.
134. Gallop, R. and R. DiNunzio, *Abused women's concerns about safety and the therapeutic environment during psychiatric hospitalization.* Canadian Journal of Nursing Research Archive, 1999. **31**(2).
135. Yang, M.H., et al., *The efficacy of acupressure for decreasing agitated behaviour in dementia: a pilot study.* Journal of Clinical nursing, 2007. **16**(2): p. 308-315.
136. Cormac, I., I. Russell, and M. Ferriter, *Review of seclusion policies in high secure hospitals and medium secure units in England, Scotland and Wales.* Journal of psychiatric and mental health nursing, 2005. **12**(3): p. 380-382.
137. Georgieva, I., C.L. Mulder, and E. Noorthoorn, *Reducing seclusion through involuntary medication: a randomized clinical trial.* Psychiatry research, 2013. **205**(1-2): p. 48-53.
138. Kirkevold, Ø. and K. Engedal, *Prevalence of patients subjected to constraint in Norwegian nursing homes.* Scandinavian journal of caring sciences, 2004. **18**(3): p. 281-286.

For peer review only - http://bmjopen.bmj.com/site/about/guidelines.xhtml

139. Raboch, J., et al., *Use of coercive measures during involuntary hospitalization: findings from ten European countries.* Psychiatric Services, 2010. **61**(10): p. 1012-1017.

140. Steinert, T., et al., *Diagnosis-related frequency of compulsory measures in 10 German psychiatric hospitals and correlates with hospital characteristics.* Social Psychiatry and Psychiatric Epidemiology, 2007. **42**(2): p. 140-145.

141. Gowda, G.S., et al., *Restraint prevalence and perceived coercion among psychiatric inpatients from South India: A prospective study.* Asian Journal of Psychiatry, 2018. **36**: p. 10-16.

142. Gowda, G., et al., *Clinician attitude and perspective on the use of coercive measures in clinical practice from tertiary care mental health establishment - A cross-sectional study.* Indian Journal of Psychiatry, 2019. **61**(2): p. 151-155.

143. Hotzy, F., et al., *Attitudinal variance among patients, next of kin and health care professionals towards the use of containment measures in three psychiatric hospitals in Switzerland.* BMC Psychiatry, 2019. **19**(1): p. 128.

144. Vedana, K.G.G., et al., *Physical and mechanical restraint in psychiatric units: Perceptions and experiences of nursing staff.* Archives of psychiatric nursing, 2018. **32**(3): p. 367-372.

145. Krieger, E., et al., *Patients' attitudes towards and acceptance of coercion in psychiatry.* Psychiatry Research, 2018. **260**: p. 478-485.

146. Reynolds, E.K., et al., *Use of Modified Positive Behavioral Interventions and Supports in a Psychiatric Inpatient Unit for High-Risk Youths.* Psychiatric Services, 2016. **67**(5): p. 570-573.

147. Brady, N.S., et al., *Patients' experiences of restrictive interventions in Australia: Findings from the 2010 Australian survey of psychosis.* Psychiatric Services, 2017. **68**(9): p. 966-969.

148. Bigwood, S. and M. Crowe, *'It's part of the job, but it spoils the job': A phenomenological study of physical restraint.* International journal of mental health nursing, 2008. **17**(3): p. 215-222.

149. Lee, S., et al., *Views of nursing staff on the use of physical restraint.* Journal of Psychiatric and Mental Health Nursing, 2003. **10**(4): p. 425-430.

150. Perkins, E., et al., *Physical restraint in a therapeutic setting; a necessary evil?* International journal of law and psychiatry, 2012. **35**(1): p. 43-49.

151. Sequeira, H. and S. Halstead, *The psychological effects on nursing staff of administering physical restraint in a secure psychiatric hospital:'When I go home, it's then that I think about it'.* The British Journal of Forensic Practice, 2004. **6**(1): p. 3-15.

152. Exworthy, T., et al., *Seclusion: punitive or protective?* Journal of Forensic Psychiatry, 2001. **12**(2): p. 423-433.

153. Kuosmanen, L., et al., *Seclusion experienced by mental health professionals.* Journal of psychiatric and mental health nursing, 2015. **22**(5): p. 333-336.

154. Muir-Cochrane, E.C., J. Baird, and T. McCann, *Nurses' experiences of restraint and seclusion use in short-stay acute old age psychiatry inpatient units: a qualitative study.* Journal of Psychiatric and Mental Health Nursing, 2015. **22**(2): p. 109-115.

155. Duxbury, J., et al., *Staff experiences and understandings of the REsTRAIN Yourself initiative to minimize the use of physical restraint on mental health wards.* International journal of mental health nursing, 2019.

156. Nielsen, L.D., et al., *Forensic mental health clinician's experiences with and assessment of alliance regarding the patient's readiness to be released from mechanical restraint.* International Journal of Mental Health Nursing, 2018. **27**(1): p. 116-125.

157. Kontio, R., et al., *Patient restrictions: are there ethical alternatives to seclusion and restraint?* Nurs Ethics, 2010. **17**(1): p. 65-76.

For peer review only - http://bmjopen.bmj.com/site/about/guidelines.xhtml

158. Chien, W.-T., et al., *Psychiatric inpatients' perceptions of positive and negative aspects of physical restraint.* Patient Education and Counseling, 2005. **59**(1): p. 80-86.
159. Knowles, S.F., J. Hearne, and I. Smith, *Physical restraint and the therapeutic relationship.* The Journal of Forensic Psychiatry & Psychology, 2015. **26**(4): p. 461-475.
160. Ezeobele, I., et al., *Patients' lived seclusion experience in acute psychiatric hospital in the U nited S tates: a qualitative study.* Journal of psychiatric and mental health nursing, 2014. **21**(4): p. 303-312.
161. Faschingbauer, K.M., C. Peden-McAlpine, and W. Tempel, *Use of seclusion: finding the voice of the patient to influence practice.* Journal of psychosocial nursing and mental health services, 2013. **51**(7): p. 32-38.
162. Holmes, D., S.L. Kennedy, and A. Perron, *The mentally ill and social exclusion: a critical examination of the use of seclusion from the patient's perspective.* Issues in mental health nursing, 2004. **25**(6): p. 559-578.
163. Bergk, J., et al., *A randomized controlled comparison of seclusion and mechanical restraint in inpatient settings.* Psychiatric Services, 2011. **62**(11): p. 1310-1317.
164. Kontio, R., et al., *Seclusion and restraint in psychiatry: patients' experiences and practical suggestions on how to improve practices and use alternatives.* Perspectives in psychiatric care, 2012. **48**(1): p. 16-24.
165. Larue, C., et al., *The experience of seclusion and restraint in psychiatric settings: perspectives of patients.* Issues in Mental Health Nursing, 2013. **34**(5): p. 317-324.
166. Bonner, G., et al., *Trauma for all: a pilot study of the subjective experience of physical restraint for mental health inpatients and staff in the UK.* Journal of Psychiatric and Mental Health Nursing, 2002. **9**(4): p. 465-473.
167. Fish, R. and C. Hatton, *Gendered experiences of physical restraint on locked wards for women.* Disability & Society, 2017. **32**(6): p. 790-809.
168. Wilson, C., et al., *Mental health inpatients' and staff members' suggestions for reducing physical restraint: A qualitative study.* Journal of psychiatric and mental health nursing, 2018. **25**(3): p. 188-200.
169. Fish, R., *'Behind This Wall' - Experiences of Seclusion on Locked Wards for Women.* Scandinavian Journal of Disability Research, 2018. **20**(1): p. 139-151.
170. Gowda, G., et al., *Caregivers' attitude and perspective on coercion and restraint practices on psychiatric inpatients from South India.* Journal of Neurosciences in Rural Practice, 2019. **10**(2): p. 261-266.
171. Larue, C., et al., *Identification and analysis of factors contributing to the reduction in seclusion and restraint for a population with intellectual disability.* Journal of Applied Research in Intellectual Disabilities, 2018. **31**(2): p. e212-e222.
172. Wilson, C., et al., *Is restraint a 'necessary evil' in mental health care? Mental health inpatients' and staff members' experience of physical restraint.* International journal of mental health nursing, 2017. **26**(5): p. 500-512.
173. Holmes, D., S.J. Murray, and N. Knack, *Experiencing Seclusion in a Forensic Psychiatric Setting: A Phenomenological Study.* Journal of Forensic Nursing, 2015. **11**(4): p. 200-213.
174. Bonner, G. and N. Wellman, *Postincident review of aggression and violence in mental health settings.* Journal of psychosocial nursing and mental health services, 2010. **48**(7): p. 35-40.
175. Bowers, L., et al., *Manual restraint and shows of force: the City-128 study.* International Journal of Mental Health Nursing, 2012. **21**(1): p. 30-40.
176. Tompsett, C.J., S. Domoff, and P. Boxer, *Prediction of restraints among youth in a psychiatric hospital: application of translational action research.* Journal of clinical psychology, 2011. **67**(4): p. 368-382.

For peer review only - http://bmjopen.bmj.com/site/about/guidelines.xhtml

177. Boumans, C., et al., *Nurses' decision on seclusion: patient characteristics, contextual factors and reflexivity in teams.* Journal of Psychiatric and Mental Health Nursing, 2012. **19**(3): p. 264-270.
178. Mason, T. and E. Whitehead, *Some specific problems of secluding female patients.* Medicine, Science and the Law, 2001. **41**(4): p. 315-324.
179. Ryan, R. and B. Happell, *Learning from experience: Using action research to discover consumer needs in post-seclusion debriefing.* International Journal of Mental Health Nursing, 2009. **18**(2): p. 100-107.
180. Whitecross, F., A. Seeary, and S. Lee, *Measuring the impacts of seclusion on psychiatry inpatients and the effectiveness of a pilot single-session post-seclusion counselling intervention.* International Journal of Mental Health Nursing, 2013. **22**(6): p. 512-521.
181. Bleijlevens, M.H., et al., *Process evaluation of a multicomponent intervention program (EXBELT) to reduce belt restraints in nursing homes.* Journal of the American Medical Directors Association, 2013. **14**(8): p. 599-604.
182. Pellfolk, T.J.E., et al., *Effects of a restraint minimization program on staff knowledge, attitudes, and practice: a cluster randomized trial.* Journal of the American Geriatrics Society, 2010. **58**(1): p. 62-69.
183. Schreiner, G.M., C.G. Crafton, and J.A. Sevin, *Decreasing the use of mechanical restraints and locked seclusion.* Administration and Policy in Mental Health and Mental Health Services Research, 2004. **31**(6): p. 449-463.
184. Ching, H., et al., *Reducing the use of seclusion in a forensic psychiatric hospital: assessing the impact on aggression, therapeutic climate and staff confidence.* The Journal of Forensic Psychiatry & Psychology, 2010. **21**(5): p. 737-760.
185. Long, C.G., et al., *Reducing the use of seclusion in a secure service for women.* Journal of Psychiatric Intensive Care, 2015. **11**(2): p. 84-94.
186. Smith, S. and J. Jones, *Use of a sensory room on an intensive care unit.* Journal of psychosocial nursing and mental health services, 2013. **52**(5): p. 22-30.
187. Espinosa, L., et al., *Milieu improvement in psychiatry using evidence-based practices: the long and winding road of culture change.* Archives of psychiatric nursing, 2015. **29**(4): p. 202-207.
188. Kontio, R., et al., *Impact of eLearning course on nurses' professional competence in seclusion and restraint practices: a randomized controlled study (ISRCTN32869544).* Journal of Psychiatric and Mental Health Nursing, 2011. **18**(9): p. 813-821.
189. Goulet, M.-H., C. Larue, and A.J. Lemieux, *A pilot study of "post-seclusion and/or restraint review" intervention with patients and staff in a mental health setting.* Perspectives in psychiatric care, 2018. **54**(2): p. 212-220.
190. Blair, E.W., et al., *Reduction of Seclusion and Restraint in an Inpatient Psychiatric Setting: A Pilot Study.* Psychiatric Quarterly, 2017. **88**(1).
191. Newman, J., O. Paun, and L. Fogg, *Effects of a Staff Training Intervention on Seclusion Rates on an Adult Inpatient Psychiatric Unit.* Journal of psychosocial nursing and mental health services, 2018. **56**(6): p. 23-30.
192. Elzubeir, K. and S. Dye, *Can amount and duration of seclusion be reduced in psychiatric intensive care units by agreeing SMART goals with patients?* Journal of Psychiatric Intensive Care, 2017. **13**(2): p. 109-116.
193. Huizing, A.R., et al., *Short-term effects of an educational intervention on physical restraint use: a cluster randomized trial.* BMC geriatrics, 2006. **6**(1): p. 17.
194. Abdel-Hussein, N.H. and S.H. Mohamed, *Effectiveaness of an educational program on nurses' knowledge toward restraint and seclusion for inpatients at psychiatric teaching hospitals.* Indian Journal of Public Health Research and Development, 2018. **9**(12): p. 1175-1180.
195. Bak, J., et al., *Mechanical restraint in psychiatry: Preventive factors in theory and practice. A Danish–Norwegian Association study.* Perspectives in psychiatric care, 2014. **50**(3): p. 155-166.

For peer review only - http://bmjopen.bmj.com/site/about/guidelines.xhtml

196. Bak, J., et al., *Comparing the effect of non-medical mechanical restraint preventive factors between psychiatric units in Denmark and Norway.* Nordic journal of psychiatry, 2015. **69**(6): p. 1715-1725.
197. Keski-Valkama, A., et al., *A 15-year national follow-up: legislation is not enough to reduce the use of seclusion and restraint.* Social Psychiatry and Psychiatric Epidemiology, 2007. **42**(9): p. 747-752.
198. Goulet, M.H., C. Larue, and A. Dumais, *Evaluation of seclusion and restraint reduction programs in mental health: A systematic review.* Aggression and Violent Behavior, 2017. **34**: p. 139-146.
199. Lee, S.W., Jane Sayer, Ann-Marie Parr, Richard Gray, Kevin Gournay, Soo, *Physical restraint training for nurses in English and Welsh psychiatric intensive care and regional secure units.* Journal of mental Health, 2001. **10**(2): p. 151-162.
200. Kontio, R., et al., *Nurses' and physicians' educational needs in seclusion and restraint practices.* Perspectives in psychiatric care, 2009. **45**(3): p. 198-207.
201. Hatta, K., et al., *Association between physical restraint and drug-induced liver injury.* Neuropsychobiology, 2007. **56**(4): p. 180-184.
202. Bowers, L., et al., *Student psychiatric nurses' approval of containment measures: relationship to perception of aggression and attitudes to personality disorder.* International Journal of Nursing Studies, 2007. **44**(3): p. 349-356.
203. Braham, L.G., J.F. Heasley, and S. Akiens, *An evaluation of night confinement in a high secure hospital.* Mental Health Review Journal, 2013. **18**(1): p. 21-31.
204. Chu, S., et al., *The impact of a night confinement policy on patients in a UK high secure inpatient mental health service.* Journal of Forensic Practice, 2015. **17**(1): p. 21-30.
205. Hottinen, A., et al., *Attitudes towards different containment measures: a questionnaire survey in Finnish adolescent psychiatry.* Journal of psychiatric and mental health nursing, 2012. **19**(6): p. 521-527.
206. Wharewera-Mika, J.P., et al., *Strategies to reduce the use of seclusion with tangata whai i te ora (Maori mental health service users).* International Journal of Mental Health Nursing, 2016. **25**(3): p. 258-265.
207. Haugom, E.W. and A. Granerud, *Shielding in Mental Health Hospitals: Description and Assessment by Staff.* SAGE Open, 2016. **6**(1): p. 2158244016629526.
208. Papadopoulos, C., et al., *Events preceding changes in conflict and containment rates on acute psychiatric wards.* Psychiatric Services, 2012. **63**(1): p. 40-47.
209. Looi, G.-M.E., Å. Engström, and S. Sävenstedt, *A self-destructive care: self-reports of people who experienced coercive measures and their suggestions for alternatives.* Issues in mental health nursing, 2015. **36**(2): p. 96-103.
210. Barr, L., D. Wynaden, and K. Heslop, *Promoting positive and safe care in forensic mental health inpatient settings: Evaluating critical factors that assist nurses to reduce the use of restrictive practices.* International Journal of Mental Health Nursing, 2019.
211. Bak, J. and H. Aggernæs, *Coercion within Danish psychiatry compared with 10 other European countries.* Nordic Journal of Psychiatry, 2012. **66**(5): p. 297-302.
212. Jaeger, M., et al., *Informal coercion in acute inpatient setting—Knowledge and attitudes held by mental health professionals.* Psychiatry research, 2014. **220**(3): p. 1007-1011.
213. Seo, M.K., S.H. Kim, and M. Rhee, *Coercion in psychiatric care: Can paternalism justify coercion?* International Journal of Social Psychiatry, 2012. **59**(3): p. 217-223.
214. Lovell, A., D. Smith, and P. Johnson, *A qualitative investigation into nurses' perceptions of factors influencing staff injuries sustained during physical interventions employed in response to service user violence within one secure learning disability service.* Journal of clinical nursing, 2015. **24**(13-14): p. 1926-1935.
215. Tateno, M., et al., *Attitude of young psychiatrists toward coercive measures in psychiatry: a case vignette study in Japan.* International journal of mental health systems, 2009. **3**(1): p. 20.

For peer review only - http://bmjopen.bmj.com/site/about/guidelines.xhtml

216. Elmer, T., et al., *Informal coercion as a neglected form of communication in psychiatric settings in Germany and Switzerland.* Psychiatry Research, 2018. **262**: p. 400-406.
217. Gustafsson, N. and M. Salzmann-Erikson, *Effect of Complex Working Conditions on Nurses Who Exert Coercive Measures in Forensic Psychiatric Care.* Journal of Psychosocial Nursing and Mental Health Services, 2016. **54**(9): p. 37-43.
218. Jalil, R., et al., *Mental health nurses' emotions, exposure to patient aggression, attitudes to and use of coercive measures: Cross sectional questionnaire survey.* International journal of nursing studies, 2017. **75**: p. 130-138.
219. Martello, M., et al., *Nurses' Perceptions of Engaging With Patients to Reduce Restrictive Practices in an Inpatient Psychiatric Unit.* Health Care Manager, 2018. **37**(4): p. 342-353.
220. McKeown, M., et al., *'Catching your tail and firefighting': the impact of staffing levels on restraint minimisation efforts.* Journal of Psychiatric and Mental Health Nursing, 2019.
221. Molewijk, B., et al., *Staff's normative attitudes towards coercion: the role of moral doubt and professional context-a cross-sectional survey study.* BMC medical ethics, 2017. **18**.
222. Raveesh, B., et al., *Staff and caregiver attitude to coercion in India.* Indian Journal of Psychiatry, 2016. **58**(6 Supplement 2): p. 221-229.
223. Georgieva, I., C. Mulder, and A. Wierdsma, *Patients' preference and experiences of forced medication and seclusion.* Psychiatric Quarterly, 2012. **83**(1): p. 1-13.
224. Haw, C., et al., *Coercive treatments in forensic psychiatry: a study of patients' experiences and preferences.* Journal of Forensic Psychiatry & Psychology, 2011. **22**(4): p. 564-585.
225. Sequeira, H. and S. Halstead, *"Is it meant to hurt, is it?" Management of violence in women with developmental disabilities.* Violence against women, 2001. **7**(4): p. 462-476.
226. Sustere, E. and E. Tarpey, *Least restrictive practice: its role in patient independence and recovery.* Journal of Forensic Psychiatry and Psychology, 2019.
227. Larsen, I.B. and T.B. Terkelsen, *Coercion in a locked psychiatric ward: Perspectives of patients and staff.* Nursing Ethics, 2014. **21**(4): p. 426-436.
228. Whittington, R., et al., *Approval ratings of inpatient coercive interventions in a national sample of mental health service users and staff in England.* Psychiatric Services, 2009. **60**(6): p. 792-798.
229. Reisch, T., et al., *Comparing Attitudes to Containment Measures of Patients, Health Care Professionals and Next of Kin.* Frontiers in Psychiatry, 2018. **9**.
230. Rippon, D., K. Reid, and G. Kay, *Views on restrictive practices on young people in psychiatric wards.* Nursing Times, 2018. **114**(7): p. 24-28.
231. Ryan, C. and L. Bowers, *Coercive manoeuvres in a psychiatric intensive care unit.* Journal of Psychiatric and Mental Health Nursing, 2005. **12**(6): p. 695-702.
232. Johnston, M.S. and J.M. Kilty, *"It's for their own good": Techniques of neutralization and security guard violence against psychiatric patients.* Punishment & Society, 2016. **18**(2): p. 177-197.
233. MacKay, I., B. Paterson, and C. Cassells, *Constant or special observations of inpatients presenting a risk of aggression or violence: nurses' perceptions of the rules of engagement.* Journal of Psychiatric and Mental Health Nursing, 2005. **12**(4): p. 464-471.
234. Delaney, K.R. and M.E. Johnson, *Keeping the unit safe: Mapping psychiatric nursing skills.* Journal of the American Psychiatric Nurses Association, 2006. **12**(4): p. 198-207.
235. Happell, B. and S. Koehn, *Seclusion as a necessary intervention: The relationship between burnout, job satisfaction and therapeutic optimism and justification for the use of seclusion.* Journal of advanced nursing, 2011. **67**(6): p. 1222-1231.

For peer review only - http://bmjopen.bmj.com/site/about/guidelines.xhtml

236. Johnson, M.E. and K.R. Delaney, *Keeping the unit safe: A grounded theory study.* Journal of the American Psychiatric Nurses Association, 2006. **12**(1): p. 13-21.
237. Jonker, E., et al., *Patient aggression in clinical psychiatry: perceptions of mental health nurses.* Journal of Psychiatric and Mental Health Nursing, 2008. **15**(6): p. 492-499.
238. Langan, C. and C. McDONALD, *Daytime night attire as a therapeutic intervention in an acute adult psychiatric in-patient unit.* Psychiatric Bulletin, 2008. **32**(6): p. 221-224.
239. Maguire, T., M. Daffern, and T. Martin, *Exploring nurses' and patients' perspectives of limit setting in a forensic mental health setting.* International journal of mental health nursing, 2014. **23**(2): p. 153-160.
240. Salzmann-Erikson, M., et al., *The core characteristics and nursing care activities in psychiatric intensive care units in Sweden.* International Journal of Mental Health Nursing, 2008. **17**(2): p. 98-107.
241. Millar, R. and N. Sands, *'He did what? Well that wasn't handed over!'Communicating risk in mental health.* Journal of psychiatric and mental health nursing, 2013. **20**(4): p. 345-354.
242. Sjöstrand, M., et al., *Ethical deliberations about involuntary treatment: interviews with Swedish psychiatrists.* BMC medical ethics, 2015. **16**(1): p. 37.
243. Woods, P., *Risk assessment and management approaches on mental health units.* Journal of psychiatric and mental health nursing, 2013. **20**(9): p. 807-813.
244. Cowan, D., et al., *Developing a guideline for structured content and process in mental health nursing handover.* International journal of mental health nursing, 2018. **27**(1): p. 429-439.
245. Cullen, S.W., S.B. Nath, and S.C. Marcus, *Toward understanding errors in inpatient psychiatry: a qualitative inquiry.* Psychiatric quarterly, 2010. **81**(3): p. 197-205.
246. Gifford, M.L. and J.E. Anderson, *Barriers and motivating factors in reporting incidents of assault in mental health care.* Journal of the American Psychiatric Nurses Association, 2010. **16**(5): p. 288-298.
247. Martin, T. and M. Daffern, *Clinician perceptions of personal safety and confidence to manage inpatient aggression in a forensic psychiatric setting.* Journal of psychiatric and mental health nursing, 2006. **13**(1): p. 90-99.
248. Mezey, G., Y. Hassell, and A. Bartlett, *Safety of women in mixed-sex and single-sex medium secure units: staff and patient perceptions.* The British Journal of Psychiatry, 2005. **187**(6): p. 579-582.
249. Brennan, G., C. Flood, and L. Bowers, *Constraints and blocks to change and improvement on acute psychiatric wards–lessons from the City Nurses project.* Journal of psychiatric and mental health nursing, 2006. **13**(5): p. 475-482.
250. Kuosmanen, A., et al., *Changes in patient safety culture: A patient safety intervention for Finnish forensic psychiatric hospital staff.* Journal of nursing management, 2019. **27**(4): p. 848-857.
251. Lavelle, M., et al., *Managing medical emergencies in mental health settings using an interprofessional in-situ simulation training programme: A mixed methods evaluation study.* Nurse education today, 2017. **59**: p. 103-109.
252. Bowers, L., et al., *Serious untoward incidents and their aftermath in acute inpatient psychiatry: The Tompkins Acute Ward Study.* International Journal of Mental Health Nursing, 2006. **15**(4): p. 226-234.
253. Gabrielsson, S., et al., *Knowledge of the patient as decision-making power: staff members' perceptions of interprofessional collaboration in challenging situations in psychiatric inpatient care.* Scandinavian journal of caring sciences, 2014. **28**(4): p. 784-792.
254. Ward, L., *Ready, aim fire! Mental health nurses under siege in acute inpatient facilities.* Issues in mental health nursing, 2013. **34**(4): p. 281-287.

For peer review only - http://bmjopen.bmj.com/site/about/guidelines.xhtml

255. Ryan, P., et al., *Demographic and situational variations in levels of burnout in European mental health services: a comparative study.* Diversity in Health and Social Care, 2007. **4**(2): p. 101-112.
256. Nathan, R., et al., *Staff responses to the therapeutic environment: A prospective study comparing burnout among nurses working on male and female wards in a medium secure unit.* The Journal of Forensic Psychiatry & Psychology, 2007. **18**(3): p. 342-352.
257. Vlayen, A., et al., *A nationwide Hospital Survey on Patient Safety Culture in Belgian hospitals: setting priorities at the launch of a 5-year patient safety plan*. 2012, BMJ Publishing Group Ltd.
258. Ajalli, A., et al., *Explanation of Patient Safety Provided by Nurses in Inpatient Psychiatric Wards in Iran: A Qualitative Study.* Iranian Journal of Psychiatry and Behavioral Sciences, 2018. **12**(4).
259. Wood, D. and N. Pistrang, *A safe place? Service users' experiences of an acute mental health ward.* Journal of Community & Applied Social Psychology, 2004. **14**(1): p. 16-28.
260. Jones, J., et al., *Psychiatric wards: places of safety?* Journal of psychiatric and mental health nursing, 2010. **17**(2): p. 124-130.
261. Ireland, C.A., et al., *Predicting security incidents in high secure male psychiatric care.* International Journal of Law and Psychiatry, 2019. **64**: p. 40-52.
262. Pelto-Piri, V., et al., *Feeling safe or unsafe in psychiatric inpatient care, a hospital-based qualitative interview study with inpatients in Sweden.* International Journal of Mental Health Systems, 2019. **13**(1): p. 23-23.
263. Haines, A., et al., *Factors impacting perceived safety among staff working on mental health wards.* Bjpsych Open, 2017. **3**(5): p. 204-211.
264. Kanerva, A., T. Kivinen, and J. Lammintakanen, *Communication elements supporting patient safety in psychiatric inpatient care.* Journal of psychiatric and mental health nursing, 2015. **22**(5): p. 298-305.
265. Kuosmanen, A., et al., *Patient safety culture in two Finnish state-run forensic psychiatric hospitals.* Journal of forensic nursing, 2013. **9**(4): p. 207-216.
266. Wu, J.-C., et al., *Determinants of workplace violence against clinical physicians in hospitals.* Journal of occupational health, 2015: p. 15-0111-OA.
267. Kanerva, A., J. Lammintakanen, and T. Kivinen, *Nursing Staff's Perceptions of Patient Safety in Psychiatric Inpatient Care.* Perspectives in psychiatric care, 2016. **52**(1): p. 25-31.
268. Stead, K., et al., *Teams communicating through STEPPS.* Medical Journal of Australia, 2009. **190**(S11): p. S128-S132.
269. Mahoney, J.S., et al., *Supporting a psychiatric hospital culture of safety.* Journal of the American Psychiatric Nurses Association, 2012. **18**(5): p. 299-306.
270. Higgins, N., et al., *Implementation of the Safewards model in public mental health facilities: A qualitative evaluation of staff perceptions.* International Journal of Nursing Studies, 2018. **88**: p. 114-120.
271. Bowers, L., K. Gournay, and D. Duffy, *Suicide and self-harm in inpatient psychiatric units: a national survey of observation policies.* Journal of Advanced Nursing, 2000. **32**(2): p. 437-444.
272. O'Brien, L. and R. Cole, *Mental health nursing practice in acute psychiatric close-observation areas.* International Journal of Mental Health Nursing, 2004. **13**(2): p. 89-99.
273. Stein, W., *The use of discharge risk assessment tools in general psychiatric services in the UK.* Journal of Psychiatric and Mental Health Nursing, 2002. **9**(6): p. 713-724.
274. Stübner, S., G. GROß, and N. Nedopil, *Inpatient risk management with mentally ill offenders: results of a survey on clinical decision-making about easing restrictions.* Criminal behaviour and mental health, 2006. **16**(2): p. 111-123.

For peer review only - http://bmjopen.bmj.com/site/about/guidelines.xhtml

275. O'Neill, C., et al., *Long-stay forensic psychiatric inpatients in the republic of Ireland: aggregated needs assessment.* Irish Journal of Psychological Medicine, 2003. **20**(4): p. 119-125.
276. Rees, P. and J. Manthorpe, *Managers' and staff experiences of adult protection allegations in mental health and learning disability residential services: a qualitative study.* British Journal of Social Work, 2009. **40**(2): p. 513-529.
277. Koukia, E., et al., *Security rules and banned items in psychiatric acute admission wards in Athens, Greece.* International journal of mental health nursing, 2010. **19**(6): p. 428-436.
278. Silvana, S., et al., *Ergonomics in the psychiatric ward towards workers or patients?* Work, 2012. **41**(Supplement 1): p. 1832-1835.
279. True, G., et al., *Adverse events in veterans affairs inpatient psychiatric units: Staff perspectives on contributing and protective factors.* General Hospital Psychiatry, 2017. **48**: p. 65-71.
280. Vandewalle, J., et al., *Patient safety on psychiatric wards: A cross-sectional, multilevel study of factors influencing nurses' willingness to share power and responsibility with patients.* International journal of mental health nursing, 2018. **27**(2): p. 877-890.
281. Gerace, A. and E. Muir-Cochrane, *Perceptions of nurses working with psychiatric consumers regarding the elimination of seclusion and restraint in psychiatric inpatient settings and emergency departments: An Australian survey.* International journal of mental health nursing, 2019. **28**(1): p. 209-225.
282. Vahidi, M., et al., *Therapeutic Relationships and Safety of Care in Iranian Psychiatric Inpatient Units.* Issues in Mental Health Nursing, 2018. **39**(11): p. 967-976.
283. Holth, F., et al., *Extreme challenges: psychiatric inpatients with severe self-harming behavior in Norway: a national screening investigation.* Nordic Journal of Psychiatry, 2018. **72**(8): p. 605-612.
284. de Jonghe-Rouleau, A.P., A.M. Pot, and J.F. de Jonghe, *Self-injurious behaviour in nursing home residents with dementia.* International Journal of Geriatric Psychiatry: A journal of the psychiatry of late life and allied sciences, 2005. **20**(7): p. 651-657.
285. Sansone, R.A., J.S. McLean, and M.W. Wiederman, *The relationship between medically self-sabotaging behaviors and borderline personality disorder among psychiatric inpatients.* Primary care companion to the Journal of clinical psychiatry, 2008. **10**(6): p. 448.
286. Gough, K. and A. Hawkins, *Staff attitudes to self-harm and its management in a forensic psychiatric service.* The British Journal of Forensic Practice, 2000. **2**(4): p. 22-28.
287. O'DONOVAN, A., *Pragmatism rules: the intervention and prevention strategies used by psychiatric nurses working with non-suicidal self-harming individuals.* Journal of Psychiatric and Mental Health Nursing, 2007. **14**(1): p. 64-71.
288. Tofthagen, R., A.-G. Talseth, and L. Fagerström, *Mental Health Nurses' Experiences of Caring for Patients Suffering from Self-Harm.* Nursing research and practice, 2014. **2014**.
289. Lundegaard Mattson, Å. and P.-E. Binder, *A qualitative exploration of how health care workers in an inpatient setting in Norway experience working with patients who self-injure.* Nordic Psychology, 2012. **64**(4): p. 272-290.
290. Thomas, J.B. and C.O. Haslam, *How people who self-harm negotiate the inpatient environment: the mental healthcare workers perspective.* Journal of Psychiatric & Mental Health Nursing (John Wiley & Sons, Inc.), 2017. **24**(7): p. 480-490.
291. Shaw, D.G. and P.T. Sandy, *Mental health nurses' attitudes toward self-harm: Curricular implications.* Health Sa Gesondheid, 2016. **21**.
292. Sandy, P.T., *The use of observation on patients who self-harm: Lessons from a learning disability service.* Health Sa Gesondheid, 2016. **21**.

For peer review only - http://bmjopen.bmj.com/site/about/guidelines.xhtml

293. James, K., et al., *Harm reduction as a strategy for supporting people who self-harm on mental health wards: the views and experiences of practitioners.* Journal of Affective Disorders, 2017. **214**: p. 67-73.
294. Brown, J. and N. Beail, *Self-harm among people with intellectual disabilities living in secure service provision: A qualitative exploration.* Journal of Applied Research in Intellectual Disabilities, 2009. **22**(6): p. 503-513.
295. Lindgren, B.-M., C. Aminoff, and U.H. Graneheim, *Features of everyday life in psychiatric inpatient care for self-harming: an observational study of six women.* Issues in mental health nursing, 2015. **36**(2): p. 82-88.
296. Weber, M.T., *Triggers for self-abuse: A qualitative study.* Archives of Psychiatric Nursing, 2002. **16**(3): p. 118-124.
297. Gibson, J., et al., *Dialectical behaviour therapy-informed skills training for deliberate self-harm: A controlled trial with 3-month follow-up data.* Behaviour research and therapy, 2014. **60**: p. 8-14.
298. Booth, R., et al., *Living through distress: a skills training group for reducing deliberate self-harm.* Behavioural and cognitive psychotherapy, 2014. **42**(2): p. 156-165.
299. Bowers, L., et al., *Relationship between service ecology, special observation and self-harm during acute in-patient care: City-128 study.* The British Journal of Psychiatry, 2008. **193**(5): p. 395-401.
300. Kool, N., et al., *Improving communication and practical skills in working with inpatients who self-harm: a pre-test/post-test study of the effects of a training programme.* BMC psychiatry, 2014. **14**(1): p. 64.
301. Drew, B.L., *No-suicide contracts to prevent suicidal behavior in inpatient psychiatric settings.* Journal of the American Psychiatric Nurses Association, 1999. **5**(1): p. 23-28.
302. Sjöström, N., J. Hetta, and M. Waern, *Sense of coherence and suicidality in suicide attempters: a prospective study.* Journal of psychiatric and mental health nursing, 2012. **19**(1): p. 62-69.
303. Swogger, M.T., K.A. Van Orden, and K.R. Conner, *The relationship of outwardly directed aggression to suicidal ideation and suicide attempts across two high-risk samples.* Psychology of violence, 2014. **4**(2): p. 184.
304. Hill, R.M., et al., *The Columbia-Suicide Severity Rating Scale: Associations between interrupted, aborted, and actual suicide attempts among adolescent inpatients.* Psychiatry research, 2017. **255**: p. 338-340.
305. Inoue, K., et al., *A large-scale survey of inpatient suicides: comparison between medical and psychiatric settings.* Psychiatry research, 2017. **250**: p. 155-158.
306. Ellis, T.E., et al., *Collaborative Assessment and Management of Suicidality in an inpatient setting: Results of a pilot study.* Psychotherapy, 2012. **49**(1): p. 72.
307. Ellis, T.E., et al., *Impact of a suicide-specific intervention within inpatient psychiatric care: The Collaborative Assessment and Management of Suicidality.* Suicide and Life-Threatening Behavior, 2015. **45**(5): p. 556-566.
308. Awenat, Y.F., et al., *A qualitative analysis of suicidal psychiatric inpatients views and expectations of psychological therapy to counter suicidal thoughts, acts and deaths 11 Medical and Health Sciences 1117 Public Health and Health Services 11 Medical and Health Sciences 1103 Clinical Sciences 17 Psychology and Cognitive Sciences 1701 Psychology.* Bmc Psychiatry, 2018. **18**(1): p. 334-334.
309. Cleary, M., et al., *Suicidal patients and special observation.* Journal of psychiatric and mental health nursing, 1999. **6**(6): p. 461-467.
310. Takahashi, C., et al., *The impact of inpatient suicide on psychiatric nurses and their need for support.* BMC psychiatry, 2011. **11**(1): p. 38.
311. Vråle, G. and E. Steen, *The dynamics between structure and flexibility in constant observation of psychiatric inpatients with suicidal ideation.* Journal of Psychiatric and Mental Health Nursing, 2005. **12**(5): p. 513-518.

For peer review only - http://bmjopen.bmj.com/site/about/guidelines.xhtml

312. Vandewalle, J., et al., *'promoting and preserving safety and a life-oriented perspective': A qualitative study of nurses' interactions with patients experiencing suicidal ideation.* International Journal of Mental Health Nursing, 2019: p. No-Specified.
313. Davis, S.E., I.S. Williams, and L.W. Hays, *Psychiatric inpatients' perceptions of written no-suicide agreements: An exploratory study.* Suicide and Life-Threatening Behavior, 2002. **32**(1): p. 51-66.
314. Esposito-Smythers, C., T.J. McClung, and A.M. Fairlie, *Adolescent perceptions of a suicide prevention group on an inpatient unit.* Archives of suicide research, 2006. **10**(3): p. 265-275.
315. SUN, F.K., et al., *Nursing people who are suicidal on psychiatric wards in Taiwan: action/interaction strategies.* Journal of psychiatric and mental health nursing, 2005. **12**(3): p. 275-282.
316. Sun, F.K., et al., *Patients and nurses' perceptions of ward environmental factors and support systems in the care of suicidal patients.* Journal of clinical nursing, 2006. **15**(1): p. 83-92.
317. Pfeiffer, P.N., et al., *Development and pilot study of a suicide prevention intervention delivered by peer support specialists.* Psychological Services, 2018: p. No-Specified.
318. Caspi, E., *Does self-neglect occur among older adults with dementia when unsupervised in assisted living? An exploratory, observational study.* Journal of elder abuse & neglect, 2014. **26**(2): p. 123-149.
319. Bowers, L., et al., *Locked doors: a survey of patients, staff and visitors.* Journal of Psychiatric and Mental Health Nursing, 2010. **17**(10): p. 873-880.
320. Haglund, K. and L. von Essen, *Locked entrance doors at psychiatric wards–advantages and disadvantages according to voluntarily admitted patients.* Nordic journal of psychiatry, 2005. **59**(6): p. 511-515.
321. Muir-Cochrane, E., et al., *Investigation into the acceptability of door locking to staff, patients, and visitors on acute psychiatric wards.* International journal of mental health nursing, 2012. **21**(1): p. 41-49.
322. Cowman, S. and L. Bowers, *Safety and security in acute admission psychiatric wards in Ireland and London: a comparative study.* Journal of clinical nursing, 2009. **18**(9): p. 1346-1353.
323. Simpson, A., et al., *The relationship between substance use and exit security on psychiatric wards.* Journal of advanced nursing, 2011. **67**(3): p. 519-530.
324. Kalagi, J., et al., *Requirements for the implementation of open door policies in acute psychiatry from a mental health professionals' and patients' view: A qualitative interview study 11 Medical and Health Sciences 1117 Public Health and Health Services.* BMC Psychiatry, 2018. **18**(1): p. 304.
325. Fletcher, J., et al., *Working towards least restrictive environments in acute mental health wards in the context of locked door policy and practice.* International journal of mental health nursing, 2019. **28**(2): p. 538-550.
326. Van der Schaaf, P., et al., *Impact of the physical environment of psychiatric wards on the use of seclusion.* The British Journal of Psychiatry, 2013. **202**(2): p. 142-149.
327. Verbeek, H., et al., *Effects of small-scale, home-like facilities in dementia care on residents' behavior, and use of physical restraints and psychotropic drugs: a quasi-experimental study.* International psychogeriatrics, 2014. **26**(4): p. 657-668.
328. Curtis, S., et al., *Compassionate containment? Balancing technical safety and therapy in the design of psychiatric wards.* Social Science & Medicine, 2013. **97**: p. 201-209.
329. Dreyfus, S., L. Phillipson, and R. Fleming, *Staff and family attitudes to fences as a means of detaining people with dementia in residential aged care settings: The tension between physical and emotional safety.* Australian Journal of Social Issues, 2018. **53**(2): p. 107-122.

For peer review only - http://bmjopen.bmj.com/site/about/guidelines.xhtml

330. Bayramzadeh, S., *An Assessment of Levels of Safety in Psychiatric Units.* HERD, 2017. **10**(2): p. 66-80.
331. Bellantonio, S., et al., *Efficacy of a geriatrics team intervention for residents in dementia-specific assisted living facilities: Effect on unanticipated transitions.* Journal of the American Geriatrics Society, 2008. **56**(3): p. 523-528.
332. Chandler, G., *From traditional inpatient to trauma-informed treatment: Transferring control from staff to patient.* Journal of the American Psychiatric Nurses Association, 2008. **14**(5): p. 363-371.
333. Wilkes, L., et al., *Environmental approach to reducing agitation in older persons with dementia in a nursing home.* Australasian Journal on Ageing, 2005. **24**(3): p. 141-145.
334. Gebhardt, R. and T. Steinert, *Should severely disturbed psychiatric patients be distributed or concentrated in specialized wards? An empirical study on the effects of hospital organization on ward atmosphere, aggressive behavior, and sexual molestation.* European psychiatry, 1999. **14**(5): p. 291-297.
335. Stolker, J.J., H.L. Nijman, and P.-H. Zwanikken, *Are patients' views on seclusion associated with lack of privacy in the ward?* Archives of psychiatric nursing, 2006. **20**(6): p. 282-287.
336. Kulkarni, J., et al., *Establishing female-only areas in psychiatry wards to improve safety and quality of care for women.* Australasian Psychiatry, 2014. **22**(6): p. 551-556.
337. Bowers, L. and M. Crowder, *Nursing staff numbers and their relationship to conflict and containment rates on psychiatric wards—A cross sectional time series Poisson regression study.* International Journal of Nursing Studies, 2012. **49**(1): p. 15-20.
338. Triplett, P., et al., *The Milieu Manager: A Nursing Staffing Strategy to Reduce Observer Use in the Acute Psychiatric Inpatient Setting.* Journal of the American Psychiatric Nurses Association, 2017. **23**(6): p. 422-430.
339. Hunt, I.M., et al., *Ligature points and ligature types used by psychiatric inpatients who die by hanging.* Crisis, 2012.
340. Ružić, K., et al., *Aggresiveness in institutionalised schizophrenic patients and the selection of antipsychotics.* Collegium antropologicum, 2011. **35**(1): p. 265-269.
341. Rodríguez-Leal, C.M., et al., *Electrocardiographic surveillance in a psychiatric institution: avoiding iatrogenic cardiovascular death.* International journal of psychiatry in clinical practice, 2017. **21**(1): p. 64-66.
342. Seemüller, F., et al., *The controversial link between antidepressants and suicidality risks in adults: data from a naturalistic study on a large sample of in-patients with a major depressive episode.* International journal of neuropsychopharmacology, 2009. **12**(2): p. 181-189.
343. Haw, C., J. Stubbs, and G.L. Dickens, *Barriers to the reporting of medication administration errors and near misses: an interview study of nurses at a psychiatric hospital.* Journal of Psychiatric and Mental Health Nursing, 2014. **21**(9): p. 797-805.
344. Soerensen, A.L., et al., *The medication process in a psychiatric hospital: are errors a potential threat to patient safety?* Risk management and healthcare policy, 2013. **6**: p. 23.
345. Keers, R.N., et al., *What causes medication administration errors in a mental health hospital? A qualitative study with nursing staff.* Plos One, 2018. **13**(10): p. e0206233-e0206233.
346. Bademli, K. and K. Buldukoglu, *Oral medication management in inpatient psychiatric care in Turkey.* Journal of psychiatric and mental health nursing, 2009. **16**(4): p. 355-362.
347. Prins, M.C., et al., *Use of a structured medication history to establish medication use at admission to an old age psychiatric clinic: a prospective observational study.* CNS drugs, 2013. **27**(11): p. 963-969.

For peer review only - http://bmjopen.bmj.com/site/about/guidelines.xhtml

348. Xie, N., et al., *Understanding Mental Health Nurses' Perceptions of Barcode Medication Administration: A Qualitative Descriptive Study.* Issues in Mental Health Nursing, 2019. **40**(4): p. 326-334.
349. Strudwick, G., et al., *Thank you for asking: Exploring patient perceptions of barcode medication administration identification practices in inpatient mental health settings.* International Journal of Medical Informatics, 2017. **105**: p. 31-37.
350. Cottney, A. and J. Innes, *Medication-administration errors in an urban mental health hospital: A direct observation study.* International journal of mental health nursing, 2015. **24**(1): p. 65-74.
351. Dickens, G., J. Stubbs, and C. Raw, *Delegation of medication administration: an exploratory study.* Nursing Standard, 2008. **22**(22).
352. Haw, C., J. Stubbs, and G. Dickens, *An observational study of medication administration errors in old-age psychiatric inpatients.* International Journal for Quality in Health Care, 2007. **19**(4): p. 210-216.
353. Cottney, A., *Improving the safety and efficiency of nurse medication rounds through the introduction of an automated dispensing cabinet.* BMJ Open Quality, 2014. **3**(1): p. u204237. w1843.
354. Dolan, M. and H. Kirwan, *Survey of staff perceptions of illicit drug use among patients in a medium secure unit.* Psychiatric Bulletin, 2001. **25**(1): p. 14-17.
355. Hughes, E., et al., *Novel psychoactive substance use by mental health service consumers: an online survey of inpatient health professionals' views and experiences.* Advances in Dual Diagnosis, 2018. **11**(1): p. 30-39.
356. Gonzalez-Pinto, A. and S.R. De Azua, *S.2.04 Adherence to treatment in bipolar disorders.* European Neuropsychopharmacology, 2011. **21**: p. S108-S109.
357. Meehan, T., P. Morrison, and S. McDougall, *Absconding behaviour: an exploratory investigation in an acute inpatient unit.* Australian & New Zealand Journal of Psychiatry, 1999. **33**(4): p. 533-537.
358. Muir-Cochrane, E., et al., *The inpatient psychiatric unit as both a safe and unsafe place: Implications for absconding.* International Journal of Mental Health Nursing, 2013. **22**(4): p. 304-312.
359. Nurjannah, I., M. FitzGerald, and K. Foster, *Patients' experiences of absconding from a psychiatric setting in Indonesia.* International journal of mental health nursing, 2009. **18**(5): p. 326-335.
360. Grotto, J., et al., *Risk assessment and absconding: perceptions, understandings and responses of mental health nurses.* Journal of clinical nursing, 2015. **24**(5-6): p. 855-865.
361. Bowers, L., J. Alexander, and C. Gaskell, *A trial of an anti-absconding intervention in acute psychiatric wards.* Journal of Psychiatric and Mental Health Nursing, 2003. **10**(4): p. 410-416.
362. Simpson, A.I., et al., *The impact of structured decision making on absconding by forensic psychiatric patients: results from an AB design study.* BMC psychiatry, 2015. **15**(1): p. 103.
363. Algase, D.L., et al., *Wandering and the physical environment.* American Journal of Alzheimer's Disease & Other Dementias®, 2010. **25**(4): p. 340-346.
364. Colombo, M., et al., *Wanderers: features, findings, issues.* Archives of gerontology and geriatrics, 2001. **33**: p. 99-106.
365. Hunt, I.M., et al., *Suicide after absconding from inpatient care in England: an exploration of mental health professionals' experiences.* Journal of Mental Health, 2016. **25**(3): p. 245-253.
366. Hunt, I.M., et al., *Suicide amongst psychiatric in-patients who abscond from the ward: a national clinical survey.* BMC psychiatry, 2010. **10**(1): p. 14.

For peer review only - http://bmjopen.bmj.com/site/about/guidelines.xhtml

367. Nijman, H., et al., *Door locking and exit security measures on acute psychiatric admission wards.* Journal of Psychiatric and Mental Health Nursing, 2011. **18**(7): p. 614-621.
368. Whaley, A.L., *A two-stage method for the study of cultural bias in the diagnosis of schizophrenia in African Americans.* Journal of Black Psychology, 2004. **30**(2): p. 167-186.
369. Whaley, A.L., *Cultural mistrust and the clinical diagnosis of paranoid schizophrenia in African American patients.* Journal of Psychopathology and Behavioral Assessment, 2001. **23**(2): p. 93-100.
370. Green, R., et al., *Implementing seclusion in forensic mental health care: A qualitative study of staff decision making.* Archives of Psychiatric Nursing, 2018: p. No-Specified.
371. Koukia, E., M.G. Madianos, and T. Katostaras, *"On the spot" interventions by mental health nurses in inpatient psychiatric wards in Greece.* Issues in mental health nursing, 2009. **30**(5): p. 327-336.
372. Lindsey, P.L., *Psychiatric nurses' decision to restrain: the association between empowerment and individual factors.* Journal of psychosocial nursing and mental health services, 2009. **47**(9): p. 41-49.
373. Mann-Poll, P.S., et al., *Factors contributing to mental health professionals' decision to use seclusion.* Psychiatric Services, 2011. **62**(5): p. 498-503.
374. Marangos-Frost, S. and D. Wells, *Psychiatric nurses' thoughts and feelings about restraint use: a decision dilemma.* Journal of advanced nursing, 2000. **31**(2): p. 362-369.
375. Brown, B. and T. Rakow, *Understanding clinicians' use of cues when assessing the future risk of violence: a clinical judgement analysis in the psychiatric setting.* Clinical psychology & psychotherapy, 2016. **23**(2): p. 125-141.
376. Fuller, J. and J. Cowan, *Risk assessment in a multi-disciplinary forensic setting: Clinical judgement revisited.* The Journal of Forensic Psychiatry, 1999. **10**(2): p. 276-289.
377. Abraham, S., *Managing patient falls in psychiatric inpatient units: Part 1.* The health care manager, 2016. **35**(1): p. 21-27.
378. Fonad, E., et al., *Falls in somatic and dementia wards at Community Care Units.* Scandinavian journal of caring sciences, 2009. **23**(1): p. 2-10.
379. Tängman, S., et al., *Precipitating factors for falls among patients with dementia on a psychogeriatric ward.* International psychogeriatrics, 2010. **22**(4): p. 641-649.
380. Garfinkel, D., et al., *High efficacy for hip protectors in the prevention of hip fractures among elderly people with dementia.* Journal of the American Medical Directors Association, 2008. **9**(5): p. 313-318.
381. Holmes, D., et al., *An evaluation of a monitoring system intervention: falls, injuries, and affect in nursing homes.* Clinical nursing research, 2007. **16**(4): p. 317-335.
382. Powell-Cope, G., et al., *A qualitative understanding of patient falls in inpatient mental health units.* Journal of the American Psychiatric Nurses Association, 2014. **20**(5): p. 328-339.
383. Li, P.H., et al., *Infection preventionists' challenges in psychiatric clinical settings.* American Journal of Infection Control, 2019. **47**(2): p. 123-127.
384. D'Lima, D., et al., *Patient safety and quality of care in mental health: a world of its own?* BJPsych bulletin, 2017. **41**(5): p. 241-243.
385. Sakinofsky, I., *Preventing suicide among inpatients.* The Canadian journal of psychiatry, 2014. **59**(3): p. 131-140.
386. Leape, L., et al., *Transforming healthcare: a safety imperative.* BMJ Quality & Safety, 2009. **18**(6): p. 424-428.
387. Gandhi, T.K., et al., *Transforming concepts in patient safety: a progress report.* BMJ Qual Saf, 2018. **27**(12): p. 1019-1026.

For peer review only - http://bmjopen.bmj.com/site/about/guidelines.xhtml

388. Schwappach, D.L.B., *Review: Engaging Patients as Vigilant Partners in Safety:A Systematic Review.* Medical Care Research and Review, 2010. **67**(2): p. 119-148.
389. Davis, R.E., N. Sevdalis, and C.A. Vincent, *Patient involvement in patient safety: How willing are patients to participate?* BMJ Quality & Safety, 2011. **20**(1): p. 108-114.
390. Institute, N.P.S.F.s.L.L., *Safety is personal: partnering with patients and families for the safest care*. 2014, Moston: MA.: National Patient Safety Foundation.
391. Providers, N., *Funding for mental health at a local level: Unpicking the variation.* NHS Providers, 2016.
392. Prince, M., et al., *No health without mental health.* The lancet, 2007. **370**(9590): p. 859-877.
393. Unsworth, J., M. McKeever, and M. Kelleher, *Recognition of physical deterioration in patients with mental health problems: the role of simulation in knowledge and skill development.* Journal of Psychiatric and Mental Health Nursing, 2012. **19**(6): p. 536-545.
394. Cooper, J., et al., *The National Patient Safety Foundation agenda for research and development in patient safety.* MedGenMed: Medscape general medicine, 2000. **2**(3): p. E38-E38.
395. Institute of Medicine Committee on Quality of Health Care in, A., in *To Err is Human: Building a Safer Health System*, L.T. Kohn, J.M. Corrigan, and M.S. Donaldson, Editors. 2000, National Academies Press (US)

Copyright 2000 by the National Academy of Sciences. All rights reserved.: Washington (DC).
396. Emanuel, L., et al., *What exactly is patient safety?*, in *Advances in patient safety: new directions and alternative approaches (Vol. 1: Assessment)*. 2008, Agency for Healthcare Research and Quality.
397. Hox, J.J. and H.R. Boeije, *Data collection, primary versus secondary.* 2005.
398. Appleby, L., J. Shaw, and T. Amos, *National confidential inquiry into suicide and homicide by people with mental illness.* The British Journal of Psychiatry, 1997. **170**(2): p. 101-102.
399. Elliott, J.H., et al., *Living systematic reviews: an emerging opportunity to narrow the evidence-practice gap.* PLoS medicine, 2014. **11**(2): p. e1001603.
400. Long, L., *Routine piloting in systematic reviews—a modified approach?* Systematic reviews, 2014. **3**(1): p. 77.
401. Landis, J.R. and G.G. Koch, *The measurement of observer agreement for categorical data.* biometrics, 1977: p. 159-174.
402. Gopalakrishnan, S. and P. Ganeshkumar, *Systematic reviews and meta-analysis: understanding the best evidence in primary healthcare.* Journal of family medicine and primary care, 2013. **2**(1): p. 9.
403. Dewa, L.H., et al., *Identifying research priorities for patient safety in mental health: an international expert Delphi study.* BMJ open, 2018. **8**(3): p. e021361.

For peer review only - http://bmjopen.bmj.com/site/about/guidelines.xhtml

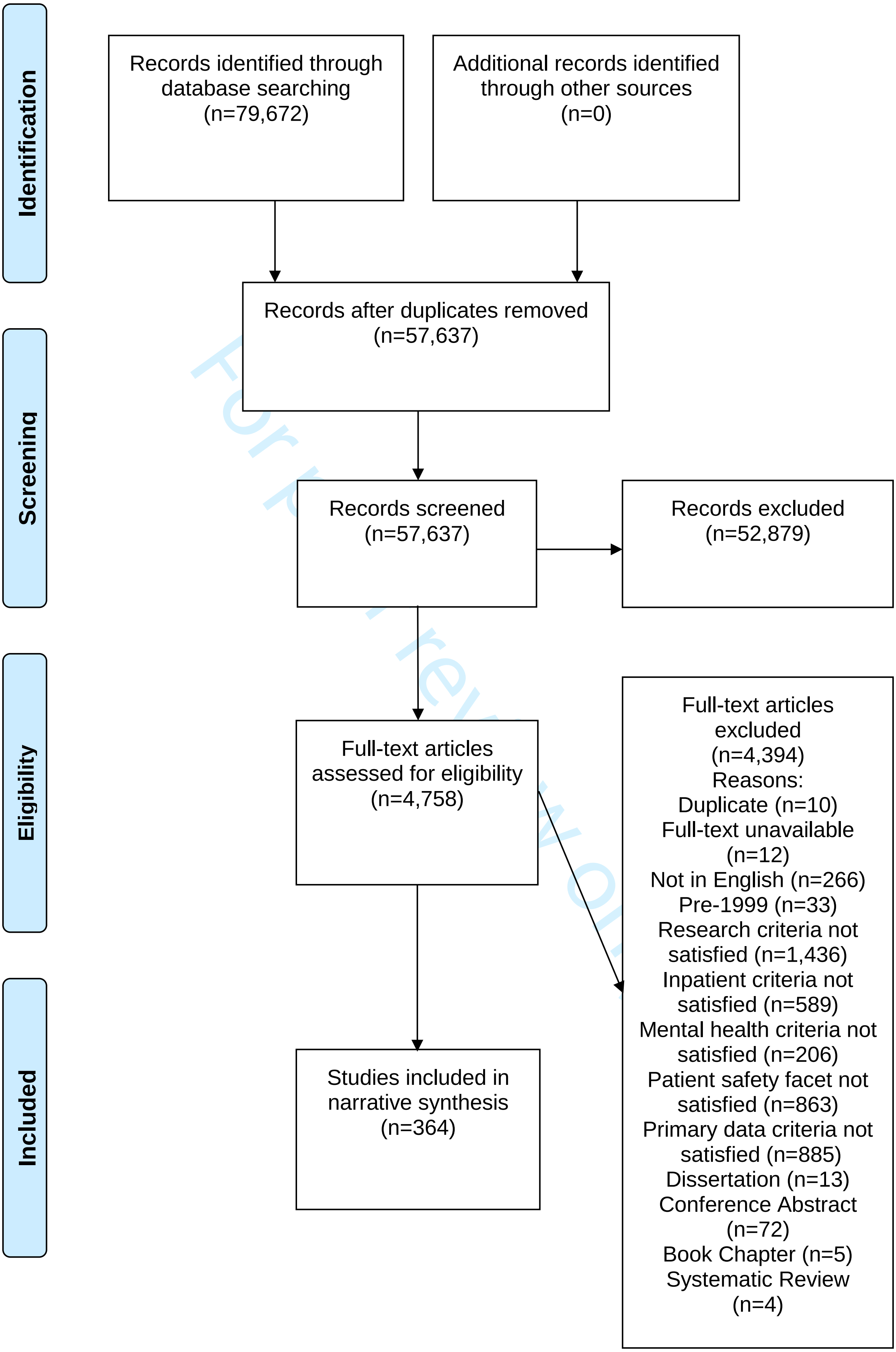

Figure 1: Flowchart of studies

Online supplement 1

Development of inclusion and exclusion criteria for screening

Facets

- Research
- Patient Safety
- Mental Health
- Inpatient Setting

Each one of these becomes an ***inclusion criteria*** by having an agreed definition across the research team.

Good agreement was found between the two researcher's judgements $\kappa = 0.72$ (95% CI, 0.69 to 0.74), $p < .0005$. Disagreements were discussed until consensus was reached. Subsequently, two independent reviewers screened the full text articles of the abstracts that indicated that the paper met, or could meet, the inclusion criteria. Agreement rates were calculated using the kappa statistic. Substantial agreement was reached (k=0.64 (95% CI, 0.55 to 0.73), $p < .0005$).

- Mental Health (defined as a field comprising various professions, such as psychiatry and social work, that deals with the promotion of mental and psychological well-being and the prevention, diagnosis, or treatment of mental disorders as listed in the Diagnostic and Statistical Manual 5th edition (American Psychiatric Association, 2013))
- Patient Safety (defined as "The avoidance, prevention and amelioration of adverse outcomes or injuries stemming from the process of healthcare" (Vincent, 2006))
- Research (defined as diligent and systematic inquiry or investigation into a subject in order to discover or revise facts, theories, applications etc.)
- Inpatient Setting (defined as hospital settings which provide continuous care for a period of over 24 hours)

Inclusion Criteria:

- Interventions/outcomes- Articles that report patient safety outcome measures.

Exclusion Criteria:

- Population- Articles that use the words 'forensic', 'secure', 'closed' or 'locked' can be taken to refer to an inpatient environment.

- Population- Articles that amalgamate data from both inpatient and outpatient settings such that data for an inpatient only sample is not available.

- Interventions/outcomes- Articles that solely examine the reliability or validity of risk assessment tools, with no relation to the management of the risk that the tool is measuring.

- Interventions/outcomes- Articles that do not measure patient safety outcomes (this aspect needs to be overtly expressed in the article, as an aim or research question).

For peer review only

- Interventions/outcomes- Clinical effectiveness versus patient safety. Clinical effectiveness concerns providing the best possible treatment for patients (e.g. with effective drug treatment) as opposed to being a patient safety issue. To be included, the treatment being studied has to be linked to a patient safety outcome/measurement (like a behaviour or adverse event).

- Interventions/outcomes- Articles reporting staff perceptions/feelings concerning safety related issues are only to be included when directly related to, or discussed in terms of, a patient safety outcome/measurement (like a behaviour or adverse event).

- Settings- Articles that are not based on purely mental health care settings (i.e. exclude general hospital wards and nursing homes that do not specify mental health specialised wards). Mental health needs to be separately examined and linked to patient safety.

- Settings- Articles based in primary care, community or social care settings.

- Settings- Articles that report on patient safety events/outcomes that occur outside of the inpatient setting.

Other general exclusion criteria:

- Articles that are reviews of any kind (including literature and systematic reviews). These will be excluded, but the reference lists will be reviewed and cross-referenced to ensure we have included all relevant articles that were used in data synthesis. Will see if grads can assist with this part of the process.

- Articles that are not empirical research; i.e. articles that do not have clearly defined hypotheses, research questions or aims that generate new primary data (data being collected for the purposes of the research). Secondary data is to be excluded, even if a new analysis has been done on it. Look for results and method sections here to ensure there were clear aims and that the data produced relates to those aims. For quantitative studies, there should be hypotheses and for qualitative studies, there should be clear aims or a research question.

- Opinion/editorials/commentaries (save any information we come across for developing our priorities in line with the expert consensus study)

- Articles not in English

- Conference abstracts

- Audits (this is not new data but routinely collected data).

- Case studies based on individual patients and their clinical presentation should be excluded. Inclusion of case study in the research facet is to pick up case studies based on health organisations or countries etc. (surrounding mental health and patient safety) not individual people.

- Reports and book abstracts

For peer review only

- Dissertations (not peer-reviewed).
- ‘Snapshot’ studies that provide clinical pictures of a patient group/setting (no new data).
- Epidemiological studies. Studies describing mental health conditions or the difference between similar conditions within an inpatient setting does not satisfy the patient safety facet (there is no management of patient safety).
- Ambiguities that prevent researchers from confirming any criteria (not enough resource available to contact researchers).

The process for reviewing full-text articles

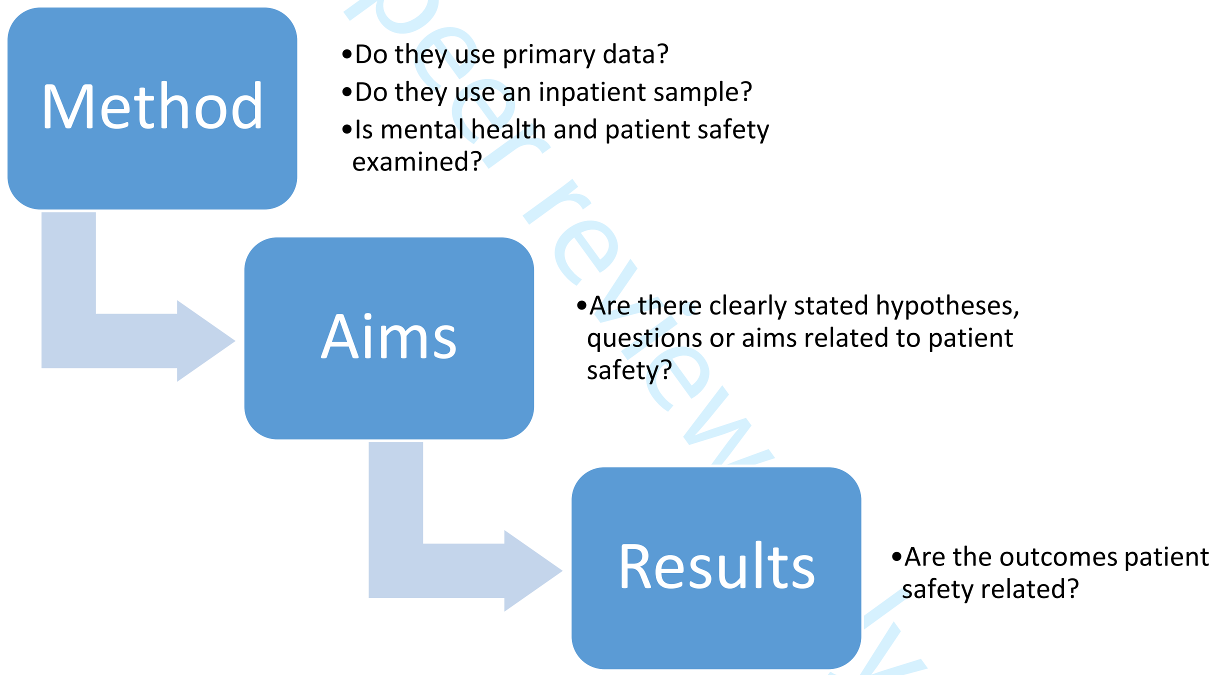

For peer review only

Online supplement 2

Full search strategy

Search term
Mental health.ts.
Mental well-being.ts.
Mental well being.ts.
Psychological well being.ts.
Psychological well-being.ts.
Mental disorder*.ts
Mental illness*.ts.
Mental disease*.ts.
Psychiatr*.ts.
Anxiety disorder*.ts.
Delirium.ts.
Dementia.ts.
Dissociative disorder*.ts.
Factitious disorder*.ts.
Impulse control disorder*.ts.
Mood disorder*.ts.
Affective disorder*.ts.
Psychotic disorder*.ts.
Depressive disorder*.ts.
Neurotic disorder*.ts.
Personality disorder*.ts.
Conduct disorder*.ts.
Schizophreni*.ts.
Somatoform disorder*.ts.
Substance related disorder*.ts.
Clinical Psychology.ts.

Impulsive behavio?r.ts.
Adjustment disorder*.ts.
Eating disorder*.ts.
Sleep disorder*.ts.
Neuros?s.ts.
Psychos?s.ts.
Delusion*.ts.
Paranoia.ts.
Hallucination*.ts.
Addiction*.ts.
Dependence.ts.
Misuse.ts.
New psychoactive substance*.ts.
Legal high*.ts.
Depression.ts.
Panic disorder*.ts.
Phobia*.ts.
Health anxiet*.ts.
Bipolar disorder*.ts.
Alcohol abuse.ts.
Alcoholism.ts.
Obsessive compulsive disorder*.ts.
Obsessive thought*.ts.
Intrusive thought*.ts.
Post traumatic stress disorder*.ts.
Post-traumatic stress disorder*.ts.
Cognitive Behavio?ral Therap*.ts.
Psychotherap*.ts.
Person centred therap*.ts.

Person-centred therap*.ts.
Counselling.ts.
Antidepressant medication*.ts.
Antipsychotic medication*.ts.
Antianxiety medication*.ts.
Psychotropic medication*.ts.
Mindfulness based cognitive therap*.ts.
Mindfulness-based cognitive therap*.ts.
Mindfulness based relapse prevention.ts.
Mindfulness-based relapse prevention.ts.
Mindfulness based stress reduction.ts.
Mindfulness-based stress reduction.ts.
Electroconvulsive therap*.ts.
Verbal de-escalation.ts.
Therapeutic.ts.
Functional Analys?s.ts.
Dialectical Behavio?r Therap*.ts.
Dysexecutive syndrome.ts.
ALL MENTAL HEALTH TERMS COMBINED WITH OR (MENTAL HEALTH FACET.ts.)
Patient safety.ts.
Adverse event*.ts.
Adverse drug event*.ts.
Sentinel event*.ts.
Incident*.ts.
Error*.ts.
Near miss*.ts.
Close call*.ts.
Never event*.ts.
Critical outcome*.ts.

Adverse outcome*.ts.
Unanticipated outcome*.ts.
Suicide*.ts.
Self-harm.ts.
Self harm.ts.
Behavio?r control.ts.
Restraint.ts.
Seclusion.ts.
Safety management.ts.
Failure to diagnose.ts.
Failure of diagnos?s.ts.
Under diagnosis.ts.
Over diagnosis.ts.
Misdiagnosis.ts.
Dual diagnos?s.ts.
Delay in diagnos?s.ts.
Wrong diagnos?s.ts.
Incorrect diagnos?s.ts.
Safety culture.ts.
Safety climate.ts.
Fall*.ts.
Slip*.ts.
Trip*.ts.
Accident prevention.ts.
Patient accident*.ts.
Patient in road traffic accident*.ts.
Collision with an object.ts.
Contact with an object.ts.
Contact with sharp*.ts.

Collision with sharp*.ts.
Exposure to hazardous substance*.ts.
Inappropriate patient handling.ts.
Inappropriate patient positioning.ts.
Elope.ts.
Wander.ts.
Runaway.ts.
Abscond*.ts.
Escorted leave.ts.
Unescorted leave.ts.
Aggressi*.ts.
Violence.ts.
Assault*.ts.
Abus*.ts.
Disruptive behavio?r.ts.
Racial attack*.ts.
Sexual attack*.ts.
Sexually inappropriate.ts.
Physical attack*.ts.
Verbal attack*.ts.
Missing patient*.ts.
Failure in access.ts.
Unexpected readmission*.ts.
Reattendance*.ts.
Unplanned admission*.ts.
Transfer to specialist care unit*.ts.
Delay in discharge.ts.
Failure to discharge.ts.
Inappropriate discharge.ts.

Planning failure.ts.
Self discharge.ts.
Self-discharge.ts.
Discharge against medical advice.ts.
Failure in referral process*.ts.
Failure to return from authorised leave.ts.
Transfer delay*.ts.
Transfer failure*.ts.
Inappropriate transfer*.ts.
Unsafe transfer*.ts.
Unsafe clinical environment*.ts.
Inappropriate clinical environment*.ts.
Inappropriate admission of a minor to an adult setting.ts.
Inappropriate transfer of a minor to an adult setting.ts.
Poor clinical assessment*.ts.
Lack of clinical assessment*.ts.
Lack of risk assessment*.ts.
Wrong scan*.ts.
Wrong x-ray*.ts.
Wrong specimen*.ts.
Inadequate scan*.ts.
Inadequate x-ray*.ts.
Inadequate specimen*.ts.
Incomplete scan*.ts.
Incomplete x-ray*.ts.
Incomplete specimen*.ts.
Mislabelled scan*.ts.
Mislabelled x-ray*.ts.
Mislabelled specimen*.ts.

Unlabelled scan*.ts.
Unlabelled x-ray*.ts.
Unlabelled specimen*.ts.
Missing scan*.ts.
Missing x-ray*.ts.
Missing specimen*.ts.
Failure to interpret test result*.ts.
Delay to interpret test result*.ts.
Failure to act on test result*.ts.
Delay to act on test result*.ts.
Failure to receive test result*.ts.
Delay to receive test result*.ts.
Incorrect test result*.ts.
Incorrect report*.ts.
Missing test result*.ts.
Missing report*.ts.
Failure to undertake test*.ts.
Delay to undertake test*.ts.
Patient confidentiality.ts.
Communication failure*.ts.
Failed communication*.ts.
Failure in communication*.ts.
Failure to receive informed consent.ts.
Inadequate handover.ts.
Documentation delay*.ts.
Mislabelled documentation.ts.
Missing documentation.ts.
Inadequate documentation.ts.
Wrong documentation.ts.

Illegible documentation.ts.
Mislabelled healthcare record*.ts.
Inadequate healthcare record*.ts.
Missing healthcare record*.ts.
Wrong healthcare record*.ts.
Illegible healthcare record*.ts.
Mislabelled referral letter*.ts.
Inadequate referral letter*.ts.
Missing referral letter*.ts.
Wrong referral letter*.ts.
Illegible referral letter*.ts.
Misfiled documentation.ts.
No access to documentation.ts.
Patient incorrectly identified.ts.
Delay in obtaining clinical assistance.ts.
Difficulty in obtaining clinical assistance.ts.
Delay in recogni?ing complication* of treatment.ts.
Failure in recogni?ing complication* of treatment.ts.
Delay in monitoring.ts.
Failure to monitor.ts.
Failure to follow up.ts.
Infection Control.ts.
Failure of sterili?ation of equipment.ts.
Contamination of equipment.ts.
Health care acquired infection*.ts.
Healthcare acquired infection*.ts.
Health care associated infection*.ts.
Healthcare associated infection*.ts.
Wound infection*.ts.

Surgical site infection*.ts.
Unsafe environment*.ts.
Inappropriate environment*.ts.
Unsafe equipment.ts.
Inappropriate equipment.ts.
Availability of equipment.ts.
Availability of bed*.ts.
Availability of IT.ts.
Staff shortage*.ts.
Unavailability of staff.ts.
Lack of skilled staff.ts.
Unskilled staff.ts.
Lack of suitably trained staff.ts.
Failure of device*.ts.
Failure of equipment.ts.
Unavailability of device*.ts.
Extended stay.ts.
Extended episode* of care.ts.
Failure to discontinue treatment*.ts.
Infusion injur*.ts.
Missing needle*.ts.
Missing swab*.ts.
Missing instrument*.ts.
Retained needle*.ts.
Retained swab*.ts.
Retained instrument*.ts.
Theatre list details incorrect.ts.
Inappropriate treatment*.ts.
Wrong treatment*.ts.

Unplanned return to theatre.ts.
Maternal death*.ts.
Anaesthetic complication*.ts.
Intensive Therapy Unit Admission*.ts.
Intensive Treatment Unit Admission*.ts.
Intensive Care Unit Admission*.ts.
Venous thromboembolism*.ts.
Pulmonary embolism*.ts.
Readmission of mother.ts.
Stillbirth*.ts.
Neonatal death*.ts.
Birth trauma*.ts.
Term baby admitted to neonatal unit.ts.
Undiagnosed f?etal abnormalit*.ts.
Pressure ulcer*.ts.
Padded room*.ts.
Ligature point*.ts.
Self-neglect.ts.
Self neglect.ts.
Splint*.ts.
Head bang*.ts.
Head-bang*.ts.
ALL PATIENT SAFETY TERMS COMBINED WITH OR (PATIENT SAFETY FACET.ts.)
Research.ts.
Academic work.ts.
Academic understanding.ts.
Theor*.ts.
Randomised controlled trial*.ts.
Controlled clinical trial*.ts.

Random allocation.ts.
Double blind method.ts.
Single blind method.ts.
Single blind stud*.ts.
Double blind stud*.ts.
Triple blind stud*.ts.
Multicentre stud*.ts.
Random sample*.ts.
Evidence base*.ts.
Evidence scan*.ts.
Systematic review*.ts.
Scoping review*.ts.
Narrative review*.ts.
Literature review*.ts.
Meta narrative*.ts.
Meta synthesi*.ts.
Meta-analys*.ts.
Clinical trial*.ts.
Placebo*.ts.
Comparative stud*.ts.
Evaluation stud*.ts.
Evaluative stud*.ts.
Descriptive stud*.ts.
Community trial*.ts.
Follow up stud*.ts.
Prospective stud*.ts.
Longitudinal stud*.ts.
Qualitative.ts.
Quantitative.ts.

Focus group*.ts.
Semi-structured interview*.ts.
Quality improvement project*.ts.
Data collection.ts.
Data analysis.ts.
Survey*.ts.
Observation*.ts.
Ethnograph*.ts.
Intervention*.ts.
Investigation*.ts.
Experiment*.ts.
Case stud*.ts.
Delphi.ts.
Nominal group technique*.ts.
Nominal group stud*.ts.
Consensus stud*.ts.
ALL RESEARCH TERMS COMBINED WITH OR (RESEARCH FACET.ts.)
Hospital*.ts.
Acute care.ts.
Secondary care.ts.
Tertiary care.ts.
Unit*.ts.
Ward*.ts.
Low secure.ts.
Medium secure.ts.
High secure.ts.
Secure facilit*.ts.
Forensic*.ts.
Inpatient*.ts.

Triage.ts.
ALL INPATIENT SETTING TERMS COMBINED WITH OR (INPATIENT SETTING FACET.ts.)
FINAL SEARCH RESULT: ALL FACETS COMBINED WITH AND (MENTAL HEALTH AND PATIENT SAFETY AND RESEARCH AND INPATIENT SETTING.ts.)

Final search string:

((Patient safety or Adverse event* or Adverse drug event* or Sentinel event* or Incident* or Error* or Near miss* or Close call* or Never event* or Critical outcome* or Adverse outcome* or Unanticipated outcome* or Suicide* or Self-harm or Self harm or Behavio?r control or Restraint or Seclusion or Safety management or Failure to diagnose or Failure of diagnos?s or Under diagnosis or Over diagnosis or Misdiagnosis or Dual diagnos?s or Delay in diagnos?s or Wrong diagnos?s or Incorrect diagnos?s or Safety culture or Safety climate or Fall* or Slip* or Trip* or Falling or Slipping or Tripping or Accident prevention or Patient accident* or Patient in road traffic accident* or Collision with an object or Contact with an object or Contact with sharp* or Collision with sharp* or Exposure to hazardous substance* or Inappropriate patient handling or Inappropriate patient positioning or Elope or Wander or Runaway or Abscond* or Escorted leave or Unescorted leave or Aggressi* or Violence or Assault* or Abus* or Disruptive behavio?r or Racial attack* or Sexual attack* or Sexually inappropriate or Physical attack* or Verbal attack* or Missing patient* or Failure in access or Unexpected readmission* or Reattendance* or Unplanned admission* or Transfer to specialist care unit* or Delay in discharge or Failure to discharge or Inappropriate discharge or Planning failure or Self discharge or Self-discharge or Discharge against medical advice or Failure in referral process* or Failure to return from authorised leave or Transfer delay* or Transfer failure* or Inappropriate transfer* or Unsafe transfer* or Unsafe clinical environment* or Inappropriate clinical environment* or Inappropriate admission of a minor to an adult setting or Inappropriate transfer of a minor to an adult setting or Poor clinical assessment* or Lack of clinical assessment* or Lack of risk assessment* or Wrong scan* or Wrong x-ray* or Wrong specimen* or Inadequate scan* or Inadequate x-ray* or Inadequate specimen* or Incomplete scan* or Incomplete x-ray* or Incomplete specimen* or Mislabelled scan* or Mislabelled x-ray* or Mislabelled specimen* or Unlabelled scan* or Unlabelled x-ray* or Unlabelled specimen* or Missing scan* or Missing x-ray* or Missing specimen* or Failure to interpret test result* or Delay to interpret test result* or Failure to act on test result* or Delay to act on test result* or Failure to receive test result* or Delay to receive test result* or Incorrect test result* or Incorrect report* or Missing test result* or Missing report* or Failure to undertake test* or Delay to undertake test* or Patient confidentiality or

For peer review only

Communication failure* or Failed communication* or Failure in communication* or Failure to receive informed consent or Inadequate handover of care or Inadequate handover or Documentation delay* or Mislabelled documentation or Missing documentation or Inadequate documentation or Wrong documentation or Illegible documentation or Mislabelled healthcare record* or Inadequate healthcare record* or Missing healthcare record* or Wrong healthcare record* or Illegible healthcare record* or Mislabelled referral letter* or Inadequate referral letter* or Missing referral letter* or Wrong referral letter* or Illegible referral letter* or Misfiled documentation or No access to documentation or Patient incorrectly identified or Delay in obtaining clinical assistance or Difficulty in obtaining clinical assistance or Delay in recogni?ing complication* of treatment or Failure in recogni?ing complication* of treatment or Delay in monitoring or Failure to monitor or Failure to follow up or Infection Control or Failure of sterili?ation of equipment or Contamination of equipment or Healthcare associated infection or Healthcare acquired infection or Health care associated infection or Health care acquired infection or Health care acquired infection* or Healthcare acquired infection* or Health care associated infection* or Healthcare associated infection* or Wound infection* or Surgical site infection* or Unsafe environment* or Inappropriate environment* or Unsafe equipment or Inappropriate equipment or Availability of equipment or Availability of bed* or Availability of IT or Staff shortage* or Unavailability of staff or Lack of skilled staff or Unskilled staff or Lack of suitably trained staff or Failure of device* or Failure of equipment or Unavailability of device* or Extended stay or Extended episode of care or Extended episode* of care or Failure to discontinue treatment* or Infusion injur* or Missing needle* or Missing swab* or Missing instrument* or Retained needle* or Retained swab* or Retained instrument* or Theatre list details incorrect or Inappropriate treatment* or Wrong treatment* or Unplanned return to theatre or Maternal death* or Anaesthetic complication* or Intensive Therapy Unit Admission* or Intensive Treatment Unit Admission* or Intensive Care Unit Admission* or Venous thromboembolism or Venous thromboembolism* or Pulmonary embolism* or Readmission of mother or Stillbirth* or Neonatal death* or Birth trauma* or Term baby admitted to neonatal unit or Undiagnosed f?etal abnormalit* or Pressure ulcer* or Padded room* or Ligature point* or Self-neglect or Self neglect or Splint* or Head bang* or Head-bang* or Patient safety) and (Research or Academic work or Academic understanding or Theor* or Randomised controlled trial* or Controlled clinical trial* or Random allocation or Double blind method or Single blind method or Single blind stud* or Double blind stud* or Triple blind stud* or Multicentre stud* or Random sample* or Evidence base* or Evidence scan* or Systematic review* or Scoping review* or Narrative review* or Literature review* or Meta narrative* or Meta synthesi* or Meta-analys* or Clinical trial* or Placebo* or Research design or Comparative stud* or Evaluation stud* or Evaluative stud* or Descriptive stud* or Community trial* or Follow up stud* or Prospective stud* or Longitudinal stud* or Qualitative or Quantitative or Focus

For peer review only

group* or Semi-structured interview* or Quality improvement project* or Data collection or Data analysis or Survey* or Observation* or Ethnograph* or Intervention* or Investigation* or Experiment* or Case stud* or Delphi or Nominal group technique* or Nominal group stud* or Consensus stud* or (Research or Descriptive research methods or Psychological research or qualitative research or mixed methods research or Experimental research or Applied research or research methodology or Medical research or Research implementation or Research design or Scientific research or Research findings or Sociological research or Research reports or Social research or Empirical research methods or Research methods or Social service research or Social welfare research or quantitative research or Research projects or Health services research)) and (Mental health or Mental well-being or Mental well being or Psychological well being or Psychological well-being or Mental disorder* or Mental illness* or Mental disease* or Anxiety disorder* or Delirium or Dementia or Dissociative disorder* or Factitious disorder* or Impulse control disorder* or Mood disorder* or Affective disorder* or Psychotic disorder* or Depressive disorder* or Neurotic disorder* or Personality disorder* or Conduct disorder* or Schizophreni* or Somatoform disorder* or Substance related disorder* or Clinical Psychology or Impulsive behavio?r or Adjustment disorder* or Eating disorder* or Sleep disorder* or Neuros?s or Psychos?s or Delusion* or Paranoia or Hallucination* or Addiction* or Dependence or Misuse or New psychoactive substance* or Legal high* or Depression or Panic disorder* or Phobia* or Health anxiet* or Bipolar disorder* or Alcohol abuse or Alcoholism or Obsessive compulsive disorder* or Obsessive thought* or Intrusive thought* or Post traumatic stress disorder* or Post-traumatic stress disorder* or Cognitive Behavio?ral Therap* or Psychotherap* or Person centred therap* or Person-centred therap* or Counselling or Antidepressant medication* or Antipsychotic medication* or Antianxiety medication* or Psychotropic medication* or Mindfulness based cognitive therap* or Mindfulness-based cognitive therap* or Mindfulness based relapse prevention or Mindfulness-based relapse prevention or Mindfulness based stress reduction or Mindfulness-based stress reduction or Electroconvulsive therap* or Verbal de-escalation or Therapeutic or Functional Analys?s or Dialectical Behavio?r Therap* or Dysexecutive syndrome or Mental health or mental disorders or Psychiatr*) and (Hospital* or Acute care or Secondary care or Tertiary care or Low secure or Medium secure or High secure or Secure facilit* or Forensic* or Inpatient* or Triage or (Acute hospitals or Mental health hospitals or hospitals) or In patients or Unit* or Ward*)).ts.

For peer review only - http://bmjopen.bmj.com/site/about/guidelines.xhtml

Online supplement 3

Research categories, subcategories by subsequent articles and reference

Category	Subcategory	Articles *(Reference number)*
Interpersonal Violence	Aggression	49 (*20-21, 23-25, 30, 35-38, 40-41, 44-52, 58-59, 66, 68-71, 74-79, 81-82, 86-89, 94, 97, 100-101, 104, 107-108, 112-113*)
	Violence	33 (*22, 26-29, 31-33, 43, 53-57, 64, 72-73, 80, 83-85, 90-93, 95-96, 98, 102-103,109-111*)
	Challenging Behaviour	10 (*114-123*)
	Violence and Aggression	12 (*34, 39, 42, 60-63 ,65, 67, 99, 105-106*)
	Critical Incidents	4 (*128-131*)
	Conflict	4 (*124-127*)
	Sexual Assault	2 (*133-134*)
	Agitation	1 (*135*)
	Abuse	1 (*132*)
	**Total**	**116 *(32%)***
Coercive Interventions	Restraint	23 (*138, 148-151, 155-156, 158-159, 166-167, 172,174-176, 181-183, 193, 195-196, 199, 201*)
	Seclusion	20 (*136-137, 147, 152-153, 160-162, 169, 173, 177-180, 184-186, 191-192, 206*)
	Attitudes to Coercion	25 (*142-145, 167, 211-30*)
	Seclusion & Restraint	16 (*146, 154, 157, 163-165, 170-171, 187-190, 194, 197-198, 200*)
	Containment	4 (*202-205*)
	Process of Coercion	6 (*139-141, 231-233*)
	Alternative Interventions	1(*209*)
	Shielding	1 (*207*)
	Conflict	1 (*208*)
	Personal Factors	1 (*210*)
	**Total**	**98 *(27%)***
Safety Culture	Process	18 (*234-251*)
	Culture	19 (*252-270*)
	Policy	8 (*271-278*)
	Building Therapeutic	2 (*281-282*)

For peer review only

	Relationships	
	Patient/Family Engagement	2 (279-280)
	**Total**	**49 *(13%)***
Harm to Self	Self-harm	18 (283-300)
	Suicidal Behaviour	17 (301-317)
	Self-neglect	1 (318)
	**Total**	**36 *(10%)***
Safety of the Physical Environment	Security	7 (319-325)
	Environment Design	5 (326-330)
	Transitions of Care	3 (331-333)
	Patient Distribution	3 (334-336)
	Staffing	2 (337-338)
	Ligatures	1 (339)
	**Total**	**21 *(6%)***
Medication Safety	Adverse Events	5 (340-344)
	Medication Administration	5 (348-352)
	Medication Management	3 (345-347)
	Medication Dispensing	1 (353)
	Adherence	1 (356)
	Substance Use	2 (354, 355)
	**Total**	**17 *(5%)***
Unauthorised Leave	Absconding	9 (357-362, 365-367)
	Wandering	2 (363-364)
	**Total**	**11 *(3%)***
Clinical Decision Making	Incident Management	5 (370-374)
	Risk Assessment	2 (375-376)
	Diagnosis	2 (368-369)
	**Total**	**9 *(2%)***
Falls	Falls	4 (377-379, 382)
	Injuries	2 (380-381)
	**Total**	**6 *(1%)***
Infection Prevention & Control	Infection Prevention & Control	1 (383)

	**Total**	**1 *(<1%)***

For peer review only

Online supplement 4

Data extraction tables by research category

Interpersonal Violence

Author, year, country	Study design	Setting	Participants	Aims/ objectives related to patient safety	Outcomes related to patient safety	Study quality
Almvik, Rasmussen & Woods (2006) Norway	Observation/ Questionnaire/ Quantitative	Geriatric psychiatric wards and special care units for patients with dementia (SCU) within nursing homes (n=2)	Residents (n=82); 43.9% male; mean age 81.96 (range 56-104).	To explore violent incidents in an elderly population	39% of patients were reported to be violent. The mean score on the SOAS-R severity scale was 9.89 (median 10, SD 5.00, 95% CI 9.21–10.56). The situations where the client was denied something were the most provocative ones (37.9% of occurrences). Verbal aggression was the most frequent type of attack (58%). The staff were the most frequent targets of the violent incidents (78.8%). The incident had no consequences for the victim in 47.6% of cases. Talking to patients was the most common measure taken to stop aggression (63.7%). Most incidents occurred in the morning (42.9%).	Fair
Amoo & Fatoye (2010) Nigeria	Questionnaire/ Quantitative	Inpatient psychiatric wards of a neuropsychiatric hospital	Psychiatric inpatients (n=305); 69.8% male. Age range of the aggressive patients 13-66.	To investigate aggressive behaviour and related variables in psychiatric inpatients	43 patients manifested aggressive behaviour (13.8% of sample). Most aggressive behaviour occurred during evening and night periods (69.8% of incidents) and was evenly distributed throughout the week. Physical aggression was the most common type of behaviour (37.2%) and female nurses were the most common targets. Hallucinations were the most common probable cause (34.9%).	Fair

For peer review only

Arguvanli, et al. (2015) Turkey	Pre-post questionnaire/ Quantitative	Psychiatric inpatient clinics within hospitals (n=2)	Nurses (n=27). 78% female. Mean age 33.2±5.4 (range 22-47)	To evaluate the Aggression Management Training Program (AMTP) in psychiatric inpatient clinics	96.3% of participants reported that they did not receive aggression management training. Participant knowledge and functional reactions to aggression increased significantly from pre-post intervention.	Fair
Bahareethan & Shah (2000) UK	Questionnaire/ Quantitative	Continuing care psychogeriatric wards within a London hospital (n=2)	Patients (n=39); 26% male. Median age 83 (range 68-99).	To investigate aggressive behaviour and staff perception and attitudes in psychogeriatric wards	There was a moderate correlation between aggressive behaviour (RAGE scale) and staff perception of the patient (MAS scale) (r=+0.67, p<0.00001). The highest correlation was between the RAGE score and the MAS subscale of 'alienation of the patient (r=+0.85, p<0.00001).	Fair
Bennett, Ramakrishna & Maganty (2011) UK	Questionnaire/ Quantitative	A psychiatric intensive care unit (PICU) within a medium-secure unit	Nursing and multidisciplinary staff of the PICU (n=43)	To investigate staff views of different management techniques for disturbed behaviour in PICUs	Individual counselling was the preferred choice of management (n=40, mode rank given=1; mean rank given=1.25; median rank given=1; SD=0.77)	Fair
Ben-Zeev, et al. (2017) USA	Questionnaires / Quantitative	Secure hospital	Inpatients (n=27). 86% male. Mean age 33+11.13	To examine multimodal technologies to identify correlates of violence among inpatients with serious mental illness.	Self-reported delusions were associated with violent ideation (odds ratio [OR]=3.08), damaging property (OR=8.24), and physical aggression (OR=12.39). Alcohol and cigarette cravings were associated with violent ideation (OR=5.20 andOR=6.08, respectively), damaging property (OR=3.71 and OR=4.26, respectively), threatening others (OR=3.62 and OR=3.04, respectively), and physical aggression (OR=6.26, and OR=8.02, respectively). Drug cravings were associated with violent ideation (OR=2.76) and damaging property	Fair

For peer review only

					(OR=5.09). Decreased variability in physical activity and noisyward conditionswere associatedwith violent ideation (OR=.71 and OR=2.82, respectively).	
Berg, et al. (2013) Europe	Interviews/ Qualitative	Forensic psychiatric units (n=4) in Belgium (n=1) Finland (n=1), Netherlands (n=1) and the UK (n=1)	Staff in the units (n=58). Mean age 36 (range 20-75)	To investigate staff perceptions of aggressive behaviour in forensic psychiatric patients	Participants described aggressive behaviour as consisting of verbal, non-verbal and physical dimensions. Staff being authoritative with patients was described as provoking aggressive behaviour and more commonly leading to physical management techniques. Limit-setting situations were also identified as provoking aggressive behaviour towards staff.	Good
Berg, Kaltiala-Heino & Välimäki (2011) Europe	Interviews/ Qualitative	Forensic psychiatric units (n=4) in Belgium (n=1) Finland (n=1), Netherlands (n=1) and The UK (n=1)	Staff in the units (n=58). Mean age 36 (range 20-75)	To explore practices for managing adolescent aggressive behaviour in forensic psychiatric units	Participants favoured the use of verbal and less restrictive aggression management interventions. The use of coercive management interventions was described as a last option. Participants described using knowledge of the patient, the level of aggression, the situation development and available resources to decide on appropriate action. Countries differed on the practical responses to aggression but shared the same basic components.	Good

For peer review only

Bharwani, et al. (2012) USA	Observation and questionnaire/ Mixed methods	An Alzheimer unit within a continuing care retirement community	Residents of the unit (n=18). 54% female. Average age 82	To explore the benefits of a Behaviour-Based Ergonomic Therapy (BBET) program designed as an intervention for challenging behaviour	Resident falls decreased by 32.5% during the first 6 months of BBET implementation; from an average of 6.67 falls per month (n=40 in the 6 months before implementation) to 4.5 falls per month (n=27 in the first 6 months of implementation). Staff and family members commented on a reduction in agitation, including emergency calls about resident behaviour.	Fair
Biancosino, et al. (2009) Italy	Questionnaire and chart review/ Quantitative	Public (n=113) and private (n=32) acute psychiatric inpatient facilities	Psychiatric inpatients in the facilities (n=1324). 51% male. 28.2% were 35-44.	To examine the nature and predictors of aggressive behaviour in psychiatric inpatients	6.3% of patients had been physically restrained. Violent patients were more likely to be restrained than non-violent patients (up to 51.4% of this group (χ2= 203.07; p<0.001). No association with violent behaviour was found when treatment setting variables were examined (restrictiveness, standardization of the process of care, treatment, and staff number/qualification).	Fair
Bilici et al. (2016) Turkey	Quantitative	Locked psychiatric clinic	Staff (n=137). Chief nurse (n=9), resident (n=34), chief resident (n=10), assistant clinic chief (n=3) and clinic chief (3).	To examine the rates of exposure to violence including quantity, types of violence and compare to occupation types.	The majority of staff had been exposed to a violent incident (92.7%) and viewed security measures to be insufficient (87.6%). Positive correlation with the number of years working in the locked unit and number of exposures to verbal aggression (p<0.05).	Fair

For peer review only

Björkdahl, Hansebo & Palmstierna (2013) Sweden	Non-randomised pre-post/ Quantitative	Psychiatric inpatient units (n=41). Emergency and admission wards (n = 2), general wards (n = 30), psychiatric intensive care units (n = 2), drug and alcohol dependence wards (n = 2) and forensic wards (n = 5).	Staff in the units (n=854 before training; n=260 after). 40% male before training; 42% male after. Inpatients (n=297 before training; n=156 after). 55% male before training; 51% male after. Age range <25->40.	To explore the influence of a violence prevention and management staff-training programme on the climate related to violence management in psychiatric inpatient wards.	Staff perceived the violence management climate as significantly more positive on the trained wards compared with the wards that had not yet been trained, Mann–Whitney P=0.045. Specifically, the areas viewed more positively were: ward rules (odds [OR] = 1.97, P=0.001), the emotional regulation of staff members in challenging situations (odds [OR] = 2.24, P=0.007), the staff's interest in possible causes for patient aggression (odds [OR] = 1.64, P=0.031) and the staff's readiness to intervene at an early stage of patient aggression (odds [OR] = 2.10, P=0.001). On the trained wards, patients viewed perceptions of the staff's interest in finding possible causes for patient aggression significantly more positively (odds [OR]= 1.98, P=0.022)	Fair
Boström, et al. (2012) Canada	Questionnaire/ Mixed methods	Resident care units representing two models of residential care, Residential Alzheimer's Care Centres RACCs (n = 2) and Secured Dementia Units SDUs (n = 2).	Staff in the units (n=91). 98% female.	To explore aggressive incidents experienced by frontline staff in two types of residential care units	The most frequent type of aggression was physical assault (50% of staff reported experiencing this). 79% of incidences were officially reported by staff. Aggressive incidents were associated with working in SDUs rather than RACCs in a regression model (F =4.667, df = 6, p < 0.001). Other factors associated with aggressive incidents were: staff educational level, experience,	Fair

For peer review only

					exhaustion, staffing levels and staff informal interactions.	
Bowers (2009) UK	Cross-sectional/ Quantitative	Acute psychiatric wards (n=136) in 67 hospitals within 26 National Health Service (NHS) Trusts	All staff and patients within the units	To explore the effect of the positive appreciation of patients by staff, the staff's management of their own emotional reactions to patient behaviour, and the provision of an effective structure on conflict in acute psychiatric wards.	Only 18% of the variance in containment rates was explained by conflict. The regression model found that effective structure and order on the ward (the order and organization subscale and the program clarity subscale of the WAS) was the factor most strongly predictive of lower conflict and containment rates (Coefficient=-0.048, p=0.048).	Good
Bowers, et al. (2009) UK	Questionnaire/Quantitativ e	Acute psychiatric wards (n=136) in 67 hospitals within 26 National Health Service (NHS) Trusts	All staff and patients within the units	To explore the relationship of patient violence to other patient characteristics, features of the service and physical environment and staff factors.	Higher levels of aggression were associated with high patient turnover, alcohol use by patients, ward doors being locked, and higher staffing numbers (especially qualified nurses).	Fair
Calabro, Mackey & Williams (2002) USA	Pre-post evaluation/Quantitative	An acute care psychiatric hospital	Staff (n=118). 67% female. Modal age group 40-49	To evaluate the effectiveness of a training program to prevent and manage patient violence within acute psychiatric wards	There were improvements in post-test measures for: knowledge (t[109]=7.29, p < 0.001); attitude (t[109]=-5.68, p < 0.001); self-efficacy (t[114]=-2.82, p < 0.01); and intention to use the training techniques	Fair

For peer review only

					(t[114]=-1.99, p < 0.05).	
Camuccio, et al. (2012) Italy	Focus groups/ Qualitative	Acute psychiatric inpatient units (n=7)	Nurses (n=33). 70% female. Mean age 40.	To explore the experiences of acute psychiatric inpatient unit nurses when managing aggressive and distressed patients	The predominant theme was of fear, and this consisted of: fear of harm to self or patient; team safety; the known/unknown patient; non-psychiatric patients; and patient physique. Co-ordination with other services was expressed as key to good quality care.	Fair
Carlson, et al. (2010) USA	Observation, chart review and semi-structured interviews/ Quantitative	A psychiatric inpatient unit	Patients (n=130; n=151 admissions). 80% male. Mean age 9.64 ± 2.09	To examine the safety and efficacy of liquid risperidone to reduce rages in children admitted to a psychiatric inpatient unit.	75% of the sample had multiple rages in hospital. No adverse events related to the medication were observed. In the 16 children who had more than 3 rage outbursts during hospitalisation, there was a significant drop in duration of the outbursts from the non-medicated state to the highest dose state (t(15)=3.43, p<0.004).	Fair

For peer review only

Caspi (2015) USA	Observation, chart review and semi-structured interviews/ Qualitative	Special dementia care units within an assisted living residence (n=2)	Residents (n=12). 92% female. Mean age 81.	To explore aggression between residents (ABBR) with dementia within assisted living residences	For the majority of ABBR incidents there were observable causes and triggers (77% in one unit; 57% in the other). The most common cause was problematic seating arrangement (n=17). There were 12 effective staff management strategies: being alert; being proactive; being informed about aggression history; redirecting residents; offering to take a walk; separating; positioning, repositioning, or changing seating arrangement; refocusing; distracting the person; staying calm; never arguing with a resident; seeking help from other staff members.	Good
Caspi, et al. (2001) Israel	Double-blind crossover trial/ Quantitative	A maximum security unit within a mental health centre	Schizophrenic inpatients (n=30). 100% male. Mean age 37.2 ±9.6 (range 20-65)	To examine the efficacy of augmenting antipsychotic treatment with pindolol to reduce aggression in schizophrenic inpatients.	Pindolol treatment significantly reduced the number of aggressive incidents towards objects and other persons (0.59 versus 1.46, F=6.09, P<0.02; 1.96 versus 3.23, F=4.17, P<0.05, respectively); and reduced the severity of the incidents (0.89 versus 3.58, F=19.42, P<0.0001; 2.89 versus 6.85, F=10.11, P<0.004, respectively).	Fair

For peer review only

Chan, et al. (2005) Hong Kong	Pre-post questionnaire/ Quantitative	A developmental disability unit within a mental health hospital	Patients (n=89). Experimental group: 60% female. Control group: 59% female. Modal age group 31-40	To evaluate the impact of multisensory therapy on the challenging behaviour of patients with developmental disabilities	Both groups of patients experienced reductions in levels of challenging behaviour over time. Between the mid- and post-1 assessments, the control group had greater reductions in challenging behaviour (F=4.26, p=0.04). Between the post-2 and post-3 assessments, the experimental group had greater reductions in challenging behaviour (F=4.22, p=0.04).	Fair
Chaplin, et al. (2008) UK	Questionnaire/ Mixed methods	Older adult (OA) wards (n=75) and adults of working age (WAA) wards (n=88) within 66 English and Welsh NHS trusts and independent sector providers	Staff and patients (n=3,332)	To explore physical aggression/assault on psychiatric wards	Nurses on WAA wards were more likely to report dealing with aggressive incidents (85%, x^2=11 p=0.0006); using rapid tranquilisation (68%, x^2=73 p<0.0001); receiving adequate training in aggression management/prevention (79%, x^2=6.5 p=0.011); and having a personal alarm (86%, x^2=35 p=0.0001) than nurses on OA wards. The frequency of restraint was the same between the two types of wards. Provision of ward based activity and therapy were significantly better for patients on OA than WAA wards.	Poor

For peer review only

Chen, Hwu & Wang (2009) Taiwan	Questionnaire/ Quantitative	A psychiatric hospital	Staff (n=222). 53% female. Modal age group 30-44.	To explore staff responses to workplace violence in a psychiatric hospital	Staff experiencing verbal abuse were most likely to take no action (27%). Staff experiencing sexual harassment were more likely to report the incident to senior staff than staff experiencing other types of violence (76%). The most common form of management following an incident of physical violence or racial harassment was medication injection (29% and 30%); and for incidents of verbal abuse, bullying/mobbing and sexual harassment it was issuing a verbal warning (30%, 29% and 29%). The most common reason for not reporting violent incidents was that it was considered unimportant.	Fair
Chen, et al. (2007) Taiwan	In-depth interviews/ Qualitative	A psychiatric hospital	Staff victims of assault (n=13). Age range 23-60.	To explore the effects of assault on staff caring for schizophrenic patients in a psychiatric hospital	All staff reported that they did not receive enough post-incident support. The most common time for violence to occur was during ward inspections when staff were alone. The most severe psychological harm to staff was post-traumatic stress disorder. Staff gave suggestions for preventing further attacks: pre-placement training, good practice (asking for consent, not being alone, taking precautions and respecting patients' rights), and addressing organisational culture.	Fair

For peer review only

Cole, Baldwin & Thomas (2003) UK	Questionnaire/ Quantitative	Acute inpatient wards (n=3), a rehabilitation ward (n=1), and an intensive care ward (n=1) within a general adult psychiatric unit	Staff (n=109)	To explore staff practices and emotions concerning patient sexual assault in a psychiatric unit	The actions that most participants felt should be taken following a sexual assault were recording in notes (98.1% agreed) and informing teams involved (98.1% agreed). The emotional response to patient sexual assault that most participants reported feeling 'a lot/extremely' was being calm (38.3%). the majority of participants worried about being blamed in the instance of a sexual assault.	Fair
Cutcliffe (1999) UK	Semi-structured interviews/ Qualitative	An inpatient unit within a psychiatric hospital	Qualified nurses (n=6)	To explore the experiences of nurses who experience violence perpetrated by psychiatric patients.	3 key themes emerged: personal construct of violence, feeling equipped, and feeling supported. A relationship was found between exposure to violence and nurse's ability to manage these therapeutically. This is influenced by formal systems in place within the unit.	Fair
Daffern (2007) UK	Questionnaire/Qualitative	A high secure Dangerous and Severe Personality Disorder (DSPD) service within a secure hospital	Nursing staff and patients (n=18)	To explore nursing staff ability to assess the function of psychiatric patients' aggressive behaviour	The nursing staff's assessment of the functions of aggression differed from the patients' most significantly for the following: to enhance status or social approval, to obtain tangibles, and to observe suffering.	Fair
Daffern, et al. (2009) UK	Pre-post questionnaires/Quantitative	A high secure Dangerous and Severe Personality Disorder (DSPD) service within a secure hospital	Patients (n=51). Mean age 34 (range 18-54)	To evaluate whether structured risk assessment followed by results and management recommendations can reduce the frequency of aggression in DSPD patients	There was no significant difference in the frequency of reported aggression across the phases of the intervention. Staff reported that they did not feel the structured risk assessment was superior to clinical judgement and did not use it regularly in the management of aggression.	Fair

For peer review only

Daffern, Mayer & Martin (2006) Australia	Longitudinal/ Quantitative	Acute (n=2) and continuing care (n=1) wards within a secure forensic psychiatric hospital	Staff and patients	To explore the relationship between staff gender ratio and incidents of aggression in acute psychiatric wards	Verbal aggression was the most common form of aggression (n = 196, 62% of incidents). Victims were mostly staff (70%). Staff gender did not significantly affect the frequency or severity of aggressive incidents, or the likelihood of seclusion being used.	Fair
Daffern, Ogloff & Howells (2003) Australia	Longitudinal/ Quantitative	Acute (n=3) and continuing care (n=2) wards within a secure forensic psychiatric hospital	Staff and patients	To explore aggression and seclusion in secure psychiatric settings and to examine the reporting of aggression using aggression-specific recording instruments	Verbal aggression was the most common form of aggression (n = 205, 62% of incidents). Victims were mostly staff (n=229). 21% of aggressive incidents resulted in seclusion. There was no significant relationship between seclusion and severity of incidents, victim type (staff or patient), time of day or number of incidents in the preceding week.	Fair
Danivas, et al. (2016) India	Observational study/Quantitative	Acute psychiatric inpatient wards (n=2)	Inpatients (n=63)	To examine prevalence of aggression and coercion in a acute psychiatric ward.	There were 229 violent incidents reported; 55.9% identified as severe on SOAS-RI.Parents provoked 35% of the violent incidents and were the targets of the agression in 56% of the cases. Some incidents were managed using coercive measures (38%).	Good

For peer review only

Davies, et al. (2019) UK	Quantitative	Medium secure psychaitric hospital	Patients; Intervention group (n=22) and control group (n=17)	To evaluate the effectiveness of PBS in reducing frequency, management difficulty and severity of challenging behaviour	PBS significantly reduced challenging behaviour frequency and management frequency in the intervention group compared to the control group. Reductions were still evident six months after.	Fair
de Looff, et al. (2018) The Netherlands	Cross-sectional/Quantitative	Forensic psychiatric hospitals (n=4)	Forensic nursing staff (n=114). 59% female. Mean age 35 (SD=9.7)	To explore the relationship between type and severity of aggressive behaviour experienced by nursing staff and burnout symptoms	A positive association was found between physical aggression as experienced by staff and burnout symptoms (p=0.003)	Good
De Niet, Hutschemaekers & Lendemeijer (2005) Netherlands	Quasi-experimental time-series/ Mixed methods	A closed ward of a psychiatric institution	Nursing staff (n=23)	To explore reasons for the decline in the amount of reported aggression incidents when using the Staff Observation Aggression Scale (SOAS)	The number of reported aggression incidents decreased over the course of the intervention (t=2.598, df=55, P=0.012). Nursing staff did not perceive a reduction in the amount of aggression incidents, and stated that the SOAS did not add anything to the aggression management process, hence the reduction in reported incidents using this form.	Fair

For peer review only

Delaney, et al. (2001) Australia	Questionnaire, focus groups and chart review/ Mixed methods	Acute inpatient facilities (n=4)	Survey: nursing staff (n=59). 56% male. Modal age group 41-50. Focus groups: nursing staff	To explore the management of aggressive patients in psychiatric inpatient settings	88% of participants had been assaulted, with the majority (53%) stating that they had not attended aggression management training in the past year. Participants felt that history/background information of the patient, ongoing assessment, individualised care, peer support, policies/manual accessibility and stress management were key to effective aggression management.	Fair
De Young, Just & Harrison (2002) USA	Questionnaire/ Quantitative	A behaviour management unit within a long-term care facility	Patients (n=32). 53% male. Mean age 73 (range 38-95).	To evaluate a program designed to reduce the frequency of aggressive, agitated or disruptive (AAD) behaviours in a behaviour management unit	The most common AAD behaviours were resisting care and becoming upset/losing temper. There was a significant reduction in AAD incidents over the 6 month intervention period (t (df 18) =4.47, p=0.01). Staff reported using timing strategies to manage AAD behaviours most frequently.	Fair

For peer review only

Dickens, Piccirillo & Alderman (2013) UK	Questionnaire/ Quantitative	Low and medium secure wards of adult mental health care pathways of men's and women's services of a healthcare trust	Staff (n=72). Patients (n=98). 57% male. Mean age 34 (range 18-65)	To explore staff and patient attitudes concerning the management of violence and aggression in a secure, forensic mental health service	Patients and staff agreed on most internal causes of aggression, but differed on 2 points: patients agreed that it is difficult to prevent aggression and that being left alone can calm aggressive patients, whereas staff disagreed. Staff were more likely than patients to agree that external factors were causative of aggression. Patients and staff agreed on most situational/interactional causes of aggression, but patients felt that aggression could happen when staff do not listen, whereas staff disagreed with this. Patients and staff agreed on most management techniques, but patients felt that seclusion/restraint was sometimes overused, whereas staff did not, and patients felt that medication should be used more, whereas staff did not.	Fair
Duxbury & Whittington (2005) UK	Questionnaire/ Mixed methods	Inpatient mental health wards (n=3)	Nurses (n=80; 76% female) and patients (n=82; 51% female)	To explore the views of staff and patients concerning the causes and management of aggression in inpatient mental health wards	Both patients and staff agreed that external factors (restrictive environments) contributed to aggression. Staff were more likely to agree that a patient's illness contributed to aggression. Patients were more likely to agree that interactions/situations contributed to aggression. Staff were more likely to endorse management techniques such as medication and seclusion, although both	Fair

					groups agreed that restraint was an inevitable part of keeping the wards safe.	
Evans & Petter (2012) UK	Questionnaires/ Mixed methods	A PICU	Staff (n=15). 60% male. Age range 30-50.	To explore staff perceptions of violence and how safe they feel on a PICU	Overall staff reported feeling safe and supported at work. Themes arising from the interviews highlighted personal and systemic factors that relate to feeling safe and supported. The most commonly mentioned personal factors were: aggression as illness related, and nurses remaining emotionally unaffected. The most commonly mentioned systemic factors were: fear of letting the team down, lack of senior management support (fear of blame), reliance on external staff as increasing risk, and first admissions being higher risk.	Fair
Foley, et al. (2003) USA	Structured interview/ Mixed methods	Special care dementia units (n=53) within nursing facilities (n=49)	Staff (n=32)	To identify what differentiates successful from unsuccessful behaviour management of dementia patients in SCUs	Factors were found to be associated with successful management of behaviour problems: behaviour prevalence/severity, management techniques, use of psychotropic medication/restraint, comorbidity, and family interaction.	Fair

For peer review only

Gallop, et al. (1999) Canada	Semi-structured interviews/Qualitative	Acute care psychiatric units within hospitals (n=3)	Patients (n=20). 100% female.	To explore safety concerns amongst abused women in inpatient psychiatric environments	The most prevalent concerns raised were: mixed-gender units (preferring separate areas), night-time routines (restricting medications and staff contact at night), and primary nursing (as important for feeling safe). Participants expressed a desire to be involved in decision making.	Fair
Giles, et al. (2005). USA	Descriptive/Quantitative	Locked skilled nursing facility	Psychiatric inpatients (n=40); 27% female; mean age 46 (range 21-71)	To examine the effectiveness of a programme to reduce aggression in inpatients	The programme resulted in a small reduction in aggressive incidents.	Fair
Hallett & Dickens (2015) UK	Cross-sectional/Mixed methods	Secure inpatient mental health unit	Clinical staff (n=72); 50% female	To explore perspectives of clinical staff about de-escalation including interventions used in de-escalation, what staff believe constitutes de-escalation and which interventions are effective	Half of the staff suggested medication was used as an intervention for de-escalation. 15% incorrectly stated that interventions such as seclusion, restraint and emergency intramuscular medication could be used as a de-escalation technique. Different aggression types were identified as needing different interventions and a personalised approach.	Fair
Higueras, et al. (2006) Spain	Experimental/ Quantitative	Inpatient psychiatric ward of a general hospital	Psychiatric inpatients; baseline (n=83); mean age 41; 40% female	To examine the effects of a humour-based activity on disruptive behaviours	Disruptive behaviour significantly reduced after the intervention compared to baseline. Specifically, attempted escape, self-harm and fighting significantly reduced.	Fair
Hvidhjelm, et al. (2016) Denmark	Quasi-experimental design with a pre- and post-test/ Quantitative	Psychiatric wards (n=15) assigned to intervention (n=7) and control (n=8) group	Patients in control wards (n=595). 51.1% male. Mean age 45.1 + 16.7.<br><br>Patients in intervention (n=519) wards. 53% male. Mean age 46.4 +	To evaluate the effect of routine use of the Brøset Violence Checklist (BVC) throughout the entire admission on the risk of patient aggression as assessed at the ward	The analysis was conducted at the ward level because each ward was allocated to the intervention and control groups. At baseline, the risk of aggression varied between wards, from one	Good

For peer review only

			18.8.	level	aggressive incident per patient per 1,000 shifts to 147 aggressive incidents per patient per 1,000 shifts. The regression discontinuity analysis found a 45% reduction in the risk of aggression (Odds Ratio (OR) = 0.55, 95% confidence interval: 0.21–1.43). The study did not find a significant reduction in the risk of aggression after implementing a systematic short-term risk assessment with the BVC.	
Hylén, et al. (2019) Sweden	Focus groups and interviews/ Qualititative	Psychiatric inpatient care wards (n=3): general inpatient care, forensic care and addictive care.	Nurses and assistant nurses (n=17) and their managers (n=3)	To describe the nursing staff and ward managers' experiences of safety and violence in (1) the everyday meeting with the patient and (2) meetings that are violent or present a risk of violence	The qualitative content analyses resulted in four themes: the relationship with the patient is the basis of care; the organizational culture affects the care given; knowledge and competence are important for safe care; and the importance of balancing influence and coercion in care. The staff had a varied ability to meet patients in a respectful way.	Fair
Ilkiw-Lavalle & Grenyer (2003) Australia	Mixed-methods design/ Mixed-methods	Psychiatric inpatient unit (n=4)	Staff (n=29); 48% female; mean age 33 and 29 inpatients (n=29); 35% female; mean age 31	To examine views of staff and patients involved in incidents of aggression to understand emotions experienced, perceptions of causes and recommendations for reducing aggression	Staff and patient views differed on the cause of the aggression: staff felt it was the patient's mental illness and patient's felt illness, interpersonal and environmental factors were equally to blame for the aggression. Overall participants were satisfied with the management of	Fair

For peer review only

					aggressive incidents.	
Ireland, Halpin & Sullivan (2014) UK	Semi-structured interviews/ Qualitative	Forensic psychiatric inpatient hospital	Male inpatients (n=16); mean age 31	To examine motivations for forensic patients' engagement in critical incidents	The main themes included engaging in critical incidents to seek deliberate isolation, to gain control, to get needs met, a need to communicate and because they were influenced by peers.	Fair
Ireland, et al (2019) UK	Exploratory study/quantitative	High secure psychiatric hospital	Study 1 - patients (n=44); study 2 - patients (n=53) and staff (n=167); study 3 - (n=414)	To investigate the impact of making changes to factors relevant to engaging and experiencing intra-group aggression (bullying) in a secure hospital	Indirect changes to the environment lead to more positive effects, in relation to bullying, than direct changes.	Fair
Isaak, et al. (2016) Israel	Questionnaires pre-post evaluation/Quantitative	A maximum security unit at a mental health centre	All staff (pre-intervention n=112; post-intervention n=85). 53% female	To evaluate the effectiveness of an intervention to improve safety climate in a secure psychiatric setting	Six months after the intervention, significant improvements were found in perceptions of management's commitment to safety (p=0.02) and in communication about safety issues (p=0.056)	Fair
Jacob, et al. (2013) India	Observational/ Quantitative	Child and adolescent inpatient service	Children inpatients (n=31); 16% female; Mean age 12.9	To identify characteristics of aggressive incidents and how they are managed	Almost a quarter of all inpatients (n=131) demonstrated aggression (23.7%). Aggression was most likely to occur at night and against family members	Fair

For peer review only

Janicki (2009) UK	Semi-structured interviews and case note review/ Mixed methods	Women's medium secure hospital	Participants (n=15); patients (n=6), nurses (n=3); and other professionals (n=6)	To explore staff and patient perspectives on the involvement of the criminal justice system as a response to an assault on the medium secure ward	Overall, participants felt the involvement of the CJS was essential to ensure high morale and that incidents were dealt with so that this deterred other patients from committing assault	Fair
Jeffs, et al. (2012) Canada	Explorative/ Qualitative	Inpatient mental health care agencies (n=3)	Staff: Site A (n=24), site B (n=19), Site C (n=24); patients: site A (n=9), site B (n=9), site C (n=10)	To gain insight into how service providers and service users experience and define near misses.	Overall near misses were identified as safety threats and issues associated with patients' mental illness and also situations that avoid harm to others and prevent an incident	Good
Kelly, et al. (2017) USA	Cross-sectional/Quantitative	Psychiatric hospital	Staff (n=323)	To examine staff wellbeing and safety procedures in relation to aggression	The majority of staff had experienced verbal aggression (98%) and over two thirds of staff had experienced physical assault in the hospital (69.5%). Consequences of the assaults included staff not feeling staff (44.6%) and wanting to be more protected at work (90.1%). Staff wellbeing and safety concerns were adversely affected by conflicts eith other staff members. Staff-patient relationships , stress reactivity and violence are linked.	Fair
Kelly, et al. (2017) USA	Cross-sectional/Quantitative	Psychiatric hospital	Staff, female (n=348).	To examine social support in psychiatric inpatient staff following an assault	Over-two thirds experienced physical assault. 54% of staff reported feeling very unsafe or unsafe at work. 28% of staff who had experienced an assault did not receive post-assault support.	Good

For peer review only

Killick & Allen (2005) UK	Experimental/ Quantitative	Psychiatric inpatient unit	Experimental staff group (n=27); 66.7% female; 40.9 mean months' work experience [89.2]	To assess the effects of three training courses on managing aggression, staff knowledge and satisfaction before, during and after training and at one year follow-up	Staff knowledge and confidence in managing aggression increased following training but was not maintained at 1 year follow-up	Poor
Koukia, et al. (2013) Greece	Cohort design/Qualitative	Psychiatric hospital (n=3)	Nurses (n=26); 62% female; mean age 36 years [7.7] (range 23-54)	To identify nurses' interventions, views, and attitudes concerning critical incidents.	Nurses reported six interventions included counselling, performing security practices, monitoring thought disturbances, contacting the psychiatrist on-call, contacting the chief nurse on-call and administrating medication.	Fair
Lamanna, et al. (2016). Canada.	Interviews/ Qualititative	Psychiatric department of a general hospital.	"Inpatients (n=14). 64.3% female. Mean age 49.1+18.9 (range 18-77)	Lamanna, et al. (2016). Canada.	Interviews/ Qualititative	Psychiatric department of a general hospital.
Lantta, et al. (2015) Finland	Intervention design, literature review, case-note review and focus groups/ Mixed methods	Mental health inpatient units (n=3)	Nurses (n=22)	To explain the intervention of implementing a structured violence risk assessment procedure in mental health inpatient units using the Ottawa Model of Research Use (OMRU) as a guiding framework. To also consider nurses' perspectives of its clinical utility and implementation process	Some staff felt the model was useful but it was less preferred than nurse's own clinical judgement in some instances	Poor

For peer review only

Lantta, et al. (2016). Finland.	Focus groups/ Qualititative	Inpatient psychiatric wards (n=3): acute admissions ward, acute forensics ward and treatment and rehabilitation ward	Nurses (n=22). 73% female.	To explore nurses' experiences of violent events in psychiatric wards, give insight into ward climates and examine suggestions for violence prevention.	Nurses' experiences of violent events included a variety of warning signs and high-risk situations which helped them to predict forthcoming violence. Patient-instigated violent events were described as complicated situations involving both nurses and patients. When the wards were overloaded with work or emotions, or if nurses had become cynical from dealing with such events, well-being of nurses was impaired and nursing care was complicated. Suggestions for violence prevention were identified, and included, for example, more skilled interaction between nurses and patients and an increase in contact between nurses and patients on the ward.	Fair
Lanza, et al. (2016) USA	Experimental/ Quantitative	Locked psychiatric units (n=7)	Violence Prevention Community Meeting group (n=4) and control group (n=3).	To assess the violence prevention community meeting (VPCM) as an effective intervention to reduce workplace violence in acute care psychiatric units	Per week aggression rates reduced slightly in the VPCM hospitals (0.6% (95% CI: -5.6%, 6.5%; non-significant)), but reduced significantly more in the control hospitals (5.1% (95% CI: 0.4%, 9.6%; significant)).	Poor
Lanza, et al. (2009) USA	Pre-post experimental design/Quantitative	Acute psychiatric inpatient unit at a Veterans Affairs hospital	Male patients. Mean age 42.6. Staff (n=21)	To test the efficacy of the VPCM for reducing patient violence	There was a decrease of physical violence of 89% from pre-treatment to treatment and a decrease of 57% from pre- to post-treatment.	Fair

For peer review only

Lawn & Pols (2003) Australia	Mixed-methods	Locked psychiatric ward	Inpatients (n=24). Staff (n=26)	To explore experiences regarding smoking and violence in locked inpatient settings	There was conflict between staff and patients over staff controlling the supply of cigarettes. This was made worse by the physical structure of the smoking area.	Poor
Lehmann, McCormick & Kizer (1999) USA	Descriptive/ Quantitative	Veterans medical centres and free-standing clinics (including inpatient psychiatric units) (n=166)	N/A	To examine the scope and impact of violence in hospital settings	During October 1990 to 1991 there were 6,592 incidents of physical assault in inpatient psychiatric units. The incident rate per 100,000 patient days was 177.9, equating to the highest proportion across medical units. A quarter of staff recommended more training (24.1%)	Poor
Lipscomb, et al. (2012) USA	Questionnaire/ Mixed methods	Residential addiction treatment centres (ATCs) (n=13)	Staff (n=409); 59% female	To examine the relationship between violence prevention safety climate measures and self-reported violence toward staff.	Predictors of violence included management commitment to violence prevention as "never/hardly ever" (OR=4.30), client actively resisting program (OR=2.34) and working with clients with unknown history of violence (OR=1.91)	Fair
Lowe, Wellman & Taylor (2003) UK	Vignette and questionnaire/ Quantitative	Acute psychiatric inpatient unit	Nurses (n=70)	To examine nurses response to scenarios of conflict incidents	Autonomy and limit-setting were acknowledged as the most notable responses to incidents of conflict. Across nurses these were most likely to cause disagreement.	Fair
McCann, Baird & Muir-Cochrane (2014) Australia	Questionnaire design/ Quantitative	Locked old age psychiatric inpatient units (n=3)	Staff (n=85). Registered nurses (61.1%, n = 52), enrolled nurses (27.1%, n = 23) and medical and allied health staff (11.8%, n = 10); 66% female; Mean age 43 (range 24-62 years)	To examine the attitudes of clinical staff toward the causes and management of aggression	Causes of aggression were multifactorial. Staff felt patient factors did not contribute to aggression. They felt other patients and staff contributed to aggression and specific cultural groups were more likely to be aggressive. Participants differed in their view of prevention strategies for aggression. Medication	Fair

For peer review only

					was deemed the more viable option but staff were undecided on the appropriateness of seclusion.	
McLaughlin, et al. (2010) UK	Pre-post intervention study/ Quantitative	Acute psychiatric inpatient ward	Nurses (n=18); median age 33 (range 23-52)	To examine the potential effectiveness of an intervention for managing verbal aggression	Post intervention focus group analysis revealed there was an increase in staff diffusing the situation to manage verbal aggression	Poor
Meaden, Hacker & Spencer (2013) UK	Interview/ Quantitative	High dependency inpatient units	Nurses (n=25); 48% female	To assess the ability of an adapted early warning signs type of dynamic risk assessment to more accurately predict aggressive incidents	Receiver Operating Characteristic (ROC) analyses revealed that the assessment had moderate predictive validity regarding aggression (AUC=0.5) but effect size was small.	Fair
Meehan, McIntosh & Bergen (2006) Australia	Focus groups/ Qualitative	High Secure Forensic Unit (HSFU)	Patients (n=27); 23% female	To explore the patient perspective on aggressive behaviour	There were five main themes that explored the cause of aggression: the environment, empty days, staff interactions, medication issues and patient centred factors. Potential management to reduce aggression was identified as improved training, separation of acutely disturbed patients, early intervention, improving staff attitudes and implementation of effective justice procedures.	Fair

For peer review only

Murphy, & Siv (2007) USA	Pre-post questionnaire/ Quantitative	Child and adolescent residential treatment unit	Patients (n=20). Average age in treatment as usual (TAU) group 14.8 and in Mode Deactivation group 15.7	To replicate previous findings supporting the effectiveness of Mode Deactivation Therapy (MDT).	The results showed MDT to be more effective then TAU in reducing both physical aggression and therapeutic restraints. MDT showed a reduction of 66.8% in physical aggression compared to TAU (27.9%). MDT showed a reduction of 70.7% in therapeutic holds compared to TAU (24.7%).	Fair
Needham, et al. (2005) Switzerland	Randomised controlled trial/ Quantitative	Acute psychiatric inpatient wards (n=6)	Nurses in control group (n=28); 50% female; mean age 39.1. Nurses in treatment group (n = 30); 60% female.	To investigate the effects of a training course in the management of aggressive behaviour in psychiatric acute inpatient settings.	No statistically significant differences of the means were found between the intervention and control groups on the positive perception of aggression (p=0.912), the negative perception of aggression (p=0.315), or the tolerance scale (p= 0.614).	Fair
Needham, et al. (2004) Switzerland	Feasibility study/ Quantitative	Acute mental health care inpatient settings (n=2)	Nurses. Patients (n=576); 41% females, mean age 38, age range 15–88	To implement a risk-prediction procedure and a standardized aggression management intervention.	There was a significant reduction in the percentage of days with attacks against persons (trend-test P = 0.04) and percentage of days with usage of coercive measures against any patient (trend-test P =0.01).	Good
Nijman, et al. (2005) UK	Cross-sectional/ Quantitative	Acute wards (n=12), psychiatric intensive care units (n=2), long-term rehabilitation wards (n=2), wards for the elderly mentally ill (n=3), and forensic wards at a medium security level (n=5)	Nurses (n=154); 58% female; 70% were under 40, and 36% were younger than 30	To explore psychiatric nurses' experiences with aggression	33 of 148 respondents (22%) said that they had not been able to go to work due to workplace violence at least once during the year. They had stayed at home for a total of 172 days, with an average of 5.2 sick days per nurse (range 1–23 days). Severe physical violence was the strongest predictor of sick leave (r=0.50). Frequent sexual harassment and intimidation also increased the likelihood	Fair

For peer review only

					of sick leave (r=0.38).	
Niu, et al. (2019) Taiwan	Cross-sectional/Quantitative	Acute psychiatric hospital	Nurses (n=429)	To examine prevalence of violence in acute psychiatric settings in staff.	The majority of staff had experienced workplace violence (88.3%); just over half (55.7%) had experienced physical violence.	Good
Nolan, et al. (2009) USA	Questionnaire/ Quantitative	Specialised research unit	Patients	To describe the reasons for aggressive behaviour reported by patients and staff and the relationships between those reasons and the subsequent interventions delivered in response to aggression.	Patients reported more often than staff that aggression was caused by external factors. Staff cited internal factors more often than patients. Responses to aggression were not related to the cause of the behaviour.	Fair
Olsson, et al. (2015) Sweden	Semi-structured interviews/ Qualitative	A maximum security forensic psychiatric hospital	Patients (n=12); 83% male; mean age 37.	To examine the experiences of forensic inpatients that have decreased their risk of becoming violent in forensic care.	3 themes emerged: 1) Staff's attitudes and actions. Patients' perception of staff´s ability to manage conflicts. 2) Patients' own insight and actions: Being insightful and managing the situation Dealing with aggression Attending to signs of warnings. 3) Interactions in the health care environment: Experiences of the physical environment- participants found overcrowded wards to be stressful and anxiety-provoking.	Fair

For peer review only

Olsson & Schon (2016) Sweden	Interviews/Qualitative	A maximum security forensic psychiatric hospital	Staff (n=13). 54% male. Mean age 30	To explore forensic psychiatric staffs' methods of violence prevention and compare these to recovery-oriented care	Three themes arose: internal knowledge; peer security; and control-oriented strategies	Fair
Park & Lee (2012) South Korea	Non-random experiment/ Quantitative	A psychiatric hospital	Inpatients in experimental group (n=22) and control group (n=22). 100% male (age range 30-60)	To measure the effect of behaviour modification using Short-Term Token Economy (STTE) on aggressive behaviour.	In the experimental group, aggressive behaviour decreased by 20.8% compared with the comparison group after STTE. The number of verbal attacks decreased by 27.6%. Property damage or physical attacks decreased by 14.3%.	Fair
Paschali, Lange-Asschhenfeldt and Kamp (2017) Germany	Cross-sectional/Quantitative	Secure psychiatric hospital	Inpatients (n=66).	To identify risk factors that lead to aggression using the SOAS-R scale	Over half of patients who had been involved in an aggressive incident had dementia (57%). 80% had a precipitating event. Majority of incidents were directed towards nurses.	Fair

For peer review only

Pekurinen, et al (2017) Finland	Cross-sectional/Quantitative	Psychiatric unit (n=84)	Staff (n=1033)	To examine the association between workplace factors and the likelihood of aggression	Workplace strain was significantly associated with aggression (OR 1.65, p = 0.02). Poor and average staff satisfaction (OR 1.83, p = 0.04, OR 1.93, p = 0.02, respectively) were also significantly related to increased levels of aggression.	Good
Phillips (2011) UK	Focus groups/ Qualitative	Inpatient wards	Mental health staff	To discuss issues raised during workshops aimed at encouraging and enabling staff to work in a gender-sensitive way to develop strategies for decreasing sexual assault incidence for female patients who may have histories of being abuse victims.	Staff taking part in the workshops expressed the importance of maintaining boundaries, developing adequate policies, and one-to-one observation. Issues regarding the truth of allegations and roles of the female patients in sexual assault incidents was mentioned as an issue. It was agreed that adequate staffing levels are essential for effective patient care.	Fair
Podubinski, et al. (2017) Australia	Cohort/Quantitative	Acute psychiatric units (n=2)	Inpatients (n=200). 66% female.	To examine different factors on aggression during psychiatriic hospitalisation	Just over a third of patients were deemed to be aggressive (35%). Interpersonal factors including hostile-domniance interpersonal style (OR 1.29 95% 1.17-1.43), psychopathy (OR 1.35 95% 1.16-1.58), aggressive script rehearsal (OR 1.21 95% 1.06-1.38), attitudes towards violence (OR 1.15 1.04-1.27), trait anger (OR 1.07 1.02-1.12) and disorganised (OR 1.09 95% 1.03-1.16) and excited symptoms	Good

For peer review only

					(PANSS) (OR 1.18 95% 1.04-1.34) all significantly predicted aggression.	
Price, et al. (2018) UK	Interviews/ Qualititative	In-patient wards (n=5) including male psychiatric intensive care units (n=3), female acute ward (n=1) and male acute ward (n=1)	Clinical staff (n=20). 50% female. Age range 18-60.	To obtain staff descriptions of de-escalation techniques currently used in mental health settings and explore factors perceived to influence their implementation and effectiveness.	Participants described 14 techniques used in response to escalated aggression applied on a continuum between support and control. Techniques along the support-control continuum could be classified in three groups: 'support' (e.g. problem-solving, distraction, reassurance) 'non-physical control' (e.g. reprimands, deterrents, instruction) and 'physical control' (e.g. physical restraint and seclusion). Charting the reasoning staff provided for technique selection against the described behavioural outcome enabled a preliminary understanding of staff, patient and environmental influences on de-escalation success or failure. Importantly, the more coercive 'non-physical control' techniques are currently conceptualised by staff as a feature of de-escalation techniques, yet, there was evidence of a link between these and increased aggression/use of restrictive practices.	Fair

For peer review only

Quirk, Lelliott & Seale (2005) UK	Longitudinal/ Qualitative	Inpatient wards (n=3) in general hospitals (n=2) and a psychiatric hospital (n=1)	Observations made by a research sociologist.	To explore how patients manage risks arising from their interaction with other patients on the ward, such as assault and sexual harassment.	Patients seemed to manage interaction risks by: avoiding risky situations or people; de-escalating situations; seeking safety interventions by staff or increased surveillance; and seeking protection from other patients.	Fair
Raveendranathan, Chandra, & Chaturvedi (2012) India	Semi-structured interviews/ Quantitative	Adult psychiatric wards	Family members of patients (n=75); 51% male; mean age 29	To assess inpatient violence from victims' perspectives, in settings where family members accompanied patients during inpatient stay and played a significant role in caregiving.	Family members were the targets of violence in 70% of the 100 incidents studied. 81% of these episodes were provoked. Provocation factors included the patient being prevented from leaving the ward and strict rules enforced by the staff. Family members suggested several preventative measures: more staff, sedation, and improved communication.	Fair
Reininghaus, et al. (2007) UK	Questionnaire/ Quantitative	A high secure psychiatric hospital	Nurses (n=636); 68% male. Mean age 39	To investigate different stress resistance resources (SRR) in the stress processes of staff at a psychiatric hospital when physical assault occurs.	Physical assault was found to be significantly related to psychological distress ($P = 0.004$, $B = 0.181$, 95% CI 0.060–0.303, $R2 = 0.014$). Self-esteem, self-confidence and coping were found to be effective SRRs for mediating levels of psychological distress following a physical assault.	Fair

For peer review only

Ryan, et al. (2004) US	Prospective/Quantitative	An inpatient psychiatric hospital	Patients (n=111); 56% male; mean age 13.9 (SD= 2.8)	This study examined the frequency and nature of violence directed at staff in a state inpatient psychiatric hospital.	The highest reported reason (68%) for assaults of staff by patients was related to verbal exchanges with staff: 'request, direction, or command potentially viewed by the patient as limit setting or as coercive'	Fair
Sato, et al. (2017) Japan	Cross-sectional / Quantitative	Emergency psychiatric (EP) wards (n=8), acute psychiatric (AP) wards (n=6), and standard (S) wards (n=16) across psychiatric hospitals (n=20)	Patients:<br>EP wards: (n=111). 64.9% male. Mean age 42.4+21.3<br>AP wards: (n=131). 58.8% male. Mean age 50.3+17.6<br>S wards: (n=201). 60.7% male. Mean age 51.4+13.7<br>Psychiatric nurses.	To examine the characteristics of aggressive incidents by ward type	Findings suggest that ward environment was an important factor influencing aggressive behaviour. In acute and emergency psychiatric wards, staff members were the most common target of aggression. In acute psychiatric wards, staff requiring patients to take medication was the most common provocation, and verbal aggression was the most commonly used means. In emergency psychiatric wards, victims felt threatened. In contrast, in standard wards, both the target and provocation of aggression were most commonly other patients, hands were used, victims reported experiencing physical pain, and seclusion was applied to stop their behaviour.	Good
Schwartz & Park (1999) US	Cross-sectional/ Quantitative	A psychiatric facility	Residents (n=517) 52% male; age range 25-61.	To investigate the prevalence of assaults on psychiatric residents and the violence management training they receive.	73% of residents reported being threatened, and 36% reported being physically assaulted. A third received no violence management training, and a third felt that their training was inadequate.	Fair

For peer review only

Selenius & Strand (2017) Sweden	Interviews/Qualitative	A high secure forensic hospital	Forensic psychiatric inpatients (n=13). 100% female	To explore how female psychiatric patients describe their aggression and self-harm behaviours	Three themes arose: from triggers to negative thoughts and emotions; strategies for handling negative thoughts and emotions; and satisfaction	Good
Sival, et al. (2000) Netherlands	Prospective cohort/ Quantitative	Wards of a psychogeriatric nursing home (n=2)	Residents (n=64); 72% female; mean age 80.2 (SD=7.8)	To investigate the effects of introducing a behaviour rating scale on the prevalence and management of aggressive behaviours in psychogeriatric patients.	The frequency of aggressive behaviour reported increased significantly ($p<.001$), while prescribing of psychotropic drugs decreased significantly ($p<.05$).	Fair
Sjöström, et al. (2001) Sweden	Quasi-experimental/ Quantitative	Inpatient psychiatric care	Nursing staff members (n=185) before training (n=144) after training.	To investigate whether aggressive behaviour and injury-related sick leave would be reduced by staff training and to explore predictors of violent behaviour.	No statistically significant reduction was found in the number of aggressive patients or in the number of staff members on sick leave. Directed verbal aggressiveness (OR = 1.92, P = 0.04) and violence towards things (OR = 1.82, P = 0.02). were found to be predictors of violence.	Fair
Skovdahl, Kihlgren & Kihlgren (2003) Sweden	Cross-sectional/ Quantitative	Units housing residents with dementia (n=2)	Unit 1: Caregivers (n=3); 100% female; mean age 38.5, age range 36-42; female resident (n=1); aged 92. Unit 2: Caregivers (n=6); 67% female; mean age 29.5, age range 22-43; female resident (n=1); aged 85.	To investigate, using video recordings, interactions between those with dementia and aggressive behaviour, and caregivers reporting experiencing problems dealing with this behaviour.	Interactions followed either a positive or negative spiral pattern. Caregivers who had reported problems dealing with behavioural symptoms focused on finishing the task (goals). Caregivers who were satisfied with their management of aggressiveness, focused on the processes of how goals could be achieved.	Fair

For peer review only - http://bmjopen.bmj.com/site/about/guidelines.xhtml

Speziale, et al. (2009) Canada	Pre-post intervention/ Quantitative	An inpatient facility for older adults with serious mental illness.	Staff who received the geriatric programme curriculum (n=99)	To examine the impact of training in Gentle Persuasive Approaches (GPA) on staff knowledge and competency regarding challenging patient behaviours (verbal and physical) and also on patient risk events and occupational health incidents.	Surveys found that GPA training significantly improved staff's response to challenging behaviours, and their learning of management strategies. The predicted use of body containment techniques decreased after training. Physical aggression rates declined by 50% 3 months after training (χ^2 (2, N = 564) = 27.51, p = .0001).	Fair
Spokes, et al. (2002) UK	Cross-sectional/ Qualitative	Psychiatric inpatient units (n=13); comprising adult acute admissions wards (n=10), psychiatric intensive care units (n=2) and a low-secure unit on five hospital sites	Qualified mental health nurses (n=68) and unqualified care assistants (n=40)	To investigate the views of mental health nurses about staff behaviours and other factors which may impact upon inpatient violence.	Three themes were identified by participants as being involved in the occurrence of violent incidents: clinical skills (experience, knowledge of techniques, job grade, etc.), personal characteristics (self-confidence, calmness, control, etc.) and interpersonal skills (rapport with patients, explaining things, etc.) Respondents also emphasized training needs, both in terms of new knowledge and of means of coping with actual physical violence.	Fair
Stevenson, et al. (2015) Canada	Cross-sectional/ Qualitative	Acute inpatient psychiatry settings	Registered nurses (n=12); 67% female; median age 37.5, range 27-57.	To explore nurses exposure to and experiences of patient violence, as well as the strategies they describe as influencing current practices of patient violence	For many, patient violence was considered "part of the job." Nurses often struggled with role conflict between one's duty to care and one's duty to self when providing care following a critical incident involving violence. Issues of power, control and stigma also influenced nurse participant perceptions and their responses to patient violence. The majority had difficulty identifying	Good

For peer review only

					any strategies that they perceived would have been successful to prevent the violence in their workplaces.	
Stone, et al. (2011) Australia	Cross-sectional/Mixed methods	A mental health inpatient setting	Nurses (n=107)	To determine the nature of interventions nurses used in response to aggression and whether interventions used varied with perceived causes of aggression. To identify the relationship between swearing and verbal aggression, and the impact of both on nurses.	The most frequently reported intervention for incidents of verbal aggression were talking to the patient (70% of incidents). The majority reported that exposure to swearing was highly distressing. Females appeared to be very distressed irrespective of the situation, whereas males reported being significantly more distressed by relatives of patients swearing at them.	Fair
Sukhodolsky, Cardona & Martin (2005) USA	Cross-sectional/ Quantitative	An inpatient psychiatric unit	Child inpatients (n=86); 67% male; mean age 10.8 (SD=2.4);	To evaluate the contribution of aggressive and non-compliant behaviours to restraint and seclusion use, length of stay, and psychotropic medication use in a psychiatric inpatient population.	Severity of aggressive behaviour was significantly associated with the use of seclusion and restraint, but not with the length of hospitalization or the number of psychiatric medications at discharge. By contrast, the levels of noncompliant behaviour were associated with the length of hospitalization and the number of psychiatric medications, but not with the use of seclusion and restraint.	Good
Sutton, et al. (2013) New Zealand	Prospective/Qualitative	Inpatient mental health units (n=4)	Clinical staff: (n= 40) 90% female. Service users (n = 20) 90% female	To investigate whether sensory-based approaches can develop the knowledge and practice of managing aggression in mental health settings.	Three main themes were found that described elements of sensory modulation that were perceived as helpful in the management of aggression: (i) facilitating a calm state; (ii) enhancing interpersonal connection; and (iii) supporting self-management.	Fair

For peer review only

Tema, Poggenpoel & Myburgh (2011) South Africa	Cross-sectional/ Qualitative	A forensic ward	Psychiatric nurses (n=9); 78% female; age range 26-58	To explore psychiatric nurses' experiences of patients' aggressive behaviour in a forensic ward and to develop recommendations for managers to equip nurses with the skills and knowledge necessary to effectively manage patient aggression.	Four themes emerged: challenging therapeutic relationships with patients; experiences of fear resulting from threats of aggression; experiences of disempowerment related to a lack of recognition; and experiences of emotional and physical distress related to interactions with patients. Recommendations for management were to provide nurses access to: information, support, resources, opportunity and growth.	Fair
Terkelsen & Larsen (2016) Norway	Observational/Interviews/ Qualitative	A locked psychiatric ward	Patients (n=12; 75% male; age range 17-53) and staff (n=22; 64% male; age range 20s-60s)	To explore what happens in a dangerous situation on a locked psychiatric ward and how staff and patients interpret them	Three themes arose: atmosphere and material surroundings; stereotypes; and triggers	Good
Tomagova, et al (2016) Slovakia	Cross-sectional/Quantitative	Psychiatric inpatient wards	Nurses (n=223)	To examine differences between staff educational training and patient aggression.	The majority of staff had experienced patient aggression (98.6%). Verbal aggression had the highest incidence (3.27 ± 1.04; p<0.077) followed by physical aggression without a weapon (2.61 ± 2.22). Staff had a negative attitude towards patient aggression (offensive, destructive and intrusive) dermined the formation of aggression management. Nurses expressed strongest aggreement in using restrictive tools and medication to manage patient aggression.	Good

For peer review only

Trenoweth, (2003) UK.	Cross-sectional/ Qualitative	A secure mental health environment	Mental health nurses (n=10)	To explore how mental health nurses make risk assessments in situations where violence is perceived to be likely.	It was found that nurses use information regarding their knowledge of the patient, observations of behaviour, assessing the situation as a whole, and then team working to intervene, when assessing risk in potentially violent situations.	Fair
Umut, et al. (2012) Turkey	Cross-sectional/ Quantitative	A research and training hospital	Inpatients (n=104); 31% female; age range 20-65	To investigate the relationship between violence and insight, clinical symptoms and treatment adherence in schizophrenia patients.	Treatment adherent patients scored significantly higher on the insight measure (Z=2.793; p<0.01). Non-treatment adherent patients scored significantly higher on the measure of aggression (Z=2.992; p<0.01).	Fair
van den Bogaard, et al. (2018) Netherlands	Quantitative	Specialised treatment centre with closed wards for mild to borderline intellectual functioning (n=3)	Staff (n=40)	To examine incidents of aggression, changes over time and and the circumstances of which the incidents happen.	There were 264 incidents of which 210 were deemed aggressive; mean incidents 8.9 per year. The most common incident type was verbal aggression (57.1%) followed by physical aggression (31.9%) and aggression against property (9.5%).	Fair
Van de Sande, et al. (2011) Netherlands	Cluster randomised controlled trial/ Quantitative	Acute psychiatric wards (n=2)	Experimental ward: baseline period (n=80); Mean age 38 (SD=13), 66% male. Intervention period (n=207); Mean age 38, 65% male. Control ward: baseline period (n=90); mean age 40 (SD=11) 60% male. Intervention period (n=251); Mean age 39, 55% male.	To investigate the effect of risk assessment on the prevalence of aggression and time in seclusion for acute psychiatric ward inpatients.	The experimental wards demonstrated a reduction in aggressive incidents (relative risk reduction, P<0.001), patients engaging in aggression (relative risk reduction, P<0.05), and time spent in seclusion (P<0.05) compared to control wards. No reductions were found in the number of seclusions or the number of patients exposed to seclusion.	Good

For peer review only

Van Wijk, Traut & Julie (2014) South Africa	Phenomenological, Cross-sectional/ Qualitative	Mental health facilities (n=2)	Inpatients (n=40); age range 21-55	To explore patients' perceptions of environmental and staff contributory factors for their aggression and violence; and to propose prevention and management strategies for this behaviour.	Two categories of contributory factors were found: environmental factors (such as living conditions and ward atmosphere), and the attitude and behaviour of staff.	Fair
Wright, et al. (2005) UK	Cross-sectional/ Quantitative	Acute inpatient settings	Nurses (n=771); 63% female; mean age 36.5 (SD=9.2)	To report and discuss the findings of a survey of nurse training and practice in the prevention and management of violence in acute psychiatric units	Most respondents reported a good balance of theory and practical skills training, although some aspects of theoretical training were not explored in enough depth. Reported confidence in the ability to use skills safely or effectively was fairly low. Training was generally considered to be safe and well run.	Good
Wright, et al. (2014) UK	Cross-sectional / Qualitative	High secure facilities (n=3)	Patients (n=8); 100% male. Staff (n=10); 70% male	To explore and compare staff and patient attitudes to the management of violence and aggression in a high security hospital	Seven themes emerged for both staff and patients: 'Environmental stimuli' where boredom was identified as a reason for violence and aggression occurring, 'Medication' in terms of aggression management, medication could be seen as both positive and negative, and 'Relationships' where therapeutic relationships were valued by both staff and patient and aggression was directly related to staff attitudes.	Poor

For peer review only

Wystanski (2000) Canada	Observational, Longitudinal/ Quantitative	A psychogeriatric inpatient ward in a psychiatric hospital.	Inpatients (n=29); 66% female; mean age 73.9 (SD=7.6);	To investigate the relationship between psychosocial stimulation and medication changes, and assaultive behaviour in a psychogeriatric ward.	Those with organic syndromes were more likely to become, and stay, aggressive. Psychosocial stimulation and changes in non-psychotropic medications influenced the duration of the aggressive behaviour.	Poor
Yang, et al. (2007) Taiwan	Pre-post experimental/ Quantitative	A nursing home specializing in care for patients with dementia.	Individuals living with dementia (n=20); 65% male; mean age 74.2 (SD=6.7), age range 65-86	To evaluate the effectiveness of acupressure in reducing agitation in those with dementia.	Significant differences on all outcome measures were found between control and experimental phases, indicating a positive treatment effect (Cohen–Mans-field Agitation Inventory, daily agitation records about physical attack, verbal and non-verbal attack and non-physical attack).	Fair
Yip, et al. (2013) UK	Quasi-experimental/ Quantitative	A high secure hospital	High risk patients (n=59); 100% male; age range 18-65	To evaluate a programme's effectiveness in reducing violent attitudes, anger, coping processes, social problem solving, disruptive behaviour and social functioning in forensic psychiatric patients.	Medium to large treatment effects were found for the treatment group in relation to: self-reported measures of violent attitudes, social problem-solving and coping. Staff also rated behaviour on the wards as being markedly improved post-treatment.	Good
Zuzelo, Curran & Zeserman (2012) USA	Cross-sectional/ Qualitative	An inpatient psychiatric unit	Professional nurses and behavioural health associations (N = 19); 47% male; age range >50 (5), 40-50 (3), 30-40 (6), <30 (5).	To explore nursing staff's responses to violent incidents perpetrated by patients against caregivers.	The themes that arose regarding management of violence were: sharing information about violence, therapeutic and non-therapeutic intervention, recognizing team influences, experiencing emotions following violence, and understanding the work environment.	Good

For peer review only

Zwijsen, et al. (2014) Netherlands	Cluster randomized controlled trial/Quantitative	Nursing care homes with special care units for people with dementia (n=17)	Residents with dementia (n=659); 70% female; mean age 84 (SD=7.3)	To evaluate the use of a care program in decreasing challenging behaviour and the prescription of psychoactive drugs without increasing the use of restraints.	Participants in the intervention condition compared with the control condition differed significantly in the presentation of challenging behaviours. Significant effects were found on the use of antipsychotics and antidepressants. No effect on use of restraints was observed.	Good

For peer review only

Coercive Interventions

Author, year, country	Study design	Setting	Participants	Aims/ objectives related to patient safety	Outcomes related to patient safety	Study quality
Abdel-Hussein & Mohamed (2018) Iraq	Questionnaires pre-post evaluation/Quantitative	A psychiatric teaching hospital	Nurses (n=25). 56% female. Modal age group 30-39	To evaluate the effectiveness of an educational programme aimed at increasing nurses' knowledge of restraint and seclusion in psychiatric wards	There was a significant increase in nurses' knowledge of restraint and seclusion across the course of the educational programme ($p=<0.01$)	Poor

For peer review only

Bak & Aggernæs (2012) Europe	Questionnaire/ Quantitative	Inpatient facilities in Denmark, Sweden, Norway, Finland, Iceland, Belgium, Netherlands, United Kingdom, Ireland, France and Italy.	Governmental health authorities (European Platform of Supervisory Organisations, EPSO) and the Psychiatric Section of the European Union of Medical Specialists (Union Européenne des médicins Spécialistes, UEMS).	To compare the use of coercive measures on psychiatric inpatients of different European countries	All countries allowed the use of forced medication in some form in 2009. The UK was the only country where mechanical restraint was not allowed and Denmark was the only country where seclusion was not allowed. Coercion was perceived differently across countries. Forced medication/long period was considered worst in Norway; forced medication/short period in Belgium; mechanical restraint in Finland, Iceland and France; seclusion in the UK; holding/physical restraint in Netherlands and mechanical restraint/ambulatory in Sweden, Denmark and Iceland. In the countries using both seclusion and mechanical restraint, mechanical restraint was regarded as the most intrusive.	Poor
Bak, et al. (2014) Denmark & Norway	Questionnaire/ Quantitative	Inpatient psychiatric hospital units in Denmark (n=87) and Norway (n=96)	Clinical nurse managers (n=90).	To investigate manual restraint-preventative practices and their association with the frequency of manual restraint episodes in psychiatric hospital units in Denmark and Norway	Three factors were found to be associated with lower rates of mechanical restraint: mandatory review (exp[B] = .36, $p < .01$), patient involvement (exp[B] = .42, $p < .01$), and no crowding (exp[B] = .54, $p < .01$).	Good
Bak, et al. (2015) Denmark & Norway	Questionnaire/ Quantitative	Inpatient psychiatric hospital units in Denmark (n=87) and Norway (n=96)	Clinical nurse managers (n=90).	To examine how manual restraint- preventive factors may be associated with the differing number of manual restraint episodes in Denmark and Norway.	Staff education [exp(B)= 0.34, P=0.00], was associated with a lower frequency of manual restraint episodes in Denmark. In Norway, three factors were associated with a higher frequency of manual	Good

For peer review only

					restraint episodes: cognitive milieu therapy [exp(B)=7.46, P=0.00], patient-centred care [exp(B)=5.01, P=0.00] and alarm systems [exp(B)=3.72, P=0.00]. Six factors were associated with the difference in manual restraint episodes between the two countries: identification of the patients' crisis triggers [exp(B)=- 10%], patient–staff ratio [exp(B)=-11%], staff education [exp(B)=-51%], acceptable work environment [exp(B)=-15%], substitute staff [exp(B)=-17%] and separation of acutely disturbed patients [exp(B)=13%].	
Barr, et al. (2019) Australia	Interviews/Qualitative	State Forensic Mental Health Service	31 registered and enrolled nurses	To: (i) document the experiences of nurses working in the forensic mental health setting, (ii) articulate their perceived unique skill set to manage challenging patient behaviours, and (iii) determine how their experiences and skill set can inform practice changes to reduce the use of restrictive practices.	Four categories: Working in a challenging but interesting environment, Specialty expertize, Exposure to aggression and resilience as a protective factor, Effective teamwork and leadership	Good
Bergk, et al. (2011) Germany	Randomised controlled trial/ Quantitative	A psychiatric hospital	Psychiatric inpatients in the hospital (n=102). Those randomly assigned to seclusion intervention (n=12). 75% male. Mean age 40.8 (SD=10.1) (range 23-61). Those randomly assigned to mechanical restraint intervention (n=14). 71% male. Mean age 38.6 (SD=12.0)	To explore opinions regarding the restrictiveness of seclusion and mechanical restraint on psychiatric patients	There was no significant difference in perceived coercion (CES scores) between the seclusion and mechanical restraint groups. No significant differences were found between the seclusion and mechanical restraint groups in the number of adverse events. The mechanical restraint	Fair

For peer review only

			(range 20-63). Those not randomly assigned to seclusion intervention (n=48). 38% male. Mean age 40.2 (SD=12.1) (range 19-66). Those not randomly assigned to mechanical restraint intervention (n=28). 46% male. Mean age 39.7 (SD=13.4) (range 18-64).		group experienced lower levels of fear then the seclusion group (mechanical restraint median score =1.00, range= 1–5; seclusion median score=2.25, range=1–5, p=.049).	
Bigwood & Crowe (2008) New Zealand	Descriptive phenomenological/ Qualitative	An acute adult psychiatric inpatient service	Registered comprehensive nurses and registered psychiatric nurses working in the service (n=7). 57% male.	To examine how nurses experience physical restraint of patients within an inpatient psychiatric service	The predominant theme was one of 'it's part of the job' and expected. However, participants describe being conflicted and fearful of physical restraint and prefer to use other management techniques.	Fair
Blair, et al. (2017) USA	Pre-post intervention/ Quantitative	Psychiatric inpatient service	Patient admissions pre-intervention (n=3884). 49.7% female.<br><br>Patient admissions post-intervention (n=8029). 48.5% female.	To describe an intervention designed to decrease seclusion and restraint and present the results of a pilot study that evaluated the effectiveness of this program.	Statistically significant associations were found between the intervention and a decrease in both the number of seclusions (p < 0.01) and the duration of seclusion per admission (p < 0.001). These preliminary results support the conclusion that this intervention was effective in reducing use of seclusion.	Fair
Bleijlevens, et al. (2013) Netherlands	Quasi-experimental and questionnaire/ Mixed methods	Psychogeriatric nursing home wards (n=15) in Dutch nursing homes (n=6)	Nursing home staff (n=143); Nurse specialists (n=2); delegates representing nursing home associations; relatives of nursing home residents (n=38 residents).	To assess an intervention to reduce the use of belt restraints in psychogeriatric nursing homes.	In more than 50% of the cases in which a belt was removed, no alternative interventions were used. The most frequently used alternative interventions were infrared barrier alarm systems (21%) and adjustable low-height beds (12%). 96% of participants felt that the intervention met their learning needs. 76% of the 38 resident's relatives did not agree with the use of belt restraints but thought it	Fair

For peer review only

					was necessary before the intervention, and 78% were satisfied with the policy change. 79% felt involved in the decision making process regarding belt restraint removal. Three barriers to implementation of the intervention were found: availability of preferred alternative interventions, removing all physical restraint at the same time (increasing risk), and time constraints.	
Bonner, et al. (2002) UK	Semi-structured interviews/ Qualitative	Psychiatric inpatient unit(s)	Staff in the units (n=12); patients in the units (n=6)	To explore the experiences of restraint from patient and staff perspectives in psychiatric inpatient units	Ward atmosphere and failed communication were seen as antecedents to restraint incidents. The incident itself was characterised by fear and embarrassment, with staff regarding restraint as a last resort. Debriefing and the need for understanding the incident were reported as helpful after a restraint incident. Both patients and staff spoke of the fear of re-traumatization and the difference in care from temporary and permanent members of staff.	Fair
Bonner & Wellman (2010) UK	Questionnaire/ Quantitative	Acute psychiatric inpatient units and PICUs within an NHS Mental Health Trust	Staff in the units (n=30), 57% female; patients in the units (n=30), 57% female	To explore the usefulness of post incident reviews after incidents of restraint in psychiatric inpatient units	97% of staff and 94% of patients reported that they found the post incident review process useful. The element that most participants agreed was useful was the opportunity for discussion after an incident (100% of staff; 93% of patients). 61% of staff and 20% of patients believed the incident could have been	Fair

For peer review only

					predicted. 67% of staff and 50% of patients believed the incident had been well managed.	
Boumans, et al. (2012) Netherlands	Questionnaire/Quantitative	Long stay wards (n=2); a forensic psychiatric ward; and a crisis intervention ward within a psychiatric hospital.	Staff in the units (n=60). 57% male.	To explore the importance of several factors in nurse decision making on seclusion and to explore the effect of reflexivity on the decision to seclude patients.	Approachability was found to be the patient variable with the greatest impact on likelihood of seclusion: if the patient was 'hardly approachable' the mean tendency to seclude was 0.52 higher than when they were deemed 'approachable' (95% CI 0.44, 0.60). The more reflexive a team was, the less likely they were to seclude (Pearson correlation coefficient -0.97, P = 0.017).	Fair
Bowers, et al. (2012) UK	Randomised controlled trial/Quantitative	Acute psychiatric wards (n=136) in 67 hospitals within 26 National Health Service (NHS) Trusts	All staff and patients within the units	To explore the relationship of manual restraint and show of force to conflict behaviours, containment methods, environment, routines and staff variables in acute psychiatric wards	Manual restraint was used less (0.20 incidents per day) than show of force (0.28 incidents per day). Both were associated with aggressive behaviours and the enforcement of treatment and detention. Staff provision was associated with the use of these coercive interventions. Clearer ward structure and routine was associated with decreased use of manual restraint and show of force.	Fair
Bowers, et al. (2007) UK, Netherlands, Finland, and Australia	Questionnaire/ Quantitative	Psychiatric inpatient settings	Psychiatric professionals (n=844). UK (n=114; modal age under 30; 61% female). Netherlands (n=146; modal age under 30; 65% female). Finland (n=304; modal age 30-39; 44% female). Australia (n=280; modal age 40-49; 67% female).	To explore the attitudes towards containment measures of psychiatric professionals in four countries	Staff in Finland expressed the highest level of approval of containment, and staff in the UK expressed the least. Preferences for different containment measures were influenced by whether they considered it: safe for the patients; preventative of injury to others; and rapidly	Fair

For peer review only

					calming.	
Brady, et al. (2017) Australia	Cross-sectional/Quantitative	Psychiatric hospital	Inpatients (n=625)	To examine patient experiences of restrictive interventions in inpatient settings	Most patients had experienced restrictive interventions (68%) and a third had experienced seclusion (35%). Reported benefits of restrictive interventions were lower in those who had experienced the most interventions.	Good
Braham, Heasley & Akiens (2013) UK	Questionnaire and semi-structured interviews/ Mixed methods	One admitting ward from four clinical services within a high secure hospital (male mental health, personality disorder, learning disability and the women's service)	Patients (n=31; 72% male; mean age 37; range 22-56) and staff (n=84) within the wards.	To evaluate the impact of night confinement in a high secure hospital	Night confinement was not shown to have any adverse effects. Before the pilot, the majority of staff (70%) felt that it could have adverse effects on patients, and the majority of patients (74%) felt that the effects would be minimal. After the pilot, 46% of staff felt that the impact had been minimal, and 58% of patients felt that the impact had been positive.	Fair
Chien, Chan & Kam (2005) China	Semi-structured interviews and clinical records/ Qualitative	Acute admission wards (n=2) within a psychiatric hospital	Psychiatric inpatients (n=30). Mean age 31	To explore the perspectives of psychiatric inpatients concerning the use of restraint.	The majority of participants reported positive feelings towards staff who had shown concern and had offered help during restraint. Negative effects of restraint were related to patient needs not being met. In particular: concern, empathy, active listening, and information about restraint.	Fair

For peer review only

Ching, et al. (2010) Australia	Questionnaires/Quantitative	Units within a secure forensic psychiatric hospital (n=5): male acute units (n=2); female acute unit (n=1); mixed-sex sub-acute unit (n=1); rehabilitation unit (n=1)	Phase 1: Staff (n=60; 50% female; modal age group 18-30) and patients (n=13; 31% female; modal age group 18-30). Phase 2: Staff (n=61; 48% female; modal age group 31-40) and patients (n=7; 29% female; modal age group 18-30).	To evaluate a group of interventions for reducing the use of seclusion in a forensic psychiatric hospital.	There was no change in levels of staff confidence to manage aggression from pre-post intervention. There was a significant reduction in reported absconding risk from pre-post intervention (F (1, 92) =4.2, p=0.04). Post-intervention, staff were more likely to report that seclusion was used therapeutically (t(112)=-2.41, p=0.02).	Good
Chu, et al. (2015) UK	Pre-post questionnaire/ Quantitative	Wards (n=6) within a high-secure forensic mental hospital	Patients (n=30). Mean age 39 (range 23-60). Ward staff: Night confinement respondents (n=144); EssenCES respondents (n=119)	To explore the views of staff and patients concerning a new night-time confinement policy within a high-secure forensic hospital	No significant differences from pre-post night-time confinement were found for patient sleep quality, behaviour, ward atmosphere, or therapy engagement. Patient attitudes towards night-time confinement generally became more positive from pre-post implementation. Staff attitudes remained largely negative, however, proportions decreased post-implementation.	Fair
Cormac, Russell & Ferriter (2005) UK	Questionnaire/ Quantitative	High secure hospitals and medium secure units (n=39)	Senior managers (n=39)	To explore the use of seclusion and seclusion policies and procedures in medium and high secure units	69% of the units reported using seclusion in the past year. All policies followed Code of Practice guidelines. Seclusion environment requirements varied. Additional requirements found in some policy documents were thought to potentially enhance practice: privacy and dignity, observation, fittings and fixtures, cleanliness, communication, physical health, ending of seclusion and contact with significant others.	Poor

For peer review only

Duxbury, et al. (2019) UK	Interviews/Qualitative	Acute mental health wards (n=14)	Staff (n=36)	To explore the views of staff regarding the value and impact of an intervention aimed at reducing levels of restraint (REsTRAIN Yourself)	Eight themes arose: leadership and impetus towards organizational change; data to inform practice; meaningful activities and events; changing hearts and minds; use of restraint reduction tools; patient voice and agency; debriefing techniques; and embedding and sustaining development	Fair
Elmer, et al. (2018) Germany and Switzerland	Questionnaires/Quantitative	Inatient departments of psychiatric clinics (n=5)	Mental health professionals (n=424). 62% female. Modal age group 26-35	To investigate mental health professionals' recognition, attitude, and application of informal coercion in psychiatric institutions	Stronger forms of coercion were more likely to be underestimated by respondents. Acceptance of coercive measures was predicted by use of the measure by the respondent, and the attitude that coercion is a form of treatment and not an offence	Fair
Elzubeir & Dye. (2017) UK	Pre-post intervention/ Quantitative	Psychiatric intensive care unit	Pre-intervention: patients (n=70). 51% male. Mean age 38.9. Post-intervention: patients (n=23). 74% male. Mean age 32.6	To investigate the effect on rate and duration of seclusion episodes when Specific, Measurable, Achievable, Reproducible, Time bound (SMART) targets are agreed with patients and health professionals for ending a period of seclusion.	Following the intervention, there was a reduction in the total number of seclusion episodes in all settings as well as a reduction in total seclusion time spent in both the Low Stimulus Area and the Locked Seclusion Room. We also observed a decline in the number of seclusion episodes in the less restrictive, longer-term seclusion setting (ECS) although overall there was a four-fold increase in the time spent in the longer-term seclusion setting.	Good

For peer review only

Espinosa, et al. (2015) USA	Pre-post evaluation/Mixed methods	Inpatient psychiatric units (n=15) within a large multi-site medical centre	Staff and patients	To evaluate interventions to improve milieu on psychiatric inpatient units	Restraint and seclusion rates reduced dramatically, but this meant that staff confidence in their skills regarding these interventions decreased. Violent incidents also reduced in frequency.	Poor
Exworthy, et al. (2001) UK	Questionnaire/ Quantitative	Maximum security hospitals (n=3) and medium security units	Psychiatrists and doctors (n=117)	To explore the views of doctors and psychiatrists concerning the practice of seclusion in secure units	56.4% of respondents agreed that seclusion is a form of treatment. 82% agreed that it was not a form of punishment. 60% felt that it helped avoid the use of medication and 86% support its use when a patient is threatening physical violence to others. 70% were against its use for self-harm behaviours. 45% felt that seclusion should be defined in law.	Poor
Ezeobele, et al. (2014) USA	Semi-structured interviews/ Qualitative	A psychiatric acute care hospital	Patients (n=20). 60% male. Mean age 28 (range 19-53)	To explore the psychiatric patients' lived seclusion experience.	60% of participants felt that seclusion was a penalizing and negative experience. Themes regarding the experience of seclusion were: feeling alone, staff exerting power/control, feeling resentful of staff, and having time for meditation.	Good
Faschingbauer, Peden-McAlpine & Tempel (2013) USA	Semi-structured interviews/ Qualitative	Psychiatric inpatient facilities	Psychiatric inpatients (n=12). 50% female. Mean age 33 (range 18-50)	To explore the psychiatric patients' lived seclusion experience.	Themes described by patients regarding the experience of seclusion were: hope for respect and communication, emotional response to seclusion (heightened anger/anxiety), and insight into behaviour and positive coping skills (need debriefing with staff).	Fair

For peer review only

Fish & Hatton (2017) UK	Interviews/Qualitative	Female wards (n=3) within a locked forensic learning disability unit	Staff (n=10) and residents (n=16; 100% female)	To explore women's experience of physical restraint in a secure learning disability setting	Three themes arose: gendered experiences of restraint, reasons for using restraint and alternatives to restraint	Fair
Fish (2018) UK	Interviews/Qualitative	Female wards (n=3) within a locked forensic learning disability unit	Staff (n=10) and residents (n=16; 100% female)	To explore the experiences of seclusion of women in a secure learning disability setting	Four themes arose: the seclusion room environment, reasons for using seclusion, termination of seclusion and alternatives to seclusion	Fair
Georgieva, Mulder & Wierdsma (2012) Netherlands	Questionnaire/ Quantitative	Acute psychiatric hospital	Psychiatric inpatients (n=161); 54% female.	To examine patient's experience and preferences regarding coercive interventions and associated factors	Previous experience of seclusion predicted preference for seclusion in emergency situations. However, most inpatients preferred medication in an emergency situation.	Fair
Georgieva, Mulder & Noorthoorn (2013) Netherlands	Experimental/ Quantitative	Acute psychiatric hospital	Psychiatric inpatients (n=520). Involuntary medication, (n=236); 48% male; mean age 40 and seclusion (n=284); 53% male; mean age 40	To examine the number and duration of seclusion and coercive measures relating to inpatients who are allocated to involuntary medication intervention compared with seclusion (TAU)	Relative risk (RR) of seclusion was lower for patients who received involuntary medication than TAU. Seclusion episode duration and the number of coercive incidents were not significantly different between the two groups.	Fair
Goulet and Larue (2018) Canada	Participatory case study/quantitative	Acute psychiatric hospital	Patients (n=3) and staff (n=14)	To explore the context in which seclusion and restraint practice take place.	Three overarching themes were identified: patient characteristics (e.g. etiology of violence), staff characteristics (e.g. feelings of safety) and environmental characteristics (e.g. overcrowding).	Good

For peer review only

Goulet, Larue and Lemieux (2018) Canada	Participatory methodology/Qualitative	Acute adult psychiatric care unit	Staff (n=12) and patients (n=3)	To develop and evaluate post-seclusion and/or restraint review (PSRR) intervention	Seclusion use was significantly reduced 6 months after the implementation (21.3% to 10.4%; p.046) but not for restraint (p=0.334).	Good
Gowda, et al (2018) India	Cohort/Quantitative	Psychiatric hospital	Inpatients (n=200)	To examine prevalence of coercive measures used in a psychiiatric hospital	Across the patients, 66.5% experienced more or one restraint measures, 58% experienced chemical restraint, 32% involuntary medication, 20% experienced physical restraint and 18% experienced seclusion.	Fair
Gowda, et al (2019) India	Cross-sectional/Quantitative	Psychiatric hospital	Inpatients and caregivers (n=200)	To examine caregivers perspectives on coercion practices.	Chemical restraint was deemed the most acceptable (82.5%) followed by physical restraint (71%) and seclusion (25.5%).	Fair
Gowda, et al. (2019) India	Questionnaire/Quantitativ e	Inpatient psychiatry	189 psychiatrists	To assess clinicians' attitude and perspective on the use of coercive measures in psychiatric practice.	Coercion proved to be a common measure applied in nearly 70% of the patients studied. The 189 psychiatrists participating in the study almost all perceived coercion as care, protection and safety, and as protection from dangerous situations. About 66% of psychiatrists perceived physical and chemical restraint (sedation) as necessary and acceptable in acute emergency care. One-third of the	Fair

For peer review only

					psychiatrists felt their patients lost autonomy, dignity, and the possibility of interpersonal contact. The same amount agreed that some patients could have been treated with less restriction and fewer coercive measures.	
Gustafsson & Salzmann-Erikson (2016) Sweden	Interviews/ Qualititative	Forensic psychiatric care	Nurses (n=8). 63% female. Mean age 44.5 (range 32-55).	To describe nurses' experiences and thoughts concerning the exertion of coercive measures in forensic psychiatric care.	Results described participants' thoughts and experiences of coercive measures from four main categories: (a) acting against the patients' will, (b) reasoning about ethical justifications, (c) feelings of compassion, and (d) the need for debriefing.	Good
Hatta, et al. (2007) Japan	Cohort/Quantitative	PICU	Psychiatric inpatients; restrained patients (n=106) and non-restrained patients (n=528)	To investigate the effects of physical restraint on the development of drug induced liver injury	Prevalence of drug induced liver injury was significantly higher for those who were restrained (8.5%) than the non-restrained group (1.9%; odds ratio 4.81). Rates of those receiving antipsychotics were higher in the restrained group.	Fair
Haugom & Granerud (2016) Norway	Descriptive/ Qualitative	Psychiatric wards (n=57)	Staff (n=149)	To investigate how psychiatric patients and staff describe and assess the practice of shielding	Shielding is an ambiguous practice. Shielding as a form of control was seen as more important than as a form of treatment.	Fair

For peer review only

Haw, et al. (2011) UK	Semi-structured interviews and case note review/ Mixed methods	Low and medium secure wards within a psychiatric hospital	Patients (n=57); 48% male; median age 29 (range 19-52)	To explore patients' experiences of, and preferences for, physical restraint, forced medication and seclusion	Coercive treatments were generally perceived as negative experiences; however, 16% of participants reported that the last episode of seclusion or restraint had been positive. Most patients preferred medication to seclusion. Patients felt that advance statements and views concerning restrictive practice should be a part of their care plans.	Fair
Holmes, Kennedy & Perron (2004) Canada	Phenomenological semi-structured interview study/ Qualitative	Specialised psychiatric unit	Psychiatric inpatients (n=6)	To explore the experiences of seclusion of patients with severe and persistent psychiatric disorder	Themes emerged: emotional experience of seclusion (feelings of rejection, isolation, etc.); patients' perceptions of seclusion (as punitive and a way of exerting social control), and coping mechanisms. The lack of staff contact during seclusion seemed to have more of a negative impact on patients than the seclusion itself.	Fair
Holmes, Murray & Knack (2015) Canada	Phenomenological semi-structured interview study/ Qualitative	Forensic psychiatric inpatient hospital	Participants (n=26): Forensic psychiatric inpatients (n=13) and forensic psychiatric nurses (n=13)	To study the lived experience of the secluded room	Three main themes from inpatients emerged: experience of seclusion, assessing quality of care and space of confinement. Themes from staff interviews were: resorting to seclusion, observing and assessing patients and experiencing seclusion. The therapeutic relationship is important to both staff and patients.	Fair
Hottinen, et al. (2012) Finland	Descriptive/ Quantitative	Adolescent closed ward of a general hospital	Clinical staff (n=128); 74% female; 75% nurses	To investigate staff attitudes towards containment measures used in adolescent psychiatric wards	Preferred containment methods were medication, transfer to specialist locked wards and mechanical restraint. The net bed was the least preferred containment method.	Fair

For peer review only

Hotzy, et al (2019) Switzerland	Cohort/Quantitative	Acute psychiatric inpatient hospitals (n=3)	Patients (n=418), staff (n=364) and patient relatives (n=180)	To compare and contrast perspectives of using coercive measures in psychiatric sites in Switzerland	Mean global attitude towards containment measures was 2.51 (SD=0.66). PRN medication was deemed the most acceptable across groups (M 1.62 ± 0.85). Stark differences were identified across the three sites in relation to attitude and use of containment measures in psychiatric hospitals.	Good
Huizing, et al. (2006) Netherlands	Experimental/ Quantitative	Psychogeriatric nursing home wards (n=5)	Residents with dementia (n=167); baseline (n=145); experimental (n=83); mean age 82; 78% female and post-intervention (n=144); experimental (n=86); mean age 82; 73% female	To investigate the effects of an educational intervention on the use of physical restraints in psychogeriatric nursing home wards	Despite education on the use of restraint, the use of restraint did not significantly decrease in the experimental group. Residents in the control group experienced significantly more restraint than the educational group.	Fair
Jaeger, et al. (2014) Switzerland	Cross-sectional/ Quantitative	Acute psychiatric hospital	Mental health professionals (n=39); 59% female	To evaluate how staff recognise different levels of coercion and treatment pressures	Results showed low levels of coercion were adequately recognised, whereas high levels of coercion were underestimated.	Fair
Jalil, et al. (2017) UK	Cross-sectional, correlational and observational design/ Quantitative	Secure mental health hospitals (n=3)	Mental health nurses (n=68). 70.6% female. Mean age 41.6 + 9.0	To identify relationships between mental health nurses' exposure to patient aggression, their emotions, their attitudes towards coercive containment measures, and their involvement in incidents involving seclusion and restraint	Nurses who reported greater exposure to a related set of aggressive behaviours, mostly verbal in nature, which seemed personally derogatory, targeted, or humiliating, also reported higher levels of anger-related provocation. Exposure to mild and severe physical aggression was unrelated to nurses' emotions. Nurses' reported anger was significantly positively correlated with their endorsement of restraint	Good

For peer review only

					as a management technique, but not with their actual involvement in restraint episodes. Significant differences in scores related to anger and fatigue, and to fatigue and guilt, between those involved/not involved in physical restraint and in physical restraint plus seclusion respectively were detected.	
Johnston & Kilty (2016) Canada	Interviews/Qualitative	Psychiatric units (n=2)	Security guards (n=8). 100% male	To explore how security guards use neutralization to shift responsibility during violent encounters on to medical staff in psychiatric units	Themes drawn from the interviews included: mitigation of responsibility through discourses of blame; refusing injury: ''It's for their own good''; constructing patients as dangerous and deserving of punishment; condemning the nurses; and appealing to militant codes of conduct	Fair
Keski-Valkama, et al. (2007) Finland	Descriptive/ Quantitative	Psychiatric hospitals	Patients restrained in 1990 (n=94), 1991 (n=107), 1994 (n=71), 1998 (n=59), 2004 (n=59). Patients secluded in 1990 (n=75), 1991 (n=43), 1994 (n=86), 1998 (n=40), 2004 (n=36)	To examine the use of restraint and seclusion over time	The number of seclusion and restraint incidents decreased over the five time periods; however the duration of seclusion significantly increased over time (v2 (4) = 36.111, $p < 0.001$).	Fair
Kirkevold & Engedal (2004) Norway	Descriptive/ Quantitative	Regular units (RU) for patients with dementia (n=142). Special care units (SCU) for inpatients with dementia (n=80)	Staff interview details not recorded. Interviews based on inpatients (n=1501): RU (n=1057); 66% female; mean age 84.4 [6.9]; SCU (n=444); 75% female; mean age 83.4 [6.1]	To identify the prevalence of patient restraint in nursing homes	Significantly more restraint occurred in SCUs than in RUs (45.0% and 36.7% respectively). The most common restraint interventions were mechanical restraint and use of force/pressure in daily activities.	Fair

For peer review only

Knowles, Hearne & Smith (2015) UK	Semi-structured interview/ Qualitative	Medium secure unit	Inpatients (n=8); mean age 39; 13% female	To explore the impact of employing physical restraint on maintaining therapeutic relationships between staff and patients	Five themes were found: Restraint reinforces the inequality of power in the staff-patient relationship; abusive, degrading and traumatic experiences; whether restraint justification influences whether it is an accepted intervention; negative attitudes and motives of some staff; and coping with having no power during and after restraint.	Fair
Kontio, et al. (2012) Finland	Interviews/ Qualitative	Psychiatric inpatient hospital	Inpatients (n=30); 37% female; mean age 41 (range 20-64 years)	To explore patient perspectives on the use of, and future directions of restraint and seclusion	In general patients had negative experiences during seclusion/restraint and did not have enough information before it happened regarding why the intervention occurred. Future improvement ideas included recent information about their care plan and treatment, and reasons why they were restrained. Alternatives to restraint and seclusion included empathetic patient–staff interaction, meaningful activities, therapeutic community, and biological treatments.	Fair
Kontio, et al. (2011) Finland	Experimental/ Quantitative	Psychiatric inpatient wards in general hospital (n=12)	Nurses (n=158); intervention group (n=95); 55% female; mean age 43 [9.0] (range 25-60) and control group (n=63); 52% female; mean age 45 [10] (range 24-64)	To examine the effect of an online learning course on nurse competence in restraint and seclusion interventions	Knowledge of physical restraint improved at three month follow-up whereas knowledge of seclusion did not change. Self-efficacy increased in the intervention group.	Fair
Kontio, et al. (2009) Finland	Focus group/ Qualitative	Psychiatric hospital (n=2)	Staff (n=27); 52% female; mean age 44 years (range 26-59)	To examine staff perspectives on current educational needs in relation to implementing seclusion and restraint, and their future needs	Difference found on guidance regarding seclusion and restraint: some staff wanted structured guidelines and some wanted to rely on knowledge/experience. All seemed to want more education regarding the ethical, clinical and legal	Fair

For peer review only

					aspects of restraint and seclusion interventions. This was in addition to a desire for staff support to ensure the success of the intervention.	
Kontio, et al. (2010) Finland	Focus group/ Qualitative	Psychiatric hospital (n=2)	Staff (n=27); 52% female; mean age 44 years (range 26-59)	To explore what happens when an aggressive incident occurs in a psychiatric ward and what alternatives to restraint and seclusion are possible	Staff felt that the patient perspective was not considered enough. Staff also proposed numerous alternatives to seclusion and restraint including being present and having a conversation with the patient. Cooperation and communication via multi-professional agreements that involve the patient perspective were deemed useful.	Fair
Krieger, et al. (2018) Germany	Naturalistic/quantitative	Psychiatric intensive units (n=3)	Involuntary patients (n=213) and voluntary patients (n=51)	To examine patient's attitudes towards coercion	Perspectives varied aross the four different types of coersion (mechanical restraint, forced medication, seclusion, involuntary hospitalisation). The majority of patients felt helpless and desparate particularly during mechanical restraint (80.6% and 75% respectively) and seclusion (90.0% and 73.7% and respectively). Patients reported that they would prefer a soft room and least prefer mechanical restraint when "coercive interventions against my will are neccessary" (M=2.46 ± 1.26, M=3.19 ± 1.05)..	Good

For peer review only

Kuosmanen, et al. (2015) Finland	Experimental/ Qualitative	Acute psychiatric hospital	Staff (n=2)	To explore the experience of being secluded and to understand and evaluate the impact of seclusion from a staff perspective.	Overall, staff perceived their seclusion experience as negative and inhumane. The staff felt anxiety and frustration at being locked up. Future practical suggestions for change included updating seclusion practice guidance and re-designing seclusion facilities. Ideas included introducing a clock and normal height bed and chair.	Poor
Larsen & Terkelsen (2014) Norway	Observational/ Qualitative	Locked psychiatric ward	Inpatients (n=12). Staff (n=22)	To explore coercion experience	There were four main themes: corrections and house rules, coercion as a necessary intervention, material surroundings as being of great importance, and being treated as a human being. Staff and patients differed in their experience. Patients felt inferior, and staff sometimes felt guilty when implementing the interventions.	Fair
Larue, et al. (2013) Canada	Structured interview/ Mixed methods	Psychiatric hospital	Inpatients (n=50); 38% female	To identify and describe perceptions of the seclusion and restraint protocol in a psychiatric hospital	Just over half of inpatients indicated they were not offered an alternative to restraint (n=28). Eighteen patients felt seclusion fulfilled their need for calm, sleep and safety. Almost all said staff did not follow up with them after their seclusion/restraint experience and deemed this as an essential need in the future.	Fair

For peer review only

Larue, et al. (2016) Canada	Focus groups/Qualitative	A psychiatric hospital intellectual disability programme	Staff (n=24) and family members (n=7)	To explore how staff and family members of psychiatric inpatients feel about reductions in coercive measures and the interventions used by staff that contributed to this reduction	Four themes arose: leadership; organisational culture; characteristics of the care providers; and characteristics of the patients	Fair
Lee, et al. (2003) UK	Descriptive/ Mixed methods	Secure inpatient mental health unit and PICU	Nurses (n=269)	To examine staff views of their last experience of employing physical restraint	The majority of nurses reported positive results after the coercive incident. Issue raised regarding the after effects of the incident were concern and ambivalence. Negative aspects of interventions included injury, management and clinical issues. Future improvements reported included less crowed environment, improved staff training in de-escalation and adopting positive care philosophy around relationships with patients	Poor
Lee, et al. (2001) UK	Cross-sectional/ Quantitative	Regional secure inpatient mental health units and PICUs (n=63)	Nurses (n=338); 53% female; mean age 36.2 [8.9] (range 19-63)	To investigate training in physical restraint in order to compare course content and length, and injuries in training	Course content varied despite a core curriculum in place. Length of course also differed (range 0.5-21 days) but two thirds attended a 5 day course. Sometimes refresher courses did not happen as expected. About a third of respondents reported being hurt in training.	Poor
Long, et al. (2015) UK	Pre-post intervention design/ Quantitative	Medium secure inpatient unit	Patients (n=38). Experimental group (n=19), and matched control group (n=19); mean age 31.1 (range 19-49); 100% female.	To assess the effectiveness of interventions designed to minimise the use of seclusion in response to risk behaviours.	There was a significant decrease in the number of seclusion incidents and risky behaviour post-intervention change, as well as more positive staff ratings of patient behaviour, improved treatment engagement	Fair

For peer review only

					and reduced patient time spent in medium security. Staff perceived training and use of de-escalation techniques as the most effective whereas patients felt individual engagement and initiatives to reduce bullying, harassment and discrimination was the most effective.	
Looi, Engstrom & Savenstedt (2015) Sweden	Self-report/Qualitative	Psychiatric inpatient unit	Inpatients (n=19)	To explore the perceptions of people who self-harm regarding alternatives to coercive measures and how this relates to lived experiences of psychiatric care	Content analysis revealed three main themes: understanding rather than neglect, mutual relation rather than distrust, and professionalism rather than counterproductive care.	Fair
Lovell, Smith & Johnson (2015) UK	Semi-structured interviews/ Qualitative	Secure learning disability service	Nurses (n=20)	To explore the views of learning disability nurses regarding physical intervention incidents, and contributory factors of injuries sustained	The overarching theme, knowledge and understanding, placed three other themes in context: physical intervention techniques employed, staff interpretation of the safety incident and the impact on staff. All staff felt it was important to know the patient and use an individualised approach when an incident occurred.	Fair
Mackay, Paterson & Cassells (2005) UK	Unstructured interviews/ Qualitative	Sector Acute Psychiatric Admission wards (n=3) and PICU (n=1)	Nurses (n=6)	To explore the perceptions of observing for patients with perceived risk of violence/aggression and important factors in day to day practice	Procedure, skills and role were identified as three higher level categories. An additional six categories made up "role": intervening; maintaining patient safety and that of others; prevention de-escalation; and managing aggressive and violent incidents; assessment; communication; and therapy. Staff skills were built within this and	Fair

For peer review only

					deemed interconnected.	
Martello, et al. (2018) Canada	Interviews/Qualitative	An inpatient psychiatric unit	Nurses (n=6). 84% female	To explore the views of nurses towards engaging with patients to reduce the use of restrictive practices in an inpatient psychiatric unit	Four themes arose: unit engagement practices, managing the escalation by engaging with patient, engaging during the use of restrictive practices, and factors influencing engaging with patient to reduce restrictive practices	Good
Mason & Whitehead (2001) UK	Structured interview/ Mixed methods	Forensic psychiatric inpatient hospital	Female inpatients	To explore the problems associated with secluding female inpatients	There were 34 seclusion incidents with the longest duration identified as 23 days, 8 hours and 45 minutes. The most common reason for seclusion was actual violence to staff (928 hours), threat of violence (238 hours) and threat of property damage (129 hours). Staff rationale for stripping patients while in seclusion was for patients' safety. PRN medication was usually given before a seclusion.	Fair
McKeown, et al. (2019) UK	Interviews/ Qualititative	Acute mental health wards (n=14)	Staff (n = 130). 52% male.<br>Service users (n = 32). 47% male.	To explore views on staffing levels in a context of attempting to minimize physical restraint practices on mental health wards.	Five themes were identified regarding how staffing levels impact experiences and complicate efforts to minimize physical restraint. Themes—“insufficient staff to do the job”; “detriment to	

					staff and service users"; "a paperwork exercise: the burden of non-clinical tasks"; "false economies"; and, "you can't do these interventions."	
Mistler, et al. (2017) USA	Pre-post evaluation/Mixed methods	An acute care state hospital	Inpatients (n=13). 83% male; 92% white; mean age 33 [SD 10.7]	To examine the feasability, usability and acceptability of a mindfulness mobile app intended to reduce aggression in psychiatric inpatients	All participants reported the app to be easy to use and engaging. 83% of participants felt comfortable using the app and would recommend it to others. Participants reported that the app gave them a sense of control and alleviated boredom	Fair
Molewijk, et al. (2017) Norway	Cross-sectional/ Quantitative	Acute ward (n=2)	Mental health care professionals (n=379). 40% male.	To examine the following: 1). What are mental health care professionals' normative attitudes towards coercion and how often do they experience moral doubt? 2). How is health care professionals' experienced moral doubt related to their normative attitude towards coercion? 3). How are professional and contextual characteristics related to the staff's normative attitude towards coercion and to experiencing moral doubt?	On average employees neither agreed nor disagreed with the statements that described coercion as offending, respondents from Acute Care (Hospital 2) seemed to agree a bit more that coercion could be seen as offending. On average, respondents from all wards agreed that coercion could be seen as care and security.	Good

Muir-Cochrane, et al. (2015) Australia	Semi-structured interviews/ Qualitative	Old age psychiatric unit (n=3)	Mental health nurses (n=39)	To explore nurses experiences of restraint and seclusion and resistance to getting rid of these interventions	"Lack of accessible alternatives to restraint and seclusion" was the overarching theme. Interrelated themes covered environmental factors contributing to restraint and seclusion interventions; the consequences of poor staff-patient relationships; and the influence of ward environment on restraint and seclusion intervention implementation (an unfavourable physical environment impacts upon the aggression, restraint and seclusion tactics used).	Fair
Newman, Paun & Fogg. (2018) USA	Pre-post intervention and survey /Mixed methods	Adult inpatient psychiatric unit	Staff members (n=35) who completed the 3-month post-survey	To evaluate the effects of a 90-minute trauma-informed care-based staff training on patient seclusion rates in an adult psychiatric unit.<br><br>To evaluate staff knowledge and attitudes about seclusion trauma, commitment to seclusion alternatives, confidence in personal de-escalation skills, and use of new de-escalation.	Seclusion rates were reduced from a 6-month preintervention average of 2.95 seclusion hours per 1,000 patient hours to a 6-month post-intervention average of 0.29 seclusion hours per 1,000 patient hours, a 90.2% reduction.<br><br>Completed staff surveys showed significant staff knowledge gains, non-significant changes in staff attitudes about seclusion, non-significant changes in staff de-escalation skill confidence, and use of the new resource sheet by only 17% of staff .	Fair

For peer review only

Nielsen, et al. Denmark (2018)	Focus groups/ Qualititative	Forensic mental health setting including secure units (n=4) and rehabilitation unit (n=1)	Clinicians (n=17) composed of nurses (n=8), social and healthcare assistants (n=8) and nurse assistant (n=1). 76% female. Age range 31-60	To report on clinicians' experiences of the clinician-patient alliance during mechanical restraint (MR), including what and how parameters of alliance are assessed regarding the patient's readiness to be released from MR.	The results show that a pre-established personal clinician-patient alliance formed the basis for entering into, and weighing the quality of, the alliance during MR. In consideration of the patient's psychiatric condition, the clinicians observed and assessed two quality parameters for the alliance: 'the patient's insight into or understanding of present situation' (e.g. the reasons for MR and the behaviour required of the patient to discontinue restraint) and 'the patient's ability to have good and stable contact and cooperation with and across clinicians.	Fair
Papadopoulos, et al. (2012) UK	Cross-sectional/ Qualitative	Acute inpatient psychiatric wards (n=16)	Ward managers and consultant psychiatrist (n=120)	To investigate what influences the likelihood of patient conflict (verbal abuse, violence, and rule breaking) and containment (seclusion, manual restraint, and enforced medication)	Negative staff morale increased the likelihood of conflict and containment, whereas positive staff practice (environment improvement, increased staff activity, proactive ward manager) decreased the likelihood of such events occurring.	Good
Pellfolk, et al. (2010) Sweden	Cluster-randomized controlled trial/ Quantitative	Group dwelling units for people with dementia (n=40)	Control group at baseline: Residents (n=162); mean age 83.4 and staff (n=162); mean age 43.2. Intervention group at baseline: Residents (n=191); mean age 80.5 and staff (n=184); mean age 43.5	To evaluate a restraint minimization education program.	In the intervention group, staff had more knowledge about restraint, but attitudes toward restraint use were not significantly different from the control group. The likelihood of being restrained was lower in the intervention group (OR) =0.21, 95% (CI) =0.08–0.57, *P*=.002, n=281).	Good

For peer review only

Perkins, et al. (2012) UK	Semi-structured interviews and focus groups/ Qualitative	An acute adult mental health setting	Nursing staff (n=30)	To explore the attitudes of staff towards restraint.	Factors thought to influence the decision to restrain were: contextual demands; lack of alternatives; the escalatory effects of restraint; and perceptions of risk. Nurses described restraint as a “necessary evil” due to the unpredictability of mental illness and their work environment.	Fair
Raboch, et al. (2010) Europe and Israel	Questionnaire/ Quantitative	Psychiatric inpatient facilities	Patients (n=2,030); age range 18-65.	To compare the use of coercive measures in psychiatric inpatient facilities in different European countries.	1,462 coercive measures were used with 770 patients (38%). 21%-59% of patients received coercive measures, with the most common reason being patient aggression against others. In eight of the ten countries, the most common measure was forced medication, and in two of the countries it was mechanical restraint. Seclusion was rare (only 6 countries reported it).	Fair

For peer review only

Raveesh, et al (2016) India	Questionnaire/ Quantitative	Department of Psychiatry	Psychiatrists (n=210). 80% male. Caregivers (n=210). 54.4% male.	To assess attitudes of Indian psychiatrists and caregivers toward coercion.	Both groups agreed that coercion was related to scarce resources, security concerns, and harm reduction. Both groups agreed that coercion is necessary, but not as treatment. Older caregivers and male experienced psychiatrists considered coercion related to scarce resources to violate patient integrity. All participants considered coercion necessary for protection in dangerous situations. Professionals and caregivers significantly disagreed on most items.	Good
Reisch, et al (2018) Switzerland	Cross-sectional/Quantitative	Psychiatric hospital (n=3)	Patients (n=435), Staff (n=372) and patient relatives (n=230)	To compare and contrast perspectives of using coercive measures in a psychiatric hospital	34.7% of patients had experienced severe coercion; of these, the measures were rated less acceptable than those who hadn't had that experience ($t=3.15$, $p=0.002$). Staff felt coercive measures were more acceptable than patients or their family members. Patients also strongly rejected intramusclar injection medication.	Fair

For peer review only

Reynolds, et al. (2016) USA	Naturalistic/quantitative	Youth psychiatric inpatient setting	Young inpatients (n=1485)	To examine the effectiveness of the Positive Behavioural Interventions and Supports (M-PBIS) to reduce seclusion and restraint in a youth psychiatric inpatient setting	The number of seclusion and restraint incidents significantly reduced after using the intervention (pre=142 to post=102; p<0.001). The number of prescribed PRN medications also significantly reduced (pre=301 to post=223; p<0.001).	Fair
Rippon, Reid & Kay. (2018) UK	Focus groups and interviews/ Qualititative	CYP (children and young people) psychiatric inpatient services	Health professionals (n=11), non-clinical staff (n=6), service users and relatives (n=9)	To gain an understanding of the attitudes of frontline health professionals, non-qualified staff, patients and relatives regarding the use of restrictive practices to manage behaviours that challenge in CYP psychiatric inpatient services.	Although restrictive practices were sometimes seen as necessary to manage certain situations – for instance, when staff felt there was a risk of a patient committing suicide – they were also considered to be potentially harmful to staff and patient wellbeing.	Fair
Ryan & Bowers (2005)	Cross-sectional observational/ Qualitative	A PICU in an inner city hospital	Staff nurses and patients	To explore the implementation of coercive manoeuvres in a PICU.	Within the PICU, nurses practised several coercive strategies, which enabled them to manage low-level conflict situations. More serious conflict necessitated	Fair

For peer review only

					more severe containment methods. For example, 'time out' was used regularly (confinement in the patient's own bedroom or in an open seclusion room), as well as seclusion.	
Ryan & Happell (2009) Australia	Participatory action research/Qualitative	Adult acute mental health inpatient units with high-dependency unit suites and seclusion facilities (n=2)	Mental health nurses (MHN) (n= 31) and consumer consultants (CC) (n=4)	To describe current practice and debriefing needs of staff, in order to gauge the need for a training program to facilitate post-seclusion debriefing.	Themes emerged from the data: 1) Debriefing, support, and flexibility- MHNs stated that debriefing consumers after seclusion is important. 2) Inherently unethical? Some CCs had concerns around being involved in a debriefing program, for example: "Seclusion is not an evidence-based intervention, it is a breach of human rights" 3) Support us, don't preach to us- debriefing should be about providing psychological support. Consumer choice is also important.	Good
Schreiner, Crafton & Sevin (2004) US.	Pre-post intervention study/ Mixed methods	An adolescent inpatient unit	Inpatients (n=23); 56% male; age range 13-17.	To describe the effects of a staff re-education campaign in reducing the use of physical restraints treating adolescents with developmental delays and severe psychiatric disturbances	During the assessment phase, a monthly average of 18.67 seclusion events were recorded. During the intervention phase, monthly seclusion events declined to 12.14, a decrease of 35%.	Fair
Seo, Kim & Rhee (2012) South Korea	Descriptive/ Quantitative	Psychiatric hospital	Inpatients (n=248); 38% female; mean age 38.6 [11.4]	To examine whether coercive interventions can be justified by the assumption of incompetence, the assumption of dangerousness and the assumption of impairment	Legal status, perceived coercion and experienced coercive measures were justified as measures of coercion under the assumption of incompetence.	Poor

For peer review only

Sequeira & Halstead (2001) UK	Interviews/ Mixed methods	Secure wards in a psychiatric hospital (n=5)	Patients (n=116); 22% female; age range 13-54.	To investigate the use of coercive measures with patients who have developmental disabilities and to explore the experience of these measures.	Women had a significantly higher probability of being given rapid tranquilization following violent incident, seclusion was more likely to be used with men. Interviews with women demonstrated a commonly held understanding of interventions as punishment and expressions of intense anger and anxiety.	Fair
Sequeira & Halstead (2004) UK	Cross-sectional / Qualitative	Secure mental health service	Nursing staff (n=17); 53% male; age range 18-50. Restrained patients (n=14) and patients who observed the same event (n=5)	To examine the experiences of physical restraint processes reported by nursing staff	A range of emotional responses by nursing staff included distress, anxiety and crying. These were sometimes corroborated by patient descriptions. Staff used laughter to reduce stress following an incident, and many reported feeling little emotion during incidents, instead taking any distress home.	Fair
Smith & Jones (2014) UK	Descriptive and semi-structured interviews/ Mixed methods	A PICU within an inner city heath service	Staff members (n=10); 90% male. Patients (n=7); 100% male	To explore the effects of a sensory room on a PICU on seclusion rates and staff and patients' experiences	Interviews revealed a perception by staff that seclusion rates had reduced, despite no significant reduction occurring. Both staff and patients expressed that the sensory room was positive and therapeutic, improving staff-patient communication and patients' experience of the PICU.	Fair

For peer review only

Steinert, et al. (2007) Germany	Prospective/ Quantitative	Psychiatric hospitals (n=10)	Treated cases (n=36,690)	To investigate the prevalence of coercive measures in different psychiatric hospitals	9.5% of 36,690 cases were exposed to coercive measures. On average, these measures were applied 5.4 times per case and lasted 9.7 hours each. The use of seclusion and restraint guidelines was associated with a lower incidence of coercive measures.	Fair
Sustere & Tarpey (2019) UK	Interviews/Qualitative	A medium secure unit within an NHS hospital	Inpatients (n=12). 100% male	To explore patients' experiences of least restrictive practice and it's relationship to independence and recovery	Five themes arose: positive changes, perceived lack of transparency, social isolation, institutionalisation, and normality	Fair
Tateno, et al. (2009) Japan	Cross-sectional vignette study/ Quantitative	Psychiatric inpatient facilities	Young psychiatrists (n=183)	To explore Japanese psychiatrists' attitudes about emergency coercive interventions for patients with acute psychosis.	Most participants agreed that the vignette case should be admitted to hospital and secluded. Regarding the likelihood of prescribing restraint, results varied; psychiatrists at general hospitals were more likely to prescribe restraint than those working at university or psychiatric hospitals.	Fair
Tompsett, Domoff & Boxer (2011) USA	Prospective/ Quantitative	A secure psychiatric hospital	Adolescents (n=149); 59% male; mean age 14 (SD=3)	To investigate predictors of aggression in adolescent inpatients.	Unique predictors of restraint involvement were found: history of aggression against adults and history of previous psychiatric hospitalizations. No predictors were significant for the extent of restraint involvement.	Good

For peer review only

Vedana, et al (2018) Brazil	Semi-structured interviews/Qualitative	Psychiatric units (n=2)	Nurses (n=29)	To explore and understand nurses experiences and perceptions of using physical restraint.	Almost all staff had had training about how to perform physical restraint (93.1%) but over half had experienced restraint related accidents (58.2%). Three main themes were identified: agressiveness and restraint: unpleasant, challenging and harmful situations; the need and purpose of the physical restraint and strategies to reduce physical restraint-related damage.	Fair
Wharewera-Mika et al. (2016) New Zealand	Focus groups/Qualitative	District health boards (n=4)	Cultural advisors (n=5), Maori nurse leaders (n=5), Maori consumer advisors (n=2), Maori elders (n=4)	To explore Maori views of initiatives to reduce or prevent seclusion of mental health inpatients	Three themes arose: access to a Maori wordview; transforming practice; and Rangatiratanga (leadership, power, and control)	Fair
Whitecross, Seeary & Lee (2013) Australia	Interventional/ Quantitative	Inpatient wards	Patients in the TAU group (n=14); 71% male; mean age 35.8 (SD=9.9). Intervention group (n=17); 76% male; mean age 37.8 (SD=10.1)	To identify the impacts of seclusion on patients and evaluate the effectiveness of a post-seclusion counselling intervention in reducing time spent in seclusion and trauma experienced.	There was no difference in trauma experience between the TAU group and post-seclusion counselling intervention group.	Fair
Whittington, et al. (2009) UK	Cross-sectional/ Qualitative	Acute care mental health services	Service users (n=1,361); 67% female. Staff (n=1,226); 48% female	To ascertain the degree of approval amongst service users and staff for commonly used coercive measures in acute mental health care	Both service users and staff disapproved strongly of mechanical restraint and expressed a relative preference for compulsory intramuscular medication and seclusion. Male staff, older service users and staff who had implemented coercion reported greater approval of coercive measures.	Fair

For peer review only

Wilson, et al. (2017) UK	Interviews/Qualitative	Adult mental health inpatient	13 patients and 22 staff members	To feed key findings into a coproduced, evidence-based proactive care toolkit that eliminates reliance on restraint in mental health care	One main theme: Is restraint a necessary evil? Two subthemes: It never is very nice' and 'It's got to be done. . .it's a necessary evil'	Fair
Wilson, et al. (2018). UK	Interviews/ Qualititative	Adult mental health inpatient wards	Inpatients (n=13). 54% female. Age range 18-65. Staff members (n=22). 68% female. Age range from early 20s to late 50s.	To explore mental health patients' and staff members' suggestions for reducing physical restraint.	Findings centred on four overarching themes: improving communication and relationships between staff/patients; making staff-related changes; improving ward environments/spaces; and having more activities. However, concerns were raised around practicalities/feasibility of their implementation.	Good

For peer review only

Safety culture

Author, year, country	Study design	Setting	Participants	Aims/ objectives related to patient safety	Outcomes related to patient safety	Study quality
Ajalli, et al. (2018) Iran	Interviews/ Qualititative	Acute care wards (n=8)	Supervisors (n=2), head nurses (n=4) and registered nurses (n=13). 47% female. Median age 38.	To focus on nurses' opinions and experiences about patient safety in inpatient psychiatric wards	Nurses' experiences are reflected based on the main theme, including "intelligent care and protection from risk creators." Two themes and eight related categories were emerged including "vigilant care (warning of highrisk patients, curious attention, care of vulnerable patients, early intervention) and "close observing (confronting with dangerous concealment, access control, objective observation, continuous observation)".	Good
Bowers, Gournay & Duffy (2000) UK	Questionnaire/ Quantitative	A random stratified sample of NHS Trusts providing psychiatric inpatient services in England and Wales (n=27).	Directors of Nursing for these Trusts	To explore the observation policies and usage in psychiatric inpatient services	12% of the services had no written observation policy, and 38% had no clinical recording system. Only two policies studied used the same terminology for the different levels of intensity of constant observation (CO). There was variation across Trusts in who was qualified to undertake CO and whether agency staff were employed to do this. Gender of staff was a consideration for most Trusts when deciding who to allocate to CO. Most policies stated that patients should be given a rationale for CO. The most agreed upon reason for using CO was	Fair

For peer review only

					to reduce harm/suicide.	
Bowers, et al. (2006) UK	Questionnaire/ Qualitative	14 acute psychiatric wards and 3 psychiatric intensive care units across 3 hospital sites, within 1 NHS Trust	Staff from these wards (n=56). Ward Managers (n = 16), F Grade mental health nurses (n = 17), Occupational Therapists (n = 14) and Consultant Psychiatrists (n = 9).	To explore the impact of serious incidents on inpatient psychiatric wards	Staff reported feelings of shock, depression, demoralisation, loss, and grief after incidents, with periods of rumination, guilt and anxiety. Following incidents, levels of containment increased, along with the focus on risk assessment. Staff reported the following as making it difficult to emotionally process the incidents: the pace of ward life, a lack of external support, and management investigations. Patient responses to the incidents were rarely acknowledged.	Fair
Brennan, Flood & Bowers (2006) UK	Observation and action research/ Qualitative	Generic acute admission wards (n=2)	City Nurses within the wards (n=2). All staff and patients within the wards	To explore change implementation issues in psychiatric acute admission wards	Barriers to change were: limited staffing resources, the physical environment, insufficient beds, unclear hierarchical structure and multidisciplinary issues, over demanding ward managers, and anxiety about serious incidents.	Fair
Cowan, et al. (2018) Australia	Participant observations, focus groups and interviews/ Mixed methods	Acute mental health inpatient units (n=2)	Patients (n=233) Nurses (n=36)	To develop a guideline to support uniform structure and process in mental health nursing handover.	Data collected as part of that investigation contributed to the development of a handover guideline that incorporates the key components of structure, content, and leadership. The research indicated a link between these components, and further revealed the necessity to have other forums, such as supervision and clinical review, to ensure that handover serves its	Fair

For peer review only

					intended purpose in an efficient manner	
Cullen, Nath & Marcus (2010) USA	Semi-structured interviews/ Qualitative	Inpatient psychiatric units within urban teaching hospitals (n=2)	Staff (n=17). 53% female	To explore the typology of errors and precipitating factors in inpatient psychiatry	Preventive errors (such as falls, suicide) were mentioned the most (38% of errors mentioned). Provider factors were most commonly attributed to the precipitation of errors (74% of factors mentioned). Most of the broad typologies and precipitating factors echo those found in general medicine and surgery, whilst the specifics are unique to inpatient psychiatry.	Fair
Delaney & Johnson (2006) USA	Observation and semi-structured interviews/ Qualitative	Inpatient psychiatric units (n=2)	Staff (n=16; age range 22-50+) and patients (n=12; 50% female; age range 22-56)	To explore de-escalation processes and the skills of staff in inpatient psychiatric units to create a safe environment	Staff behaviours can create a safe milieu and positive culture. Important skills for maintaining safety were: awareness, attending, caring, and connecting.	Fair
Gabrielsson, et al. (2014) Sweden	Focus groups/Qualitative	The inpatient ward of a psychiatric clinic	Staff (n=26). 54% female.	To explore staff perceptions of inter-professional collaboration relating to challenging situations in psychiatric inpatient care.	Physicians were described as distant decision-makers, ward managers as suspicious supervisors, psychiatric nurses as mediating moderators, and nursing assistants as informed performers. Expectations of staff during challenging situations were: to talk to each other, control the situation, know the patient and set the stage. The common thread throughout all discussion was recognising knowledge of the patient as decision-making power in challenging situations.	Fair

For peer review only

Gerace, et al. (2018) Australia	Interviews/Qualitative	Acute psychiatric settings	13 nurses, 7 consumers	To investigate how empathy is developed and maintained when there is conflict between nurses and consumers, and the ways in which empathy can be used to achieve positive outcomes.	Antecedents of the empathy experience (MY role as a nurse - the orle of my nurse, What nurses and consumers brought to the situation, the sitution) Processes (Perspective taking as trying to understand); Intrapersonal outcomes (Feelings for the consumer); Interpersonal outcomes (being there, Empathic relationshop withstand conflict, Empathy inflences nurse-consumer satidfaction).	Fair
Gifford & Anderson (2010) Canada	Nominal group consensus technique/ Qualitative	Inpatient psychiatric mental health care facility	Healthcare nurses with inpatient nursing experience (n=16); 75% female; mean age 49	To identify staff attitudes to reporting assault in an inpatient psychiatric setting	Safety culture was the predominant factor in deciding whether to report an incident. The design of the reporting system, and the perceived effect on patients were also deemed important when making a reporting decision.	Fair
Happell & Koehn (2011) Australia	Descriptive/ Quantitative	Acute inpatient unit	Psychiatric nurses (n=123); 51% female; mean age 41 (range 20-62)	To investigate associations between burnout, job satisfaction and therapeutic optimism, and seclusion attitudes	A significant negative relationship was identified between optimism and seclusion attitudes. Staff attitudes influenced the likelihood of secluding patients. Staff with higher levels of therapeutic optimism and lower levels of emotional exhaustion are less likely to support the use of seclusion.	Fair

For peer review only

Haines, et al. (2017) UK	Cross-sectional/Quantitative	NHS psychiatric wards (n=60)	Staff (n=191). 50% female. Modal age group 25-34 (27%)	To identify the factors affecting perceived safety of staff on mental health inpatient wards	The factors that were found to increase perceptions of staff safety were: ward brightness, more patient beds, lower staff to patient ratios, less dayroom space and more urban views	Good
Higgins, et al. (2018) Australia	Interviews/Qualitative	Acute mental health wards (n=3)	Registered nurses (n=15). Age range 23-59	To explore the views of nursing staff regarding the factors that impact their ability to implement Safewards in acuteadult inpatient wards	Four themes arose: mixed views of Safewards; support from senior staff; understanding practice is just as important as individual skills training; and project teams need to recognise and acknowledge the local culture if it is to be changed	Fair
Ireland, et al. (2019) UK	Questionnaire/Quantitativ e	Male high secure forensic psychiatric facility	Staff (n=151; 61% male). Patients (n=62; 100% male). Age range 23-59	To examine the relationship between interpersonal factors and security incidents on a psychiatric ward	Perception of fair treatment was found to be a mediating link between dominant and hostile interpersonal styles, assertive control and victimisation incidents	Good
Johnson & Delaney (2006) USA	Descriptive/ Qualitative	Psychiatric inpatient unit	Staff (n=16); 56% female and patients (n=12); 50% female; mean age 33	To describe the context and conditions under which specific interventions were used to assist patients to regain control	The overarching theme was dimensions of “keeping the unit safe" that was split and interconnected between ideology, people, space, and time.	Fair
Jones, et al. (2010) UK	Semi-structured interviews/ Qualitative	Acute psychiatric hospital	Inpatients (n=60); 40% female; mean age 43 (19-81)	To explore the experiences of service users on acute inpatient psychiatric wards regarding safety and security	The majority of patients felt safe and supported by staff and other service users during their stay in hospital. However, safety incidents such as aggression, drug misuse or bullying negatively influenced this	Fair

For peer review only

					perspective, resulting in patients feeling unsafe.	
Jonker, et al. (2008) Netherlands	Cross-sectional/ Quantitative	Wards of a mental health institution (n=6)	Nurses (n=85); 68% female	To explore nurses' perceptions of the process of managing aggression and the determinants of the use of coercive interventions	Nurses felt in control of most aggression incidents and felt that they had social support from colleagues. Despite the high prevalence of aggressive incidents, staff did not feel that it was a large problem.	Fair
Kanerva, et al. (2015) Finland	Semi-structured interviews/ Qualitative	Psychiatric inpatient unit	Nurses (n=26); mean age 39 (range 23-60)	To describe the communication components that support patient safety	Communication that supports patient safety in psychiatric inpatient care was the main theme and overarched three sub-themes of fluent information transfer, open communication culture and being active in communication.	Fair
Kanerva, Lammintakanen & Kivinen (2016). Finland	Semi-structured/ Qualitative	Psychiatric hospital (n=2)	Nurses (n=26); 61.5% female; mean age 39 years (23-60)	To explore nursing staff's views of patient safety in inpatient care	Two themes were: experiences of safety (issues related to feelings about patient safety), and implementation of safe care (practical issues related to patient safety). Medication safety was deemed particularly important, whereas seclusion, restraint and suicide were barely mentioned. More emphasis was given to the skills staff felt they need to give safe care.	Fair
Koukia, et al. (2010) Greece	Questionnaire/Quantitative	Inpatient wards (n=14) in acute psychiatric hospitals (n=3)	Mental health nurses (n=164); 51% female; mean age 36.5 [3.5]	To investigate safety measures taken by mental health nurses and identify security policies in acute mental health wards	Standardised protocols did not exist across the hospitals. There was a lack of safety measures across the wards. Banned substances and search procedures varied greatly.	Fair

For peer review only

Kuosmanen, et al. (2013) Finland	Cross-sectional/Quantitative	Forensic hospital (n=2)	Staff (n=283); 51% female	To evaluate the current patient safety culture and identify potential improvements	Nearly two-thirds rated the patient safety level as excellent or very good. Teamwork within psychiatric units received the highest score (72% positive), and non-punitive response to errors received the lowest (26% positive).	
Kuosmanen, et al. (2019) Finland	Questionnaire/Quantitative	Forensic psychiatric hospitals (n=2)	Staff at the control hospital (n=84 baseline; n=77 follow-up). 54% female baseline; 62% female follow-up. Staff at the study hospital (n=199 baseline; n=207 follow-up). 50% female baseline; 52% female follow-up	To examine how the implementation of a patient safety incident reporting system impacts upon patient safety culture within a forensic psychiatric setting	Five patient safety culture dimensions showed a significant ($p<0.05$) increase in positive response rates (supervisor/manager expectations and actions regarding patient safety; handoffs and transitions; feedback and communication about errors; non-punitive response to error), whereas none were found for the control hospital.	Good
Langan & McDonald (2008) Ireland	Descriptive/ Quantitative	Acute psychiatric inpatient unit	Staff (n=27); mean age 35.7 [9.9]. Patients (n=35); 43% female; mean age 38.7 [10.0]	To identify the prevalence of placing patients in night attire, its clinical and demographic associations, and attitudes towards it	Patients were unhappy and uncomfortable in the night attire and suggested they should be able to wear what they want. Staff had a different view and felt only voluntary patients should be allowed to have their own clothes. Staff felt patients wearing night-attire helped with security and reducing absconding and self-harm.	Fair

For peer review only

Lavelle, et al. (2017) UK	Pre-post evaluation/Mixed methods	Psychiatric triage wards (n=2)	Staff (n=53). Mental health nurses (n=36); psychiatrists (n=6); healthcare assistants (n=9); activity co-ordinators (n=2)	To explore the impact of training on the knowledge, attitudes, and confidence levels of staff towards medical deterioration in psychiatric inpatients	Knowledge (p<0.001), confidence (p<0.001) and attitudes (p<0.02) towards managing medical deterioration significantly improved. Focus group themes were: improved confidence, understanding of effective communication, self-reflection, team working and sense of responsibility	Fair
Maguire, Daffern & Martin (2014) Australia	Semi-structured interviews/ Qualitative	Forensic hospital	Patients (n=12) Nurses (n=12); 58% female	To explore patient and staff perspectives of limit setting in a forensic hospital	Patients were unfamiliar with limit setting as a term and understood it as setting boundaries for behaviour. Staff didn't like the term as they perceived it to mean controlling the patients. Both suggested it was necessary to ensure safety. Overall, a therapeutic relationship and a consistent, empathetic, authoritative, and knowledgeable approach to limit setting was deemed important.	Fair
Mahoney, et al. (2012) USA	Pre-post questionnaire design/ Quantitative	Psychiatric Hospital	Staff: pre-intervention (n=108), post-intervention (n=143)	To explore how a programme aiming to improve team work and patient safety can be used effectively by describing the implementation process and identifying any team differences before and after implementation	The programme was implemented with success. The team attributes positively differed post-intervention (p<.01). This included team foundation, functioning, performance, skills and climate and atmosphere.	Poor

For peer review only

Martin & Daffern (2006) Australia	Questionnaire design/ Quantitative	Forensic psychiatric inpatient hospital	Staff (n=69); 46% female	To examine staff perceptions of personal safety and confidence	Clinicians reported feeling safe and confident in managing a violent incident. However, staff reported factors that affected their ability to feel confident including: knowledge, experience and skill; working as a team; and their use of intervention strategies.	Fair
Mezey, Hassell & Bartlett (2005) UK	Semi-structured interviews/ Qualitative	Single-sex and mixed-sex medium secure units (n=16)	Inpatients (n=31); 19% female. Staff (n=58); 53% female	To assess the extent to which women inpatients consider themselves to be safe and to whether these views are identified in staff	Patients in both types of unit experienced violence, both sexual and physical. However, women patients in the single-sex units experienced more intimidation, threats and physical abuse but less sexual and physical violence.	Fair
Millar & Sands (2013) Australia	Exploratory descriptive design/ Qualitative	Acute inpatient psychiatric unit	Inpatients (n=25) covering 500 handovers	To identify the frequency and type of risk information transferred at handover	Patient safety is compromised by the reduced or inadequate communication over risk. Verbal communication was the most common method for handover updates.	Good
Nathan, et al. (2007) UK	Prospective/ Quantitative	A medium secure forensic psychiatric hospital	Nurses on the women's ward that were followed-up (n=14); 71% female; mean age 38.1 (SD=10.00). Nurses on the men's ward that were followed-up (n=14); 93% male; mean age 37.7 (SD=9.8)	To explore gender differences in psychopathology in forensic mental health settings and how this may affect the risk of staff burnout	Nurses on the women's ward were found to score significantly higher in the emotional exhaustion component of burnout. Suggested inter-related factors that are key to the development of emotional exhaustion include: nature of the mental disorder may differ between genders, and men can display more aggressive behaviour whilst women display more relational aggression.	Fair

For peer review only

O'Brien & Cole (2004) Australia	Participatory action research/ Qualitative	Acute inpatient general hospital psychiatric facility	Patients, relatives, carers and nurses (n=42)	To explore the context and experiences of nurses, patients, and relatives in the close-observation area and to develop recommendations for clinical practice	Data revealed three main themes: design and environment (concern was raised about the lack of privacy and security), lack of activity and structured time, and nursing care (some patients indicated that they did not feel cared about).	Fair
O'Neill, et al. (2003) Ireland	Structured interviews/ Mixed methods	A psychiatric hospital	Inpatients. Length of Stay < 2 years (n=45); median age 29.3) Length of Stay > 2 years (n=43); Medium age 48.8, and key workers.	To describe the patient groups present in Irish forensic psychiatric beds and to ascertain unmet treatment and placement needs	Twenty (47%) of the long-stay group and eleven (24%) of those with shorter durations of stay were felt to be inappropriately placed. 30% of long-stay patients could be safely transferred to lower levels of security within six months and 63% within three years.	Fair
Pelto-Piri, et al. (2019) Sweden	Interviews/Qualitative	General psychiatric clinic (n=1); psychiatric addiction clinic (n=1); forensic psychiatric clinic (n=2)	Adult patients (n=17)	To explore patients' perceptions of feeling safe or unsafe in psychiatric inpatient care	Interview themes were: predictable and supportive services increase feelings of safety; communication and responsibility increase feelings of safety; powerlessness and unpleasant encounters reduce feelings of safety	
Qi, et al. (2014) China	Cross-sectional/ Quantitative	Psychiatric hospitals (n=2)	Nurses (n=705); 100% female; mean age 31.3 (SD=7.1)	To compare the level of work-related stress between female nurses working in psychiatric and general hospitals and explore associated individual and environmental factors	Psychiatric nurses were more likely to be exposed to workplace violence (23.5% vs. 80.5%, p < .001), and had greater levels of stress in the domains of working environment (p < .001) and patient care (accidents, patient-staff relationships, and the impact of patient suffering or death) (p < .001).	Good

For peer review only

Rees & Manthorpe (2010) UK	Semi-structured interviews/ Qualitative	Residential services (n=8)	Managers (n=13) and care workers (n=10).	To explore the impact of adult protection investigations on managers of residential services and staff	Service managers stated that the application of policy and practice can be both beneficial and detrimental to their services (including service disruption, stress for residents, staff and managers). Multi-agency collaboration, transparency, training, reflective practice, and effective supervision were given as influencing the implementation of adult protection policies.	Fair
Ryan (2007) Europe	Longitudinal/ Quantitative	Acute inpatient psychiatric centres	Staff (n=205)	To compare, across six European mental health services, the levels of occupational stress and burnout among mental health workers in acute psychiatric hospitals	There were no statistically significant differences between sites and teams regarding emotional exhaustion. Great Britain had the highest score for depersonalisation.	Fair
Salzmann-Erikson, et al. (2008) Sweden	Cross-sectional/ Qualitative	A PICU	Caregivers (n=18); 50% female; age range 23-56.	To describe which care activities are practiced by registered nurses and assistant nurses in the PICU.	Categories describing the fundamentals of the PICU were found: protests and refusal of treatment due to lack of capacity, escalating behaviours, and using coercive measures to manage violence.	Fair
Silvana, et al. (2012) Italy	Cross-sectional/ Qualitative	A psychiatric ward	Nurses (n=20)	To plan ergonomic improvement from preliminary results of a psychiatric ward case-study	Ergonomic issues were found: a locked and polluted environment (smoking indoors) and an unusual staff gender ratio (hospital management considers staff gender ratio a safety issue due to men's strength, allowing them to cope with aggressive behaviour). No mental health care programmes are available for nursing staff, not even after	Fair

For peer review only

					safety incidents.	
Sjöstrand, et al. (2015) Sweden	Cross-sectional/ Qualitative	General psychiatry, forensic psychiatry, and addiction psychiatry settings	Psychiatrists (n=8); 50% male; age range 30-68.	To explore the psychiatrists' ethical reasoning regarding involuntary psychiatric treatment.	Participants were focused on the consequences of involuntary treatment, balancing risk of damaging the therapeutic alliance against ensuring patients received the treatment they needed. Suicidal and psychotic patients were examples where involuntary care was seen as justified. However, it was also argued that risk of suicide might not be sufficient for justified involuntary care. Organisational factors were seen as sometimes resulting in compulsory treatment that could have been avoided.	Fair

For peer review only

Stead, et al. (2009) Australia	Pre-post intervention/ Quantitative	An inpatient mental health facility	Multidisciplinary staff	To evaluate the implementation of a staff educational program aimed at increasing team work and patient safety, addressing three outcomes: observed team behaviours; attitudes and opinions; and clinical performance and outcomes	Changes implemented included the restructuring of multidisciplinary meetings and the introduction of structured communication tools. There was also a significant improvement in aspects of patient safety culture (frequency of event reporting, and organisational learning) and knowledge, skills and attitudes scores increased by 6.8%. Seclusion rates also reduced.	Fair
Stein (2002) UK	Cross-sectional/ Qualitative	Mental health inpatient facilities	Clinical directors (n=25), medical directors, (n=22), managers (n=6), consultants (n=3), nurses (n=1), other (n=1)	To explore the development and effective use of tools to predict risk to patients and public following discharge into the community	60.3% of sites used a discharge checklist, so development of risk assessment tools is varied at Trust level. Developments seem to be stimulated by clinical governance in the absence of an overarching national strategy. It seems that information technology for risk data collection is not widely used, instead using paper and informal communication.	Fair
Stübner, Groß & Nedopil (2006) Germany	Cross-sectional/ Quantitative	Forensic institutions (n=8)	Heads of department or their locum tenens	To investigate risk factors for incidents during hospitalization and criteria allowing easing of regulations and confinement	Among the risk factors, 87% of the terms related to patient characteristics, while among the protective factors the proportion was 77.5%. Good relations with the therapeutic team accounted for 14.6% of the criteria for easing restrictions, while poor relations accounted for less than 4% of the risk factors.	Fair

For peer review only

Truea, et al. (2017) USA	Interviews/Qualitative	7 Veteran's Health Administration inpatient psychiatric units	Staff (n=20) working within 7 VHA hospitals including psychiatrists (n=7) and other management/staff (N=13)	To identify risk factors and protective factors, along with the mechanisms by which they relate to patient safety events in this setting in order to inform interventions geared toward improving quality of care for persons with serious mental illness	Two broad thematic domains related to patient safety: risks – threats to patient safety events at the system-, provider-, and patient-level; and protective factors associated with psychiatric inpatient safety—processes and infrastructure in the treatment environment that, when in place, thwart or mitigate these risks.	Good
Vahidi, et al. (2018) Iran	Interviews/Qualitative	Psychiatric referral center. The center is comprised of separate male and female inpatient units that have similar conditions and regulations.	Patients at the point of discharge (n=7); Staff (n=19)	To generate more understanding of the nature of therapeutic relationships in Iranian psychiatric inpatient settings and how these relationships may be enhanced to improve quality and safety of care.	Facilitators of a safe environment included “supportive relationship with patients” and “improving patient capacity for self-efficacy/self-control”. Inhibitors of a safe environment included “detachment from patients” and “domination over patients”.	Good
Vandewalle, et al. (2018) Belgium	Questionnnaires/Quantitative	173 psychiatric wards within 37 hospitals in Belgium	705 nurses	To investigate the demographic and contextual factors that influence the willingness of nurses on psychiatric wards to share power and responsibility with patients concerning patient safety.	The willingness of psychiatric nurses to accept a new role is positively associated with being male, older, employed on an open ward, and perceiving personal competence and support to facilitate patient participation. The ‘acceptance of a new role’ component does not include items about specific patient safety situations and conditions, such as aggression, self-harm, or suicidal ideation. The receptivity of nurses to patients’	Good

For peer review only

					factual and challenging questions is negatively associated with nurses' willingness to accept a new role.	
Vlayen, et al. (2012) Belgium	Cross-sectional / Quantitative	Acute (n=90), psychiatric (n=42) and long-term (n=11) care hospitals	Staff in acute (n=47,635), psychiatric (n=6341) and long-term care hospitals (n=1249)	To explore patient safety culture in Belgian hospitals and the underlying safety culture dimensions	Strengths of patient safety culture were: team work, supervisor/ manager expectations and actions promoting safety, and organisational learning. Handoffs and transitions, staffing, management support for patient safety, non-punitive response to error and teamwork across units could be improved. Positive dimension scores were higher for psychiatric and long-term care hospitals than for acute hospitals, suggesting that patient safety is more encouraged within these settings.	Good
Ward (2013) Australia	Cross-sectional / Qualitative	An inpatient mental health care facility	Nurses (n=13); 100% female	To investigate nursing practices, the nurse-patient relationship, violence, and aggression in acute inpatient mental health care	Some workplace stressors included poor staffing skill matrix, complex patient diagnoses, and limited workspace design. Effective communication was seen as essential to prevent violence. Coping mechanisms included debriefing with other colleagues.	Good

For peer review only

Wood & Pistrang (2004) UK	Cross-sectional / Qualitative	An acute inpatient psychiatric unit	Patients (n=9); nursing staff (n=7)	To explore the experiences of safety and threat from the perspective of psychiatric inpatients	Ten themes relating to factors that influenced feelings safety were grouped into three clusters—patient interactions, staff behaviour and attitudes, and non-consensual treatment. Patients expressed feeling vulnerable and helpless.	Fair
Woods (2013) Canada	Cross-sectional / Qualitative	Inpatient mental health units (n=7) and a forensic unit	Registered Psychiatric Nurses (n=33); Registered Nurses (n=2); Licensed Practical Nurse (n=1); Special Care Aides (n=7); Social Workers (n=2); Student Nurse (n=1); and other (n=2). Total (n=48)	To identify and describe current risk assessment and management approaches used in adult inpatient mental health and forensic units	Key issues were discussed as important: the pitfalls of relying on clinical judgement alone; considering risk as a wider concept; risk management being reactive; lack of education and training, and client involvement.	Fair

For peer review only

Harm to self

Author, year, country	Study design	Setting	Participants	Aims/ objectives related to patient safety	Outcomes related to patient safety	Study quality
Awenat, et al. (2018) England	Interviews/Qualitative	NHS mental health service in Northern England with five acute adult psychiatric wards	Patients in five acute adult psychiatric wards (n=20), aged 22-65	To investigate suicidal in-patients' views and expectations of a novel ward-based suicide-focussed psychological therapy intervention	Two themes: Theme 1 - 'A therapy that works', epitomised participants' perceptions of the influences and necessary components for effective ward-based suicide-focused psychological therapy. Theme 2 - 'Concerns about in-patient suicide-focused therapy' depicted participants' fears about engaging with therapy.	Good

For peer review only

Booth, et al. (2014) Ireland	Questionnaire/Quantitative	A psychiatric hospital	Patients (n=114). 81% female. Mean age 35	To evaluate the effectiveness of a brief intervention for decreasing self-harm and increasing distress tolerance.	There was a decrease in the frequency of self-harm incidents from pre-intervention (M=13.68, SD=21.81) to post-intervention (M=4.50, SD=11.01) (p=.02, N=48) and from pre-intervention to 3 months post-intervention (M=3.62, SD=11.33) (p=.01, N=48). There was an increase in distress tolerance from pre-intervention (M=6.32, SD=2.35) to post-intervention (M=10.36, SD=3.68) (p=.00, N=32) and from pre-intervention to 3 months post-intervention (M=9.72, SD=4.33) (p=.00, N=32). There was a decrease in inpatient days from pre-intervention (M=39.90, SD=33.25) to post-intervention (M=23.09, SD=40.56) (p=0.01, N=65), from pre-intervention to 3 months post-intervention (M=8.78, SD=25.40) (p=.00, N=65) and from post-intervention to 3 months post-intervention (p=.00, N=65).	Fair
Bowers, et al. (2008) UK	Questionnaire/ Quantitative	Acute psychiatric wards (n=136) in 67 hospitals within 26 National Health Service (NHS) Trusts	Staff and patients within the units	To explore the relationship between special observation and self-harm rates in acute psychiatric wards	Constant special observation was not associated with self-harm, but intermittent observation (OR=0.82 (0.78–0.87); p<0.001), levels of qualified nursing staff (OR=0.94 (0.90–0.98); p<0.01), and more intense programmes of patient activities (OR=0.53 (0.38–0.75); p<0.001) were associated with reduced self-harm.	Good

For peer review only

Brown & Beail (2009) UK	Semi-structured interviews/ Qualitative	A secure service for people with intellectual disabilities and challenging behaviour	Residents (n=9). 56% male.	To explore the experiences of self-harm, and interventions for this self-harm, among residents with intellectual disabilities within secure accommodation	Self-harm management interventions imposed by the service were mostly perceived as controlling, punitive and evoked strong negative reactions from residents.	Good
Caspi (2014) USA	Observation and semi-structured interviews/ Qualitative	Special dementia care units within an assisted living residence (n=2)	Residents (n=12). 92% female. Mean age 81 (range 75-86).	To explore self-neglect and other behaviours when residents with dementia are unsupervised within assisted living residences	There were 158 incidents of negative behaviour expression, emotional states and self-neglect. 56% of these were self-neglect incidents. 97% of these took place during times when residents were not engaged in structured activity. The incidents included a wide variety of safety risks, including falls, attempted absconding and risk of aggressive interactions.	Good
Cleary, et al. (1999) Australia	Interviews/ Qualitative	Acute inpatient psychiatric wards (n=4) within a psychiatric hospital	Clinical registered nurses (n=10)	To explore the role of registered nurse in the care of patients on special observation	9 themes were developed to highlight the topics discussed by nurses when considering their role in the special observation process: safety; therapeutic relationships; supporting patients and carers; consequences of special observation for nurses; continuity of care concerns; peer support; suicide indicators; responsibilities and rights; and nurses, doctors and the hospital hierarchy.	Fair

For peer review only

Davis, Williams & Hays (2002) USA	Questionnaire/ Quantitative	An acute-care psychiatric facility	Patients (n=135). Mean age 36. 70% female	To explore the views of suicidal psychiatric inpatients concerning no-suicide agreements	Patients had an overall positive view of no-suicide agreements, relating to the therapeutic features, coerciveness and detachment of the process. 92% of participants agreed that they had confidence in their ability to keep to their commitment.	Fair
de Jonghe-Rouleau, Pot & de Jonghe (2005) Netherlands	Questionnaire/ Quantitative	Psycho-geriatric wards of a large nursing home (n=3)	Residents (n=110). 77% female. Mean age 83 (range 67–105)	To explore self-injurious behaviour (SIB) in nursing home residents with dementia	SIB was reported in 22% of patients. Pinching, scratching and banging one's fist against objects were the most commonly reported behaviours (8% of patients). SIB was associated with: prescribed psychotropics (OR=5.62), immobility (OR=3.71) and restraint (OR=6.19).	Fair
Drew (1999) USA	Questionnaire/ Quantitative	Psychiatric hospitals (n=84)	Staff	To explore the use of no-suicide contracts in psychiatric hospitals	The most common suicide prevention intervention used by the hospitals was limiting access to objects used for self-harm (100%). 79% of the hospitals used no-suicide contracts. Contracts were mostly drawn up after suicidal ideation was expressed (83% of hospitals that used no-suicide contracts) by nurses. They were mostly verbal (74%). 53% of respondents estimated suicide behaviour frequency of 10+ incidents a year.	Fair
Ellis, et al. (2012) USA	Open-trial case-series/ Quantitative	A private psychiatric hospital	Patients (n=20). 80% female. Mean age 37 (range 21-55)	To evaluate a program designed to reduce the risk of suicide in inpatient psychiatric hospitals	From pre-post treatment, there were significant reductions (effect sizes= > .80) in depression, hopelessness, suicide cognitions, and suicidal ideation, as well as improvement on contributory factors of	Fair

For peer review only

					suicidality. The factors with the highest effect sizes were: depressive symptoms, psychological pain, and self-hate.	
Ellis, et al. (2015) USA	Controlled trial/ Quantitative	A private psychiatric hospital	Patients (n=52). 69% female. Mean age 33 (range 18-68)	To evaluate the effectiveness of a program designed to reduce the risk of suicide in inpatient psychiatric hospitals above and beyond that of intensive, psychotherapeutic, milieu-based treatment	The program group showed greater improvement on measures of suicidal ideation and suicidal cognition (effect sizes= > .80).	Fair
Esposito-Smythers, McClung & Fairlie (2006) USA	Descriptive/ Quantitative	An acute adolescent psychiatric inpatient unit	Patients (n=250). 60% female. Mean age 15 (range 12-18)	To explore participant perceptions of a suicide prevention group for psychiatrically hospitalised patients	94% of participants reported that they learnt something from the group that would prevent them attempting suicide in the future. The most helpful part of the group was reported to be the Reasons to Live List (32% of participants stated this was most helpful). The least helpful part was reported to be the Safety List (30%).	Fair
Gibson, et al. (2014) Ireland	Controlled trial/ Quantitative	Psychiatric hospital	Psychiatric inpatients (n=103). (range 18-60); Living through distress programme (LTD), (n=82); 79% female; mean age 38 and waiting list (TAU) (n=21); 57% female; mean age 32	To examine whether additional DBT skills training improved outcomes including self-harm compared to TAU	Self-harm significantly reduced in the LTD group compared with TAU (49% of the LTD group had a reduction in self-harm of 75% or greater) and this was maintained at 3 month follow-up.	Fair
Gough & Hawkins (2000) UK	Questionnaire/ Quantitative	Forensic psychiatric hospital	Clinical staff (n=77)	To explore staff attitudes regarding self-harm in psychiatric patients	Staff attitudes towards self-harm varied. Staff felt self-harm was used to communicate with others about how distressed they are (M=2.9; SD = 0.8). Some staff felt self-harm wasted staff time (M=1.9;	Poor

For peer review only

					SD= 1.0). Overall, staff felt more training on self-harm was needed.	
Hill, et al. (2017) USA	Questionnaires and interview/ Quantitative	Acute-care psychiatric hospital	Adolescent psychiatric inpatients (n=142). 59.9% female. Mean age 14.73 (range 12–17)	To better understand the role of interrupted and aborted attempts in suicide risk assessment.	Results suggest that interrupted and aborted suicide attempts are associated with the frequency of actual suicide attempts, controlling for suicidal ideation and depressive symptoms.	Fair
Holth, et al. (2018) Norway	Questionnaire/Mixed methods	Inpatient wards within mental health centres (n=32) and psychiatric hospitals (n=29)	Department managers (n=61)	To assess extensive psychiatric hospitalisation due to self-harm, associated severe medical sequelae and health service collaboration issues	There were 427 cases of extensive hospitalisation due to self-harm. In 109 cases, there were severe medical consequences, including five deaths. Collaboration issues were recorded in 122 cases (including diagnosis, treatment and resource disagreements).	Fair
Inoue, et al. (2017) Japan	Questionnaire/Quantitative	Psychiatric settings within hospitals (n=97)	Staff	To investigate inpatient suicides and risk factors in psychiatric settings	131 inpatient suicides were recorded in inpatient settings. Hanging was the most common suicide method (58% of suicides) and most happened on the ward (47%) between 8.00 and 16.00 (43%). The most comon risk factor was previous suicide attempt or self-harm (46%). The most common psychiatric diagnosis was schizophrenia (39%).	Fair

For peer review only

James, et al. (2017) UK	Survey and interview/ Mixed methods	Acute psychiatric wards (n=31)	Phase I: Inpatient mental health practitioners (n=387). 57% female. Age range 20-60 or over. Phase II: Inpatient mental health practitioners (n=18). 72% female. Age range 20-49.	To explore nursing practitioners' perspectives and experiences of harm reduction practices for self-harm on mental health wards.	Practitioners who had implemented the approach reported positive outcomes including a reduction in incidence and severity of self-harm and a perceived increase in empowerment of service users. Practitioners with no experience of using harm reduction were concerned that self-harm would increase in severity, and were unsure how to assess and manage risk in people under a harm reduction care plan. Some fundamentally disagreed with the principle of harm reduction for self-harm because it challenged their core beliefs about the morality of self-harm, or the ethical and potential legal ramifications of allowing individuals to harm themselves.	Good
Kool, et al. (2014) Netherlands	Quasi-experimental pre-post-test/ Quantitative	Mental health centres (n=8) and a forensic-psychiatric centre (n=1)	Staff (n=178); mean age 38	To measure the effects of a self-harm reduction programme on attitudes towards self-harm patients; self-efficacy in managing self-harm patients; and the distancing of self-harm patients and treatment staff	After the programme, staff were significantly more likely to have perceived confidence in assessing and referring self-harm patients; managing them effectively; have an emphatic approach; and be able to cope effectively with regulation in relation to self-harm guidance.	Fair

For peer review only

Lindgren, Aminoff & Graneheim (2015) Sweden	Participant observational and interview study/ Qualitative	Psychiatric hospital	Women inpatients (n=6); median age 23.5 (range 21-37 years)	To describe features of everyday life of patients who self-harm	The main factor associated with everyday life was being surrounded by the disorder. This encompassed residing in a confusing environment, being confined to routines and rules around safety that lack consistency. Loneliness was an issue. Staff spent limited time with the patients.	Fair
Lundegaard Mattson & Binder (2012) Norway	Cross-sectional/ Qualitative	A psychiatric hospital	Healthcare workers (n=8)	To explore the perceptions, emotions and actions of healthcare staff when working with patients who self-harm	Several themes emerged, detailing: the frustration at having to use coercive interventions; the process of change from coercion to alliance; experiences of useful management strategies; and the distinction between self-harm and suicide attempt. On a structural level, a cap was put on the number of patients diagnosed with Emotionally Unstable Personality Disorder, and staff felt that this made it easier to work with patients, reducing frequency of self-injury. Management also became more involved in supporting staff training to reduce use of coercion.	Good

For peer review only

O'Donovan (2007) Ireland	Semi-structured interviews/ Qualitative	Acute psychiatric inpatient units (n=2)	Psychiatric nurses (n=8); 75% female (age range 25-55)	To explore the practices of psychiatric nurses relating to people who self-harm, but are not suicidal	Participants emphasized preventing self-harm and providing a physically safe environment. The primary methods for ensuring a service user's safety were: removal of sharp objects; and requesting that service users stay in their night clothes. Participants reported spending 15 to 90 min a day with each service user in therapeutic interaction. This was then used to inform staff of the potential for self-harm, to assess mood, and to inform future care.	Fair
Pfeiffer, et al. (2019) USA	Questionnaire/Quantitativ e	Psychiatry units of two midwestern facilities.	70 adult psychiatric inpatients	To assess the acceptability, feasibility, and fidelity of a peer specialist intervention titled Peers for Valued Living (PREVAIL) to reduce suicide risk, incorporating components of motivational interviewing and psychotherapies targeting suicide risk into recovery-based peer support.	Those in the PREVAIL arm completed an average of 6.1 (SD 5.0) peer sessions over the course of 12 weeks. Fidelity was rated for 20 peer support sessions, and 85% of the peer specialist sessions demonstrated adequate fidelity to administering a conversation tool regarding hope, belongingness, or safety, and 72.5% of general support skills (e.g., validation) were performed with adequate fidelity. Participants' qualitative responses (n 23) were highly positive regarding peer specialists' ability to relate, listen, and advise and to provide support specifically during discussions about suicide.	Fair

For peer review only

Sansone, McLean & Wiederman (2008) USA	Cross-sectional/ Quantitative	A community hospital	Inpatients (N=120). 61% female; mean age 38.69, SD=11.74 (range 18-74)	To explore, in a sample of psychiatric inpatients, the relationship between self-sabotaging behaviours and borderline personality disorder	76 respondents (63.3%) reported engaging in medically self-sabotaging behaviour, with the average number of different behaviours of this type being 4.11 (SD = 3.93). The most commonly endorsed behaviours (endorsed by around a quarter of participants) were: damaging self on purpose and seeking medical treatment; not going for medical treatment; to purposefully hurt self; not taking a prescribed medication; and involvement in dangerous situations.	Fair
Sandy (2016) United Kingdom	Interviews/Qualitative	Learning disability service in the west of England. Service comprises of 7 locked clinical areas with six registered nurses working in each area	Registered nurses (n=35)	To explore nurses' knowledge and understanding of the use of observation on patients who self-harm in a learning disability service in the United Kingdom.	Three superordinate themes 1) observation: its meaning, 2) observation: does it prevent self-harm? 3) Observation: making it work.	Good

For peer review only

Shaw & Sandy (2016) UK	Mixed methods/Qualitative	A large forensic mental health unit, containing medium and low secure facilities.	Mental health nurses (n=61), aged 25-56	To report the attitudes of nurses toward user who self-harm in secure environments, and propose educational recommendations	One super-ordinate theme (attitudes to self-harm), two second order themes had several subthemes. Positive attitdues included: need for training, understanding of self-harm, unconditional acceptabce, partnership working optimism and provision of choice activities. Negative attitudes included rigid authorative apporach, refusal to undertake training, blanket approach and insensitive responses.	Good
Sjöström, Hetta & Waern (2012) Sweden	Prospective cross-sectional/ Quantitative	Psychiatric units	Inpatients who attended follow-up interview (n=98)	To investigate whether low Sense of Coherence (SOC) is a predictor of suicidality and of risk of a repeat suicide attempt	2 months after a suicide attempt, low baseline SOC was significantly associated with an eightfold increase in risk of high suicidality (*P* = <0.001). Low SOC score at baseline was also associated with repeat attempts within 3 years (*P* = 0.038), but was not an independent predictor when other mental health symptoms were taken into account.	Good
Sun, et al. (2005) Taiwan	Cross-sectional/ Qualitative	Psychiatric admission wards	Patients (n=15); 60% female; age range 16-47. Psychiatric nurses (n=15); 100% female; age range 21-49.	To develop a suicide care theory that could help nurses to improve suicide prevention and care	In developing the theory, four categories were highlighted relating to interaction strategies: holistic assessment of people who are suicidal; protection; basic care; and advanced care.	Fair

For peer review only

Sun, et al. (2006) Taiwan	Cross-sectional/ Qualitative	Acute psychiatric wards and a psychiatric stress ward	Patients (n=15); 60% female; age range 16-47. Psychiatric nurses (n=15); 100% female; age range 21-49.	To: (1) explore and examine psychiatric nurses' and patients' perceptions of the care offered to patients with suicidal ideations on psychiatric wards, and (2) develop a nursing theory to guide the care of patients at risk of suicide	Fifteen categories were generated, relating to contexts and intervening conditions of care for suicidal patients. These categories centred on the therapeutic relationship, staff attitudes, blame, autonomy, staff powerlessness due to lack of training, and time constraints.	Fair
Swogger, Van Orden & Conner (2014) USA	Cross-sectional/ Quantitative	Acute inpatient hospitals (n=3)	Patients (n=892); 58% male; mean age 29.9 (SD=6.2) age range 18-40	To study the relationship of outwardly-directed aggression to suicidal ideation and attempts in psychiatric inpatients	Outwardly-directed aggression was associated with suicide attempts, but not ideation. It was also associated with planned, but not unplanned, suicide attempts.	Fair
Takahashi, et al. (2011) Japan	Cross-sectional/ Quantitative	Psychiatric medical institutions (n=8)	Nurses (n=531); 63% female; mean age 41.9 (SD=12.3)	To investigate issues related to staff experiences of patient suicide in mental health nursing	55% of participants had experienced patient suicide. 13.7% of participants were at a high risk of post-traumatic stress disorder. However, only 15.8% stated that they had access to post-suicide mental health care programmes. There was also low attendance at in-hospital seminars on suicide prevention or mental health care for nurses (26.4% and 12.8% respectively).	Fair

For peer review only

Thomas & Haslam (2017) UK	Interviews/Qualitative	Mental health inpatient settings	Staff (n=10). 80% female. Age range 23-31	To explore the experience of mental health staff of self-harm prevention and distress management	Interview themes were: characteristics of the inpatient environment, experiences of distress and urges to self-harm in the inpatient environment, when the inpatient environment does not provide alternative means of managing distress, ways the inpatient environment does provide alternative means of managing distress, and factors that influnece patient responses to inpatient environment and interventions	Fair
Tofthagen, Talseth & Fagerström (2014) Norway	Cross-sectional/ Qualitative	Psychiatric clinics (n=4)	Mental health nurses (n=15); 87% female	To explore the experiences of mental health nurses in caring for inpatients who self-harm	Two main categories were identified: challenging and collaborative nurse-patient relationships, and promoting well-being through nursing interventions. Participants sought to understand the self-harm behaviour, its triggers and signs in a person-centred way, helping patients learn to be aware of their feelings/behaviours and learn coping strategies.	Fair

For peer review only

Vandewalle, et al. (2019) Belgium	Interviews/ Qualititative	Closed ward in psychiatric hospital	Nurses (n=5). Age range 25- >55.	To uncover and understand the actions and aims of nurses in psychiatric hospitals during their interactions with patients experiencing suicidal ideation.	The findings show that nurses' actions and aims in their interactions with patients experiencing suicidal ideation are captured in the core element 'promoting and preserving safety and a life-oriented perspective'. This core element represents the three interconnected elements 'managing the risk of suicide', 'guiding patients away from suicidal ideation', and 'searching for balance in the minefield'.	Good
Vråle & Steen (2005) Norway	Cross-sectional/ Qualitative	Acute psychiatric wards	Nurses (n=5); 60% female	To explore the dynamics of performing constant observation of suicidal patients	The process of constant observation seems to follow organizing phases, from assessment to transitioning out of the process. The process requires a balance between the need for control and keeping the patient from self-harm, structure and flexibility, as well as maintaining a therapeutic relationship.	Fair
Weber (2002) USA	Cross-sectional/ Qualitative	A locked psychiatric hospital	Patients (n=9); 100% female	To explore how self-abusing women in a state psychiatric hospital defined self-abuse	Four themes were identified: the need to be listened to and receive help; specific triggers for self-abuse (noise on the ward, especially screaming and profanity); the causes of self-abuse; and how to stop the self-abuse (someone talking to them during times of crisis, and staff providing	Fair

For peer review only

comfort or distraction).

For peer review only

Safety of the Physical Environment

Author, year, country	Study design	Setting	Participants	Aims/ objectives related to patient safety	Outcomes related to patient safety	Study quality
Bayramzadeh (2016) USA	Focus groups/Qualitative (retrospecitve audit not reported here)	Institute for mental health in the southeast region of the USA. 81-beds patients admitted for substance and alcohol abuse, adult psychiatry, child and adolescent patients, and geriatric patients	Staff (n=9)	To explore if incident reports and staff perceptions confirm the differences in safety levels among different areas in the facility and if specific types of safety incidents occur more frequently in specific areas of the facility?	Participatns indicated that locations designated as Safety Level 4 (patient bedrooms and bathrooms) were the most problematic, followed by locations designated as Safety Level 3 (lounges and activity rooms).	Poor
Bellantonio, et al. (2008) USA	Randomized controlled trial/ Quantitative	Dementia-specific assisted living facilities (n=2)	Residents of the facilities with dementia (control group, n=52; intervention group, n=48)	To investigate the effects of a multidisciplinary team intervention on unexpected transitions from assisted living in people with dementia	Falls were the most frequent reason for unanticipated transition (n=40). The intervention reduced the risk of unanticipated transitions by 13%, but this was not statistically significant (p=0.67). The intervention reduced the risk of death by 63%, but this was not statistically significant (p=0.08).	Good
Bowers & Crowder (2012) UK	Questionnaire/ Quantitative	Acute psychiatric wards (n=32)	All staff and patients within the units	To explore the relationship between staffing numbers and adverse incidents on the wards of psychiatric hospitals	Lower qualified nurse staffing levels were associated with higher conflict (Incident Rate Ratio (IRR) =1.03)) and containment (IRR=1.03) rates.	Good
Bowers, et al. (2010) UK	Questionnaire/ Quantitative	Acute psychiatric wards (n=136) in 67 hospitals within 26 National Health Service (NHS) Trusts	Staff (n=638; 62% female; modal age group 25-34), patients (n=393; 52% female; modal age group 35-44) and visitors (n=168; 50% female; modal age group 35-44) within the units	To explore the acceptability of door locking to staff, patients and visitors within acute psychiatric wards	Five factors were found to relate to the acceptability of door locking: adverse effects, staff benefits, patient safety benefits, patient comforts and cold milieu. Patients felt more negatively about door locking than staff.	Fair

For peer review only

Chandler (2008) USA	Interviews/ Qualitative	An inpatient psychiatric unit within a community hospital	Staff (n=10)	To explore the experience of staff when moving from traditional care to trauma-informed care within inpatient psychiatric units	The transition was described by staff in the context of creating a culture of safety. The overarching theme was of transferring control from staff to patients, and this included: changed perspectives, collaborative patient–staff relationships, the implementation of safety protocols, and the prescription of individualized evidence-based educational resources.	Fair
Cowman & Bowers (2008) UK & Ireland	Comparative questionnaire/ Quantitative	Acute psychiatric wards in England (n=87) and acute admission psychiatric wards in Ireland (n=37)	Staff	To explore safety and security measures in acute psychiatric wards in England and Ireland	Irish wards were found to have higher levels of security and safety restrictions. Irish wards are more likely to ban items, restrict access to areas/items, routinely search patients, have access to security guards at all times, and have staff carry personal alarms. Door locking procedures were similar, with 29% of English wards and 28% of Irish wards always keeping doors locked.	Fair

For peer review only

Curtis, et al. (2013) UK	Unstructured interviews/ Qualitative	An inpatient mental health care facility	Staff (Phase 1 n=23; Phase 2 n=8; Phase 3 n=26)	To explore the views of staff concerning technical safety and therapy in the design of an inpatient mental health facility	Participants mentioned how responsibility for technical safety was being invested in the physical infrastructure. Surveillance and observation were also important. Staff felt that relying on technical safety measures meant shirking responsibility for risks they should manage. They had concerns about how focusing on technical safety might conflict with a therapeutic approach.	Fair
Dreyfus, Phillipson & Fleming (2018) Australia	Focus groups/Qualitative	Aged care facilites for individuals with dementia	Staff (n=25) and family members (n=6)	To explore attitudes of staff and family members to fences in dementia care settings	Focus group themes were: fences as a necessary evil, tension between physical and emotional safety, pressure on staff to keep residents safe, fences as a threat to physical safety, the positive impact of fences on emotional safety and wellbeing. There were also ideas for mitigating the negative effects of fences on residents' wellbeing	Fair

For peer review only

Fletcher, et al. (2019) Australia	Facilitated forums/ Mixed methods	Acute mental health hospital wards	Patients (n=9). 56% male. Mean age 44 (range 29-62). Carers (n=9). 89% female. Mean age 61 (range 46-73). Staff (n=17). 65% female. Mean age 40 (range 23-56).	To describe the research process and findings from stakeholder facilitated forums that revised and approved a set of evidence informed recommendations, to improve the least restrictive practices of acute mental health wards within the context of a locked doors policy in Queensland, Australia.	All participants endorsed the recommendation that acute wards should take a recovery-oriented approach. This was discussed at length in every forum, with many of the recommendations in the Recovery Orientation category rated highly by participants. Reducing boredom and increasing availability of peer support workers were considered key to achieving this.	Fair
Gebhardt & Steinert (1999) Germany	Pre-post questionnaire/ Quantitative	Acute general psychiatry wards (n=4) within a psychiatric hospital	Patients (n=183). 55% female. Mean age (Time 1=38; Time 2=38; Time 3=36). Staff (n=162)	To explore the effects of an equal distribution of severely disturbed patients on social climate, aggressive behaviour, and sexual molestation in acute wards	A significant improvement of ward atmosphere (Rao's R=1.654, P=0.037) and a reduction of aggressive behaviour ($\chi 2>16$, df=2, P<0.001) was found after structural changes were implemented, whereas the impact on sexual molestation could not be determined as these events were rare at baseline.	Fair
Haglund & von Essen (2005) Sweden	Semi-structured interviews/ Qualitative	Psychiatric inpatient unit	Voluntarily admitted inpatients (n=20); median age 43 years (range 19-87)	To describe patient perspectives of advantages and disadvantages of being cared for on a unit that has locked doors	Advantages included protecting patients and staff from external factors, providing patients with security and efficient care, and providing staff with a sense of control. Disadvantages included: making patients feel confined, making patients dependent on staff, and making patients feel worse emotionally.	Poor

For peer review only

Hunt, et al. (2012) UK	Descriptive/ Quantitative	Psychiatric inpatient wards	Inpatient suicides who died by hanging between 1999-2007 (n=344); median age 39 (range 17-85); 63% male	To establish the principal ligatures and ligature points on the ward and whether there are trends over time	During 1999-2007, a quarter of inpatients who died by hanging, did so within a week of admission (25%); half had been on a high or medium observation level (49%). A third of the deaths were thought to be preventable (33%). The door, hooks, handles and windows were collectively the most common ligature points (59%). Belts, sheets or towels accounted for almost two-thirds of the ligatures (61%).	Fair
Kalagi, et al. (2018) Germany	Interviews/ Qualititative	Acute psychiatric wards	Psychiatrists (n=15). 60% female. Mean age 35.3 (range 28–54). Psychiatric nurses (n=15). 60% female. Mean age 35.2 (range 24–63). Patients (n=15). 80% male. Mean age 38.9 (range 20–60).	To assess the opinions and values of relevant stakeholders with regard to the requirements for implementing open wards in psychiatric hospitals.	The interviewees identified conceptual, personnel and spatial requirements necessary for an open door policy. Observation and door watch concepts are judged to be essential for open wards, and patients appreciate the therapeutic value they hold. However, nurses find the door watch problematic. All groups suggest seclusion or small locked divisions as a possible way of handling agitated patients. All stakeholders agree that such concepts can only succeed if sufficient, qualified staff is available. They also agree that freedom of movement is a key element in the management of acutely ill patients, which can be achieved with an open door policy. Finally, the interviewees suggested removing the door from direct view to prevent	Good

For peer review only

					absconding.	
Kulkarni, et al. (2014) Australia	Descriptive/Quantitative	Psychiatric ward (n=2)	Inpatients (n=65) and staff (n=20)	To evaluate the impact of a female-only area within a mixed-gender inpatient psychiatry service on female experiences of patient safety and care	There were significantly more patient safety incidents within the mixed-gender inpatient ward compared to the female only area. Specifically, female patients and staff perceived female-only wards as safer.	Fair
Muir-Cochrane, et al. (2012) UK	Semi-structured interviews/ Qualitative	Acute psychiatric unit	Participants (n=35); 43% female; mean age 43 years. Inpatients (n=15); visitors (n=6); nurses (n=14)	To explore perceptions of the acceptability of locking doors on inpatient wards	Several themes found that acknowledge the importance of locking doors on inpatient wards. Generally, all participants felt door locking helped reduce absconding. Staff felt guilt and fear of being blamed for the patient absconding, whereas patients expressed depression and low self-esteem when doors were locked.	Fair
Simpson, et al. (2011) UK	Cross-sectional/ Quantitative	Acute psychiatric wards (n=136)	Exit security staff and qualified nurses	To investigate the relationship between rates of drug/alcohol use on acute psychiatric wards and exit security measures	Daily rates of alcohol/drug use by wards are likely to be skewed, as few wards reported high levels. Door locking and security measures do not seem to be significantly related to alcohol use (r=-0.035, p=0.690) or other substance use (r = 0.108, p = 0.216).	Fair

For peer review only

Stolker, Nijman & Zwanikken (2006) Netherlands	Prospective/ Quantitative	A locked ward of a psychiatric hospital	Patients (n=54); 70% male; mean age 36, age range 18-58.	To explore whether residing in single- versus multiple-bed rooms in a psychiatric ward influenced psychiatric patients' views regarding seclusion	Findings suggest that seclusion is perceived as a less negative experience after having resided in a multiple-bed room before being secluded, $t(30) = 3.4$, $P < .05$, compared to patients residing in a single bed room.	Good
Triplett, et al. (2017) USA	Pre-post intervention and survey /Quantitative	Inpatient psychiatric units (n=2)	Nursing staff	To manage patient acuity with less reliance on observers, maintain a safe milieu, keep patient beds open to admissions, and reduce observer cost.	Both units saw decreases in acts of aggression (Unit A from a monthly average of 0.9 to 0.5 events per month; Unit B dropped from 0.2 events per month to 0.1), though neither was statistically significant. Unit A saw an increase in the overall number of patients requiring seclusion or restraints, but this actually represented a decrease when adjusted for the overall increase in patients seen, though neither was statistically significant. Unit B saw decreases in the number of patients put in seclusion or restraints during the pilot that were not statistically significant. Overall minutes in seclusion or restraints increased on Unit A, and this increase persisted when adjusted for increased patient throughput on the unit, though no measure was statistically significant.	Good

For peer review only

Van der Schaaf, et al. (2013) Netherlands	Longitudinal/ Quantitative	Forensic wards (n=25)	Patients (n=616); 78.7% male; mean age 37.6 (SD=10.5)	To explore the effect of design features on the seclusion risk, frequency, and duration within locked wards for intensive psychiatric care	Design features increasing the risk of being secluded were: 'presence of an outdoor space'; the availability of 'special safety measures'; and a large 'number of patients in the building'. Design features decreasing the risk of being secluded were: more 'total private space per patient'; a higher 'level of comfort'; and greater 'visibility on the ward'.	Good
Verbeek, et al. (2014) Netherlands Ì	Quasi-experimental/ Quantitative	Nursing homes with specialized psychogeriatric, somatic, or rehabilitation wards	Residents (n=124) in small-scale living facilities; 80% female; mean age 82.4 (SD=7.9). Control (n=135); 70% female; mean age 83.1 (SD=6.5)	To examine the effects of small-scale living facilities on residents' behaviour, focusing on neuropsychiatric symptoms, social engagement, and the use of physical restraints and psychotropic drugs	The small-scale facilities employed significantly fewer physical restraints and psychotropic drugs compared with traditional wards. Levels of social engagement and physically non-aggressive behaviour were higher in small-scale facilities than traditional wards.	Good
Wilkes, et al. (2005) Australia	Quasi-experimental/Quantitative	A Special Care Unit (SCU) at a nursing home	Persons with dementia (n=16); 81% female	To explore the effects on challenging (agitated) behaviours, when relocating patients with dementia to a special unit	Results revealed no significant differences in resident aggressive behaviour scores ($P = 0.220$) over time. Verbal agitation reduced throughout the 6 months of the study after moving into a SCU ($P < 0.01$).	Fair

For peer review only

Medication safety

Author, year, country	Study design	Setting	Participants	Aims/ objectives related to patient safety	Outcomes related to patient safety	Study quality
Bademli & Buldukoglu (2009) Turkey	Questionnaire/ Quantitative	Inpatient psychiatric wards in hospitals (n=34)	Psychiatric nurses (n=471); 100% female. Mean age 32.4+/-7.3	To describe oral medication management by nurses in psychiatric wards	73.9% of nurses collected medication history data, with 90.23% asking whether the patient had taken the medication previously. The orders were checked by 80.5% of nurses every day. If a nurse was not sure of something, 93.4% asked the physician. 59.7% of the nurses checked all the patients' mouths after each pill was given. The most common patient reaction during medication administration was refusal to take the medication (93.2%). 86.8% of nurses informed the physician when this occurred. Nurses primarily observed the patient to evaluate the effect of a medication (84.3%). Age, education and years of experience created a significant difference in approaches to patients who did not take their medications.	Fair
Cottney (2014) UK	Pre-post observation/ Quantitative	An acute adult inpatient mental health ward	Staff	To evaluate the benefits of automated dispensing cabinets (ADCs) for increased medication safety	There was a reduction in the rate of medication administration errors from 8.9% to 7.2%, but this was not statistically significant (p=0.065, 95% CI 0% to 3.5%.	Poor

Cottney & Innes (2015) UK	Observation/ Quantitative	Inpatient mental health wards (n=43) within a mental health trust	Staff and patients	To explore medication-administration errors within a mental health trust	In 4177 medication administration opportunities, 139 medication errors were detected (3.3%). The error most frequently made was incorrect dose omission (37%). 11% of the errors had a serious clinical severity level. Four factors predicted medication error: administering nurse having to attend to other duties during the medication round (relative risk (RR) =1.48, 95% (CI): 1.14–1.93, p=0.003); number of 'pro re nata' (prn) doses given (RR=1.15, 95% CI: 1.03–1.28, p=0.012); number of patients on the ward (RR=1.06, 95% CI: 1.01–1.13, p=0.03); and number of regular doses due (RR=1.02, 95% CI: 1.02–1.03, p=≤ 0.0001).	Good
Dickens, Stubbs & Haw (2008) UK	Observational/Quantitative	Inpatient wards for older people (n=2) within a psychiatric hospital	Registered nurses (n=9)	To explore the delegation of medication administration within older people mental health wards	78% of delegated medication doses were to another registered nurse, but 22% were delegated to care workers. Care workers were more likely to administer medication to aggressive/confused patients. Errors occurred in 20% of doses, with the most common being the opening/crushing of medication without authorisation (41% of errors). 99% of errors were in the preparation/recording and so not attributable to the administrator of the medication.	Fair

For peer review only

Dolan & Kirwan (2001) UK	Questionnaire/ Quantitative	A medium secure unit for mentally disordered offenders (MSU)	Staff (n=62)	To explore staff perceptions of illicit drug use and its impact on MSUs	60% of staff reported awareness of drug misuse on the unit. The majority of staff (82%) mentioned the adverse effects on psychopathology (worsening symptoms, aggression). 45% also expressed concern regarding negative impacts upon staff-patient relationships. Most staff commented that the unit had policies of bag, room or visitor searching. 31% of staff felt that the police should be called if patients were in possession of drugs, 40% felt that sanctions should be imposed.	Fair
Haw, et al. (2007) UK	Cross-sectional/ Mixed methods	Elderly long-stay wards in a psychiatric hospital (n=2)	Nurses (n=9)	To use observations to determine the frequency and nature of medication administration errors	In 1423 doses, 369 medication errors were made (25.9%). The most frequent error type was crushing tablets without the authorization of the prescriber (28.7%). The median rate of medication error was one in every 6.4 doses.	Poor
Haw, et al. (2014) UK	Vignettes and semi-structured interviews/ Qualitative	Psychiatric hospital	Nurses (n=50)	To explore reasons for not reporting medication errors	48% of nurses stated that they would report a medication error made by someone else, and 40% would report a near-miss involving themselves. Thematic analysis revealed reasons for this was four-fold: knowledge, fear, burden of work, and excusing the error.	Fair
Hughes, et al. (2018) Europe, Australasia, the Middle East, South East Asia, North America and Africa	Online survey/ Quantitative	Inpatient mental health setting	Mental health professionals (n=98) working in Europe (n=77) 79%, Australasia (n=9) 9%, the Middle East (n=6) 6%, South East Asia (n=4) 4%, North America (n=1) 1% and	To undertake a scoping survey to explore inpatient mental health workers' perceptions of novel psychoactive substances (NPS) use by consumers.	Over 90% of participants reported observing at least one adverse event relating to NPS use in the previous month. The majority of participants reported that patients had used NPS during their inpatient admission. Three quarters	Fair

For peer review only

			Africa (n=1) 1%.		were not clear if their workplace had a policy about NPS. Most wanted access to specific NPS information and training. Participants reported that they lacked the necessary knowledge and skills to manage NPS use in the patients they worked with.	
Gonzalez-Pinto, et al. (2010) Spain	Prospective Observational/ Quantitative	An inpatient setting	Non-adherent inpatients (n=161) and adherent inpatients (n=460)	To describe the frequency of adherence and non-adherence with anti-manic and mood stabilizing medication among bipolar disorder patients, and to identify factors associated with treatment adherence	Higher levels of insight was associated with higher levels of medication adherence. Cannabis abuse/dependence during the acute phase of the patient's condition, work impairment, and higher levels of hallucination/delusion at baseline were associated with lower levels of medication adherence.	Fair
Keers, et al. (2018) England	Interviews/Qualitative	A mental health National Health Service (NHS) hospital.	20 Student and Registered Mental Health Nurses	To investigate in-depth the underlying causes of MAEs in a mental health National Health Service (NHS) hospital.	The majority of errors involved medications used to treat central nervous system (CNS) disorders (n = 21), and most commonly involved either wrong drug (n = 7), wrong patient (n = 5) or wrong dose errors (n = 4). The reported MAEs involved nurses of varying levels of experience post-qualification, with over half occurring when participants had 1 year or less (n = 14, 54%) experience (including 2 student nurse errors) and 7 (27%) when nurses had more than 5 years' experience. Each error and near miss (active failure) was preceded by combinations of underlying error and violation	Good

For peer review only

					provoking conditions.	
Prins, et al. (2013) Netherlands	Observation and structured interviews/Quantitative	An inpatient old age psychiatric clinic of a large psychiatric teaching hospital	Patients (n=50); mean age 68.9; 52% female	To compare the number of medication use discrepancies at admission when using the structured history of medication use (SHIM) procedure and usual procedure for taking medication history	Compared with usual care, the SHIM procedure identified a discrepancy in medication use in 78% of patients, and provided a more comprehensive and accurate approach to obtain medication history.	Good
Rodreguez-Leal, et al. (2016) Spain	Longitudinal/Quantitative	All in-patients addmitted to a psychiatric hospital	Psychiatric inpatients (n=225), 54% male, mean age 54.64. 76.7% had schizophrenia and schizoaffective disorders. 9.2% had major affective disorder. 5.5% of patients had a diagnosis form axis II as main diagnosis. The rest (8.6%) had other mental illness.	To detect QT interval prolongation among inpatients in a mental health setting	Over the 12-month study period, 225 patients were evaluated and 9 cases (4%) of long QT segment were detected. Findings led to treatment modification and patient close monitoring. No sudden cardiac deaths occurred during the study period.	Fair
Ružić, et al. (2011) Croatia	Cross-sectional/ Quantitative	Forensic psychiatric institutions (n=2)	Psychiatric patients (n=98); mean age 47	To examine the effect of antipsychotics selection (typical or atypical) on patient aggressiveness, side effects, and hospitalisation length	There were no significant differences between participants receiving typical and atypical antipsychotics.	Fair
Seemüller, et al. (2009) Germany	Naturalistic prospective/ Quantitative	Psychiatric university hospitals (n=7) and psychiatric district hospitals (n=5)	Inpatients (n=1014); 63% female; mean age 45.03 (SD=11.89)	To explore any suicidality promoting effects of antidepressants that cause an increased rate of suicidality-related events in high risk populations under routine treatment conditions	The rate of suicides (13.44/1000 patient-years) was low in the routine treatment condition compared to the rate observed in randomized controlled antidepressant trials. Predictors of extended emergence of suicide ideation were: Age (younger individuals were at higher risk), treatment	Good

For peer review only

					resistance, number of hospitalizations, and presence of akathisia and comorbid personality disorder.	
Sørensen, et al. (2013) Denmark	Descriptive, cross-sectional/ Quantitative	Psychiatric wards at a university hospital (n=3)	Patients (n=67); 64% female; mean age 46, age range 20-79	To investigate errors in the medication process in an inpatient psychiatric setting	There were errors in 9/324 (3%) opportunities for error of dispensed drugs in the observational study, and in 9/67 (13%) of dispensed drugs in the unannounced control visit, of which the majority was associated with one nurse assistant. Fewest errors were detected at the prescribing stage.	Fair
Strudwick, et al. (2017) Canada	Interviews/ Qualititative	Inpatient care settings: forensic, youth, geriatric, acute, and rehabilitation services (n=5)	Inpatients (n=52)	To elicit patient perceptions of barcode medication administration identification practices in inpatient mental health settings	Six themes emerged as a result of the inductive data analysis. These included: management of information, privacy and security, stigma, relationships, safety and comfort, and negative associations with the technology. Patients also indicated that they would like a choice in the type of identification method used during barcode medication administration. As well, suggestions were made for how barcode medication administration practices could be modified to become more patient-centered.	Good

For peer review only

Xie, at al. (2019) Canada	Interviews/Qualitative	Mental health and addiction academic teaching hospital	Mental health nurses (n=10).	To explore mental health nurses' perceptions of practices to improve barcode medication administration take-up in mental health inpatient settings	Interview themes were: safety, clinical workflow, education, accountability and strategies	Good

For peer review only

Unauthorised Leave

Author, year, country	Study design	Setting	Participants	Aims/ objectives related to patient safety	Outcomes related to patient safety	Study quality
Algase, et al. (2010) USA	Observational, questionnaire/ Quantitative	Long-term care units (LTCs) with dementia-specific units (N=28). These included nursing homes (n=22) and assisted living facilities (n=6)	Residents with dementia (n=122); 77% female; mean age 83.7 (range 68-102)	To investigate the influence of the physical environment on wandering behaviour	80% of wandering occurred in the residents' room, the community day room, the hall, or the eating halls. Location, light and sound impacted upon wandering behaviour. Odds ratio was highest for proximity to people (odds [OR] = 1.87).	Good
Bowers, Alexander & Gaskell (2003) UK	Controlled trial/ Quantitative	Acute psychiatric wards (n=5) in a hospital	All staff and patients within the units	To evaluate an intervention to reduce absconding by patients from acute psychiatric wards	Absconding fell by 25% during the intervention. Severe violent incidents increased significantly during the intervention ($\chi 2=7.74$, P=0.005). However, wards that saw a decrease in absconding, there was no change in the frequency of violent incidents, and there were significant decreases in door locking.	Good
Colombo, et al. (2001) Italy	Questionnaire/ Quantitative	An SCU	Inpatients (n=132). 100% female. Mean age 78.6±9.4	To develop a profile of female wanderers with dementia within an SCU	Wandering occurred in 51% of participants. Wanderers were found to fall 3 times as much as non-wanderers over 3 months (1.6 and 0.6 falls respectively).	Poor
Grotto, et al. (2015) Australia	Interpretative inquiry/ Qualitative	Inpatient mental health units (n=3)	Mental health nurses (n=11); 55% female; age range (35-60)	To explore nurses' perceptions of assessment and management practices inpatient absconding	Clinical judgement influenced absconding assessment including historic markers of absconding. Nurses deemed risk assessment methods to be inadequate. There was no standardised management for dealing with absconding patients.	Fair
Hunt, et al. (2010) UK	Descriptive/ Quantitative	Psychiatric inpatient wards	Inpatient suicides away from ward (n=469); 66% male	To describe inpatient suicides by individuals who had absconded and to compare against those on agreed leave	Absconders were significantly more likely to die by jumping than those on agreed leave (49% vs 30%, p< 0.001 respectively). Absconders were also more likely to die within the first week of admission (19% vs 8%, p< 0.001) and to be non-compliant with medication (25% vs 13%, p< 0.001). Factors that could have	Fair

For peer review only

					reduced the likelihood of suicide were suggested: more patient supervision, better treatment compliance, and increased staff numbers, communication and training.	
Hunt, et al. (2016) UK	Questionnaire and interview/ Mixed methods	Inpatient ward	Mental health staff (n=21). 57% male. Median age 46 (range 37–58).	To identify the characteristics of inpatients who died by suicide after absconding and to explore these and further key issues related to suicide risk from the perspective of clinical staff.	Four themes were identified as areas of concern for clinicians: problems with ward design, staffing problems, difficulties in assessing risk, and patient specific factors.	Good
Meehan, Morrison & McDougall (1999) Australia	Semi-structured interviews/ Quantitative	Acute psychiatric unit in a general hospital campus	Inpatients (n=14); 36% female; mean age 37 (range 19-58 years)	To explore absconding behaviour from the patient perspective	Six issues were identified regarding absconding behaviour: boredom, lack of interesting activities, disturbed ward environment, the need for hospitalisation, issues at home, and perceived rewards from absconding. Situational and environmental factors were likely to increase the absconding risk.	Poor
Muir-Cochrane, et al. (2013) Australia	Semi-structured interviews/ Qualitative	An acute psychiatric unit	Mental health patients (n=12). 33% male	To explore the experiences of people who had been held involuntarily in an inpatient psychiatric unit and who had absconded or attempted to abscond	The inpatient unit is perceived as a safe or unsafe place, depending on physical, individual, social, and symbolic aspects of the unit. Absconding occurred when the unit was perceived as unsafe. Factors that would decrease the likelihood of absconding were identified: a therapeutic relationship with staff, familiarity with the unit, a comfortable environment, and positive interactions with peers.	Fair

For peer review only

Nijman, et al. (2011) UK	Questionnaire/ Quantitative	Acute psychiatric wards (n=133)	A qualified nurse	To investigate the prevalence of door locking and the use of other exit security measures on psychiatric wards, as well as investigating relationships between locking exit doors and absconding	There was no statistically significant relationship between exit security measures and absconding rates. However, there was a reduction of approximately 30% in absconding rates when the ward door was permanently locked compared to when doors were open.	Fair
Nurjannah, FitzGerald, & Foster (2009) Indonesia	Semi-structured interviews/ Qualitative	A psychiatric hospital	Patients (n=16). 94% male; mean age 33, range 13-65	To provide a rich description of the experience of patients related to incidents of absconding in a psychiatric setting	Three themes were highlighted: the call to home, hopes and realities, and us and them. The first theme concerned patients' need to connect with others and to feel safe. The second theme concerned patients' wishes to feel happy, which were often not compatible with realities of life at home or in the ward. The final theme concerned the competing interests and different opinions of patients regarding others, such as hospital staff and family.	Fair
Simpson, et al. (2015) Canada	Intervention design/ Quantitative	Forensic inpatient units (n=8) within a psychiatric hospital	Inpatients (n=188)	To investigate the impact of a new policy designed to reduce incidents of absconding in a forensic setting	The absconding rate decreased from 17.8% pre-implementation, to 13.8% during implementation, and further to 12.0% post-implementation. The most common reason for absconding was boredom/frustration.	Good

For peer review only

Clinical Decision Making

Author, year, country	Study design	Setting	Participants	Aims/ objectives related to patient safety	Outcomes related to patient safety	Study quality
Brown & Rakow (2015) UK	Questionnaire/ Quantitative	Psychiatric hospitals	Clinicians with an active role in violence risk assessment (n=9)	To explore clinicians' use of static and dynamic cues when assessing risk in psychiatric inpatients	Clinicians mostly used history of recent violence cues in assessments of in-hospital risk. Clinicians mostly used in-hospital frequency and severity of violence cues when assessing the risk posed by the patient if they remained in the current hospital.	Fair
Fuller & Cowan (1999) UK	Natural experiment/ Quantitative	Inpatient units (n=2) within a forensic hospital	Patients (n=75). 87% male. Modal age group 20-29	To assess the accuracy of staff predictions of patient clinical risk in forensic settings	The most common risk event was acting out against fellow patients (25% of risk events). Multidisciplinary clinical judgement predicted a variety of risk events at a higher than chance level. The risk event with the highest AUC statistic was prediction of serious incidents involving staff (AUC=0.856, p=≤ 0.0001).	Fair
Green, et al. (2018) UK	Focus group/ Qualitative	Medium secure ward	Registered nurses (n=12). 75% female.	To specifically explore the decision-making process behind qualified nurses' decisions to implement the use of seclusion in forensic mental health care.	Participants described the need to reduce the use of seclusion and the problematic nature of its utility as an ongoing intervention in contemporary mental healthcare. It was clear that there were complexities and competing variables involved in the decision-making process. The data analysis resulted in the identification of four themes: 1) seclusion as a last resort, 2) presenting behaviours, 3) organisational influences and 4) professional judgement.	Good
Koukia, Madianos & Katostaras (2009) Greece	Cohort design/ Qualitative	Psychiatric hospital	Nurses (n=103); 57% female; mean age 36.3 [7.7]	To explore the on-the-spot decision making process of nurses when faced with a critical incident	When violent incidents occur, physical restraints were frequently used. However, reassurance and support were common interventions when a critical incident occurred. Nurses wanted	Fair

For peer review only

					more skills training and higher autonomy.	
Lindsey (2009) USA	Correlational descriptive design/ Quantitative	Psychiatric hospital	Nurses (n=30); 87% female	To explore nurses' decisions to restrain psychiatric inpatients, and associations with work empowerment and individual patient/staff characteristics	When needed nurses chose high frequency medication and did not display a standardised decision making process in their restraint use.	Fair
Mann-Poll, et al. (2011) Netherlands	Vignette and questionnaire/ Quantitative	Inpatient wards (n=4)	Staff (n=82)	To identify factors that contribute to decision making in relation to seclusion	The factors contributing to decisions to seclude patients came mostly from rater characteristics (e.g. type of care received from staff, frequency of participation in seclusion, experience of using seclusion as an intervention) and vignette variables (e.g. how approachable patient seemed, dangerousness and availability of rooms).	Fair
Marangos-Frost & Wells (2000) Canada	Ethnographic/ Qualitative	Psychiatric inpatient unit	Staff (n=6)	To understand nurses' decisions to restrain patients and feelings about those decisions	There were four main themes: framing the situation as a potential for immediate harm; unsuccessful exploration of alternative interventions; the conflicted nurse; and the conditions of restraint	Fair
Whaley (2001) USA	Cross-sectional/ Quantitative	A psychiatric hospital	Patients (n=118), age range 18-59	To evaluate agreement between clinical diagnoses and research diagnoses of schizophrenia for African American psychiatric patients. To explore the relationship of cultural mistrust with any disagreement	Levels of agreement for diagnoses was poor in 5 out of 6 comparisons. There were, however, significantly more cases using clinical diagnoses than other methods. Level of cultural mistrust did not seem to predict the excess in clinical diagnoses of paranoid schizophrenia.	Fair

For peer review only

Whaley (2004) USA	Cross-sectional/ Quantitative	A psychiatric hospital	Patients (n=94)	To explore cultural bias and diagnoses of schizophrenia for African American psychiatric patients	Highest rates of agreement were found between clinical and best estimate diagnoses of schizophrenia (kappa=.77, p< .01). Patients' cultural mistrust was positively associated with the number of clinical diagnoses of paranoid schizophrenia, b=.364, se=.123, p < .01, and best estimate diagnoses of paranoid schizophrenia, b= .314, se= .128, p< .05. This implies a bias towards diagnosing cultural attitudes as pathology in African American psychiatric patients.	Fair

Falls

Author, year, country	Study design	Setting	Participants	Aims/ objectives related to patient safety	Outcomes related to patient safety	Study quality
Abraham (2016) USA	Cross-sectional/Quantitative	Inpatient psychiatric units, USA	Psychiatric inpatient program supervisors, managers, directors, or administrators (n=80). 45.5% were 55-64, 36.4% were 45-54. 3.0% were 25-34. One participant was older than 65.	To explore psychiatric unit directors' perceptions of the factors that contribute to patient falls in psychiatric inpatient units in the State of Michigan.	Team work was the most common extrinsic factor related to decreasing patient falls (M, 4.55 [0.61]). Supervision and 15-min checks were also deemed relevant (M 4.00 [0.88] and M 3.56 [0.84] respectively). Participants agreed mostly that staff training and education rather than employing certified psychiatric technicians were needed to prevent falls in the unit (M, 4.32 [0.59 and 4.05 [1.07] respectively). One on one supervision had the lowest level of agreement as factor to reduce falls in the unit (M 2.92 [1.19]).	Fair

Fonad, et al. (2009) Sweden	Longitudinal/Quantitative	Dementia nursing home units	Staff nurses	To explore associations between falls and: fall risks, fractures, the use of physical restraints and the use of medications in dementia nursing home units	737 fall incidents ocurred over the 4 year period. Falls were associated with fractures, assessed risk of falling, the use of certain medication (neuroleptics and sleeping medication), and physical restraints (wheelchairs, belts and bed rails).	Fair
Garfinkel, et al. (2007) Israel	Longitudinal case-control/Quantitative	Dementia-specialised departments (n=4) within a medical centre	Patients (n=206).	To evaluate the efficacy of hip protectors in reducing hip fractures in dementia patients.	There was no significant difference in the fall rate between patients wearing/not wearing hip protectors. There was a significant decrase in the rate of hip fracture per fall for those patients wearing hip protectors (P= 0.007, relative risk (RR)=5.63).	Fair
Holmes, et al. (2007). USA	Quasi-experimental study	Special care unit (SCU) for inpatients with dementia	Residents with dementia (n=78): intervention group (n=38) and comparison unit (n=40); no other details mentioned	To assess the extent to which modern technology can augument or substitute for direct staff intervention in late-evening and nighttime situations. Specifically that modern technology would significantly contribute to the reduction of falls, accidents and injuries	The modern technology intervention did not significantly reduce the number of falls or accidents on the unit compared to comparison unit. Furthermore there was no extra staff-perceived burden with the presence of the intervention.	
Powell-Cope, et al. (2014). United States.	Descriptive Study using focus groups	One inpatient unit at a Veterans' hospital	Registered nurses (n=22), physical therapist (n=1) and physicians (n=2)	Determine the barriers and facilitators for implementing fall prevention and protection programs in inpatient psychiatry.	Results were grouped into 3 fall-related categories: Fall Risk Assessment (e.g. Participants noted a ceiling effect with existing fall risk instruments and lack of sensitivity to psychiatry populations) Clinical Fall Risk Precautions (e.g. use of bed modifications to improve mobility), Programmatic Fall Prevention (e.g. The necessity of using an interdisciplinary focus for falls prevention programs was	Good

For peer review only

					echoed by all study participants)	
Tängman, et al. (2010). Sweden.	Prospective study	A 24 bed psychogeriatric ward	Patients (n=91); 50.5% female; mean age 80.3, age range 60-94 (SD=7.4)	The aim of this study was to identify precipitating factors for falls among people with dementia.	The most likely factor or combination of factors could be ascertained in 247 falls (83%). Acute disease or symptoms of disease and/or acute drug side-effects were, alone or in combination with other factors, judged to precipitate more than three out of four falls.	Fair

Infection prevention and control

Author, year, country	Study design	Setting	Participants	Aims/ objectives related to patient safety	Outcomes related to patient safety	Study quality
Li, et al. (2019) Taiwan	Interviews/ Qualitative	Psychiatric hospitals (n=6) and general hospitals with psychiatric facilities (n=2)	Psychiatric infection preventionists (n=13). 100% female. Mean age 41.9 (range 31- 48)	To understand how infection preventionists (IPs) perceived their challenges and how these challenges negatively affect their infection prevention work in psychiatric clinical settings.	Data analysis revealed that the participants' experiences of working as IPs in the psychiatric wards and associated challenges were captured by 6 themes: (1) lack of preservice training in psychiatric infection control, (2) insufficient staffing in practice, (3) working within environmental limits, (4) patient noncompliance, (5) undervaluation of the importance of infection control by professionals, and (6) involvement of hospital administrators.	Fair

For peer review only

**Author, year**	**Abstract and title:** *Did they provide a clear description of the study?*	**Introduction and aims:** *Was there a good background and clear statement of the aims of the research?*	**Method and data:** *Is the method appropriate and clearly explained?*	**Sampling:** *Was the sampling strategy appropriate to address the aims?*	**Data analysis:** *Was the description of the data analysis sufficiently rigorous?*	**Ethics and bias:** *Have ethical issues been addressed, and necessary ethical approval gained? Has the relationship between researchers and participants been considered?*	**Results:** *Is there a clear statement of the findings?*	**Transferability or generalizability:** *Are the findings of this study transferable to a wider population?*	**Implications and usefulness:** *How important are these findings to policy and practice?*	**Overall Quality Score**	**Overall Quality Rating**
Abdel-Hussein & Mohamed (2018)	20	20	20	20	10	10	20	20	10	16.67	**Poor**
Abraham (2016)	20	20	20	30	20	0	30	20	20	20.00	**Fair**
Ajalli et al. (2018)	30	20	30	20	40	30	40	30	30	30.00	**Good**
Algase et al. (2010)	30	30	30	30	40	20	40	30	30	31.11	**Good**
Almvik, Rasmussen & Woods (2006)	30	20	20	20	20	20	40	20	20	23.33	**Fair**
Amoo & Fatoye (2010)	30	30	20	20	30	20	40	20	40	27.78	**Fair**
Arguvanli, et al. (2015)	30	20	30	20	30	20	30	20	30	25.56	**Fair**
Awenat et al. (2018)	40	40	30	30	30	30	30	40	40	34.44	**Good**
Bademli & Buldukoglu (2009)	30	20	30	20	30	20	40	20	40	27.78	**Fair**
Bahareethan & Shah (2000)	30	20	30	20	30	20	30	20	40	26.67	**Fair**
Bak & Aggernæs	30	20	20	10	10	0	30	20	20	17.78	**Poor**

For peer review only

(2012)											
Bak, et al. (2013)	30	20	30	30	40	20	40	30	40	31.11	**Good**
Bak, et al. (2015)	30	20	30	30	40	20	40	30	30	30.00	**Good**
Barr et al. (2019)	30	40	30	30	30	20	30	30	30	30.00	**Good**
Bayramzadeh (2016)	30	30	10	10	10	20	20	20	20	18.89	**Poor**
Bellantonio, et al. (2008)	30	20	40	30	30	20	40	30	40	31.11	**Good**
Bennett, Ramakrishna & Maganty (2011)	20	20	20	20	40	0	40	20	40	24.44	**Fair**
Ben-Zeev et al. (2017)	30	20	30	30	30	10	30	30	30	26.67	**Fair**
Berg, et al. (2013)	40	20	40	20	30	20	40	20	40	30.00	**Good**
Berg, Kaltiala-Heino & Välimäki (2011)	30	40	40	20	40	20	40	20	30	31.11	**Good**
Bergk, et al. (2011)	40	20	30	20	30	20	40	20	30	27.78	**Fair**
Bharwani, et al. (2012)	30	20	30	20	20	0	30	20	30	22.22	**Fair**
Biancosino, et al. (2009)	30	30	30	20	30	0	40	20	40	26.67	**Fair**
Bigwood & Crowe (2008)	30	20	40	20	40	20	30	20	30	27.78	**Fair**
Bilici et al. (2016)	20	30	30	20	30	20	30	20	40	26.67	**Fair**
Björkdahl, Hansebo & Palmstierna (2013)	30	20	30	20	30	20	40	20	30	26.67	**Fair**
Blair et al (2017)	30	20	20	30	30	20	30	30	20	25.56	**Fair**
Bleijlevens,	40	30	30	20	30	20	30	20	30	27.78	**Fair**

For peer review only

et al. (2013)											
Bonner & Wellman (2010)	30	20	40	20	20	20	40	20	40	27.78	**Fair**
Bonner, et al. (2002)	20	20	40	20	30	20	30	20	40	26.67	**Fair**
Booth, et al. (2014)	30	20	30	20	20	20	40	20	40	26.67	**Fair**
Boström, et al. (2011)	30	40	30	20	20	20	40	20	40	28.89	**Fair**
Boumans, et al. (2012)	20	40	30	20	30	0	40	20	40	26.67	**Fair**
Bowers & Crowder (2012)	40	20	30	30	40	20	30	30	40	31.11	**Good**
Bowers (2009)	30	30	30	30	40	20	30	30	40	31.11	**Good**
Bowers, Alexander & Gaskell (2003)	40	20	30	30	30	20	40	30	30	30.00	**Good**
Bowers, et al. (2006)	30	20	30	20	20	20	40	20	30	25.56	**Fair**
Bowers, et al. (2007)	40	20	20	20	30	20	30	20	40	26.67	**Fair**
Bowers, et al. (2008)	30	20	30	30	40	20	40	30	40	31.11	**Good**
Bowers, et al. (2009)	30	20	30	30	30	20	30	30	40	28.89	**Fair**
Bowers, et al. (2010)	30	20	30	30	30	20	30	30	30	27.78	**Fair**
Bowers, et al. (2012)	30	20	30	30	30	20	30	30	30	27.78	**Fair**
Bowers, Gournay & Duffy (2000)	40	20	30	20	20	0	30	20	30	23.33	**Fair**
Brady et al. (2017)	40	30	30	30	30	20	30	30	40	31.11	**Good**
Braham, Heasley & Akiens (2013)	30	20	30	20	20	30	30	20	30	25.56	**Fair**

For peer review only

Brennan, Flood & Bowers (2006)	20	20	30	30	20	30	40	30	30	27.78	**Fair**
Brown & Beail (2009)	40	20	30	20	40	30	40	20	30	30.00	**Good**
Brown & Rakow (2016)	40	20	40	20	30	20	30	20	40	28.89	**Fair**
Calabro, Mackey & Williams (2002)	30	20	30	20	30	20	40	20	40	27.78	**Fair**
Camuccio, et al. (2012)	30	30	30	20	20	20	40	20	40	27.78	**Fair**
Carlson, et al. (2010)	30	20	30	20	10	20	30	10	20	21.11	**Fair**
Caspi (2014)	40	20	30	20	20	40	40	20	40	30.00	**Good**
Caspi (2015)	20	30	30	20	30	40	40	20	40	30.00	**Good**
Caspi, et al. (2001)	30	20	30	20	20	20	40	20	30	25.56	**Fair**
Chan, et al. (2005)	30	20	30	30	10	20	40	30	20	25.56	**Fair**
Chandler (2008)	20	20	30	20	30	20	40	20	40	26.67	**Fair**
Chaplin, et al. (2008)	0	20	20	20	0	0	40	20	20	15.56	**Poor**
Chen, et al. (2007)	30	20	40	20	20	20	30	20	30	25.56	**Fair**
Chen, Hwu & Wang (2009)	30	20	30	20	30	20	30	20	30	25.56	**Fair**
Chien, Chan & Kam (2005)	30	20	40	20	30	20	40	20	30	27.78	**Fair**
Ching, et al. (2010)	20	40	30	20	40	20	40	20	40	30.00	**Good**
Chu, et al. (2015)	30	20	30	20	0	10	40	20	30	22.22	**Fair**
Cleary, et al. (1999)	20	20	30	20	20	0	40	20	30	22.22	**Fair**
Cole,	30	20	20	20	30	0	30	20	30	22.22	**Fair**

For peer review only

Baldwin & Thomas (2003)											
Colombo, et al. (2001)	20	20	20	20	10	0	30	20	20	17.78	**Poor**
Cormac, Russell & Ferriter (2005)	0	20	20	20	10	0	30	20	30	16.67	**Poor**
Cottney & Innes (2015)	30	20	40	20	30	30	40	20	40	30.00	**Good**
Cottney (2014)	30	20	30	10	0	0	30	20	30	18.89	**Poor**
Cowan et al. (2018)	30	20	30	20	40	20	40	20	30	27.78	**Fair**
Cowman & Bowers (2008)	30	20	20	20	40	0	30	20	40	24.44	**Fair**
Cullen, Nath & Marcus (2010)	30	20	20	20	40	20	30	20	40	26.67	**Fair**
Curtis, et al. (2013)	20	20	20	20	30	20	30	20	30	23.33	**Fair**
Cutcliffe (1999)	20	20	30	20	30	20	30	20	40	25.56	**Fair**
Daffern (2007)	20	20	20	20	0	10	30	20	40	20.00	**Fair**
Daffern, et al. (2009)	20	20	30	20	30	20	40	20	40	26.67	**Fair**
Daffern, Mayer & Martin (2006)	20	40	40	10	30	10	30	20	40	26.67	**Fair**
Daffern, Ogloff & Howells (2003)	20	20	40	10	10	20	30	20	40	23.33	**Fair**
Danivas et al. (2016)	40	30	40	30	40	20	40	20	40	33.33	**Good**
Davies et al. (2019)	30	30	30	20	30	20	30	20	40	27.78	**Fair**
Davis,	20	20	30	20	20	20	30	20	40	24.44	**Fair**

For peer review only

Williams & Hays (2002)											
de Jonghe-Rouleau, Pot & de Jonghe (2005)	20	20	30	30	20	10	40	30	30	25.56	**Fair**
de Looff et al. (2018)	40	40	30	30	40	20	30	30	40	33.33	**Good**
De Niet, Hutschemaekers & Lendemeijer (2005)	30	30	30	20	20	0	40	20	30	24.44	**Fair**
De Young, Just & Harrison (2002)	30	20	30	20	20	20	30	20	40	25.56	**Fair**
Delaney & Johnson (2006)	30	20	30	20	30	20	40	20	0	23.33	**Fair**
Delaney, et al. (2001)	20	20	20	20	20	0	30	20	40	21.11	**Fair**
Dickens, Piccirillo & Alderman (2013)	20	20	30	20	40	20	30	20	30	25.56	**Fair**
Dickens, Stubbs & Haw (2008)	30	20	40	20	10	30	30	20	40	26.67	**Fair**
Dolan & Kirwan (2001)	40	20	20	20	0	0	40	20	30	21.11	**Fair**
Drew (1999)	30	20	30	20	0	10	40	20	40	23.33	**Fair**
Dreyfus et al. (2018)	30	30	30	20	20	10	40	20	10	23.33	**Fair**
Duxbury & Whittington (2005)	30	20	30	20	20	20	30	20	40	25.56	**Fair**
Duxbury et al. (2019)	30	30	20	10	20	10	40	20	30	23.33	**Fair**
Ellis, et al. (2012)	20	20	30	20	30	20	40	20	30	25.56	**Fair**

For peer review only

Ellis, et al. (2015)	20	30	30	20	30	20	40	20	40	27.78	**Fair**
Elmer et al. (2018)	30	20	20	20	30	20	30	20	40	25.56	**Fair**
Elzubeir et al (2017)	40	30	40	20	40	30	40	20	40	33.33	**Good**
Espinosa, et al. (2015)	30	20	20	10	0	0	20	20	30	16.67	**Poor**
Esposito-Smythers, McClung & Fairlie (2006)	20	20	30	20	10	20	40	20	40	24.44	**Fair**
Evans & Petter (2012)	30	30	30	20	30	20	30	20	40	27.78	**Fair**
Exworthy, et al. (2001)	20	20	30	20	0	0	40	20	20	18.89	**Poor**
Ezeobele, et al. (2014)	30	30	40	20	30	20	40	20	40	30.00	**Good**
Faschingbauer, Peden-McAlpine & Tempel (2013)	30	20	40	30	30	20	40	20	30	28.89	**Fair**
Fish & Hatton (2017)	20	30	20	10	20	30	40	20	20	23.33	**Fair**
Fish (2018)	30	20	20	10	20	30	30	20	30	23.33	**Fair**
Fletcher et al. (2019)	30	20	40	20	30	20	30	20	30	26.67	**Fair**
Foley, et al. (2003)	20	30	40	20	30	20	40	20	40	28.89	**Fair**
Fonad, et al. (2009)	30	30	30	20	30	10	40	20	40	27.78	**Fair**
Fuller & Cowan (1999)	20	20	40	20	30	0	30	20	40	24.44	**Fair**
Gabrielsson, et al. (2014)	30	20	30	20	30	20	40	20	40	27.78	**Fair**
Gallop, et al. (1999)	20	30	40	20	40	20	40	20	30	28.89	**Fair**

For peer review only

Garfinkel, et al. (2007)	30	10	40	20	20	20	30	20	30	24.44	**Fair**
Gebhardt & Steinert (1999)	30	20	30	20	30	0	40	20	30	24.44	**Fair**
Georgieva, Mulder & Noorthoorn (2013)	30	20	40	30	40	30	40	30	40	33.33	**Fair**
Georgieva, Mulder & Wierdsma (2012)	20	20	30	30	40	20	40	30	40	30.00	**Fair**
Gerace et al. (2018)	30	20	20	20	30	30	20	20	20	23.33	**Fair**
Gibson et al. (2014)	30	20	40	30	40	20	40	20	30	30.00	**Fair**
Gifford & Anderson (2010)	30	30	30	20	30	20	40	30	30	28.89	**Fair**
Giles et al. (2005)	30	40	40	30	10	0	30	20	20	24.44	**Fair**
Gonzalez-Pinto et al. (2010)	30	40	30	20	40	30	40	20	30	31.11	**Good**
Gough & Hawkins (2000)	30	20	30	30	20	10	30	20	30	24.44	**Poor**
Goulet et al (2017)	30	30	30	40	40	20	30	40	40	33.33	**Good**
Goulet et al (2018)	30	20	40	30	40	20	40	30	40	32.22	**Good**
Gowda et al (2018)	30	30	30	20	20	20	20	20	40	25.56	**Fair**
Gowda et al (2019)	30	20	20	20	20	20	30	20	40	24.44	**Fair**
Gowda et al. (2019)	30	20	20	10	20	20	20	20	20	20.00	**Fair**
Green et al. (2018)	30	30	40	20	30	30	40	20	30	30.00	**Good**
Grotto et al. (2014)	30	20	30	20	40	20	30	20	40	27.78	**Fair**

Gustafsso et al (2016)	30	30	30	20	40	30	40	20	30	30.00	**Good**
Haglund & von Essen (2005)	30	20	30	20	30	20	30	20	20	24.44	**Poor**
Haines et al. (2017)	40	30	30	20	40	20	40	20	40	31.11	**Good**
Hallett & Dickens (2015)	40	30	30	40	30	20	40	30	40	33.33	**Fair**
Happell & Koehn (2011)	30	30	40	20	40	20	40	20	40	31.11	**Fair**
Hatta et al. (2007)	30	20	30	20	30	10	40	20	30	25.56	**Fair**
Haugom & Granerud (2016)	30	20	30	10	40	30	40	20	40	28.89	**Fair**
Haw et al. (2007)	40	20	30	20	20	20	20	20	30	24.44	**Poor**
Haw et al. (2011)	30	20	40	20	40	20	30	20	40	28.89	**Fair**
Haw et al. (2014)	40	20	40	20	40	20	40	20	30	30.00	**Fair**
Higgins et al. (2018)	40	30	30	20	30	20	40	20	30	28.89	**Fair**
Higueras et al. (2006)	30	20	30	20	30	20	20	20	40	25.56	**Fair**
Hill et al. (2017)	30	20	30	30	30	20	30	30	30	27.78	**Fair**
Holmes et al. (2007)	30	20	30	30	40	10	30	20	40	27.78	**Fair**
Holmes, Kennedy & Perrorn (2004)	40	20	40	20	40	40	40	20	40	33.33	**Fair**
Holmes, Murray & Knack (2015)	30	20	30	20	40	30	40	20	40	30.00	**Fair**
Holth et al. (2018)	40	20	30	20	30	20	40	20	30	27.78	**Fair**
Hottinen et	30	40	40	20	40	20	20	20	40	30.00	**Fair**

For peer review only

al. (2012)											
Hotzy et al (2019)	30	30	30	20	30	20	30	20	40	27.78	**Good**
Hughes et al. (2018)	30	20	30	30	30	20	30	30	40	28.89	**Fair**
Huizing et al. (2006)	40	30	40	30	40	30	30	30	40	34.44	**Fair**
Hunt et al (2016)	40	30	20	30	40	20	40	30	20	30.00	**Good**
Hunt et al. (2010)	40	20	40	30	40	20	40	40	30	33.33	**Fair**
Hunt et al. (2012)	30	20	40	30	40	10	40	30	30	30.00	**Fair**
Hvidhjelm et al (2016)	30	20	40	30	40	30	40	30	30	32.22	**Good**
Hylén et al. (2019)	20	20	30	10	40	30	40	20	30	26.67	**Fair**
Ilkiw-Lavalle & Grenyer (2003)	30	20	30	20	30	30	30	20	40	27.78	**Fair**
Inoue et al. (2017)	30	20	30	20	30	20	40	20	20	25.56	**Fair**
Ireland et al (2019)	20	20	30	20	10	20	30	20	30	22.22	**Fair**
Ireland et al. (2019)	30	40	30	20	40	20	30	20	40	30.00	**Good**
Ireland, Halpin & Sullivan (2014)	30	30	40	20	30	20	30	20	30	27.78	**Fair**
Isaak et al. (2016)	30	40	30	20	30	10	30	20	40	27.78	**Fair**
Jacob et al. (2013)	30	20	40	20	20	20	30	20	40	26.67	**Fair**
Jaeger et al. (2014)	30	30	40	10	40	20	30	20	30	27.78	**Fair**
Jalil et al. (2017)	40	40	30	30	40	20	40	30	40	34.44	**Good**
James et al. (2017)	40	20	30	40	40	20	30	40	30	32.22	**Good**
Janicki (2009)	30	20	30	30	40	30	40	40	40	33.33	**Fair**

For peer review only

Jeffs et al. (2012)	30	30	40	30	40	40	40	40	40	36.67	**Good**
Johnson & Delaney (2006)	40	20	30	20	40	10	40	20	40	28.89	**Fair**
Johnston & Kilty (2016)	20	30	20	10	10	20	40	20	10	20.00	**Fair**
Jones et al. (2010)	30	20	40	20	40	20	40	20	40	30.00	**Fair**
Jonker et al. (2008)	30	30	30	20	40	20	40	20	40	30.00	**Fair**
Kalagi et al. (2018)	40	30	30	30	40	30	40	30	30	33.33	**Good**
Kanerva et al. (2015)	30	20	40	20	40	20	40	20	40	30.00	**Fair**
Kanerva, Lammintakanen & Kivinen (2016)	30	20	40	20	40	20	40	20	30	28.89	**Fair**
Keers et al. (2018)	40	40	40	30	40	30	30	30	30	34.44	**Good**
Kelly et al. (2016)	20	20	40	20	30	20	30	20	40	26.67	**Fair**
Kelly et al. (2017)	30	30	40	30	30	20	40	30	40	32.22	**Good**
Keski-Valkama et al. (2007)	30	20	40	30	40	20	40	20	30	30.00	**Fair**
Killick & Allen (2005)	30	20	40	20	30	10	20	20	20	23.33	**Poor**
Kirkevold & Engedal (2004)	30	20	40	30	10	20	30	40	30	27.78	**Fair**
Knowles, Hearne & Smith (2015)	30	20	30	30	40	20	40	30	40	31.11	**Fair**
Kontio et al. (2010)	30	30	30	20	40	20	30	20	40	28.89	**Fair**
Kontio et al. (2009)	30	20	30	30	40	20	40	30	40	31.11	**Fair**
Kontio et al.	40	20	40	20	40	20	40	20	40	31.11	**Fair**

For peer review only

(2011)											
Kontio et al. (2012)	30	20	40	30	40	20	40	40	40	33.33	**Fair**
Kool et al. (2014)	40	20	30	20	40	20	40	20	40	30.00	**Fair**
Koukia, Madianos & Katostaras (2009)	30	20	40	20	40	20	20	20	40	27.78	**Fair**
Koukia et al. (2010)	30	20	40	20	40	20	30	20	40	28.89	**Fair**
Koukia et al. (2013)	30	20	40	30	40	10	30	20	40	28.89	**Fair**
Krieger et al. (2018)	30	30	40	30	40	10	40	30	40	32.22	**Good**
Kulkarni et al. (2014)	30	20	30	20	40	20	20	20	40	26.67	**Fair**
Kuosmanen et al. (2013)	30	30	30	20	40	20	30	30	30	28.89	**Fair**
Kuosmanen et al. (2015)	30	20	30	10	10	10	20	20	30	20.00	**Poor**
Kuosmanen et al. (2019)	40	40	30	30	40	20	40	30	40	34.44	**Good**
Lamanna et al. (2016)	40	20	30	20	30	10	40	20	30	26.67	**Fair**
Langan & McDonald (2008)	30	20	30	30	20	20	40	20	30	26.67	**Fair**
Lantta et al. (2015)	20	20	20	10	20	20	20	20	40	21.11	**Poor**
Lantta et al. (2016)	40	20	40	20	30	20	40	20	30	28.89	**Fair**
Lanza et al. (2009)	20	20	40	20	40	20	30	20	40	27.78	**Fair**
Lanza et al. (2016)	20	20	20	10	20	10	20	20	20	17.78	**Poor**
Larsen & Terkelsen (2014)	30	40	40	20	40	40	40	20	30	33.33	**Fair**
Larue et al. (2013)	30	20	40	30	40	30	40	30	30	32.22	**Fair**
Larue et al.	30	40	30	20	10	20	30	20	40	26.67	**Fair**

For peer review only

(2016)											
Lavelle et al. (2017)	40	30	30	20	20	20	40	20	40	28.89	**Fair**
Lawn & Pols (2003)	30	20	20	20	20	10	20	20	30	21.11	**Poor**
Lee et al. (2001)	30	20	20	20	10	10	20	20	30	20.00	**Poor**
Lee et al. (2003)	20	10	20	10	20	10	20	10	30	16.67	**Poor**
Lehmann, McCormick & Kizer (1999)	40	20	20	20	10	10	20	20	30	21.11	**Poor**
Li et al. (2019)	30	30	30	20	30	20	40	20	30	27.78	**Fair**
Lindgren, Aminoff & Graneheim (2015)	30	20	40	20	40	20	40	20	30	28.89	**Fair**
Lindsey (2009)	30	20	40	30	40	20	40	30	40	32.22	**Fair**
Lipscomb et al. (2012)	30	20	20	20	40	10	30	20	40	25.56	**Fair**
Long et al. (2014)	30	20	20	20	40	20	30	20	40	26.67	**Fair**
Looi, Engstro m & Savenstedt (2015)	30	20	30	10	40	20	40	10	30	25.56	**Fair**
Lovell, Smith & Johnson. (2015)	40	20	40	20	40	40	40	20	30	32.22	**Fair**
Lowe, Wellman & Taylor. (2003)	30	20	30		40	20	30	30	40	30.00	**Fair**
Lundegaard Mattson & Binder (2012)	40	30	30	20	40	40	40	20	30	32.22	**Good**
Mackay, Paterson &	30	30	40	30	30	10	40	40	40	32.22	**Fair**

For peer review only

Cassells (2005)											
Maguire, Daffern & Martin (2014)	30	20	40	20	40	20	40	20	40	30.00	**Fair**
Mahoney et al. (2012)	30	30	20	10	20	20	20	20	20	21.11	**Poor**
Mann-Poll et al. (2011)	30	40	30	20	40	10	30	20	40	28.89	**Fair**
Marangos-Frost & Wells (2000)	30	30	40	20	40	20	40	20	40	31.11	**Fair**
Martello et al. (2018)	30	30	30	20	40	20	40	20	40	30.00	**Good**
Martin & Daffern (2006)	30	40	40	20	20	20	40	20	40	30.00	**Fair**
Mason & Whitehead (2001)	30	40	30	20	20	10	30	20	40	26.67	**Fair**
McCann, Baird & Muir-Cochrane (2014)	30	20	40	20	30	20	30	20	40	27.78	**Fair**
McKeown et al. (2019)	40	30	30	20	40	30	40	20	30	31.11	**Good**
McLaughlin et al. (2010)	20	20	20	20	20	10	30	20	40	#REF!	**Poor**
Meaden, Ha cker & Spencer (2013)	30	20	40	20	40	20	40	20	40	30.00	**Fair**
Meehan, McIntosh & Bergen. (2006)	30	20	40	20	40	20	40	20	30	28.89	**Fair**
Meehan, Morrison & McDougall. (1999)	30	20	30	20	20	10	30	20	40	24.44	**Poor**

For peer review only

Mezey, Hassell & Bartlett (2005)	30	20	40	30	40	20	40	40	40	33.33	**Fair**
Millar & Sands (2013)	30	40	40	30	40	20	40	40	40	35.56	**Good**
Mistler et al. (2017)	30	30	30	20	30	20	40	20	40	28.89	**Fair**
Molewijk et al (2017)	40	40	40	20	40	30	30	20	30	32.22	**Good**
Muir-Cochrane et al. (2012)	30	20	40	20	40	20	40	20	40	30.00	**Fair**
Muir-Cochrane et al. (2013)	30	20	30	20	40	20	40	20	20	26.67	**Fair**
Muir-Cochrane et al. (2015)	40	20	40	20	40	20	40	20	40	31.11	**Fair**
Murphy & Siv (2007)	20	20	20	30	20	20	30	30	30	24.44	**Fair**
Nathan et al. (2007)	30	40	30	20	30	30	30	20	30	28.89	**Fair**
Needham et al. (2004)	30	20	30	30	30	40	40	30	30	31.11	**Good**
Needham et al. (2005)	30	20	30	20	30	20	40	20	20	25.56	**Fair**
Newman et al. (2018)	30	30	30	10	30	30	30	20	40	27.78	**Fair**
Nielsen et al. (2018)	30	20	40	20	30	20	40	20	30	27.78	**Fair**
Nijman et al. (2011)	30	30	20	30	40	20	40	30	20	28.89	**Fair**
Nijman et al. (2005)	30	30	30	20	40	20	40	20	30	28.89	**Fair**
Niu et al. (2019)	40	30	40	30	40	30	40	30	30	34.44	**Good**
Nolan et al. (2009)	30	20	20	20	30	30	40	30	30	27.78	**Fair**
Nurjannah, FitzGerald & Foster	20	20	30	20	40	30	40	20	40	28.89	**Fair**

For peer review only

(2009)											
O'Donovan (2007)	20	20	30	20	30	30	30	20	30	25.56	**Fair**
O'Brien & Cole (2004)	20	20	30	20	40	30	40	20	30	27.78	**Fair**
Olsson & Schon (2016)	30	20	30	20	30	20	40	20	30	26.67	**Fair**
Olsson et al. (2015)	30	30	40	30	40	30	40	30	20	28.89	**Fair**
O'Neill et al. (2003)	30	30	30	20	30	20	30	20	20	25.56	**Fair**
Papadopoulos et al. (2012)	30	30	30	30	30	20	40	30	40	31.11	**Good**
Park & Lee (2012)	20	30	30	30	30	20	40	20	40	28.89	**Fair**
Paschali et al. (2017)	30	20		20	40	20	20	20	30	25.00	**Fair**
Pekurinen et al (2017)	40	30	40	20	40	20	40	20	40	32.22	**Good**
Pellfolk et al. (2010)	40	20	30	30	30	20	40	30	30	30.00	**Good**
Pelto-Piri et al. (2019)	40	30	40	20	40	20	40	20	30	31.11	**Good**
Perkins et al. (2012)	20	20	20	20	40	20	30	20	20	23.33	**Fair**
Pfeiffer et al. (2019)	30	40	30	30	20	20	30	30	30	28.89	**Fair**
Phillips (2011)	20	30	20	20	30	20	30	20	30	24.44	**Fair**
Podubinski et al. (2017)	40	40	40	40	40	40	40	40	30	38.89	**Good**
Powell-Cope et al. (2014)	40	30	30	20	30	30	40	20	40	31.11	**Good**
Price et al. (2018)	40	20	30	20	40	20	40	20	30	28.89	**Fair**
Prins et al. (2013)	40	20	40	20	30	40	40	20	20	30.00	**Good**
Quirk, et al. (2005)	40	30	30	10	20	20	30	20	30	25.56	**Fair**
Raboch et al.	30	20	30	30	30	30	30	30	30	28.89	**Fair**

For peer review only

(2010)											
Raveendran athan, Chandra & Chaturvedi (2012)	30	20	30	30	10	20	30	30	30	25.56	**Fair**
Raveesh et al (2019)	40	30	30	40	30	20	40	40	30	33.33	**Good**
Rees & Manthorpe (2010)	30	20	30	20	20	30	30	20	30	25.56	**Fair**
Reininghaus et al. (2007)	20	10	30	20	30	20	30	20	30	23.33	**Fair**
Reisch et al (2018)	30	20	30	20	30	20	20	20	30	24.44	**Fair**
Reynolds et al. (2016)	30	30	30	20	10	10	30	20	30	23.33	**Fair**
Rippon et al (2018)	30	30	20	10	40	10	40	20	20	24.44	**Fair**
Rodreguez-Leal et al. (2016)	30	20	30	40	20	10	30	30	30	26.67	**Fair**
Ružić et al. (2011)	20	20	30	20	30	20	40	20	20	24.44	**Fair**
Ryan & Bowers (2005)	30	30	40	20	30	20	30	20	30	27.78	**Fair**
Ryan & Happell (2009)	30	20	30	30	30	30	40	30	30	30.00	**Good**
Ryan et al. (2004)	40	20	30	30	30	20	40	30	20	28.89	**Fair**
Ryan et al. (2007)	30	30	30	30	40	20	30	30	20	28.89	**Fair**
Salzmann-Erikson et al. (2008)	30	20	30	20	40	20	40	20	20	26.67	**Fair**
Sandy (2016)	40	30	40	40	20	30	30	20	30	31.11	**Good**
Sansone, McLean & Wiederman	30	10	30	40	20	20	30	30	20	25.56	**Fair**

For peer review only

(2008)											
Sato et al. (2017)	30	30	30	30	40	20	40	30	30	31.11	**Good**
Schreiner, Crafton & Sevin (2004)	20	10	20	20	30	30	30	20	30	23.33	**Fair**
Schwartz & Park (1999)	40	30	20	20	20	20	40	20	20	25.56	**Fair**
Seemüller et al. (2009)	40	40	30	30	40	40	40	30	20	34.44	**Good**
Selenius & Strand (2017)	30	30	40	30	40	20	40	30	30	32.22	**Good**
Seo, Kim & Rhee (2012)	30	20	20	20	20	10	20	10	20	18.89	**Poor**
Sequeira & Halstead (2001)	20	10	20	20	20	30	30	20	30	22.22	**Fair**
Sequeira & Halstead (2004)	30	20	20	20	40	30	40	20	30	27.78	**Fair**
Shaw & Sandy (2016)	30	40	40	40	20	20	30	30	30	31.11	**Good**
Silvana et al. (2012)	20	20	30	10	20	20	30	10	20	20.00	**Fair**
Simpson et al. (2011)	30	20	30	10	40	30	30	10	30	25.56	**Fair**
Simpson et al. (2015)	40	30	30	20	40	30	40	20	40	32.22	**Good**
Sival et al. (2000)	40	20	30	20	40	30	40	20	20	28.89	**Fair**
Sjöstrand et al. (2015)	40	20	30	20	40	20	40	20	20	27.78	**Fair**
Sjöström et al. (2001)	10	10	30	20	30	30	40	20	30	24.44	**Fair**
Sjöström, Hetta & Waern (2012)	30	30	30	30	30	20	40	30	30	30.00	**Good**
Skovdahl, Kihlgren &	20	10	40	20	40	40	30	20	30	27.78	**Fair**

For peer review only

Kihlgren (2003)											
Smith & Jones (2014)	20	20	20	20	30	30	40	20	30	25.56	**Fair**
Sørensen et al. (2013)	30	20	30	30	40	30	40	30	20	30.00	**Fair**
Speziale et al. (2009)	30	20	30	20	20	30	40	20	30	26.67	**Fair**
Spokes et al. (2002)	20	20	30	30	20	20	30	30	30	25.56	**Fair**
Stead et al. (2009)	30	20	30	20	20	20	30	20	30	24.44	**Fair**
Stein (2002)	20	30	30	20	30	30	30	20	30	26.67	**Fair**
Steinert et al. (2007)	30	20	30	10	30	40	30	10	30	25.56	**Fair**
Stevenson, Jack, O'Mara & LeGris (2015)	40	30	40	30	30	40	40	30	30	34.44	**Good**
Stolker, Nijman & Zwanikken (2006)	30	30	30	30	30	30	40	30	30	31.11	**Good**
Stone et al. (2011)	30	30	30	20	40	20	40	20	30	28.89	**Fair**
Strudwick et al. (2017)	30	30	30	20	40	30	40	20	40	31.11	**Good**
Stübner et al. (2006)	40	20	30	20	20	20	30	20	30	25.56	**Fair**
Sukhodolsky, Cardona & Martin (2005)	30	30	30	30	40	20	40	30	30	31.11	**Good**
Sun et al. (2005)	30	20	30	20	40	20	30	20	30	26.67	**Fair**
Sun et al. (2006)	30	30	30	20	40	30	40	20	20	28.89	**Fair**
Sustere & Tarpey (2019)	20	40	30	10	30	20	40	20	20	25.56	**Fair**
Sutton et al. (2013)	30	20	20	30	40	20	30	30	30	27.78	**Fair**

For peer review only

Swogger, Van Orden & Conner (2014)	20	20	30	30	30	0	30	30	30	24.44	**Fair**
Takahashi et al. (2011)	30	20	30	20	30	30	40	20	20	26.67	**Fair**
Tängman et al. (2010)	30	20	30	20	30	20	40	20	20	25.56	**Fair**
Tateno et al. (2009)	40	20	30	20	20	20	40	20	20	25.56	**Fair**
Tema, Poggenpoel & Myburgh (2011)	30	30	20	20	30	30	40	20	30	27.78	**Fair**
Terkelsen & Larsen (2016)	30	40	30	20	30	40	40	20	30	31.11	**Good**
Thomas & Haslam (2017)	30	30	30	10	30	30	40	10	40	27.78	**Fair**
Tofthagen, Talseth & Fagerström (2014)	30	30	30	20	30	20	40	20	20	26.67	**Fair**
Tomagova et al (2016)	30	40	40	20	30	20	30	20	40	30.00	**Good**
Tompsett, Domoff & Boxer (2011)	40	30	30	30	30	20	30	30	30	30.00	**Good**
Trenoweth (2003)	30	20	20	20	30	30	40	20	30	26.67	**Fair**
Triplett et al. (2017)	40	30	30	10	40	20	40	20	40	30.00	**Good**
Truea et al. (2017)	30	30	30	40	30	20	30	30	30	30.00	**Good**
Umut et al. (2012)	30	20	20	20	30	30	40	20	20	25.56	**Fair**
Vahidi et al. (2018)	30	30	40	30	30	30	30	30	30	31.11	**Good**
Van de Sande et al. (2011)	40	20	30	20	40	40	40	20	30	31.11	**Good**

For peer review only

van den Bogaard et al. (2018)	30	30	40	20	10	20	30	20	30	25.56	**Fair**
Van der Schaaf et al. (2013)	30	30	40	30	40	10	40	30	30	31.11	**Good**
Van Wijk, Traut & Julie (2014)	30	20	30	30	30	30	30	30	30	28.89	**Fair**
Vandewalle et al. (2018)	30	40	40	30	40	40	30	30	30	34.44	**Good**
Vandewalle et al. (2019)	40	20	30	20	40	30	40	20	30	30.00	**Good**
Vedana et al (2018)	30	20	20	20	20	20	20	20	30	22.22	**Fair**
Verbeek et al. (2014)	40	20	40	30	30	30	30	30	30	31.11	**Good**
Vlayen et al. (2012)	40	30	30	20	30	30	30	20	40	30.00	**Good**
Vråle & Steen (2005)	20	20	20	20	30	30	40	20	30	25.56	**Fair**
Ward (2013)	20	30	20	20	40	30	30	20	30	26.67	**Fair**
Weber (2002)	30	20	20	30	40	10	30	30	20	25.56	**Fair**
Whaley (2001)	20	20	20	20	20	10	30	20	30	21.11	**Fair**
Whaley (2004)	20	20	20	30	20	10	20	30	30	22.22	**Fair**
Wharewera-Mika et al. (2016)	30	20	20	10	20	20	40	20	30	23.33	**Fair**
Whitecross, Seeary & Lee (2013)	40	20	30	20	30	20	40	20	30	27.78	**Fair**
Whittington et al. (2009)	40	20	20	20	20	20	20	20	20	22.22	**Fair**
Wilkes et al. (2005)	30	20	20	30	20	20	30	30	30	25.56	**Fair**
Wilson et al (2018)	40	30	30	20	40	30	40	20	30	31.11	**Good**
Wilson et al. (2017)	30	30	30	20	30	20	20	30	30	26.67	**Fair**

For peer review only

Wood & Pistrang (2004)	30	20	20	20	30	0	40	20	30	23.33	**Fair**
Woods (2013)	20	20	30	20	30	30	40	20	30	26.67	**Fair**
Wright et al. (2005)	40	30	30	30	0	30	40	30	30	28.89	**Fair**
Wright et al. (2014)	10	20	10	20	10	20	30	20	30	18.89	**Poor**
Wu et al. (2015)	30	30	30	20	40	30	40	20	40	31.11	**Good**
Wystanski (2000)	30	10	20	30	20	0	30	20	10	18.89	**Poor**
Xie et al. (2019)	30	30	40	20	40	20	40	20	30	30.00	**Good**
Yang et al. (2007)	30	20	40	20	30	20	30	20	30	26.67	**Fair**
Yip et al. (2013)	40	30	30	30	40	30	40	30	30	33.33	**Good**
Zuzelo, Curran & Zeserman (2012)	30	20	30	20	40	30	40	20	40	30.00	**Good**
Zwijsen et al. (2014)	40	30	40	30	40	40	40	30	40	36.67	**Good**

For peer review only

Online supplement 6 – Forrest plots

Prevalence of physical aggression

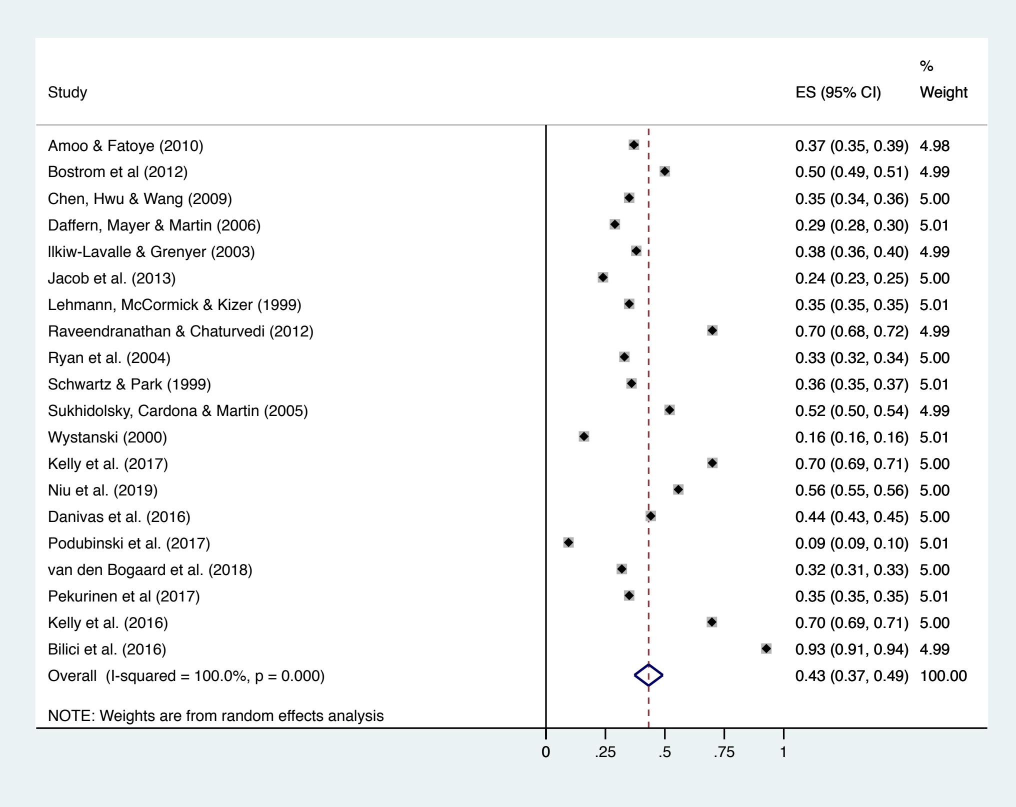

Prevalence of verbal aggression

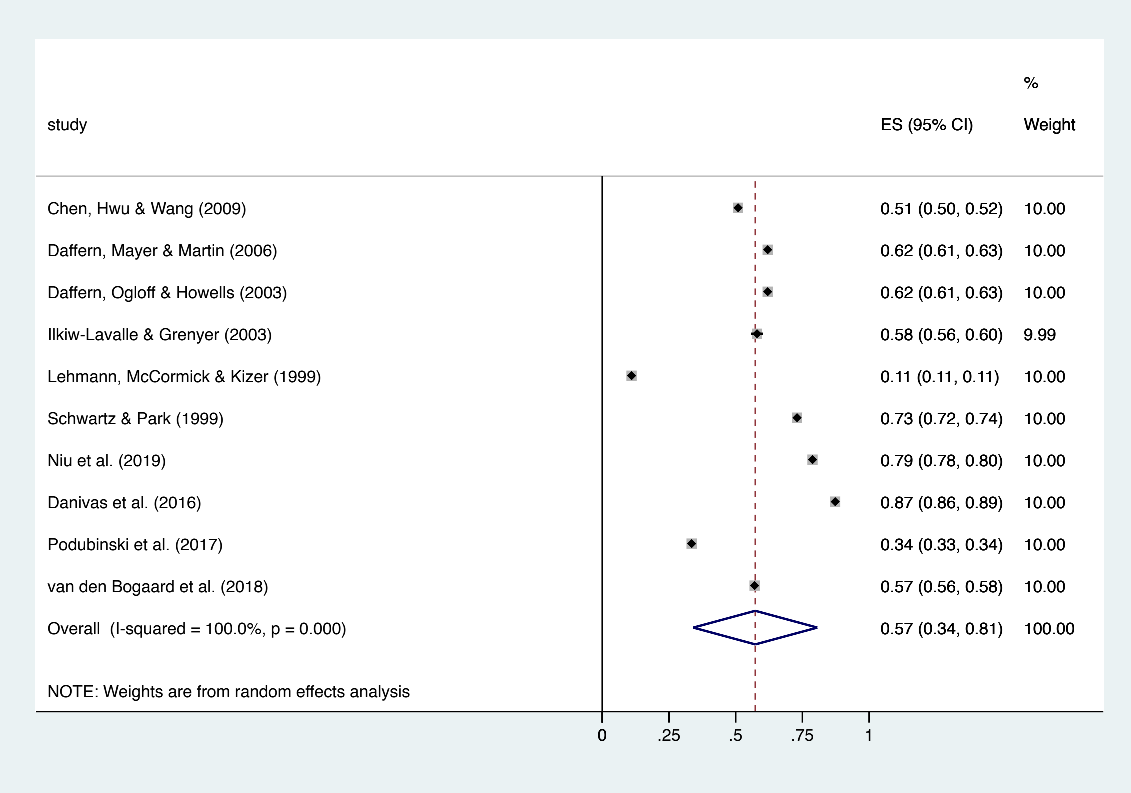

For peer review only - http://bmjopen.bmj.com/site/about/guidelines.xhtml

Use of coercive interventions

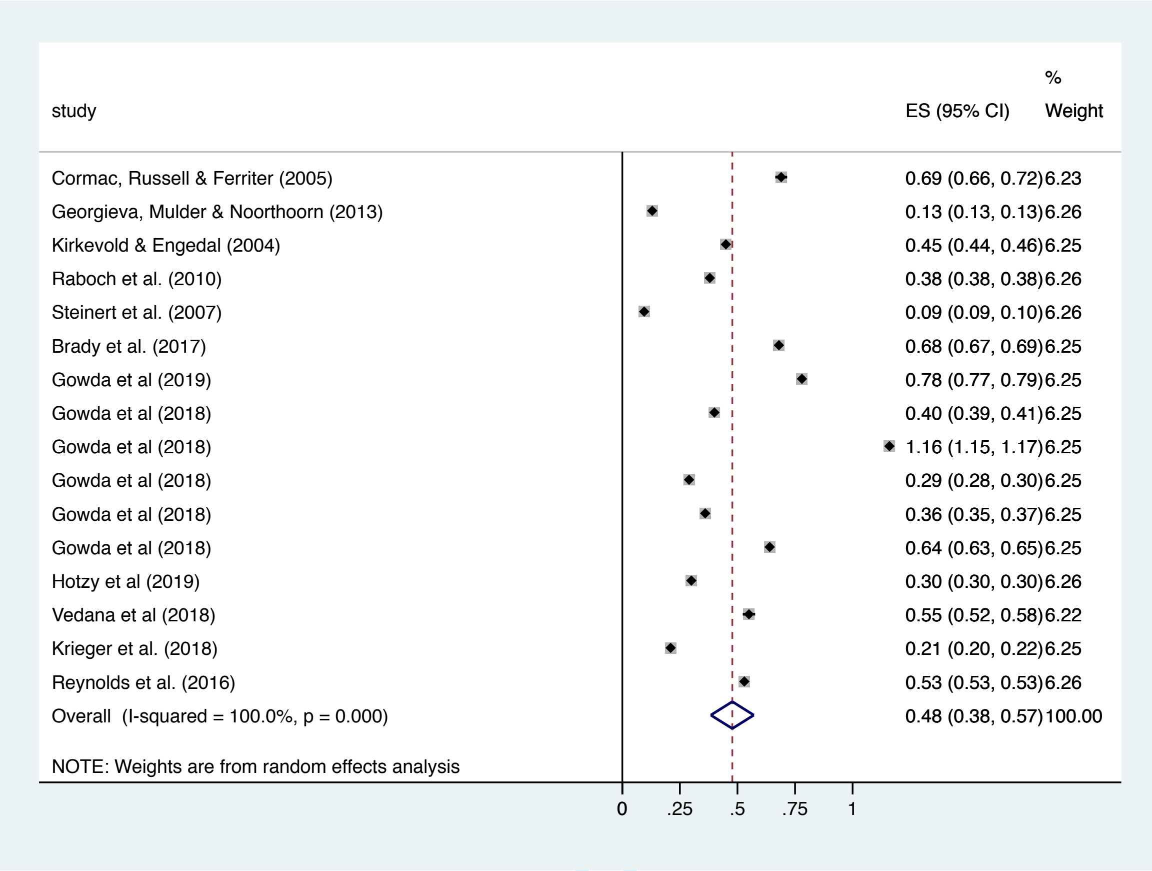

Prevalence of wandering behaviour

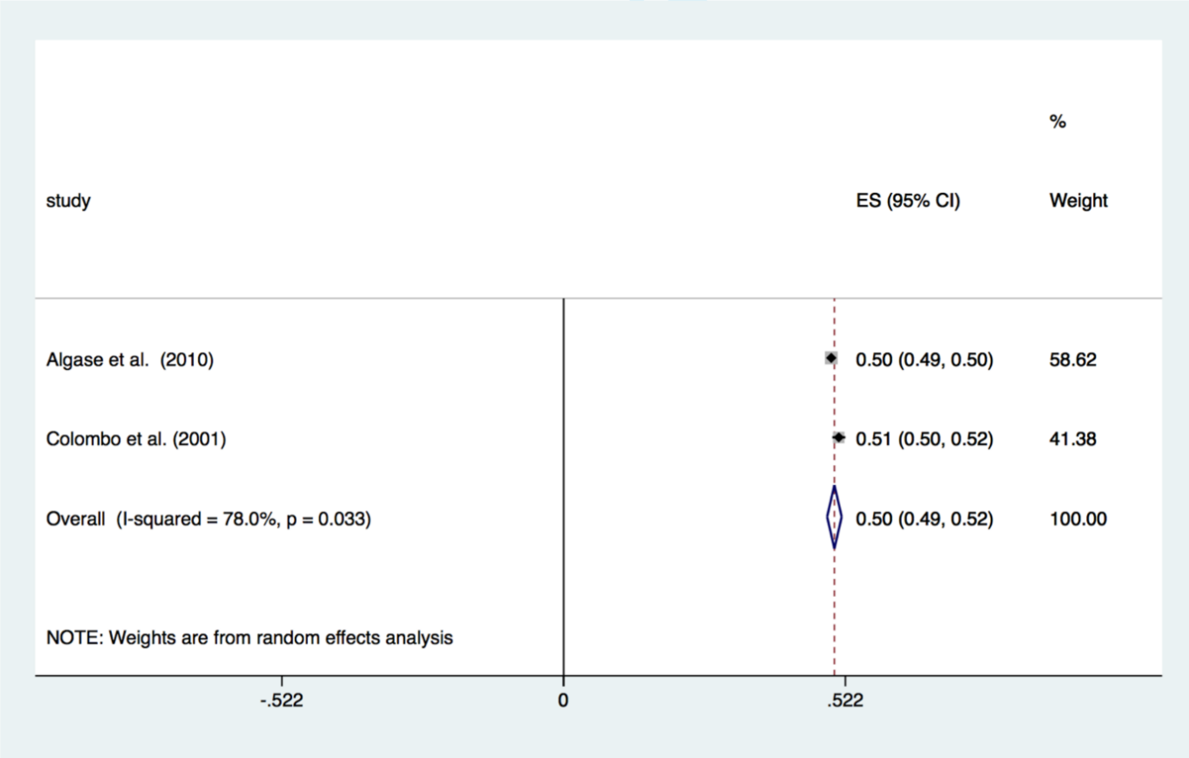

PRISMA 2009 Checklist

Section/topic	#	Checklist item	Reported on page #
**TITLE**			
Title	1	Identify the report as a systematic review, meta-analysis, or both.	1
**ABSTRACT**			
Structured summary	2	Provide a structured summary including, as applicable: background; objectives; data sources; study eligibility criteria, participants, and interventions; study appraisal and synthesis methods; results; limitations; conclusions and implications of key findings; systematic review registration number.	2
**INTRODUCTION**			
Rationale	3	Describe the rationale for the review in the context of what is already known.	4
Objectives	4	Provide an explicit statement of questions being addressed with reference to participants, interventions, comparisons, outcomes, and study design (PICOS).	4
**METHODS**			
Protocol and registration	5	Indicate if a review protocol exists, if and where it can be accessed (e.g., Web address), and, if available, provide registration information including registration number.	4
Eligibility criteria	6	Specify study characteristics (e.g., PICOS, length of follow-up) and report characteristics (e.g., years considered, language, publication status) used as criteria for eligibility, giving rationale.	4,5
Information sources	7	Describe all information sources (e.g., databases with dates of coverage, contact with study authors to identify additional studies) in the search and date last searched.	4
Search	8	Present full electronic search strategy for at least one database, including any limits used, such that it could be repeated.	Online Supplement 2
Study selection	9	State the process for selecting studies (i.e., screening, eligibility, included in systematic review, and, if applicable, included in the meta-analysis).	5,6
Data collection process	10	Describe method of data extraction from reports (e.g., piloted forms, independently, in duplicate) and any processes for obtaining and confirming data from investigators.	6
Data items	11	List and define all variables for which data were sought (e.g., PICOS, funding sources) and any assumptions and simplifications made.	5,6
Risk of bias in individual studies	12	Describe methods used for assessing risk of bias of individual studies (including specification of whether this was done at the study or outcome level), and how this information is to be used in any data synthesis.	n/a
Summary measures	13	State the principal summary measures (e.g., risk ratio, difference in means).	5, Online Supplement 3

For peer review only - http://bmjopen.bmj.com/site/about/guidelines.xhtml

PRISMA 2009 Checklist

Synthesis of results	14	Describe the methods of handling data and combining results of studies, if done, including measures of consistency (e.g., I^2) for each meta-analysis.	5,6

Section/topic	#	Checklist item	Reported on page #
Risk of bias across studies	15	Specify any assessment of risk of bias that may affect the cumulative evidence (e.g., publication bias, selective reporting within studies).	n/a
Additional analyses	16	Describe methods of additional analyses (e.g., sensitivity or subgroup analyses, meta-regression), if done, indicating which were pre-specified.	n/a
**RESULTS**			
Study selection	17	Give numbers of studies screened, assessed for eligibility, and included in the review, with reasons for exclusions at each stage, ideally with a flow diagram.	7
Study characteristics	18	For each study, present characteristics for which data were extracted (e.g., study size, PICOS, follow-up period) and provide the citations.	7,8
Risk of bias within studies	19	Present data on risk of bias of each study and, if available, any outcome level assessment (see item 12).	n/a
Results of individual studies	20	For all outcomes considered (benefits or harms), present, for each study: (a) simple summary data for each intervention group (b) effect estimates and confidence intervals, ideally with a forest plot.	9-13
Synthesis of results	21	Present results of each meta-analysis done, including confidence intervals and measures of consistency.	6-13
Risk of bias across studies	22	Present results of any assessment of risk of bias across studies (see Item 15).	n/a
Additional analysis	23	Give results of additional analyses, if done (e.g., sensitivity or subgroup analyses, meta-regression [see Item 16]).	n/a
**DISCUSSION**			
Summary of evidence	24	Summarize the main findings including the strength of evidence for each main outcome; consider their relevance to key groups (e.g., healthcare providers, users, and policy makers).	13-15
Limitations	25	Discuss limitations at study and outcome level (e.g., risk of bias), and at review-level (e.g., incomplete retrieval of identified research, reporting bias).	15,16
Conclusions	26	Provide a general interpretation of the results in the context of other evidence, and implications for future research.	16,17
**FUNDING**			
Funding	27	Describe sources of funding for the systematic review and other support (e.g., supply of data); role of funders for the systematic review.	17

From: Moher D, Liberati A, Tetzlaff J, Altman DG, The PRISMA Group (2009). Preferred Reporting Items for Systematic Reviews and Meta-Analyses: The PRISMA Statement. PLoS Med 6(7): e1000097. doi:10.1371/journal.pmed1000097 For more information, visit: **www.prisma-statement.org**.

For peer review only - http://bmjopen.bmj.com/site/about/guidelines.xhtml
